# Design and Synthesis of the Active Site Environment in Zeolite Catalysts for Selectively Manipulating Mechanistic Pathways

**DOI:** 10.1021/jacs.1c04818

**Published:** 2021-07-09

**Authors:** Chengeng Li, Pau Ferri, Cecilia Paris, Manuel Moliner, Mercedes Boronat, Avelino Corma

**Affiliations:** Instituto de Tecnología Química, Universitat Politècnica de València - Consejo Superior de Investigaciones Científicas, Avenida de los Naranjos s/n, 46022 Valencia, Spain

## Abstract

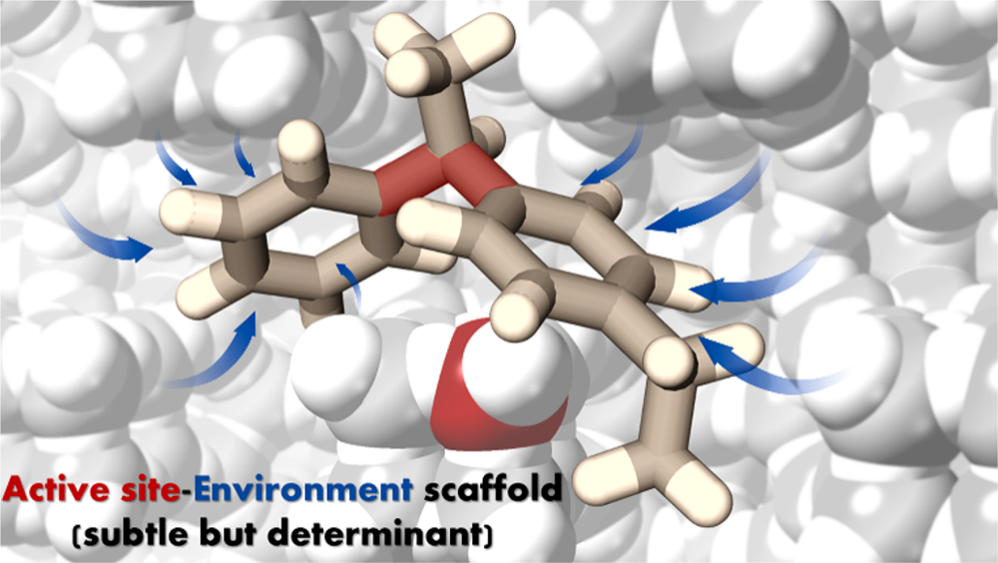

By combining kinetics and theoretical calculations, we show here the benefits of going beyond the concept of static localized and defined active sites on solid catalysts, into a system that globally and dynamically considers the active site located in an environment that involves a scaffold structure particularly suited for a target reaction. We demonstrate that such a system is able to direct the reaction through a preferred mechanism when two of them are competing. This is illustrated here for an industrially relevant reaction, the diethylbenzene–benzene transalkylation. The zeolite catalyst (ITQ-27) optimizes location, density, and environment of acid sites to drive the reaction through the preselected and preferred diaryl-mediated mechanism, instead of the alkyl transfer pathway. This is achieved by minimizing the activation energy of the selected pathway through weak interactions, much in the way that it occurs in enzymatic catalysts. We show that ITQ-27 outperforms previously reported zeolites for the DEB-Bz transalkylation and, more specifically, industrially relevant zeolites such as faujasite, beta, and mordenite.

## 1 Introduction

Production of alkylaromatics is a crucial process in the modern chemical industry since they are well-established precursors for many important intermediates and chemicals.^1−3^ Ethylbenzene (EB) is one of the industrial alkylaromatics with a higher production capacity worldwide, which is mostly consumed in the manufacture of polystyrene.^4^ Alkylaromatics are mainly produced by alkylation of benzene (Bz) with alkyl reagents such as light olefins and alcohols using acid zeolites as catalysts.^5−7^ However, the formation of undesired polyalkylated byproducts with lower added value is inevitably occurring during the industrial alkylation process.^8^ To improve selectivity, the current production of EB combines two reaction processes: alkylation of benzene with ethene, followed by EB separation and transalkylation of the polyethylated byproducts with benzene to increase the global yield of EB (Figure S1 in the Supporting Information).^9−11^

**Scheme 1:**
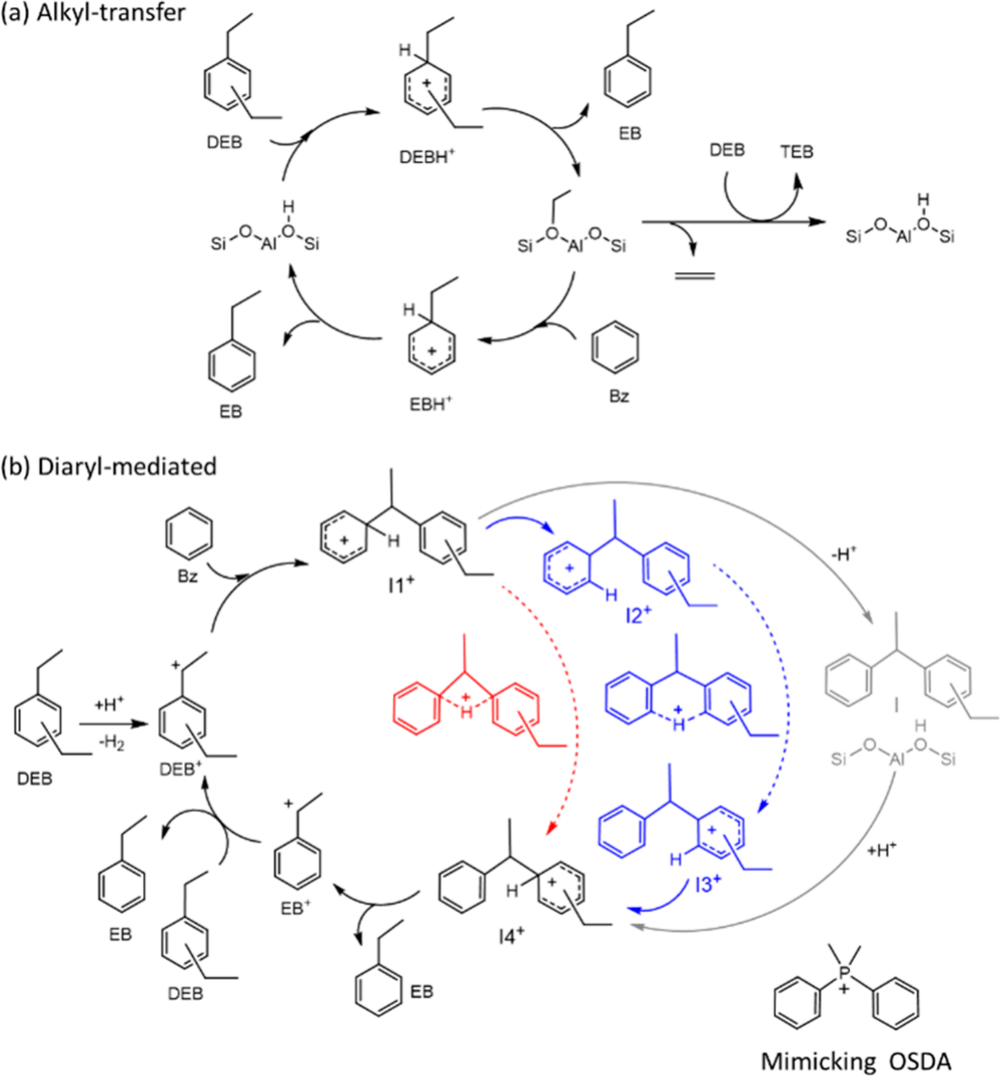
Proposed Mechanisms for the Diethylenzene–Benzene Transalkylation: (a) Alkyl-Transfer and (b) Diaryl-Mediated Pathways and the Proposed Mimicking OSDA

Some of the catalysts employed in the industrial transalkylation of diethylbenzene (DEB), which is the main byproduct of benzene ethylation, are large pore zeolites such as faujasite, beta, MCM-22, and mordenite.^9,10,12−14^ The presence of wide channels and/or cavities or hemicavities within these materials allows the diffusion of the polyethylated aromatic molecules involved in the transalkylation and the formation of the bulky diaryl intermediates. Diethylbenzene transalkylation can proceed through two main reaction pathways (Scheme 1).^15,16,25,17−24^ The alkyl transfer mechanism (Scheme 1a) involves consecutive dealkylation and alkylation steps that proceed through unstable penta-coordinated carbonium ion intermediates.^16,26^ The first dealkylation of DEB produces an EB molecule and a surface ethoxy group that, in a second step, reacts with benzene and yields EB. However, the ethoxy group can also react with EB or DEB molecules present in the zeolite channels to form undesired overethylated byproducts such as DEB and triethylbenzene (TEB).^27^ In addition, the surface ethoxy can decompose into ethene and regenerate the Brønsted acid site, further decreasing the yield of EB.

The second pathway proceeds through cationic diaryl intermediates formed by reaction of benzene with a diethylbenzenium carbenium ion (DEB^+^) (Scheme 1b).^16,28^ The initial hydride abstraction step necessary to form DEB^+^ from DEB is difficult on Brönsted acid sites, with high activation energy barriers of ∼200 kJ/mol,^22,25,29^ but it has been proposed that this step is energetically affordable on Lewis acid sites like La^3+^ cations^15,20^ or extra-framework Al species.^16,17^ Once the first DEB^+^ cation is generated, it enters the true catalytic cycle, at the end of which it is regenerated by hydrogen transfer between reactants and products. For the transalkylation to take place, a proton in the cationic I1^+^ intermediate must be transferred from one aromatic ring to the other, generating a different I4^+^ intermediate that, by cracking, produces EB. The proton transfer converting I1^+^ into I4^+^ can occur intramolecularly through four- or six-membered cationic transition states (red and blue paths in Scheme 1b) or via consecutive deprotonation and protonation steps with the participation of the zeolite acid sites (gray path in Scheme 1b). The formation of undesired higher-ethylated byproducts according to the diaryl-mediated pathway is less probable than in the alkyl transfer mechanism due to steric constraints. Therefore, in order to increase the yield of EB while decreasing catalyst deactivation during the transalkylation process, the participation of the diaryl-mediated mechanism must be maximized. To do that, we thought that the microporous structure of an optimized zeolite catalyst for EB transalkylation should be able to accommodate and stabilize the diaryl cationic intermediates and transition states.

Starting with this hypothesis, and in analogy with enzymatic catalysts, we should look for a zeolite structure with an adequate scaffold for the reaction to take place, while introducing the active site, a proton in this case, in the adequate position.^30−32^ Contrary to what happens in the case of enzymes, the zeolite structure is not so flexible during the reaction. Therefore, we must find a zeolite structure whose pores and cavities match the transition state for the reaction pathway selected. Indeed, electrostatic and, specifically, weak interactions between the walls and the transition state will decrease the activation energy of the transalkylation reaction through the preferred reaction mechanism, i.e., the diaryl-mediated pathway.

In order to achieve this, we applied a methodology for the design of zeolites involving the use of organic structure-directing agents (OSDAs) that mimic intermediates or transition states of the target reactions. This methodology includes the following working steps: (i) geometry optimization of the transition state or key intermediate of the reaction considered, (ii) synthesis of an OSDA that mimics the size, shape, and charge distribution of the transition state or intermediate, (iii) synthesis of zeolites using this OSDA, and (iv) catalyst testing of the zeolite or zeolites obtained to check if they efficiently catalyze the target reaction. Notice that in the third step of the process, that is, the zeolite synthesis, it is not possible to predict whether amorphous materials, an already existing zeolite, or a new zeolite structure will be obtained. Within our proposal, success does not necessarily imply that we obtained a new zeolite structure. Success implies that the zeolite obtained, regardless of if it is new or already known, is optimal for the specific reaction under study. In the case that the synthesized zeolite structure already exists, two possibilities arise: (i) the zeolite has already been applied in academia or industrially for the target reaction, in which case the validity of the transition state that mimics methodology to directly synthesize the optimal catalyst is confirmed, or (ii) the zeolite structure has never been claimed for the reaction under study, in which case the impact of the result is important from both fundamental and industrial points of view.

Here, following the working steps described above, we have selected an OSDA, diphenyldimethylphosphonium (DPDMP^+^), that mimics the size, shape, and charge localization of the diaryl cation intermediate involved in the transalkylation of DEB and benzene (see Scheme 1). With this OSDA, a zeolite (ITQ-27) has been synthesized which, as far as we know, was never reported for this reaction and outperforms other zeolites currently reported for the DEB-Bz transalkylation. We show here by means of kinetic and computational studies that not only the “imprinted” zeolite structure preferentially stabilizes the transition state involved in the diaryl-mediated pathway but also it favors, more than the other zeolites, this pathway with respect to the less desired alkyl transfer mechanism. All this results in a higher activity and selectivity and a lower rate of deactivation than the other zeolites that are used for DEB-Bz transalkylation.

## 2 Results and Discussion

### 2.1 Synthesis and Characterization

Based on the above exposed premises, the DPDMP^+^ mimicking the diaryl intermediates involved in the DEB-Bz transalkylation mechanism was used as OSDA, and the synthesis resulted in the selective crystallization of the ITQ-27 zeolite with the IWV framework (see details in the Supporting Information, Experimental Section). The achieved material, designated as IWV-M, shows the characteristic PXRD pattern of the IWV structure without impurities (Figure S2). Particles between 1 and 2 μm with plate-like morphology were observed by FESEM (Figure S3), and no extra-framework Al was observed by solid ^27^Al MAS NMR (Figure S4). The DEB-Bz transalkylation activity of IWV-M was tested in a fixed bed continuous reactor in gas as well as in the liquid phase and compared with that of catalysts currently employed in industry such as faujasite (FAU), beta (*BEA), MCM-22 (MWW), and mordenite (MOR).^9,10,12−14^

The reference catalysts used include two samples of FAU with different Si/Al ratio and a crystal size of 400–500 nm (CBV760 and CBV720), as well as a sample of beta zeolite (Zeolyst CP814C), with a Si/Al ratio of 16.4 composed by small crystallites with diameter below 100 nm (Table S1 and Figure S3). MOR and MWW samples with Si/Al ratios of 12.7 and 20.4, respectively, and crystallite sizes of 100 and 200 nm, respectively, were synthesized following the literature.^33,34^

### 2.2 Catalytic Test

**Figure 1:**
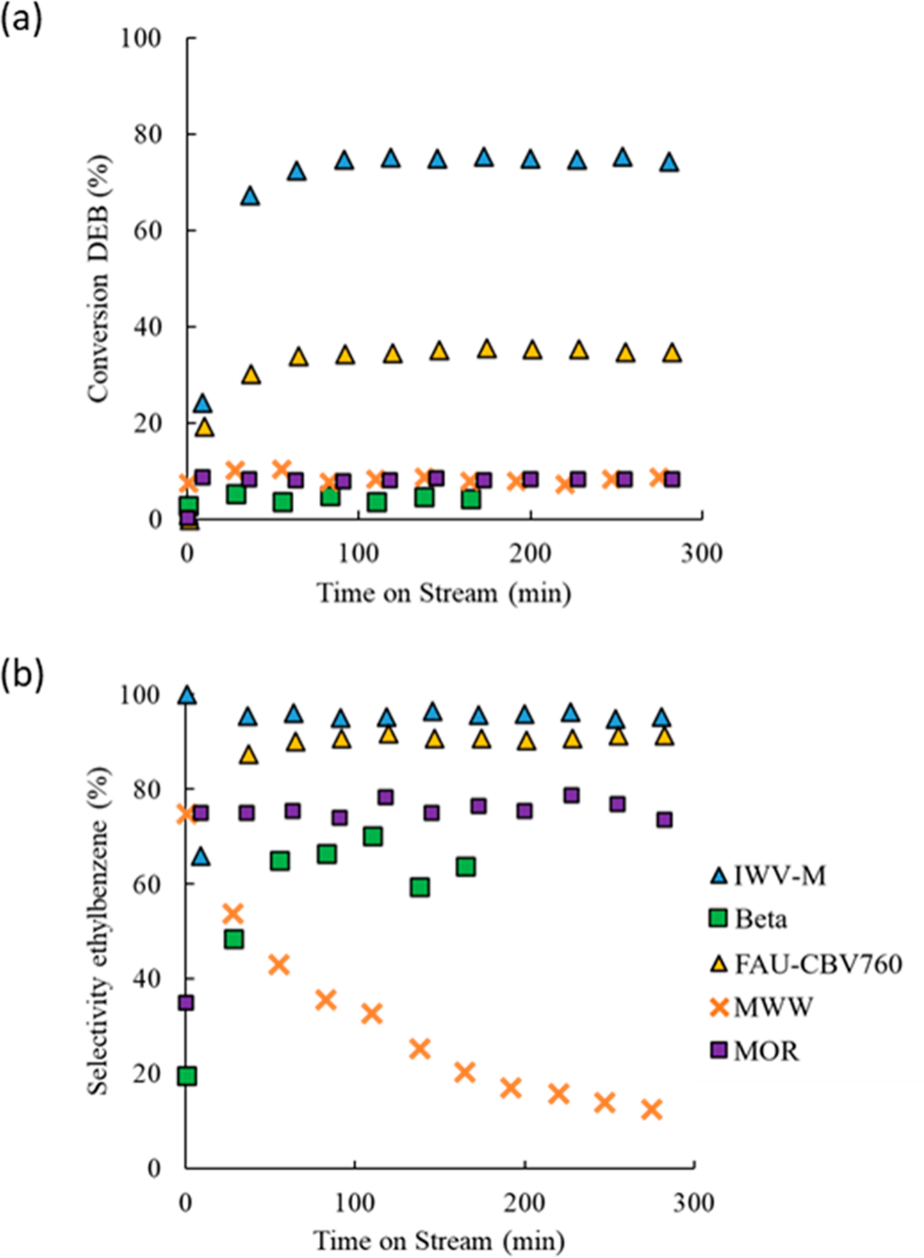
DEB conversion (a) and EB selectivity (b) with time on stream (TOS) for the transalkylation of DEB over zeolite catalysts with different framework topologies. Reaction conditions: *T* = 250 °C, *P* = 3.5 MPa, feeding composition: Bz:DEB = 3:1 (wt). WHSV_DEB_ = 10 h^–1^.

The materials were first tested in the liquid-phase DEB-Bz transalkylation at 250 °C and 3.5 MPa, with a DEB weight hourly space velocity (WHSV_DEB_) of 10 h^–1^ using a feedstock of reactant mixture (Bz/DEB ratio of 3:1 wt/wt), similar to the industrially employed conditions. With the catalyst particle size and flows selected, no control by either external or internal diffusion was observed (see details in the Supporting Information, Experimental Section). The results in Figure 1 show that IWV provides the highest activity and selectivity toward ethylbenzene (EB) under these reaction conditions compared with the different zeolite structures that have been reported for the transalkylation reaction. Thus, the steady-state DEB conversion with IWV-M approaches 75% with an EB selectivity higher than 95%, while the CBV760 catalyst, with the FAU structure and similar framework Si/Al ratio, achieved a lower steady-state DEB conversion of ∼26–28% and an EB selectivity of ∼90%.

The other materials tested, MOR and beta containing 12-ring channels and MWW with 10-ring channels and 12-ring cavities connected by 10-ring windows and hemicavities at the external surface, showed lower steady-state DEB conversion and EB selectivity. The increase in conversion at short time on stream (TOS) in all catalysts is due to the existence of a well-known induction period for transalkylation of alkyl aromatics in large pore zeolites. During this induction period, higher alkylated aromatics are formed inside the zeolite pore, resulting in a higher proportion of benzene within the products and therefore in a higher selectivity when reaching the steady state.^28^ Notably, the MWW material exhibits a clear decrease in selectivity along TOS because the formation of the bulkier products during the induction period probably blocks the smaller 10-ring channels, leaving only residual activity on the external surface.

After discovering that IWV is an excellent zeolite structure for DEB-Bz transalkylation reaction, we attempted to synthesize the zeolite with a higher Al content to further increase the catalyst activity. However, we were not successful in doing that under our synthesis conditions using DPDMP^+^ as OSDA. Nevertheless, IWV could be synthesized with a higher Al content with an imidazolium-based dicationic OSDA following the procedure described by Davis et al.^35^ In this way, an IWV sample with a Si/Al ratio of 13.4 was obtained, and it was named as IWV-D. For comparison purposes, CBV720 was employed to compare with IWV-D since its Si/Al ratio was similar (Si/Al ∼ 13.4, Table S1). The results in Figure S5 show that, at a WHSV of 10 h^–1^, the catalysts with a higher amount of Al reach higher steady-state DEB conversion, 83% and 74% for IWV-D and CBV720, respectively, and a selectivity toward EB above 95% in both cases. Since the thermodynamic equilibrium DEB conversion of the DEB-Bz transalkylation under these conditions approaches 90%, the samples were further evaluated at a much higher space velocity (WHSV_DEB_ = 40 h^–1^) to obtain lower conversion to better observe catalytic differences. Under these conditions, it is clearly observed that the zeolites with the IWV structure are more active and selective that the corresponding FAU samples with similar Al content and also more active than the corresponding MWW and MOR zeolites (Figure S5).

### 2.3 Kinetic Study

**Figure 2:**
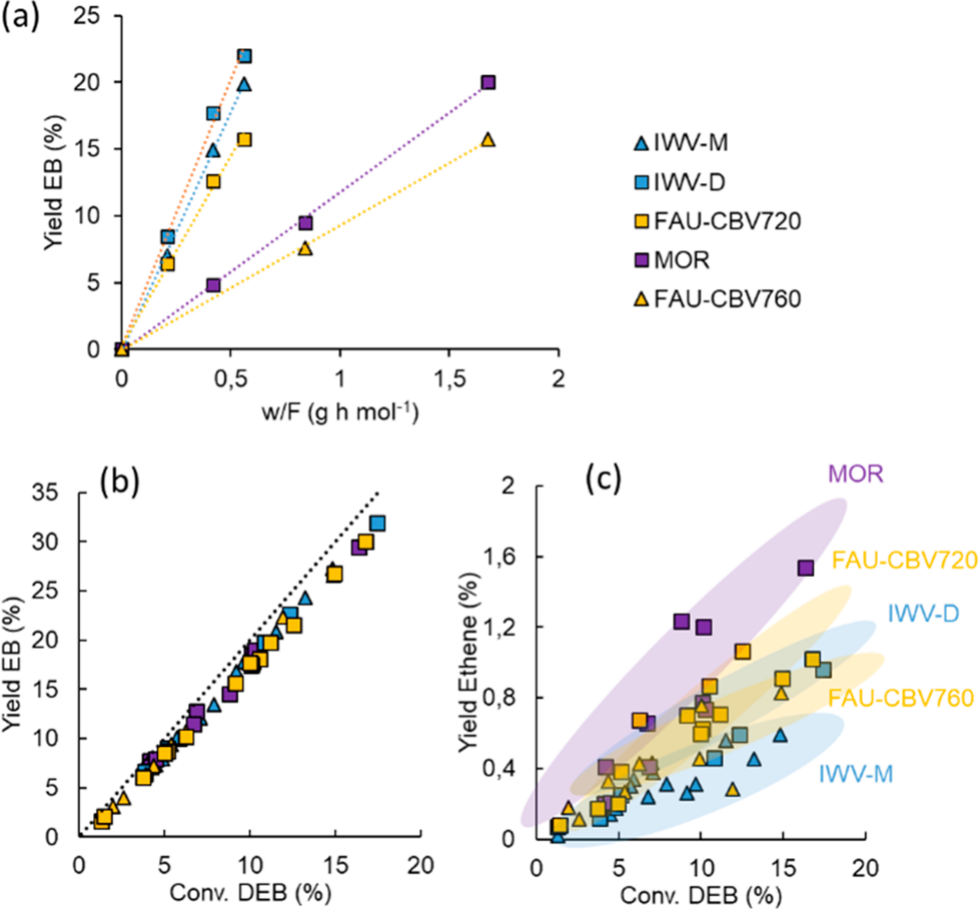
EB yield at different contact times (a) and yield of EB (b) and ethene (c) versus DEB conversion for different zeolites. Reaction conditions: *T* = 200–250 °C, P atmospheric, feeding composition: N_2_:Bz: DEB = 30:5:1 (molar), *w*/*F* = 0.21–3.36 g_cat_ h/mol_DEB_. The error bars in (a) stand for the standard deviation of at least three experiments for each data point. Conversion (a) and EB selectivity (b) with time on stream.

To have kinetic insight into the catalytic performance of the different zeolites, the catalysts were tested in gas-phase reaction conditions (see Experimental Section in the Supporting Information) in order to avoid surface saturation of the zeolite with reactants. Initial reaction rates were obtained from catalytic tests performed at different contact times (*w*/*F*, where *w* is the weight of catalysts and *F* is the feeding flow of DEB). The results plotted in Figure 2 and summarized in Table S2 clearly show again that the highest initial reaction rates are obtained with the catalysts with the IWV structure (0.36 and 0.41 mol_EB_/(g_cat_ h) for IWV-M and IWV-D, respectively), closely followed by the FAU sample with low Si/Al ratio (FAU-CBV720, 0.30 mol_EB_/g_cat_ h). The MOR and FAU-CBV760 samples exhibit similar activities (0.11 and 0.09 mol_EB_/(g_cat_ h), respectively). For the sake of comparison, the initial reaction rate was normalized with respect to the Al content leading to the following order: IWV-M > IWV-D > FAU-CBV720 > FAU-CBV760 > MOR (Table S2), which indicates a strong correlation between the framework structure and the catalytic activity.

Regarding selectivity, the mechanism depicted in Scheme 1 indicates that, besides the desired transalkylation between DEB and Bz yielding two molecules of EB, two other primary processes can take place: DEB dealkylation producing EB and ethene and disproportionation of two DEB molecules forming EB and TEB. The plots of the yield to different reaction products versus DEB conversion in Figure 2 show that the prevailing reaction is the DEB-Bz transalkylation in all zeolites, with the yield of EB approaching the stoichiometric value. Dealkylation to produce ethene increases on the catalysts with higher Al content, and its contribution to DEB conversion is more important in MOR than in other zeolite structures. In all cases, the yield of TEB is always below 1% and reaches a minimum in MOR, probably due to spatial restrictions in the unidimensional channel.^22,36^

### 2.4 How the Scaffold Selects the Reaction Mechanism

**Figure 3:**
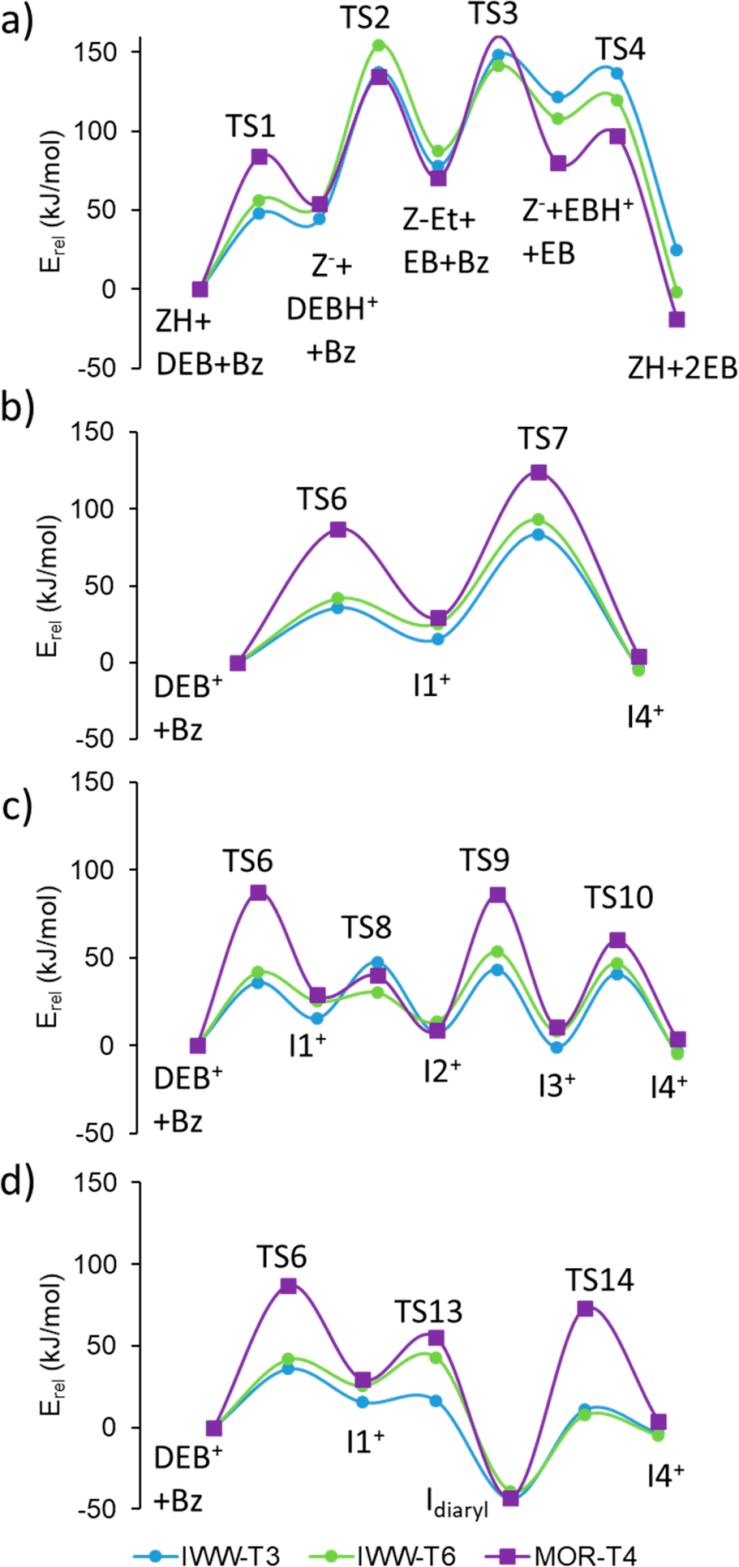
DFT energy profiles for (a) alkyl-transfer pathway; (b) diaryl-mediated pathway, direct H transfer; (c) diaryl-mediated, multiple-step H transfer; and (d) diaryl-mediated, neutral intermediate.

Following our initial hypothesis, the fact that the IWV zeolite gives higher intrinsic activity and selectivity should be related with a better stabilization of the transition states involved in the diaryl-mediated reaction pathway by zeolite confinement. To check this, we performed a detailed theoretical study of the two possible mechanistic routes presented in Scheme 1 on the IWV and MOR zeolite structures by means of periodic DFT calculations. Brønsted acid sites were placed at the two most stable locations for Al in the IWV structure, T3 and T6 (see Table S3), and at the T4 position accessible from the 12-ring channel in MOR. The geometries of all minima and transition states involved in the alkyl-transfer and diaryl-mediated pathways of DEB-Bz transalkylation and DEB dealkylation were fully optimized without restrictions at the three sites considered, IWV-T3, IWV-T6, and MOR-T4, and using the most stable isomer of DEB, para-DEB. The calculated activation and reaction energies for each elementary step are summarized in Supporting Information Table S4, and the corresponding energy profiles are plotted in Figure 3.

**Figure 4:**
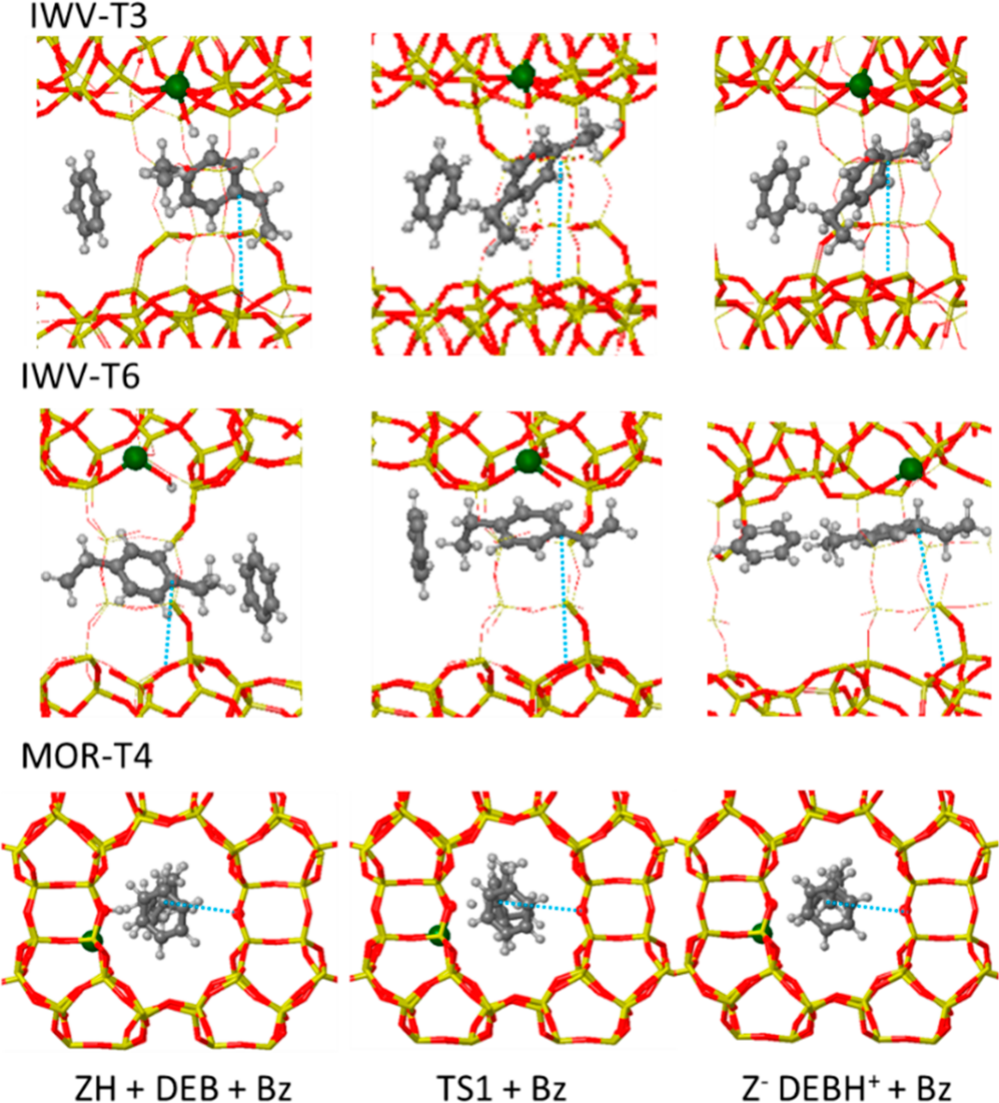
Optimized geometries of reactants, first transition state (TS1), and DEBH^+^ carbonium ion intermediate involved in the alkyl-transfer pathway on IWV-T3, IWV-T6, and MOR-T4 sites. Framework Si and O atoms are depicted as yellow and red sticks. Al, C, and H atoms are depicted as green, gray, and white balls, respectively. Dotted blue lines indicate the C–O_f_ distances given in Table S5.

According to Scheme 1, the first step in the alkyl-transfer pathway is the protonation of DEB through transition state TS1 to form a DEBH^+^ carbonium ion with a pentacoordinated C atom (see optimized geometries in Figures 4, S6, and S7). The calculated activation energies for this step in IWV are not too high (around 50 kJ mol^–1^); however, the process is endothermic, and the DEBH^+^ cations formed are unstable, with the barriers for backward decomposition into DEB reactant being lower than 4 kJ mol^–1^. The alternative ethyl transfer to the zeolite framework through TS2 requires activation energies between 90 and 100 kJ mol^–1^, which render this pathway highly improbable (see Figure 3 and Table S4).

In MOR-T4, the activation barrier for DEB protonation is higher, 84 kJ mol^–1^, but the relative stability against backward decomposition of the DEBH^+^ carbonium ion formed, with a barrier of 30 kJ mol^–1^, allows some competitive dealkylation through TS2. The surface ethoxy group generated in this step might react with benzene to form a new EB molecule with an activation energy of 100 kJ mol^–1^ or might decompose via transition state TS5, yielding ethene with a similar activation barrier of 97 kJ mol^–1^. Indeed, the experimental observation of some ethene when performing the transalkylation reaction with MOR would indicate the possible contribution of the alkyl transfer pathway in this zeolite, as suggested by the DFT calculations.

Further insight into the different behavior of IWV and MOR catalysts is provided by the optimized geometries of the structures involved, as depicted in Figures 4, S6, and S7. The 12-ring channels in the two frameworks have similar dimensions, 6.2 × 6.9 Å in IWV and 6.5 × 7.0 Å in MOR, according to the IZA database. However, larger voids are present at the intersections between the two 12-ring channel systems in IWV. These wider regions are accessible, irrespective of the Al location, and the DEBH^+^ and EBH^+^ cations involved in this pathway come close to the zeolite wall at one side of the channel while getting far from the framework oxygen placed at the other side (Figure 4). Taking the distance between the tertiary carbon atom of the DEB aromatic ring and the closest framework oxygen atom placed at the opposite side of the channel, rC-Of in Figure 4 and Table S5, as an indication of the situation of the cationic intermediates within the channels, we observe systematic larger values in IWV (between 6.5 and 9.1 Å) than in MOR (always below 5.7 Å). These values indicate that, in MOR, the reactant and intermediate species tend to be allocated in the center of the straight 12-ring channel, fully surrounded by framework oxygen atoms at the right distance to maximize the stabilization by confinement. However, the first proton transfer through TS1 requires the approximation of the organic molecule to the Brønsted acid site, causing an additional destabilization, not present in the IWV structure, which explains the higher relative energy of TS1 in MOR (see Figure 3).

**Figure 5:**
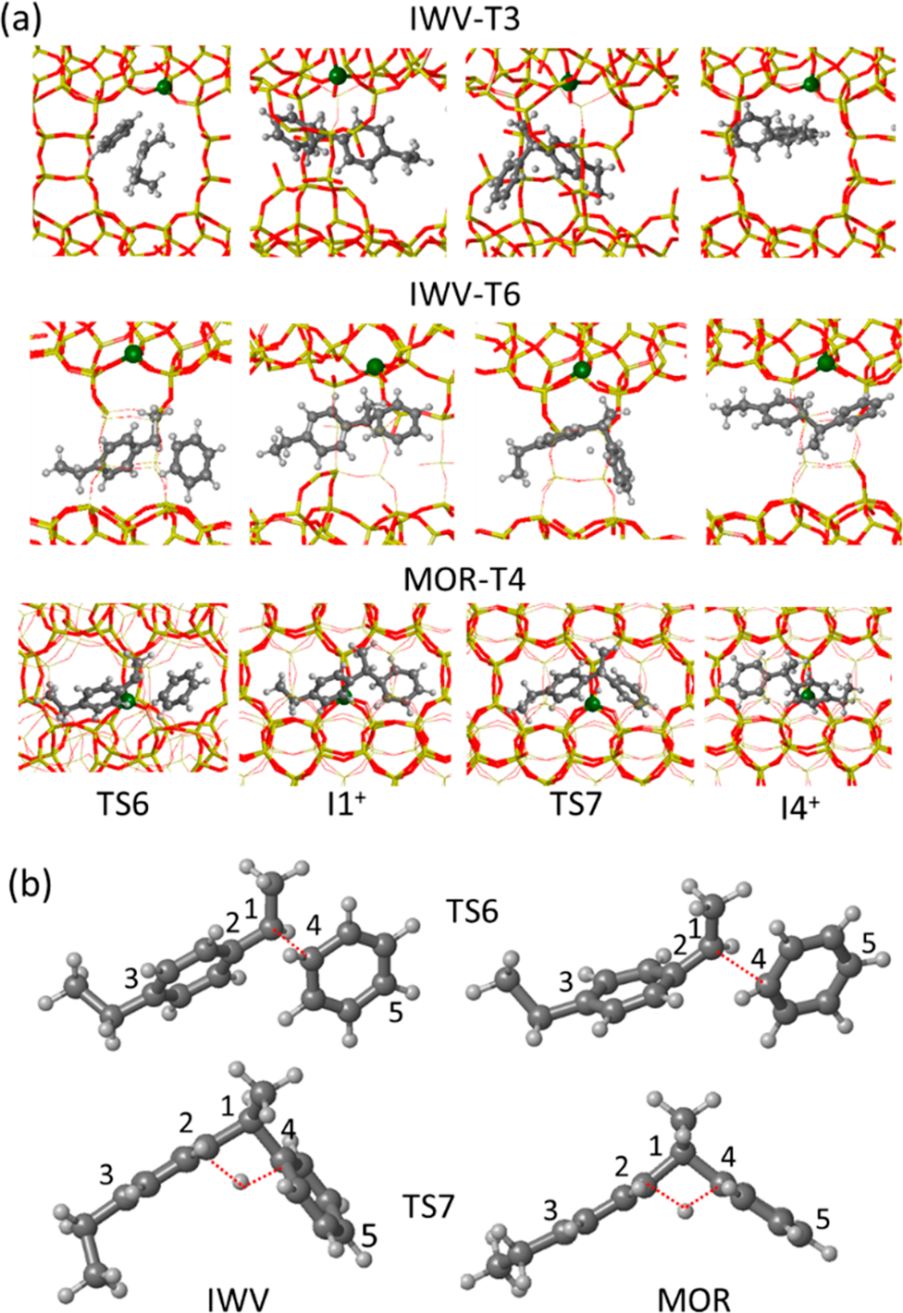
(a) Optimized geometries of the cationic I1^+^ and I4^+^ diaryl intermediates and the transition states for the formation of I1^+^ and for the direct proton transfer between aromatic rings on IWV-T3, IWV-T6, and MOR-T4 sites. Framework Si and O atoms are depicted as yellow and red sticks. Al, C, and H atoms are depicted as green, gray, and white balls, respectively. (b) Enlarged images of TS6 and TS7 in the two zeolite structures with the atom labeling used in Table S6.

The diaryl-mediated pathway starts with the preliminary formation of a carbenium ion with a tricoordinated carbon atom (DEB^+^ in Scheme 1), and this DEB^+^ carbenium ion enters the catalytic cycle by reacting with benzene through transition state TS6 to form the first diaryl cationic intermediate I1^+^. The calculated activation energies for this step are much lower in IWV (∼40 kJ mol^–1^) than in MOR (87 kJ mol^–1^) (see Figure 3 and Table S4), while the stability of the resulting I1^+^ intermediates is relatively similar in all catalyst models. The different activation energies obtained in the two zeolite structures are associated with the steric constraints imposed by the unidimensional channels or MOR on the relative orientation of the two reactant molecules and to the enhanced stabilization by confinement of TS6 and I1^+^ in the IWV channel system. As depicted in Figure 5, the DEB^+^ and Bz reactants in MOR are placed along the same channel, and the optimized distance between the two reacting carbon atoms in the transition state TS6 is long, 2.37 Å. In contrast, in the bidimensional channel system of IWV, the DEB^+^ and Bz reactants approach each other forming an angle of 120°, so that they can come closer, resulting in a shorter optimized C–C distance in TS6, 1.99 and 1.82 Å in IWV-T3 and IWV-T6, respectively (see Table S6). The geometrical constraints in MOR are also reflected in a wider C4–C1–C2 angle (117° in MOR versus 107° in IWV), a distortion of the C1–C2–C_aromatic_ angles from 120° to 112° and 126°, and a deformation of the planar structure of the DEB fragment, with a calculated C3–C2–C1 angle of 168°. Once the C1–C4 bond is formed, the diaryl intermediate I1^+^ adopts a more similar geometry in the three sites considered. The lower endothermicity of the diaryl formation step in the IWV-T3 site, 15 kJ mol^–1^, could be related to a better confinement of I1^+^ within the region of the 12-ring channel where T3 is. Formation of the I1^+^ intermediate in the IWV-T3 site is similarly easy for the less stable *ortho*- and *meta*-isomers of EB, with activation barriers of 47 and 30 kJ/mol, respectively (see Figure S8).

Once the first diaryl intermediate I1^+^ is formed, there are at least three different routes to transfer a proton from one aromatic ring to the other one, generating the I4^+^ intermediate precursor of the ethylbenzenium carbenium ion (EB^+^ in Scheme 1). The simplest process involves a direct proton transfer from C1 to C4 through a four-membered transition state, TS7 (see Figure 5). Again, the activation energy for this step is clearly higher in MOR, 95 kJ mol^–1^, than in IWV, 68 kJ mol^–1^ (see Table S4 and Figure 3). The reason, as before, is the more constrained geometry of the transition state TS7 in the straight channel of MOR, with a more open C5–C1–C3 angle and a longer C5–C3 distance (see Figure 5). Alternatively, the proton attached to C2 in intermediate I1^+^ can migrate to another carbon atom of the same ring with lower activation barriers generating an I2^+^ cationic intermediate (Scheme 1, blue path). From I2^+^, the proton can be transferred to the other aromatic ring through a six-membered ring transition state TS9 with activation barriers of 40 kJ mol^–1^ in IWV and clearly higher, 77 kJ mol^–1^, in MOR (see Table S4 and optimized structures in Figure S9). Other H-shift steps between the two rings and within the same ring have been considered (see Figure S10), all of them with calculated activation energies between 40 and 80 kJ mol^–1^. Finally, a third possibility involves the transfer of the proton to the negatively charged zeolite framework, generating a neutral diaryl molecule (I_diaryl_ in Scheme 1, gray path), which must be reprotonated by the Brønsted acid site. The deprotonation step through TS13 is clearly exothermic and kinetically easy, with activation energies ranging from 1 kJ mol^–1^ in IWV-T3 to 34 kJ mol^–1^ in MOR. The subsequent protonation of the neutral species is similarly endothermic in all cases but involves much lower activation barriers in IWV zeolite, 50–55 kJ mol^–1^, than in MOR, 121 kJ mol^–1^ (Table S4, Figure 3, and optimized structures in Figure S11).

In summary, the DFT study shows that the IWV structure intrinsically favors the diaryl-mediated pathway in the DEB-Bz transalkylation reaction because the topology of the bidimensional channel system enhances the formation of the I1^+^ intermediate. The activation barrier for the global process ranges from 40 to 70 kJ mol^–1^, depending on the route followed for the intramolecular proton transfer, generating the I4^+^ intermediate. In contrast, the alkyl-transfer route competes with the diaryl-mediated pathway in the unidimensional channels of MOR, and the calculated activation barriers are never below 80 kJ mol^–1^, in agreement with the lower activity experimentally determined for MOR.

### 2.5 Stabilization by Confinement and Acid Site Distribution

From the initial reaction rates of EB formation obtained experimentally, activation energies *E*_a_ were calculated by means of the Arrhenius plots, and Gibbs free energies Δ*G*^‡^, enthalpies Δ*H*^‡^, and entropies Δ*S*^‡^ of activation were obtained by means of the Eyring equation (see details in the Experimental Section). The results are plotted in Figure S12 and listed in Table 1. The lowest activation energies are obtained with the two IWV zeolite samples (58 kJ/mol for IWV-M and 56 kJ/mol for IWV-D), followed by the two FAU (65 kJ/mol for FAU-CBV760 and 67 kJ/mol for FAU-CBV720), being the highest value obtained for MOR (74 kJ/mol). The same trend was found for the enthalpies of activation Δ*H*^‡^ (see Table 1), indicating that the energy involved in the rate-determining step of the mechanism is lower in IWV than in FAU and the highest in MOR. On the other hand, the change in entropy is clearly negative in all cases, but the value is significantly smaller in MOR (−135 J/mol K) than in the other structures (from −152 to −160 J/mol K). Taking into account that the entropy loss associated with the formation of diaryl intermediates is much larger than that implicated in any of the elementary steps composing the alkyl-transfer pathway, these data might indicate a larger contribution of the alkyl-transfer route to the conversion of DEB in MOR. The results obtained experimentally are fairly close to the theoretical ones and follow the same order, indicating that the diaryl-mediated pathway is favored in the IWV zeolite due to a larger stabilization by confinement of the transition states and intermediates involved in this route, in agreement with the starting hypothesis.

**Table 1:** Kinetic Parameters Obtained for Different Zeolites

	*E*_a_ (kJ/mol)^a^	Δ*H*^‡⊖^ (kJ/mol)^a^	Δ*S*^‡⊖^ (J/mol K)^b^	Δ*G*^‡⊖^ (kJ/mol)^a^
FAU-CBV720	66.5	58.6	–151.6	97.6
IWV-D	55.9	55.8	–159.8	99.4
IWV-M	58.3	54.2	–158.1	97.4
MOR	74.2	70.1	–135.3	107.0
FAU-CBV760	65.1	61.0	–156.9	103.8

a Error: ±4 kJ/mol.

b Error: ±5 J/mol.

According to the kinetic parameters listed in Table 1, the activation energies obtained for IWV-M and IWV-D catalysts show no significant difference, indicating that in both IWV samples the reaction proceeds through the same mechanism. However, the initial reaction rate of DEB-Bz transalkylation normalized by aluminum amount is clearly higher for IWV-M than for any other catalyst tested, including the isostructural IWV-D (Table S2), and therefore some actions were taken to clarify the origin of this difference.

To diminish the potential influence caused by the different acid amounts, the two IWV samples were poisoned by different amounts of Na^+^ to obtain different amounts of acid sites. Then the samples were tested under the same reaction conditions as before and the initial rates plotted against acid amount (see Figure S13). The slope of these plots should represent the average turnover frequency of the acid sites in each sample. For both samples, the initial rate showed a linear decrease along with the decrease of acid site amount. The fact that the samples with Na^+^ are in the same line with the parent H-form samples indicates that the presence of Na^+^ does not provide spatial hindrance for the reaction. In all cases, IWV-M showed greater slope, indicating that the acid sites in IWV-M are in average more efficient for transalkylation than in IWV-D.

To further unravel if this difference is specific for the transalkylation reaction, the IWV-D and IWV-M samples were tested in the alkylation of Bz with ethene, which also produces EB but has no bulky reaction intermediates that can be specially accommodated by the surrounding confinement. Thus, the reaction rate should be proportionally related to the amount of accessible acid sites. The alkylation of Bz with ethene was conducted under reaction conditions similar to those previously used in the transalkylation reaction, and the contact time was controlled to obtain initial conversions lower than 20% to avoid secondary reactions (see Table S7 and Figure S14). In all cases, the main products obtained are EB and DEB from alkylation of Bz and EB, respectively. Therefore, the reaction rate of alkylation was calculated based on the yield of EB+2DEB. The initial rate of alkylation decreases in the order: FAU-CBV720 > IWV-D > MOR > IWV-M > FAU-CBV760 (see Table S8). As expected, the reaction rates are higher in the samples with more aluminum, and when they are normalized by acid amount, all samples show similar activity. Thus, it is possible to conclude that the alkylation reaction can be an indicator of the activity of all the accessible acid sites and that all the acid sites are equally active in all the samples for the less sterically demanding alkylation of benzene with ethene.

**Figure 6:**
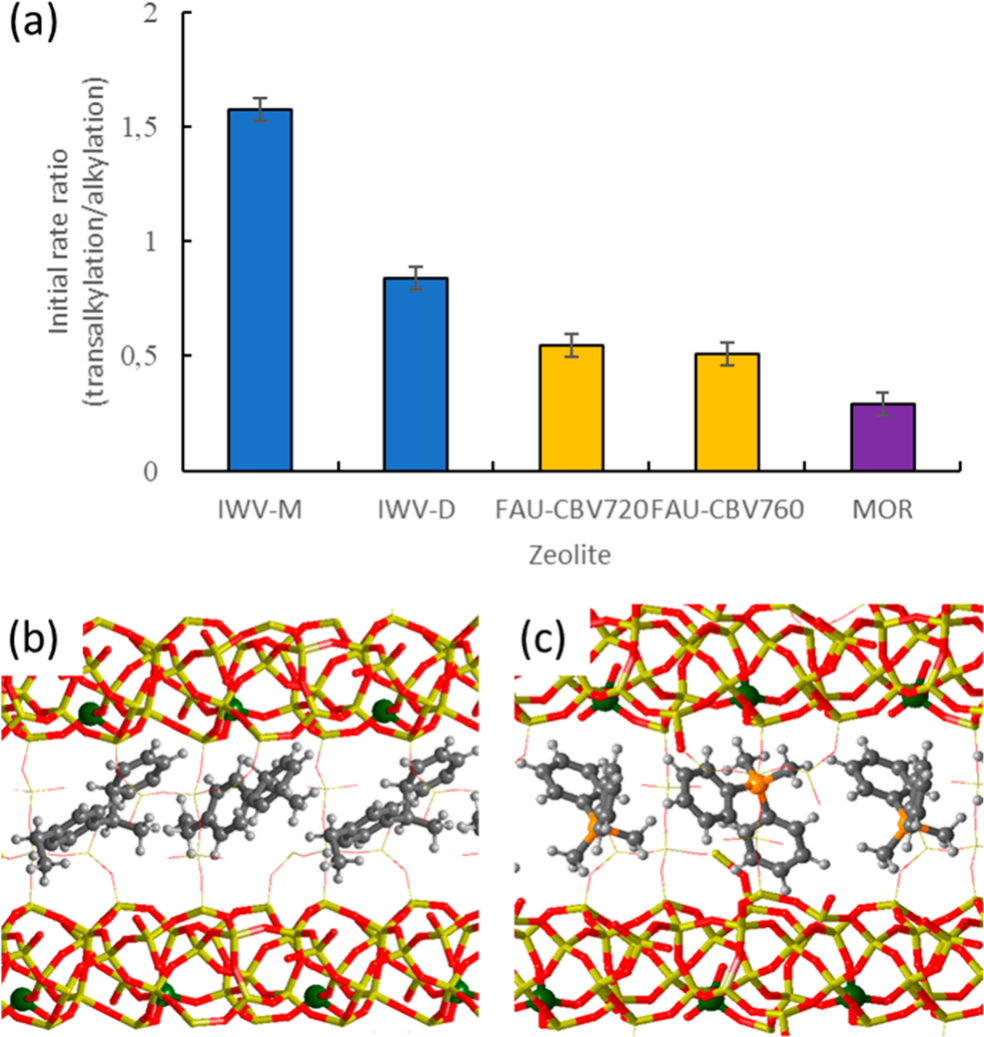
Ratio of transalkylation/alkylation initial rates for different zeolite catalysts (a) and optimized geometries of I1^+^ intermediate (b) and DPDMP^+^ OSDA (c) in the IWV zeolite. Framework Si and O atoms are depicted as yellow and red sticks. Al, P, C, and H atoms are depicted as blue, green, orange, gray, and white balls, respectively. The error bars in (a) stand for the standard deviation derived from at least three experiments from both transalkylation and alkylation reactions.

Then it appears that the relative ratio of the initial reaction rate of DEB-Bz transalkylation and Bz-ethene alkylation should indicate if the distribution of the acid sites is preferentially optimized for the transalkylation. As shown in Figure 6a, this ratio is clearly the highest in the case of IWV-M and decreases in the order: IWV-M > IWV-D > FAU-CBV720 > FAU-CBV760 > MOR. Since the reaction mechanism in IWV-M and IWV-D is not different, and the acid sites are equally active, we hypothesized that the difference between IWV-M and IWV-D in DEB-Bz transalkylation should be caused by the accessibility of the bulky diaryl intermediates to the active sites, following recent work relating enhanced catalytic activity for some reactions with particular arrangements of Al atoms in the CHA framework.^37,38^

To clarify this point, new DFT calculations were performed in which the channels of the IWV structure were completely filled with the I1^+^ diaryl intermediate (see Figure 6b). In this situation, the central C atoms of two diaryl intermediates are separated by 6.9 Å, and the shortest C–C distance between them is 3.3 Å. Interestingly, the same spatial distribution is found for the mimic OSDA used in the synthesis of IWV-M, with the P atoms being separated by 8.9 Å and the shortest C–C distance between two OSDA molecules being 4.1 Å (Figure 6c). In both cases, the maximum number of I1^+^ or OSDA species that can be accommodated in the Si_38_O_76_ triclinic unit cell used in the calculations is two, which results in an optimum Si/Al ratio of 18 for diaryl stabilization. The IWV-D sample with a Si/Al ratio of 13.4 contains almost three Al atoms per unit cell, but only two of them can be active at the same time, which would explain the lower average intrinsic reactivity for the transalkylation reaction. In contrast, the Al distribution generated by the mimic OSDA in the IWV-M sample allows all active sites to efficiently stabilize the maximum number of diaryl intermediates. If this is true, then the average TOF normalized by Al amount in IWV-D should be ∼2/3 of that in IWV-M, in which all acid sites can be active at the same time. Indeed, the ratio of the initial rate of transalkylation normalized by Al amount in the two IWV samples, 326/508, is close to this value.

## Conclusions

We have shown that when two reaction mechanisms can compete in a particular reaction (here illustrated by DEB-Bz transalkylation), and one of the mechanisms is preferred, a catalyst can be designed and synthesized in which, besides the presence of the active sites (protons) and pore dimensions large enough to accommodate the TS, the active site environment is optimized by building a most adequate catalyst scaffold. This more subtle but determinant catalytic functionality is demonstrated here through the synthesis, by an *ab initio* methodology, of a zeolite structure (ITQ-27) that optimizes site location and environment. This results in a clearly better catalyst than the reported ones for an industrially relevant reaction such as DEB-Bz transalkylation. ITQ-27 preferentially drives the reaction through a diaryl-mediated mechanism for which the acid site location and the zeolite scaffold minimize the activation energy by means of weak interactions, much in the direction on how reactions occur with enzymatic catalysts.

## Supporting Information Available

Experimental details on the synthesis of OSDA and zeolites, characterization, catalytic tests, kinetic studies, and DFT calculations. Physico-chemical properties and characterization (XRD, TEM, ^27^Al MAS NMR) of the catalyst samples used. Catalytic and kinetic data obtained with different catalyst samples. DFT-optimized geometries and calculated activation and reaction energies. Coordinates of all structures (PDF)
