# Supplementary material for: Design and Synthesis of the Active Site Environment in Zeolite Catalysts for Selectively Manipulating Mechanistic Pathways
Source: J Am Chem Soc. 2021 Jul 9;143(28):10718–26. doi: 10.1021/jacs.1c04818 (PMC8529870; doi:10.1021/jacs.1c04818)
Supplement: Supplementary file 1 — ja1c04818_si_001.pdf [file ja1c04818_si_001.pdf]

Supporting Information

Design and synthesis of the active site environment in zeolite catalysts for selectively manipulating mechanistic pathways

Chengeng Li,† Pau Ferri,† Cecilia Paris, Manuel Moliner, Mercedes Boronat,* Avelino Corma*

Instituto de Tecnología Química, Universitat Politècnica de València - Consejo Superior de Investigaciones Científicas, Avenida de los Naranjos s/n, 46022 Valencia, Spain

*Corresponding author: E-mail: acorma@itq.upv.es, boronat@itq.upv.es

† These authors have contributed equally

S1.- Methods

S1.1.- Synthesis of the Organic Structure Directing Agents (OSDAs)

S.1.1.1: Synthesis of diphenyldimethylphosphonium ($DPDMP^+$)

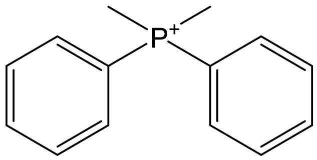

10 g of diphenylphosphine (Sigma-Aldrich) was dissolved in 50 ml of isopropanol. Then, 9.6 g K_2CO_3 was added to the above solution. At this point, 28 g of methyl iodide was added dropwise under stirring, and the resultant mixture was maintained under stirring at room temperature for 72 h. The solvent was separated by rotary evaporator under vacuum. The solid product was then washed with chloroform.

In order to prepare hydroxide form of the same template, 38.0 mmol of the iodide form of template was dissolved in 80 ml of water. Then, 76 g of anion-exchange resin (Amberlite IRN-78) was added to the solution and kept under stir for 24 hours. Finally, the solution was collected by filtration and the obtained hydroxide form of template has an exchange efficiency of at least 95%.

S.1.1.2: Synthesis of 3,3'-(butane-1,4-diyl)bis(1,2,4,5-tetramethyl-1*H*-imidazol-3-ium) (BTMI)

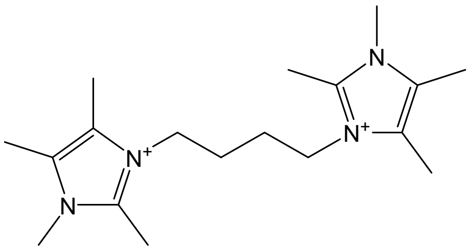

18.3 g tetramethylimidazole (Sigma-Aldrich) was mixed with 10.6 g 1,4-dibromobutane (Sigma-Aldrich) and 50 ml of dry methanol. Then the mixture was heated under reflux for 72 hours. When the reaction has finished, the mixture was cooled down and the solvent was separated by rotary

evaporator under vacuum. The solid product was then redispersed in a few drops of methanol and re-crystallized with ethylacetate. Then the solid was collected by filtration and dried at room temperature under vacuum. The collected product is bromide form of template and has a solid yield of at least 50%.

In order to prepare hydroxide form of the same template, 25.1 mmol of the bromide form of template was dissolved in water. Then 51 g of anion-exchange resin (Amberlite IRN-78) was added to the solution and kept under stir for 24 hours. Finally, the solution was collected by filtration and the obtained hydroxide form of template has an exchange efficiency of at least 95%.

S1.2.- Synthesis of zeolites

S1.2.1.- Synthesis of IWV zeolite using $DPDMP^+$ as OSDA (IWV-M)

30.01 g of a hydroxide solution of $DPDMP^+$ (8.12%wt in water) was mixed with 4.37 g of tetraethylorthosilicate (TEOS, Sigma-Aldrich, 98%wt) and 0.12 g of aluminum isopropoxide (IPA, Sigma-Aldrich, 98%wt). The mixture was stirred until the ethanol and isopropanol formed upon hydrolysis of TEOS and IPA were evaporated by heating at 50°C. The weight of synthesis gel was carefully weighed and the amount of water is controlled to $H_2O:SiO_2$ = 3.4 to facilitate further adding of HF. Finally, 0.44 g of HF solution (SigmaAldrich, 48%wt in water) was added and stirred manually with Teflon spatula, resulting in a thick gel. The final gel composition was SiO2 / 0.028 Al / 0.5 DPDMP / 0.5 HF / 4 H_2O. This gel was transferred to a Teflon-lined stainless steel autoclave and heated at 150°C in a rotary oven. The solid was recovered by filtration, extensively washed with water, and dried at 100°C overnight. The material was calcined at 580°C for 6 hours to remove the organic content located within the crystalline material. ICP analysis of the calcined IWV zeolite reveals a Si/Al molar ratio of 23.8.

Notice: when HF is added into the mixture, the exposure of the synthesis gel should be minimized in order to avoid loss of HF and SiF_4 as well as inhalation of hazardous HF vapor.

S1.2.2.- Synthesis of IWV zeolite using BTMI as OSDA (IWV-D)

1g of 1 mol/L sodium hydroxide was mixed with 1.77 g deionized water and 3.47 g solution containing 29.2% hydroxide form of BTMI. Then 0.9 g of faujasite zeolite (CBV760) was added together with 0.1 g calcined ITQ-27 zeolite seeds into the solution. The final gel composition was SiO_2 / 0.038 Al / 0.21 BTMI / 0.07 NaOH / 19 H_2O. The mixture was then transferred into a Teflon-lined autoclave and heated at 175℃ under rotation for 7 days. The product was collected by filtration and washed with abundant water and the solid product was dried in air overnight at 60°C. The resultant solid presents the characteristic PXRD pattern of the IWV zeolite.

In order to eliminate organic template trapped inside the framework, the zeolite was calcined in air at 550℃ for 6 hours. After cooled to room temperature, the solid was mixed with 1mol/L ammonium nitrate solution at a liquid/solid mass ratio of 50 without heating for 16 hours. Finally the solid was separated by filtration and dried in air at 60℃ and then calcined again in air at 500℃ for 3 hours. ICP analysis of the calcined IWV zeolite reveals a Si/Al molar ratio of 13.4.

S1.2.3.- Synthesis of MWW zeolite

0.94 g of sodium aluminate ($NaAlO_2$; 58.9% Al_2O_3, 38.7% Na_2O, 2.4% H_2O, Carlo Erba) was added to 86 g of deionized water followed by adding of 5 g of a 20%wt aqueous solution of sodium hydroxide. The mixture was kept stirred until achieving a transparent solution. Then 19.83 g of hexamethyleneimine (HMI, Sigma-Aldrich) was added to the solution and kept under stirring for 15 minutes for homogenization. Finally 15 g of fumed silica was added to the mixture forming a gel. The final gel composition was SiO_2 / 0.043 Al / 0.8 HMI / 0.02 NaOH / 20 H_2O. After mechanically stirred for 30 minutes, the gel was transferred to a Teflon-lined autoclave and heated

under rotation at 150℃ for 7 days. The solid product was then separated by filtration, washed with abundant water and then dried in air at 60℃. The resultant solid presents the characteristic PXRD pattern of the MWW zeolite.

In order to prepare the proton form of zeolite for catalysis, the zeolite was first calcined in air in a muffle under 550℃ for 6 hours. After cooled to room temperature, the solid was mixed with 1mol/l ammonium nitrate solution at a liquid/solid mass ratio of 50 without heating for 16 hours. Finally, the solid was separated by filtration and dried in air at 60℃ and then calcined again in air at 500℃ for 3 hours. ICP analysis of the calcined MWW zeolite reveals a Si/Al molar ratio of 20.4.

S1.2.4.- Preparation of MOR zeolite

13.4 g tetraethylammonium bromide (TEABr, Sigma-Aldrich), 8.76 g sodium aluminate (Carlo Erba) and 10.2 g NaOH were dissolved in 344.3 g deionized water. To this solution, 84.8 g fumed silica was added and kept under mechanic stirring till a viscos white gel is obtained. Then this gel is transferred to a Teflon-lined autoclave and heated under rotation at 150°C for 7 days. The solid product was then separated by filtration, washed with abundant water and then dried in air at 60°C. In order to prepare the proton form of zeolite for catalysis, the zeolite was first calcined in air in a muffle under 550 °C for 6 hours. After cooled to room temperature, the solid was mixed with 1 mol/L ammonium nitrate solution at a liquid/solid mass ratio of 50 without heating for 16 hours. Finally, the solid was separated by filtration and dried in air at 60°C and, then, calcined again in air at 500°C for 3 hours. The obtained material is named here as MOR

S1.3.- Characterization techniques

Powder X-ray diffraction measurements were performed with a multisample Philips X'Pert diffractometer equipped with a graphite monochromator, operating at 40 kV and 35 mA, using Cu Kα radiation (λ = 0.1542 nm).

The chemical analyses were carried out in a Varian 715-ES ICP-Optical Emission spectrometer. The samples were dissolved in HNO3/HCl/HF aqueous solution before measurement. The organic content of the as-synthesis materials was measured by elemental analysis performed with a SCHN FISONS elemental analyzer.

Textural properties, including BET surface area, micropore volume and external surface area of the samples, were measured by N_2 adsorption/desorption in a Micromeritics ASAP2000.

The morphological feature and particle sizes were determined by field emission scanning electron microscope (FESEM, JEOL JSM-6300).

MAS NMR spectra were recorded in a Bruker AVANCE III HD 400 WB. 27Al MAS NMR and ^{27}Al MAS NMR spectra were recorded in a Bruker 3.2mm probe at spinning rate of 20 kHz. ^{27}Al MAS NMR spectra were recorded with $\pi/12$ pulse length of 0.5 μs with a 1s repetition time. ^{27}Al MAS NMR spectra were acquired with selective zero quanto z-filter pulse sequence. ^{27}Al chemical shift was referred to $Al^{3+}(H_2O)_6$.

The acidity of the zeolites was determined by infrared spectroscopy combined with adsorption–desorption of pyridine at different temperatures. Infrared spectra were measured with a Nicolet 710 FT-IR spectrometer. Pyridine adsorption–desorption experiments were carried out on self-supported wafers (10 mg/cm^2) of original samples previously activated at 400 ℃ and 10^{-2} Pa for 2 h. After wafer activation, the base spectrum was recorded and pyridine vapor (6.5×10^2 Pa) was admitted into the vacuum IR cell and adsorbed onto the zeolite. Desorption of pyridine was performed under vacuum over 1h periods of heating at 350 ℃, followed by IR measurement at room temperature. All the spectra were scaled according to the sample weight. The numbers of Brønsted and Lewis acid sites were determined from the intensities of the bands at ca. 1545 and 1450 cm^{-1}, respectively.

S1.4.- DEB-Bz transalkylation catalytic tests

Prior to catalytic tests, all zeolites were pelletized, crushed and sieved to a particle size of between 0.2 to 0.4 mm. Alternatively, pellets with particle sizes of between 0.1 to 0.2 mm and 0.4 to 0.8 mm were also prepared to investigate to intraparticle diffusion. In all cases, particle sizes of 0.1 to 0.2 mm and 0.2 to 0.4 mm gave same catalytic results while 0.4 to 0.8 mm gave slightly lower activity, indicating that intraparticle diffusion starts to appear when pellet size approximates to 0.8 mm. Then, the pellet size of between 0.2 to 0.4 mm is selected for all catalytic tests.

S1.4.1.- Gas phase transalkylation

The gas phase transalkylation of diethylbenzene with benzene was carried out in a fixed bed reactor equipped with a bypass line in parallel with reactor. The feedstock is a liquid mixture of benzene and diethylbenzene (Bz:DEB weight ratio 3:1) containing n-octane as internal standard. required amount of pelletized catalyst was properly mixed with silicon carbide to reach a total volume of 12.6 ml. The catalyst was activated in dry N_2 flow at 540°C for 3 hours and then the temperature of reactor was cooled down to the reaction temperature. The abovementioned liquid mixture was fed into the bypass together with the desired amount of N_2 as carrier gas. The composition of the feeding gas has a molar ratio of N_2 : DEB : Bz = 30 : 1: 5 and was monitored on-line with a Varian-3800 gas chromatograph equipped with a 30 m 5% phenyl / 95% dimethyl polysiloxane capillary column connected to a flame ionization detector. At this stage the sample from outlet of the reactor was analyzed every 7 minutes. When the outlet composition according to GC is stable, the feeding was switched to the reactor line and the outlet of the reactor was analyzed by the same gas chromatograph every 9 minutes. The initial activity was measured by precisely sampling at Time On Stream = 0s.

S1.4.2.-Gas phase alkylation

The gas phase alkylation of benzene with ethene was carried out in a fixed bed reactor equipped with a bypass line in parallel with reactor. The feedstock is composed of two parts. One is the liquid mixture of benzene and n-octane, in where n-octane is the internal standard. The other is gas phase ethene. The two lines are fed with N_2 as carrier gas and mixed in a preheater loaded with silicon carbide at 150 °C with a composition of N_2 : Bz : ethene = 60 : 5 : 0.4 (molar ratio). Required amount of pelletized catalyst was properly mixed with silicon carbide to reach a total volume of 12.6 ml. The catalyst was activated in dry N2 flow at 540°C for 3 hours and then the temperature of reactor was cooled down to the reaction temperature. The abovementioned mixture was fed into the bypass, and the composition of the feeding gas was monitored on-line with a Varian-3800 gas chromatograph equipped with a 30 m 5% phenyl / 95% dimethyl polysiloxane capillary column connected to a flame ionization detector. At this stage the sample from outlet of the reactor was analyzed every 7 minutes. When the outlet composition according to GC is stable, the feeding was switched to the reactor line and the outlet of the reactor was analyzed by the same gas chromatograph every 9 minutes. The initial activity was measured by precisely sampling at Time On Stream = 0s.

S1.4.3.- Liquid phase transalkylation

The liquid transalkylation of diethylbenzene with benzene was carried out in a fixed bed reactor. The feedstock is a liquid mixture of benzene and diethylbenzene (Bz:DEB weight ratio 3:1) containing n-octane as internal standard. Required amount of pelletized catalyst was properly mixed with silicon carbide to reach a total volume of 12.6 ml. The catalyst was activated in dry N_2 flow at 540°C for 3 hours and then the temperature of reactor was cooled down to 150°C. The abovementioned liquid mixture was then fed into the reactor in liquid phase and the pressure was

controlled at 3.5 MPa by a back-pressure regulator. The product was analyzed on-line on a Varian-450 gas chromatograph equipped with a 30 m 5% phenyl / 95% dimethyl polysiloxane capillary column connected to a flame ionization detector. At this stage the sample from outlet of the reactor was analyzed every 7 minutes. When the outlet composition according to GC is stable, the temperature of the reactor is increased at a rate of 30°C /min to the target reaction temperature and maintain throughout the reaction.

S1.4.4.-Absence of diffusion limited process

Prior to catalytic test, the absence of diffusion limitation has been checked by testing the reaction under abovementioned procedure changing feeding flow. In gas phase reaction, the N_2 flow was adjusted within the range of 25 ml/min to 1000 ml/min while maintaining the partial pressure of the aromatics. When the flow was above 125 ml/min, the reactions gave identical catalytic results. Then all the gas phase reactions were conducted with a minimum N_2 flow of 250 ml/min to avoid diffusion limitation.

In liquid phase reaction, the feeding flow of the liquid mixture were tested at 9.9 g/h, 19.8 g/h, 33.1 g/h and 66.1 g/h while maintaining the same contact time. The latter three experiments gave identical DEB conversion and product distribution while the flow 9.9 g/h gave DEB conversion lower than the other, indicating a diffusion limitation under this flow. Thus, the reaction is always performed with the liquid mixture feeding flow in the range 19.8 – 66.1 g/h.

S1.4.5.- Kinetic study

Transalkylation reaction between Bz and DEB is a bimolecular reaction. Since benzene is fed in excessive amount than stoichiometric ratio, the concentration of Bz could be considered constant and the reaction as first order reaction. Then the first order rate law equation was employed to obtain the rate constant:

$$k = \frac{\ln(1 - Conv.DEB)}{w/F} = \frac{\ln(1 - \frac{1}{2} \cdot Yield.EB.)}{w/F}$$

Where k is the rate constant, and w/F is the contact time.

Activation energy (Ea) was calculated using the Arrhenius equation, by plotting *lnk* against 1/T:

$$lnk = \frac{-E_a}{\mathrm{R}} \cdot \frac{1}{T} + lnA$$

where k is the reaction rate constant, T is the reaction temperature and A is the pre-exponential factor.

Enthalpy of activation($\Delta H^{\ddagger}$) and entropy of activation ($\Delta S^{\ddagger}$) were obtained by plotting ln(k/T) against 1/T and then calculating with Eyring equation.

$$ln\frac{k}{T} = \frac{-\Delta \mathrm{H}^{\ddagger}}{R} \cdot \frac{1}{T} + ln\frac{\kappa k_B}{h} + \frac{\Delta S^{\ddagger}}{R}$$

where k is the reaction rate constant, T is the reaction temperature, κ is the transmission coefficient and k_B is the Boltzmann's constant.

Gibbs free energy of activation ($\Delta G^{\ddagger}$) was calculated from the above obtained enthalpy and entropy of activation using the equation:

$$\Delta \mathrm{G}^{\ddagger} = \Delta \mathrm{H}^{\ddagger} - \mathrm{T}\Delta \mathrm{S}^{\ddagger}$$

S1.5.- DFT calculations

All calculations are based on periodic density functional theory (DFT) and were performed using the Perdew−Burke−Ernzerhof (PBE) exchange-correlation functional within the generalized gradient approach (GGA),[1,2], as implemented in the Vienna Abinitio Simulation Package (VASP) code.[3] The valence density was expanded in a plane wave basis set with a kinetic energy cutoff of 600 eV, and the effect of the core electrons in the valence density was taken into account by means of the projected augmented wave (PAW) formalism.[4] Integration in the reciprocal space was

carried out at the Γ k-point of the Brillouin zone. Dispersion corrections to the energies were evaluated using the D3 Grimme's method.[5,6]

Mordenite crystallizes in an orthorhombic Cmcm space group with lattice parameters a = 18.094, b = 20.516, and c = 7.524 Å, α = β = γ = 90.0º, and has 144 atoms in the conventional unit cell.[7] To model the MOR structure we used a larger 1 × 1 × 2 supercell containing 288 atoms (96 T and 192 O), with lattice parameters a = 18.184 Å, b = 20.069 Å, c= 14.844 Å, α = 89.991, β = 90.001 and γ = 90.059 obtained after full relaxation of the experimental lattice parameters. ITQ-27 zeolite crystallizes in a orthorhombic Fmmm space group with lattice parameters a = 27.826 Å, b = 26.081 Å, and c = 13.944 Å, and has 456 atoms in the conventional unit cell.[7] In the simulations of the IWV structure we used the primitive unit cell, with triclinic symmetry and lattice parameters a = 14.544 Å, b = 15.784 Å, c= 19.055 Å, α = 48.222, β = 53.643 and γ = 77.742 obtained with the same procedure. This primitive unit cell contains 114 atoms (38 Si and 76 O). Aluminum positions for each zeolite were chosen by intrinsic stability criteria placing one unique Al per unit cell, IWV Si/Al = 37, MOR Si/Al = 95.

Electronic energies were converged to 10^{-6} eV and geometries were optimized until forces on atoms were <0.01 eV/A. Transition states were obtained using the DIMER and NEB algorithms.[8-11] During geometry optimizations, the positions of all atoms in the system were allowed to relax without restrictions.

References

1. Perdew, J. P., Burke, K. & Ernzerhof, M. Generalized gradient approximation made simple. *Phys. Rev. Lett.* **77**, 3865–3868 (1996).

2. Perdew, J. P., Burke, K. & Ernzerhof, M. Generalized Gradient Approximation Made Simple (vol 77, pg 3865, 1996). *Phys. Rev. Lett.* **78**, 1396–1396 (1997).

3. Kresse, G., Furthmüller, J. Efficient iterative Schemes for ab initio total-energy calculations using a plane-wave basis set. *Phys. Rev. B* **54**, 11169-11186 (1996).

4. Blöchl, P. E. Projector augmented-wave method. *Phys. Rev. B* **50**, 17953–17979 (1994).

5. Grimme, S. Accurate description of van der Waals complexes by density functional theory including empirical corrections. *J. Comput. Chem.* **25** 1463–1473 (2004).

6. Grimme, S. Semiempirical GGA-Type Density Functional Constructed with a Long-Range Dispersion Correction. *J. Comput. Chem.* **27**, 1787-1799 (2006).

7. http://www.iza-structure.org/databases

8. Henkelman, G. & Jónsson, H. A dimer method for finding saddle points on high dimensional potential surfaces using only first derivatives. *J. Chem. Phys.* **111**, 7010–7022 (1999).

9. Heyden, A., Bell, A. T. & Keil, F. J. Efficient methods for finding transition states in chemical reactions: Comparison of improved dimer method and partitioned rational function optimization method. *J. Chem. Phys.* **123**, 224101-224114(2005).

10. Henkelman, G., Uberuaga, B. P. & Jónsson, H. Climbing image nudged elastic band method for finding saddle points and minimum energy paths. *J. Chem. Phys.* **113**, 9901–9904 (2000).

11. Sheppard, D., Xiao, P., Chemelewski, W., Johnson, D. D. & Henkelman, G. A generalized solid-state nudged elastic band method. *J. Chem. Phys.* **136**, 074103-074110 (2012).

S2.- Supplementary Figures

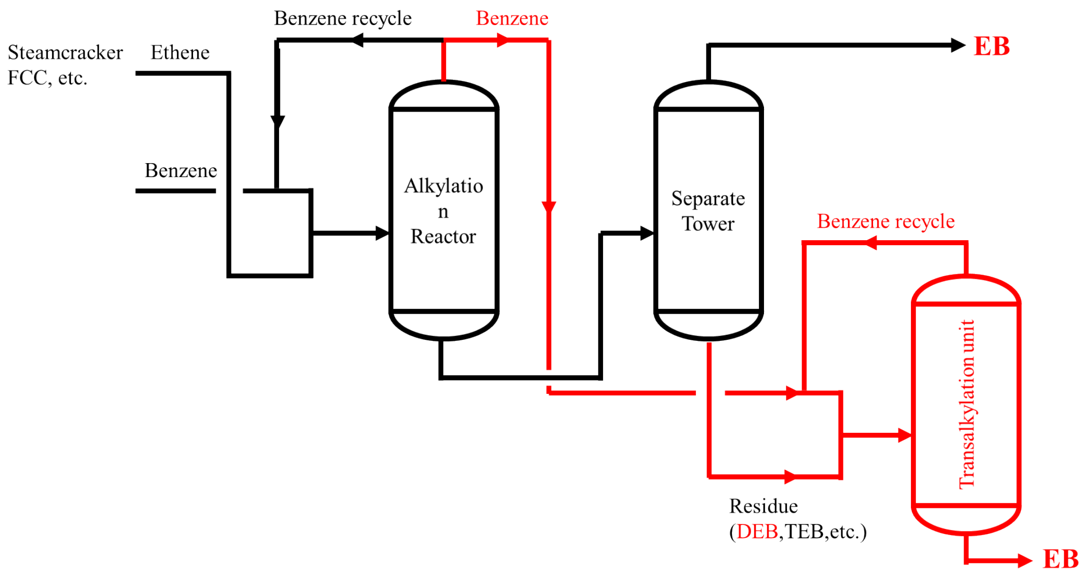

Figure S1. A brief scheme of ethylbenzene production industry, the processes in red represent transalkylation between benzene and diethylbenzene.

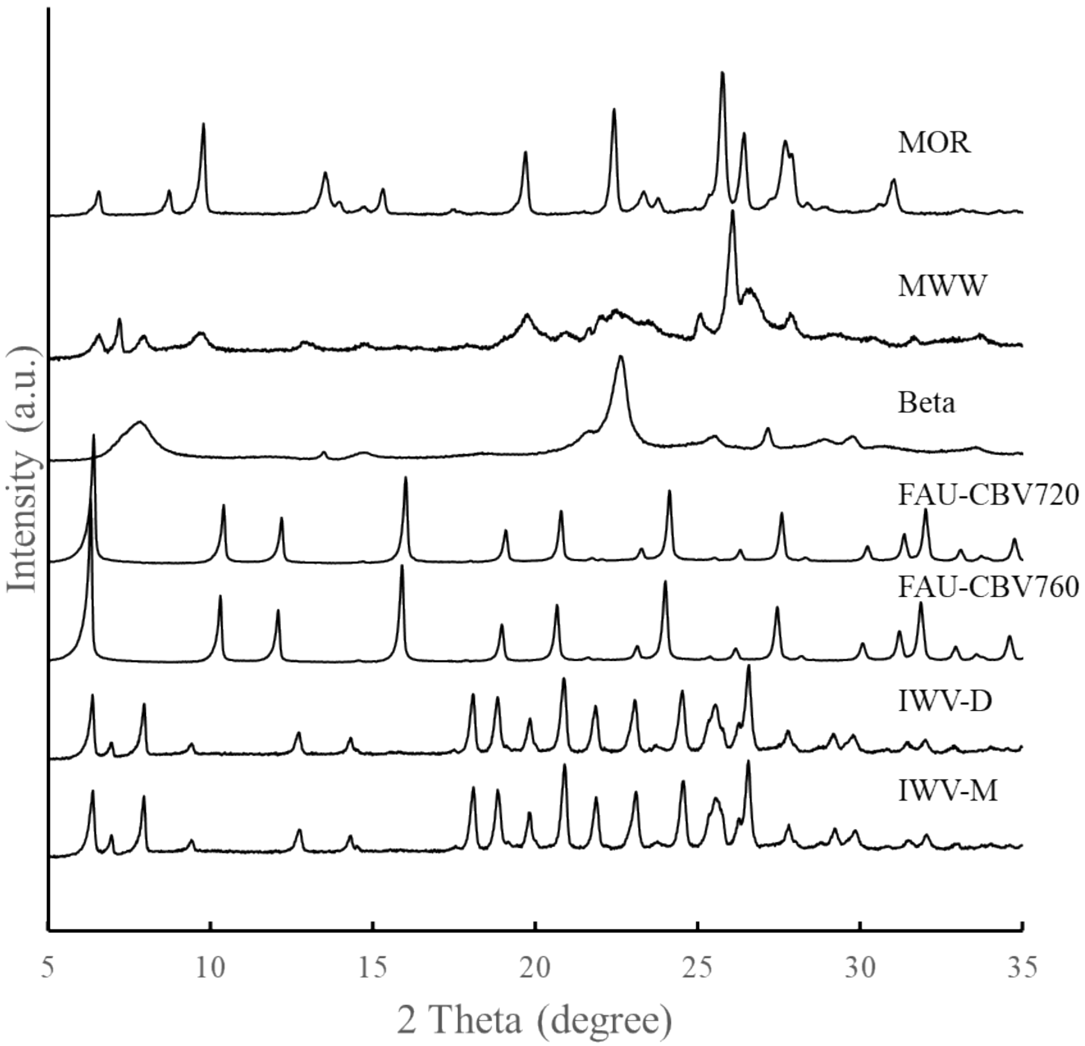

Figure S2. PXRD patterns of different zeolite catalysts involved in this study.

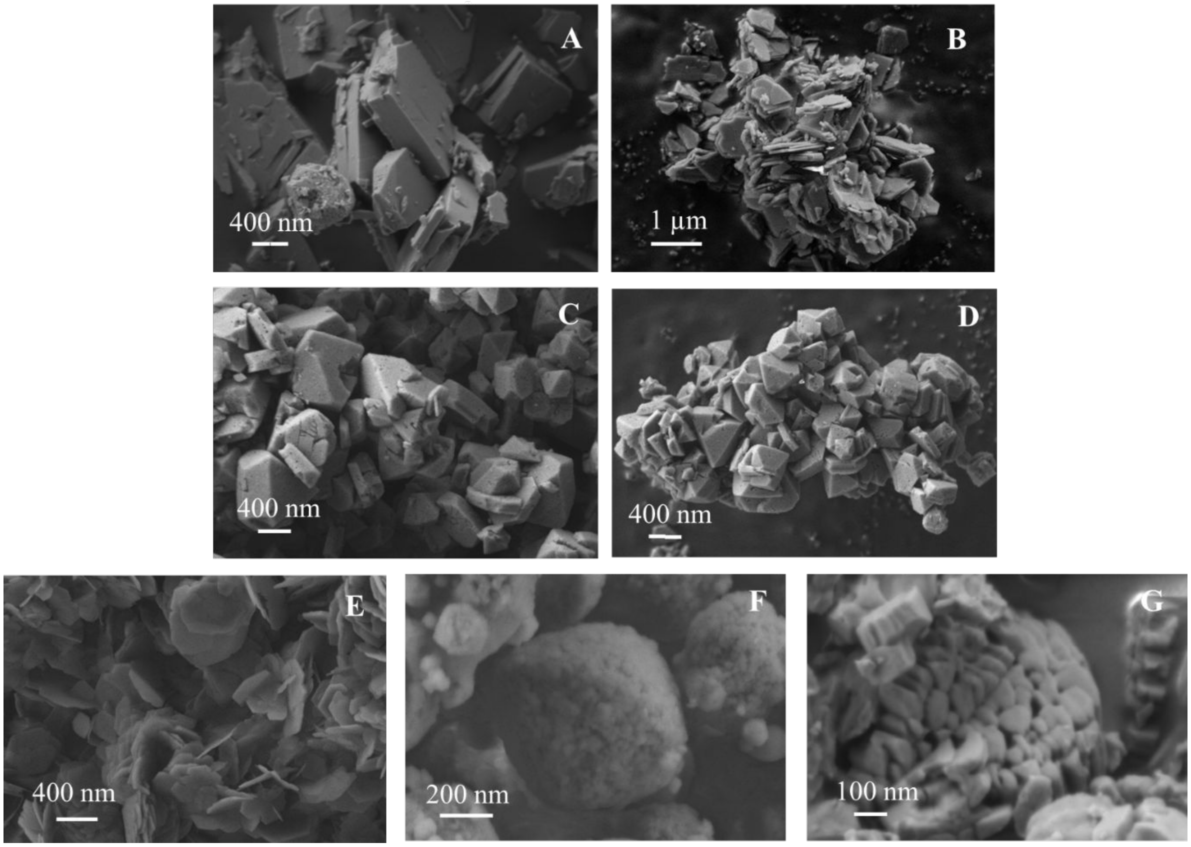

Figure S3. FESEM images of (A) IWV-M, (B) IWV-D, (C) FAU-CBV760, (D) FAU-CBV720, (E) MWW, (F) Beta and (G) MOR.

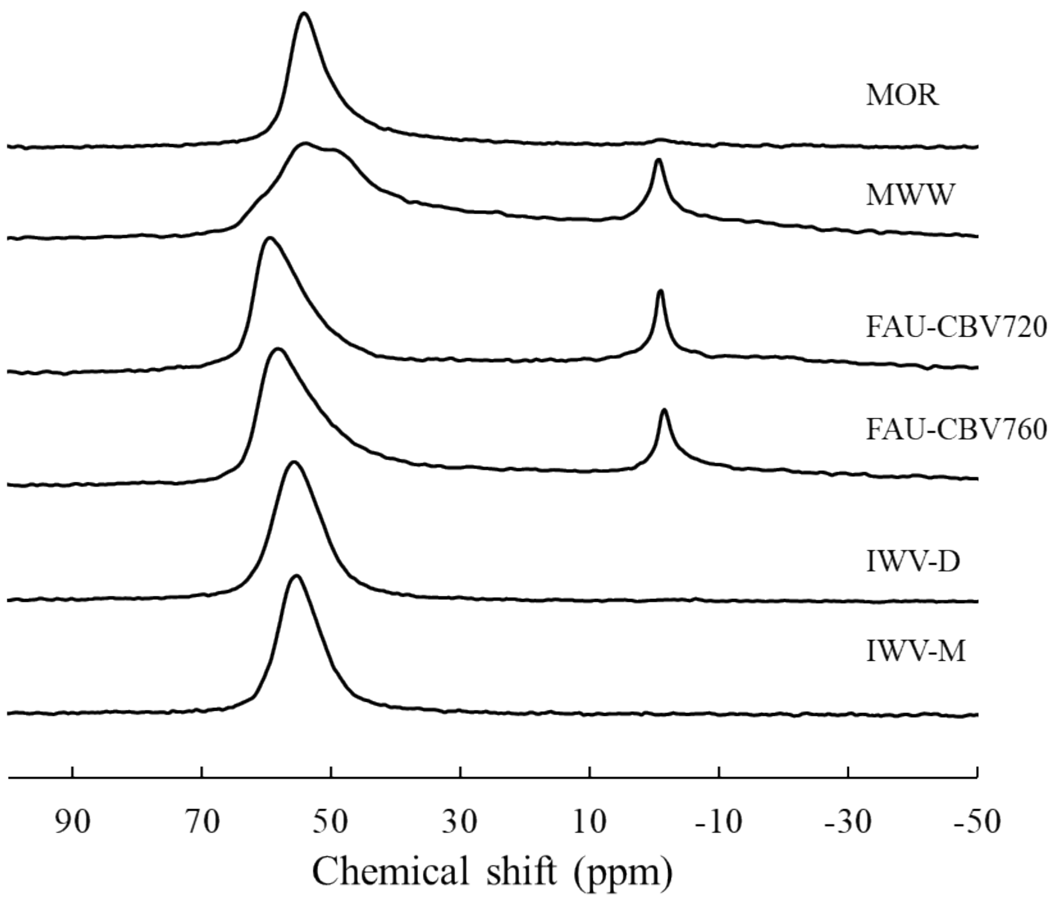

Figure S4. Solid ^{27}Al MAS NMR spectra of the calcined zeolite samples involved in this study

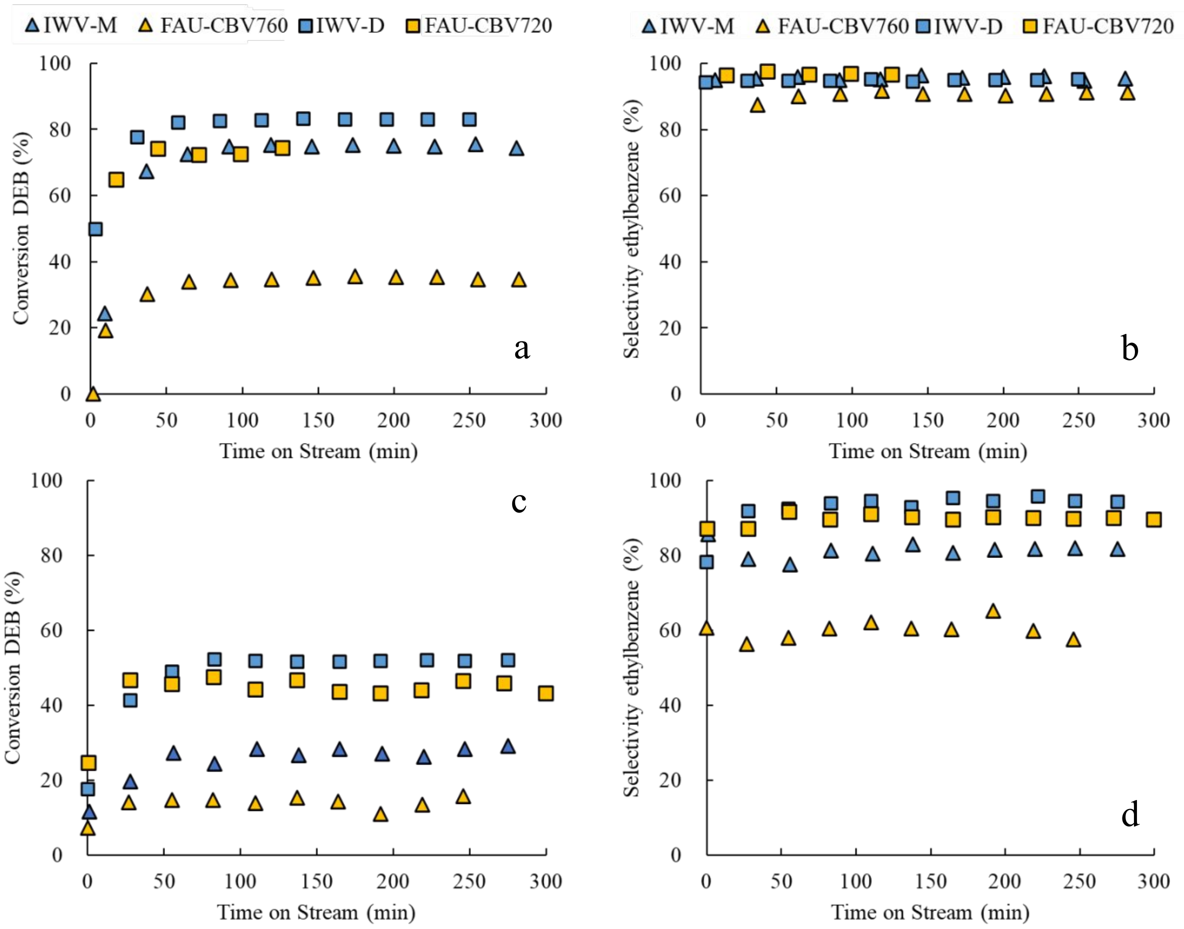

Figure S5. DEB conversion (a,c) and EB selectivity (b,d) with Time On Stream (TOS) for the transalkylation of DEB over zeolite catalysts with IWV and FAU framework topology and different Si/Al ratio. Reaction conditions: (a,b) T = 250°C; P = 3.5MPa; Bz/DEB = 3:1 (wt); $WHSV_{DEB}$ = 10h^{-1}. (c,d) T = 250°C; P = 3.5MPa; Bz/DEB = 3:1 (wt); $WHSV_{DEB}$ = 40h^{-1}.

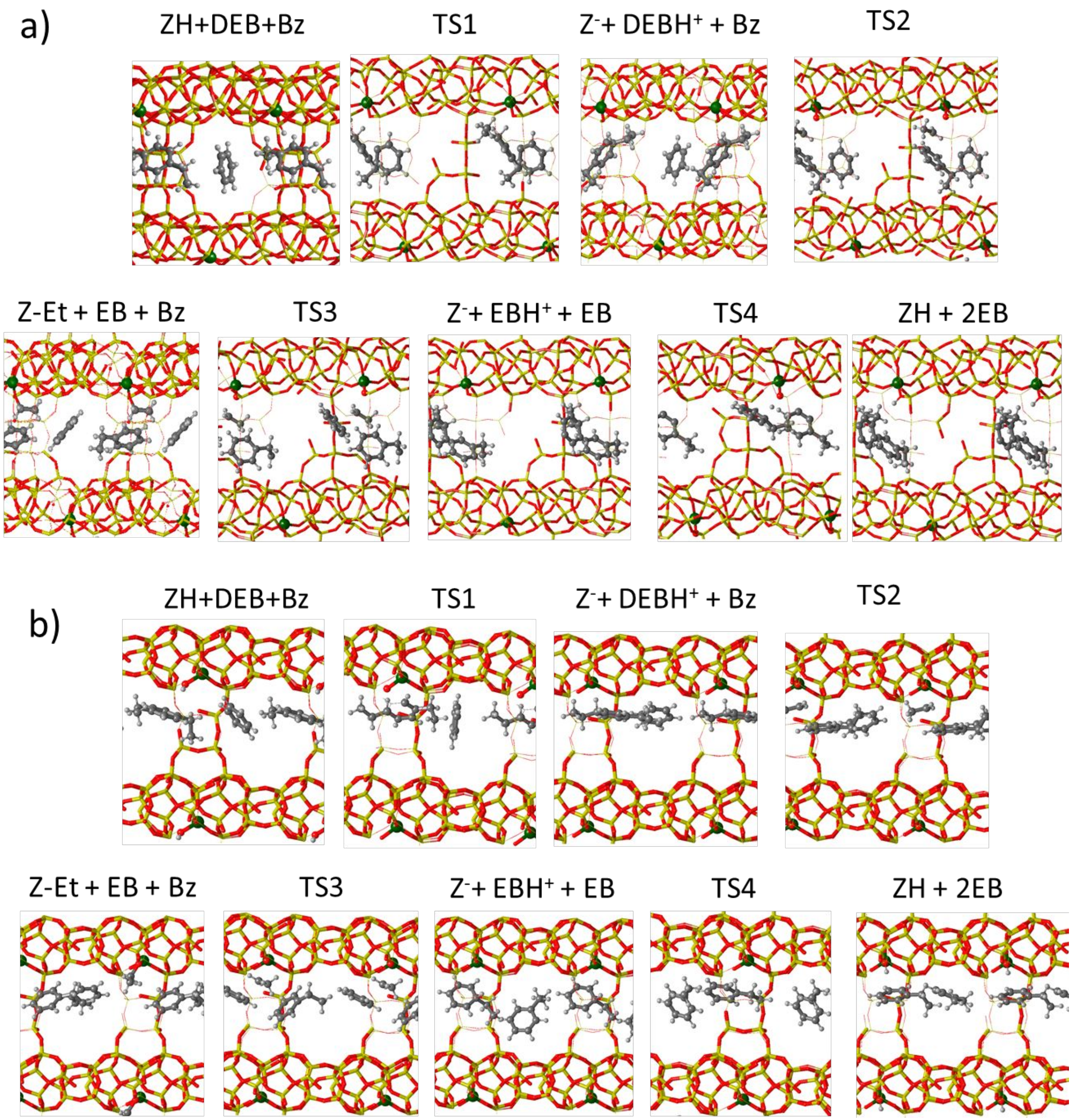

Figure S6. Figure S6. Optimized geometries of all structures involved in the alkyl-transfer pathway on (a) IWV-T3 and (b) IWV-T6 sites. Framework Si and O atoms are depicted as yellow and red sticks. Al, C and H atoms are depicted as green, grey and white balls, respectively.

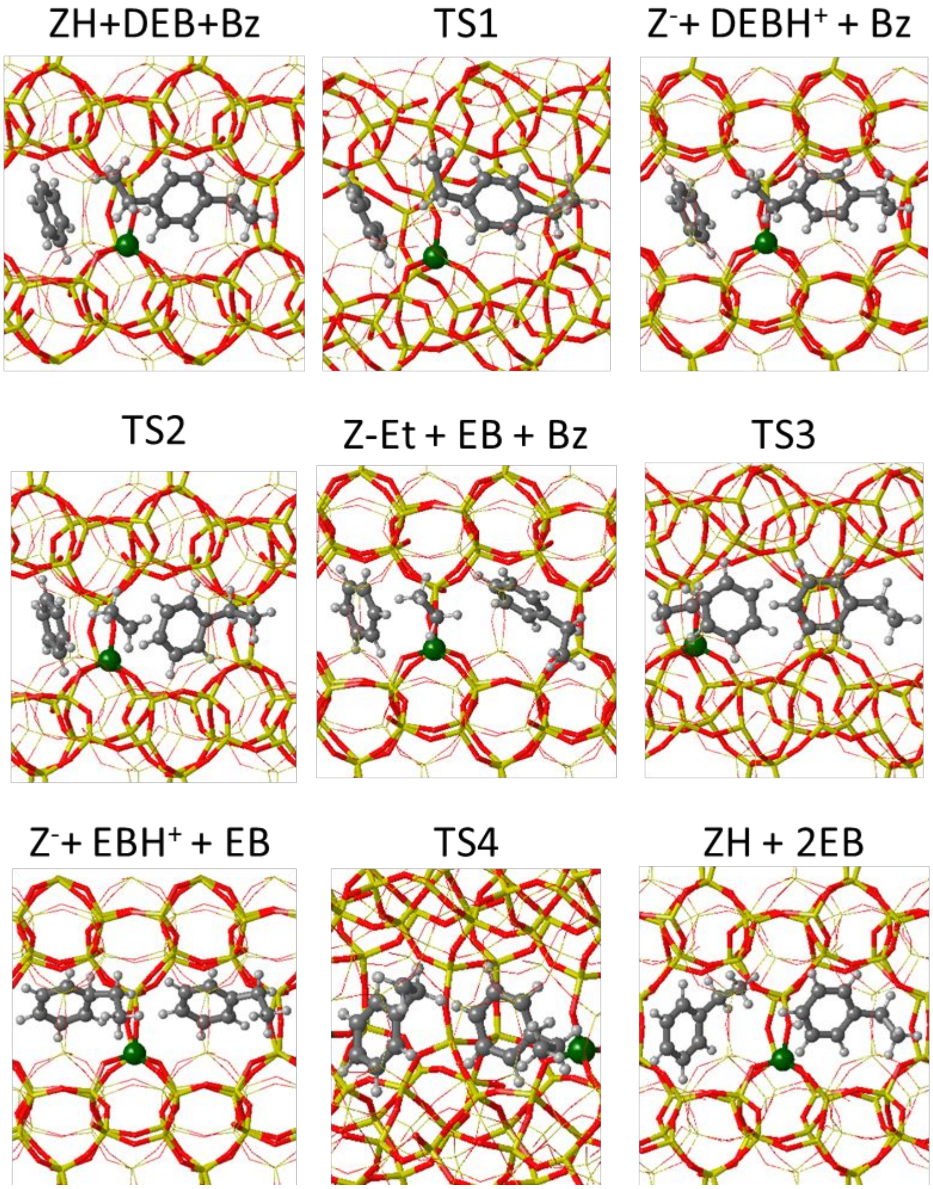

Figure S7. Optimized geometries of all structures involved in the alkyl-transfer pathway on MOR-T4 site. Framework Si and O atoms are depicted as yellow and red sticks. Al, C and H atoms are depicted as green, grey and white balls, respectively.

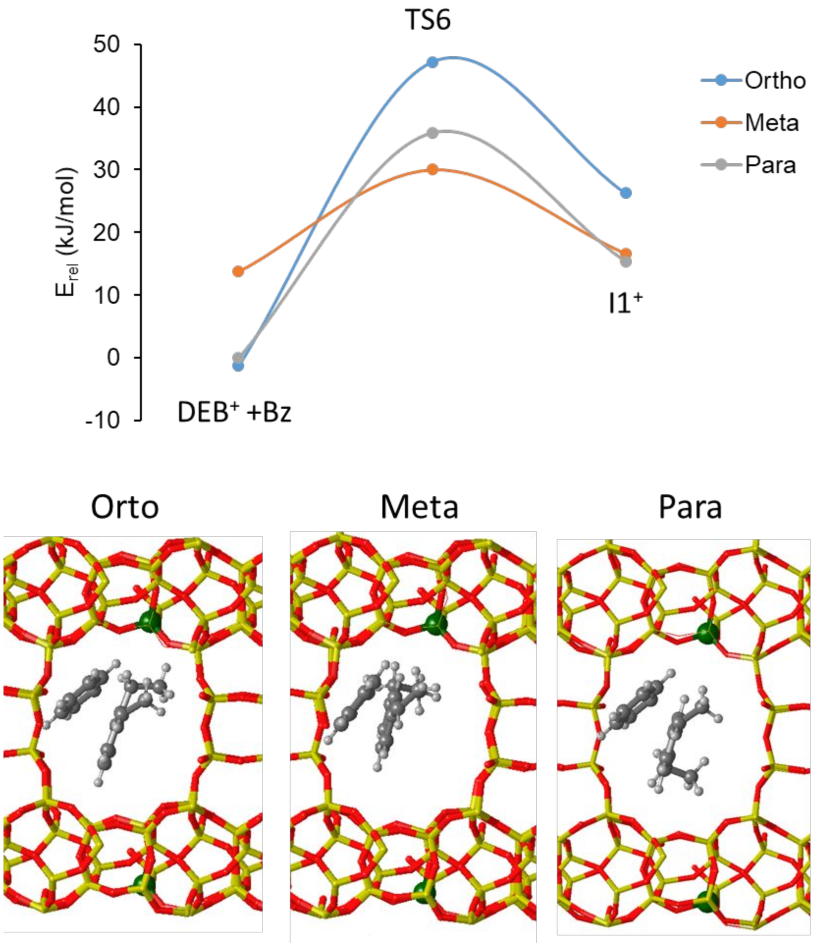

Figure S8. Calculated energy profile and optimized geometry of the transition state for formation of the $I1^+$ diaryl intermediate on IWV-T3 site for the ortho-, meta- and para- isomers of DEB. ies of all structures involved in the alkyl-transfer pathway on MOR-T4 site

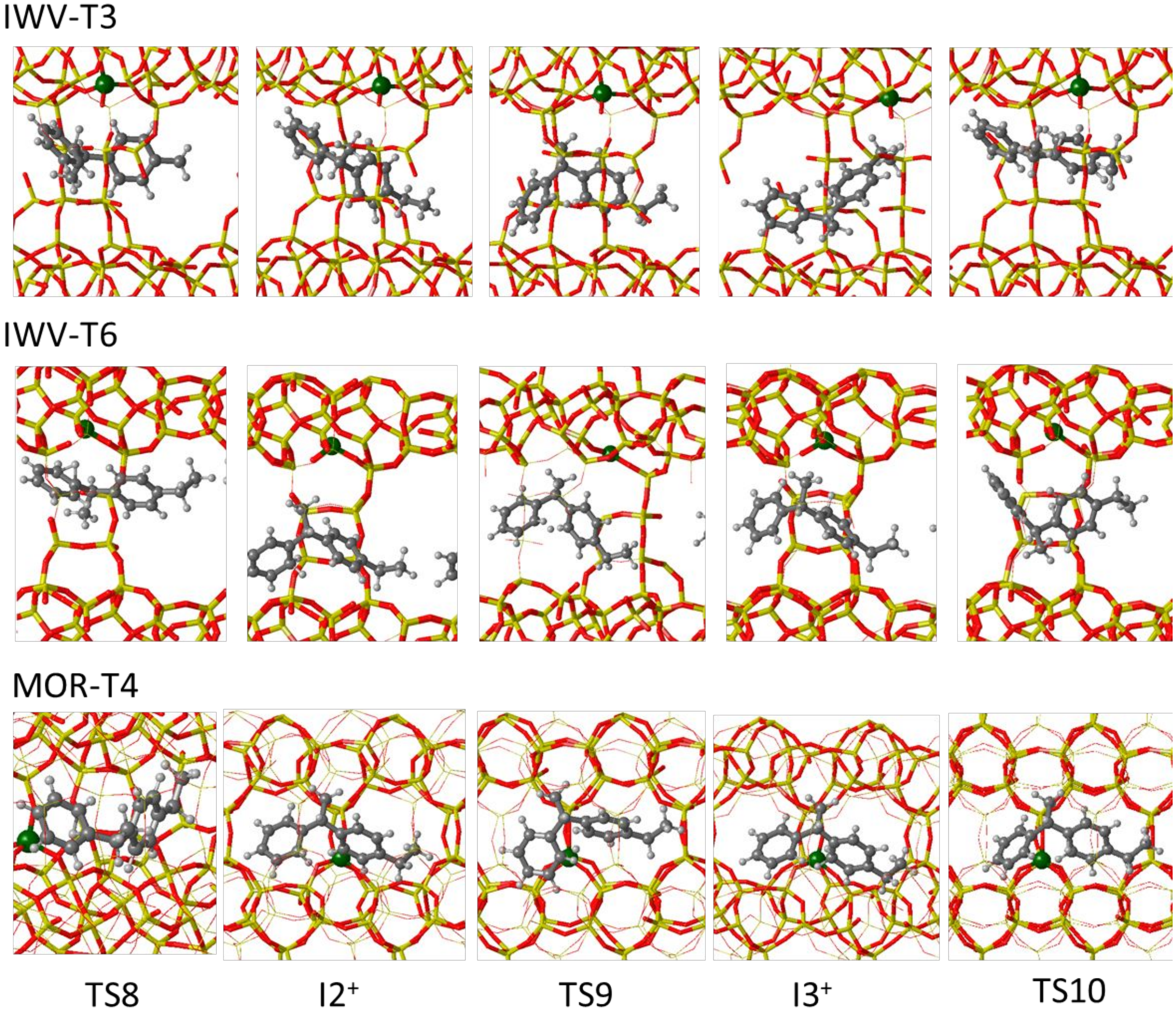

Figure S9. Optimized geometries of structures involved in the diaryl-mediated pathway (blue path in Scheme 1) on IWW and MOR zeolites. Framework Si and O atoms are depicted as yellow and red sticks. Al, C and H atoms are depicted as green, grey and white balls, respectively.

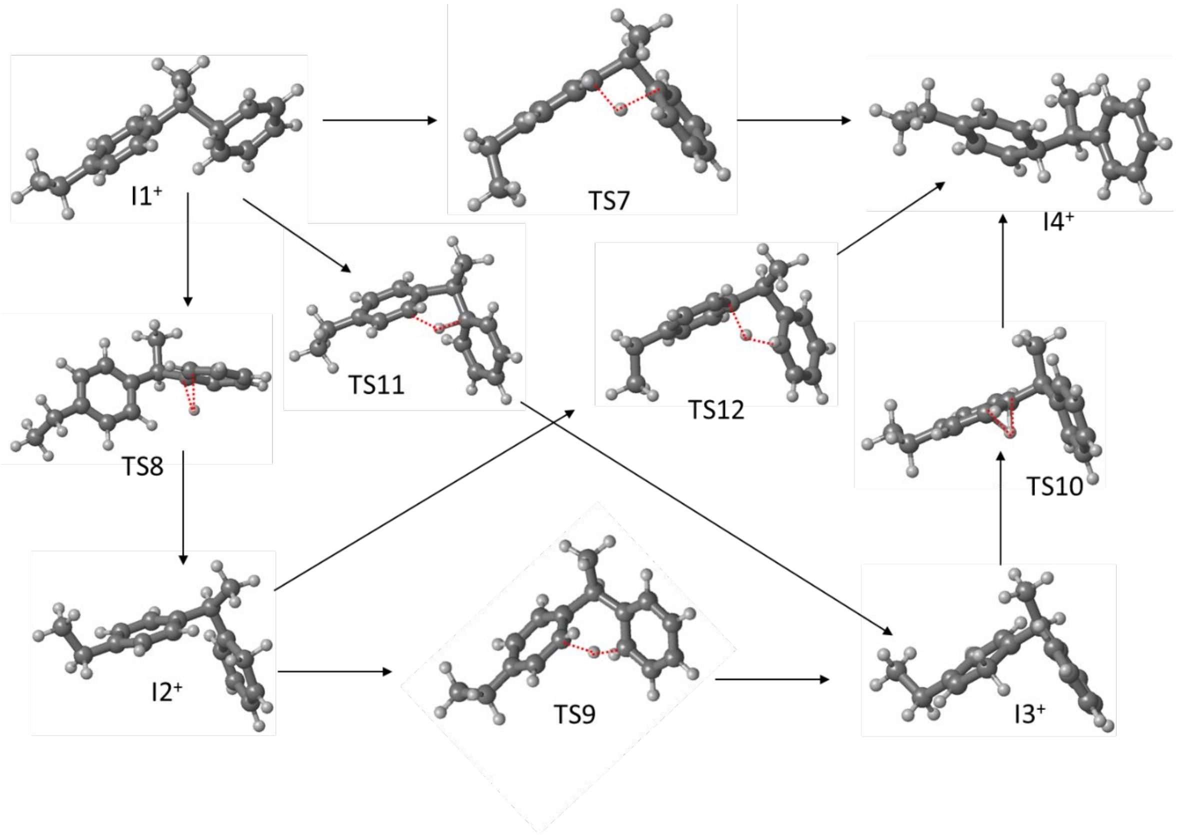

Figure S10. Optimized geometries of minima and transition states involved in possible pathways for the proton transfer converting $I1^+$ into $I4^+$ intermediates.

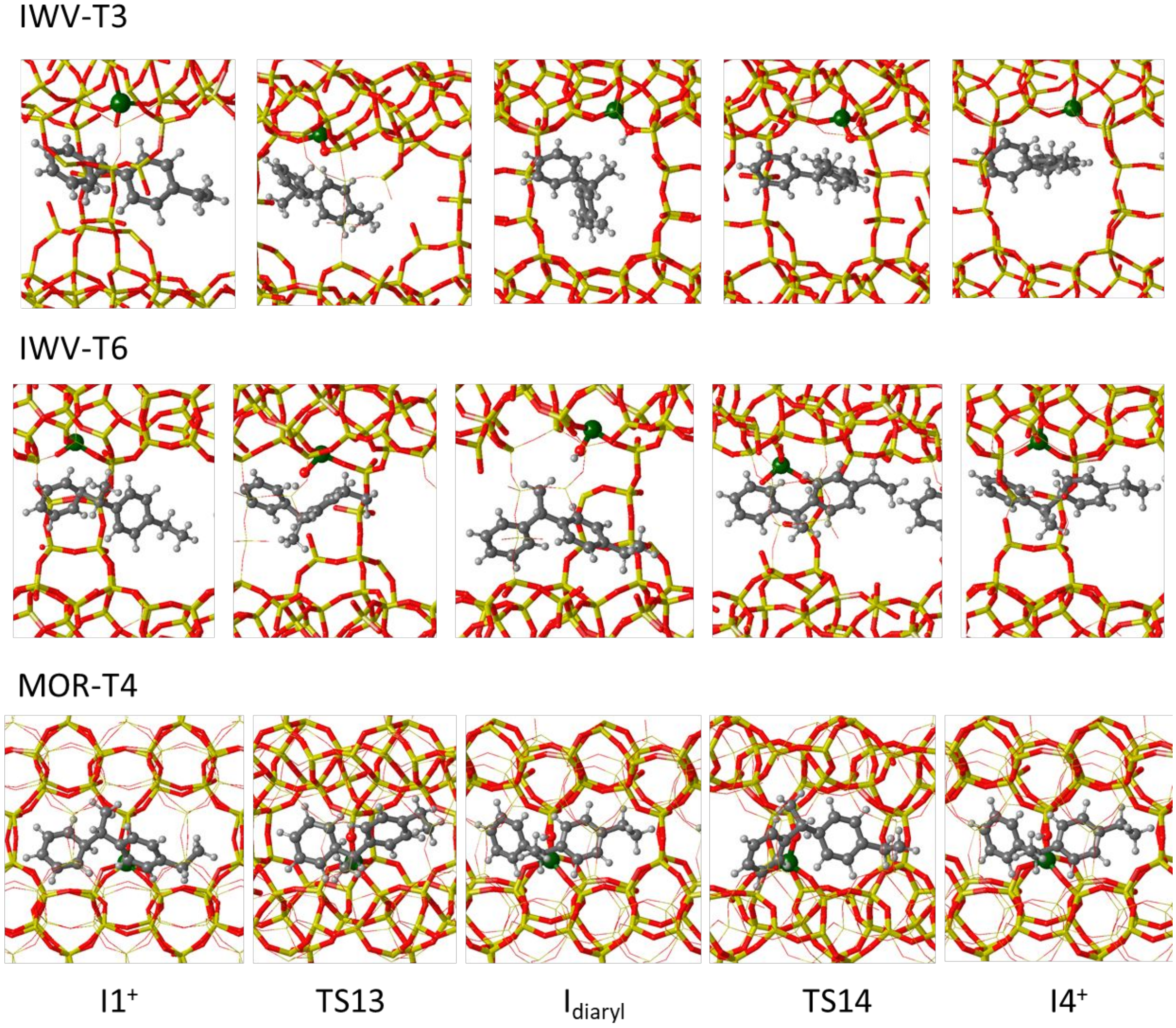

Figure S11. Optimized geometries of all structures involved in the proton transfer to the zeolite framework (gray path in Scheme 1) of the the diaryl-mediated pathway on IWV and MOR zeolites. Framework Si and O atoms are depicted as yellow and red sticks. Al, C and H atoms are depicted as green, grey and white balls, respectively.

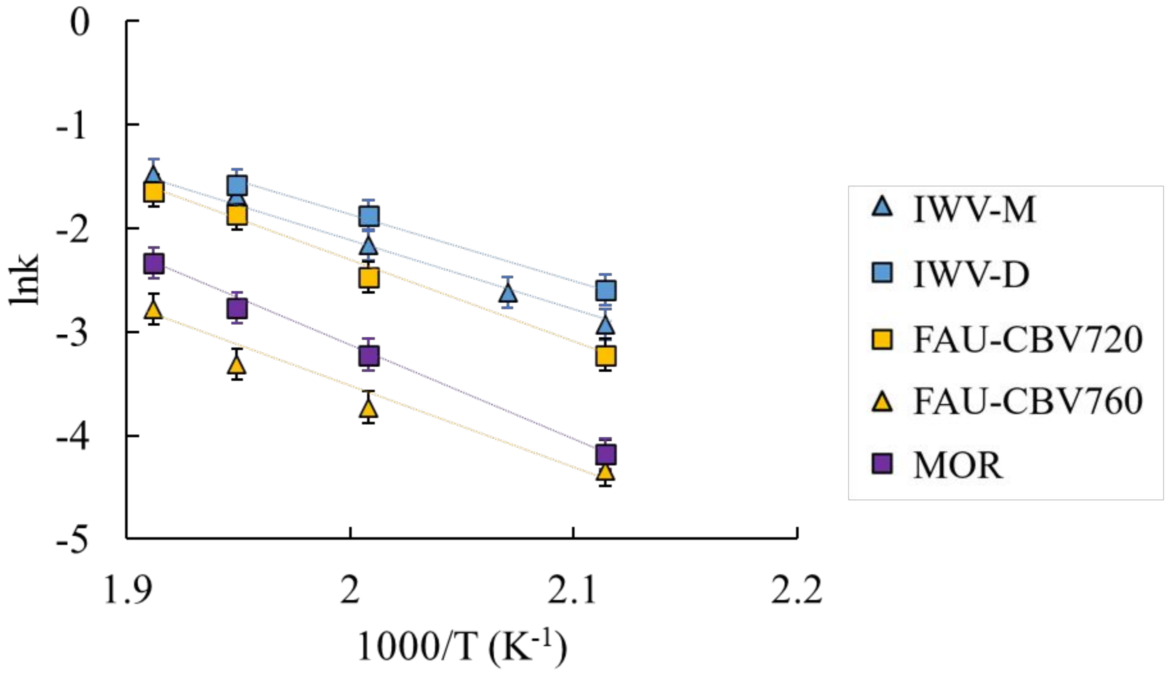

Figure S12. Arrhenius plot of DEB-Bz transalkylation over different zeolites. Reaction conditions: pressure: atmospheric, feeding composition: N_2 : Bz : DEB = 30 : 5 : 1 (molar). The error bars in the plot stand for the standard deviation of at least three experiments for each data point.

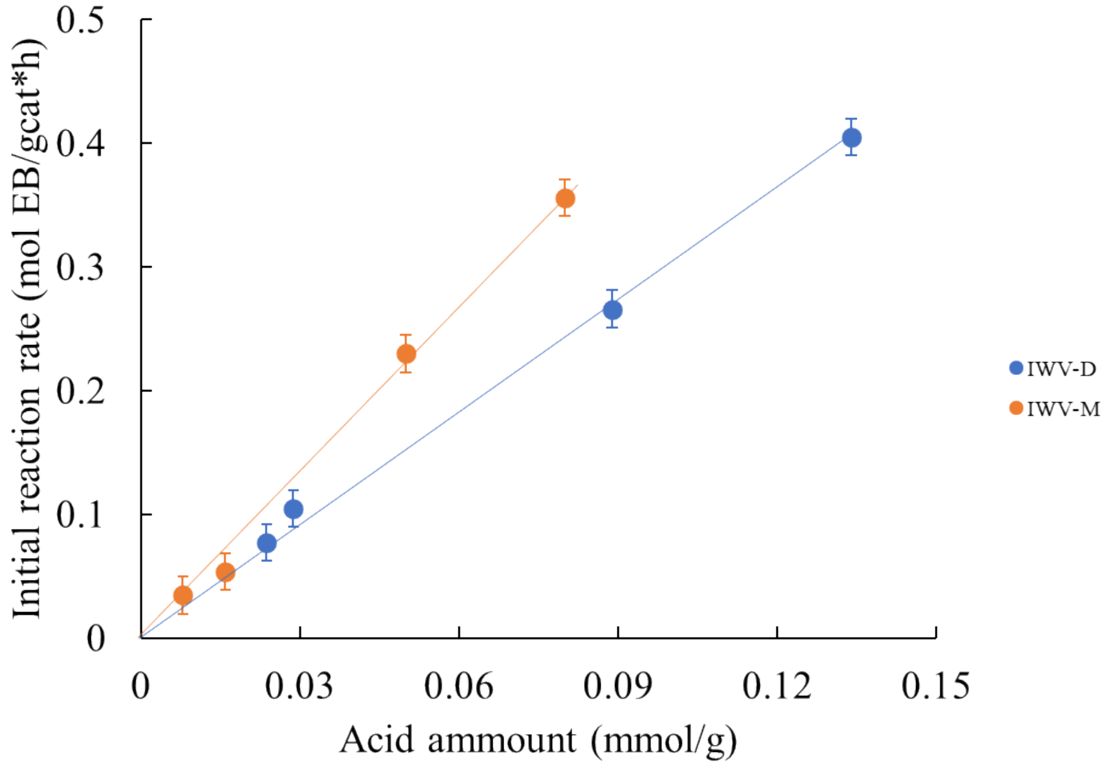

Figure S13. Initial reaction rate of DEB-Bz transalkylation for IWV-M and IWV-D with different acid amount by poisoning of Na^+. Reaction conditions: Temperature = 240°C, pressure: atmospheric, feeding composition: N_2 : Bz : DEB = 30 : 5 : 1 (molar). The error bars in he plot stand for the standard deviation of at least three experiments for each data point.

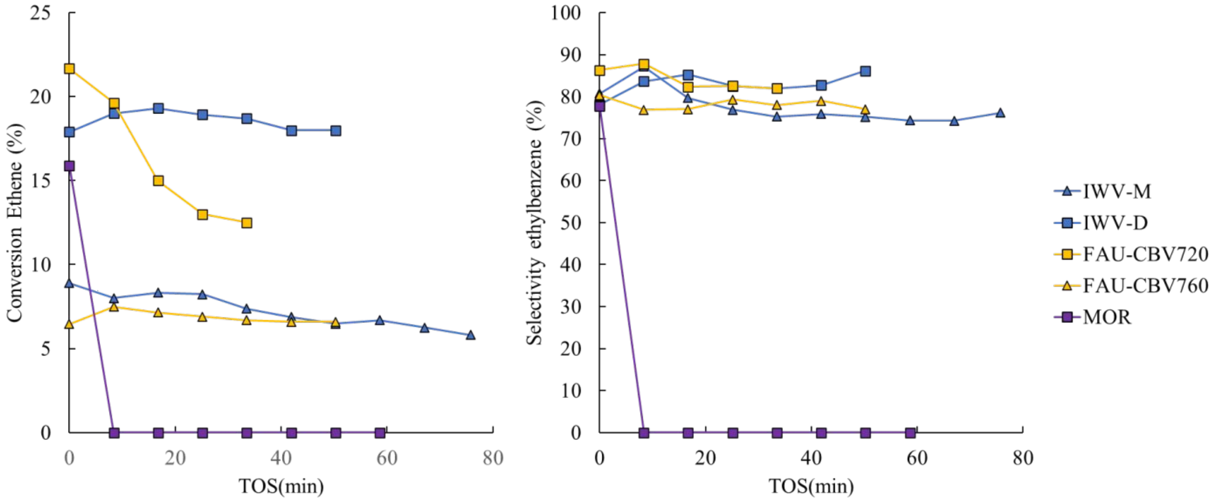

Figure S14. Benzene alkylation with ethene on different zeolite catalysts. Reaction conditions: Temperature, 240°C, pressure: atmospheric, feeding composition: N_2 : Bz : Ethene = 60 : 5 : 0.4 (molar), w/F = 0.32 (g_{cat}*h/mol $C_2^=$).

S3.- Supplementary Tables

Table S1. Physicochemical properties of the zeolite catalysts employed in this study.

Sample	Si/Al[a]	Si/Na[a]	S_{BET}[b] (m^2/g)	V_{micro}[b] (cm^3/g)	S_{ext}[b] (m^2/g)	Acid amount[c] (μmol/g) 150°C[c]	350°C[c]
IWV-M	23.8	-	460	0.22	20	309	175
IWV-D	13.4	623	566	0.26	29	453	279
FAU-CBV760	24.8	-	753	0.33	85	372	159
FAU-CBV720	13.1	-	723	0.32	75	478	296
MWW	20.4	-	424	0.16	81	348	225
Beta	16.4	-	577	0.25	73	300	201
MOR	12.7	884	406	0.19	11	733	361

[a] Measured by ICP-AES
[b] Calculated from N_2 adsorption/desorption isotherms
[c] Pyridine desorption temperature

Table S2. Initial reaction rates of DEB transalkylation in gas phase.[a]

	r_0 ($mol_{EB}/g_{cat}.h$)	$r_{0,nor}$ ($mol_{EB}/mol_{Al}.h$)
IWV-M	0.36	508
FAU-CBV760	0.09	135
MOR	0.11	86
IWV-D	0.41	326
FAU-CBV720	0.30	237

[a] Reaction conditions: Temperature = 240 °C, pressure: atmospheric, feeding composition: N_2 : Bz : DEB = 30 : 5 : 1 (molar).

Table S3. Relative stability of Al location in IWV zeolite, in kJ mol^{-1}.

Site	T1	T2	T3	T4	T5	T6	T7
Relative energy	11	13	0	5	11	1	8

Table S4. DFT calculated activation and reaction energies (in kJ/mol) for the elementary steps of the ethyl-transfer and diaryl-mediated pathways of DEB-Bz transalkylation in IWV and MOR zeolites.

	IWV-T3		IWV-T6		MOR-T4	
Step	E_{act}	ΔE	E_{act}	ΔE	E_{act}	ΔE
ZH + DEB → [TS1] → Z^-DEBH^+	48	44	56	53	84	54
Z^-DEBH^+ → [TS2] → Z-Et + EB	93	34	101	34	80	16
Z-Et + Bz → [TS3] → Z^-EBH^+	70	43	54	20	101	9
Z^-EBH^+ → [TS4] → Z-H + EB	15	-97	12	-109	18	-98
Z-Et → [TS5] → ZH + C_2H_4	115	46	108	36	97	24
DEB^+ + Bz → [TS6] → $I1^+$	36	15	42	25	87	29
$I1^+$ → [TS7] → $I4^+$	68	-19	68	-30	95	-25
$I1^+$ → [TS8] → $I2^+$	32	-8	5	-12	58	-20
$I2^+$ → [TS9] → $I3^+$	36	-8	40	-6	77	2
$I3^+$ → [TS10] → $I4^+$	42	-3	39	-13	50	-7
$I1^+$ → [TS11] → $I3^+$	76	-16	43	-18	113	-19
$I2^+$ → [TS12] → $I4^+$	77	-11	68	-19	86	-5
$I1^+$ → [TS13] → I	1	-60	17	-65	34	-73
I → [TS14] → $I4^+$	55	40	47	34	121	47

Table S5. Optimized values (in Å) of selected distances of the structures involved in the alkyl-transfer pathway. The rC-Of referes to the distance between the tertiary C atom of the aromatic ring and the closest oxygen atom at the opposite side of the zeolite channels, as depicted in Figure 5.

	IWV-T3			IWV-T6			MOR-T4		
	rC-H	rH-O	rC-Of	rC-H	rH-O	rC-Of	rC-H	rH-O	rC-Of
ZH + DEB	3.47	1.01	6.47	2.74	1.00	7.70	2.60	1.01	5.18
TS1	1.32	1.41	7.28	1.33	1.45	8.16	1.41	1.35	5.68
Z^- $DEBH^+$	1.16	1.78	7.09	1.19	1.72	8.60	1.13	2.69	5.36
	rC-C	rC-O	rC-Of	rC-C	rC-O	rC-Of	rC-C	rC-O	rC-Of
Z^- $DEBH^+$	1.56	3.48	7.66	1.55	3.43	7.91	1.56	3.35	5.36
TS2	2.41	2.18	9.12	2.36	2.26	7.11	2.40	2.24	4.02
Z-Et + EB	3.46	1.53	4.94	3.98	1.52	6.77	3.79	1.53	4.83
Z-Et + Bz	5.13	1.53	7.66	3.97	1.52	7.91	4.06	1.53	4.83
TS3	2.35	2.35	8.57	2.38	2.30	7.55	2.36	2.31	5.72
Z^- EBH^+	1.63	4.55	6.46	1.59	4.93	6.76	1.54	4.18	3.88
	rC-H	rH-O	rC-Of	rC-H	rH-O	rC-Of	rC-H	rH-O	rC-Of
Z^- EBH^+	1.11	3.26	7.29	1.11	2.58	8.11	1.16	4.11	4.99
TS4	1.45	1.45	7.21	1.30	1.49	6.28	1.36	1.50	5.68
Z-H + EB	3.83	0.98	7.41	5.60	1.00	7.42	3.22	1.01	5.16

Table S6. Optimized values of selected distances (in Å) and angles (in degrees) of the structures involved in the first steps of the diaryl-mediated pathway. The atom labelling is shown in Figure 6.

	IWV-T3			IWV-T6			MOR-T4		
	TS6	$I1^+$	TS7	TS6	$I1^+$	TS7	TS6	$I1^+$	TS7
rC1-C4	1.984	1.577		1.821	1.701		2.375	1.611	
rC2-C4	2.767	2.601	2.356	2.676	2.613	2.348	3.333	2.597	2.397
rC3-C5	7.337	7.738	6.000	7.105	7.484	6.425	8.020	7.816	7.271
aC4C1C2	105.9	114.7	99.5	107.9	109.6	98.6	116.8	112.7	101.5
aC3C2C1	177.0	175.7	164.3	177.8	178.8	174.3	168.2	169.2	171.9
aC5C4C1	112.5	149.7	175.4	115.8	122.2	179.2	122.8	133.4	170.1
aC5C1C3			87.1			94.5			113.1
rC2-H			1.463			1.492			1.488
rC4-H			1.436			1.424			1.420

Table S7. Sample information and reaction results for alkylation of Bz with ethene for different zeolite catalysts.

	IWV-M	IWV-D	MOR	FAU-CBV720	FAU-CBV760	No cat
Si/Al	23.8	13.4	12.7	13.1	24.8	
w/F (g_{cat}*h/mol $C_2^=$)	0.32	0.32	0.32	0.32	0.32	-
Conv.Ethene	8.9	17.9	15.1	20.7	7.2	0.08
Yield(%)						
Ethylbenzene	7.2	15.5	12.4	17.7	5.7	0.07
Diethylbenzene	0.2	0.5	0.9	1.1	0.7	0
Others	1.4	1.9	1.8	1.9	0.8	0

Table S8. Initial reaction rates of benzene alkylation with ethene in gas phase.[a]

	r_0 ($mol_{EB+2DEB}/g_{cat}.h$)	$r_{0,nor}$ ($mol_{EB+2DEB}/mol_{Al}.h$)
IWV-M	0.24	339
FAU-CBV760	0.22	330
MOR	0.44	338
IWV-D	0.52	415
FAU-CBV720	0.62	489

[a] Reaction conditions: Temperature = 240 °C, pressure: atmospheric, feeding composition: N_2 : Bz : DEB = 30 : 5 : 1 (molar).

S4.- DFT Optimized Coordinates of all Structures

S4.1.- IWV-Al-T3-Transalkylation

IWV-Al-T3-DEB+Benzene

```
   Al     Si     O     C     H
   1.00000000000000
    11.6056394577026367    0.0835597589612007    8.7649669647216797
    -3.6825602054595947   11.3529796600341797   10.3282289505004883
    -0.2346490621566772    0.2412826269865036   19.0520343780517578
   Al   Si   O    C    H
     1    37    76    16    21
Direct
  0.9021337195384366  0.7202070335609473  0.6877765822374833
  0.1399965998476803  0.2726462062877456  0.0312236505426004
  0.1105528211724607  0.1200605773241649  0.9978171735872160
  0.0930544987774203  0.3488982733400068  0.2668173107662427
  0.2403392168786030  0.1729888153682430  0.5462555798213260
  0.0967429236179517  0.1397502937615765  0.7627124915764973
  0.3286032707899806  0.1409691372888893  0.2623451526977652
  0.0561390716855050  0.5927224977496498  0.1218649212249017
  0.9928857457183003  0.7930529778762093  0.1132574749816397
  0.2794732435273927  0.3288941168508132  0.0777631695776443
  0.5576367941855038  0.0811943048359033  0.2305325420041582
  0.7625030195178830  0.0244986054368502  0.0934486267863709
  0.2785850006921910  0.3088976916299899  0.3128890230398588
  0.8489861105809408  0.7837749542723733  0.9297237660907345
  0.8699385134488964  0.9209550138413223  0.9841543909597519
  0.7461329201884220  0.8849800016635799  0.4251429004895234
  0.8885656827617150  0.8972721760452510  0.2194340566281057
  0.6711742495222038  0.9208580248967780  0.6935351816990925
  0.9465420359176254  0.4663089410406515  0.8347804302571973
  0.9860660538717566  0.2439576747193764  0.8746893540682574
  0.7071853830841861  0.7256085190562530  0.8893879862613505
  0.4410525248827881  0.9818280086821629  0.7333838380376591
  0.2187194949445447  1.0136234758638787  0.8880751343236203
  0.7021823866094431  0.7520589423887336  0.6577694752656160
  0.9178604427552143  0.5492458865702584  0.0618509797659856
  0.5028373694024789  0.9468923235230019  0.4697320538548626
  0.0969913756368221  0.5195802767958561  0.8739049585587391
  0.4876449155061003  0.1439137714598791  0.4844425425685697
  0.9378933118875576  0.0869416499592074  0.4659898197261598
  0.9050788118424856  0.1173044348969116  0.3057275663026143
  0.1106769474780516  0.9394319955716154  0.5159862896125661
  0.1210527238432785  0.9080039728094846  0.6938127064743308
  0.4822137290396739  0.5461122328458518  0.9197326379195107
  0.3223416135458500  0.7104686506474830  0.8905563958202868
  0.5297613898734200  0.4735345458831908  0.0915292989366639
  0.7104227287122687  0.3182944389020503  0.1066517971349839
  0.7520988063674797  0.2911737721543154  0.2638474965944176
  0.2758981906358977  0.7364696187743736  0.7381041733233958
  0.1748395560081379  0.1930183976411343  0.9975587764161310
  0.0148811733211951  0.2002962113340882  0.1644863879384408
  0.2460366444975753  0.3035294294009854  0.0244711863723776
  0.1235469733574924  0.3926646582802879  0.9375437678083428
  0.7986183523594854  0.0259684343967194  0.5843529582664420
  0.1714648763933145  0.2109119261474338  0.6228561273193021
  0.1845576312768911  0.0301274843302401  0.6401572911329778
```

0.2182894694408531	0.2460518828185198	0.4474986664975401
0.3830037457460032	0.2038869093385099	0.4803167399952317
0.0472058562760821	0.8101945526624558	0.5838834111113028
0.9905579460875537	0.6579124579740019	0.1708242419447037
0.1974748033633056	0.6664352415432394	0.0143197763671588
0.0363200662222467	0.4583034206394941	0.2332619186337563
1.0020596608841992	0.5926195371475254	0.0680137758984194
0.6061752960793536	0.6189971617260601	0.7850399817551068
0.6259694625627622	0.0199665020490258	0.1768558864380335
0.6580490403196041	0.1897990023934804	0.1635770588254879
0.4637568672512375	0.1307343519606636	0.2019269392062423
0.4858514883101438	0.9873913103099801	0.3746694777816908
0.5930751877225158	0.6060142034877809	0.0202572775401229
0.8128331775814026	0.8426388145778441	0.9846738207731544
0.9789164522887502	0.8660154872488631	0.7978073244010558
0.7486772052241594	0.7816752907162988	0.9174526774697320
0.8515236097575281	0.6480579049882912	0.0184980739497191
0.2335660004373550	1.0077912639968494	0.3798466466566521
0.8078616440246739	0.8402215999508550	0.3550597597007616
0.8427308362005356	0.9991300628004808	0.3538025180568072
0.7056903917691992	0.7751705168782986	0.5589798467358599
0.6325971765367105	0.9254397256961420	0.4280830204197241
0.9806747491944990	0.2276594133093838	0.3901536905372908
-0.0092142199374090	0.3824991449459114	0.8071313388072515
0.8200338926474786	0.3987387896346861	0.9648897311113591
0.9242457889865708	0.5741171074980957	0.7335998298215683
0.0459185283795030	0.5313030714135022	0.8116119494076123
0.3980064829665584	0.4187496827063021	0.2172384432918030
0.3553377147180411	1.0115301816567828	0.8161057174531737
0.3781724415586206	0.8529136748923201	0.7971637592068010
0.5652192656294071	0.9831732137819580	0.7150246638293843
0.4641173119825392	0.0807478176642537	0.6063884618634653
0.3989525940082170	0.4442249106247737	0.9619602449810580
0.9983198062475471	0.5307531256297122	0.9694980524763864
0.4942122559536256	0.0494267641660701	0.4723072847063974
0.8180010577942737	0.4221630654884444	0.1945640915805149
0.0392538452083838	0.1999991614534027	0.9451346585375338
0.3989587557716388	0.8231242624550258	0.6015073066447086
0.1770670556900383	0.0695073666559831	0.8058810014617402
0.2220044688352597	0.6260355293457961	0.7692501794349575
0.9329933865320468	0.8451235792775361	0.0470870727256656
0.6122024463925033	0.2527464462899253	0.3709399581549452
0.8158386965317076	0.9640920784005038	0.1660864427993144
0.0138230448956008	0.9942946797830171	0.1344242675591136
0.9533217532153960	0.0776738678503215	0.3791274032039912
0.8114360174286416	0.1926010069040112	0.3220856802383267
0.9711018740282638	0.0443198528351957	0.8463343966521462
0.1474813006173671	0.9305350449953831	0.5864751636425507
0.1774273945838168	0.8072759513810837	0.7545258753408040
0.1305851202014171	0.8783084856226644	0.0145354235798694
0.4099978110755038	0.6396858427501275	0.9225639390715645
0.3018400963755667	0.6826998917337531	0.8312845324061233
0.8474096263275663	0.1628906103329131	0.9699483515069701
0.6086960122062478	0.3846965235350544	0.1235395120661016
0.7645416457670350	0.2975161713907493	0.1693242851228132
0.2124155552311455	0.0954056692871112	0.9142205146952056
0.0685789453537726	0.2376670526298050	0.7734666864379192
0.9171545190566074	0.7907050680544617	0.2208919527735352
0.7638499987094315	0.9528201095167411	0.0590134966528020
0.0211623755670048	0.0229135754581952	0.5062089782943343
0.5133048344910888	0.4779560334548476	0.0116418100320757

```
  0.1681449761538734  0.3548822238683754  0.1560830664529629
  0.3155932094860391  0.2118745370327990  0.3008236833128994
  0.8137603674407834  0.6939663096088129  0.8171564423760980
  0.6555550196154939  0.8557399211912160  0.6594782180351482
  0.1830574445305913  0.3599176274356985  0.2865871443361541
  0.3064291202916811  0.2129104937702213  0.1636662748671636
  0.8310716984300377  0.7563836892068985  0.6277803316747839
  0.6587138862668109  0.8225118795766037  0.8150941796450044
  0.0222332310017863  0.5902470625807722  0.5169208022159354
  0.0666239870046841  0.4994558422551450  0.5224658343113255
  0.8976813516131303  0.5612453215925900  0.5998195581546837
  0.9898309430314899  0.3856219259775260  0.6059511511547396
  0.8209941578546972  0.4455746944449880  0.6857932467965822
  0.8656642838963550  0.3554847434124488  0.6904255257777121
  0.7836477852019682  0.2308050388680349  0.7827355153502528
  0.5160495106885028  0.9251160290569761  0.0821560348021969
  0.4617363349061625  1.0028153062059639  0.0955024421684902
  0.5221463397754382 -0.0806463288965864  0.0093723173855194
  0.4134953624765338  0.0748484543905411  0.0359643336666973
  0.4741145430324160 -0.0084291472411130  0.9496264971058817
  0.4198887573653805  0.0693384663209806 -0.0371064946226264
  0.1036904900720283  0.7157447449955016  0.4212732262324372
  0.8091163761770551  0.1748902914108368  0.7334345624812748
  0.1219869774960936  0.7881581689687536  0.3045142873627055
  0.1838195705335918  0.8784187804342423  0.2348189984114513
  0.1599917901347859  0.7445974081603188  0.2703669417482609
  0.0360098905118578  0.7953695737686031  0.3200888564015937
  0.7436198509589229  0.0863452876948643  0.8047919117704038
  0.8027634179059164  0.2304731707729589  0.6602875206988457
  0.8991614322831936  0.1641894206638476  0.6986330007020436
  0.1913653521612222  0.7130771687427687  0.4025648311856909
  0.0662278251834500  0.7614163795308907  0.4532968138783774
  0.1628032625385210  0.5188831986858715  0.4592594975347374
  0.8599791542698056  0.6322628288787654  0.5918566399632330
  1.0277141493156767  0.3186561826997986  0.6050822040778181
  0.7246588805535332  0.4263170190068480  0.7484185176596404
  0.5549504054253648  0.8700569574848260  0.1273852342911433
  0.4579689008959842  1.0078646178934494  0.1514958148079660
  0.5655976340896935 -0.1404915285885354 -0.0017637186758007
  0.3709998860130778  0.1354024314157136  0.0463666086770582
  0.4794296601471749 -0.0121533683142426  0.8921846332602643
  0.3823514660869354  0.1255482483487650  0.9164687019629802
  0.7915469884985477  0.1729200355372083  0.8551865447389821
  0.6899785582516048  0.2318316526160560  0.8249843302693977
  0.9211829764229577  0.5575857871313029  0.6918622920979673
```

IWV-Al-T3-TS1-Alkylation

```
  Al      Si      O      C      H
   1.00000000000000
    11.6056394577026367    0.0835597589612007    8.7649669647216797
    -3.6825602054595947   11.3529796600341797   10.3282289505004883
    -0.2346490621566772    0.241282269865036   19.0520343780517578
   Al   Si   O    C    H
     1    37    76    16    21
Direct
  0.8955663874001129  0.7038680063183149  0.6927832881148772
  0.1322562790567733  0.2624644106997137  0.0388787025714410
  0.1025649814219384  0.1065527100062637  0.0074667757085917
  0.0877797023010758  0.3397791252771628  0.2711806374657613
  0.2297046788675189  0.163163741654663 8  0.5539042876566965
```

0.0880327963518720	0.1293805958725041	0.7703151560559295
0.3226316765694980	0.1302901714394820	0.2693305480065910
0.0480055089690336	0.5833002584387534	0.1270427826549314
0.9855454230575259	0.7816008501975227	0.1206558299689255
0.2725381029326285	0.3176319481939688	0.0846268236869487
0.5528454668042633	0.0721592618810012	0.2338164723472558
0.7553012064661724	0.0114114067927418	0.1005817605465179
0.2737265863245697	0.2991272523078951	0.3180630238415362
0.8426046628441800	0.7677651479083789	0.9383481338815669
0.8628278847981617	0.9082588533446051	0.9913459659564210
0.7401244263535512	0.8764966394864963	0.4302136082479825
0.8804652732223895	0.8844991379405266	0.2278352003307198
0.6659429308168174	0.9131366474558876	0.6992203950122871
0.9412809610430832	0.4623384403472544	0.8346723195815428
0.9779358391469870	0.2320608264692197	0.8835757014584287
0.7017367285520585	0.7157966614701331	0.8931905327214662
0.4358885344252873	0.9723446676700950	0.7391682012898599
0.2114380032605262	0.0027348356665834	0.8955074145311931
0.6934269445700612	0.7436869267789156	0.6638768611400595
0.9076274442961366	0.5335361435681978	0.0715433096246547
0.4979100723666340	0.9377058917756458	0.4742126486847411
0.0909108146381172	0.5098177428462873	0.8789633607508582
0.4768441007235517	0.1315331975150377	0.4935319443932304
0.9321181484795824	0.0792014480379782	0.4716580788052842
0.8978324615604913	0.1049771292797955	0.3136470393463455
0.1029419048008253	0.9297814738850566	0.5215471541009012
0.1162437778202748	0.8964167999617397	0.6992894013919306
0.4719021303434639	0.5353875749964907	0.9288880358730118
0.3155989161265096	0.7010355914873803	0.8961080066285664
0.5245596130058104	0.4634434431457847	0.0969401775248294
0.7061122247115269	0.3096083052767527	0.1087750836115217
0.7421260451148277	0.2747329707259272	0.2725519013548704
0.2755997478823733	0.7273567447529609	0.7398613606644936
0.1640129811375599	0.1798404704434364	0.0086640802866154
0.0070387566709290	0.1934865137483217	0.1715046385840607
0.2396705776527651	0.2909346038541494	0.0319332213693475
0.1186401164304647	0.3832613583280311	0.9433747423940828
0.7940406760535257	0.0169041440592731	0.5920755986100414
0.1616476176046853	0.2017211663984349	0.6302213865536304
0.1702474745037254	0.0214342024027343	0.6473819168523467
0.2115221712792610	0.2393348093801791	0.4521851643481812
0.3716837420009064	0.1914038757256465	0.4910293947185175
0.0366131913439145	0.8040228844988740	0.5863432611593580
0.9825452856577700	0.6465046948215223	0.1783434387482979
0.1870763518269301	0.6633019367188444	0.0142757093596268
0.0346985717052870	0.4514995619137490	0.2359892423555818
0.9892048359266575	0.5780690632806297	0.0792831474059030
0.5927534179200674	0.6136141721174897	0.7922097319220358
0.6189432401059710	0.0068467376453534	0.1841829123271028
0.6552808341879416	0.1829788328032944	0.1612893174579662
0.4576330036898094	0.1202181097761027	0.2070009647509882
0.4824459238330250	0.9807734333988194	0.3774904474695898
0.5878409918396925	0.5960263914628751	0.0253125539580417
0.8081551223489952	0.8292364801447440	0.9909526877377646
0.9754067429638901	0.8440517875340381	0.8102437053905708
0.7458516779085779	0.7713529579474552	0.9203595339419922
0.8373910162464828	0.6297726850943096	0.0321889256225281
0.2281738755579507	0.9969759122372835	0.3863858969409748
0.7976393708713213	0.8276950623232215	0.3636673570351021
0.8444917401421785	0.9850480354766609	0.3580756362690987
0.6933074351732385	0.7668328945361311	0.5664377668871420

0.6321753905584672	0.9267395135281834	0.4264255868350596
0.9730804210722378	0.2206936296187100	0.3947439499081208
0.9829935408400435	0.3693852870428612	0.8174683674911539
0.8159068090707432	0.3945471384928018	0.9672372808627072
0.9225716122655188	0.5650168616413469	0.7335378399283674
0.0424261823070169	0.5210725623882245	0.8155847921339627
0.3926336347966656	0.4094398128325143	0.2226182846029813
0.3476195878101550	0.0000246315294903	0.8235574441651146
0.3766947891021154	0.8424646537217753	0.8022215860108525
0.5607647738230638	0.9769368379399819	0.7188808054051593
0.4566408635103066	0.0710780357692663	0.6133445287253914
0.3920006462838414	0.4329240583982373	0.9690135935772721
0.9915072889845287	0.5208558226241177	0.9748737569832417
0.4783972992303283	0.0340021814765191	0.4862313091433297
0.8110967758227862	0.4044431355336113	0.2032797069255318
0.0314801698424475	0.1843947692196575	0.9542535754557632
0.4000907613603645	0.8085423498606387	0.6035844826020733
0.1690092102468379	0.0611174714575171	0.8133781443502205
0.2156451793854738	0.6165826131025078	0.7754515357343279
0.9257478975716207	0.8338237216995219	0.0545747152836796
0.6020130620510880	0.2396617308257050	0.3772944582624579
0.8097302144291431	0.9514798139518073	0.1723468902385989
0.0060867131579462	0.9802212454418648	0.1443419995982849
0.9449630972991606	0.0677058301129979	0.3871421931057034
0.7971282298250649	0.1729290386094609	0.3347688383581988
0.9634408055452195	0.0325286224565819	0.8540110143757697
0.1389362916386118	0.9166918320427342	0.5946212035631268
0.1838608082862845	0.8051343193587355	0.7505533552659365
0.1230699556955980	0.8672808591685351	0.0217853037983115
0.3946537157215664	0.6237688804817532	0.9386715555965368
0.3018486799215272	0.6745803961215230	0.8327032977484214
0.8400829854995606	0.1498144392781151	0.9771722179163428
0.6023248480215055	0.3734531458085159	0.1298431907726239
0.7564324163615392	0.2821270671504219	0.1768630736068892
0.2059289240068781	0.0818622960192811	0.9242688282463210
0.0586440927876769	0.2282362879535234	0.7800351108885478
0.9100809450756591	0.7778556478982624	0.2293326755239499
0.7556612130629873	0.9393794909323332	0.0666504273879248
0.0194263261551924	0.0196526009089046	0.5073055304844798
0.5068052240608995	0.4674998039721884	0.0178542661376840
0.1605552945525756	0.3446588156378497	0.1614826712360657
0.3114742363228486	0.2007013707800645	0.3079517753973927
0.8054673255728084	0.6851045609812809	0.8189835458617301
0.6526885165035395	0.8499752695228872	0.6625694928491026
0.1788767822470215	0.3492440367866484	0.2905808345843888
0.2984531173396552	0.2025414974688438	0.1720524412421302
0.8220175220037749	0.7413492710937302	0.6337764193372100
0.6497032183722392	0.8134392406209485	0.8228567125882362
0.1424537317522756	0.6009460069693363	0.4106436273642655
0.0917416599219409	0.4816802984677340	0.5214488906307320
0.0777707344898552	0.6828669723837765	0.4059863755173698
0.9781721721972604	0.4441742035960308	0.6223950823465618
0.9639448552238942	0.6459062278052555	0.5064348154562611
0.9095550427637129	0.5247966948082144	0.6207648686356527
0.7716220623975791	0.4736748210741617	0.7081125274353480
0.4763663317165157	0.9085122771290941	0.1355834683130557
0.3786470634971955	0.9534603600550920	0.1541782788388629
0.5316240465562003	0.8934094156213753	0.0559328229396604
0.3368888321035108	0.9842992297469683	0.0921697001591198
0.4894969262100945	0.9237316989718412	0.9944131789045322
0.3924287926295824	0.9696108284278321	0.0122189750022604

```
  0.2596306798202518  0.6394208525448943  0.2980709806194686
  0.7144166502249383  0.3709364516145244  0.8408311083408034
  0.2397373903075677  0.6474742349262209  0.2229057243784390
  0.3268108139390650  0.6759798103591307  0.1408846457627215
  0.1867880760544916  0.5607299174478660  0.2732277871707845
  0.1925271234989259  0.7131176336206898  0.1967276139966838
  0.6178181463665706  0.3355613654433977  0.8934554099612226
  0.7521722747605805  0.2965976986075277  0.8590154254269513
  0.7254388356651787  0.4005894421333941  0.8743865857805854
  0.3100029002742355  0.5759641632826547  0.3202275593749552
  0.3137765195238330  0.7277352871999189  0.2437338985121348
  0.1412201537746864  0.4177050737913317  0.5262649672049664
  0.1181973217195214  0.7756703904348393  0.3207350750036892
  0.9382246459319878  0.3505180655407072  0.7056370713794672
  0.9134344349315908  0.7089533289053371  0.5020402632593101
  0.5100649597053845  0.8859454294249297  0.1825413127788356
  0.3354827028961098  0.9649602267882924  0.2164792862267172
  0.6076557848093584  0.8582423671283168  0.0415588883883266
  0.2623452392475095  0.0217910867194510  0.1045172937196273
  0.5327161169705461  0.9121420205024064  0.9321570854154743
  0.3611225877140720 -0.0046793820964789  0.9625621391939261
  0.7339665852087051  0.5468533153440229  0.6939989610347171
  0.7490680954113712  0.4445562939273013  0.6798296666257515
  0.9246388128042559  0.5451806747369280  0.6708461002791360
```

IWV-Al-T3-DEBH+Benzene

```
   Al     Si     O     C     H
   1.00000000000000
    11.6056394577026367    0.0835597589612007    8.7649669647216797
    -3.6825602054595947   11.3529796600341797   10.3282289505004883
    -0.2346490621566772    0.2412826269865036   19.0520343780517578
   Al   Si   O    C    H
     1    37    76    16    21
Direct
  0.8908017410723705  0.7033064617656271  0.6975958316243109
  0.1304110167479995  0.2626901170391378  0.0424663777748120
  0.1007812344344723  0.1091409262959157  1.0089300921944424
  0.0839517932376787  0.3402399730103497  0.2753807014677086
  0.2280868707050471  0.1641832251471349  0.5563698504843793
  0.0845646140569745  0.1300579395901716  0.7735509567880487
  0.3185202283636280  0.1297453814460574  0.2747256592312204
  0.0456334213099591  0.5841845763339559  0.1311359575388699
  0.9823382964502052  0.7829964536909130  0.1241101762097679
  0.2686304246887617  0.3177347597246864  0.0896172590561994
  0.5474047578507745  0.0712540599505898  0.2400107911983181
  0.7526563908354681  0.0139722741624579  0.1036218117128537
  0.2710334015406792  0.2993845753675752  0.3216753433390760
  0.8394029979476558  0.7693612482141219  0.9423362944543885
  0.8591724493463598  0.9096270392705302  0.9951777937661463
  0.7363394184333180  0.8775194433330918  0.4340257805785718
  0.8783150280079540  0.8867505504769304  0.2304685634965151
  0.6621500990627995  0.9134679241234820  0.7037706295306082
  0.9376743045284387  0.4628714610700608  0.8387204700009256
  0.9755944647434308  0.2350410563952232  0.8851581492537323
  0.6959681901625611  0.7190164715870673  0.8973706556604319
  0.4319387533899025  0.9728192272643710  0.7422091342869369
  0.2083993749779513  1.0039413140151174  0.8984032090089511
  0.6913308631914583  0.7421504220910364  0.6677493538652852
  0.9063774290347641  0.5354292918650640  0.0742961009564361
```

0.4942113305632441	0.9379905544020103	0.4779221106619761
0.0886761749413027	0.5099643075002447	0.8829368579833248
0.4747343642773856	0.1331816061643394	0.4954443534490124
0.9289571650053243	0.0800836772708815	0.4754263145461172
0.8953870805947099	0.1064246413697256	0.3170014960270635
0.0983096711814933	0.9304186235953306	0.5264268307099316
0.1126678126687948	0.8970031730918322	0.7037515276195223
0.4700964448191364	0.5357381014537168	0.9318959525248519
0.3125682579765842	0.7007643447872804	0.9006579385168427
0.5222996712338102	0.4652923412117868	0.0997537415169429
0.7027360321068914	0.3092342017698551	0.1140948516037939
0.7400795550155844	0.2770585100051234	0.2760701279162412
0.2711211487305988	0.7273884039116634	0.7452383267809287
0.1629009271365326	0.1816995098592145	1.0102904205738545
0.0044466421539224	0.1921651257918920	0.1751693321615642
0.2368880502753019	0.2892652331098182	0.0374506870266381
0.1180251236479280	0.3845072927318356	0.9472852270839336
0.7908364212349682	0.0166228637182207	0.5960196596179850
0.1594011796308320	0.2022127695051964	0.6332234655504434
0.1703752519573919	0.0222754677336293	0.6493387248149385
0.2082755644247299	0.2406549309519612	0.4556499577363484
0.3705662276772496	0.1943206433233930	0.4912957955012962
0.0309845823872736	0.8059275930329648	0.5904159212965301
0.9796178248380824	0.6478668006839737	0.1819099399016241
0.1854628914789563	0.6625440898568493	0.0201397622699236
0.0310452075339076	0.4521466532318250	0.2409828482016620
0.9883077836578588	0.5799730377334849	0.0818003083777409
0.5910871488924728	0.6114375797003663	0.7955895790347527
0.6161601563693986	0.0087436685513468	0.1876186495441215
0.6464642008318917	0.1810144259076751	0.1724723810625748
0.4532328579878930	0.1193840646294927	0.2117503473568463
0.4755904684581796	0.9771438377901519	0.3843429357372079
0.5823582803320744	0.5985415592044623	0.0294660257245342
0.8033258222853960	0.8295213854407186	0.9964803248164825
0.9717572560767389	0.8482415591590639	0.8135807230035417
0.7418115519018074	0.7717906947563734	0.9256429640362661
0.8367700746617344	0.6321404620319757	0.0342755583882831
0.2240564925462594	0.9966246215372091	0.3917652200488441
0.7966595104010190	0.8297504642981788	0.3663393768257224
0.8388231405202405	0.9861945948799821	0.3638346066300409
0.6882576813475507	0.7675793942677168	0.5706522847710345
0.6284291495032792	0.9264818257863695	0.4292523606026198
0.9686987745737504	0.2216930201921053	0.3989731758071086
-0.0182201149030700	0.3727484063159615	0.8169714004868363
0.8138845399630582	0.3870954028612780	0.9746159312950902
0.9159789163266663	0.5658452955201591	0.7430083840116739
0.0393343236518324	0.5179594343961322	0.8221133493940654
0.3899965735642805	0.4098146522027294	0.2260455701431623
0.3444518806806794	1.0007684365141796	0.8264903118933076
0.3728520710819128	0.8424623675645329	0.8062195533275638
0.5580862982564548	0.9791786099887950	0.7192738329831674
0.4503032402119173	0.0703383408857166	0.6176860958677743
0.3882295866037325	0.4328536915168481	0.9740192482566999
0.9897914821973179	0.5214503186798228	0.9787754457168668
0.4784141448786630	0.0377877311724313	0.4851807817691962
0.8086187848501805	0.4070890135981164	0.2066314661434930
0.0308014151651541	0.1874618769993273	0.9545864261317770
0.3963665788854718	0.8101956454079842	0.6094299480424941
0.1653766150342270	0.0622773095550034	0.8169026453830565
0.2131404769797405	0.6176839059439196	0.7783677842331702
0.9218352738602915	0.8350957755671797	0.0587244990093351

0.6001531494351123	0.2408006633613846	0.3820686027548991
0.8067686465571975	0.9527876664891534	0.1758347404730236
0.0038870938318620	0.9831179992607563	0.1461510694442880
0.9432327035487185	0.0691486695596686	0.3900042721960342
0.7967292999744898	0.1764440330630987	0.3368482410878300
0.9604892568063224	0.0327430857767464	0.8570027870841118
0.1341275816151706	0.9163198550932610	0.6000561953877297
0.1780159806373596	0.8039519116799415	0.7569464869297436
0.1201322502744588	0.8684305202104337	0.0248562458612847
0.3942175679451301	0.6254658332189772	0.9401646758028587
0.2958651816836942	0.6731833308945373	0.8394550037068135
0.8371822943529754	0.1523958058173628	0.9808248216827102
0.6023887081039152	0.3778487088110413	0.1304883877670084
0.7530721968884548	0.2836651152577596	0.1815216150089309
0.2037601723208748	0.0830015434431405	0.9269264756054267
0.0540497531481284	0.2291642516041316	0.7828705103244366
0.9080365893168399	0.7797377068335878	0.2324289748433017
0.7532002243016779	0.9425558813308370	0.0690089533235729
0.0170277693928529	0.0224931128084072	0.5103723002207750
0.5048777117639023	0.4686852257661803	0.0208427082246036
0.1562484219582178	0.3459470981964686	0.1653225152157748
0.3089776290022797	0.2001653602623970	0.3129326925612685
0.7982171026680484	0.6909725057909034	0.8202131721209456
0.6499720417950269	0.8461904215477510	0.6703097757790968
0.1760802786387957	0.3490961866561133	0.2942021807640057
0.2934985673898891	0.2027587527148850	0.1781124055780450
0.8211678714118772	0.7399917995312180	0.6345702580273228
0.6426475001665297	0.8180343462505228	0.8292252399886211
0.1457250481972868	0.6050045894107229	0.3935969168947027
0.0954424489033740	0.4858038087477474	0.5055053517037824
1.0835729439567039	0.6895104363402211	0.3874696154937145
0.9858831022157067	0.4511618798746724	0.6071007289161707
0.9739068809603969	0.6559136244971787	0.4883830522420438
0.9191474610985163	0.5346044770038745	0.6056220951096698
0.7802878242088019	0.4839034659093697	0.6869031531354142
0.4736442740724150	0.9036305375195732	0.1385607052426173
0.3772276057400990	0.9505154898477873	0.1568275096996925
0.5287975081914614	-0.1089928560511550	0.0570733106600002
0.3366893970872849	-0.0142084645332301	0.0926426713492102
0.4878223007180651	-0.0743314158868508	0.9934379746674707
0.3920875380782239	-0.0264719932946148	0.0108982948261906
0.2612342415939812	0.6415268248431407	0.2819117621733216
0.7197023190413477	0.3813096261837043	0.8203015613148252
0.2393880101071447	0.6522833198576201	0.2063836145377632
0.3262365666618362	0.6796581531242438	0.1248555492737822
0.1847404746015168	0.5666200860469726	0.2564444447134473
0.1939555787801602	0.7197845600370303	0.1796997573398472
0.6231666789602187	0.3488599819770550	0.8706141933176262
0.7549474523289661	0.3052255502891887	0.8405948530451308
0.7312551445177548	0.4109190481813064	0.8536520628906787
0.3101638402911292	0.5764050202916019	0.3040491068311598
0.3170650234237436	0.7291533234698265	0.2274137416032557
0.1438184076461372	0.4212244335335839	0.5099608792285576
1.1249687945391551	0.7814660677222646	0.3009808046930398
0.9470786754744500	0.3588766543000457	0.6922903146564227
0.9257546153071803	0.7202751270008058	0.4851329867239150
0.5067740933449980	0.8780798717378956	0.1868276758911449
0.3344943325879310	0.9605835525514257	0.2201541638134000
0.6039151079948137	-0.1454814457192044	0.0428117737330859
0.2635019872795601	0.0252100912326793	0.1043867091778948
0.5308948683283742	-0.0841621025014485	0.9299054804954519

```
  0.3617683013109969  0.0027626441899853  0.9595642798124046
  0.7435124054148053  0.5580992860607304  0.6708776867207038
  0.7633555323370730  0.4564049019911929  0.6545689399551899
  0.9267092517206286  0.5477143897678828  0.6556173595069213
```

IWV-Al-T3-TS2-Alkylation

```
   Al     Si     O     C     H
   1.00000000000000
    11.6056394577026367    0.0835597589612007    8.7649669647216797
    -3.6825602054595947   11.3529796600341797   10.3282289505004883
    -0.2346490621566772    0.2412826269865036   19.0520343780517578
   Al   Si   O    C    H
     1    37    76    16    21
Direct
  0.8906516866387526  0.7094478446087701  0.7010832044370041
  0.1314105994587649  0.2695361917412913  0.0445118134582276
  0.1026702277227968  0.1160905160250611  0.0104509533964748
  0.0849351130609012  0.3457210797955731  0.2784219843765746
  0.2294688597713475  0.1710250826723796  0.5576630010561781
  0.0857202618105919  0.1379621841753064  0.7747817996782885
  0.3175256476825182  0.1343311637496681  0.2783020394230957
  0.0450364341036284  0.5887498542783436  0.1366094222671422
  0.9832135768986578  0.7894082652150594  0.1270603946942417
  0.2691269175430025  0.3230537332380728  0.0927904025479024
  0.5462724932686587  0.0764797526812031  0.2442152550352429
  0.7541032244876036  0.0227524168597838  0.1045680680886418
  0.2725597091110316  0.3061127324336562  0.3229960106951363
  0.8402285041340933  0.7774975724101212  0.9441733693781309
  0.8602430133356306  0.9158795560116363  0.9982056065511848
  0.7360471570872967  0.8848182920034336  0.4366373435179457
  0.8794991080209866  0.8955854004121421  0.2314668567959786
  0.6621919513152543  0.9192410554112328  0.7061001308740676
  0.9377088206528984  0.4672687615245673  0.8445304343054907
  0.9773720977912804  0.2424668964572906  0.8859727452963344
  0.6967870140259791  0.7266794623525168  0.8994282579433845
  0.4313430709476970  0.9775678612278296  0.7458074206652359
  0.2096978184336296  0.0114298699446246  0.9000453979188627
  0.6916823897066716  0.7473114824426619  0.6703042206434834
  0.9048440085475886  0.5427668179434073  0.0789881450502254
  0.4930344205415991  0.9434542180628217  0.4814355314005285
  0.0871893465737530  0.5164903119052630  0.8882030322606632
  0.4742976428320274  0.1385504667006685  0.4985340738099432
  0.9302540714371799  0.0837753228669248  0.4796410149648701
  0.8975808337042844  0.1125967047140163  0.3200236403448841
  0.0986165926684494  0.9328396970256010  0.5317628052331193
  0.1141570799854407  0.9023647061020745  0.7070621127515828
  0.4728035781579263  0.5408924151612484  0.9347051817081053
  0.3131900428562193  0.7041982603913131  0.9057590606682969
  0.5254722461433750  0.4726540672953214  0.1015099193034257
  0.7029747305910696  0.3135264259335568  0.1197923606777368
  0.7415554522559675  0.2830296889160983  0.2799260043860835
  0.2715621081672152  0.7326807961787367  0.7496641408745340
  0.1647658518645208  0.1905259599438315  0.0102140522067157
  0.0072333541446531  0.1960131574949583  0.1787380391330863
  0.2389335422256317  0.2991556279137694  0.0368374464600791
  0.1145029720045431  0.3898691202553202  0.9523552836131751
  0.7910494502444942  0.0221922521394756  0.5982886304859170
  0.1593733987559100  0.2076366840778449  0.6355024338363848
  0.1738335021576774  0.0290214863576272  0.6490508324418645
```

0.2095763441968012	0.2473636360441846	0.4569964648876330
0.3720136904901021	0.2023398753536977	0.4925004524398404
0.0326881096301787	0.8078989092809130	0.5951032803440282
0.9821341563507151	0.6541631860165390	0.1857860538877577
0.1857507735202441	0.6634674548584240	0.0261928362983622
0.0284212513321048	0.4552423478453763	0.2465596068054540
0.9853455789095715	0.5873097389730750	0.0875963466165638
0.5924441500635506	0.6155911111981629	0.7976558042682702
0.6175543316170753	0.0171843831405814	0.1887544928153338
0.6426663540974067	0.1857646133038398	0.1802737386079025
0.4527267073115302	0.1249366309796940	0.2150404499693859
0.4742159895742322	0.9801076009833324	0.3887685850738862
0.5838583734109306	0.6059510381626168	0.0322138745824692
0.8034841170228220	0.8371389588189327	0.9988295668649423
0.9734254010155121	0.8557381488248269	0.8163279589356480
0.7440053870522703	0.7811230216294291	0.9255649650025340
0.8363895213835085	0.6397687737295488	0.0367760801568352
0.2236877558304165	1.0009832953163806	0.3962847491943809
0.7967785790818962	0.8386915167456476	0.3674259923076303
0.8369365938015011	0.9935623270242567	0.3679311504205036
0.6890929502626145	0.7734272512403583	0.5727599796967379
0.6274877124214118	0.9328827110707493	0.4328949020553050
0.9723571360213062	0.2250291695805788	0.4030713507340321
0.9857730350597220	0.3805737526615613	0.8189322835544552
0.8138442561476531	0.3874380165774611	0.9806955495899621
0.9108192626714577	0.5680450651101812	0.7514238786598710
0.0363390061932607	0.5270534381680509	0.8269951312271684
0.3924883300545143	0.4152094684614733	0.2276275220146461
0.3453909407160762	0.0074877063346834	0.8284195804394816
0.3716112028389251	0.8459097845896530	0.8127624722340097
0.5587241433070581	0.9855668877101784	0.7203757808695329
0.4477055040288611	0.0728338392815833	0.6223772646391692
0.3903966368443681	0.4361171489540183	0.9795270213409211
0.9897527479237325	0.5280625344939498	0.9840240447173456
0.4765987744969645	0.0446222249816037	0.4868982708232136
0.8063969293830653	0.4144381701454025	0.2107304423989440
0.0326337390205793	0.1928584688525215	0.9556747132763005
0.3961721885223342	0.8155222648445545	0.6135076398833742
0.1672661286882535	0.0720053999934440	0.8177433134136632
0.2125689817177320	0.6230158775549267	0.7832798337646367
0.9212977018790376	0.8390017432228205	0.0631359419871234
0.6012957881443821	0.2437894359274395	0.3870967765938456
0.8089074458364411	0.9629099798096562	0.1759924276753037
0.0052269829539892	0.9908993428702735	0.1482127985210158
0.9457474894192750	0.0736948035653246	0.3932313153388995
0.8018860444613360	0.1858164654908047	0.3379440091423912
0.9622187828151549	0.0385394899327046	0.8601690490693294
0.1346549569926324	0.9178783112899930	0.6055955815520747
0.1786431967695979	0.8093446657813269	0.7618110538884023
0.1206796650176922	0.8761015424840330	0.0259843077207936
0.3967679677194140	0.6303650184490052	0.9430527921249245
0.2973446822283026	0.6778162013050840	0.8433026120855019
0.8378132444663492	0.1617876818289387	0.9816656870657081
0.6065595478689330	0.3865443988538466	0.1322164147294769
0.7550480015677750	0.2883792916684457	0.1856988142125633
0.2060390490386030	0.0891460425383027	0.9295563319862963
0.0542170471385436	0.2376862629622296	0.7832087277126150
0.9085136526795018	0.7882287932942914	0.2340253666093886
0.7551447872250305	0.9504779073469406	0.0706925852461567
0.0148819953509631	0.0225509513727319	0.5185872334715665
0.5113997850820343	0.4765445558130683	0.0204844162503474

```
0.1569427031791528  0.3516996240827310  0.1679490129655785
0.3082534740186669  0.2060788672933884  0.3152002790722332
0.7988018238915873  0.6977308415330652  0.8229541644707232
0.6496198628516421  0.8505611685091201  0.6739562035677229
0.1788316813133219  0.3577830623293281  0.2940860709076476
0.2911209211780552  0.2057163457377403  0.1827643154157703
0.8213325771826294  0.7456073719787990  0.6380444645344332
0.6427365486295857  0.8243680847282342  0.8316036193616484
0.1352764724003956  0.5914467758567744  0.3729254806098839
0.0750567287595876  0.4692432706367879  0.4773721438275979
0.0698464703804853  0.6736829147809287  0.3632061833378591
0.9526144332107589  0.4296501670820901  0.5683733232571492
0.9475372386474028  0.6350806283260283  0.4531402040991752
0.8873571241805547  0.5123603153166876  0.5576530801203661
0.8739797868194386  0.5136593277442599  0.6890775924024771
0.4599095356181478  0.8997054223244492  0.1549486797754309
0.3622827277770478  0.9445122793667788  0.1704101747645127
0.5240224803488650  0.8908666472456498  0.0701067209119087
0.3294725670990573  0.9814059456911607  0.1001269423665085
0.4909235778425959  0.9273999376745042  1.0001983032510542
0.3939144757876961  0.9730981802125148  0.0149167723820344
0.2655021771292799  0.6342040943882905  0.2704857118650686
0.7618109827219093  0.4129428835310406  0.8022730117269063
0.2808859141190627  0.6620138652577541  0.1690872773424687
0.3765771800146274  0.6930061350139652  0.0951317890256722
0.2374257073487067  0.5822283105721036  0.2013695312123386
0.2410826020289743  0.7322506663514676  0.1340258001904251
0.7722223028070044  0.3286374509498884  0.8251396448736235
0.7378081278417248  0.4002896158325295  0.8761485913139686
0.6863213564339545  0.4325672993413372  0.7996141455062982
0.3084781735818349  0.5659163418089956  0.3021404971121592
0.3113257861585826  0.7156419330958809  0.2344442249135630
0.1251262044710775  0.4050022353785946  0.4862690212776057
0.1168574498792371  0.7686011548911846  0.2828794486142453
0.9067525572001728  0.3345100950723917  0.6480821908787436
0.8968346370637483  0.6987477543817261  0.4440042511309495
0.4867592436745805  0.8724665532968524  0.2083653175936240
0.3122363669029510  0.9512507215276653  0.2366850700031805
0.5999470511320230  0.8557838695771718  0.0582024291020874
0.2547416210693439  0.0184422809294766  0.1105033691207603
0.5407409752560218  0.9201527442738383  0.9343199223887777
0.3692988994228393  0.0031791727574305  0.9591831584749806
0.8704318303067372  0.6040998600332920  0.6339911733000987
0.9612218635534386  0.4992567212016030  0.6581662225430578
0.7897670363564059  0.4813010314979800  0.6207510832172747
```

IWV-Al-T3-Et+EB+Bz

```
  Al     Si     O     C     H
  1.00000000000000
   11.6056394577026367    0.0835597589612007    8.7649669647216797
   -3.6825602054595947   11.3529796600341797   10.3282289505004883
   -0.2346490621566772    0.2418262698650360   19.0520343780517578
  Al   Si   O    C    H
    1    37    76    16    21
Direct
0.9003488427843002  0.6947825622560213  0.7116069226952626
0.1370417799243940  0.2469983615307009  0.0548601375779311
0.1256360415028070  0.1041798430272156  1.0069640269810809
0.0892062013300254  0.3246131757798597  0.2913672349447436
```

0.2452448668795475	0.1462868276170455	0.5651890250155804
0.1031035892461366	0.1271410681333995	0.7740981226028972
0.3280256416983945	0.1173898927747146	0.2807057714657725
0.0491484760173167	0.5687763969721253	0.1494598915605516
1.0001193496201717	0.7772992539438568	0.1268591122883271
0.2753955032701975	0.3062781280460184	0.1007915552768503
0.5557911970206765	0.0573362463587731	0.2495950300269147
0.7716660667585857	0.0108186532911853	0.1072606776484901
0.2793945019490957	0.2840655593130629	0.3328058113754260
0.8480356303637745	0.7682972204731480	0.9466155999452377
0.8788274065879824	0.9029025345623507	0.9987309613194781
0.7426572280285467	0.8617968048617947	0.4465181943649421
0.8986220705007800	0.8879925961807129	0.2303324832266216
0.6705195121920634	0.8942061847989831	0.7151784009564803
0.9415931046491870	0.4430641014995104	0.8583050659613399
0.9960340273721977	0.2280463630659897	0.8849289299909207
0.7066980960099571	0.7018411642745042	0.9113980648332933
0.4431288787155011	0.9572410202356185	0.7510475983812848
0.2274857298825039	0.9988001869920614	0.9010186962040562
0.7010952694841653	0.7279856157695075	0.6780542666205576
0.9026347889474102	0.5290297798823355	0.0927712299883543
0.4961093437827535	0.9093470330903496	0.4929770262176283
0.0802583484167934	0.4902287869909331	0.9119695053837384
0.4894459541604951	0.1075447940896774	0.5042319632328317
0.9353249625178545	0.0637197327619338	0.4883886713580792
0.9061038147756074	0.0946562045929771	0.3248939166031040
0.1102881600742081	0.9185613782975658	0.5358127447061023
0.1222122020574793	0.8835299023488710	0.7145255844553573
0.4794446138607729	0.5250326818355864	0.9418727740335318
0.3184389862564214	0.6860749925151067	0.9113749832925869
0.5297743536276582	0.4534123093227367	0.1125165018203219
0.7079039545974558	0.2960541733679005	0.1248578269290514
0.7578040662502257	0.2728065396887666	0.2729736686347993
0.2724894460467392	0.7058009845746575	0.7609621494302393
0.1874094912891333	0.1868014488742147	0.9997525185066927
0.0237659351389923	0.1460901295268611	0.1948167750259925
0.2424775733019984	0.2903888046667852	0.0414286865307801
0.0936932388576738	0.3573788485329458	0.9847472653055034
0.7980495770425712	-0.0018683497179945	0.6103503919959911
0.1813278580208232	0.1906689172414691	0.6360839518757824
0.1864057561056638	0.0029886118682950	0.6644642253588057
0.2212132084795843	0.2163314733175660	0.4682864994251933
0.3887878237642696	0.1741522041047676	0.4970252434251977
0.0434980172099502	0.7893705716623035	0.6062434740734470
0.9980813378542401	0.6401113411404340	0.1926768294535466
0.1882821462486164	0.6403371268168371	0.0320800436242685
0.0355642183702847	0.4360892979542565	0.2580339619642251
0.9744100956002678	0.5636925300898407	0.1141640360948759
0.5992246460747476	0.5980497732876381	0.8052478876743838
0.6336298703505068	0.0058999459018757	0.1868263768412387
0.6474132395640401	0.1628135481122878	0.1957120645761261
0.4631586952492205	0.1067998713803417	0.2190037390468728
0.4819809042523005	0.9553707715887910	0.3944792032554726
0.5935443278958025	0.5832963170550143	0.0460491671947866
0.8159310852799944	0.8321204092066303	0.9963954721508057
0.9803305961449644	0.8432036092437052	0.8175423831670218
0.7508930187424276	0.7676697493154389	0.9303376616167310
0.8433528409820177	0.6317216322946113	0.0417893233245004
0.2336604778755858	0.9840530374521064	0.4006640401628532
0.8227265395268348	0.8287905985172699	0.3662342503690541
0.8157526818134334	0.9834340369059088	0.3862925151802941

0.7093858296864138	0.7508404398318491	0.5771138107731191
0.6240526601647587	0.8835103835396134	0.4543236257313427
0.9745241479603564	0.2048758159828355	0.4130752345910115
0.0034564458371016	0.3643762656312993	0.8262598154561870
0.8075805384946658	0.3686844068462465	0.9831133983348448
0.9295765444819317	0.5525143510987016	0.7491378734820080
0.0239924091302865	0.5056517472290050	0.8520233845419065
0.3961720181116865	0.3965317842608079	0.2377027475519606
0.3617415756288549	0.9964004355679981	0.8249912944353698
0.3728942510283304	0.8274704751705612	0.8223408837558545
0.5653987994244101	0.9570615830411686	0.7356190021689488
0.4698037438336986	0.0506038257984366	0.6225270149860334
0.3965281495552536	0.4187163779178141	0.9891188360697591
0.9932359967239586	0.5125802920681396	0.9997580582311323
0.4847751796873866	0.0071187562875223	0.5020864079305423
0.7957433308394438	0.4039498616827867	0.2194640918763224
0.0563154512329108	0.1755387573202751	0.9501574831226349
0.3904561817358726	0.7853594119921994	0.6223422829006264
0.1807851830120340	0.0635903579887334	0.8218772647923680
0.2115573811563940	0.5885019478516815	0.8050694315281820
0.9420841818616504	0.8207923690594076	0.0601575326053612
0.6182431680892839	0.2089542361708637	0.3883058140801249
0.8224258651188943	0.9576633619676586	0.1814495975466073
0.0261348318812655	0.9816933816318677	0.1459753877468729
0.9464900122763613	0.0517130456026259	0.4048814096154406
0.8409209910516446	0.1973864100039783	0.3098307479545289
0.9793455310223220	0.0269105604542092	0.8611526028726799
0.1483622682023998	0.9107871761585059	0.6050318323259453
0.1771278749882596	0.7800857089930260	0.7748221484987221
0.1376212417242448	0.8635880700509719	0.0259922701570695
0.4031658600007251	0.6151016334736630	0.9481575911374452
0.3083838544513578	0.6618414409854408	0.8447715222388094
0.8558579067153197	0.1491632800731813	0.9820816229190049
0.6078111286788003	0.3632757542443497	0.1445951540788275
0.7774049063741412	0.2859460495606882	0.1717228584999066
0.2301264630026084	0.0733452496849659	0.9313055406843229
0.0697493684045034	0.2308704552511534	0.7772628405739800
0.9226020040931049	0.7836133722045494	0.2286073387515766
0.7771954329299652	0.9337877068806896	0.0767023198456526
0.0244186462451410	0.0055594030767212	0.5219164871613746
0.5180583433891942	0.4641750754824304	0.0270188576909475
0.1636156381889349	0.3306044744201886	0.1806544039140592
0.3204067249924789	0.1923245516039745	0.3138263427092415
0.8119780817033874	0.6660128435934760	0.8427250040909424
0.6569564164532148	0.8343511157058058	0.6758492020303967
0.1793904800818799	0.3315058828096117	0.3127059478797472
0.3000519681149266	0.1864112044633173	0.1862262741812151
0.8266014346894068	0.7278326961667139	0.6547329492093378
0.6551503593126736	0.7916798298763214	0.8380205763082602
0.1997405246561200	0.7035479648156877	0.3070931397216833
0.2095580083392241	0.5875389479378627	0.3666036493034124
0.0855724203504540	0.7172286464109854	0.3430627482861892
0.1085359593213181	0.4880594238546113	0.4588354080025380
-0.0159844896977763	0.6181695251342533	0.4351144198538309
0.9953861000065959	0.5032580072304510	0.4930035566368138
0.9264860708579294	0.5287618124148802	0.6847947796091238
0.6632392420427853	0.1039927878060039	0.9177190579514474
0.5507947743407423	0.1063088779885363	-0.0092071467428990
0.7260519569983741	0.1870671025147356	0.7937330240427298
0.5007634228027650	0.1912406449256532	-0.0600647401111909
0.6761096063945911	0.2722153085362965	0.7428223746980777

```
  0.5633605060675813  0.2741302870150919  0.8160155226543585
  0.3099513154288501  0.8106346041516250  0.2039822307372282
  0.8089321242379807  0.4391671941751725  0.7673150267305738
  0.3773121768374626  0.8410836089334446  0.0865827437167742
  0.4567674088968663  0.9196564734295899  0.0128481428510856
  0.4066066211964505  0.7658342604047104  0.0964032278857968
  0.3189621538663867  0.8602214076018788  0.0618255997495977
  0.7331447634551098  0.4714503736398175  0.7973510725419439
  0.8085413268714766  0.4258072159219912  0.7177714765552653
  0.7965882160325074  0.3519934470899642  0.8459040399775093
  0.3708737323929350  0.7934673656475644  0.2257584352269334
  0.2840638582840917  0.8881451108116756  0.1911502809965538
  0.2977974169112009  0.5752998268144718  0.3398260551051843
  0.0767409893700158  0.8070316102600976  0.2977198497362511
  0.1182541453313256  0.3986796821140309  0.5039075142075666
  0.8962953548034437  0.6312278741074185  0.4608854399597265
  0.7012596498178760  0.0367664277714918 -0.0421409449189473
  0.5022167071452414  1.0417993957827552  0.0873988048898854
  0.8136556399075640  0.1848557234016955  0.7369457321933646
  0.4126239881852979  0.1925330193606866 -0.0032667944644920
  0.7245751146863257  0.3364462270547776  0.6461989666158325
  0.5238634164823356  0.3399136621542130  0.7766425780883666
  1.0044987648384240  0.4989541578789818  0.6547889809194263
  0.9391637990622448  0.6160931347290527  0.6067774686730705
  0.9163982123918647  0.4258040456381029  0.5646161084504897
```

IWV-Al-T3-TS3-Alkylation

```
   Al      Si      O      C      H
   1.00000000000000
    11.6056394577026367    0.0835597589612007    8.7649669647216797
    -3.6825602054595947   11.3529796600341797   10.3282289505004883
    -0.2346490621566772    0.2412826269865036   19.0520343780517578
   Al    Si    O     C     H
     1    37    76    16    21
Direct
  0.8940288949851909  0.6921110941880483  0.7095941884475349
  0.1348100639225707  0.2451905369615349  0.0560416947641730
  0.1199567389502346  0.1065140257453691  0.0046142317401087
  0.0923662879444328  0.3301087313391282  0.2816481872565242
  0.2422283419741525  0.1483416598361769  0.5609772832900847
  0.1006757486123277  0.1270139158845866  0.7719861333067894
  0.3238588262196676  0.1154887273265677  0.2810571681736696
  0.0452995185230145  0.5721646442249728  0.1436348313469873
  0.9942287156370938  0.7778465675632467  0.1263642461441883
  0.2756370836840548  0.3045664187758060  0.0978995687686463
  0.5515558830906154  0.0606213875423148  0.2453711024056449
  0.7676381740663685  0.0126712349544083  0.1037438481382122
  0.2801549344781145  0.2861556496629045  0.3273438299252879
  0.8425026729277429  0.7682085860067751  0.9462472729808074
  0.8725674898914935  0.9045544092264500  0.9973152293720435
  0.7370641582743088  0.8662008417590377  0.4433078703457997
  0.8932002809946941  0.8879558515218340  0.2294020300886030
  0.6714971198398195  0.9051246355033210  0.7082512471414794
  0.9431067957473526  0.4492536480669093  0.8513444262305225
  0.9927704407860671  0.2324878250449787  0.8800513376588062
  0.6991365447257397  0.7122435712700076  0.9053441073310813
  0.4411711224301508  0.9577473565166168  0.7510218729903372
  0.2236547361068016  0.9988933760921136  0.8998685504189231
  0.6967385483022185  0.7310718123058033  0.6754021612949827
```

0.8949195174765827	0.5275243091371798	0.0920015800076249
0.4912850434642025	0.9126478712891857	0.4886223094563835
0.0790792799887459	0.4894215003967091	0.9115886360467009
0.4818702889408058	0.1052498540136131	0.5070080301648019
0.9371894556291517	0.0679997249108106	0.4838372434186722
0.9034985033528973	0.0943955046848261	0.3255669795761692
0.1058815592780473	0.9188403141977421	0.5341000996313519
0.1177090024613879	0.8819602171624045	0.7143132132550838
0.4752758567138963	0.5209079379057313	0.9436424293346753
0.3172547269477160	0.6845426960866184	0.9130552000374350
0.5322971162369328	0.4576345221377039	0.1058760929136414
0.7077045077800791	0.2966196673803097	0.1241861015568012
0.7507376297852604	0.2674651530970322	0.2805603222663632
0.2719948095151239	0.7092363456475784	0.7588916478195117
0.1815663781351319	0.1866782261721586	0.9995839268744215
0.0234766994236139	0.1440299097984803	0.1970566872054511
0.2449724506388590	0.2873289007457810	0.0382585827447174
0.0899494858012778	0.3564232785916346	0.9908106919675289
0.8020251751566820	0.0028992421724710	0.6087153326551954
0.1770720445639114	0.1901817824909000	0.6342688255214952
0.1829771026922373	0.0055757536215317	0.6575809642046360
0.2197381668428154	0.2215429301446158	0.4625109181710958
0.3860085748138401	0.1769111600509235	0.4928270289500402
0.0340886442856003	0.7966613060437702	0.5976198872139346
0.9911497845456033	0.6407578866850686	0.1900651847781175
0.1820684378397531	0.6505107062234144	0.0226357084699439
0.0400753757642366	0.4424886149242345	0.2485747601629185
0.9690495782195625	0.5612455376115042	0.1124715839969039
0.5883147576897777	0.6056605612670742	0.8031468678116622
0.6305931494553462	0.0091662288113125	0.1830681168937080
0.6394493850220365	0.1681681674351840	0.1916767060281345
0.4596424476042595	0.1083161585436880	0.2136231438500137
0.4767133570892362	0.9583164632309723	0.3905520680205634
0.5875985293969717	0.5907459018858934	0.0393787675417071
0.8106455269348773	0.8321710194101309	0.9958567889927341
0.9764182627644514	0.8416999563878554	0.8190064704521350
0.7456136066720627	0.7716132872323540	0.9282944816551100
0.8346054352949528	0.6305560303973492	0.0431349243966795
0.2331887564126655	0.9810572720020375	0.4017230273675910
0.8176039235339070	0.8304877612593725	0.3650161803064315
0.8120002264998835	0.9866022658400394	0.3832779077602378
0.6985295780409964	0.7556616261775649	0.5763126048085332
0.6206996440373030	0.8893128391152779	0.4473478220167864
0.9768092011056981	0.2111232575701617	0.4023870255587503
0.9982935501302095	0.3691064982513081	0.8176777669718354
0.8168260284338944	0.3642107042686180	0.9863790445800038
0.9228503080043288	0.5556169140069702	0.7554685056575750
0.0365779298051733	0.5019284439206201	0.8440423243335431
0.3980456833700543	0.3976221601428565	0.2319836260366572
0.3585423756069366	0.9950736747998896	0.8267541328927379
0.3855163775571882	0.8191788063033896	0.8314779780333430
0.5736159615651123	0.9784191046145976	0.7129966234951405
0.4453956378243665	0.0372361225120477	0.6352862163584742
0.4024344830619342	0.4107328933902170	0.9870121739198199
0.9828686326323811	0.5129164806115337	0.9972302802450459
0.4816313239505964	0.0113266136719283	0.4961901106942594
0.7884778412767266	0.4023422588751969	0.2201884974741243
0.0546403118672888	0.1816847609655578	0.9439219027918222
0.3861138814415993	0.7883618118238497	0.6184639149609142
0.1807910843087998	0.0660507155563691	0.8171308826123935
0.2097804162890585	0.5869440355212110	0.8112213802762132

0.9305115468958776	0.8220854543332006	0.0646652189827656
0.6136052145569667	0.2012955323618893	0.4022336282522861
0.8164438372811385	0.9523685241497142	0.1805114564286673
0.0182861708574257	0.9854047418592164	0.1435413210624843
0.9409506269007009	0.0471667300538521	0.4088532530562702
0.8401702028614350	0.1981825338819784	0.3107713586501417
0.9773626016967729	0.0257203342985298	0.8596185287095368
0.1398066609918862	0.9006964216324307	0.6105954035505438
0.1752500860351387	0.7820304315145272	0.7743263275535203
0.1317838737941867	0.8642849804144501	0.0228889871302260
0.3842943628596204	0.5954261990831677	0.9669067891975625
0.3171981945650133	0.6740532970819685	0.8334998827542418
0.8531755128231100	0.1503691757454386	0.9800506268326105
0.6158585802127909	0.3742540264631920	0.1343707259293526
0.7632301357288506	0.2714952749829933	0.1875144446375323
0.2237430282852686	0.0728660632203600	0.9315267032133825
0.0654926901190778	0.2307601733570608	0.7749705223568157
0.9213059738494828	0.7817651752708614	0.2299195161481867
0.7702983513037944	0.9405239439443658	0.0694070154512457
0.0308800168996602	0.0165627486613256	0.5122150637122358
0.5218825359319175	0.4638319108768730	0.0218298092101052
0.1672076201423842	0.3381529934016693	0.1696227361654952
0.3196361271031242	0.1916827081787268	0.3120486970128649
0.8028910882795814	0.6837243996965007	0.8292379966911041
0.6634285312673593	0.8402065666141083	0.6710182852851697
0.1822589082381610	0.3354838122776988	0.3040656865184214
0.2900451231829432	0.1816610033816552	0.1914147151370350
0.8225280564336659	0.7197680961588668	0.6507373220135735
0.6422121103792724	0.8056817190162692	0.8386616649029185
0.3654131558757505	0.8288676726806544	0.1231050832661125
0.3672497031879174	0.7327034974971856	0.1320791838877743
0.2540635185670600	0.8428580110487206	0.1830963345450943
0.2611219551521206	0.6535357961963300	0.1979802507130604
0.1478126089207680	0.7635450945561951	0.2496570273688550
0.1505956671343716	0.6689721140135273	0.2567822294399639
0.9439516756611541	0.5355759960050889	0.6351574092970510
0.7722976617678610	0.2660080332400558	0.7927738516345325
0.6767281038983178	0.3119326511414106	0.8244481892295066
0.8776723125346951	0.3387399520735263	0.6753260141947244
0.6851926513616519	0.4296466878835323	0.7386236427620405
0.8890715079287653	0.4595615002965074	0.5887052975672273
0.7906645617499111	0.5033922142727321	0.6201066779572224
0.4810853507126197	0.9165946474841218	0.0474600461373823
0.0746473260775407	0.5664066748104923	0.5517428600464560
0.5480888109517349	0.9980847476261897	0.9126594749448230
0.6315185520049754	0.0620248017817787	0.8590941676448833
0.5714970191500924	0.9451424437584625	0.8892825799595799
0.4922282109746792	0.0484975174388494	0.8870081276966912
0.1123115775113008	0.6200417069316817	0.5521055005318574
0.1069869151232637	0.4884429689277314	0.5776506397986760
0.1078328323665168	0.6269379254860877	0.4585826844364211
0.5396142615250028	0.8677552634981608	0.0699249465438203
0.4615420640019878	0.9709029398543659	0.0691005408288468
0.4530145921811042	0.7198994820232825	0.0866029602457882
0.2511799660030946	0.9173064354117555	0.1768286689530945
0.2647512529874302	0.5798014579124030	0.2032562777323113
0.0619159260774483	0.7772284086844784	0.2941681219084553
0.7636096210672660	0.1735184804420343	0.8607152567622061
0.5948274918048149	0.2547201818628484	0.9171895368620447
0.9526048082650558	0.3043229812629304	0.6500192250406946
0.6106266048820381	0.4643573516163604	0.7638324998571349

```
  0.9696377603032005  0.5155741801025524  0.4954715836154500
  0.7982643352758342  0.5955149658743475  0.5522561922710056
  0.9018377926901360  0.6063326178705769  0.6143964461439235
  0.8885409586908145  0.4477044025811123  0.7246820813115413
  0.0679452468405510  0.6091370366130414  0.3053894912107404
```

IWV-Al-T3-EBH+EB

```
   Al     Si     O     C     H
   1.00000000000000
     11.6056394577026367    0.0835597589612007    8.7649669647216797
     -3.6825602054595947   11.3529796600341797   10.3282289505004883
     -0.2346490621566772    0.241282269865036   19.0520343780517578
   Al   Si   O    C    H
     1    37    76    16    21
Direct
  0.8723101262822925  0.6779549268937984  0.7326539686343382
  0.1232404946545827  0.2411585622243377  0.0655189885879005
  0.1044175021023749  0.0986252622720365  1.0184774872530593
  0.0851471924897800  0.3231887094287070  0.2888301805513060
  0.2296279389495746  0.1398646313130385  0.5738033375126819
  0.0884430565151399  0.1229696803306856  0.7816456557768288
  0.3065682948509020  0.1001644762058131  0.2947822666524483
  0.0288601641401533  0.5603377653879853  0.1563007162224783
  0.9838355704511470  0.7699527136348461  0.1361843521358246
  0.2640595168634750  0.2966179827923028  0.1074220665540721
  0.5387992030837857  0.0583481662730129  0.2477239677019357
  0.7568568356871402  0.0057588767018223  0.1142665858879207
  0.2739151297777117  0.2712403181998924  0.3372766002003854
  0.8288956251743065  0.7616334410501735  0.9581354658098963
  0.8633239185949847  0.8979706844692565  1.0070709820253774
  0.7246874430499933  0.8623284193018851  0.4514451424556978
  0.8802523778651774  0.8792947558028312  0.2416383882036017
  0.6575395111783778  0.9035917666924492  0.7198625809035061
  0.9339831389268375  0.4428477896050503  0.8665097771659128
  0.9793770529565845  0.2273515915859930  0.8914904669222841
  0.6806975505491027  0.7081149049071599  0.9207267703553288
  0.4238918796666078  0.9461276823636369  0.7706053818084387
  0.2099827518711041  0.9944059887918032  0.9108331804010811
  0.6796023320009720  0.7292450914673423  0.6881690997024006
  0.8778100130667146  0.5153806433064219  0.1056453586500621
  0.4811037266041922  0.9121970719876676  0.4937200817324401
  0.0725304733208176  0.4874191045762316  0.9182459182348901
  0.4663253391524952  0.0924869880746901  0.5270518905890937
  0.9206540670125622  0.0634199536353685  0.4943909744371391
  0.8882915811276516  0.0916026702189483  0.3336684918829249
  0.0867840969812906  0.9088586981371924  0.5485779842140572
  0.1020650210211141  0.8770321848549569  0.7242429274596560
  0.4565295258594451  0.5163547809434742  0.9544755164574571
  0.2996667839010742  0.6802602309167453  0.9235116348241005
  0.5230993583085731  0.4559928478924771  0.1107948843780845
  0.6936861704829952  0.2890606666680400  0.1346152811130584
  0.7238733230815019  0.2528613505221252  0.3009640519856078
  0.2628407891143498  0.7112018860130677  0.7624067795780679
  0.1658607158883453  0.1797954855030144  1.0123197523476579
  0.0144317596539686  0.1424601846765408  0.2075937356533148
  0.2373215759486817  0.2830569427210240  0.0434700177267422
  0.0789686365488276  0.3524458188228454  0.9999242703301656
  0.7870034488887078  0.0034233222191401  0.6198870179841469
  0.1629254695374063  0.1851746924176866  0.6442960084388325
```

0.1670344963268908	-0.0024707294583858	0.6726335877773739
0.2125373251860040	0.2103232135410161	0.4718754842575190
0.3719933331828816	0.1660939297617543	0.5111947332926161
0.0129088931168210	0.7832100282270956	0.6204293116450954
0.9809489580060111	0.6334936821774854	0.1980129137690722
0.1573242453927591	0.6439002258951286	0.0236229759527691
0.0402989177639355	0.4397404615438006	0.2540941472270639
0.9372022606668499	0.5283735868162212	0.1494210943199322
0.5633456845033644	0.6089240180047452	0.8121622588330953
0.6184985208903316	-0.0005170524066815	0.1937885230790846
0.6246332881040700	0.1822421690119522	0.1720547829733186
0.4393902968902742	0.0875747178350972	0.2257206887041714
0.4718257036478212	0.9675289672390990	0.3899647517543676
0.5665051930047278	0.5876156616899758	0.0539032207373730
0.7992160369562078	0.8281754911362379	1.0051296376950023
0.9600061010249953	0.8373362798243256	0.8277724953259704
0.7259318196584551	0.7639544611198251	0.9473510760222337
0.8264022828710583	0.6265749698648082	0.0529850854102786
0.2127032557173307	0.9676482046883597	0.4162035611142564
0.8023578915783299	0.8210931191827175	0.3777014591981254
0.8014856385205233	0.9879517333468776	0.3819499477548015
0.6887775769654855	0.7591450457367188	0.5833840112842953
0.6064082352703388	0.8807969895111403	0.4582000199140994
0.9659824542244685	0.2072253842303168	0.4090272191368541
-0.0154083470787124	0.3630006123431642	0.8310205630485984
0.8271399082876510	0.3484914465843807	1.0116221371794845
0.8889884613457057	0.5364411699923007	0.7921286595967350
0.0388177152421100	0.5048216479173607	0.8426840811120841
0.3922147428633261	0.3812516244240062	0.2432875767703595
0.3459645203724009	0.9942434963614641	0.8362932279796471
0.3653304532350532	0.8036676721979330	0.8658216732483488
0.5581990841708249	0.9754738053559902	0.7254732279184894
0.4203988597980651	0.0104246443211941	0.6618107507796903
0.3930491944942238	0.3991946116198928	0.9998750769157747
0.9732123682726663	0.5090079747452481	1.0053368534794833
0.4745708716081594	0.0082738437117597	0.5035853832077941
0.7640311161367624	0.3942934568980152	0.2226564230369780
0.0380195783993006	0.1713529071394782	0.9588187115725819
0.3694793221922683	0.7908760381788924	0.6199436081873462
0.1677249612502101	0.0618685710790320	0.8276743918075066
0.2036748890643549	0.5809956586640856	0.8252498302299045
0.9224863999257229	0.8149046292979796	0.0721795885781552
0.5963142061191260	0.1877328989311574	0.4327422214934414
0.8059235097226882	0.9478055539362724	0.1901611172237776
0.0056411718468220	0.9755132798476968	0.1573529467878613
0.9153667953876771	0.0397175141681206	0.4252041728763128
0.8259918231184246	0.1968158154799208	0.3151933394897637
0.9662459712799194	0.0201387353365301	0.8694701866688129
0.1259530434079775	0.9019548692869528	0.6169811324187739
0.1581532394182043	0.7750373983853421	0.7841386920913505
0.1207312135077563	0.8581479961956535	0.0334373226583354
0.3538965725465689	0.5787741941386430	0.9868687001270757
0.3203305381826078	0.6950573306830831	0.8213875824875732
0.8396144518794741	0.1443334976763523	0.9899098945269191
0.6193246215827117	0.3845215768880603	0.1228659807629899
0.7057275935301368	0.2302136707195237	0.2375362225214760
0.2092933393936049	0.0664494429889278	0.9443909570719240
0.0514550427846887	0.2254688387472544	0.7862427236804901
0.9080422932942269	0.7749110967993623	0.2392789407635375
0.7605880330928491	0.9319614460912400	0.0810940634678847
0.0140698996978142	0.0085182803107430	0.5221046211423601

```
  0.5097684491302775  0.4677693197918719  0.0256740740201034
  0.1553876756458451  0.3318874479271491  0.1768632234411112
  0.3127012406962659  0.1753115774155714  0.3236505752747445
  0.7850426873961853  0.6788397501273514  0.8466531887526791
  0.6489581123880734  0.8398747340339905  0.6820103884628081
  0.1764196113463291  0.3194406292078409  0.3133921060478219
  0.2720439577105129  0.1711074541962735  0.2051974751896591
  0.7988553076393644  0.7091243957053600  0.6747471605902848
  0.6280665131826968  0.8051537998378040  0.8504039373219833
  0.2446983773967376  0.7067736695777589  0.2500370385069682
  0.2625266530508360  0.5960713094222391  0.2899015373075974
  0.1286614151845302  0.7162314655786486  0.2904303514415105
  0.1670449307385060  0.4974130545154368  0.3678358242630337
  0.0329392699068181  0.6179368266489610  0.3677717597242856
  1.0521197905063506  0.5083171343748948  0.4061658080460895
  0.7766697479783264  0.4076648616676142  0.6918849802839860
  0.6085223698248127  0.1249061778039085 -0.0181383341480622
  0.6610571075693117  0.0659848041379672 -0.0583639585360262
  0.6719388165436418  0.2380026490490237  0.9123999381737989
  0.7776858510900977  0.1179676978376199  0.8337569861637146
  0.7898155868067284  0.3000123293379408  0.7957560670256134
  0.8430407208527493  0.2300009565325626  0.7648295465552888
  0.3492883728935285  0.8142975879371572  0.1615172268096314
  0.7272042419303966  0.4939042467999686  0.7062864542542187
  0.4132118255794764  0.8770696609260374  0.0317441224302525
  0.4893318085858915  0.9557510509925256 -0.0329719952189623
  0.4475966089963377  0.8165185943708494  0.0179528525113922
  0.3514811752601152  0.9078801892629631  0.0098133255353035
  0.7342311209700532  0.5714062530067031  0.6264951075314739
  0.6324140582136807  0.4547402537596065  0.7846846313448432
  0.7786697029392348  0.5270804375180003  0.7126387126675684
  0.4130855353066292  0.7858179803312429  0.1812789521236892
  0.3170764226151302  0.8771058746178344  0.1727489916971553
  0.3524605202225596  0.5873783013177267  0.2587651083598089
  0.1133995249281125  0.8018100075377164  0.2604113845357623
  0.1822602291080314  0.4119537670355368  0.3985176273429167
  0.9438134554707711  0.6276502733093352  0.3965160899588051
  0.5198340298636335  0.0805977627205855  0.0677834306736498
  0.6103255694329083  0.9765734863424074 -0.0036936708946700
  0.6330830285658332  0.2855927884675316  0.9413701834498546
  0.8156947648355756  0.0679327192968336  0.8077565235601520
  0.8522631740190946  0.3509804354459518  0.7798039508776473
  0.9340533635410851  0.2726417012720184  0.6811599569497060
  0.8677652709717683  0.4516223702047585  0.6071130797049311
  0.7196272223272974  0.3657708301221395  0.6963482198242659
  0.9782387341968003  0.4322780033601141  0.4638356540945303
```

IWV-Al-T3-TS4-Alkylation

```
   Al     Si     O     C     H
   1.00000000000000
    11.6056394577026367    0.0835597589612007    8.7649669647216797
    -3.6825602054595947   11.3529796600341797   10.3282289505004883
    -0.2346490621566772    0.2418282698650 36   19.0520343780517578
   Al   Si   O    C    H
     1    37   76    16    21
Selective dynamics
Direct
  0.8973472118377684  0.6402654647827148  0.7314072251319885
  0.1416859626770020  0.2002434432506562  0.0716904103755951
```

0.1401277035474777	0.0713253840804100	0.0056203939020634
0.1013407260179520	0.2859876751899719	0.2952344119548798
0.2516914606094360	0.0971733927726746	0.5757415890693665
0.1151027083396911	0.0889801383018494	0.7773003578186036
0.3306788206100449	0.0683799088001251	0.2931998670101169
0.0441381782293321	0.5241798758506776	0.1606364697217944
0.0138094425201416	0.7388572692871096	0.1313446909189223
0.2831610143184662	0.2639721333980561	0.1089510768651962
0.5595510005950928	0.0090618282556534	0.2586070895195007
0.7844077348709105	0.9672765135765076	0.1129268482327466
0.2901948094367982	0.2358324378728867	0.3419593870639801
0.8520598411560059	0.7178934216499329	0.9614893198013308
0.8928415179252623	0.8651303648948669	0.0002681201731322
0.7521990537643433	0.8221281766891479	0.4530174732208252
0.9048348665237425	0.8468462228775009	0.2377072721719753
0.6791030168533324	0.8608964681625368	0.7238033413887024
0.9419084787368773	0.3964027762413025	0.8715902566909791
0.0182347893714907	0.1944559216499336	0.8814989328384404
0.7073320746421814	0.6626943349838257	0.9235532879829407
0.4460540711879730	0.9093395471572878	0.7637462615966797
0.2376676201820374	0.9597314000129700	0.9078991413116461
0.7016822099685667	0.6915117502212524	0.6897812485694885
0.8865787982940674	0.4725931882858276	0.1222082599997520
0.5006870031356812	0.8493720293045046	0.5031918883323672
0.0722661018371585	0.4369611740112306	0.9400401711463930
0.4905891716480255	0.0423471927642822	0.5199007391929626
0.9378236532211304	0.0278934240341187	0.5011253952980042
0.9135211110115051	0.0519219189882278	0.3367167711257934
0.1072251200675971	0.8760451674461367	0.5482780933380128
0.1272953450679779	0.8350573778152465	0.7252441048622132
0.4703970551490783	0.4867011308670042	0.9590265750885012
0.3106343746185303	0.6401812434196473	0.9251244664192199
0.5384842753410339	0.4141553044319153	0.1191475540399551
0.7113463878631590	0.2472341060638428	0.1348461061716080
0.7587941884994505	0.2187597304582596	0.2858001291751862
0.2823275923728943	0.6577225923538208	0.7671966552734376
0.1925247907638550	0.1472657322883613	0.0099627608433364
0.0403993278741837	0.0923842936754227	0.2153066843748093
0.2546446621417993	0.2509869337081909	0.0455968417227272
0.0838435888290405	0.3037534952163696	0.0186885595321655
0.8029482960700987	0.9670448303222658	0.6257671713829041
0.2005535364151001	0.1479957401752472	0.6402339935302734
0.1787604391574870	0.9562525153160139	0.6812619566917392
0.2283042669296264	0.1696518659591675	0.4779468178749084
0.3948391973972319	0.1154226660728455	0.5085005164146424
0.0338506698608399	0.7506936192512514	0.6183711290359504
0.0078989267349243	0.6010371446609497	0.1973004639148712
0.1677085608243942	0.6026772260665894	0.0244700163602829
0.0593788623809814	0.4044704437255859	0.2550637423992157
0.9403538107872007	0.4930131435394287	0.1655994504690168
0.5789511799812317	0.5795757770538330	0.8167693614959719
0.6511688828468323	0.9804767966270447	0.1755492836236954
0.6312379837036132	0.1143051683902741	0.2213712632656098
0.4625540375709534	0.0507863759994507	0.2332774847745895
0.4931212663650513	0.8945369124412537	0.4011256992816929
0.5934473276138305	0.5439417362213135	0.0589408315718166
0.8320218920707700	0.7889809608459517	0.0016596267232651
0.9864143133163450	0.7767670750617981	0.8380705714225769
0.7541681528091428	0.7265996336936948	0.9428223371505740
0.8320712447166440	0.5787665843963623	0.0671926364302635
0.2302273511886611	0.9391187429428116	0.4131578803062431

0.8400980234146118	0.7912256121635437	0.3710550367832184
0.8211767077445985	0.9461117982864382	0.3924575150012971
0.7142714262008666	0.7143980264663693	0.5862736105918885
0.6333542466163634	0.8354685306549072	0.4612109363079071
0.9814949035644529	0.1712037026882172	0.4177958667278286
0.0123766660690313	0.3271763920783997	0.8272849321365358
0.8119855523109436	0.2996255755424500	0.0009049177169800
0.9224086738481376	0.5008175927363903	0.7707825697176500
0.0147425532341003	0.4436570405960083	0.8853233456611633
0.4075741171836852	0.3460235297679902	0.2498977482318878
0.3715204596519477	0.9647983312606854	0.8219163417816147
0.3713134527206421	0.7730712294578553	0.8539440035820022
0.5704293251037597	0.9195815920829772	0.7421693205833436
0.4677637219429011	0.9828339517116549	0.6402315497398378
0.4054933190345763	0.3754252791404723	0.9980058670043946
0.9892424941062926	0.4652026891708375	0.0238960981369005
0.4769530892372130	0.9426054954528811	0.5199005603790283
0.7762745618820187	0.3486580848693848	0.2411704063415527
0.1029446721076965	0.1616351008415222	0.9193184971809393
0.3973684310913085	0.7215462327003478	0.6302041411399842
0.1844728589057922	0.0294824242591858	0.8344212770462036
0.2054693102836609	0.5340397953987122	0.8321779966354370
0.9505301713943480	0.7856794595718382	0.0687993019819257
0.6240466833114625	0.1352381408214569	0.4076747894287110
0.8095827102661135	0.8883549571037314	0.2099081128835666
0.0207515656948090	0.9611386060714722	0.1390198916196824
0.9380257129669189	0.0062612593173981	0.4281516373157502
0.8570762872695921	0.1624569594860077	0.3069649636745452
0.9955697655677793	0.9857674837112429	0.8612396121025085
0.1501462459564209	0.8651019334793092	0.6158887743949889
0.1980884075164795	0.7431737184524536	0.7699023485183717
0.1531732231378568	0.8214565515518186	0.0275724399834876
0.3695348501205444	0.5509950518608093	0.9904254078865051
0.3327472805976868	0.6317661404609680	0.8354319334030152
0.8841044902801511	0.0986200422048568	0.9958730521611872
0.6265777945518494	0.3323133587837220	0.1365272849798203
0.7811625599861146	0.2350256741046906	0.1822885274887085
0.2411595582962036	0.0241261720657349	0.9471295475959778
0.0750881135463715	0.1946141719818115	0.7769201993942261
0.9458923935890196	0.7452801465988159	0.2327527254819871
0.7904633283615086	0.9028837084770202	0.0704927369952206
0.0298222303390505	0.9717140197753908	0.5306993126869199
0.5208359956741333	0.4314458668231964	0.0335283130407334
0.1694289743900299	0.2868421375751496	0.1885584592819214
0.3313291072845458	0.1433413475751877	0.3239538073539734
0.8098269701004028	0.6273965835571289	0.8541875481605532
0.6813554763793945	0.8089711070060730	0.6718920469284057
0.1938894242048264	0.2865250408649445	0.3167181313037872
0.3078848123550415	0.1444582343101502	0.1941746175289154
0.8178536295890806	0.6638196706771849	0.6812373995780947
0.6548086404800413	0.7554342746734601	0.8512443304061916
0.4435339868068695	0.8722147345542908	0.1101893112063410
0.3474522829055785	0.7785391211509705	0.1795518249273300
0.4185616374015811	0.9740189909935000	0.0907753258943560
0.2303587347269059	0.7859122753143313	0.2287800461053848
0.3021896481513977	0.9810317754745483	0.1409711539745333
0.2068464607000347	0.8868029117584231	0.2108440399169922
0.0110001564025879	0.4660449326038362	0.5692768096923828
0.6790763735771179	0.4331002235412599	0.7577456831932069
0.6356129646301270	0.3151761889457703	0.8666355609893799
0.8013541698455811	0.4881794154644012	0.6682643890380859

```
  0.7132229208946227  0.2488338947296143  0.8853105306625368
  0.8854623233245469  0.4255273280533302  0.6841636137108795
  0.8351492881774902  0.3007206916809082  0.7935193777084352
  0.5703041553497316  0.8659546971321106  0.0592485330998898
  0.1175645589828494  0.5243031382560739  0.5364932417869568
  0.6571770906448364  0.9531038403511048  0.9217000603675842
  0.7505345344543458  0.9609425067901612  0.8886538147926328
  0.6422345042228710  0.9224107265472454  0.8907361030578586
  0.6458330750465393  0.0424373149871826  0.8804875016212463
  0.2006355524063112  0.5475814938545257  0.4551379680633535
  0.1099770069122323  0.6067882776260376  0.5181795954704285
  0.1247358322143555  0.4635058343410492  0.6090297102928162
  0.5711811780929565  0.7744514346122742  0.0984369069337847
  0.6033817529678345  0.8864089846611023  0.0870966166257858
  0.3646523356437680  0.6982108354568486  0.1962158828973780
  0.4921180009841918  0.0485112667083741  0.0363427847623822
  0.1569240987300872  0.7123278379440309  0.2819099128246310
  0.2860762476921081  0.0601973533630371  0.1259465515613556
  0.6169266104698181  0.4812574386596680  0.7444058060646057
  0.5390771031379700  0.2742107510566711  0.9379684329032901
  0.8363997936248779  0.5809153318405152  0.5846282243728638
  0.6767421960830688  0.1564548015594482  0.9699211120605469
  0.9004249469097731  0.4639477765152256  0.7286295779068783
  0.8968136310577393  0.2489309906959534  0.8017129302024841
  0.0234177410602572  0.3864676356315612  0.5824528336524969
  0.0064586699008942  0.5273652076721191  0.4947276711463928
  0.1158299297094343  0.8928309082984927  0.2504207789897929
```

IWV-Al-T3-2EB

```
   Al     Si     O     C     H
   1.00000000000000
    11.6056394577026367    0.0835597589612007    8.7649669647216797
    -3.6825602054595947   11.3529796600341797   10.3282289505004883
    -0.2346490621566772    0.2412826269865036   19.0520343780517578
   Al   Si   O    C    H
     1    37    76    16    21
Direct
  0.8867667095273424  0.6947585312585682  0.7169936275738615
  0.1284534911498250  0.2429846416340322  0.0607519505195624
  0.1145449383989597  0.1033485119197445  1.0101781251644684
  0.0830285167489096  0.3247665920250137  0.2914084122947115
  0.2359366775210218  0.1440968412840152  0.5685354169192904
  0.0936351363210849  0.1223869269617093  0.7788402540918433
  0.3173975459422892  0.1119688131439940  0.2868448518017841
  0.0388000422867545  0.5686913028800291  0.1521470760276667
  0.9879417561037029  0.7740854107096433  0.1333636845524380
  0.2675587006180430  0.3010079024607460  0.1053868990906066
  0.5440993236152073  0.0536091359540640  0.2556308311571257
  0.7608645331101992  0.0083112048933749  0.1119495778625500
  0.2711028596787507  0.2809460235522639  0.3359939804258940
  0.8375348467488709  0.7656095672780920  0.9521811800665837
  0.8675013567501652  0.9001136440845980  1.0043079659156238
  0.7323759355388446  0.8625020784837908  0.4495703092388450
  0.8874341758730160  0.8858182065820231  0.2351532303926480
  0.6642364512437715  0.8972681930717955  0.7174720624712387
  0.9334811858322010  0.4367912393463469  0.8657115727907078
  0.9854662710165704  0.2259291960940386  0.8881913317083554
  0.6929665976767415  0.7036330268894135  0.9167432278252170
  0.4346291547578492  0.9541965185575796  0.7564098381666058
```

0.2167445769422662	0.9950294356407033	0.9062944309028255
0.6906062642465941	0.7256923614407094	0.6830155560566586
0.8910536057861030	0.5255871806890902	0.0979225399831201
0.4850488623194543	0.9073013365313678	0.4976548759195641
0.0715700603502157	0.4860610238337018	0.9180656629300477
0.4773831392232474	0.1039062867949668	0.5109968695612996
0.9286117137157067	0.0631906851689777	0.4919907559811163
0.8968689524199525	0.0922841080090015	0.3309384633157423
0.1008324875556800	0.9159848081908081	0.5410931972948859
0.1122962519065722	0.8796582625062810	0.7206839482384267
0.4681804918738612	0.5192967122908648	0.9498717973879603
0.3085887935072248	0.6812314481406767	0.9192781659546517
0.5230009525824724	0.4521942875845700	0.1155806070914959
0.6980548166732035	0.2907926809555337	0.1332474547300401
0.7468467709126290	0.2675676933631169	0.2824588508711856
0.2632101650342004	0.7031086355538481	0.7671432820966685
0.1760745336612315	0.1829752155807425	1.0057959759705815
0.0166610230078209	0.1433096894466667	0.2017682588798362
0.2364991023594598	0.2877186767932006	0.0436007428878937
0.0835883647280625	0.3530898828069438	0.9925110033446735
0.7933626111047088	-0.0026758468199026	0.6169801691215024
0.1707915042528956	0.1870059412196117	0.6405833638137771
0.1785477042132846	0.0005617880371762	0.6670252925022200
0.2116714328345384	0.2138255685375603	0.4716850094593499
0.3799424906517279	0.1738225565892812	0.4989800179414247
0.0295358638553421	0.7888660841458170	0.6113030590926511
0.9853142033321773	0.6367929138211265	0.1987670790442103
0.1763845739641303	0.6462414908802540	0.0322477981994997
0.0306748174804204	0.4368573414079787	0.2570134774018124
0.9639011286536334	0.5609916316111576	0.1180668827111379
0.5825863370493963	0.6004136289589922	0.8102161189702458
0.6233116407156529	0.0030044372648138	0.1927940546402717
0.6325101796524497	0.1599587982013154	0.2037161631059777
0.4529525350604902	0.1033405981980039	0.2230258373474588
0.4689490114145549	0.9516951730384721	0.4008067625336380
0.5813019715147826	0.5832842623126867	0.0511133368124267
0.8046193711939811	0.8275948964805026	1.0034607396155875
0.9703403851910392	0.8407341785303528	0.8232264608798708
0.7396679780206946	0.7677584709736968	0.9354953954867606
0.8318313857945094	0.6281111977770781	0.0464102070514023
0.2251425585402808	0.9778104273402376	0.4077644129334859
0.8130655081931623	0.8284199842941069	0.3703406073838702
0.8054831777941569	0.9847538241582823	0.3885259728414326
0.6968888945230546	0.7528298369409470	0.5812235480651098
0.6141712200291620	0.8838952744042636	0.4565371196650077
0.9677542717219458	0.2053989449967825	0.4129245132003037
-0.0091237385979239	0.3639389143523474	0.8257216972555197
0.8018776985274484	0.3597844365786044	0.9927410054052326
0.9144274428591789	0.5491099793685169	0.7622691030773054
0.0198849420543218	0.5003685563514343	0.8549348993842638
0.3888729935915126	0.3922968011667948	0.2412997540064599
0.3509042294732304	0.9895511235671811	0.8337104773418400
0.3746351042008397	0.8197531273232541	0.8295329780014417
0.5627496214607365	0.9656290885530547	0.7288939406750949
0.4491246672458918	0.0424022879570725	0.6342616869881685
0.3923231551363921	0.4089824317105530	0.9961711653511498
0.9802416964206986	0.5084435472231759	1.0051423146248160
0.4742862338642658	0.0060864302910685	0.5054187208429376
0.7839221962809804	0.4008264894029895	0.2253657474705016
0.0466843133262801	0.1786784666119658	0.9521278116656303
0.3802194367774442	0.7830909978401563	0.6280327844054969

0.1733116942332572	0.0594635518291734	0.8244584034329754
0.2021493162258263	0.5846751387123030	0.8128476902278192
0.9299090512107729	0.8175398590975145	0.0667883247483261
0.6083470328342495	0.2020960977201900	0.4005072832204226
0.8110505011860349	0.9547753935798828	0.1867334030054293
0.0152538247735742	0.9804696697466510	0.1486468841754681
0.9335768303390694	0.0449118256136027	0.4148700273113738
0.8337040509343452	0.1963520495505103	0.3156316147164425
0.9705063813643833	0.0216469413872742	0.8661740049617688
0.1375612158121937	0.9037376178469125	0.6130352966163195
0.1666646752491752	0.7762399059167203	0.7820685001965826
0.1254602559967418	0.8607697625906026	0.0322232705773634
0.3817827389377116	0.5988014581855734	0.9678842160131035
0.3012295615453807	0.6608150793315600	0.8489641293501142
0.8456687117003637	0.1463829957414593	0.9873357966991667
0.6045710622106258	0.3660092146848302	0.1451768112379012
0.7637137878603452	0.2759281781587686	0.1848418761521606
0.2185887797022557	0.0724766736047448	0.9341730085206116
0.0600769606478204	0.2254254622777300	0.7829016875366007
0.9105976469009451	0.7802706902780117	0.2351722447883147
0.7665971626142305	0.9316792695435194	0.0813172093456492
0.0206997468006744	0.0085510937303522	0.5221382891504973
0.5144843833157480	0.4629683963528609	0.0284095159617894
0.1575333264305202	0.3301805926155233	0.1810498924401098
0.3111205332102685	0.1882734968202847	0.3182865807689738
0.7977617606986040	0.6700959962702389	0.8453215368643326
0.6569117254752818	0.8354267338909368	0.6774332975353901
0.1727290009741302	0.3301186395412232	0.3138672484578092
0.2860579674255297	0.1783912523230110	0.1952226085251495
0.8146754841720787	0.7129730137054792	0.6633571852935896
0.6402193762625159	0.7948290982543090	0.8450659290613942
0.2239655444174492	0.6963176041328587	0.2556665418253578
0.2243021926810146	0.5778445085689951	0.3122847772320657
0.1132738987411168	0.7183768853554052	0.2995033557314712
0.1170906760361399	0.4843072156949323	0.4098232718369366
0.0055903703350112	0.6250579585299071	0.3972772688081439
1.0072848346378018	0.5077451823591499	0.4524866364777336
0.7781104133258377	0.3872382754174392	0.7737839906740276
0.7477696207437744	0.0988274786669236	0.8462978785446834
0.6313410712984597	0.0303408897905374	-0.0455771724291498
0.7963015949267392	0.2130383324323495	0.7895059756282641
0.5639303629391246	0.0767162873851926	0.0054490024507342
0.7289813553627099	0.2607572722254041	0.8393591499726484
0.6124304964935754	0.1912012976006556	0.9480740318872805
0.3401764330233135	0.7972827438994927	0.1492120144073983
0.7382400930577028	0.4751658386726564	0.6970540446956710
0.4137415310220062	0.8058297408386629	0.0437284132715717
0.4973401995379987	0.8803529233686425	-0.0327127779647666
0.4371114225782737	0.7233695087055783	0.0697767365623644
0.3623044142486053	0.8199387986565918	0.0135584446287906
0.7821134205339110	0.5680169769553467	0.6416676046340517
0.7613896376752457	0.4655716200434427	0.6367965289594664
0.6407895949157794	0.4576617818890120	0.7542318796112338
0.3944371480577726	0.7848036132471986	0.1765918603937932
0.3206200311580885	0.8819151376542562	0.1204342843254607
0.3096074874177314	0.5588287796182083	0.2791192963944847
0.1117871734078791	0.8102008859627297	0.2559001716224840
0.1191154820632854	0.3928299574997726	0.4527747709471887
0.9204415754928972	0.6442259982870691	0.4296272775493224
0.8012634199437769	0.0627912604448692	0.8065319318385248
0.5939928949344243	0.9408052364255585	-0.0007726723627682

```
  0.8877306046138002  0.2660850526065416  0.7055625046977989
  0.4735041318318343  1.0233609883064809  0.0905771063862545
  0.8927512276890023  0.5251350106709068  0.7365229018518765
  0.5592932982848237  0.2267260974153758  0.9886013825304739
  0.7456287353176713  0.3909212371023739  0.8406648141427067
  0.8771049031214530  0.4124338273510075  0.7157129422359370
  0.9234578835290130  0.4348690753092060  0.5279026376957021
```

S4.2.- IWV-Al-T6-Transalkylation

IWV-Al-T6-DEB+Benzene

```
   Al     Si     O     C     H
   1.00000000000000
    11.6056394577026367    0.0835597589612007    8.7649669647216797
    -3.6825602054595947   11.3529796600341797   10.3282289505004883
    -0.2346490621566772    0.2412826269865036   19.0520343780517578
   Al   Si   O    C    H
     1    37    76    16    21
Direct
  0.5361294633486047  0.9281124601031492  0.4726979662118402
  0.1616023532101520  0.2795126692965893  0.0327021268060922
  0.1349493489692243  0.1319738840771045  0.9894263124335757
  0.1045019406252092  0.3418572973614805  0.2794992867102691
  0.2524551977040752  0.1739577866700651  0.5492082924829943
  0.1207434251728167  0.1569451451807409  0.7529840525626001
  0.3424185981361308  0.1383021178702653  0.2711487385602019
  0.0697354571207996  0.5822837900528089  0.1340982445354880
  0.0276893714329863  0.8047305946475403  0.0964714821121318
  0.2948909657388762  0.3303649119315416  0.0843435015066434
  0.5747628271974122  0.0743067793556519  0.2279462092053894
  0.7879625418077900  0.0287795794895014  0.0867984306711466
  0.3021940824451846  0.3045838728773080  0.3120713211469155
  0.8744899034784968  0.7759861660989167  0.9339894613361186
  0.9027854946015187  0.9265689608298741  0.9738633293000176
  0.9220832743284446  0.7063805121948632  0.7020519960153032
  0.7853211382246630  0.8773358134262109  0.4225976526186832
  0.9114075025424512  0.9042349066690510  0.2114304991156610
  0.6853574378344732  0.9127964126316954  0.7006581805544017
  0.9549846710445243  0.4666008266119793  0.8442661359222918
  0.0112357276341259  0.2570995425184095  0.8645090456205733
  0.7323884616615246  0.7238533927163195  0.8898840655433911
  0.4523879267911768  0.9788589104360892  0.7373998587705248
  0.2489189674350450  0.0326809906184489  0.8750259293303665
  0.7349988943437404  0.7386366826731970  0.6589983669895223
  0.9184273269047132  0.5284459828791047  0.0930307484311554
  0.0989178750724018  0.5070869948947393  0.9002430793430047
  0.4959058294618965  0.1166765020630243  0.5034746181135730
  0.9074308021829057  0.1172667846978697  0.4499159717300556
  0.8910013577894024  0.1445675151491815  0.2770429345675683
  0.0809577814582284  0.9710308834456511  0.4975587654032179
  0.1119873123772512  0.9382690295079708  0.6605401137043373
  0.4790484830453283  0.5856991910669112  0.8796415215424744
  0.3043986218711633  0.7423821144871168  0.8588215015897849
  0.5255557539729265  0.5054416431484869  0.0546643834228151
  0.6883344393739789  0.3443520993477538  0.0767068102963617
  0.7274726281999805  0.3052864259730314  0.2401453999605966
  0.2592420808089640  0.7646402168125309  0.7046299646475798
  0.1890644928564959  0.2012283870250423  0.9987722660029783
```

0.0255358483488173	0.2247801152969529	0.1522487705713894
0.2548278268493334	0.2765511835086421	0.0544998386653782
0.1792474390950100	0.4180008501617672	0.9258255622191263
0.7844829692665620	0.0449334294218185	0.5863471081311223
0.1888135009594328	0.2211058429622677	0.6168533622319966
0.1609567742176732	0.0508192662536333	0.6279344593391453
0.2820522150548456	0.2768257300896901	0.4193902711165651
0.3702107270528189	0.1477351236633091	0.5403784971656493
0.0296685735455145	0.8307637059122891	0.5783234723862678
0.0319561184199724	0.6696844767074149	0.1580289186038143
0.1958036706075467	0.6533391261274109	1.0006438783673017
0.0885901115400853	0.4716516712071370	0.2311008220389239
0.9662251410636549	0.5318090099802552	0.1497602167435855
0.6124718723371619	0.6284851430272156	0.7605153167836034
0.6519350927200065	0.0338654657160258	0.1534044639866520
0.6521054376782357	0.2045886846270651	0.1516138307055739
0.4543513756803918	0.0875059314572787	0.2378347364742364
0.5452661725919487	0.9788640226530591	0.3559597932322917
0.6155780570403794	0.6052976048779988	1.0088449097442289
0.8484088056251485	0.8562675014018909	0.9655726714222579
1.0021653967730473	0.8404606547706731	0.8026822625017555
0.7662673689105824	0.7596028649884725	0.9360098036605933
0.8792695005120751	0.6473054072888814	0.0307780694658394
0.2195999894195715	0.0275008534245449	0.3804283550620636
0.8423232003569630	0.8363749154522249	0.3489152978099829
0.8690185739148467	0.0127403369266767	0.3278902199459413
0.7988372344374925	0.7878305332286806	0.5289104384324632
0.6477873964580539	0.8697661723270663	0.4789447639066333
0.9709905797641081	0.2458499027536562	0.3870285827267528
0.0081258472635819	0.3920762066300372	0.8051156360374057
0.8111535434670305	0.4118745124725510	0.9428560265113768
0.9888048940055962	0.6037869631783906	0.7263131504011715
0.0095866601754841	0.4595004859776843	0.9012716268170778
0.3999737418690613	0.4366358408230909	0.1882846085428805
0.3860118900983976	0.0326673885630201	0.7992631218700692
0.3490047155566594	0.8719204581950883	0.8042894786379027
0.5518247230590867	0.9266157589531345	0.7573756500728013
0.5169099128005408	0.0805429727972448	0.5950253403492491
0.3990285160251787	0.4574197906278040	0.9564365442926364
0.0246995754385723	0.5197124348753244	0.9966900845270807
0.4933090012301394	1.0103859172266030	0.5100447622140701
0.8033938459042854	0.4110885431821119	0.1993990027098528
0.0722492045308033	0.2153539749599837	0.9279255605897601
0.4036771609083257	0.7887890396238429	0.6023592882996792
0.2037293301770397	0.0941356060430076	0.7947895009194226
0.1899368287055190	0.6395658382263660	0.7674069057858333
0.9715249680910271	0.8458320796120155	0.0290095902828847
0.6001722408414718	0.2408039852310519	0.3701314465796213
0.8263779268916041	0.9682695642196885	0.1724176164254997
0.0354214355621805	0.0054509986377733	0.1225702971204787
0.8740767231908129	0.1383883055905207	0.3727632333506507
0.7980510365959397	0.2066731566627319	0.2494613758265179
-0.0022582194727660	0.0556719857047496	0.8409292174859839
0.0699494455065646	0.9833075186026138	0.5810507777439179
0.2169462797436294	0.8742104613704680	0.6333532024648902
0.1619804257040031	0.8956878860134047	0.9988353133787062
0.4147289564643322	0.6863841440997437	0.8339605251943359
0.2527114771215035	0.7583772310461542	0.7955102902734142
0.8776197101999131	0.1650512588394049	0.9632476572381954
0.5888838634139066	0.4048262666588779	0.0594969984479661
0.7070837081591027	0.3633636437376689	0.1423044980752834

```
0.2436292361433033  0.1081631448279163  0.9074248133735265
0.0919843390026769  0.2598619176881523  0.7567190723010975
0.9428026904062361  0.8054333602836892  0.2019047240147551
0.7961999419867100  0.9493205282493227  0.0575911074031411
0.0013253234174542  0.0398733532419598  0.4533581417280244
0.4944958622506025  0.5760762289603706  0.9624197819070874
0.1792563413991853  0.3481990478593918  0.1686270361968463
0.3485384420543889  0.2003967329608039  0.3120082513746525
0.8473961959887936  0.6971271966685821  0.8146042298193430
0.7026633016513997  0.8457555307739411  0.6571331148390032
0.1766157262164259  0.3039716142352440  0.3320959366750852
0.3488036096785644  0.2398263869883204  0.1530413388594680
0.8276185985317766  0.6888583993601691  0.6897916331754507
0.7026389940843090  0.8339647329254692  0.8031983150641211
0.5181160912760548  0.4534729784359791  0.7217626923851206
0.4409185658625732  0.3899015445980474  0.7394108113785310
0.5589289388790545  0.5781723376409554  0.6287451539570096
0.4039859519994667  0.4477265255597283  0.6685792716974892
0.5245028501133460  0.6363045416291436  0.5555573283855579
0.4457186699388239  0.5720951887006894  0.5744170681828377
0.4132106566366401  0.6374820185069575  0.4914395041963688
0.2310587811867159  0.8093546867200219  0.3802942337010784
0.1440801921558802  0.8390675854085492  0.3632504492957851
0.3515975556652603  0.8765663846189291  0.2825599025926401
0.1777391091626338  0.9362387503228510  0.2484755269658472
0.3853651888380249 -0.0261595456558727  0.1676931509385258
0.2984661528315816  1.0036721659448296  0.1506506841865325
0.5610939846200366  0.3885644921246951  0.7957077399618523
0.3344775104423517  0.5585417598826801  0.5145660751339223
0.6652978496630404  0.3442263130138455  0.7487971317574348
0.6943173257431839  0.2962468793726837  0.8047730251668634
0.6385689604205720  0.2834754575321993  0.7493570889498746
0.7424925488264396  0.4194595168846264  0.6561488570229614
0.3196431498972763  0.6131566048224152  0.4474418138433519
0.3766869424237647  0.4941387879044327  0.5097957510967518
0.2462435929333043  0.5077933684498277  0.6035387816893347
0.4861638503280151  0.3119267894644248  0.8893864570358051
0.5897062933470779  0.4479639516503371  0.7953448457631445
0.4090935613067392  0.2927021139402634  0.8100887142374235
0.6201066085837800  0.6301677169086242  0.6119493830098693
0.3436141786756562  0.3945938994316744  0.6856824720210296
0.5672524487153832  0.7323359466919386  0.4762791953190797
0.2043421923745507  0.7342353897541497  0.4701510661096112
0.0498882026923182  0.7867149481253448  0.4393470353366344
0.4196471091479554 -0.1457970454972322  0.2955260613360416
0.1099264591936180  0.9590789210848757  0.2351943356405737
0.4794686178747212  0.0266600471726142  0.0917839644235713
0.3244051276651885  1.0797693518179707  0.0613067207791345
0.3689047459644288  0.7015959764465173  0.4959588871922879
0.4973540214211547  0.6929353003259733  0.3985744795428660
0.4246116847349406  0.7200280622141731  0.6092452714372496
```

IWV-Al-T6-TS1-Alkylation

```
 Al     Si     O     C     H
 1.00000000000000
  11.6056394577026367    0.0835597589612007    8.7649669647216797
  -3.6825602054595947   11.3529796600341797   10.3282289505004883
  -0.2346490621566772    0.241282269865036   19.0520343780517578
 Al   Si   O    C    H
```

```
     1    37    76    16    21
Direct
  0.5432001237823672  0.9155238222233605  0.4808408726136417
  0.1735314201297462  0.2881914345227389  0.0267284742160012
  0.1676853511908147  0.1464101515397886  0.9733509463004131
  0.1220936424415816  0.3492824562005530  0.2821396485620852
  0.2719702526694878  0.1748640251652017  0.5440281174880426
  0.1400424762661568  0.1640870593735314  0.7463836677450804
  0.3544830896706803  0.1375246313928356  0.2738724152432609
  0.0806482269105449  0.5886959727495765  0.1379718802754749
  0.0468074107579716  0.8153048145722817  0.0913083881897880
  0.3041336725838695  0.3299151491678084  0.0895708851383254
  0.5888504489050707  0.0769588372431733  0.2300947098490181
  0.8157535024781980  0.0475780468155531  0.0762797310433459
  0.3243209270285152  0.3136030585726547  0.3065487572266394
  0.8829989744254534  0.7866596953097384  0.9355803906981763
  0.9202750977700338  0.9362402958388866  0.9708382491138563
  0.9307405093173726  0.7072947374470290  0.7049145128426549
  0.7972679233112741  0.8810638431741213  0.4232497862199052
  0.9396120343769871  0.9246486872223456  0.1993299381446029
  0.7030195871565444  0.9192073673128297  0.6937358435799807
  0.9674607130957456  0.4626956786467388  0.8504838168910938
  0.0416452581076405  0.2707810359681341  0.8484886000782028
  0.7493582351054747  0.7354008798397116  0.8840152921961056
  0.4711135370071697  0.9821850499939568  0.7295171135686559
  0.2698069283202427  0.0387932091302447  0.8689278733499106
  0.7436293573617258  0.7388729396472152  0.6608296075240984
  0.9278248557549995  0.5386535837944559  0.0946130640032591
  0.1077524024812003  0.5166933402069346  0.9019474877550739
  0.5164939840858188  0.1193550782615483  0.4925076079565769
  0.9348631012197620  0.1238897957522468  0.4398382704094315
  0.9058951249585984  0.1550192845691854  0.2714377680012409
  0.0945527701224882  0.9659875259361026  0.5003665763007165
  0.1183922456494894  0.9447703319799372  0.6616692666621800
  0.4907613107209372  0.5823184504982999  0.8807828049484374
  0.3128420935113723  0.7396077296122124  0.8650789086335530
  0.5485442184628337  0.5111210654547241  0.0467655437666458
  0.7038503905408664  0.3417168719911887  0.0841034345436321
  0.7382026230904133  0.3187537792745956  0.2408211220454108
  0.2723624238611999  0.7729547767617185  0.7060648908373727
  0.2234901284031073  0.2269912606700009  0.9723207671766192
  0.0327456492451605  0.2200171048931648  0.1398437686236488
  0.2503973017178630  0.2775010018232460  0.0670345043114915
  0.1915081771574019  0.4296861280248625  0.9257784705461825
  0.8060078462749636  0.0488750878622638  0.5727979058345478
  0.2158186907717354  0.2224624907315759  0.6111050419831017
  0.1669836032249715  0.0696614471628240  0.6123153018752364
  0.3172120938954240  0.2863250298864111  0.4080677840057439
  0.3788638646631413  0.1243732081029733  0.5482639927174291
  0.0430641841824817  0.8274223905676599  0.5753423568374845
  0.0429350192769506  0.6768032462228152  0.1617600968998028
  0.2100558300002102  0.6581003086746556  0.0077148690895296
  0.0932958363651512  0.4742048055882316  0.2390311396202899
  0.9787553504970775  0.5440959320910910  0.1482216082659750
  0.6221601090984878  0.6296913548162592  0.7577657220580625
  0.6812730186468745  0.0586084490063077  0.1381861335010539
  0.6485553371340625  0.1971054807193945  0.1828276832828631
  0.4709275734380950  0.0921202055090216  0.2356817545375363
  0.5533585386263057  0.9562018812381644  0.3611950640733129
  0.6341144858035663  0.6156441409345450  0.9993181074313213
  0.8543976431545282  0.8630302966062776  0.9723027206130210
```

0.0095769442202629	0.8582434124651016	0.8034198957684412
0.7766999894120930	0.7685153931440560	0.9357186535818325
0.8919232483388573	0.6588719652552463	0.0287586205175816
0.2369927303784213	0.0195429010313366	0.3892271005421401
0.8725308069618940	0.8543065129804149	0.3367616323530704
0.8630217120352706	0.0171262637317464	0.3443933893165003
0.8120360473729071	0.7901106248104739	0.5291120030407821
0.6603191238791333	0.8600365752655914	0.4775360856444913
0.9948159887663899	0.2433933297641190	0.3930651727147227
0.0373182677473878	0.4038787347019731	0.7949472692133499
0.8271705208460972	0.3838862305891813	0.9597840228747543
0.9860333185597018	0.5971199022575026	0.7402220192896651
0.0210268163904643	0.4632624639476095	0.9044707281700729
0.4245397822264376	0.4429287038174163	0.1805277989651597
0.4050791486358294	0.0409968350183370	0.7862796094944912
0.3696957357938482	0.8701698829100738	0.8020893455473008
0.5721173903398575	0.9380905778026700	0.7490002117059926
0.5358673911084426	0.0767882996023150	0.5874310613785797
0.4161330250663672	0.4500745995751003	0.9588429690366718
0.0285220333736072	0.5204780455739321	0.0050093746079485
0.5420544635752850	0.0345394766969070	0.4685552837312548
0.8080420169964746	0.4248295638770109	0.2042914127508355
0.1076766205441692	0.2255396929020883	0.9076016208919062
0.4100957374212402	0.8003668985232650	0.6100596290709066
0.2153802816157790	0.0999777586512686	0.7969508877268900
0.1929833453586729	0.6495490024350581	0.7710318773670332
0.9914840103631480	0.8555043231923046	0.0230810528040596
0.6061261462875428	0.2558384938450480	0.3675913279642316
0.8526047272805349	0.9905812072703457	0.1624593105015610
0.0643145456770941	0.0246393123179195	0.1096512727014504
0.9207022002254169	0.1661044944378687	0.3433822298661788
0.8112226626262922	0.2220993944654388	0.2513518066521544
0.0149330763814308	0.0640510037825511	0.8350355376295485
0.0709399119948353	0.9741564221982151	0.5903811461575648
0.2219925772644660	0.8803924213136020	0.6412186141881756
0.1839925000567785	0.9010926026273162	0.9933834123682106
0.4171768898428164	0.6753178392474850	0.8447860991708324
0.2495217659339924	0.7538266930324589	0.8119006156412661
0.9064000304871757	0.1807432729724392	0.9495035474379065
0.6152381814614196	0.4114607666322411	0.0519227000491068
0.7274462574998857	0.3769294985298556	0.1365644950454660
0.2729542669123270	0.1132194163942882	0.8996142190892151
0.1138982845129038	0.2732884358445833	0.7412177246045192
0.9643004645089273	0.8254607959603378	0.1900987337231203
0.8193787570942402	0.9612778334273542	0.0552015859087551
0.0246295577093169	0.0415461710795443	0.4451064947806407
0.5136809275823768	0.5750509731375441	0.9583463807680246
0.2010331576368042	0.3586369735174127	0.1689285867136677
0.3605688653928480	0.2069210623676670	0.3078472511423794
0.8660684214712173	0.7099192190131435	0.8086979606314374
0.7091386494380223	0.8446069561730997	0.6606827903098000
0.1963890439429672	0.3206333530131655	0.3306238721009686
0.3519712413054020	0.2289821820783961	0.1610180569826206
0.8320157199947608	0.6885420956260634	0.6973935514715133
0.7226611850647664	0.8461413648907335	0.7943316576756705
0.5486014686021256	0.5961089315207049	0.5854161339764978
0.4334144977256373	0.6033608713953921	0.6191066125600225
0.6464032163873725	0.6643439501029957	0.4630286194513554
0.4153629996417464	0.6783921728944118	0.5341309004889361
0.6293050356770223	0.7381048189374584	0.3774459335254476
0.5136492117809079	0.7534997683781003	0.4084831693476827

```
  0.4905667167745558  0.7990506766706165  0.3210707097136010
  0.0929182799903240  0.7999614851278692  0.3508614448923576
  0.1529570527336498  0.7717430924194610  0.2815416876951321
  0.1392522176154953  0.9098676515794472  0.3029904019224562
  0.2593297044977070  0.8534495973813353  0.1643462765110393
  0.2459445524694059  0.9913269610824904  0.1861178592595615
  0.3058876807785909  0.9632061373443570  0.1166882565790874
  0.5657692909561799  0.5152974923684710  0.6793337689732863
  0.3674585130917944  0.8163112540652896  0.3579743703169807
  0.5525814077042998  0.3893358230033591  0.7264188618961244
  0.5648653524239264  0.3315619394839666  0.7955473266745976
  0.4631273686255673  0.3489270984501615  0.7664708687410309
  0.6197804950498854  0.3921654636034311  0.6536639211822557
  0.3617373006300652  0.8565282485520428  0.2869934948769047
  0.2951181347135391  0.7310839324699373  0.4399696746764624
  0.3497416559975566  0.8755307760333482  0.3736062117103662
  0.4987066512778420  0.5092562410656880  0.7543725621690418
  0.6546960470820909  0.5531961479250294  0.6421389102035913
  0.3582409005967777  0.5510310090845542  0.7135039831933079
  0.7360659013126584  0.6589813982027407  0.4363278131068741
  0.3258415896966494  0.6853818035539774  0.5617345861616173
  0.7048180132567664  0.7891219109081934  0.2831625819234515
  0.0091694288325234  0.7372390584236845  0.4416379611545582
  0.1165452499502134  0.6861694007809471  0.3186850565096939
  0.0916680172311424  0.9319395744314770  0.3569917157874525
  0.3055963727312070  0.8317698611191464  0.1099820568743919
  0.2820611430108623  0.0770146902141466  0.1487971033750867
  0.3884246821029858  0.0272769039470052  0.0249996022824170
  0.5617246544477954  0.8823036377971351  0.2315735057142522
  0.5015834230558244  0.7333119761672455  0.3138737247804392
  0.5313419719420784  0.8478987992906789  0.3896923773252607
```

IWV-Al-T6-DEBH+Benzene

```
   Al     Si     O     C     H
   1.00000000000000
    11.6056394577026367    0.0835597589612007    8.7649669647216797
    -3.6825602054595947   11.3529796600341797   10.3282289505004883
    -0.2346490621566772    0.2412826269865036   19.0520343780517578
   Al   Si   O    C    H
     1    37    76    16    21
Direct
  0.5284928313134958  0.9170409803821131  0.4817761476507360
  0.1627637746751929  0.2836352876563824  0.0336126885733014
  0.1519520907747680  0.1552517612434008  0.9692333338789677
  0.1108057523823817  0.3562751909205096  0.2758164927349897
  0.2613345701487162  0.1810896047150214  0.5426127753330549
  0.1240141950579296  0.1674543967435971  0.7475903749582192
  0.3429957179734540  0.1452367876092535  0.2730803441117561
  0.0689099118732370  0.5953289926915561  0.1322043684742985
  0.0312681214933751  0.8178809036538796  0.0941649971032199
  0.2970402151140460  0.3368973221878524  0.0851555522959587
  0.5768531747740504  0.0861394782637863  0.2281949066518749
  0.7944962432198474  0.0451069882074563  0.0831024174397192
  0.3103448598269059  0.3198774263977878  0.3054836083345588
  0.8738143013145213  0.7905109891411235  0.9342405810581855
  0.9029981303323316  0.9407949955785556  0.9721973707973602
  0.9205241441027626  0.7154071653844184  0.7023936805904416
  0.7801415879782477  0.8854277102466094  0.4263023238528945
  0.9190804803203926  0.9255366343056598  0.2024501620938461
```

0.6903315200287891	0.9271064249139354	0.6940146715505844
0.9551133396458982	0.4730756373298456	0.8439866596670101
0.0274932319339841	0.2757597896253011	0.8491569376440188
0.7370130343475630	0.7416539052050977	0.8834248271862364
0.4580660712193161	0.9885599854271032	0.7295852895762448
0.2544598521596063	0.0421128977587394	0.8701028132696036
0.7347278133161190	0.7476122566174007	0.6587481886111852
0.9118006543474485	0.5400115896899804	0.0967999073519152
0.0897090358515096	0.5126648421936416	0.9074240976245175
0.5035859068557910	0.1181172256433217	0.4977050098521610
0.9148578848467540	0.1336827124866138	0.4420791300806415
0.8929044834033411	0.1588814530711585	0.2733659922376996
0.0784147811024766	0.9780950312801271	0.4995991474229672
0.1097436716353133	0.9453503879889804	0.6612479185470687
0.4786847530503344	0.5932541521323492	0.8791939587506069
0.3025008046597614	0.7512761409652146	0.8558105625294539
0.5314538071148065	0.5227312213462523	0.0467012681046697
0.6908608163796637	0.3575786088899041	0.0739760120094470
0.7321747885217941	0.3240228794653191	0.2317445083486616
0.2618747478944743	0.7712063725021725	0.7004841435858387
0.2083679056114653	0.2239300374008270	0.9780159361424272
0.0294356628841758	0.2030390490951436	0.1585722044988337
0.2550971673062475	0.2858616549933184	0.0542582975845853
0.1665513434189397	0.4207536004091092	0.9394844820923383
0.7878144811459913	0.0602596080320067	0.5763372566778122
0.1994016483247983	0.2260441494595989	0.6124140362197243
0.1665042384450934	0.0657312226578696	0.6136049855570112
0.2956729998311775	0.2911100434991236	0.4109918488230904
0.3753313496613437	0.1451170145827488	0.5383578905637588
0.0276500259698987	0.8394632275860543	0.5742528284269253
0.0281039669543841	0.6804696749390621	0.1595791556342544
0.1920563258395404	0.6707912365010241	0.9968028094595009
0.0926137269031003	0.4860010442641989	0.2255766493244981
0.9639837955023752	0.5417374341206634	0.1514104094615883
0.6114621664857840	0.6392562148406977	0.7575668875460130
0.6641465578503948	0.0647359664322286	0.1372769006210622
0.6399235373018811	0.2153423226457774	0.1654456846088772
0.4543264569162091	0.0931760406511904	0.2414386719070973
0.5537143772801817	0.9815032097040175	0.3521977772759378
0.6180650306496864	0.6254873841141099	1.0003747818755511
0.8425802257204512	0.8662663667492254	0.9715126491855796
1.0026707573254212	0.8587308819234633	0.8038815644196007
0.7686238871139408	0.7749346615847028	0.9323861328148159
0.8790961865643809	0.6622569400279119	0.0304745716186229
0.2196783191082247	0.0342240607656876	0.3870906404449297
0.8617622620340953	0.8638486801831305	0.3353519863356975
0.8345626674878424	0.0227849520301970	0.3514074172465507
0.8009540605861485	0.7973063038134500	0.5278822930587017
0.6419226162099593	0.8556932683086379	0.4858910564059289
0.9786016650473883	0.2574304563980207	0.3868215123510563
0.0156113558843963	0.4077492106435381	0.7946040954396449
0.8121489748602927	0.4082392773734392	0.9449134556293658
0.9850457138963680	0.6114135504249654	0.7300922305358960
0.0064380073155024	0.4625120189808231	0.9047271573882465
0.4081182427183929	0.4515468677432612	0.1816607837511939
0.3906574779222506	0.0454163222623435	0.7883431759288981
0.3561745531700536	0.8807917117581582	0.7966137561701673
0.5559291364692449	0.9384488922436948	0.7529411544237687
0.5258857073065061	0.0881834076194805	0.5865342379973466
0.4066425096244481	0.4603206578497983	0.9567763638508773
0.0103779534286238	0.5224048953132375	1.0060135928641751

0.5061912201694735	0.0112694591986259	0.5048474217946113
0.7902663663747316	0.4281962556041496	0.2055339785485273
0.1137722617072193	0.2515693250833643	0.8850344018200655
0.3975945804974185	0.7921638085688307	0.6012850445305321
0.2021185036449898	0.1094018641258098	0.7957948983555116
0.1809414701774697	0.6430530544748303	0.7756544418069877
0.9665182447175249	0.8602233942919336	0.0338537001015434
0.6042249962153740	0.2439496870249879	0.3631640060593442
0.8179839212080429	0.9662140915827139	0.1821240469104048
0.0347066397170111	0.0418096194735813	0.1011340720008902
0.8915315030732056	0.1688826748174688	0.3533665272901100
0.8215890814854425	0.2429748346015434	0.2237347433326634
1.0032617820888685	0.0638233013044212	0.8351173321028513
0.0571414520088238	0.9825843407027754	0.5913468201285710
0.2079341015611611	0.8759042011942687	0.6381777454727183
0.1692343941851580	0.9039755035847455	0.9908514755183645
0.4062147395170728	0.6857732585101537	0.8402065680650965
0.2507704652941586	0.7668468923260714	0.7934900646867831
0.8977404167826096	0.1742643846548238	0.9661194494412905
0.5988661274365364	0.4257253235235133	0.0473063513346044
0.7170026655917158	0.3871503268039011	0.1291786912156555
0.2539873141109202	0.1105835874853835	0.9076066433195733
0.0893664331157612	0.2738104314689051	0.7460109120346929
0.9590992195357623	0.8257178598895133	0.1946656248060261
0.8001902888321631	0.9740231830853157	0.0466823400287136
1.0047242490045238	0.0517185298206321	0.4485528418328013
0.4964938443790174	0.5894605581347954	0.9577822580625568
0.1851779954209867	0.3596609406194626	0.1665844590400958
0.3531518430939592	0.2154335019115147	0.3050718160978214
0.8505746562801879	0.7115449278651205	0.8109971816033887
0.7046543129653844	0.8554136282932636	0.6556977545600954
0.1840317853526212	0.3229547400592432	0.3263113910482128
0.3464073352385960	0.2404811991039187	0.1564124334198522
0.8231599028084572	0.6936622417303600	0.6948566692942872
0.7123545969501234	0.8541837826983038	0.7927931906895979
0.5890447825476408	0.4922696992689915	0.6802600910381864
0.4659622455743296	0.4458431499537375	0.7446504483317999
0.6570244621938559	0.6028094458404494	0.5595130856200233
0.4117250785542813	0.5081887497426648	0.6918925019368547
0.6035837665880723	0.6673847579735019	0.5064378961851197
0.4758169032014559	0.6264628375542124	0.5716553705293925
0.4391460493023621	0.6679896652574746	0.4937772227604045
0.2349261757182011	0.8578876621053708	0.3529458657724124
0.1518956933789604	0.8883806973233258	0.3307841519276764
0.3575079779767699	0.9038600611840402	0.2580461261163830
0.1917162224802218	0.9651708465199557	0.2135081420691252
0.3973494482223149	0.9807662088598049	0.1406473851819346
0.3144184380282525	1.0115041436378867	0.1183355413744013
0.6489216449044585	0.4289685701486716	0.7372398762390849
0.3045788823192283	0.6288848240835327	0.5624445083439356
0.5814735487906587	0.2992275491261031	0.8568531228308253
0.6349584695204371	0.2630801696719096	0.8886027930258397
0.4953616661155583	0.2902549291740383	0.9266093936063855
0.5634389514570334	0.2433309551985890	0.8484246538829181
0.2868817913173692	0.6634983677007014	0.5003547747765786
0.2665869132985638	0.5312735537278424	0.6312892013787581
0.2562766741704458	0.6623467118898293	0.6063496563421383
0.6655284662113827	0.4829328480631503	0.7485974624109398
0.7390761418412943	0.4376935690212567	0.6707819845357288
0.4146200896917931	0.3590811161044989	0.8362750407362770
0.7525994462327520	0.6373129030784200	0.5088403090897855

```
  0.3169041298963322  0.4710338739076930  0.7426983484747163
  0.6558501560494391  0.7535171697255966  0.4139375509839729
  0.2035204560264147  0.7998529607252256  0.4442455059356477
  0.0561706271231902  0.8526693632577532  0.4047897768438249
  0.4225293134779474  0.8812298991520323  0.2749793606993042
  0.1265966940646694  0.9887615453628816  0.1967801029474016
  0.4931591334916461  1.0170447843837520  0.0666769926416731
  0.3451270624921606  1.0716257523614297  0.0268752030910435
  0.4754621049612463  0.7659158649019826  0.4222748804081834
  0.4851321360678082  0.6334735585021113  0.4505869650826763
  0.4399851932046343  0.6853946619084484  0.5951987602130040
```

IWV-Al-T6-TS2-Alkylation

```
   Al     Si     O     C     H
   1.00000000000000
    11.6056394577026367    0.0835597589612007    8.7649669647216797
    -3.6825602054595947   11.3529796600341797   10.3282289505004883
    -0.2346490621566772    0.241282269865036   19.0520343780517578
   Al   Si   O    C    H
     1    37    76    16    21
Direct
  0.5237236146376476  0.9253812925969497  0.4812402179794830
  0.1542917528220009  0.2903091600350733  0.0295090900833781
  0.1344362111870429  0.1451435644759319  0.9828619065466292
  0.1011982472400955  0.3549017078142112  0.2763670991162994
  0.2478548710183526  0.1812414767214289  0.5454153940762043
  0.1146973080513307  0.1651109096272260  0.7511552381111681
  0.3337585928644882  0.1452867764444621  0.2732623120169210
  0.0608023055395590  0.5939209669216816  0.1341182945882964
  0.0199139680344484  0.8145295100250711  0.0967511928552905
  0.2885246011072738  0.3379415643111650  0.0841896184192367
  0.5666379476206035  0.0841498513219908  0.2306838387581680
  0.7835046141757402  0.0432542143621293  0.0847209470245707
  0.2990605236122033  0.3178885730158999  0.3079205314147537
  0.8646569275839042  0.7877500234563251  0.9352991837789112
  0.8946401506854859  0.9375733784993225  0.9739327878398835
  0.9120455483451495  0.7147968146503355  0.7021223207300538
  0.7748213686218960  0.8836525897779632  0.4250339034849895
  0.9079495981305657  0.9192324107155438  0.2079340875595659
  0.6814906850677769  0.9264811014584993  0.6954558301452284
  0.9485469126212184  0.4743893386386810  0.8439331202346947
  0.0099575150722350  0.2699929436406560  0.8576878985596929
  0.7269330872204590  0.7391099347373802  0.8852449986039287
  0.4477697703627162  0.9904272464136793  0.7320632715384510
  0.2423451356284613  0.0394018702293104  0.8754368505838033
  0.7262362130720741  0.7477226776596863  0.6588943725591082
  0.9090677097633905  0.5394440182393887  0.0935130668720016
  0.0894160666523106  0.5183808790851352  0.9002346160136736
  0.4931153888995699  0.1252380400347977  0.4990534867482538
  0.9047700568846859  0.1309018055970856  0.4428151673948287
  0.8814061937759272  0.1570843994487043  0.2734240765635896
  0.0699571722037637  0.9775417093766834  0.4986888994861522
  0.0990607418906936  0.9477586020939304  0.6609135718492286
  0.4692132987620513  0.5938707300535492  0.8785781982340084
  0.2930969608972889  0.7523105478965135  0.8578204813496608
  0.5209096160511536  0.5199499907504814  0.0482366874849474
  0.6817296162408765  0.3566137689849155  0.0744931599602232
  0.7183487024634042  0.3210612708454887  0.2367342812930752
  0.2530488939174202  0.7771535292615245  0.7012552293528349
```

0.1920754522033889	0.2199611618626394	0.9854596808962937
0.0160520965342655	0.2275444579883547	0.1475249309668792
0.2425693776309140	0.2848559386561617	0.0571518095240478
0.1721024413751652	0.4302325202216665	0.9243053234576661
0.7805373664138228	0.0587841758464262	0.5784063771149135
0.1884558571398478	0.2263519474110253	0.6151184702693294
0.1489368772088105	0.0681673722414768	0.6173696439138328
0.2838390332542359	0.2909937407614018	0.4129454948742799
0.3604720244985873	0.1411706658583554	0.5422016868583363
0.0193795623336089	0.8388523698785013	0.5735057471022171
0.0174626717820905	0.6771345207801670	0.1636687179746927
0.1856870024594067	0.6719678115893761	0.9993967334750913
0.0827870946700442	0.4839131239259459	0.2286431062532158
0.9585646132484886	0.5416821284585259	0.1497176582898702
0.6017589257293913	0.6421525687423716	0.7563716913968439
0.6483846391130675	0.0505834183253907	0.1497078155272668
0.6365722784605572	0.2152268421782034	0.1589494996094120
0.4451012684119603	0.0929906347097360	0.2419771961654049
0.5396185326858520	0.9854835512120710	0.3570602762797621
0.6087660672575430	0.6221640690798318	1.0015018769993529
0.8347164252310992	0.8646713294173788	0.9713601546777033
0.9918709426985565	0.8568480488146475	0.8036557052842710
0.7577391412211975	0.7700474859677486	0.9363150135998853
0.8729835882054462	0.6600973981982824	0.0293736449853306
0.2112215884686315	0.0332275570869667	0.3859479615253569
0.8429681337242969	0.8487519764789940	0.3451576316405773
0.8459942011089784	0.0225394071910951	0.3346481375006071
0.7946864228361220	0.7990447649826201	0.5270762067625377
0.6361778860568000	0.8617719418856951	0.4843705990082756
0.9689240548649057	0.2570384969229830	0.3835544522507872
0.0075326364604463	0.4050153391269616	0.7988801843391002
0.8053968460007863	0.4125787466922606	0.9445498283620495
0.9781899729830821	0.6118303855865398	0.7288101994420891
0.0021171400550924	0.4671436234746431	0.9016732267742673
0.3971650222856196	0.4492529092497963	0.1831568362782641
0.3788054368334045	0.0402157517333293	0.7978921788888623
0.3485104499827563	0.8827607114396258	0.7959316705198447
0.5481785215393132	0.9406318715483752	0.7513101982497533
0.5106254340674987	0.0964166383475989	0.5902134641920143
0.3976353702899572	0.4612523092069857	0.9548710740837318
0.0120464451682427	0.5249776353231961	1.0011563194649946
0.5081526917027127	0.0211672902770404	0.5007221843550198
0.7903883375351322	0.4253383949002414	0.2007748948902942
0.0764661827556055	0.2291098939941105	0.9160591031601010
0.3915276908397643	0.8010433699069397	0.6039222605052287
0.1931641827285606	0.1018796891225669	0.7985840787142924
0.1767118792596737	0.6509211955468086	0.7688132714342706
0.9637546224134516	0.8573409535612216	0.0288915368306929
0.5890535469477222	0.2551727789899849	0.3660338748253067
0.8205612306324797	0.9812604869083932	0.1717499804742053
0.0313081875658152	0.0219697922998265	0.1177902207914884
0.8756853866105719	0.1579547645871633	0.3606934013661094
0.7922914243311746	0.2255610689548591	0.2431574059576215
0.9909407171860962	0.0647384567176029	0.8385998535989677
0.0492993257729099	0.9831889999970739	0.5894882039503975
0.2023279401774183	0.8832087836799860	0.6357580549220213
0.1561019672169973	0.9019591183247007	0.9991571626105065
0.3960098863365100	0.6863741026730473	0.8407062417278323
0.2356900259323922	0.7672412823276999	0.7996835825453886
0.8751899944949358	0.1783451335900328	0.9604994745884823
0.5878160786195780	0.4222161350009783	0.0497346055082450

```
  0.7024860635904535  0.3820642723152951  0.1351093974477494
  0.2406649965396318  0.1135498254415624  0.9080303283459746
  0.0851263990946527  0.2706167392814094  0.7514251947847922
  0.9378453035496636  0.8197187710396797  0.1991447520819165
  0.7909845019942503  0.9634650330253504  0.0559460561154095
 -0.0032281626683196  0.0512492011731493  0.4472508378259700
  0.4859449049600092  0.5871026746077388  0.9593254829028427
  0.1781713679682922  0.3625083946703419  0.1645051106417815
  0.3422323094695572  0.2128703066865873  0.3083147298396745
  0.8419034553212357  0.7119674023968284  0.8102005666293575
  0.6973428920331290  0.8555305590042495  0.6561804063363431
  0.1722587906800968  0.3186931391086093  0.3296543088191131
  0.3377570932882582  0.2419945864529542  0.1561903223350377
  0.8138856571142293  0.6919473107755428  0.6960042188194739
  0.7004200003219505  0.8520086705164204  0.7966455248998268
  0.6038354150269388  0.4139346610316990  0.6995365308812969
  0.6774704729462895  0.5185234781101267  0.5762243252352584
  0.4823597717560077  0.3704665504199290  0.7588427177095255
  0.6331762630178663  0.5741997787298911  0.5125358757964614
  0.4368596068892471  0.4257145922755737  0.6961381250528613
  0.5115822015983991  0.5286802706545443  0.5719067291308286
  0.4378396639975653  0.6756839976031596  0.5749967221816313
  0.2508769064517522  0.8491173551904166  0.3488368538630057
  0.1617884310236137  0.8521798872524807  0.3392365000344707
  0.3717749623057235  0.8986302606762930  0.2491740316148831
  0.1937188641692399  0.9046419248135933  0.2299576359455371
  0.4037197288516083  0.9510183543400561  0.1398912334301802
  0.3146919459544096  0.9540874242217303  0.1302048614879712
  0.6552725578155653  0.3486715547744265  0.7655157965924188
  0.4012497043706322  0.7228640379106057  0.5000921968826101
  0.6814746493107213  0.4128582760022749  0.7894441709617128
  0.7221537498923019  0.3633397581773447  0.8344672149167043
  0.7436443617891966  0.5034452234833883  0.7052892268604769
  0.5987536453495721  0.4202493288763667  0.8466076086610859
  0.3797639357253286  0.8050715090394034  0.4783072385616988
  0.4752692618383820  0.7508683715019447  0.4133217667706803
  0.3218764624234502  0.6602956698682632  0.5463050846804798
  0.7393253777476572  0.3389084878118205  0.7115858557733932
  0.5929109699673500  0.2583536379846482  0.8510158379445971
  0.7713506832828345  0.5540426196528000  0.5296703252318955
  0.4244114844717417  0.2902673746234610  0.8544025990587802
  0.6925951096357060  0.6525351713872305  0.4163255966708930
  0.3435684610500368  0.3887418036452646  0.7425372217363664
  0.2252885934151221  0.8092748458657431  0.4339717576562045
  0.0673921011606366  0.8141772879046610  0.4167541943649569
  0.4417321751318449  0.8983049265709290  0.2554101211949614
  0.1242293016086688  0.9072842643465002  0.2224649377115288
  0.4982245183094861  0.9892642840744678  0.0624275726960619
  0.3394375056203102  0.9947855707083190  0.0450413083445109
  0.3744100243278607  0.6064888644431142  0.6721320805466462
  0.5265258031135069  0.7143462940531450  0.5360437967844197
  0.4822516227849228  0.5585556090646900  0.5197547097431007
```

IWV-Al-T6-Et+EB+Bz

```
   Al     Si     O     C     H
   1.00000000000000
    11.6056394577026367    0.0835597589612007    8.7649669647216797
    -3.6825602054595947   11.3529796600341797   10.3282289505004883
    -0.2346490621566772    0.2412826269865036   19.0520343780517578
```

```
   Al   Si   O    C    H
     1    37    76    16    21
Direct
  0.5230328498081941  0.9255280328912469  0.4804512574621491
  0.1540965869487078  0.2827787336332080  0.0349182649955757
  0.1317513123827402  0.1470283213928884  0.9818416394349332
  0.1045000262041984  0.3598892648640635  0.2676328940046758
  0.2461165968392462  0.1835798900893222  0.5445554046254177
  0.1120289299035774  0.1664728003423668  0.7511801292311606
  0.3334899000103401  0.1458435413933645  0.2748864027621843
  0.0561243732584385  0.5971305290044430  0.1268148401925599
  0.0186521614802422  0.8156619888934539  0.0963406414791522
  0.2937660255358411  0.3334053058187269  0.0826338693362637
  0.5709628716992842  0.0909807548165618  0.2251954822334910
  0.7806074005678821  0.0433056714958641  0.0848413131923471
  0.2968818462064792  0.3233043188923148  0.3056450092079822
  0.8613055944530540  0.7888618555872310  0.9368564254204765
  0.8912033171703765  0.9388761068424499  0.9747371954693607
  0.9126012849186292  0.7189364439796236  0.7000724230026436
  0.7645015170728873  0.8823356107739230  0.4296371358079601
  0.9074461708618546  0.9197798360466906  0.2075996849464742
  0.6836997175686216  0.9324747290381920  0.6923066658471813
  0.9492529555731327  0.4811354260692693  0.8394926629831314
  0.0041367474999788  0.2707991218157940  0.8594519396654994
  0.7274041209246508  0.7430915648555217  0.8827353118480360
  0.4458727550893171  0.9879008331507971  0.7364554021577642
  0.2406795135730942  0.0415922490360444  0.8735351760239438
  0.7256668384598238  0.7519509517636972  0.6581525391191897
  0.8934339938817092  0.5350999606859940  0.1039799095593500
  0.0785736770335046  0.5109554107430265  0.9135071545820443
  0.4859589920044814  0.1147942063444526  0.5066958177827884
  0.9036783587152485  0.1379323519195253  0.4388038686029233
  0.8810369158842687  0.1571196733902481  0.2731082762208782
  0.0662257240755467  0.9837941398126918  0.4981504910760157
  0.0928276741584037  0.9489822607639089  0.6621024470738410
  0.4667843060514023  0.5925409961214815  0.8814930526975288
  0.2937154740088316  0.7549436659320878  0.8517904591374394
  0.5170552683767958  0.5258025396062734  0.0478419260844761
  0.6802501570805910  0.3653536647782063  0.0668467449681677
  0.7177541202779983  0.3190332996504835  0.2350110354528931
  0.2465040610235398  0.7792374957398048  0.6971646818696012
  0.1923085775155872  0.2224122970970209  0.9816074849573608
  0.0209869813220784  0.2028489116829955  0.1626358135254146
  0.2500990943964585  0.2828014400751223  0.0526815581670984
  0.1564831996551082  0.4206496081080889  0.9432787877520380
  0.7810211163592127  0.0657407483208042  0.5753205764303500
  0.1840331645618290  0.2272324553099238  0.6154557683534626
  0.1531007765627875  0.0652357733326869  0.6175239737295006
  0.2762277116151202  0.2917645513158559  0.4148491617919151
  0.3633952868177774  0.1514165699687062  0.5349825531871394
  0.0166141176732778  0.8453880922546609  0.5723833498085659
  0.0217626180882603  0.6806055903885256  0.1556121376979575
  0.1656725382369590  0.6763888848872541  0.9846129284983588
  0.0957559450531196  0.4940908642998300  0.2068260626271718
  0.9417085193230375  0.5349850473569651  0.1623127630509663
  0.6007189295542560  0.6455203425574449  0.7584338306710749
  0.6435504391560054  0.0450152775560935  0.1555505108643012
  0.6427435514229430  0.2285815654569620  0.1350595251198916
  0.4397451670765170  0.0849444684203541  0.2560179742191956
  0.5608627812934400  1.0107521544498064  0.3422403832864872
  0.6071654329787596  0.6286429187542923  0.9981024224437627
```

0.8315896312357706	0.8660049492304207	0.9720881147724458
0.9895924644348180	0.8576347375076456	0.8053679107880736
0.7560733602317777	0.7725608607140544	0.9357374181918929
0.8680012023280584	0.6608773071657073	0.0317423516812376
0.2083726635610865	0.0370337983723355	0.3890759312458722
0.8376933022119459	0.8542198750234976	0.3444395013428290
0.8197269797484950	0.0240609006706524	0.3449543457933993
0.7903853555949303	0.8029110542125844	0.5271174850961255
0.6229181629726743	0.8487245341328807	0.4976256711726763
0.9692899724525702	0.2664482462659875	0.3749525096855781
0.0012511224426797	0.4059745259209686	0.8008116211014266
0.8051693569393983	0.4325057313377308	0.9344780884793890
0.9846015199611289	0.6200616550690577	0.7216650459100070
0.0024570897697902	0.4699185035907636	0.8991368715837605
0.3960227128879305	0.4546983056081110	0.1841227473360716
0.3777559124214783	0.0422699569604606	0.7974191515550242
0.3463084300537824	0.8742854551624364	0.8085024244063943
0.5495475466385220	0.9440997964518236	0.7514671265782781
0.5050577331715960	0.0888570753407423	0.5954265100293050
0.4023401568362567	0.4581163753274134	0.9565404820754468
-0.0064594455261208	0.5146625336552133	1.0161525370718618
0.4805694170623867	-0.0009693376529373	0.5272407393497380
0.7703109706932212	0.4263244801068679	0.2087523624891212
0.0692339195735132	0.2280386015428191	0.9193613036005327
0.3807608791168437	0.8052233978528223	0.5808298238960643
0.1941919073230153	0.1049348901684481	0.7942347353183670
0.1679805776367092	0.6456317674688511	0.7868738635737742
0.9574904994671704	0.8581160197937335	0.0328004771200572
0.5945183210625790	0.2315161641440671	0.3698862139620549
0.8225284623369339	0.9809888033123162	0.1678513471405060
0.0299313863309898	0.0233163900664151	0.1183254222816225
0.8696643875552906	0.1594346928939743	0.3619741273826449
0.8148016048280278	0.2465443037010899	0.2184130158032511
0.9894600071927142	0.0645012604557749	0.8391514144121601
0.0411091414384521	0.9892211774749918	0.5910024138596626
0.1901525667288938	0.8786644790777095	0.6363838423562328
0.1548576558567644	0.9042018481427041	0.9958084271246814
0.3871997596090420	0.6782138238270624	0.8481607765829531
0.2709235399556789	0.7943919618937628	0.7606449008219501
0.8685964476058755	0.1802217387732922	0.9618676208396828
0.5833945805125970	0.4289534449456877	0.0471508993027286
0.6942012861262001	0.3773240089847268	0.1390800962973348
0.2367965956701463	0.1152604463572557	0.9067983507014293
0.0800788155235644	0.2703530826098823	0.7537056036940937
0.9410256747090961	0.8194816893451022	0.1997227912204540
0.7863315343690535	0.9664958649908895	0.0539708801594678
0.9966497278908538	0.0591164636444328	0.4428770336624301
0.4781060692473202	0.5910745090337168	0.9623100397568711
0.1806875481619123	0.3549310873157442	0.1645074867258908
0.3430788722321918	0.2204339375761213	0.3031519365033729
0.8415274333538398	0.7130666566144561	0.8103067250950772
0.6983585084258777	0.8609882980196646	0.6537612125704788
0.1721064179047300	0.3260071912344715	0.3236094800269683
0.3428620552151677	0.2369258461264557	0.1549352360236124
0.8148938372431377	0.6964661178381858	0.6933097861189568
0.7056986520724294	0.8585054603343230	0.7911644531773434
0.5291667643153437	0.3707085410298515	0.7767161545588666
0.4845543364500579	0.3149408904371309	0.7639828805608651
0.5646925610497111	0.4957460227133001	0.6945737735408735
0.4766889475630902	0.3819280209938669	0.6712471464984058
0.5571012615319706	0.5633928263395943	0.6015411466081440

```
  0.5135071436589650  0.5065514987366233  0.5892961176346189
  0.3877928823491094  0.7842140531759905  0.5107877631290956
  0.2465933190563920  0.8458897856936077  0.3564167145146426
  0.1660906092663853  0.8643837130721772  0.3329441646881640
  0.3699900778885987  0.8901439725460472  0.2640921363092121
  0.2091323118317274  0.9268266072160214  0.2171884871423476
  0.4131763031023972  0.9529484530944329  0.1481883983068119
  0.3326374326647883  0.9711007436602908  0.1248548285172209
  0.5427560028497872  0.2983035702169327  0.8735392044438182
  0.3053340436311266  0.6677267542473775  0.5852479700136707
  0.6639541343702723  0.2728209593979549  0.8332412052402534
  0.6718118110332576  0.2198942294654758  0.9052585953076385
  0.6743397816055825  0.2232907478621466  0.8085485325716231
  0.7384481364459596  0.3566092011910975  0.7542362450636160
  0.3321501573389572  0.6616907087862941  0.5214984911942200
  0.3166221741864645  0.5907847009565080  0.6510233575810563
  0.2110839401894113  0.6653425414162403  0.6289835742018688
  0.4695853130969393  0.2134352859272162  0.9543298232580057
  0.5350261509363476  0.3469966532629515  0.8983186377728740
  0.4546383071370678  0.2176954315297625  0.8281045413362583
  0.5986427564486855  0.5405337883701107  0.7038018508441681
  0.4417322440060849  0.3365319388452269  0.6631351368461558
  0.5859073248879972  0.6605269932188937  0.5388968460871428
  0.2123210477756233  0.7975602041517034  0.4468141566413978
  0.0697429467148232  0.8300470436740992  0.4048761122893357
  0.4335608826569569  0.8773245787627599  0.2811654194776416
  0.1462801048129238  0.9408810895080743  0.1990805950107067
  0.5095820889572253  0.9879053593295071  0.0762802997195389
  0.3660560444265235  1.0199110493091503  0.0345440754104281
  0.3710900742632055  0.8613165740546522  0.4504369877403624
  0.4817484211936962  0.7883950002954377  0.4582702439087895
  0.5082408254636902  0.5588059420490208  0.5162969026670479
```

IWV-Al-T6-TS3-Alkylation

```
  Al      Si      O      C      H
   1.00000000000000
    11.6056394577026367    0.0835597589612007    8.7649669647216797
    -3.6825602054595947   11.3529796600341797   10.3282289505004883
    -0.2346490621566772    0.241282269865036   19.0520343780517578
   Al   Si   O    C    H
     1    37    76    16    21
Direct
  0.5143037412724533  0.9239478817393154  0.4843899011165853
  0.1453337216595381  0.2995655041743088  0.0324076078776190
  0.1379661763561144  0.1640778671494736  0.9742312562840751
  0.0951293867204383  0.3671937735099753  0.2793759395426533
  0.2426860507041262  0.1879176640574708  0.5474109160563266
  0.1133865458823303  0.1807743435013371  0.7473332103684595
  0.3278613585686937  0.1541467571281590  0.2748638489418896
  0.0514146119082150  0.6051218049483491  0.1356971219011204
  0.0188467672517549  0.8312122969455360  0.0924945922040756
  0.2790827703954811  0.3456135851659738  0.0892495818169760
  0.5627710034613909  0.0954881850546935  0.2303184628141631
  0.7841899994618639  0.0609053811109588  0.0794179938275794
  0.2943901451197981  0.3291116376350869  0.3087361030298093
  0.8570768630582317  0.8009184543789284  0.9366241593308160
  0.8925150927039278  0.9537549490736101  0.9706832949708677
  0.9042268011284360  0.7251643383699948  0.7046786276935911
  0.7638919878614244  0.8930363356983885  0.4284862611617035
```

0.9098772815589822	0.9393975508753631	0.2012144912225367
0.6768311159174591	0.9372390093829224	0.6944769370021735
0.9414637076715767	0.4832207817326199	0.8474313741913038
0.0117019876133885	0.2866166204611217	0.8504832213055045
0.7213989537105016	0.7520640921981530	0.8851772857449627
0.4429128808980007	0.9988271383131245	0.7334562527612289
0.2418126436508067	0.0549911820250107	0.8713879161195316
0.7176828275000661	0.7557161174324378	0.6618427616334027
0.8909864844271173	0.5493472274905965	0.1037410822603683
0.0733730432489408	0.5276349841231047	0.9106395943071677
0.4849475160134389	0.1241720326904691	0.5023931400070494
0.9041156316252803	0.1434696672560901	0.4400250840672429
0.8750599930174492	0.1685694292157535	0.2740583317203226
0.0652481107573917	0.9866151947535078	0.4998679837558681
0.0908927630728390	0.9578877597245201	0.6631956826715192
0.4622462592419980	0.6005097984542590	0.8819311691737111
0.2876243013102132	0.7608461407881991	0.8597709415056984
0.5168005498606201	0.5307590816236297	0.0484719188428192
0.6760452655510698	0.3661173000487102	0.0777029261241053
0.7096554713476829	0.3331864403177632	0.2392817101729107
0.2453779788840161	0.7884598674524274	0.7013983814149406
0.1953240631619987	0.2423609383161628	0.9740945501920255
0.0077203262011208	0.2208251491579863	0.1511408165949278
0.2279006238389900	0.2914179153090438	0.0664275024173834
0.1551592771959676	0.4399213640031199	0.9380448748832387
0.7773752186220961	0.0682628612404683	0.5744036835075605
0.1895879383838319	0.2373417495327242	0.6127117885316324
0.1373652899429247	0.0791693044877022	0.6205115390404169
0.2826431341284548	0.2990211953930300	0.4135400952787288
0.3514743800914184	0.1392106925593213	0.5478304181406479
0.0126995486345146	0.8482525058304123	0.5747799025060791
0.0167925845723815	0.6939608986998150	0.1583271363090178
0.1730677841050559	0.6762800512095354	1.0001387728690814
0.0744406910774545	0.4960175150822935	0.2298503554051827
0.9427085945359805	0.5518397997533299	0.1584108059954055
0.5948691767637018	0.6472343064252740	0.7601879527647034
0.6504799075933306	0.0726716903059588	0.1405921067428153
0.6255853550910676	0.2236021488472830	0.1694551291361051
0.4421212057031645	0.1062284692142154	0.2394857292180597
0.5355804137962601	0.9894543497401850	0.3560715134505993
0.6031131903508848	0.6352988637960861	0.0004799146750945
0.8316342490071598	0.8818917098407955	0.9677384298384761
0.9848628388100834	0.8647306436358455	0.8064371833048368
0.7506366070936695	0.7840396129277907	0.9365313768411910
0.8599608111624647	0.6726776606362832	0.0356288443687935
0.2075516135506333	0.0386389341020749	0.3889946890387825
0.8459627571870050	0.8722867857461157	0.3371400848945483
0.8175939838848334	0.0319852545894876	0.3520942471037684
0.7849796134130008	0.8079762200580968	0.5299193384747454
0.6257948331857229	0.8593744510143920	0.4887744472708144
0.9644383611719244	0.2662692741395375	0.3884549330252854
0.0069463194704525	0.4205768336584227	0.7952270659582433
0.7993927539626388	0.4146757004939422	0.9504022748732399
0.9678863424145601	0.6203794886788372	0.7340734825912199
0.9937193336312509	0.4761757450022585	0.9058688385801577
0.3946039351968721	0.4592314229067248	0.1842305998685004
0.3779439059870707	0.0574482959065136	0.7904958721098951
0.3404247761114802	0.8860105636979382	0.8081908728044792
0.5449203018921115	0.9538549324292241	0.7510021840599619
0.5043822237025802	0.0964420972668541	0.5919783376374133
0.3888136190631226	0.4681715328149796	0.9592312049437909

0.9899102909057771	0.5303910840655033	0.0142255141277801
0.4989295082379813	0.0198361738780084	0.5044664030159887
0.7689157436806937	0.4378590567349151	0.2120335615164300
0.0807335865652958	0.2461097635793388	0.9061669587573403
0.3783015714105222	0.8039778193532292	0.6026068071620456
0.1892640849084627	0.1177528272502803	0.7974967769754295
0.1573582945523510	0.6617673135132009	0.7813672527365274
0.9621748688702146	0.8734951031655114	0.0249502408061489
0.5794402073136414	0.2551126647304057	0.3694991188115029
0.8210708068316086	1.0004240255165455	0.1662485907778400
0.0336499191280080	0.0422718242382657	0.1099481443350595
0.8833804478105045	0.1785910324062002	0.3498154913757419
0.7955177255241263	0.2489178195742811	0.2354117341606395
0.9882118321668257	0.0815296869391417	0.8356804620235782
0.0419639371029955	0.9926254503037464	0.5912474592927373
0.1960616786675267	0.8961822039245518	0.6354330028405540
0.1558815320368743	0.9174750012365211	0.9946536791030947
0.3896842494687072	0.6938054532162347	0.8438981597062780
0.2418855670183247	0.7876706277750818	0.7888512234354796
0.8777216195299778	0.1942744566817343	0.9534121760527650
0.5855330705359233	0.4344663337481641	0.0484019081795235
0.6979618582866988	0.3968483331971441	0.1343373566435966
0.2435685199176314	0.1291974058574783	0.9027115519717012
0.0860317660494082	0.2900491664562426	0.7422944580621859
0.9388301182716720	0.8396792675340485	0.1925354693551931
0.7885893504750741	0.9781131534131974	0.0543804694453250
-0.0037126197239363	0.0637162884351240	0.4440387649452526
0.4794027874394763	0.5948262521040965	0.9619005148278433
0.1726299616953726	0.3721295450704995	0.1692402630679894
0.3335230341864147	0.2234171194562592	0.3091789326272294
0.8363941734379335	0.7237717501316147	0.8114721066307489
0.6856550073482436	0.8619778067694296	0.6612691104654790
0.1673895700556783	0.3352074069382215	0.3306441502491677
0.3287317928310610	0.2474458744019367	0.1597723525872610
0.8062654800548472	0.7027650902455989	0.6982217436099736
0.6978714831206493	0.8653215740804857	0.7939146170663345
0.5412750954604127	0.4296678233784623	0.6970971041975421
0.5174185601490917	0.3796115637945206	0.6681494775947943
0.6053017218707221	0.5512687860927222	0.6068998872597644
0.5580276922863644	0.4487858495691975	0.5516527919142438
0.6458732318703823	0.6208752123037298	0.4904374784285479
0.6231045999879194	0.5696903813193418	0.4621742940775693
0.3812027195148466	0.7149906693909149	0.5432972454770433
0.3965948682017907	0.6835234493865605	0.4308435620229544
0.2709398432708748	0.6490115600390298	0.4972672807890785
0.4664331330182027	0.8015491031318227	0.3186814546452481
0.2160538020182389	0.7330616485256080	0.4547193110671560
0.4109709286413568	0.8848479460060227	0.2764509037327192
0.2859984995442630	0.8507153391173150	0.3448438059166502
0.5018406896227533	0.3539478180066373	0.8213038635509653
0.3429602184421574	0.5890989623723906	0.6511340331174027
0.5968367770099891	0.2968717620790365	0.8344413324520134
0.5664899073062469	0.2426527601983769	0.9255025850132298
0.6150315428350062	0.2391633810168283	0.8168846896213944
0.6816466198714474	0.3658409379167863	0.7703008797968978
0.4016976161486392	0.5416629283203125	0.6277668439221715
0.3572468012398444	0.5859290543346830	0.7035057836357692
0.2488049395773752	0.5434111910588376	0.7069985642171245
0.4174269021250006	0.2835675627174846	0.8873159570392766
0.4839366333313396	0.4093018459341661	0.8414344389637888
0.4661263250509750	0.2855201510036953	0.7378566655682195

```
  0.6227312890870634  0.5920086544576463  0.6285461452782430
  0.5395704710915397  0.4080435979272981  0.5305726162960148
  0.6959653722491747  0.7148500962831580  0.4220389796397268
  0.4411239212534407  0.6168085829466958  0.4594882456729030
  0.2167375174438356  0.5573213398297081  0.5826492882578876
  0.5637061416059879  0.8275887323504137  0.2656562515702345
  0.1189828357329813  0.7073802405565409  0.5072832900886188
  0.4647187245239817 -0.0235926793667421  0.1911014586831034
  0.2426157991697244 -0.0833439578334637  0.3124205398968982
  0.3190463062377906  0.7635319685298656  0.5306814184962901
  0.4759686212811129  0.7654741031680302  0.4730045645280948
  0.6564734031853012  0.6234709391503047  0.3707678710063589
```

IWV-Al-T6-EBH+EB

```
   Al     Si     O     C     H
   1.00000000000000
    11.6056394577026367    0.0835597589612007    8.7649669647216797
    -3.6825602054595947   11.3529796600341797   10.3282289505004883
    -0.2346490621566772    0.241282269865036   19.0520343780517578
   Al   Si   O    C    H
     1    37    76    16    21
Direct
  0.5166789216417107  0.9066495507240855  0.4701145207878122
  0.1486959300623132  0.2783453155884982  0.0166982593166970
  0.1369913345198414  0.1393140478303365  0.9625768914030099
  0.0961900404538203  0.3437119589504118  0.2666625568267162
  0.2450065358533744  0.1676439612687383  0.5329868582724446
  0.1110698683300489  0.1553418273258510  0.7363944022830233
  0.3288584772170673  0.1323437142893907  0.2610420285357482
  0.0561219491933747  0.5825714943292001  0.1218877364361133
  0.0184259590384847  0.8058643403754586  0.0816326193358407
  0.2802944502088455  0.3247484591966666  0.0748753831098278
  0.5616928491085355  0.0722862586458008  0.2181905183681074
  0.7813167167205420  0.0333138534341399  0.0714461266210355
  0.2960947558174766  0.3061428595743801  0.2953048239346240
  0.8589273062377445  0.7769418818432580  0.9235311031608151
  0.8910197799438971  0.9281313824339845  0.9601854435379900
  0.9060918320713552  0.7024815646560419  0.6913930988914758
  0.7686621814649965  0.8713156463030650  0.4142453714373392
  0.9067423753485536  0.9119648995573255  0.1923138553811519
  0.6776151003478408  0.9146602123007761  0.6816790976999068
  0.9423736280054831  0.4603377646612825  0.8340302266616216
  0.0110248886572619  0.2618471425615810  0.8398826319119026
  0.7223358150671958  0.7294446762288060  0.8720814606251294
  0.4447947054886594  0.9778085819663972  0.7177398124238578
  0.2411716996629682  0.0298810337970084  0.8595338829849100
  0.7198313850923257  0.7328676172616528  0.6487695355478744
  0.9016749962099385  0.5275906500090717  0.0834130836202436
  0.0819214229608192  0.5061105968966015  0.8902822067477622
  0.4895486490786595  0.1094912993237798  0.4851684990490732
  0.9036665076132726  0.1208784159975961  0.4278221857565330
  0.8774811919034287  0.1466688673673169  0.2602223072239890
  0.0658811135957537  0.9647357190158212  0.4867249344604084
  0.0933443950959674  0.9364902547756999  0.6490388369692857
  0.4630163411959418  0.5799119920204940  0.8674033097756393
  0.2881955742521133  0.7391000295309007  0.8460470938895268
  0.5170327213295840  0.5092705693542843  0.0345542406676709
  0.6764051346595462  0.3439843276506636  0.0639234406880979
  0.7127135670150627  0.3109918337661306  0.2247129069323409
```

0.2476626164825479	0.7653830250604950	0.6895126991970651
0.1949711236956161	0.2150394349563403	0.9643296690351127
0.0093337336913650	0.2089119519339359	0.1327967424335904
0.2306601081739822	0.2702648296949965	0.0511499333551321
0.1655377059076734	0.4188894401394850	0.9139056279635860
0.7763734157229910	0.0470343960493773	0.5618964259734049
0.1886131526858726	0.2158396933087570	0.6000697797172738
0.1420701201847552	0.0578150082515834	0.6051595101791022
0.2841801251033647	0.2783368843503275	0.3993584293304177
0.3548404942167429	0.1225621477456063	0.5332985543339089
0.0148579327400970	0.8256519199224099	0.5620953575685737
0.0145214811836379	0.6683258624189923	0.1483815689692560
0.1819018046951566	0.6572894263603383	0.9882695025279326
0.0761239259311737	0.4723389925649291	0.2180902324582275
0.9526732527657190	0.5305909894562897	0.1382944060697634
0.5965925030936343	0.6252365308599201	0.7472263783144315
0.6491964239310339	0.0480109874357076	0.1297427340740367
0.6253429928349090	0.2022339280296258	0.1539504930667056
0.4409550925483304	0.0815383285625345	0.2274963785601003
0.5339350692244023	0.9689631511262258	0.3442997866565968
0.6030747130581872	0.6138226251327805	0.9867897444399163
0.8299354930053259	0.8536785391703438	0.9596853214234509
0.9849883638297381	0.8469355030404353	0.7912889798025906
0.7513711454798097	0.7586818845883907	0.9253065151948113
0.8682813925352354	0.6496188344476527	0.0168240498231672
0.2074947436086275	0.0179208405016734	0.3745155828904770
0.8467430740562008	0.8446924264490804	0.3273109381586197
0.8328994312614895	0.0092871470726507	0.3329570923865079
0.7876310477039111	0.7840379940741143	0.5172456321334513
0.6312897525545157	0.8460594443010735	0.4713811492892859
0.9648124483046180	0.2433282002714157	0.3763476904541606
0.0045857161749026	0.3956873794253689	0.7835677725688390
0.8001900592241153	0.3939889132405408	0.9360543540831883
0.9708300937869691	0.5981947567778917	0.7199510046705944
0.9969903836421262	0.4526880198845133	0.8909835575461031
0.3950002034664414	0.4369747415554758	0.1706611519676875
0.3774347957454865	0.0333719057618583	0.7780364009992957
0.3435819825327620	0.8690838970902963	0.7853588575719915
0.5444010863253743	0.9290578775196636	0.7385870753467209
0.5092226679223348	0.0802151606859718	0.5754289808364622
0.3887700675958634	0.4478747882756275	0.9449354909306551
1.0016431720594730	0.5087468105455422	0.9941020304896083
0.5071144121450024	1.0088529937714208	0.4823365789994274
0.7804463515980752	0.4154189397338004	0.1919494060606641
0.0897317399719746	0.2289671742703052	0.8851330201936791
0.3832070937913493	0.7873603253553394	0.5933418886342713
0.1888859314633353	0.0963112070056494	0.7848582077713868
0.1657159639239282	0.6395887647730919	0.7602309539261141
0.9597999144269128	0.8489708041772482	0.0157325034589664
0.5819227047217967	0.2423954707430436	0.3535711799078313
0.8136505860111755	0.9662965899981075	0.1625029240048095
0.0275263348687610	0.0211134570880859	0.0966998047776895
0.8835871380387017	0.1570368160630677	0.3368713229196202
0.7897330886912819	0.2181176884951485	0.2295263443277895
0.9885162252028619	0.0542615322709803	0.8233956693456332
0.0447143793229276	0.9720259207229156	0.5765588276025525
0.1957789549212530	0.8716889866759076	0.6250354006869536
0.1560389190815538	0.8920937824414582	0.9812301452617073
0.3922777743272776	0.6745134300394331	0.8270391075579880
0.2288363849540545	0.7545579760860041	0.7893769724031717
0.8787864393989759	0.1660906780432513	0.9485058832683887

```
  0.5865364616338534  0.4135530349306072  0.0342585739612893
  0.6999991968199183  0.3731151207738695  0.1207654003883676
  0.2414387878539563  0.1013783112565885  0.8939544424191914
  0.0800584969321063  0.2636017135948501  0.7329664616381741
  0.9412473448587204  0.8135968375842870  0.1820163523971078
  0.7884731687212158  0.9565927784943228  0.0397539511089509
  0.9940039432176631  0.0393570978313737  0.4338515544232168
  0.4788287034222443  0.5734753577665714  0.9486061622630212
  0.1718378555123191  0.3493180976149420  0.1561718496261773
  0.3359706923117523  0.2002556881724119  0.2962588665356907
  0.8371164625510675  0.7008599744008905  0.7984669410529568
  0.6896826272367375  0.8399902986393598  0.6470836758039900
  0.1687442931018792  0.3100578089232429  0.3179853519516707
  0.3303106501793719  0.2280546880639709  0.1456372137348589
  0.8084581192786822  0.6791788503918336  0.6851519013651672
  0.6994912540535133  0.8439302170372714  0.7805577825403313
  0.8520504249558259  0.4464732240357758  0.5812412740380311
  0.8648754835746482  0.3395777589910863  0.6083873137063238
  0.8264548356437222  0.5266963499933570  0.5010463889902846
  0.8525836442998468  0.3129897836186784  0.5569112358146991
  0.8143134466485691  0.5005525770069970  0.4493317743804393
  0.8273039387740693  0.3935800039042823  0.4770804617948390
  0.4900496567260887  0.4839628909021971  0.5709741390780880
  0.3546829917063377  0.6143125270715732  0.5739082228168469
  0.3614146940920631  0.4991652800716375  0.6086717550675432
  0.3056470000527894  0.6850633538101859  0.5169082787846603
  0.3120813301207099  0.4635357280684936  0.5804867788388246
  0.2594532606758931  0.6448830661160824  0.4931894477565003
  0.2620100988028948  0.5343757983055130  0.5252213297557307
  0.8718095311662611  0.4782974126346996  0.6314496796443887
  0.4954528324591415  0.3591303400145401  0.6207712384770414
 -0.0056865947425413  0.5715677082268050  0.5369209650056929
  0.0095539927436447  0.5906680369834525  0.5757610036363171
  0.0662430265095575  0.5385660836293170  0.5020737642973260
 -0.0007278727710629  0.6551056945350663  0.4610944761904019
  0.5843315071922351  0.3536969663197801  0.5984439984859197
  0.4275897782248894  0.2903725359194123  0.7187657491161599
  0.4831943121567418  0.3380029621657412  0.5817505811777054
  0.8657919350980073  0.3974327952384304  0.7095190084485117
  0.8008766628947939  0.5138839974706928  0.6632607343940446
  0.8850791360584809  0.2766441724953977  0.6704830746151887
  0.8178289855602892  0.6111701649192108  0.4775533515058639
  0.8634624797342020  0.2297465878453800  0.5783656283383144
  0.7968672179408132  0.5651237834548061  0.3853590993913576
  0.3891922750627672  0.6444305789237726  0.5939931921223287
  0.3047949815184183  0.4306501715291400  0.7088924913455843
  0.3024883542054532  0.7708744648774188  0.4909188523133274
  0.3146642318160386  0.3777722895163678  0.6063109898811225
  0.2192036860936402  0.7003569657378060  0.4494081132045063
  0.2230238605107687  0.5055378114730881  0.5074340253528271
  0.5536936838500004  0.5515227352715155  0.4725032602011898
  0.5130121641757237  0.5067804643601538  0.6032071050404697
  0.8192359650307400  0.3733479087492884  0.4359029604529076
```

IWV-Al-T6-TS4-Alkylation

```
   Al     Si     O     C     H
   1.00000000000000
    11.6056394577026367    0.0835597589612007    8.7649669647216797
    -3.6825602054595947   11.3529796600341797   10.3282289505004883
```

```
    -0.2346490621566772    0.2412826269865036   19.0520343780517578
   Al   Si   O    C    H
     1    37    76    16    21
Direct
  0.5166789216417107  0.9066495507240855  0.4701145207878122
  0.1486959300623132  0.2783453155884982  0.0166982593166970
  0.1369913345198414  0.1393140478303365  0.9625768914030099
  0.0961900404538203  0.3437119589504118  0.2666625568267162
  0.2450065358533744  0.1676439612687383  0.5329868582724446
  0.1110698683300489  0.1553418273258510  0.7363944022830233
  0.3288584772170673  0.1323437142893907  0.2610420285357482
  0.0561219491933747  0.5825714943292001  0.1218877364361133
  0.0184259590384847  0.8058643403754586  0.0816326193358407
  0.2802944502088455  0.3247484591966666  0.0748753831098278
  0.5616928491085355  0.0722862586458008  0.2181905183681074
  0.7813167167205420  0.0333138534341399  0.0714461266210355
  0.2960947558174766  0.3061428595743801  0.2953048239346240
  0.8589273062377445  0.7769418818432580  0.9235311031608151
  0.8910197799438971  0.9281313824339845  0.9601854435379900
  0.9060918320713552  0.7024815646560419  0.6913930988914758
  0.7686621814649965  0.8713156463030650  0.4142453714373392
  0.9067423753485536  0.9119648995573255  0.1923138553811519
  0.6776151003478408  0.9146602123007761  0.6816790976999068
  0.9423736280054831  0.4603377646612825  0.8340302266616216
  0.0110248886572619  0.2618471425615810  0.8398826319119026
  0.7223358150671958  0.7294446762288060  0.8720814606251294
  0.4447947054886594  0.9778085819663972  0.7177398124238578
  0.2411716996629682  0.0298810337970084  0.8595338829849100
  0.7198313850923257  0.7328676172616528  0.6487695355478744
  0.9016749962099385  0.5275906500090717  0.0834130836202436
  0.0819214229608192  0.5061105968966015  0.8902822067477622
  0.4895486490786595  0.1094912993237798  0.4851684990490732
  0.9036665076132726  0.1208784159975961  0.4278221857565330
  0.8774811919034287  0.1466688673673169  0.2602223072239890
  0.0658811135957537  0.9647357190158212  0.4867249344604084
  0.0933443950959674  0.9364902547756999  0.6490388369692857
  0.4630163411959418  0.5799119920204940  0.8674033097756393
  0.2881955742521133  0.7391000295309007  0.8460470938895268
  0.5170327213295840  0.5092705693542843  0.0345542406676709
  0.6764051346595462  0.3439843276506636  0.0639234406880979
  0.7127135670150627  0.3109918337661306  0.2247129069323409
  0.2476626164825479  0.7653830250604950  0.6895126991970651
  0.1949711236956161  0.2150394349563403  0.9643296690351127
  0.0093337336913650  0.2089119519339359  0.1327967424335904
  0.2306601081739822  0.2702648296949965  0.0511499333551321
  0.1655377059076734  0.4188894401394850  0.9139056279635860
  0.7763734157229910  0.0470343960493773  0.5618964259734049
  0.1886131526858726  0.2158396933087570  0.6000697797172738
  0.1420701201847552  0.0578150082515834  0.6051595101791022
  0.2841801251033647  0.2783368843503275  0.3993584293304177
  0.3548404942167429  0.1225621477456063  0.5332985543339089
  0.0148579327400970  0.8256519199224099  0.5620953575685737
  0.0145214811836379  0.6683258624189923  0.1483815689692560
  0.1819018046951566  0.6572894263603383  0.9882695025279326
  0.0761239259311737  0.4723389925649291  0.2180902324582275
  0.9526732527657190  0.5305909894562897  0.1382944060697634
  0.5965925030936343  0.6252365308599201  0.7472263783144315
  0.6491964239310339  0.0480109874357076  0.1297427340740367
  0.6253429928349090  0.2022339280296258  0.1539504930667056
  0.4409550925483304  0.0815383285625345  0.2274963785601003
  0.5339350692244023  0.9689631511262258  0.3442997866565968
```

0.6030747130581872	0.6138226251327805	0.9867897444399163
0.8299354930053259	0.8536785391703438	0.9596853214234509
0.9849883638297381	0.8469355030404353	0.7912889798025906
0.7513711454798097	0.7586818845883907	0.9253065151948113
0.8682813925352354	0.6496188344476527	0.0168240498231672
0.2074947436086275	0.0179208405016734	0.3745155828904770
0.8467430740562008	0.8446924264490804	0.3273109381586197
0.8328994312614895	0.0092871470726507	0.3329570923865079
0.7876310477039111	0.7840379940741143	0.5172456321334513
0.6312897525545157	0.8460594443010735	0.4713811492892859
0.9648124483046180	0.2433282002714157	0.3763476904541606
0.0045857161749026	0.3956873794253689	0.7835677725688390
0.8001900592241153	0.3939889132405408	0.9360543540831883
0.9708300937869691	0.5981947567778917	0.7199510046705944
0.9969903836421262	0.4526880198845133	0.8909835575461031
0.3950002034664414	0.4369747415554758	0.1706611519676875
0.3774347957454865	0.0333719057618583	0.7780364009992957
0.3435819825327620	0.8690838970902963	0.7853588575719915
0.5444010863253743	0.9290578775196636	0.7385870753467209
0.5092226679223348	0.0802151606859718	0.5754289808364622
0.3887700675958634	0.4478747882756275	0.9449354909306551
1.0016431720594730	0.5087468105455422	0.9941020304896083
0.5071144121450024	1.0088529937714208	0.4823365789994274
0.7804463515980752	0.4154189397338004	0.1919494060606641
0.0897317399719746	0.2289671742703052	0.8851330201936791
0.3832070937913493	0.7873603253553394	0.5933418886342713
0.1888859314633353	0.0963112070056494	0.7848582077713868
0.1657159639239282	0.6395887647730919	0.7602309539261141
0.9597999144269128	0.8489708041772482	0.0157325034589664
0.5819227047217967	0.2423954707430436	0.3535711799078313
0.8136505860111755	0.9662965899981075	0.1625029240048095
0.0275263348687610	0.0211134570880859	0.0966998047776895
0.8835871380387017	0.1570368160630677	0.3368713229196202
0.7897330886912819	0.2181176884951485	0.2295263443277895
0.9885162252028619	0.0542615322709803	0.8233956693456332
0.0447143793229276	0.9720259207229156	0.5765588276025525
0.1957789549212530	0.8716889866759076	0.6250354006869536
0.1560389190815538	0.8920937824414582	0.9812301452617073
0.3922777743272776	0.6745134300394331	0.8270391075579880
0.2288363849540545	0.7545579760860041	0.7893769724031717
0.8787864393989759	0.1660906780432513	0.9485058832683887
0.5865364616338534	0.4135530349306072	0.0342585739612893
0.6999991968199183	0.3731151207738695	0.1207654003883676
0.2414387878539563	0.1013783112565885	0.8939544424191914
0.0800584969321063	0.2636017135948501	0.7329664616381741
0.9412473448587204	0.8135968375842870	0.1820163523971078
0.7884731687212158	0.9565927784943228	0.0397539511089509
0.9940039432176631	0.0393570978313737	0.4338515544232168
0.4788287034222443	0.5734753577665714	0.9486061622630212
0.1718378555123191	0.3493180976149420	0.1561718496261773
0.3359706923117523	0.2002556881724119	0.2962588665356907
0.8371164625510675	0.7008599744008905	0.7984669410529568
0.6896826272367375	0.8399902986393598	0.6470836758039900
0.1687442931018792	0.3100578089232429	0.3179853519516707
0.3303106501793719	0.2280546880639709	0.1456372137348589
0.8084581192786822	0.6791788503918336	0.6851519013651672
0.6994912540535133	0.8439302170372714	0.7805577825403313
0.8520504249558259	0.4464732240357758	0.5812412740380311
0.8648754835746482	0.3395777589910863	0.6083873137063238
0.8264548356437222	0.5266963499933570	0.5010463889902846
0.8525836442998468	0.3129897836186784	0.5569112358146991

```
  0.8143134466485691  0.5005525770069970  0.4493317743804393
  0.8273039387740693  0.3935800039042823  0.4770804617948390
  0.4900496567260887  0.4839628909021971  0.5709741390780880
  0.3546829917063377  0.6143125270715732  0.5739082228168469
  0.3614146940920631  0.4991652800716375  0.6086717550675432
  0.3056470000527894  0.6850633538101859  0.5169082787846603
  0.3120813301207099  0.4635357280684936  0.5804867788388246
  0.2594532606758931  0.6448830661160824  0.4931894477565003
  0.2620100988028948  0.5343757983055130  0.5252213297557307
  0.8718095311662611  0.4782974126346996  0.6314496796443887
  0.4954528324591415  0.3591303400145401  0.6207712384770414
 -0.0056865947425413  0.5715677082268050  0.5369209650056929
  0.0095539927436447  0.5906680369834525  0.5757610036363171
  0.0662430265095575  0.5385660836293170  0.5020737642973260
 -0.0007278727710629  0.6551056945350663  0.4610944761904019
  0.5843315071922351  0.3536969663197801  0.5984439984859197
  0.4275897782248894  0.2903725359194123  0.7187657491161599
  0.4831943121567418  0.3380029621657412  0.5817505811777054
  0.8657919350980073  0.3974327952384304  0.7095190084485117
  0.8008766628947939  0.5138839974706928  0.6632607343940446
  0.8850791360584809  0.2766441724953977  0.6704830746151887
  0.8178289855602892  0.6111701649192108  0.4775533515058639
  0.8634624797342020  0.2297465878453800  0.5783656283383144
  0.7968672179408132  0.5651237834548061  0.3853590993913576
  0.3891922750627672  0.6444305789237726  0.5939931921223287
  0.3047949815184183  0.4306501715291400  0.7088924913455843
  0.3024883542054532  0.7708744648774188  0.4909188523133274
  0.3146642318160386  0.3777722895163678  0.6063109898811225
  0.2192036860936402  0.7003569657378060  0.4494081132045063
  0.2230238605107687  0.5055378114730881  0.5074340253528271
  0.5536936838500004  0.5515227352715155  0.4725032602011898
  0.5130121641757237  0.5067804643601538  0.6032071050404697
  0.8192359650307400  0.3733479087492884  0.4359029604529076
```

IWV-Al-T6-EBH*+EB

```
   Al     Si     O     C     H
   1.00000000000000
    11.6056394577026367    0.0835597589612007    8.7649669647216797
    -3.6825602054595947   11.3529796600341797   10.3282289505004883
    -0.2346490621566772    0.2412826269865036   19.0520343780517578
   Al   Si   O    C    H
     1    37    76    16    21
Direct
  0.5392946263360462  0.9100671118711368  0.4786428970104212
  0.1730922280937492  0.2809157629827211  0.0246124795590446
  0.1613465524337784  0.1426383203314317  0.9706209440868943
  0.1219115006041211  0.3491122751916694  0.2711469636095749
  0.2700799500691020  0.1730614448709429  0.5379201415807439
  0.1367706589889667  0.1590888851862755  0.7430430953890461
  0.3524061072376697  0.1351284358587007  0.2692399455293436
  0.0785658881336113  0.5871737676981756  0.1298940051968379
  0.0403779114438266  0.8099119485976847  0.0900060511451049
  0.3068200490665766  0.3289164359270602  0.0804461330834977
  0.5864207071868823  0.0777542583391584  0.2252721195885755
  0.8081453146703300  0.0424573301313093  0.0746085057900876
  0.3204387613946703  0.3103769588375658  0.3015811115833130
  0.8814611473018329  0.7836392648933969  0.9304649548414579
  0.9140491444154131  0.9336991845380892  0.9675310270073507
  0.9287659588064010  0.7060782233674653  0.6992354425522653
```

0.7893248710895101	0.8758772605102257	0.4219324474790264
0.9328913070997105	0.9183301126092053	0.1979759149788191
0.7028822854933937	0.9197959070450518	0.6886827512666120
0.9679695638515462	0.4646204889965558	0.8399964210858835
0.0368030784944470	0.2672542617507553	0.8454415397915123
0.7456784410174160	0.7337172681600114	0.8797965711499737
0.4695297515276287	0.9818799304314608	0.7244937395245732
0.2644987274100084	0.0326077996412236	0.8672163444647013
0.7443212442405394	0.7387246241269938	0.6546701272257629
0.9250102661729521	0.5343283491141027	0.0897107410725784
0.1044547808843926	0.5091137378455637	0.8993716746062508
0.5144782857903698	0.1141459336437513	0.4914696870178054
0.9284539822607802	0.1246054904374695	0.4335678934852652
0.9005305942577946	0.1517026877679756	0.2660560856839369
0.0895321986888419	0.9684476033735713	0.4928160247126276
0.1151755210531652	0.9422147027160966	0.6551152202214938
0.4873407313396727	0.5836491845481331	0.8734404812059984
0.3110351468395590	0.7421758900673890	0.8543302327528531
0.5421389574140817	0.5140781698087579	0.0394003721907962
0.7020351222026232	0.3500824375681074	0.0683403468821189
0.7376921486387116	0.3186682118505061	0.2286766398158371
0.2689412582619373	0.7694577116223026	0.6983394544970419
0.2202696735945494	0.2188834023319601	0.9712003298463480
0.0346087296290038	0.2078093004010878	0.1428727314478042
0.2560617838182780	0.2740121467331404	0.0578665813378811
0.1862849336336821	0.4210621982999541	0.9253143671289096
0.8032235058710365	0.0510977240607989	0.5686483311499617
0.2111973603652470	0.2183074520803027	0.6075957656854850
0.1683575167251530	0.0643437334350623	0.6071503282767726
0.3093734914488156	0.2848758244870594	0.4042110864923006
0.3802895717200011	0.1279948625435796	0.5374092938996093
0.0381528193744669	0.8290076422853740	0.5691130271176534
0.0358598000085314	0.6718896333479986	0.1577861148085694
0.2042807804990750	0.6636940430427717	0.9958120675280667
0.0996494114541203	0.4767204638818383	0.2252314764830873
0.9757860692201510	0.5351401561492432	0.1457630984922667
0.6203647490886540	0.6327199531269972	0.7511433043223690
0.6742152299112830	0.0535090829418554	0.1365054250147641
0.6487511185880296	0.2079811094001418	0.1607604559695565
0.4652971267884639	0.0848354641397039	0.2352550214511600
0.5602619428401757	0.9740463760703572	0.3504906360384713
0.6265736702108163	0.6181253825279512	0.9934961444937548
0.8510903648827038	0.8597224618298659	0.9677295581683694
0.0054380291036840	0.8574318477476071	0.7964612005858271
0.7731716621778096	0.7610152460892833	0.9351511947047626
0.8953916137891595	0.6585243217633800	0.0210704227414444
0.2317705033373440	0.0200479940883082	0.3817582944169324
0.8675418812126400	0.8498419127151942	0.3345362866249985
0.8480770480551707	0.0159480226929507	0.3389948951999564
0.8123686256300947	0.7933827995366718	0.5218069756003094
0.6508755929005239	0.8443858477425938	0.4830054684320144
0.9917480456042534	0.2480434018301948	0.3788989776318066
0.0344297031723005	0.4023950893607837	0.7873981160468024
0.8260046080262139	0.3960824664077776	0.9424755339939632
0.9946583261791064	0.6026874345395915	0.7271237913561883
0.0200189140543877	0.4573769678495718	0.8986421072472599
0.4202528721929747	0.4401310771644701	0.1756953326954217
0.4003326000485312	0.0337870194608865	0.7881274393689612
0.3709767039293223	0.8718224097540312	0.7909055703312569
0.5702946309813506	0.9353367555540816	0.7438884320970998
0.5323804215214657	0.0852451306740329	0.5825241279577860

0.4181272561027348	0.4497951206673655	0.9501906945390866
0.0244021292717785	0.5145724998692071	1.0011040899492674
0.5322638389196186	0.0123392235843872	0.4899954041783524
0.8022392478972351	0.4244225542880184	0.1966024072036404
0.1037948240531093	0.2263502628051174	0.9031248350533707
0.4040881705302923	0.7933570597380044	0.5997731934495200
0.2133152551652541	0.0942921972244564	0.7921037749515893
0.1895734043740597	0.6424573215131157	0.7683796079772717
0.9823420635101062	0.8534604016832362	0.0233486382594268
0.6080891612020309	0.2460823493560419	0.3590598592943783
0.8448353574608347	0.9794277135393485	0.1618069643837458
0.0564036344596667	0.0213056202559124	0.1067855033585370
0.9039949363346920	0.1579507780527145	0.3462944546182389
0.8196920253882886	0.2305602124113946	0.2283477797043353
0.0105828681214166	0.0605675270334788	0.8316854939038520
0.0674862546568738	0.9767946859519063	0.5822255771329905
0.2142416023251001	0.8730372472981098	0.6358871981035861
0.1780039763160850	0.8952191950557991	0.9910431206687623
0.4114037375570597	0.6733573356833940	0.8377794187003564
0.2510173953718791	0.7588081976215708	0.7975814093891607
0.9016575483098173	0.1759056945943031	0.9490624949232228
0.6135996930869181	0.4202445591947524	0.0365230478455700
0.7239101169113724	0.3802146127826186	0.1253707523101145
0.2646105145124382	0.1073618703387037	0.8984914233577884
0.1105319442908562	0.2671380878043413	0.7397946998511786
0.9619861011815553	0.8180295999788063	0.1902128258113283
0.8104301453076264	0.9607039240778557	0.0489857336917402
0.0195649835691729	0.0435335874629541	0.4377967806080870
0.5040399538063397	0.5789434321104169	0.9532910329335003
0.2007517563619006	0.3578480367553038	0.1584144004003888
0.3587705238369208	0.2026407488909655	0.3049658510097118
0.8607603401968132	0.7059075206095474	0.8052906749757975
0.7141397063353740	0.8443448031112142	0.6551451652546816
0.1933718259886107	0.3149053193529963	0.3236988169834538
0.3543086197743974	0.2306152597317751	0.1536466786515164
0.8311495489817869	0.6828745748416243	0.6930522516750650
0.7232017504867354	0.8489912836261373	0.7886176864155156
0.3761297447970181	0.8224661128717469	0.3388788101091388
0.4979722951127593	0.8262987414853734	0.2861258650538592
0.2941018970323611	0.7459909762151307	0.4609935316278403
0.5395085329090575	0.7582484224651214	0.3531751040591756
0.3319561360239485	0.6707900765583090	0.5361767858923120
0.4606602003717823	0.6814204670484877	0.4771534029409625
0.4995136136535996	0.6134893075620710	0.5530946797735730
0.7879519741518318	0.3840574462780125	0.7460783595372028
0.7862282597686497	0.4898704396388919	0.6552246231960914
0.8823232640703830	0.3378482893451685	0.7133835801019310
0.8762200534694072	0.5481805581375981	0.5349448201061190
0.9726010622750916	0.3955680908185317	0.5934503152700170
0.9695802301348742	0.5009350350294444	0.5037850699228410
0.6889059765219169	0.3185832114869835	0.8763155162586578
0.6297016806677965	0.6134767720329746	0.4920485893872520
0.5950273854571628	0.2154740639199930	0.9388868774527790
0.5252367192845648	0.1672971006052593	0.0341485519423497
0.5520985729326325	0.2490847813048621	0.8964699948556897
0.6376898312259772	0.1522289842480266	0.9321105743774403
0.6471327228553860	0.5593967106406827	0.5572452590019973
0.6511141158959745	0.5762782953122535	0.4534236813915373
0.6912517779570555	0.7040861862094863	0.4208106372551640
0.6450545727207465	0.3812610427231823	0.8834160724728440
0.7276226333262581	0.2828851305402683	0.9228545576321190

```
  0.5631904169635357  0.8860785219837191  0.1888163255964898
  0.1989823795672526  0.7385272963815156  0.5060587443748399
  0.6348911496620080  0.7676828296583372  0.3067692786128313
  0.2783685665344141  0.5761640725307704  0.6038510694993982
  0.3492534909225312  0.8797336350694795  0.2821917060926332
  0.7136098008251991  0.5270351617907892  0.6795129613803581
  0.8847723961321673  0.2554827206369246  0.7831742639393597
  0.8739563759049378  0.6310762129051481  0.4654707167900017
  0.0457135262586676  0.3586197625440358  0.5698469338676968
  0.0396373740463811  0.5460108529238780  0.4100579903016394
  0.4819127578317258  0.6543036184232397  0.5883189146214921
  0.4366897340040262  0.5219868570919541  0.6335769177797195
  0.2988669276118371  0.6840002955943315  0.5971853721223997
```

IWV-Al-T6-TS4*-Alkylation

```
   Al     Si     O     C     H
  1.00000
    14.5438127517700195       0.0000000000000000       0.0000000000000000
     3.3510317802429199      15.4238224029541016       0.0000000000000000
    11.2960309982299805      10.5371932983398438      11.1561899185180664
   Al     Si     O     C     H
   1     37     76     16     21
Selective dynamics
Direct
 0.5443029403686523    0.9180058240890503    0.4760368466377258
 0.1762801557779312    0.2909478545188904    0.0208317786455154
 0.1706997156143188    0.1498928666114807    0.9666158556938171
 0.1248207986354828    0.3538650274276733    0.2742195725440979
 0.2740688621997833    0.1784422397613525    0.5371682643890381
 0.1417022049427032    0.1671031117439270    0.7405409216880798
 0.3560179471969604    0.1405259519815445    0.2686335742473602
 0.0817320495843887    0.5924241542816162    0.1313311457633972
 0.0487423427402973    0.8188499212265015    0.0846683159470558
 0.3073491156101227    0.3337563574314117    0.0824532583355904
 0.5910552740097046    0.0849535465240479    0.2226668894290924
 0.8215403556823730    0.0536497272551060    0.0680298656225204
 0.3261185884475708    0.3171546757221222    0.3003382682800293
 0.8856323957443237    0.7904936075210571    0.9284378290176392
 0.9232563972473145    0.9393888115882874    0.9637979865074158
 0.9324227571487427    0.7104687690734863    0.6984896659851074
 0.7988272905349731    0.8847804069519043    0.4174977838993073
 0.9420366883277893    0.9282556772232056    0.1930999755859375
 0.7057627439498901    0.9232960939407349    0.6869304180145264
 0.9694676399230957    0.4665693640708923    0.8436638116836548
 0.0443643331527710    0.2747985720634460    0.8417069911956787
 0.7517647147178650    0.7385706305503845    0.8774030208587646
 0.4736830294132233    0.9858230352401733    0.7222688198089600
 0.2723031640052795    0.0419045686721802    0.8623496294021606
 0.7460393905639648    0.7427366971969604    0.6541470885276794
 0.9297886490821838    0.5418166518211365    0.0882423147559166
 0.1093114018440247    0.5185281038284302    0.8963861465454102
 0.5187031626701355    0.1222615838050842    0.4860710203647614
 0.9361907839775085    0.1283743083477020    0.4329753220081329
 0.9068542122840881    0.1590716242790222    0.2650592625141144
 0.0958278179168701    0.9701359272003174    0.4927268028259277
 0.1204358339309692    0.9484027624130249    0.6538156270980835
 0.4921567440032959    0.5862576365470886    0.8738053441047668
 0.3141894936561584    0.7437591552734375    0.8573022484779358
 0.5503634214401245    0.5151476263999939    0.0393583141267300
```

0.7059605121612549	0.3467183709144592	0.0753108710050583
0.7403986454010010	0.3234951496124268	0.2319042533636093
0.2743264734745026	0.7752857804298401	0.6980617046356201
0.2258713245391846	0.2285242080688477	0.9674988985061646
0.0358706638216972	0.2215119898319244	0.1351031213998795
0.2535230517387390	0.2802085280418396	0.0607235580682755
0.1921726465225220	0.4311473965644836	0.9203819036483765
0.8085321187973022	0.0531075298786163	0.5667276382446289
0.2162846028804779	0.2247243225574493	0.6057212948799133
0.1708229184150696	0.0730974972248077	0.6038635969161987
0.3185381889343262	0.2911583185195923	0.4015667140483856
0.3814979493618011	0.1290664374828339	0.5407062172889709
0.0436048805713654	0.8314502239227295	0.5680316090583801
0.0440858900547028	0.6803843975067139	0.1553533971309662
0.2092922329902649	0.6636964082717896	0.9993940591812134
0.0971135199069977	0.4792841374874115	0.2305222749710083
0.9786146283149719	0.5456473827362061	0.1441510766744614
0.6233831048011780	0.6353968977928162	0.7502030730247498
0.6922006607055664	0.0769845098257065	0.1245697960257530
0.6520648598670959	0.2043119966983795	0.1733346581459045
0.4723516702651978	0.0950617194175720	0.2305987030267715
0.5470665097236633	0.9643903970718384	0.3573446869850159
0.6363734006881714	0.6190095543861389	0.9922837615013123
0.8563073277473450	0.8664002418518066	0.9659659266471863
0.0112747848033905	0.8635225296020508	0.7954324483871460
0.7785767912864685	0.7707930207252502	0.9300832152366638
0.8966214656829834	0.6635695099830627	0.0203567463904619
0.2387342751026154	0.0224305391311646	0.3822994232177734
0.8760266900062561	0.8590374588966370	0.3297473788261414
0.8621544837951660	0.0216855704784393	0.3380681872367859
0.8145423531532288	0.7948998212814331	0.5222060680389404
0.6621648669242859	0.8633284568786621	0.4719710648059845
0.9973246455192566	0.2485061287879944	0.3845787942409515
0.0407128632068634	0.4073772430419922	0.7887886166572571
0.8290483951568604	0.3882468342781067	0.9510762691497803
0.9895236492156982	0.6013430953025818	0.7327942848205566
0.0200663208961487	0.4654835462570190	0.9006791710853577
0.4270766079425812	0.4457492232322693	0.1738140285015106
0.4071687757968903	0.0443976521492004	0.7793024778366089
0.3726266622543335	0.8740036487579346	0.7939865589141846
0.5744773149490356	0.9416360259056091	0.7420070767402649
0.5384803414344788	0.0816341042518616	0.5801381468772888
0.4197144508361816	0.4532150626182556	0.9517990350723267
0.0311656594276428	0.5225638747215271	0.9995025396347046
0.5423942208290100	0.0347598195075989	0.4661450386047363
0.8087366223335266	0.4296185970306396	0.1964397877454758
0.1092192530632019	0.2276603579521179	0.9023121595382690
0.4111629724502563	0.8025324344635010	0.6005571484565735
0.2171028256416321	0.1030552983283997	0.7910293340682983
0.1938384175300598	0.6510971784591675	0.7654248476028442
0.9940835237503052	0.8593478202819824	0.0161527767777443
0.6086673736572266	0.2582622170448303	0.3593468666076660
0.8555210232734680	0.9951527714729309	0.1561390310525894
0.0676057934761047	0.0274432152509689	0.1036962121725082
0.9187453389167786	0.1695815324783325	0.3387261927127838
0.8152788877487183	0.2285222113132477	0.2403598725795746
0.0159379839897156	0.0684477686882019	0.8290740251541138
0.0725402235984802	0.9782617092132568	0.5826706886291504
0.2226873636245728	0.8824248909950256	0.6341332197189331
0.1861795783042908	0.9042226672172546	0.9870064258575439
0.4167879819869995	0.6774389743804932	0.8394544124603271

0.2533570826053619	0.7592945694923401	0.8016983270645142
0.9098008871078491	0.1863319873809814	0.9414180517196655
0.6170897483825684	0.4158478379249573	0.0432938635349274
0.7293893098831177	0.3821701705455780	0.1276447325944901
0.2751963734626770	0.1155691146850586	0.8939039707183838
0.1162290871143341	0.2764179110527039	0.7353287935256958
0.9667782187461853	0.8304966688156128	0.1824040412902832
0.8267418146133423	0.9673264026641846	0.0458991155028343
0.0268186032772064	0.0468075275421143	0.4367233216762543
0.5145716071128845	0.5796353220939636	0.9515106081962585
0.2040352672338486	0.3633591532707214	0.1608556509017944
0.3613421022891998	0.2085068523883820	0.3042865991592407
0.8687936067581177	0.7133520245552063	0.8020478487014771
0.7130378484725952	0.8493312597274780	0.6529653668403625
0.1987276673316956	0.3243592679500580	0.3233557641506195
0.3550537526607513	0.2339072376489639	0.1547609865665436
0.8320278525352478	0.6900864839553833	0.6935510635375977
0.7247890233993530	0.8494499921798706	0.7881255149841309
0.3936741650104523	0.8686600923538208	0.2796601057052612
0.5036463737487793	0.8448279500007629	0.2320480495691299
0.3373262882232666	0.8355261683464050	0.3872027695178986
0.5532693862915039	0.7841561436653137	0.2915531098842621
0.3936884701251984	0.7858861684799194	0.4434820711612701
0.4967344105243683	0.7475774884223938	0.4014478027820587
0.5408926010131836	0.6774691343307495	0.4735441803932190
0.7890502214431763	0.3879212737083435	0.7467205524444580
0.7884500026702881	0.4938721954822540	0.6550446748733521
0.8845057487487793	0.3423652052879333	0.7150002717971802
0.8807159662246704	0.5530807971954346	0.5348324179649353
0.9770467281341553	0.4010100364685059	0.5951359868049622
0.9752255678176880	0.5065794587135315	0.5046049952507019
0.6878315210342407	0.3217762112617493	0.8768098354339600
0.6602151393890381	0.6560480594635010	0.4150894284248352
0.5955778956413269	0.2189444303512573	0.9372529387474060
0.5239089727401733	0.1704815328121185	0.0323855653405190
0.5547908544540405	0.2529038786888123	0.8928561806678772
0.6390735507011414	0.1556690335273743	0.9310389161109924
0.6861077547073364	0.6073470711708069	0.4734925031661987
0.6552211642265320	0.6031595468521118	0.3983957171440125
0.7321254611015320	0.7405580878257751	0.3291724324226379
0.6428377032279968	0.3841758370399475	0.8836895823478699
0.7247346043586731	0.2858162522315979	0.9249618053436279
0.5510348081588745	0.8726086616516113	0.1458166390657425
0.2506172955036163	0.8468752503395081	0.4311253130435944
0.6356508135795593	0.7642375826835632	0.2522507011890411
0.3360923230648041	0.7394442558288574	0.5382843017578125
0.3638693988323212	0.9092853069305420	0.2256231755018234
0.7148857116699219	0.5304045677185059	0.6785827875137329
0.8862589597702026	0.2600670456886292	0.7853923439979553
0.8792231082916260	0.6360942125320435	0.4647510945796967
0.0511500537395477	0.3647814989089966	0.5722419023513794
0.0472640991210938	0.5524374842643738	0.4109230935573578
0.5447822809219360	0.7213796973228455	0.4990162253379822
0.4695099592208862	0.5915843248367310	0.5597560405731201
0.4606355428695679	0.8735027313232422	0.4019977152347565

IWV-Al-T6-2EB

```
Al    Si    O    C    H
1.00000000000000
```

```
    11.6056394577026367    0.0835597589612007    8.7649669647216797
    -3.6825602054595947   11.3529796600341797   10.3282289505004883
    -0.2346490621566772    0.2412826269865036   19.0520343780517578
   Al   Si   O    C    H
     1    37    76    16    21
Direct
  0.5430849813292965  0.9178713871362898  0.4789781864364224
  0.1676941748005008  0.2724791434401269  0.0363232540771790
  0.1432062574168247  0.1237966049676350  0.9940362602911266
  0.1111372260213523  0.3339234837435673  0.2853465981900822
  0.2577753276845867  0.1653626399130889  0.5537431380028623
  0.1273859323375520  0.1478332313598445  0.7582891874247790
  0.3473031867944825  0.1267346787676361  0.2792692176521970
  0.0742603540333337  0.5735087217909057  0.1405988926915263
  0.0340331321095434  0.7960716199640911  0.1023755180150566
  0.2995775547492583  0.3192623473041937  0.0922344550083868
  0.5813603042717467  0.0654409975786673  0.2340140629502965
  0.7956838775486099  0.0212329607937114  0.0918142576027094
  0.3097523633714312  0.2971798640073954  0.3161679061535151
  0.8798315333290917  0.7673428254282403  0.9406529233363113
  0.9087901477862991  0.9175877701147744  0.9802140760000585
  0.9264041999145727  0.6956446514003297  0.7084734213265613
  0.7913162872203170  0.8674359140279676  0.4295527245078816
  0.9197854223444943  0.8975626001358600  0.2160418368389940
  0.6929356182163235  0.9036819557467828  0.7048211401706690
  0.9619963764732045  0.4567809740255437  0.8505984888327189
  0.0188883426843181  0.2484796236583351  0.8692917079035476
  0.7396391776867384  0.7165371362774399  0.8939977086807739
  0.4592327465399929  0.9695541039715343  0.7427565772370821
  0.2555779572674772  0.0232291981221051  0.8804894382792032
  0.7396719733115101  0.7266521392124260  0.6662070436363996
  0.9225492543143960  0.5190815948612658  0.1004110565872965
  0.1045749290421130  0.4995796616069849  0.9061620627072720
  0.5004753732859444  0.1065446986057514  0.5100091787242800
  0.9165204530854358  0.1099191512194800  0.4522735908748921
  0.8965724757814763  0.1369043118855985  0.2811595276256170
  0.0854975438399215  0.9594370054988662  0.5038612110804250
  0.1148823947413724  0.9298031447596932  0.6665107861702007
  0.4847437562860636  0.5745933974066644  0.8850772546482977
  0.3095257571608605  0.7324830792206479  0.8650324341790211
  0.5334150478786227  0.4982169808171779  0.0575167205351125
  0.6947940804021459  0.3359258141822894  0.0822612950151175
  0.7311282845291767  0.2966024955891347  0.2470537116674690
  0.2640893380845635  0.7582704376173555  0.7092846442807317
  0.2001088545748561  0.1975360615195937  0.9980520367162482
  0.0294816723617408  0.2157586090058262  0.1536202614066409
  0.2566710275569369  0.2657996629716039  0.0637353266274139
  0.1870409507551175  0.4123472812701793  0.9303495129367101
  0.7930670779092246  0.0352048522316238  0.5887106010579244
  0.1950449508613053  0.2117136231906080  0.6222286299581532
  0.1630604590981411  0.0469124767713435  0.6283667594495463
  0.2928535651934964  0.2711896607540058  0.4214923283635520
  0.3722198172144257  0.1324069109904104  0.5498562128291373
  0.0322663362007737  0.8206871376316767  0.5813040889272019
  0.0368375068840746  0.6604632282090658  0.1651235056850263
  0.1989277068331622  0.6453443070362778  1.0061835551171074
  0.0945807703078540  0.4636164054908446  0.2365744871863225
  0.9699968640896487  0.5222455737445892  0.1575073557222207
  0.6166735473340023  0.6184287883767250  0.7643121392394301
  0.6597683259076703  0.0266604542335956  0.1580709332854812
  0.6565167925647668  0.1958012926723496  0.1592729665035851
```

0.4603525100518275	0.0774445957671561	0.2449897217188665
0.5539832704619341	0.9694754854255481	0.3610600040097323
0.6223643400669501	0.5983120807600312	1.0123319342446788
0.8529885663324042	0.8468669245009572	0.9731645424827489
1.0083795194718246	0.8327578070188709	0.8096666247069604
0.7728511909753990	0.7515215570697169	0.9410055464679321
0.8838787695191748	0.6381342154225804	0.0375670377163261
0.2260688908016598	0.0136559791253309	0.3905807936845170
0.8494505923441845	0.8283158848332967	0.3541793417038881
0.8724722996614613	0.0034613076961410	0.3361165198152107
0.8068148300477945	0.7781927189168832	0.5345159799450088
0.6531530168484002	0.8574065578966876	0.4873863538889545
0.9781238394728955	0.2370401405088957	0.3932168665974971
0.0167879536713211	0.3836734421924277	0.8098614199411367
0.8183967876623324	0.4001858790982770	0.9498061064363950
0.9941080181072932	0.5939686466012064	0.7337968402469484
0.0163305485837458	0.4499247793452445	0.9076748465610921
0.4075224564635746	0.4289074623904948	0.1910730899179890
0.3925510889015086	0.0229618957687424	0.8049335807766630
0.3570866151709864	0.8609789274973539	0.8106125973756412
0.5602126983017743	0.9192493790722827	0.7606238443045883
0.5217759916653812	0.0719274257885479	0.6008060720845925
0.4066412644591919	0.4442605876179184	0.9635628353276799
0.0289719630004704	0.5102079723270611	1.0041542928087903
0.5014817067454733	1.0005012405531568	0.5156242041917670
0.8072137448118559	0.4018619080768352	0.2068545597389198
0.0794828558210223	0.2054755129717566	0.9331911077765713
0.4077400266579948	0.7820312238260382	0.6064003924332706
0.2103839898714243	0.0850433946122472	0.8001368010557308
0.1933521656536752	0.6328749265796245	0.7736628404981005
0.9775418688549889	0.8367700162176500	0.0352750249489830
0.6026255682614128	0.2323142156909661	0.3764676834722588
0.8352538552549460	0.9621909930791669	0.1763393225694043
0.0435081891824285	-0.0016751609565650	0.1286638436809209
0.8858413114972706	0.1354211532133635	0.3717562392545777
0.8011642405324415	0.1974470914831499	0.2570719361285225
0.0041692576728024	0.0464154763304630	0.8467758973211815
0.0684829988866523	0.9674623310840758	0.5922255988626168
0.2213778510173337	0.8677082382953045	0.6384985410791391
0.1689254117867201	0.8861669314778040	1.0040301123401734
0.4165645464951977	0.6721999275386310	0.8430272989079539
0.2597303363326025	0.7526787969513865	0.7987654807833627
0.8849276124073778	0.1574735701911262	0.9674970087130441
0.5972378003681414	0.3978590824401741	0.0623990625171931
0.7126979055364652	0.3560387812704157	0.1476072605709980
0.2504331799641917	0.0984147724365349	0.9130675146517999
0.0992180550891561	0.2514152942104880	0.7612708926373255
0.9509189802702028	0.7989250712423611	0.2062640950089750
0.8031399747046726	0.9410083062488895	0.0636218404140477
0.0116664939631486	0.0338158522479649	0.4534751324506305
0.5031089924041328	0.5681362572047060	0.9647684643616771
0.1863714331789708	0.3405948992674256	0.1742074866080085
0.3535452680185694	0.1915346314276146	0.3178116028483696
0.8547434775837714	0.6899700997068413	0.8185607588872825
0.7075559604476864	0.8334367753386539	0.6650205047966933
0.1832040588453935	0.2970900799385343	0.3377708848610446
0.3510638261438576	0.2261391149190633	0.1626018108561170
0.8287259159448135	0.6733979216780870	0.7015749249754334
0.7105691307701305	0.8270902042735733	0.8069059933819838
0.4981170968464156	0.4660556891396543	0.6919827851244892
0.4151069526211064	0.4195528697290261	0.7015135954060540

```
  0.5552539846757137  0.5895458088611492  0.5991989672805637
  0.3863125311430183  0.4937610130873902  0.6237807309538326
  0.5294405032681190  0.6642980411267573  0.5187675566498197
  0.4432163336720427  0.6167077139847911  0.5320217119273730
  0.1736513145291110  0.6315608969508787  0.4858857068936759
  0.3524079582532418  0.8165538285809615  0.2991927410195695
  0.2293383551305438  0.7629609760042402  0.3724889043210176
  0.4074226999685231  0.9376272949030866  0.1946614681752358
  0.1625964496026799  0.8347734509142741  0.3373894314008908
  0.3396963225392577  1.0082057599117384  0.1607160960488732
  0.2173230810997381  0.9561789927275691  0.2324869912320240
  0.5290619363611635  0.3859058654063573  0.7758992431513196
  0.0494595278013397  0.5737197011966918  0.5425661430948097
  0.6509225354865378  0.3666993441734502  0.7167044870741273
  0.6718397218063757  0.3093322069699401  0.7793221798770418
  0.6507975324415155  0.3241743879809093  0.6888242187515141
  0.7230310270361825  0.4522606413571088  0.6366676749571545
  0.0185758492845619  0.4780728298626353  0.6217213202862036
 -0.0170003706157165  0.6136176323774925  0.5716458313245518
  0.0509066003922379  0.5840860398844251  0.4787754066247654
  0.4583478065272057  0.2992984741474368  0.8564544688358198
  0.5310422965464506  0.4256370404228089  0.8055435634420838
  0.3724073871223926  0.3233739811967681  0.7715926739468126
  0.6211327030095913  0.6277342216919218  0.5891195263290396
  0.3201715526532243  0.4554763366886284  0.6341416818674772
  0.5794609425698155  0.7592884940400473  0.4430890985293561
  0.4061668959898569  0.7615712981009237  0.3243639792051747
  0.4245774554378392  0.7206908574064057  0.6013305323463166
  0.5029776899525736  0.9768193250507068  0.1401481266101399
  0.0669842639023342  0.7958707247214617  0.3918254009079733
  0.3817586584704413  1.1028779524606347  0.0790908766040445
  0.1636807917363810  1.0098810694469875  0.2065572715963654
  0.2356045087331142  0.5835959721903337  0.4659962213451008
  0.1682429845669372  0.6196819695127662  0.5523596089414968
  0.4221589259653988  0.6752173848008922  0.4702555243958930
```

S4.3.- MOR-Al-T4-Transalkylation

MOR-Al-T4-DEB+Benzene

```
   Al     Si     O     C     H
   1.00000000000000
    18.1842269897460938   -0.0090872207656503   -0.0001607598969713
    -0.0105508984997869   20.0691013336181641    0.0015994716668501
    -0.0001797722798074    0.0011982563883066   14.8440780639648438
   Al   Si   O    C    H
     1    95   192    16    21
Direct
  0.5930503175130646  0.7169023424072587  0.6441463811860871
  0.3154994373109457  0.0745970776419381  0.0386261210195599
  0.3032484683284834  0.3199518553225051  0.0406389491367399
  0.8129506384504959  0.5761308288169555  0.0342258895391711
  0.8100171371427501  0.8006900395174923  0.0366537913366347
  0.6919976775023163  0.9275247187229787  0.2847215177914252
  0.7081901365339752  0.6829949976172012  0.2851142573837765
  0.1956080838682524  0.4240032933759941  0.2891661207411383
  0.1946464790231779  0.2001986300075563  0.2867939511159615
  0.6936622369001481  0.0779373881839937  0.2365423484857437
  0.6939100143411647  0.3034040136227190  0.2373337269015230
```

0.1931787476758678	0.5735135144595913	0.2428973629355797
0.2029775176181494	0.8203871364084205	0.2400260182956049
0.3103196334310944	0.9249472228707851	0.4917825481567877
0.3129937019059946	0.7013538007027741	0.4917315550795034
0.8154110845656997	0.4298066444848554	0.4883465421659254
0.8027709773398873	0.1844266152034911	0.4847684554423250
0.7081435059483232	0.9196600979762206	0.4924886542826636
0.6958671692979860	0.6833838812671438	0.4938611195985987
0.1857699712252995	0.4370222898194008	0.4942248299375238
0.2092752095597012	0.1908237672442879	0.4938931000124594
0.2983779709500702	0.0852853412971648	0.2462642048395671
0.3111536511633508	0.3161971997776248	0.2480263551363069
0.8216085518409499	0.5672111489303830	0.2367713882627862
0.7997615996191421	0.8143213810162736	0.2418827731951925
0.3206625488527096	0.9358358221616069	0.2864233754406065
0.2969670959086331	0.6904762930515159	0.2854235395037464
0.7979055644148919	0.4201821383817473	0.2800988834054038
0.8093858904656752	0.1859977689659716	0.2767514973532515
0.6881500836481020	0.0666392634327818	0.0315822552338682
0.7091144505705026	0.3137165372173063	0.0312820022908033
0.2096472407792526	0.5850843842631489	0.0347880227091604
0.1954789208513502	0.8171045889846538	0.0326383805470886
0.0857555782952630	0.3795292778181525	0.1356251716074010
0.0891497733612363	0.2246033613154914	0.1305863964434672
0.5891889330429485	0.8751545166184087	0.1413523290857871
0.5862416158906807	0.7154520710935560	0.1482333296276556
0.9206617678941902	0.6249931751719732	0.3837419286022353
0.9170698269182546	0.7799393091590736	0.3820064555819994
0.4170613205338874	0.1282886030526593	0.3952669410710604
0.4202194204010053	0.2861393081761783	0.3991804013395448
0.9172670420251152	0.3776694851307316	0.1314296409136507
0.9198657885388042	0.2188622021699007	0.1267891417218830
0.4212158679636397	0.8782093658280001	0.1432230471011596
0.4182016706113428	0.7236303223301636	0.1485552451316736
0.0888359966971186	0.6272999748477767	0.3830038517517327
0.0865653846482670	0.7844491750262549	0.3803969301303039
0.5854803469052270	0.1254703827161550	0.3888816476804094
0.5892235795442342	0.2808458519885482	0.3944473558709339
0.3161122666146505	0.0755017512212947	0.5383673681808988
0.3031812524709600	0.3213918144464984	0.5405413365539142
0.8130965671542431	0.5790053777564129	0.5386058400974909
0.8081667807343674	0.8016208163756859	0.5364887038407592
0.6913115979570583	0.9291044602287059	0.7850794450837675
0.7087360313311583	0.6819496398316928	0.7925144352820326
0.1948030535837204	0.4245526006708453	0.7878415529401058
0.1946187031988675	0.2001417483833453	0.7866846559453966
0.6947118839005940	0.0777336018077268	0.7361163156814629
0.6934862789482374	0.3040905000941890	0.7376566349585044
0.1904277369965186	0.5741703687690773	0.7420324928832738
0.2014322458660964	0.8202221467874119	0.7404029930806999
0.3106497654326780	0.9246019031361959	0.9919355248528144
0.3126972508032115	0.7015759498160496	0.9934389682808337
0.8162514902542918	0.4274482219127014	0.9863668217266947
0.8034196314271626	0.1837259328255632	0.9848465902323615
0.7077742980204375	0.9179197408970693	0.9924096818488944
0.6952141471651133	0.6820770642948385	1.0004584992070105
0.1877758921099404	0.4366958970095938	0.9934379018853784
0.2095362402851605	0.1898631748530815	0.9938863746757933
0.2991480289076140	0.0851041294958451	0.7459477157680388
0.3109986330202679	0.3166230298235237	0.7476649950050326
0.8252870240942105	0.5666856408670775	0.7436931687768313

0.7975418128461861	0.8125471875718288	0.7440870956578152
0.3196501931813936	0.9351900068443479	0.7864692750863458
0.2941838502374366	0.6899686153977337	0.7878850768372281
0.7985210279498595	0.4178539944508239	0.7800621860225480
0.8089567569426079	0.1873754181077011	0.7769866248332955
0.6873873075249021	0.0673290063139979	0.5305441450195836
0.7097303777224386	0.3143911066843538	0.5314885643023645
0.2056765293752492	0.5871907886868053	0.5339231077897696
0.1963367539480811	0.8173260528810125	0.5325848199530520
0.0844881369471818	0.3782772114130635	0.6367217253739610
0.0882985599449385	0.2231260875236933	0.6311922437083111
0.5882278037763400	0.8792799949935954	0.6410478334163017
0.9233178832369533	0.6238101936106955	0.8843445639103082
0.9173102204855851	0.7777058366733999	0.8781513468238022
0.4173582047079495	0.1272503552245183	0.8959576343369744
0.4200946615684116	0.2851705390076399	0.8998685749490388
0.9169934003841168	0.3762834822056050	0.6302473928300029
0.9196419967841583	0.2183152314088312	0.6261179243831844
0.4221565363710685	0.8786540124764953	0.6416865671363947
0.4146127128094051	0.7223103105958175	0.6485068482558668
0.0903968276953848	0.6268486620512433	0.8858319636849350
0.0861360125870758	0.7845647772114068	0.8817328675109324
0.5861665728551975	0.1248816606419606	0.8882611504651371
0.5891493465205296	0.2804485066453333	0.8935126536447774
0.1348003279375243	0.3875376809121165	0.2263343082269951
0.1184422913444048	0.2157974873853086	0.2337462250120057
0.2531526925096013	0.1203341472590672	0.4889682699192990
0.6144804212379917	0.9146739753051258	0.2321801357118217
0.6325695727105736	0.6792660855383333	0.2274178367226618
0.7391858395329215	0.6235109000833684	0.5469923994045395
0.8726345310909726	0.6164759742637798	0.4748191510868124
0.8852840733035106	0.7876334095310182	0.4839644963850754
0.7565146763699020	0.8850754413298256	0.2345051832753532
0.3933378857951870	0.0856369738017486	0.4842495393341997
0.3741101184269015	0.3269839714292848	0.4747664415563956
0.2698716656751396	0.3791628077015338	0.2951434335470724
0.8936671934499334	0.4179491602087881	0.0404785934633163
0.8763006502327764	0.1794969553163472	0.0476280805779275
0.7689263844680528	0.1217626453248683	0.2317758961161176
0.3699583388824280	0.8855518591032298	0.0542177746676527
0.3897664765211574	0.7165450959324644	0.0447086323998731
0.2555260301891711	0.6188566702735672	0.2928406323246792
0.1160261716105897	0.5829205259640607	0.2971693667947654
0.1317424358354081	0.8267962149661846	0.3052235103331207
0.2350160975564958	0.8813797843058044	0.9863351723515427
0.6331416761467249	0.1167624952821890	0.2973038198860805
0.6170845608165487	0.2906088458588149	0.2907845075452836
0.7530563839658960	0.3841520222159429	0.0381226037054288
0.8922775356797342	0.5763046650034930	0.3032521038834069
0.8681556601310191	0.8231042231538492	0.3114405207606706
0.7822627438642560	0.8755123488812643	0.0116108459583429
0.3633364630523023	0.1126159994448287	0.3110277829584169
0.3885373788357190	0.3031060072232317	0.2992269206934545
0.2444702403969088	0.3767288615398575	0.0132857148541095
0.1151489863957810	0.4293220630121176	0.0568308196587037
0.1401286131257476	0.1819459683386072	0.0617679622356027
0.2216576176113692	0.1246326299908893	0.2649234152754081
0.6409789473811053	0.8942054807363637	0.0569275041297012
0.6161258804745544	0.6915113068942723	0.0499871706187815
0.7666671517572787	0.6297886448169991	0.2458742295631223
0.1396535912648530	0.6146283199939400	0.4710340251075094

0.1212251983197120	0.7985901407279199	0.4797034150509374
0.2628895638138009	0.8766836791161480	0.2655316803502603
0.6143090932912779	0.0759505276241401	0.4679314435365227
0.6410776875710135	0.3231756235770958	0.4626038521646232
0.7230943895249725	0.3783617346983590	0.2594793406096961
0.8646026330106066	0.3960834267538969	0.2155506897807438
0.8863718140110191	0.1992514327234040	0.2247731687247474
0.7429246751981662	0.1287865919942741	0.5127898737349038
0.3933788861893305	0.9275098137177784	0.2233706067525328
0.3647375001822518	0.6819000260359128	0.2156110009368719
0.2817038812273149	0.6273271482327598	0.5136304566634435
0.2590851047825728	0.2506673123242978	0.2535403731601000
0.7436874458349026	0.7492476902286350	0.0140367493743691
0.7646063835183730	0.2562110813340394	0.9954707751109452
0.2400166496517278	0.7469374614604150	0.2501770184658569
0.7432957495601630	0.7575041826938106	0.2784640441697353
0.2654543738706195	0.2468846815840383	0.0312131878152101
0.2495473300744618	0.7528715211450934	0.0268971492656433
0.7563848885814157	0.2509248084262641	0.2696693195206353
0.0009875269519376	0.3979591673388287	0.1582538368464936
0.0056320669306930	0.1970715009982433	0.1243942339555437
0.5053134828261036	0.8968878688159584	0.1172550569812610
0.5004742614412722	0.6936931815618098	0.1560107051159719
0.0052893467546679	0.6059468305162610	0.4072690934079309
0.0011921768545031	0.8073023467103830	0.3800658030247848
0.5001727001401144	0.1079941271808289	0.3668835577658861
0.5057546207004692	0.3082554360241422	0.4031141446887588
0.0928057021319411	0.3028337072420346	0.1008031499132142
0.5941920296644884	0.7956079473628151	0.1594266934713018
0.9146277665175391	0.7020635672251601	0.3502462043825041
0.4116971534039569	0.2069742107169926	0.4200336549584993
0.9114308461062778	0.2985009140386211	0.1102570469082973
0.4159652896989035	0.8016208822433391	0.1791193752655565
0.0937411885508642	0.7056446770431376	0.3558923730900320
0.5932604329678679	0.2024153378378830	0.4228248046920268
0.1793391047981911	0.2098124752357281	0.3938933316238263
0.1607442357636012	0.4325519844211690	0.3892703816351872
0.6899107660841245	0.6634493567535499	0.3880401334267887
0.6838857601139743	0.9046080232063357	0.3888312559446886
0.8314498959784525	0.7950882383816549	0.1428032831516516
0.8499779157776569	0.5633393394765195	0.1327633858093243
0.3298096461564193	0.3321820551635900	0.1435761441038637
0.3250313130138520	0.0973966960436479	0.1428260118704770
0.8286095127300540	0.1723087773225699	0.3816094943173389
0.8238528991222284	0.4076481892273440	0.3837157079683378
0.3283120288218070	0.7106068763000694	0.3847240400313394
0.3455214781799712	0.9326329623727451	0.3916508012981962
0.1759531362002054	0.8316177268214039	0.1371838339509258
0.1832275210757207	0.5957850473054453	0.1385528251604355
0.6773723736607927	0.2945791904782056	0.1304464167805947
0.6602874862547587	0.0688317428943892	0.1357176214250096
0.2184453097248650	0.4959152212294896	0.2467414663343881
0.7131496424623486	0.0061484983712276	0.2807699224504593
0.7927940134568687	0.5054364784708553	0.9867520793330482
0.2913227704799264	0.9967106564604942	0.0355606764851343
0.7790700774512348	0.4983965057700606	0.2631234024573744
0.2818890139437608	0.0069930775744662	0.2653383059782572
0.2283965180787994	0.5072436334764496	0.0146488522897769
0.7277156513695344	0.9957710001064022	0.0109698078150615
0.1337734313273390	0.3863032805674934	0.7273451126480583
0.1176970141742251	0.2140308553001337	0.7343009288618494

0.2523298635664645	0.1188997457186297	0.9886807236819659
0.6142426258695950	0.9209088986133233	0.7306517825868482
0.6412651067913350	0.6740457223319548	0.7247999388451486
0.7375126170148355	0.6186429649860085	0.0452402782748357
0.8706257685559466	0.6173138191552635	0.9721350555875600
0.8833619044314389	0.7832917994608528	0.9792754314695590
0.7547172337091478	0.8835465506028035	0.7377717447808463
0.3928800243926506	0.0847433100646702	0.9847919547481050
0.3751016358109809	0.3260676401299493	0.9764486294513739
0.2700281622314015	0.3808302196442626	0.7925914817461536
0.8932561524058588	0.4186882109713093	0.5411049419767224
0.8748679206902957	0.1780868664977898	0.5486664931684404
0.7684070543474399	0.1237422917823131	0.7306258256342097
0.3683206927204379	0.8846661248866979	0.5547409380139068
0.3913795665697075	0.7111287139437534	0.5441219970870561
0.2528302675800736	0.6184699177007703	0.7937910659085572
0.1134964819255137	0.5835861037866189	0.7969284607728848
0.1300354630251462	0.8264538503884611	0.8049567887743797
0.2336480507979129	0.8829122450758592	0.4866358663592847
0.6327391774746554	0.1156053160047947	0.7960023116801984
0.6162385019887744	0.2904366625492865	0.7897143537254042
0.7524421632987011	0.3853258110565365	0.5384933993859228
0.8997517874771591	0.5717554979123020	0.8051741685333985
0.8703902556354376	0.8217990715357122	0.8067923224446544
0.7819552455125063	0.8778097680542528	0.5171194226938896
0.3642036777285882	0.1114740454783117	0.8112307299412639
0.3871770091586949	0.3026015003196289	0.8007525798062106
0.2450795024750793	0.3796142551470735	0.5167973391832987
0.1133709841537233	0.4277893073640782	0.5574243243313809
0.1400843195159527	0.1816792366984393	0.5619027841744961
0.2228427619908781	0.1249271041839348	0.7649249562545521
0.6395379958648281	0.8998930260459901	0.5559802579116460
0.6154280867671668	0.6928911640962728	0.5358798527786933
0.7727339982780834	0.6280713300373408	0.7700393630008674
0.1440258657690061	0.6110741826941357	0.9702059057740632
0.1189655405524867	0.8018959293791795	0.9808486402009615
0.2609190700594919	0.8766292895609241	0.7667077343243632
0.6164416171003908	0.0756490690703642	0.9669072606053418
0.6409915497496768	0.3228421894327851	0.9617277225865146
0.7215442832838940	0.3794063328075545	0.7593143160101742
0.8630755365304753	0.3911293316745522	0.7142785139172241
0.8856971464111214	0.2017296946898440	0.7248593655015353
0.7461814737069916	0.1260753232062125	0.0137389097643097
0.3923441872607702	0.9241653224912183	0.7244823842391108
0.3618263784994684	0.6823000893600981	0.7167899389302080
0.2852506788865984	0.6262535159822707	0.0164820651452565
0.2574062626941322	0.2520332042223157	0.7526738860191965
0.7444062954851171	0.7517086584824516	0.5011699634074251
0.7661858695388806	0.2580203740673606	0.4943665497748252
0.2385114112899808	0.7468425542197978	0.7512143471130238
0.7448296948281706	0.7566088315628436	0.7897263001464496
0.2639460745939575	0.2489496992545269	0.5305474879270251
0.2545213652006281	0.7557941683002042	0.5284095446913357
0.7559676721644333	0.2523643762353217	0.7723818162768565
-0.0000659793787243	0.3972428139721339	0.6596158024007570
0.0050075253365757	0.1948682118614786	0.6246689855204306
0.5042441128949765	0.9026274459583272	0.6162311874262780
0.4973198156499298	0.6860776638557136	0.6640173351312525
0.0072263999273650	0.6070736283372606	0.9146753342100740
0.0006886424297443	0.8063356457000245	0.8797289957990533
0.5006296424562747	0.1069374236676789	0.8684441564724251

0.5055824031877869	0.3075615815633775	0.9027576907615550
0.0911236951485977	0.3015358225664774	0.6022626499002515
0.5905012487916241	0.8010936668156987	0.6603836911121966
0.9176859954907605	0.6998275413456627	0.8463047276862221
0.4118543977221767	0.2059906065546721	0.9203818715666675
0.9134055995694931	0.2976013027399634	0.6052388150326743
0.4215521649740088	0.8001475652127767	0.6733620987045847
0.0962591866040483	0.7055028684348339	0.8607047357752773
0.5938958814654079	0.2019556245881398	0.9217110251632865
0.1800698540687900	0.2096579588178966	0.8939745622797042
0.1618816474532584	0.4345869979995938	0.8886500917262677
0.6798439964180069	0.6684199145970590	0.8949762602776966
0.6803153038731012	0.9069496462154404	0.8893477354233008
0.8228378368280548	0.7902208686376265	0.6434477905133512
0.8490476718357123	0.5688082564332370	0.6380269897158335
0.3314123262662786	0.3315823262831126	0.6433596653564478
0.3259952303494404	0.0978229410022520	0.6427063502078726
0.8286220541723460	0.1722058138466413	0.8813709030155603
0.8250536675884176	0.4026745220094953	0.8828946563878379
0.3269929085098007	0.7099895186958499	0.8858412540668692
0.3449282029098774	0.9332317545750730	0.8915092656936098
0.1753919869414276	0.8310111597367016	0.6369698198328485
0.1813220960109389	0.5985284533049898	0.6383901122200669
0.6787962418874742	0.2940647247795664	0.6305201485396954
0.6617069855602264	0.0669762605172535	0.6354346885490320
0.2150668979348006	0.4963386075534470	0.7430904523995154
0.7169617094635815	0.0069509667244344	0.7817247703758278
0.7914987877162706	0.5075215999671666	0.4938107858971741
0.2921347326252635	0.9975054845079188	0.5348193100508210
0.7833534735397201	0.4966652912146520	0.7645110234070512
0.2822779381406153	0.0066524895528300	0.7635124064130013
0.2225133661993981	0.5093217222158496	0.5136461089073607
0.7287634629260905	0.9981363812992228	0.5053759742907293
0.4666541014755028	0.5544645370053674	0.8810905104827709
0.4082464696466871	0.5182536051828249	0.8433029677059855
0.5253812588798554	0.5716302623432390	0.8244637275598183
0.4089982928659121	0.4984739768078238	0.7530952758183730
0.5265208058645581	0.5513649623582049	0.7339443390868534
0.4688316303264657	0.5133396333493095	0.6968655884531451
0.4737870778466535	0.4872035971502355	0.6016370757960133
0.5288970339856003	0.5744971528124512	0.3855267347136570
0.4569410355825537	0.5700182695708669	0.3529915752988956
0.5788173637678574	0.5230942577111154	0.3679249540041925
0.4345489006954042	0.5144749215519651	0.3030570403096396
0.5565868907306483	0.4674286332698346	0.3182275698509051
0.4844545966979479	0.4630477083867894	0.2857950991950632
0.4687283026672482	0.5710054897545842	-0.0199051948501349
0.4017243143877973	0.4899796528011811	0.5487573067497994
0.5141452882179360	0.5200174798621315	0.0338298133783935
0.5142908571497329	0.5323238900405038	0.1060386007851123
0.4913344692982592	0.4695419035438343	0.0257865745386148
0.5714310784931032	0.5190461793388224	0.0099467265353939
0.4091378901364813	0.4691158888230384	0.4810495494377849
0.3581049748948798	0.4616089244933466	0.5826664683058659
0.3824230975565845	0.5415218496328374	0.5412901912250204
0.4123016236292327	0.5722360323084874	0.0067624316748342
0.4925665592867977	0.6208935332538889	-0.0102157744961949
0.3613692331387223	0.5051001230513418	0.8858733700882604
0.5712613474322273	0.6006040351226997	0.8511859661412265
0.3625489806497074	0.4702875835493623	0.7265912633824378
0.5752475577013084	0.5619323127115305	0.6930691757636996

```
  0.5462459814713205  0.6179936456276223  0.4241831288223191
  0.4182203062573538  0.6101294869560601  0.3668139121566795
  0.6352081974572257  0.5266667791400351  0.3926851488866902
  0.3782035807544603  0.5112100470537111  0.2780269380851649
  0.5957667361017187  0.4276785987449371  0.3042015975370607
  0.4673025885197551  0.4194522572265815  0.2471819886101226
  0.5168308472974399  0.5146864133941541  0.5649203794204852
  0.4927873578199338  0.4349796773047492  0.6046644423493490
  0.4965439574643404  0.6405423859838641  0.6933713375131477
```

MOR-Al-T4-TS1-Transalkylation

```
   Al     Si     O     C     H
   1.00000000000000
    18.1842269897460938   -0.0090872207656503   -0.0001607598969713
    -0.0105508984997869   20.0691013336181641    0.0015994716668501
    -0.0001797722798074    0.0011982563883066   14.8440780639648438
   Al   Si   O    C    H
     1    95   192    16    21
Selective dynamics
Direct
  0.5857723355293230  0.7146797776223125  0.6434229612350472
  0.3147222101688384  0.0746521949768176  0.0371397100389004
  0.3031394481659080  0.3202055096626850  0.0397063456475756
  0.8124085664749148  0.5761272311211441  0.0330730937421347
  0.8093668222427369  0.8006237745286819  0.0355367697775391
  0.6913423538208777  0.9274784326555103  0.2844904959201817
  0.7065253853797915  0.6831563115121354  0.2847284376621250
  0.1954870969057083  0.4257181882859157  0.2882485985756026
  0.1946008652448702  0.2006293088198095  0.2865335345268250
  0.6936749815940857  0.0778115987777832  0.2359833717346575
  0.6938475370407104  0.3029660880566311  0.2368239760399138
  0.1930865496397038  0.5754956007005224  0.2407666891813535
  0.2020703107118693  0.8208999633791155  0.2373255938291762
  0.3098132312297821  0.9267881512642953  0.4905585348606114
  0.3085669577121836  0.7068428397180411  0.4879266023636282
  0.8147293329238895  0.4302550256252812  0.4877023994923022
  0.8027587532997787  0.1841962188482573  0.4841269254684868
  0.7076344490051267  0.9192181229593461  0.4925779402256462
  0.6951256394386293  0.6841864585877344  0.4937305748463062
  0.1871767342090756  0.4387818574905972  0.4934379756451113
  0.2091404944658444  0.1919195204973549  0.4933218955994409
  0.2977839708328306  0.0849329233169720  0.2448409944772951
  0.3107612133026451  0.3171076178551054  0.2474245727062482
  0.8191522955894861  0.5672308206559519  0.2356385588646135
  0.7982143759727478  0.8144272565842721  0.2406847774982789
  0.3197636902332327  0.9359024167063271  0.2851032912731418
  0.2959987521171571  0.6922587752343536  0.2826338410377502
  0.7973408699036301  0.4202900230885537  0.2794280946254730
  0.8094521760940553  0.1856081485748783  0.2761724293232318
  0.6881734132766725  0.0666367560625186  0.0309542883187564
  0.7089706063270569  0.3136063516140630  0.0307170934975175
  0.2098759561777204  0.5856080651284576  0.0319986082613500
  0.1950472593307542  0.8177332282067391  0.0307609718292982
  0.0856141746044168  0.3800759911537827  0.1352653652429779
  0.0892149806022644  0.2249367088079454  0.1302208602428600
  0.5890278220176806  0.8755261898041818  0.1407082974910822
  0.585476219654083l  0.7155438661576609  0.1471506059169851
  0.9181200861930854  0.6255722045899773  0.3819933533668884
  0.9152081012725827  0.7803751230241172  0.3804494142532617
```

0.4169283509254457	0.1283668726682883	0.3932452797890139
0.4200934767723093	0.2865355610847913	0.3979583084583286
0.9169040918350225	0.3779460787773778	0.1308172494173214
0.9198250770568848	0.2189897000789945	0.1263015866279629
0.4211964905262030	0.8788338899613473	0.1415904909372332
0.4175765812397174	0.7238965034485934	0.1458413004875221
0.0871122255921372	0.6277755498886988	0.3812122344970707
0.0851055756211299	0.7862049937249566	0.3791112005711167
0.5855849385261539	0.1256112158298590	0.3880186080933281
0.5891277790069581	0.2807024121285150	0.3936356008053210
0.3165008425712585	0.0769772529602137	0.5375669598579457
0.3035928606987012	0.3223250806332074	0.5398266315460213
0.8118841052055360	0.5792810320854187	0.5379516482353867
0.8082860708237323	0.8017320036889168	0.5357962846755986
0.6901664137840273	0.9287829995157273	0.7838218212128503
0.7051368951798066	0.6815267205239890	0.7895931601525482
0.1952597498893738	0.4266013801097900	0.7867071032524844
0.1943211704492750	0.2016199529171022	0.7860664725304414
0.6947069764138006	0.0775273516774298	0.7352913618088478
0.6933753490447998	0.3037912249565453	0.7365580201149909
0.1898214221000725	0.5763993859291956	0.7404193878173836
0.1992674767971076	0.8233472108842701	0.7381259799004433
0.3110848963260651	0.9249258041381897	0.9895313382148750
0.3114437758922883	0.7026296257974030	0.9907383322716915
0.8158217072487295	0.4274325072766167	0.9855508804322102
0.8033512234687805	0.1837667077780093	0.9839869737625130
0.7077339291572680	0.9181277155878741	0.9914190769195750
0.6944417953491377	0.6823017597199822	0.9982056021691208
0.1876960396766686	0.4369888305664801	0.9922958612443431
0.2092875540256612	0.1902984678745598	0.9928884506226672
0.2988404333591461	0.0863373428583251	0.7448245286941536
0.3108772635459900	0.3181508779526089	0.7471101880074387
0.8236925601959231	0.5670614838601038	0.7429577708244332
0.7956734299660385	0.8119089603425268	0.7427682280541279
0.3192014992237352	0.9368453621866047	0.7843706011772164
0.2919994592666627	0.6940661668778757	0.7843180298806487
0.7983568906784061	0.4181223809720080	0.7790932655335632
0.8090181946755357	0.1871337592602093	0.7759199142456944
0.6872221827507019	0.0672068074345699	0.5299454927445114
0.7095648050308230	0.3142007291317450	0.5305062532424931
0.2035267800092732	0.5884996056557358	0.5310754179955185
0.1923824697732925	0.8223132491112801	0.5302038192749028
0.0851703137159375	0.3798154890537918	0.6352109313011177
0.0884440094232559	0.2245499193668367	0.6303727626800537
0.5875911116600409	0.8775723576547808	0.6407435536385238
0.9227175116539955	0.6238049864769629	0.8833572864533310
0.9161128401756581	0.7775993943216100	0.8772931098939020
0.4172496497631072	0.1274302452802951	0.8949540853501073
0.4201964139938354	0.2855313420295789	0.8991050124169665
0.9170661568641666	0.3772212266922432	0.6291104555130763
0.9194954037666320	0.2191057056189056	0.6252380013465889
0.4210294485092398	0.8794817328454365	0.6405551433563325
0.4154652953148228	0.7242501378060043	0.6460039615631936
0.0897627323865944	0.6278415322305158	0.8839862346650411
0.0851563811302203	0.7858152985574127	0.8798639774323217
0.5861552357673936	0.1246655136346937	0.8875387907028206
0.5890967249870300	0.2801625132561376	0.8927852511406824
0.1342600733041830	0.3888983726502345	0.2261732816696422
0.1184102818369866	0.2159069627523761	0.2332135885954282
0.2536839246749878	0.1218860372901219	0.4881489276886414
0.6136939525604435	0.9145560860636015	0.2322143167257527

0.6303751468658322	0.6796476244927765	0.2277951836586285
0.7383797764778725	0.6238700747491265	0.5466780066490181
0.8720628619194422	0.6161760091782496	0.4743891358376129
0.8858079910278327	0.7886128425599480	0.4833247661591014
0.7551859617233276	0.8852811455728716	0.2327031046152276
0.3937220871448517	0.0868728384375736	0.4833822548389788
0.3748784363269806	0.3271843492985428	0.4744933545589958
0.2696496546268651	0.3806457519532095	0.2934097349643996
0.8931178450584414	0.4178493916989252	0.0396021679043799
0.8761812448502243	0.1798463165760318	0.0469047352671667
0.7691993117332460	0.1213052347302568	0.2311520278454084
0.3706556558609009	0.8865326046943708	0.0519694499671523
0.3891401588916789	0.7158472537995676	0.0418118834495560
0.2555049359798539	0.6201364398003710	0.2915434837341670
0.1153926029801373	0.5859082341194809	0.2934423685074404
0.1309252232313218	0.8266336917878956	0.3025470674038094
0.2361456751823717	0.8806875944137581	0.9832532405854175
0.6334864497184757	0.1174026280641687	0.2965120077133183
0.6169349551200867	0.2902119755745315	0.2898806929588322
0.7523083090782322	0.3843754827976886	0.0372643508017079
0.8892894983291826	0.5763295888901413	0.3025195002555851
0.8658812642097531	0.8239186406137652	0.3108632266521837
0.7821284532547237	0.8755470514299244	0.0103293284773834
0.3635551929473877	0.1111592873931081	0.3094075024128314
0.3883026838302613	0.3048008084297538	0.2984135746956271
0.2445790767669734	0.3769821226597272	0.0114957485347995
0.1153251752257347	0.4297302589416552	0.0559362918138552
0.1402166485786562	0.1825138181448227	0.0611853264272300
0.2218417376280120	0.1251668930053933	0.2647397816181185
0.6412807703018015	0.8948382139207243	0.0568302758038096
0.6160753965378153	0.6910691261292383	0.0495291240513353
0.7639365792274828	0.6295493245126202	0.2435554265976150
0.1334425359964452	0.6094540953637482	0.4708353877068112
0.1160626634955502	0.8059530258179597	0.4784263670445068
0.2625856697559358	0.8760279417039722	0.2652903199196190
0.6142871379852296	0.0760841369628988	0.4669804871082819
0.6410460472106936	0.3233570456505426	0.4614980220794682
0.7228566408157352	0.3778717219830215	0.2589445114135975
0.8642470240593944	0.3966772854328847	0.2147993445396633
0.8864718079566960	0.1990815848112547	0.2241909056902163
0.7431743144989014	0.1283811777830125	0.5121313929558517
0.3925616443157433	0.9274156689644906	0.2219909876585194
0.3641529083252075	0.6825686693192818	0.2133861333132027
0.2742393314838409	0.6331394314765931	0.5041795372963608
0.2589399516582490	0.2515067458152771	0.2536109089851379
0.7431502938271149	0.7492799758912397	0.0119876489043246
0.7644746303558353	0.2563254535198693	0.9942674636842127
0.2382570207118974	0.7469252347947046	0.2439985275268811
0.7417572140693667	0.7575318217278207	0.2768505811691413
0.2657582759857178	0.2470502853393816	0.0300150103867083
0.2480724155902864	0.7524753808976099	0.0275813676416901
0.7563177943229675	0.2504903674125999	0.2688456773758302
0.0006477084825747	0.3984227478505061	0.1571411937475381
0.0055832890793683	0.1974641084671394	0.1236114427447496
0.5053640604019168	0.8978168964387032	0.1164042502641793
0.4996172189712527	0.6934360861779595	0.1531932353973538
0.0030509107746185	0.6069590449334341	0.4020062386990055
0.9993651509286021	0.8069453835489088	0.3745895624161160
0.5003459453583420	0.1084403097629632	0.3653428256512108
0.5058625936508176	0.3081657886505773	0.4019819796085853
0.0929609611630445	0.3032872676849893	0.1009952649474256

0.5933628678322281	0.7958399653436066	0.1576285660266880
0.9108270406723055	0.7024812102319122	0.3486603498459239
0.4108525514602661	0.2072068005800687	0.4166690409183886
0.9110723733902788	0.2986540794373206	0.1101835519075418
0.4158470630645790	0.8018874526024911	0.1758409887552452
0.0953755080699918	0.7068295478822769	0.3602952659130466
0.5933156609535218	0.2024280279874802	0.4226267635822750
0.1788347959518433	0.2105762958526648	0.3934079110622986
0.1616053283214569	0.4344806075097700	0.3886526226997379
0.6897892355918888	0.6633331775666279	0.3877407312393572
0.6839786171913167	0.9043746590616228	0.3884078860283477
0.8302620053291717	0.7948818802835576	0.1418498903513048
0.8484081625938630	0.5629683732987677	0.1319716572761570
0.3293552994728090	0.3326213061810196	0.1426607370376589
0.3239372670650810	0.0975954011082828	0.1412614583969326
0.8287625312805748	0.1721776425838888	0.3809942603111661
0.8233121633529643	0.4080667495727556	0.3830637037754063
0.3261655271053633	0.7155225276947773	0.3809303343296471
0.3448449969291687	0.9343725442887399	0.3902381658554454
0.1754604727029812	0.8342780470849842	0.1346951723098799
0.1847960948944100	0.5974690914154951	0.1359285414218903
0.6772513985633852	0.2944286465645164	0.1297836303711102
0.6603901982307434	0.0688409879803740	0.1351360678672792
0.2176061719656098	0.4976008236409114	0.2452647536993345
0.7124251723289490	0.0061418744735434	0.2806052863597874
0.7919720411301356	0.5053164958954012	0.9854802489281451
0.2906593084335429	0.9967441558839408	0.0335811823606509
0.7770353555679324	0.4980903565884403	0.2617639005184176
0.2804372906684875	0.0066581247374432	0.2625980377197266
0.2284034788608550	0.5074883699417994	0.0134815638884914
0.7279955148696903	0.9960232377054066	0.0099583398550756
0.1343270987272262	0.3886002302170242	0.7258763313294296
0.1174317449331428	0.2155932337045678	0.7337534427642822
0.2517533600330587	0.1192158386111484	0.9869966506959335
0.6131536364555361	0.9207653999330413	0.7297244071961206
0.6376546025276408	0.6718148589135552	0.7229452729226186
0.7367088198661846	0.6183978915215874	0.0425270684063473
0.8710006475448611	0.6168572902681204	0.9718917608262263
0.8830806016922049	0.7828807830811871	0.9789278507233590
0.7536915540695235	0.8833135962488360	0.7366376519204109
0.3923083841800691	0.0851964801550073	0.9837272763252266
0.3756027519703209	0.3264160454273664	0.9761945605279247
0.2707859277725241	0.3831325173378647	0.7912278175354012
0.8927602767944338	0.4194435179234074	0.5400829911232177
0.8749438524246218	0.1786434352398139	0.5478920936584480
0.7686954736710251	0.1233729422092625	0.7299170494080357
0.3675830662250870	0.8861431479455097	0.5527821183205166
0.3847696781158488	0.7182856202126604	0.5422807335854284
0.2517388463020531	0.6214453577996636	0.7920660376549495
0.1124985516071353	0.5850692987442673	0.7943992018699654
0.1280537396669394	0.8288099765779435	0.8034002780914986
0.2317509353160890	0.8870241642000050	0.4847038686275956
0.6331666707992555	0.1158533245325219	0.7955844998360387
0.6162418127060957	0.2906646430492760	0.7890599370003514
0.7522623538971716	0.3851365745068154	0.5379610061646275
0.8981472849846123	0.5717787742615572	0.8046382069588431
0.8680137395858963	0.8210720419885487	0.8066985607147541
0.7819034457206727	0.8779362440110948	0.5168988108635593
0.3640780746936800	0.1123434156179648	0.8101260662078865
0.3871127963066101	0.3040982484817841	0.8004025816918492
0.2466517090797426	0.3815234303475094	0.5155850052834213

0.1145808175206247	0.4293200671673850	0.5563430786133580
0.1401207596063669	0.1826075315475737	0.5615812540054321
0.2228500992059706	0.1267387717962485	0.7637153267860513
0.6390677094459536	0.8993899226189705	0.5557749867439278
0.6150596141815358	0.6938974857331613	0.5350124239921912
0.7704358696937789	0.6278975009918245	0.7687278985978501
0.1437896788120275	0.6111769676209936	0.9677872061730829
0.1185420304536823	0.8028473258019725	0.9788644909859862
0.2600960135459891	0.8783363103868519	0.7653841972351784
0.6163381934165975	0.0757368132472181	0.9664660096169940
0.6406286358833313	0.3230091333389928	0.9611902832985767
0.7214434146881101	0.3793069422245465	0.7580184936524179
0.8634810447692964	0.3925026655197654	0.7135992050170906
0.8856657147407538	0.2019894868135892	0.7240110039711006
0.7460525035858158	0.1264425516128546	0.0130607523024098
0.3913203179836325	0.9276184439660243	0.7211845517158516
0.3585131168365704	0.6857334971429015	0.7134119272232063
0.2857104241848009	0.6260595917703057	0.0108281038701543
0.2568580508232118	0.2540093362331718	0.7528908848762520
0.7455072999001247	0.7515675425530182	0.4989413022995456
0.7657825350761961	0.2577421069145738	0.4934515655041448
0.2343220710754490	0.7491813898088320	0.7482438683510619
0.7420952320098879	0.7559807896615833	0.7865954637528096
0.2633029222488403	0.2504547238350243	0.5297898650169377
0.2476592659950264	0.7594889998437310	0.5228242278099452
0.7557986378669739	0.2520776093006780	0.7711176276207737
0.0002763183438220	0.3981454968453121	0.6574942469597744
0.0051155588589610	0.1964989751577645	0.6235979199410524
0.5038418173790034	0.9016248583794768	0.6151236891746521
0.4929664452400790	0.6835486815639445	0.6602947321979069
0.0068992851302029	0.6072899699212337	0.9128575325013389
0.9994336962699921	0.8066634535791583	0.8783279657364741
0.5005658864974976	0.1069567799568307	0.8677592277528183
0.5057026147842404	0.3082095980644879	0.9017012715339668
0.0920880436897308	0.3029502928257428	0.6012884378433966
0.5918797254562720	0.7998481392861460	0.6607761383056648
0.9169186353684626	0.6997024416924953	0.8454180955887844
0.4123054444789888	0.2064054161310198	0.9190973639489082
0.9127218723298274	0.2984815835953442	0.6048217415809639
0.4172074496746065	0.8025198578836472	0.6750910282135018
0.0956744924187671	0.7065876126290862	0.8593716025353247
0.5935918688774868	0.2018903642893116	0.9210779070855323
0.1794524341821671	0.2106512933969509	0.8933024406433873
0.1617425978183842	0.4353316128254460	0.8875133395195247
0.6779713630676716	0.6683341860772239	0.8931911587716111
0.6798505783081472	0.9072574973107900	0.8886037468911244
0.8228414058685302	0.7900564074517695	0.6426563858986608
0.8478536605835617	0.5682453513145833	0.6373427510262014
0.3318632543087006	0.3324561417103253	0.6428807377815954
0.3261926174164268	0.0994482263922843	0.6419162750244980
0.8285889029502869	0.1723680943250983	0.8805524110795120
0.8243694901467129	0.4025095701217729	0.8820412158966988
0.3242225646972829	0.7135056257249578	0.8832536935807042
0.3452406823635304	0.9344530701638373	0.8892784714699631
0.1729261130094570	0.8356359004975412	0.6352077126502998
0.1818202733993626	0.5999327301980826	0.6365137696266182
0.6782059073448837	0.2940640747547738	0.6295306682587506
0.6613199710845947	0.0676949024200521	0.6347162127494820
0.2160631120205005	0.4988679885865138	0.7434307932853707
0.7164581418037415	0.0066451011225587	0.7805014848709874
0.7899962663651414	0.5076650381089136	0.4932356476784460

```
  0.2922804057598056  0.9992982149126729  0.5341160893440472
  0.7815874218941298  0.4967612028122582  0.7643277049065403
  0.2808119654655457  0.0082783494144699  0.7628685832023628
  0.2246302217245102  0.5108377933502207  0.5133038759232382
  0.7281779050827236  0.9979079365732378  0.5052253603935710
  0.5110310316085821  0.5385323762895006  0.8988739848138229
  0.4380012750625610  0.5540384650231288  0.8732852339744576
  0.5636931657791140  0.5233544707298935  0.8317314386369067
  0.4208444725457747  0.5581409381911354  0.7821179361510727
  0.5479448704438907  0.5336891346652513  0.7420258969222535
  0.4759844168639031  0.5533628263822685  0.7128980662126061
  0.4560419602361110  0.5349535385872413  0.6145194438832546
  0.5276095867156982  0.5759043693543360  0.3913977742195493
  0.4559130370616929  0.5707585215569199  0.3578380048275358
  0.5778783559799195  0.5244336128235519  0.3756086528301735
  0.4342212080955833  0.5144671797752548  0.3090668320655827
  0.5563921928405762  0.4680631160736110  0.3268867135048374
  0.4844936132431451  0.4629313349724862  0.2937142252922062
  0.5305246710777283  0.5345978140831057  0.9962422251702728
  0.3750213417297062  0.5199097911022657  0.5999956797852987
  0.5180993676185608  0.4627929925919281  0.0309570524841575
  0.5331997871399392  0.4601893126965615  0.1025049909949333
  0.4601599574089066  0.4480322599411037  0.0239606183022261
  0.5516747236251874  0.4270720779896811  0.9929842352868128
  0.3648660267183774  0.5089085663286250  0.5287378033956249
  0.3568605437420208  0.4771715186009389  0.6406605118569538
  0.3408634992214605  0.5629138117191016  0.6181644755684872
  0.4964025616646442  0.5694792270661331  0.0352480821311501
  0.5885033011437132  0.5485633015633409  0.0055648051202301
  0.3974457383156172  0.5641322135926418  0.9257569909096798
  0.6194691658020023  0.5098524689675034  0.8517207503319626
  0.3641766623686046  0.5664127008008946  0.7619762868651853
  0.5905141107895786  0.5262877319967505  0.6911478180982776
  0.5445261001586913  0.6199783086777613  0.4291424155235612
  0.4170506000518926  0.6111528277398129  0.3696474432945334
  0.6340913176536561  0.5286518335343063  0.4008872509002716
  0.3781453669071151  0.5107463002206231  0.2831403017044071
  0.5958447456360446  0.4282197654248242  0.3143945634365086
  0.4677080810070224  0.4186144471169289  0.2564240992069247
  0.4737820739721518  0.5743759707265482  0.5677744207223575
  0.4886986510743961  0.4908883251502926  0.5962337605958143
  0.4861727761214312  0.6219902477877395  0.6955145225365420
```

MOR-Al-T4-DEBH+Benzene

```
   Al     Si     O     C     H
   1.00000000000000
    18.1842269897460938   -0.0090872207656503   -0.0001607598969713
    -0.0105508984997869   20.0691013336181641    0.0015994716668501
    -0.0001797722798074    0.0011982563883066   14.8440780639648438
   Al   Si   O    C    H
     1    95   192    16    21
Direct
  0.5892279873084673  0.7163762227176090  0.6402158714679599
  0.3146857083529183  0.0744041038890496  0.0302380666197059
  0.3035740839020799  0.3196412615760924  0.0333767727091767
  0.8147836572324578  0.5764284750359392  0.0301233450801483
  0.8109351562660521  0.8016366931988574  0.0305558886553641
  0.6924081827668389  0.9272951495228807  0.2803038353045776
  0.7076451723424335  0.6824961942909505  0.2799445972886637
```

0.1956125028954530	0.4250761595722637	0.2821213556773943
0.1955001741127532	0.2001394909905959	0.2805992351525529
0.6942630711054728	0.0776281197461367	0.2320143048046090
0.6948101886190717	0.3030045358958285	0.2329388532933425
0.1932142036264760	0.5744801034979147	0.2334187842514636
0.2044669030098244	0.8192279167225816	0.2299465977280349
0.3128956088993707	0.9241172597739807	0.4831578808081975
0.3105646842707730	0.7018240044586669	0.4836242157433450
0.8146582897143640	0.4295753475483508	0.4839090475482355
0.8025313971007112	0.1837232970347619	0.4800865129481858
0.7087913120551584	0.9185770969093048	0.4882862404750469
0.6992659383781231	0.6853930531503284	0.4879664495314027
0.1881808374395074	0.4360855235182344	0.4878136388403480
0.2095638783529921	0.1893739580911903	0.4873704318213489
0.2982628620720508	0.0838853202660935	0.2380202514899518
0.3112496605347148	0.3167436516246050	0.2410943641698300
0.8218452033128369	0.5671843789772562	0.2329239460211128
0.7989259991339773	0.8138444924069176	0.2360353299607481
0.3211821679750423	0.9352027199056047	0.2775122453642064
0.2972896406371500	0.6901989080299815	0.2767092798788643
0.7977756531306376	0.4200996805509281	0.2756030641870431
0.8102349821280250	0.1855807121971388	0.2723142647210063
0.6876177309036289	0.0670234420775377	0.0271002986354846
0.7092487650886375	0.3138969255151033	0.0266742687241866
0.2110580756203793	0.5853804665218163	0.0255760798044174
0.1969839067814721	0.8166416518828609	0.0227341467240826
0.0855376394580459	0.3792732167384816	0.1305116727661891
0.0893582196427366	0.2242592280577861	0.1249964658816936
0.5910386390606570	0.8750498105097175	0.1358362802244063
0.5873430816793331	0.7152503797966874	0.1421413618705714
0.9215975784825398	0.6245950596183187	0.3799152700642708
0.9178865829779104	0.7802079140713095	0.3758577777840659
0.4168537447110980	0.1260700771407671	0.3873017292723820
0.4201024546769364	0.2845048541635392	0.3921286979118068
0.9168440738579839	0.3772747010510465	0.1266960227282095
0.9199421304450192	0.2182976411175624	0.1223414555845959
0.4229367665575116	0.8787184449695375	0.1349626848853979
0.4188934903638953	0.7236789824785856	0.1404870391440652
0.0909919969810150	0.6266800992275872	0.3764392401725525
0.0874431624853411	0.7855105583209221	0.3723461615772345
0.5856731540384903	0.1240024848697764	0.3843561270639477
0.5894467097703386	0.2789180536785721	0.3894229049143460
0.3149509146552151	0.0737136148951281	0.5305902393473875
0.3033462851703964	0.3189418148773474	0.5339634735449541
0.8136669355581303	0.5785562485423379	0.5345059763523762
0.8124632494400518	0.8022257344045989	0.5294695071964595
0.6931878751282750	0.9298029743088289	0.7795719794804031
0.7071514991724975	0.6812012299552200	0.7853859556922912
0.1949115176756088	0.4249937685164967	0.7815804631336497
0.1944405073237374	0.2006792425946789	0.7804304603901159
0.6963681318570010	0.0778421920796660	0.7314526707605750
0.6943923606600395	0.3034983123138427	0.7326117319809069
0.1917395490171898	0.5747766561057586	0.7338516531995564
0.2023379603433383	0.8207901624322607	0.7316099581709868
0.3125469976859714	0.9247096691774059	0.9829185592516633
0.3139001557138730	0.7008925449139339	0.9838564249943803
0.8156003392477693	0.4277263792343421	0.9815769428655950
0.8030233150945691	0.1840361562286774	0.9802739314118325
0.7096205184023047	0.9188622210814361	0.9870218473886123
0.6968365544919304	0.6830814724600061	0.9938632164244816
0.1875881549096468	0.4364438420894882	0.9870462257824328

0.2091273158868282	0.1898493085370841	0.9866810432642792
0.2984578231512532	0.0847468593619993	0.7384486060247494
0.3109982239650327	0.3174002236887857	0.7414985013007117
0.8249999217713384	0.5664864493518486	0.7396137512242983
0.7969860189687714	0.8116524155332164	0.7371465178538075
0.3200170596770024	0.9359523251568418	0.7781331914491688
0.2957055778230399	0.6906933397705123	0.7776118574186268
0.7986598553110618	0.4179376877648637	0.7750373414239804
0.8102254735916078	0.1874654584363764	0.7724327525238136
0.6874677661267538	0.0664114128104104	0.5262941943516346
0.7094193704453740	0.3135245528696364	0.5264594815200434
0.2095923457585120	0.5844307109648202	0.5260117618433847
0.1949893283422994	0.8175426332882804	0.5232372700382685
0.0854051329773280	0.3785216173025254	0.6295844900803197
0.0885941698745158	0.2234228395133000	0.6238480341689489
0.5911090728414033	0.8772456746478777	0.6391129235379311
0.9247382561055275	0.6229561327192229	0.8794571998430267
0.9175014108944920	0.7772120311707129	0.8721837265603950
0.4173225609044512	0.1267815234594971	0.8874193683546029
0.4206465723024777	0.2851881850414213	0.8921625337876352
0.9173186716427453	0.3764646209689190	0.6253570145528599
0.9197441178706069	0.2182708107453132	0.6215613383821053
0.4225191315426323	0.8782233983750898	0.6356785386372963
0.4180669829383767	0.7235771505777836	0.6394801343719599
0.0915034792234717	0.6273725163735318	0.8768356877945580
0.0863863314134975	0.7852219110250918	0.8723877479330836
0.5861640662207730	0.1239639606473143	0.8832093857856820
0.5894393010562223	0.2794629599688855	0.8888059167923908
0.1339885581480375	0.3873981962629025	0.2217040543819833
0.1189480321604064	0.2151513621078632	0.2279607043453099
0.2516910995499595	0.1182028767176597	0.4808596879575597
0.6149901775391444	0.9144062227634595	0.2273033098828751
0.6319143635431964	0.6778714004626344	0.2218409054682937
0.7408624135439330	0.6243071317894575	0.5412949706706693
0.8756154433842392	0.6139198970459012	0.4719668091388948
0.8917741766957992	0.7917741479940742	0.4796335216071302
0.7565804943289847	0.8850593121926160	0.2291745042239622
0.3922542407085156	0.0846455746924673	0.4768390570985435
0.3749742607542712	0.3244966372301845	0.4694051635542192
0.2701347969033270	0.3803730970641833	0.2868361469896182
0.8928503327668184	0.4171088475310488	0.0355916378450331
0.8755108185936349	0.1791014234511096	0.0437125831398594
0.7692967492004815	0.1217895364097307	0.2268665140860653
0.3726626386954888	0.8867331766073044	0.0452074187841093
0.3907022364551221	0.7149892362985856	0.0365427313971508
0.2568975229162396	0.6179627444690071	0.2836582780054890
0.1158243730692350	0.5856217117593416	0.2863421109721659
0.1335950524285473	0.8252576248361405	0.2954922585269179
0.2379497572005122	0.8801316515693384	0.9765448370069301
0.6338586683527246	0.1163889280043801	0.2930475965013972
0.6182107598348997	0.2888107657538422	0.2862715119257653
0.7515760698839863	0.3851458548369520	0.0331109971516897
0.8923550543929766	0.5761060780583845	0.2995447777852259
0.8663962157074920	0.8219939709682074	0.3067534120683973
0.7845357596387247	0.8769539435709801	0.0060180671331806
0.3647952619042575	0.1088588295047270	0.3021151419463809
0.3885536500418055	0.3044041007841367	0.2929087346857348
0.2457754703782807	0.3772277721790284	0.0055665623350141
0.1159085047498164	0.4287024338181778	0.0516035773368610
0.1406192871384067	0.1821078429185208	0.0558897383678013
0.2228559056568087	0.1246700197932309	0.2591655319619419

0.6435802911413158	0.8943077954830063	0.0522543754803473
0.6176341871649713	0.6907701167249272	0.0442308530283178
0.7666803717997471	0.6295919322862497	0.2408421708989778
0.1424678064407131	0.6070163450423803	0.4613897321474253
0.1173235219980585	0.8062697344874814	0.4717946719119093
0.2652467675958880	0.8744569095874009	0.2571880180424052
0.6142523146117151	0.0746110395007897	0.4636003594773216
0.6402609549659006	0.3219804626769535	0.4580756216518186
0.7229078951929587	0.3782371756945601	0.2550866818936490
0.8644930434517872	0.3957625060502767	0.2110702671851977
0.8875629899594892	0.1982962659680479	0.2207065007230714
0.7426897049878948	0.1280201461730270	0.5073153612848452
0.3942914096532509	0.9278863265885950	0.2147026579890368
0.3666417177014795	0.6817397903980824	0.2087410458144495
0.2845209271699125	0.6254648112559482	0.5041313746662701
0.2593164862710658	0.2511820375967117	0.2466908415435766
0.7438786324122378	0.7511202649761223	0.0064246791475565
0.7655398986800390	0.2572971920014365	0.9900289686784606
0.2404363372348650	0.7451603228627878	0.2371109044532065
0.7415012461131113	0.7572431996948261	0.2704104616588013
0.2656792856380631	0.2468441237926999	0.0231165040835264
0.2500050187026247	0.7514264960822040	0.0172407860144080
0.7580777325834225	0.2511054541907783	0.2648083510615297
0.0007673852824422	0.3979566170004200	0.1523422722665072
0.0058387409347761	0.1967624860071689	0.1185455678991775
0.5074029346030650	0.8967662966177372	0.1104445038579454
0.5012723370954485	0.6935345366396757	0.1473653503293764
0.0070487241400433	0.6066635462299175	0.4003475460604773
0.0018718344137969	0.8063357178082344	0.3650974034219248
0.5006138191429221	0.1063892594399110	0.3613464957822126
0.5057600579747719	0.3060950825696555	0.3962475525992243
0.0929539810636540	0.3026286501183953	0.0957385584971057
0.5956509190778156	0.7952859686323651	0.1534266189171622
0.9131796791658501	0.7016699772234709	0.3477885868942223
0.4102745594481265	0.2051866598276320	0.4095485069260315
0.9113711017845055	0.2979808351044891	0.1062833819414167
0.4166540429938990	0.8017881322683950	0.1696539450518534
0.0986205777524978	0.7059010887644536	0.3563158965309766
0.5930345491240679	0.2008797369127778	0.4189561467694647
0.1807143074333844	0.2104718839623292	0.3876619108915910
0.1626582021199969	0.4353939359292100	0.3828088934400524
0.6897182697133308	0.6633793230533962	0.3828204779242985
0.6842864201213591	0.9045021118484702	0.3842911864282628
0.8313116458308059	0.7951866425997230	0.1369510797575089
0.8506257174899645	0.5630331712129746	0.1290191731733190
0.3302128938005082	0.3319851857063136	0.1362685909417515
0.3237088316493344	0.0974184742756939	0.1344043761354846
0.8291822049228255	0.1717124367507350	0.3771936815463856
0.8234915744948512	0.4075659729429139	0.3792595443881824
0.3256612660607971	0.7119860559355116	0.3762281383806076
0.3464317420889428	0.9341451188480276	0.3824327669271901
0.1777804574629369	0.8319591377141949	0.1272178912349812
0.1845979787813956	0.5971419353705334	0.1289535990827742
0.6782055062136491	0.2942618069884874	0.1259473691679348
0.6603679778356882	0.0686893370805660	0.1314172742702887
0.2169615846668487	0.4963647181895148	0.2368109918731604
0.7137958485445788	0.0059386070115778	0.2761901883702108
0.7931875329906498	0.5059704487314621	0.9824782059242113
0.2911834016008659	0.9964195650130730	0.0267358231329061
0.7791510975157554	0.4982587515020613	0.2584584192282762
0.2805633210646097	0.0054902832520716	0.2547757571438822

0.2288215320148192	0.5069062120815754	0.0074373218386979
0.7285779445831719	0.9969412615825066	0.0058841249969874
0.1347922942464788	0.3863554264210260	0.7204037392848067
0.1182755826749467	0.2150423252471987	0.7269138859945181
0.2516094423473012	0.1188537275662169	0.9802076715618380
0.6160800555282581	0.9238687834214331	0.7251474246537672
0.6391377771787565	0.6705397590202078	0.7195084608831173
0.7402506799943893	0.6204642695161829	0.0393352825575392
0.8742484181435323	0.6161563886191354	0.9690319852325670
0.8844534926624825	0.7834709278092026	0.9738200179601565
0.7558125726661333	0.8835147263780272	0.7320277512539036
0.3921533763487364	0.0852689435597393	0.9767274185529554
0.3757735285573132	0.3259432823482074	0.9693860398813861
0.2710513573138342	0.3821621942711150	0.7868520926768781
0.8926518402716451	0.4182703168583765	0.5362159925331945
0.8740656818713133	0.1779609538710225	0.5450589182217449
0.7694518498616570	0.1246072215793180	0.7247661303374656
0.3733264681335660	0.8863783387194439	0.5445153241227637
0.3862950274812086	0.7175508628432290	0.5362568501793921
0.2546414735332612	0.6186101432053090	0.7854064328429670
0.1143563692348854	0.5846145374827123	0.7873553007936513
0.1299384292794435	0.8266845488710086	0.7949657512020744
0.2380247856712807	0.8798247249955475	0.4768507923912758
0.6345859630484807	0.1153384244380875	0.7923643297710709
0.6174877430381491	0.2895957350597618	0.7854413621686090
0.7516022710884438	0.3846955742837292	0.5334226289272105
0.8995126235238949	0.5708331646801359	0.8011910249017820
0.8694045468004329	0.8200495311891451	0.8009912965131892
0.7837024093393443	0.8777651373374592	0.5109801595948740
0.3647705814961523	0.1108526258645581	0.8022899111495603
0.3882098637297393	0.3048412997044010	0.7934369276386545
0.2443091690332317	0.3753838305233427	0.5064419066944574
0.1153688062539620	0.4280354525915837	0.5508627699034659
0.1397679136747475	0.1813068214287512	0.5546773168357391
0.2230794688537164	0.1258596053149291	0.7582553927369867
0.6408992107237954	0.8977189269676580	0.5518847409390636
0.6213683290223740	0.6998222293075658	0.5320041499546522
0.7728491057586633	0.6280368584709153	0.7656091416891941
0.1464872248023613	0.6115457295221919	0.9599663789756887
0.1202718797870048	0.8025075932033576	0.9710512558820451
0.2618284523287702	0.8766077812953240	0.7600811349369148
0.6153834680514176	0.0751488399702304	0.9629313256969082
0.6403564695634080	0.3220877652859677	0.9578206938963405
0.7219434675105771	0.3791554491093126	0.7532373300670508
0.8643752460615943	0.3921091992184705	0.7101952612763758
0.8874597764507420	0.2015554995486714	0.7211613721125070
0.7442065960812291	0.1276686038106207	0.0089380498091826
0.3916195851923944	0.9280447706339149	0.7138940234897559
0.3623703447931905	0.6812559778042812	0.7062692560231660
0.2877859328524897	0.6248284086633519	0.0065118432488962
0.2578895463707337	0.2528889065038560	0.7487279444724477
0.7537772898361405	0.7502100644851237	0.4877669635803783
0.7658026759835733	0.2573802824624692	0.4887923876443904
0.2386042233323547	0.7470459201180898	0.7431049277543121
0.7432714352021231	0.7558516958368712	0.7810104619421213
0.2656978509404093	0.2457067740899557	0.5263454234794457
0.2444930166323010	0.7504263957494146	0.5164201753750058
0.7577597409693777	0.2528492896984756	0.7674776959923978
0.0008553614160172	0.3974509971636033	0.6525772654623310
0.0052409686352216	0.1953488706430919	0.6180356272371741
0.5065085625909256	0.8981218281352567	0.6135617195542726

0.4973613733123894	0.6912736315897218	0.6480791941125369
0.0092447636940819	0.6060941790051408	0.9071818685527030
0.0007613423190867	0.8065068275216879	0.8718601742387737
0.5008688197892260	0.1062655836303987	0.8614273077549033
0.5060433208071964	0.3078530681499603	0.8963734230614536
0.0918839054459149	0.3018320074016494	0.5947853400099050
0.5988282872136574	0.8006467520988789	0.6642970280444459
0.9185554514045410	0.6989880696915138	0.8419175704763705
0.4118879525759568	0.2059676013990107	0.9105338083365848
0.9129935048002820	0.2976375804942012	0.6013575369058902
0.4133814416918080	0.8017103666919219	0.6707665979426775
0.0962165437443814	0.7059724176935875	0.8520669018122351
0.5927824019291872	0.2011606179726921	0.9171543539063509
0.1784817150772978	0.2099636081879345	0.8874661765075241
0.1613928583358057	0.4342872070617397	0.8823055079909581
0.6796367360361052	0.6684455355006618	0.8891671118621012
0.6821867114830338	0.9078611161024498	0.8840922918807659
0.8237457702217479	0.7898693820454865	0.6368166446097129
0.8490351218891373	0.5677974876818539	0.6341347195986817
0.3301853656903007	0.3322003639533873	0.6368207401269776
0.3242804741574061	0.0967996240002076	0.6347280799233394
0.8286773949926552	0.1718987687318692	0.8770654474190387
0.8241353184352973	0.4025942992494437	0.8782629526077458
0.3289377188137281	0.7093634424382490	0.8764339445857063
0.3468821635360663	0.9342105025377619	0.8827567912177071
0.1773075420997832	0.8326220042016265	0.6281999538400562
0.1838767527021131	0.5977162254284163	0.6297102614015594
0.6789403929528879	0.2929761417888930	0.6257007484087667
0.6622323298095879	0.0673545339797975	0.6313217053172765
0.2163221028308881	0.4967622736036919	0.7377126299656503
0.7202991638293149	0.0074867621903628	0.7768368455967872
0.7905349252933968	0.5071167548340482	0.4902258102868914
0.2912648524740322	0.9958419601611088	0.5269532092562795
0.7825288279761288	0.4965845849555856	0.7603808344326044
0.2800961697392989	0.0069153300308243	0.7576205853593626
0.2289650427813760	0.5060386563571526	0.5111579509827622
0.7288960418029259	0.9972694891706114	0.5018382745709609
0.4508297959377898	0.5393714998416822	0.9510846778990444
0.3987755119316216	0.5045765704049227	0.8972681307859832
0.5057859390139048	0.5789166914229743	0.9094471614821271
0.4008263859717662	0.5092626155808053	0.8052478382710501
0.5095095523460782	0.5835310440806478	0.8171797867587384
0.4553990018359313	0.5507067472106244	0.7590902823019142
0.4859871077416111	0.5230543626638448	0.6680323675132991
0.5230167069279804	0.5852742076350018	0.4154334688023969
0.4545462460738836	0.5781650144457611	0.3737694385497260
0.5720371556531086	0.5315856860900197	0.4168285463876933
0.4346121324447609	0.5177325925707772	0.3336590213110397
0.5524058023295138	0.4710960740550901	0.3766725191892905
0.4836961398020495	0.4641040074811495	0.3351416273719187
0.4493275587541916	0.5315661951388997	0.0507975208257526
0.4260280136615546	0.5052649899863991	0.6002969482917619
0.4924477214212357	0.4679828266109268	0.0776379728547179
0.4921977782327345	0.4634388535393403	0.1513924260855915
0.4672575065362693	0.4232473990874806	0.0480531694632489
0.5498029285801944	0.4710894230768872	0.0546093539846947
0.4513377709155799	0.4899329364731760	0.5363670789073427
0.3911086397270792	0.4639642114981316	0.6236637969514950
0.3906950315013517	0.5484213334558563	0.5858499813697061
0.3920888527573037	0.5268301594323039	0.0739296653413188
0.4743873228478794	0.5751441062650305	0.0835250972740236

```
  0.3570236248099534  0.4744162466580528  0.9307637281532914
  0.5449740828411576  0.6058940249738357  0.9515983248986745
  0.3600459143562519  0.4832105455732130  0.7644554541918354
  0.5517285563434634  0.6140226566347504  0.7845260614051826
  0.5384056609212754  0.6323709781024169  0.4468517667081606
  0.4167954294789741  0.6201978242321504  0.3726229037917888
  0.6257241373501259  0.5370235485200308  0.4489170346219632
  0.3808187088022350  0.5124415070774866  0.3016993230649868
  0.5907665413942853  0.4294012757979435  0.3779780902340490
  0.4685140774830803  0.4167363459855425  0.3042616912784489
  0.5218568237645224  0.5620360781257702  0.6402655906696504
  0.5203831922336009  0.4794626529109825  0.6835327919546164
  0.4222610249571327  0.5952938468444938  0.7355849517978136
```

MOR-Al-T4-TS2-Transalkylation

```
   Al     Si     O     C     H
   1.00000000000000
    18.1842269897460938   -0.0090872207656503   -0.0001607598969713
    -0.0105508984997869   20.0691013336181641    0.0015994716668501
    -0.0001797722798074    0.0011982563883066   14.8440780639648438
   Al   Si   O    C    H
     1    95   192    16    21
Direct
  0.5904807153108386  0.7100500100148808  0.6339232752324443
  0.3153902677620545  0.0675326438815796  0.0255587606339707
  0.3041056386963492  0.3122830842950677  0.0288506491804906
  0.8149809610681893  0.5683968951688763  0.0246195111289208
  0.8124240189355184  0.7936628687509819  0.0263890448142637
  0.6940465923667390  0.9201222242254835  0.2754226520354044
  0.7084957234375800  0.6753634851202078  0.2749255662975735
  0.1961669942322638  0.4178155706545882  0.2769570924764931
  0.1952654486742523  0.1930937444820047  0.2765822418401850
  0.6946053883934804  0.0703513511769895  0.2276083469946300
  0.6955105070525545  0.2951405064022675  0.2289552814795505
  0.1929721656074558  0.5672863443681022  0.2276410770687197
  0.2039503659930224  0.8123303644833546  0.2253685555305183
  0.3128205202329905  0.9173146197405313  0.4779204788381214
  0.3111035891273985  0.6941088828457982  0.4781260544656873
  0.8138415898462862  0.4224538655266594  0.4793343558586012
  0.8025084167418240  0.1767840381395689  0.4755923392173243
  0.7106030189217974  0.9118324905906660  0.4837382320413057
  0.7003648384198236  0.6782079844794605  0.4839015412178864
  0.1891816530749453  0.4282823965055480  0.4822968823301201
  0.2090632808245071  0.1815248206577532  0.4833430744377501
  0.2975377720653318  0.0761779902146613  0.2329845401390501
  0.3113826547178345  0.3094975657601796  0.2364762890454272
  0.8213176470498135  0.5595421718207499  0.2273472164697980
  0.8002975253808154  0.8065745856795844  0.2314110661855016
  0.3202242800248306  0.9283902054617342  0.2723684111487714
  0.2965389326728325  0.6833433436553490  0.2710930350407657
  0.7975542991715848  0.4126519059616742  0.2709777734691026
  0.8113832103811246  0.1782512975667470  0.2679893425172112
  0.6878796442265364  0.0591162441162685  0.0224403128033615
  0.7092686285570037  0.3060604208573169  0.0231622030854562
  0.2112833793789286  0.5779628132111335  0.0198047330077992
  0.1975963837535583  0.8100727172617846  0.0184729502177891
  0.0863582772291037  0.3718675130389983  0.1254699931087005
  0.0901092972242886  0.2169429171846734  0.1201955144350256
  0.5910444733566398  0.8679614642258273  0.1323178918985642
```

0.5872585416892488	0.7083301170714507	0.1391757965390630
0.9214770865893818	0.6166209877919696	0.3753836946658857
0.9182373251301555	0.7723253323562204	0.3721214127437392
0.4165941299286621	0.1166444461839633	0.3828069489009890
0.4201044869555260	0.2751908594263809	0.3870394566450682
0.9174272344105697	0.3694529894329506	0.1231372301478772
0.9208593806279166	0.2104237947448992	0.1185941574671697
0.4228600271625400	0.8716977645861265	0.1303904740148350
0.4188757671857723	0.7166465274340879	0.1353478165377850
0.0909981108257539	0.6186595802743933	0.3707663851014634
0.0879052391799421	0.7774739541903133	0.3679792273907974
0.5854583489833662	0.1151239778543422	0.3808191240542891
0.5894548278667008	0.2699704470105023	0.3857576531924293
0.3143817459145388	0.0659872671112565	0.5269864167268902
0.3035328397076349	0.3106905460763805	0.5294641003355594
0.8139558627205754	0.5708677207494068	0.5305422703659272
0.8131644581915971	0.7952422414988003	0.5261504964717554
0.6934020533842263	0.9220441158149109	0.7753266535180190
0.7065423195601277	0.6742186168564389	0.7809991943424646
0.1953332674723468	0.4178794293173821	0.7773088751527041
0.1945356529262672	0.1932663706742801	0.7762334515928755
0.6971644020658506	0.0701589276300624	0.7278953665267983
0.6948621913928091	0.2957647729947527	0.7285875161492037
0.1924775674410291	0.5675416891059009	0.7283810325359444
0.2026914567109118	0.8135757987486693	0.7264123916723386
0.3133645205307353	0.9176862763182938	0.9778466229838251
0.3139964542358955	0.6943116266605952	0.9789622449006736
0.8162111339155775	0.4193092448181764	0.9776226518494782
0.8036265484985777	0.1762234651769432	0.9767806188091755
0.7095090649023933	0.9103643320059855	0.9827312334505788
0.6970300701470287	0.6753559322122636	0.9893815215162484
0.1884272937086140	0.4291321310266975	0.9825230881122587
0.2097284965370950	0.1825730544601474	0.9822370432650301
0.2985176167235605	0.0772916058120185	0.7345797761348343
0.3108854652278343	0.3099342895223538	0.7369184998798268
0.8241569218174267	0.5588846741839538	0.7359736942752448
0.7974183985635827	0.8044346913011123	0.7333187059058239
0.3205059513011672	0.9283946791529937	0.7731521899065541
0.2960788849174694	0.6839988164873614	0.7723778721600515
0.7991395564349321	0.4104000241469100	0.7713421763068922
0.8108545793669701	0.1799889354991422	0.7691109559226921
0.6881920901496165	0.0590720884311384	0.5222307123843442
0.7091990806772285	0.3058861965638315	0.5222274515627764
0.2100160076027717	0.5764654366211802	0.5200476350941488
0.1961061376586004	0.8100725267491958	0.5185904801647009
0.0861640956541960	0.3715316229348914	0.6247337784359991
0.0886549215637129	0.2163361040693688	0.6197496625898238
0.5910293086071893	0.8715556611525775	0.6329138566020329
0.9246607050682297	0.6159371358448388	0.8745515769257818
0.9182440575458287	0.7701116793929824	0.8678148124601901
0.4179062199688228	0.1197237803343837	0.8829991067722285
0.4208816739183862	0.2782377860441773	0.8872808194990457
0.9174040513557241	0.3699385181347187	0.6207547457199307
0.9195036827590692	0.2116678703522407	0.6173734433809125
0.4236960266904204	0.8720106355079569	0.6289535161009768
0.4181640223868393	0.7161842114747481	0.6337976021084819
0.0918424474840772	0.6200333421027237	0.8709693529288783
0.0872851676394802	0.7780179700412055	0.8676977772355374
0.5866465001049898	0.1168988440423553	0.8788416243781835
0.5897054634313968	0.2725653528431041	0.8843922203641746
0.1349254927266521	0.3795687061602382	0.2166656014454236

0.1188453788673974	0.2079923299791838	0.2235797140686962
0.2511435452956284	0.1103002657471241	0.4769369397216939
0.6162873114323740	0.9075846514498087	0.2230805935258915
0.6310410192968728	0.6714932602563164	0.2203482874286681
0.7419456331736692	0.6176886911347435	0.5380738232469320
0.8765274052270614	0.6054687900128922	0.4682312953510104
0.8919980996440424	0.7837789868856251	0.4758528692452603
0.7578708287272636	0.8776785532339323	0.2239853428709292
0.3916880059795606	0.0761676868272472	0.4729672918594238
0.3756604698068517	0.3149312184691253	0.4653889408507962
0.2710575885717300	0.3736080404989376	0.2821561057396737
0.8934482999171053	0.4088186703501211	0.0315845251953504
0.8758071873474037	0.1712258519561496	0.0405584977620933
0.7691648214993497	0.1151501901975659	0.2225748833050757
0.3735442615646686	0.8795986134273106	0.0399034283382673
0.3909850668265026	0.7081811442263433	0.0313591447959391
0.2562099608209670	0.6110904055666389	0.2784896211838301
0.1153015960831776	0.5781533841468633	0.2801198196011864
0.1328872665605990	0.8178092055876575	0.2906840348980072
0.2385495630235362	0.8733992651149686	0.9717545617817038
0.6338317501680735	0.1078708194994754	0.2895393594593873
0.6199663966820994	0.2792572119691272	0.2834204153949098
0.7522094052488199	0.3770905479522433	0.0296775079401048
0.8911799941553341	0.5687399285217624	0.2949762565654475
0.8671847142804957	0.8145497586816145	0.3029703944898474
0.7844867376017556	0.8685412815584056	0.0017284399303583
0.3646402393991053	0.0988811339992844	0.2978420222845770
0.3882409630353031	0.2960553339491941	0.2886774507739286
0.2455403071775492	0.3693120540968117	0.0016921131413045
0.1164604290373046	0.4217817195822883	0.0470914417177176
0.1415117180627803	0.1743827636819305	0.0518187994631693
0.2229440836922733	0.1177114547487700	0.2551815884560600
0.6433103773641018	0.8857533489308231	0.0477336510533953
0.6192608958541714	0.6844012983077679	0.0421067684349742
0.7658803013153491	0.6219887422467962	0.2335033969816771
0.1426889138786325	0.5985069500690118	0.4553827467092277
0.1187107248724275	0.7975972248431241	0.4671557134394381
0.2650106044563977	0.8667729050067490	0.2542855346119585
0.6145019053961834	0.0659673350087989	0.4600896668201284
0.6388125188824032	0.3136549322763079	0.4554396790502087
0.7227796959125005	0.3706966804743161	0.2507040027582881
0.8649430966588250	0.3886802988293037	0.2071876602212832
0.8893239449135628	0.1896970995944810	0.2171913292648479
0.7421172461512656	0.1215743842143689	0.5029342410168308
0.3933181261946860	0.9208623973867551	0.2096901854276304
0.3659983245289737	0.6750121023481348	0.2033643396771532
0.2847132305554797	0.6178556094086490	0.4978499382494383
0.2587336767870296	0.2444107945698270	0.2428554034640585
0.7466137184117019	0.7419402000414003	0.0022803796410774
0.7654768733295200	0.2491108036381078	0.9872001339509354
0.2394582103638231	0.7380634362512063	0.2316482099746345
0.7426042739354167	0.7499306226676256	0.2652309614741856
0.2663822083538713	0.2392928740900757	0.0187827380949092
0.2506455454265178	0.7449533701078983	0.0140203046093610
0.7605411769173055	0.2445218192389749	0.2593089567646662
0.0014829147563788	0.3900858358746064	0.1480638064523157
0.0067000081338141	0.1892266176736794	0.1132305379639759
0.5075279475760347	0.8903072870822278	0.1075420014175536
0.5011993422918264	0.6865867064992479	0.1436597763232331
0.0070403183403459	0.5986348117815000	0.3947325347381049
0.0022701084298692	0.7982147701639347	0.3620390049423199

0.5005559114345179	0.0968620878292136	0.3577258269039627
0.5056910668559976	0.2973224312630932	0.3911449148418020
0.0937015906347200	0.2952212978201364	0.0904254972485953
0.5949072792499318	0.7883903272525601	0.1511566330894609
0.9127901335320115	0.6938478169109122	0.3439989341202788
0.4103343493583292	0.1959378711283557	0.4039715051761384
0.9115534021001761	0.2901081804084709	0.1033979129278970
0.4165775124781878	0.7947637003839649	0.1649235716031036
0.0988726034081379	0.6979441228478946	0.3513412345195415
0.5923024602257174	0.1920027556094835	0.4156855869521517
0.1803009653046421	0.2028605250538862	0.3837035001403195
0.1629474086406700	0.4278911079600133	0.3776092263172370
0.6934904585856942	0.6557520906446741	0.3785495828766766
0.6867958756244570	0.8973373105044946	0.3796046261799069
0.8332233212498960	0.7877998340401839	0.1327433640349817
0.8506119906108879	0.5551570557112704	0.1237434227774416
0.3307117868374052	0.3241831255341509	0.1318130021005491
0.3235194596797994	0.0905796455951979	0.1298432411413094
0.8295522327587673	0.1642156839721220	0.3730505208541832
0.8230740119846950	0.4005489505401791	0.3748324314438555
0.3249778053800076	0.7053319487966559	0.3707192752397142
0.3457662592110720	0.9291210847100470	0.3773070386298078
0.1779080314606585	0.8261846052764105	0.1226747453336560
0.1847121780004796	0.5898477468799483	0.1232207566968805
0.6777083491295808	0.2865588237241445	0.1222337656565543
0.6608007579464676	0.0615641378333553	0.1268907266166347
0.2169085810519141	0.4892766195467338	0.2318954434727423
0.7155229751932010	0.9986786979312388	0.2709995392603941
0.7943718000465589	0.4976921816803977	0.9770473942455459
0.2924016534278730	0.9894188269962734	0.0217636053806047
0.7785541304378710	0.4907524709132756	0.2533521192112453
0.2785021162961623	0.9977977489527141	0.2478667413850658
0.2297340234762508	0.4996133575342712	0.0022399355526556
0.7280011429764478	0.9885330756334268	0.0017080670811679
0.1346826776076181	0.3796971819203966	0.7160272269910963
0.1181964850231699	0.2073866843092174	0.7227859933429823
0.2522005722327341	0.1115483771484640	0.9750699685181061
0.6164059506311302	0.9162118331544347	0.7205069023389999
0.6396318271274990	0.6642393324313944	0.7132258421111605
0.7394929591056627	0.6112059239997080	0.0325312947391873
0.8745423757851259	0.6088571638587987	0.9644787298345922
0.8863392450287325	0.7765309930707009	0.9699097816976155
0.7559336207514805	0.8760779335664267	0.7271339642316483
0.3931287391701921	0.0786555167786727	0.9728715412215306
0.3759059497611701	0.3185355543416610	0.9645454217049921
0.2708792581318651	0.3743677633048519	0.7824011493169472
0.8917568410074750	0.4115502045651898	0.5318688534657878
0.8737165908654611	0.1712857684736719	0.5410460296409695
0.7704657275830975	0.1166874746779029	0.7219292090101729
0.3739966070709254	0.8793091405791873	0.5381674881987299
0.3876880321011192	0.7084601934457437	0.5303656800416056
0.2551137582988786	0.6119024297204775	0.7794279770625347
0.1146652906115362	0.5779337871172026	0.7809812732855701
0.1304480577400103	0.8195214581237233	0.7899527383012889
0.2386503542293395	0.8721427284321472	0.4709862533010450
0.6351497792507577	0.1079422001247490	0.7880403303909814
0.6176793564241030	0.2828044164309982	0.7809149549465623
0.7505787010074507	0.3775344372913594	0.5286165044535320
0.8988762100761397	0.5636743463947709	0.7967929616051683
0.8693994698924160	0.8132892361923176	0.7976845256874578
0.7852175850031795	0.8710201955851122	0.5077932208884173

0.3650315256580759	0.1029773301542031	0.7983558584743681
0.3882312826550539	0.2970713290928726	0.7883579009218842
0.2454763949342145	0.3675166199919603	0.4999968351542038
0.1167226775674854	0.4206089379309890	0.5458869536322540
0.1392721661749184	0.1736080760688741	0.5506170587754167
0.2231192138484487	0.1182539122977941	0.7548033589647504
0.6420893885152366	0.8914070849393023	0.5467040460037037
0.6212948623782319	0.6915841663080120	0.5258167113246706
0.7715309346731456	0.6201608458746332	0.7615199579440817
0.1461538700403242	0.6032168332133359	0.9543062687378749
0.1210528597478399	0.7956688611459343	0.9663061310556432
0.2625580203813489	0.8688729949484045	0.7555368156928816
0.6156872682766330	0.0678365453542359	0.9583901522136931
0.6410279854175329	0.3146575565750106	0.9534893096949973
0.7227951624002393	0.3715498774277858	0.7478543106856865
0.8655371876588432	0.3864394848825961	0.7063629867268765
0.8876228958619720	0.1945896225731518	0.7170540212752440
0.7452591394006991	0.1190218980622005	0.0036901300681724
0.3918201197329989	0.9200402834573569	0.7084103095497878
0.3634499066211591	0.6757110795713510	0.7019790704205908
0.2871664551105387	0.6184264776691026	1.0005524517639888
0.2578201978076199	0.2452666193917382	0.7436172459395374
0.7531993890155455	0.7440326438028068	0.4851860442718960
0.7658063168123450	0.2505516553102253	0.4830547852319687
0.2385985418267232	0.7397123862251528	0.7365156075515451
0.7434145293877787	0.7485960819530246	0.7764133376096818
0.2653182958129848	0.2377182531724646	0.5224793245581532
0.2465780295201228	0.7433897915136113	0.5135694451158254
0.7577370177377371	0.2450059156102691	0.7643548033082341
0.0013286498799084	0.3905671422863840	0.6461677764958638
0.0050158698661227	0.1890673238821935	0.6135229002521057
0.5070920987336284	0.8941968846153729	0.6070800961847684
0.4981195928510734	0.6821282699886190	0.6422205702084005
0.0093502586356298	0.5991500735371242	0.9009889084997724
0.0015581816736529	0.7990526349916640	0.8665762561130448
0.5013949229699964	0.0991252719662526	0.8567559190586101
0.5063293833980944	0.3006152690940132	0.8914577960527643
0.0928239917495844	0.2946587145294106	0.5905350010865219
0.5960661798338516	0.7943241547276593	0.6562300340432463
0.9180805967241147	0.6919616479828259	0.8369980275697969
0.4124078328629785	0.1988654847441933	0.9054774701456346
0.9120359804179415	0.2910338851490152	0.5975082942720805
0.4181385028552679	0.7946319903953316	0.6622220053370886
0.0971860136983794	0.6988230174396995	0.8474229825665681
0.5934147357931909	0.1941082541805161	0.9124114588381241
0.1786747832371632	0.2029016146169637	0.8832355618916240
0.1616737003802240	0.4268481826395969	0.8779744210820012
0.6787742281116074	0.6622812909591914	0.8845802638161976
0.6823305954846015	0.8993879636711745	0.8795649239569825
0.8248563390440659	0.7825420480424589	0.6332879075705633
0.8482906670621257	0.5591962198405880	0.6304774437599429
0.3300520086487944	0.3248297991806386	0.6321380111988139
0.3240741334122914	0.0896764415972480	0.6307876202619618
0.8300607861171647	0.1649935027245922	0.8736172690256858
0.8242601682691044	0.3938391947100230	0.8742877669762374
0.3278745044097081	0.7038282309910113	0.8714518482219245
0.3477567148586933	0.9268553686109210	0.8776059465756978
0.1778892012693065	0.8263095307960844	0.6230441096371496
0.1846502283380443	0.5896962468568563	0.6237559348261900
0.6798760102716658	0.2847554986597987	0.6216941582717096
0.6636456923913042	0.0594740305563378	0.6274613800512389

```
  0.2166089893064329  0.4895519171277320  0.7332666789022165
  0.7204184026726401  0.9997143196269437  0.7735560592114947
  0.7893039927316529  0.4999840585396928  0.4857206147025651
  0.2905787069551584  0.9880751320720312  0.5240329755240765
  0.7815593341875453  0.4891165914218679  0.7581310252594187
  0.2804183022100932  0.9992516546686183  0.7527236767598402
  0.2303899178115962  0.4982345979469102  0.5051048057104898
  0.7307153477637685  0.9905187465000144  0.4972489625808212
  0.4762028378607777  0.5092998902159600  0.9305086468157791
  0.4285385980598044  0.4802646268847295  0.8670270519811060
  0.5502502347212196  0.5202711993352561  0.9062341835841069
  0.4538874985422294  0.4622077914214587  0.7819593334313640
  0.5763600301560232  0.5021439943281591  0.8218191096279361
  0.5279633848378419  0.4737477596148026  0.7579849660533815
  0.4972861948854289  0.5733592588962438  0.6762551377898909
  0.5334394168082545  0.5847512106001785  0.3883523015715511
  0.4588339849576766  0.5837570874552134  0.3657934303810672
  0.5697121352981173  0.5254160617128418  0.4097495689195207
  0.4199094224260231  0.5236252464914056  0.3647589447855364
  0.5309509669609648  0.4651304914055654  0.4089299282849583
  0.4561268429064074  0.4643674379463041  0.3865007280758336
  0.4500053711988420  0.5260591748269707  0.0238901703884714
  0.4528725985116733  0.5471228726043915  0.6015841065404398
  0.4717686368306801  0.4716966074070175  0.0921561905209656
  0.4535054427355171  0.4854945870016585  0.1602725527327682
  0.4468936903030814  0.4235302823638619  0.0741813107934654
  0.5318118673995526  0.4651759308371854  0.0937768218441497
  0.4750371294353459  0.4996372527652678  0.5762106004002367
  0.3944924721406404  0.5420888887785769  0.6182602376745352
  0.4593212438189584  0.5807043505264959  0.5430161034425542
  0.3899424979695134  0.5321689866886736  0.0234626325592488
  0.4742516171328438  0.5738694610451461  0.0450728245823303
  0.3711592362944405  0.4716420044030636  0.8852560857061509
  0.5873369711969928  0.5432301034836714  0.9550616704861983
  0.4164764616712924  0.4395909245696147  0.7333336046631846
  0.6339442539445398  0.5102421464659445  0.8042005193559787
  0.5629973120230269  0.6318266111881891  0.3897689060096395
  0.4315972787004375  0.6303326072928075  0.3488139690596264
  0.6281322786863581  0.5262801485500468  0.4267491580974591
  0.3616351543365874  0.5230904288684988  0.3472069212845335
  0.5589107716484876  0.4186860053353085  0.4255363620868355
  0.4257778410172057  0.4175987254575629  0.3861250250623373
  0.5567424058705228  0.5775596342640040  0.6703174885550732
  0.4730312922461506  0.5908590327006226  0.7387724947160516
  0.5496463846832934  0.4542378791596651  0.6947852370612176
```

MOR-Al-T4-Et+EB+Benzene

```
   Al     Si     O     C     H
   1.00000000000000
    18.1842269897460938   -0.0090872207656503   -0.0001607598969713
    -0.0105508984997869   20.0691013336181641    0.0015994716668501
    -0.0001797722798074    0.0011982563883066   14.8440780639648438
   Al   Si   O    C    H
     1    95   192    16    21
Direct
  0.5928155592780711  0.7207201139944651  0.6328665475429360
  0.3133379047694135  0.0739358707311117  0.0254800101140986
  0.3002865659646973  0.3193151834458731  0.0294455386794503
  0.8139957778651338  0.5773701369859471  0.0241261095134442
```

0.8134902017814155	0.8035421449982866	0.0260814557497741
0.6946005114199610	0.9302576206594620	0.2753290151891234
0.7085939454830223	0.6848843844286877	0.2743513734941262
0.1946528296129804	0.4259140989104859	0.2763725934848371
0.1941154276225551	0.2011209490936847	0.2754140797418664
0.6944081735291346	0.0796466295379799	0.2276247646125281
0.6942117340128636	0.3051896770092055	0.2298885656316224
0.1916137261979614	0.5757332787291365	0.2278585464130912
0.2041609476900317	0.8205769173169731	0.2260016725983326
0.3124755968288158	0.9250218307753951	0.4784415953682455
0.3077334187111769	0.7018046772995560	0.4772890717610550
0.8117981047393193	0.4317965092415910	0.4809527485225530
0.8011044278657429	0.1857995469433384	0.4771672458344587
0.7118644263952264	0.9212281234313424	0.4833354941922660
0.6993563498744290	0.6879003840582664	0.4832220457269573
0.1865005329663943	0.4355290156011222	0.4814967041838961
0.2075274438768197	0.1891861969341634	0.4820028101758822
0.2972797553140865	0.0842247630593075	0.2333537746307845
0.3101692472689579	0.3181476159876417	0.2364560306985850
0.8206798724228519	0.5685929081163551	0.2266868811530474
0.7998669029904136	0.8162333773459259	0.2307624062266976
0.3202530719229332	0.9365492819686992	0.2736462325088547
0.2969979830148891	0.6911052703504995	0.2710551435259551
0.7959528360504565	0.4225171983100692	0.2719908406987639
0.8094694345289818	0.1885072264989924	0.2695464023329180
0.6866041908457069	0.0677198851375859	0.0223429247670937
0.7077118683188884	0.3150457345423732	0.0231399961136116
0.2104519619946590	0.5852575313189622	0.0207213418519774
0.1974067122052132	0.8165489312953040	0.0199205223335220
0.0841412821594337	0.3796128146800686	0.1247857659725804
0.0873913599271248	0.2246103656002604	0.1193753486115516
0.5911076579630751	0.8775965919242831	0.1341553537917630
0.5875380303584858	0.7188985742188765	0.1408859002971305
0.9199370577151008	0.6255829239592350	0.3744298407841757
0.9171775761486216	0.7808753886039141	0.3709840431899020
0.4158434466589701	0.1260388772167722	0.3826625337342110
0.4186730212363869	0.2843072648383493	0.3866438320665765
0.9155067092241613	0.3786595158839070	0.1241048596205201
0.9180393507083990	0.2198655869560312	0.1197915088526750
0.4225965887133862	0.8803819335738368	0.1307563729510544
0.4185721417627206	0.7254051437240092	0.1354990153934099
0.0894746929684164	0.6273301456067110	0.3712613664288370
0.0868634806235832	0.7863089890965975	0.3679787316902398
0.5850682236304622	0.1240921563235538	0.3811893300807970
0.5886446676714806	0.2790371757116197	0.3860646032999671
0.3135119851240630	0.0745206249852381	0.5259648242278295
0.3017507629900927	0.3185756406831557	0.5286952458050039
0.8124944839964355	0.5806962038340444	0.5308448627197166
0.8129756241125216	0.8044580661494576	0.5259967064680219
0.6926626858682375	0.9304185838471080	0.7758241613945144
0.7059456653017880	0.6831412905831645	0.7823167117534664
0.1941194241259756	0.4252186526367167	0.7766203273013509
0.1942220814613456	0.1999938982953826	0.7758622189741042
0.6961237193344879	0.0785445758387544	0.7287153698623406
0.6926818551914173	0.3044565717609818	0.7304826807804520
0.1918099464460236	0.5747822852740063	0.7285391214190220
0.2029255213533855	0.8213827436587599	0.7259674392137612
0.3129238916154765	0.9246948776314545	0.9774664162921771
0.3124807344823012	0.7007741489417432	0.9819023228030108
0.8152367968502777	0.4284328912401740	0.9776252129813401
0.8017413259427175	0.1852865571722435	0.9764736915801938

0.7086148803919307	0.9189019349170113	0.9829419802283497
0.6975181736443193	0.6854705556129308	0.9899935649646221
0.1852756299319879	0.4364461629501712	0.9818538928730089
0.2071553640755568	0.1895617314553744	0.9828344051676545
0.2976680244319913	0.0844747448583921	0.7332407860538549
0.3098231316415988	0.3167956205033997	0.7362425931152280
0.8236149218531712	0.5676733140483463	0.7358796881511805
0.7979660495988921	0.8132700006613900	0.7334948502689783
0.3214164866814675	0.9357013444276150	0.7723213222537770
0.2946617024597138	0.6922471002393492	0.7745551184529414
0.7986584372786686	0.4178075294743144	0.7716231807826104
0.8094551683835624	0.1888523903902597	0.7696222863167974
0.6871677031755825	0.0680113717915677	0.5230639356836305
0.7076406864059808	0.3149328719665456	0.5238781432548715
0.2074757571308004	0.5836577897850299	0.5202699422313265
0.1940063535140539	0.8190219252611124	0.5181871927146177
0.0840788909422008	0.3789477546492779	0.6243891862443468
0.0875204528111141	0.2234487096564558	0.6198542892550298
0.5904494007569554	0.8819668788817876	0.6311546723075465
0.9242786123953184	0.6253580369581405	0.8748874770562373
0.9189438203703841	0.7798237727719413	0.8679316459883003
0.4161133667251202	0.1264680476980471	0.8825998475466995
0.4181686175217089	0.2848878860022124	0.8873777033909416
0.9159412076761124	0.3771884929486869	0.6205758990584910
0.9187064136017736	0.2196365591024022	0.6171926351400550
0.4256186372490926	0.8811856929876410	0.6277477032747411
0.4149700279425789	0.7255315507347740	0.6338783371752347
0.0918112646261843	0.6286243761630347	0.8716026435432761
0.0878962053038385	0.7862478355691381	0.8680133049376337
0.5851426652874704	0.1249702111573549	0.8794880592614009
0.5873332290054386	0.2805997587701479	0.8860014515886467
0.1344772982553427	0.3877373371385389	0.2144891589403022
0.1178395407625020	0.2154547893827449	0.2219652606337136
0.2497203832524246	0.1178792111066052	0.4756048368970767
0.6163987301004676	0.9190608187521392	0.2234013537184199
0.6306481442174821	0.6799496247003128	0.2208516872805991
0.7406144420727753	0.6279046251583985	0.5384165767337483
0.8740188638802010	0.6149886045590559	0.4666214109972299
0.8910917060527780	0.7911351704943234	0.4750772840507891
0.7573897553679718	0.8872525985414691	0.2229015513628133
0.3908595202454659	0.0849296598271246	0.4723112863108669
0.3739127658456921	0.3239760961477602	0.4647744434990151
0.2697788665948188	0.3821140830646651	0.2824340398589095
0.8920156094561096	0.4185803711124477	0.0328936727418139
0.8725010819733374	0.1802759230893851	0.0425767582580046
0.7676155232045639	0.1260446152907919	0.2220625462225535
0.3744329800889358	0.8884257473202825	0.0394298485305258
0.3899232326656524	0.7170392451150023	0.0320186245334110
0.2553542791294614	0.6195358484699098	0.2778161945138317
0.1141238244950302	0.5872072785737338	0.2806167234752713
0.1328481376142481	0.8258555108295413	0.2909647527521217
0.2399007578254674	0.8781125144400250	0.9708411579518264
0.6328906185317831	0.1158401929410758	0.2897154325377016
0.6183000608416055	0.2892499484512516	0.2834866576604173
0.7510140278914100	0.3858743850934017	0.0285945602317305
0.8908319326789063	0.5772544948858563	0.2939603732071242
0.8660645870847891	0.8241586324613777	0.3032001918970677
0.7842665826455902	0.8779675151457741	0.0013304888935203
0.3645065262420763	0.1080475036340838	0.2971583783800510
0.3873337782452391	0.3048294501013504	0.2879434982830146
0.2412839807774932	0.3757982014426109	0.0018249197468354

0.1123498808950263	0.4290708399860680	0.0449447975166478
0.1369660718833567	0.1814974938806949	0.0494941622281936
0.2225732503739878	0.1260140892035108	0.2536737429832936
0.6431024367202546	0.8935284068975262	0.0485569795936539
0.6206995725723543	0.6975171675780137	0.0435485677394623
0.7662445484268441	0.6318129423605663	0.2332176548333277
0.1402720322142570	0.6063930113113424	0.4561679877274095
0.1161431931816830	0.8080710411964201	0.4673417264208575
0.2648362409709528	0.8751731667946735	0.2554457029616797
0.6136295267832550	0.0746087273027893	0.4605830985857536
0.6378683490876871	0.3223247906097678	0.4562112294463490
0.7210188347602500	0.3809277803924060	0.2512612477646020
0.8633075415046918	0.3981581316745084	0.2083789644589316
0.8874869302952265	0.2002249391574760	0.2191924449144094
0.7402610150826123	0.1310935781299872	0.5048918618319601
0.3926095791483364	0.9297473359402091	0.2096069723302512
0.3669631317934070	0.6829201830913367	0.2041498129995085
0.2819003187617394	0.6258334953299831	0.4983807680729151
0.2574904837955136	0.2530539798718984	0.2435327016150736
0.7490632970183005	0.7509301594856407	0.0008863167633433
0.7630515529264295	0.2579370640770696	0.9856358023742359
0.2403749508308325	0.7466244383411172	0.2324572486348998
0.7419327746477482	0.7596062352906513	0.2641461371653191
0.2626351371905912	0.2460237361973449	0.0225362380563115
0.2487431652791513	0.7503696024574830	0.0193652171521597
0.7590214338756501	0.2550571900740536	0.2625764356966749
0.9997532352876936	0.3979701881147505	0.1498253897527890
0.0035894832671783	0.1978209466942015	0.1142298450451367
0.5074496726067179	0.8989562608369921	0.1088525512541133
0.5016103537522297	0.6969577993168420	0.1437770441039051
0.0053267035336590	0.6079877498593336	0.3949355902120299
0.0011785293319927	0.8066499435720099	0.3604483915870328
0.4999722619861653	0.1066393978185397	0.3582417181787139
0.5044964466967337	0.3048200268216839	0.3916706334365376
0.0910905831699740	0.3028958039493311	0.0900113984417429
0.5953142002174864	0.7985458048317717	0.1566643114268748
0.9112312159867576	0.7026456368684466	0.3419192774794778
0.4081769209801456	0.2050549155168615	0.4040700812385539
0.9087069608551385	0.2994370448106481	0.1034894142514856
0.4155141332680454	0.8035331824637629	0.1653805226501592
0.0983586465059088	0.7067195755497845	0.3531230628378647
0.5931314989348435	0.2009075506995726	0.4154673295839198
0.1781131668531011	0.2100385118833391	0.3825154363720911
0.1604278156394082	0.4350228568398021	0.3767603333700338
0.6935353316105475	0.6656107782495044	0.3780966511885774
0.6876783294254132	0.9068493374940430	0.3793674021889611
0.8336443179956106	0.7975957220426696	0.1325975723338330
0.8496418434353521	0.5637542268496599	0.1231709885699415
0.3285289101349100	0.3326663687767831	0.1314871060496484
0.3232315491471521	0.0962747717677834	0.1297439261014799
0.8271068721525717	0.1729973461528044	0.3742925349804728
0.8210366330893004	0.4107057917659251	0.3760448155887514
0.3255897079221380	0.7120590642951868	0.3711144917446855
0.3468523185155800	0.9355896740191043	0.3783685525275551
0.1778932244775116	0.8350278027509744	0.1234900354746645
0.1826731032763448	0.5979800319736128	0.1235839592173257
0.6779728492309821	0.2954926853667397	0.1230526865463022
0.6605245857288292	0.0700221585933247	0.1271515898682343
0.2153519496338900	0.4978214086592365	0.2328544374623480
0.7172930458078720	0.0085150372377432	0.2714184125590230
0.7932558960969260	0.5068350226521341	0.9764473245853531

0.2895883217580753	0.9959024515993636	0.0212279424806981
0.7770113890737703	0.5005729914266904	0.2537018160190485
0.2782361597227997	0.0061802818602927	0.2516638061866097
0.2259140086843089	0.5066850059688608	0.0033927879161177
0.7266632888516521	0.9971142536354534	0.0014980398489130
0.1333364778222477	0.3878218684020833	0.7147929466249054
0.1179370558329645	0.2143018352569966	0.7224448192405685
0.2497469655732009	0.1184891310411822	0.9767768186930502
0.6158460132110271	0.9245368043048071	0.7202347136862645
0.6380484757722549	0.6760802433206531	0.7148839212175923
0.7389345153891481	0.6207990947753266	0.0328470306311018
0.8738656957206490	0.6179610155671673	0.9645088460284947
0.8880969880731507	0.7876436975706741	0.9703057739974007
0.7550376418048514	0.8842692399608640	0.7276509810915021
0.3906380917598737	0.0847225255575675	0.9716213334457608
0.3712139016122956	0.3240673121326061	0.9636821196327436
0.2685392744709992	0.3803436978784859	0.7821901511137850
0.8897202635056433	0.4203960383687981	0.5333189073058461
0.8727836221764042	0.1786604674974623	0.5415518899676801
0.7694997860378506	0.1249723308629590	0.7230009151819762
0.3721864930700846	0.8858286975230338	0.5402188560264459
0.3830501206663980	0.7176294178694133	0.5327543451388316
0.2555646840404094	0.6195558773830933	0.7770409868304450
0.1147465380682391	0.5857267421298558	0.7823546261451764
0.1322693829787609	0.8282072041648968	0.7916341527389995
0.2377239995951122	0.8807353539697195	0.4714125899966833
0.6339140526680640	0.1159120850978995	0.7888774812443201
0.6154422032293866	0.2913405857867576	0.7826950196927766
0.7486103675630253	0.3868037302435786	0.5299275302313694
0.8982701736398619	0.5736883353322222	0.7966478040941430
0.8698805505623214	0.8228358027303169	0.7976661894685099
0.7867423724379805	0.8807545913877257	0.5071581864197102
0.3643817826111633	0.1092633223415672	0.7971155274073227
0.3871455116877620	0.3043079602355796	0.7878959526020967
0.2431831435852162	0.3751430314311767	0.4997044364679876
0.1138649620020117	0.4276289146703507	0.5446018246565003
0.1380343189202264	0.1813167938553089	0.5498570267166940
0.2220244194042016	0.1244455740484598	0.7553452597521600
0.6438801736759288	0.8992825122894264	0.5466429934857786
0.6195977781741863	0.7004130733958657	0.5247675298454413
0.7686739847523723	0.6271876489693828	0.7627340622747638
0.1465531644493940	0.6127718524797048	0.9546240721898204
0.1208130156253105	0.8027476143400306	0.9673855904028812
0.2649506805665811	0.8752018484469024	0.7533176467446939
0.6138186555798330	0.0757490190678338	0.9591628185990109
0.6380182003524808	0.3229739703721502	0.9555461880628944
0.7213437658505040	0.3797722237658717	0.7510297062345052
0.8635369230763865	0.3904709653984264	0.7065898926979086
0.8856369236138939	0.2045274959667333	0.7168700231406155
0.7433175910281559	0.1277252744841436	0.0019466913543298
0.3941534360968985	0.9264947301740043	0.7098851317131127
0.3660013955990213	0.6872135173343136	0.7084517602528448
0.2872199040053953	0.6247030640938472	0.0030171476911020
0.2579953743666569	0.2511341934375723	0.7425504980436731
0.7505719341643949	0.7547513677719788	0.4866219756183588
0.7653202512396449	0.2598214022632707	0.4860675854796507
0.2381895658897871	0.7469689271507837	0.7337999542868828
0.7447966748475291	0.7566608349529919	0.7760823134549152
0.2639805541864416	0.2454306723520210	0.5204482488618322
0.2430164547872354	0.7514856433226562	0.5105655563035078
0.7549997238390940	0.2528508676927057	0.7656835786670146

0.9995468190946961	0.3984539663341604	0.6467661434463962
0.0039148865964417	0.1957905121196519	0.6148644586808293
0.5074676992346419	0.9065457078796714	0.6032157404019012
0.4969598701170041	0.6862237978220261	0.6372487997558598
0.0089886386476094	0.6085284636421618	0.9013877374839449
0.0024803070667496	0.8081644367579298	0.8653652926721259
0.5000426121254010	0.1067810252860769	0.8571762078930975
0.5034759119523660	0.3072854953937063	0.8938950699959648
0.0906472336092154	0.3018893191571407	0.5910296250249972
0.5901791455787356	0.8042514219348469	0.6540488524498734
0.9176748155636298	0.7015756728836784	0.8379767777132812
0.4094238589860701	0.2054270904267143	0.9050766651727854
0.9127396786819393	0.2986703054370968	0.5943303708113556
0.4269473522796793	0.8026877364673579	0.6596902298656560
0.0973291460499977	0.7072361441031870	0.8463141277993023
0.5919240195554835	0.2019936051245573	0.9133519557869622
0.1787960895391556	0.2103463322244826	0.8828062772180526
0.1598756491606144	0.4346250264750419	0.8768404037609617
0.6774553126473574	0.6729362646114576	0.8856085191217645
0.6809307956790844	0.9075170070197730	0.8798816192889304
0.8251815071133975	0.7920584842478039	0.6330819093781808
0.8479251552001726	0.5708465521853229	0.6304608371742890
0.3281879404583939	0.3319492660414204	0.6314224010905926
0.3225819833259680	0.0985302249856653	0.6296605199160842
0.8299284565210270	0.1740747712591513	0.8739567341032327
0.8248581653744603	0.4028608639625818	0.8746140537900486
0.3228134012045361	0.7131050905551934	0.8742240386708989
0.3470472102546905	0.9350376856170285	0.8772231778123840
0.1768316701727571	0.8344491649172951	0.6231464688669160
0.1824879998678764	0.5967637806523143	0.6240363779146454
0.6780430161619099	0.2940478483668138	0.6233214578159066
0.6625469637566360	0.0673696127056631	0.6283222475742029
0.2162792758162738	0.4969857922141842	0.7332366865323080
0.7195609903442638	0.0079972601354998	0.7742157113157908
0.7874968798141544	0.5093691305614668	0.4881216505888976
0.2912184304153155	0.9961219908932829	0.5239114530950932
0.7837826624831317	0.4964918412798415	0.7553609210950522
0.2809497591957705	0.0057856314908415	0.7490712413736622
0.2275789531970864	0.5056260597721265	0.5043345779004668
0.7306363641850788	0.9999423553141570	0.4976908182088686
0.4834771645996801	0.5259609694948469	0.0184692171469957
0.4378954307800311	0.4749179150201405	0.9859052575631042
0.4906728991555093	0.5840740382302543	0.9667474953067594
0.4007136317836192	0.4815097526764152	0.9040284539305695
0.4532235772244303	0.5911408148119546	0.8851218090963033
0.4082568307675398	0.5397769659497890	0.8531534202794439
0.4989386822750627	0.6103558412304846	0.6443391139950965
0.5465739450002876	0.5568251234850616	0.3729692094832537
0.4802066349683798	0.5745201771060265	0.3317270894948287
0.5508260220425075	0.4988117225629395	0.4249359098229972
0.4180820428054099	0.5341005182401016	0.3422946608749828
0.4885451714668311	0.4586859410079208	0.4360512036955566
0.4222721494254579	0.4762494269821250	0.3946013302066886
0.5257008182819588	0.5173762693372952	0.1051760025146301
0.4425968589917522	0.5762967172771706	0.5861605377636184
0.6011557835560130	0.4848715767189363	0.0896754769894250
0.6307282083022576	0.4782212788338500	0.1537011177531393
0.5946626805218459	0.4357891190587880	0.0576291519045144
0.6353265568003914	0.5157097952176489	0.0448967886336858
0.4517048058644966	0.5224456383691810	0.5936932794218169
0.3859933529272825	0.5868213327788088	0.6071988269897111

```
  0.4495864724623264  0.5882859088104190  0.5145653447546944
  0.4934884793303497  0.4866040561413821  0.1523047990611290
  0.5331847406882011  0.5661361877013027  0.1377921404133839
  0.4317662585737029  0.4293394371890644  0.0253423409254409
  0.5256351146567171  0.6244026844259530  0.9908293432963551
  0.3654652232350155  0.4412743198105549  0.8800294122165103
  0.4588473545057644  0.6374962573135387  0.8472156422372010
  0.5947880262928636  0.5884093793241364  0.3646157315953080
  0.4771801115739930  0.6194675053258686  0.2906407989388293
  0.6027092608231406  0.4849174571100398  0.4568547446605873
  0.3663936570737006  0.5475177032090047  0.3097869230865264
  0.4915913622990218  0.4136719697967632  0.4768930479283925
  0.3738725599145377  0.4447697687393860  0.4030809242684276
  0.5548610092792408  0.5972873870702378  0.6231032284222070
  0.4923900410006110  0.5997587140923413  0.7162749085189493
  0.3784524680642213  0.5451994184552093  0.7897925231562438
```

MOR-Al-T4-TS3-Transalkylation

```
   Al     Si     O     C     H
   1.00000000000000
    18.1842269897460938   -0.0090872207656503   -0.0001607598969713
    -0.0105508984997869   20.0691013336181641    0.0015994716668501
    -0.0001797722798074    0.0011982563883066   14.8440780639648438
   Al   Si   O    C    H
     1    95   192    16    21
Direct
  0.5791869159505700  0.7268951299240189  0.6475467872600785
  0.3084583489661188  0.0843463858347237  0.0297400620691029
  0.2957990521723604  0.3292472135647815  0.0343926875147336
  0.8054184623629859  0.5854688608919347  0.0376519774416494
  0.8047155409894996  0.8115019473019393  0.0362914922777728
  0.6814587863382074  0.9361784736905074  0.2860037623552495
  0.6962505467229496  0.6893150927159559  0.2857431804037769
  0.1883248458637420  0.4348176602507429  0.2811161110784133
  0.1889121482758077  0.2112889532036120  0.2814281371401612
  0.6876384062544839  0.0867864393474211  0.2384570729109041
  0.6879553584138128  0.3095759994853357  0.2386573225351231
  0.1816025488003167  0.5846736343754932  0.2319546484094432
  0.1939784055152742  0.8291112708135573  0.2287962478641141
  0.3050187012763847  0.9329386737762502  0.4810013701885023
  0.2999354796234739  0.7106958783279151  0.4820232793736251
  0.8069397603570535  0.4369955191213796  0.4905331081184525
  0.7948305010008118  0.1921887223376182  0.4855143603713218
  0.6994575495104849  0.9269347554384186  0.4940040444807170
  0.6914793216267392  0.6940891623920946  0.4936643242645368
  0.1797272749078095  0.4447767396168778  0.4872747053492747
  0.2016698700470011  0.1981924322591554  0.4877288352615466
  0.2922147521024058  0.0942543857272678  0.2373799831343510
  0.3051595929757859  0.3285393405004098  0.2418132498345901
  0.8126911527813071  0.5743529479507055  0.2404900419504454
  0.7879636025432324  0.8213992603462466  0.2418736307194166
  0.3108520692819599  0.9451599886081103  0.2754800185709224
  0.2875631547420885  0.6992997553925989  0.2754504184370458
  0.7914248584487666  0.4261301792353916  0.2825324582205382
  0.8044889897518354  0.1929222922692957  0.2780192605181863
  0.6780165739727235  0.0759093947316555  0.0335314726452211
  0.7009167790886801  0.3215315351494993  0.0329583100112852
  0.2004379175745837  0.5954084534408827  0.0247809923809659
  0.1884661036574750  0.8264701384960180  0.0217633369606269
```

0.0785266821325612	0.3869231987710673	0.1307569100396468
0.0817082600924896	0.2323680036427447	0.1253898304601097
0.5812578000081178	0.8829777367088411	0.1428009228172790
0.5780599209324844	0.7247461676601845	0.1483032691319809
0.9129349285709507	0.6331456578853090	0.3863522031884681
0.9080196687810744	0.7889153041520245	0.3811418442076361
0.4101725939286516	0.1351480983051981	0.3878924249224906
0.4127151552216116	0.2940654688080979	0.3924931735819140
0.9099467062475894	0.3850340565191588	0.1329720942803258
0.9125989190650051	0.2263234220887721	0.1278326413033131
0.4128853642186776	0.8865477484869002	0.1337891357474035
0.4093019745093541	0.7316803290032652	0.1398593237936173
0.0820392538441411	0.6361789290523380	0.3768500958299509
0.0772978945474636	0.7951991897711570	0.3717236164121216
0.5787372593878886	0.1334445263794937	0.3913584636531711
0.5815990732129892	0.2878731185049884	0.3954770792274044
0.3066387768308961	0.0824071818802995	0.5300093647273206
0.2951141377347790	0.3277380416931604	0.5341467027643763
0.8051748270727282	0.5856898277738817	0.5392798144702712
0.8047734713275496	0.8108686550739668	0.5345392064253411
0.6830479309220384	0.9378092828023676	0.7865684602606469
0.6942960725277533	0.6885201731853942	0.7905617320261089
0.1888258180810642	0.4346552570648975	0.7817624604304660
0.1883485523686843	0.2119641092848845	0.7821743066651565
0.6906419955428740	0.0856680977880815	0.7386817166634396
0.6886261026746466	0.3094782497860165	0.7378102457107145
0.1821722003771437	0.5846007401776580	0.7337379226598595
0.1923234590448682	0.8301039284906816	0.7305684860748184
0.3043340428531566	0.9344297660724201	0.9819178471331294
0.3053829660723595	0.7103985968291627	0.9833315474529289
0.8064976660962431	0.4364631768963190	0.9902373788128714
0.7948151871610949	0.1919763189914987	0.9859461203059450
0.7001761177634542	0.9264557817719105	0.9943205711407676
0.6899492999032486	0.6932014422458362	0.9996538466462200
0.1792451891672885	0.4454324499255026	0.9873285206498823
0.2015874500963917	0.1994751318737781	0.9882320340925976
0.2904058367865288	0.0938318214206210	0.7383060192424771
0.3052357641941060	0.3285257108961175	0.7420538566282069
0.8126739485290361	0.5741560575880945	0.7444331877891632
0.7856769038681073	0.8193800926801811	0.7424305966108041
0.3090146588362653	0.9458664920540368	0.7771120313536868
0.2864118232125424	0.7006741894032515	0.7775420067384055
0.7914833662896789	0.4258089991514826	0.7827537186309671
0.8054209244054427	0.1938696504375558	0.7782619782168236
0.6793912209811163	0.0751821251404400	0.5331777511736755
0.7013552167250030	0.3214077347654873	0.5318706083805214
0.2015358451599623	0.5930774045639907	0.5259020122183022
0.1853899630192323	0.8265767611960886	0.5214186043675070
0.0789467561184715	0.3874803366993603	0.6301084951994780
0.0819522516227990	0.2330607690840967	0.6249937239653081
0.5807219862611741	0.8876569583540898	0.6454599885800147
0.9135876833867130	0.6322881101902923	0.8848499223980685
0.9079491854418106	0.7872338513461730	0.8778381936992776
0.4107014533898972	0.1354723913352058	0.8856059141835273
0.4132583063677991	0.2944752902941602	0.8915026433199493
0.9103721257968940	0.3854096633263945	0.6333423355424647
0.9128669696670243	0.2269332813554199	0.6275961968538644
0.4137138580211486	0.8897104982338966	0.6346223926973389
0.4062839258051521	0.7353457212439795	0.6385991515845010
0.0809372484604610	0.6365511411960766	0.8758695777287415
0.0767763495367784	0.7943220225593554	0.8706790309588450

0.5792327897514110	0.1328601970559179	0.8886395903696582
0.5822069379602535	0.2876501122849665	0.8944629231466995
0.1292295517179897	0.3939908747476030	0.2205085086711892
0.1124761413596048	0.2249265472374511	0.2280219287869255
0.2434833856698715	0.1269314922895734	0.4801790305409288
0.6038939342006120	0.9255575755005244	0.2323548584867146
0.6208597595482275	0.6839218753892292	0.2270081305158146
0.7320027543026286	0.6312925623806960	0.5442430622240785
0.8684477940494421	0.6215058130114108	0.4793709895476183
0.8848240768278866	0.8014577174521570	0.4859232691106800
0.7441193097455628	0.8918064422391242	0.2354246979721091
0.3842117653895969	0.0940101563865133	0.4770757564738122
0.3664921515798417	0.3339723267526047	0.4691141961883055
0.2653715744221471	0.3935305984285731	0.2863417313775659
0.8839775476684226	0.4263076941839237	0.0438711293265948
0.8658815328633805	0.1867827757846230	0.0515701714328117
0.7643570370744345	0.1286165394947092	0.2326348969643129
0.3651894353900348	0.8955210019038137	0.0421115416910883
0.3824847166650400	0.7232413563744127	0.0356815401166113
0.2449468301575322	0.6281822002308807	0.2828036830618185
0.1042251337702357	0.5956700931453692	0.2853531739779124
0.1223832437712924	0.8341650961566884	0.2934053021935197
0.2287183935515062	0.8911151722931114	0.9773085731492995
0.6299934372583462	0.1266705791461292	0.3023600173259725
0.6118580551249572	0.2954972056295914	0.2928377512499625
0.7433798784643521	0.3926527705682117	0.0413305218741267
0.8831498738783717	0.5843711979967150	0.3067509544880724
0.8541091676057029	0.8300662327754926	0.3142079007620681
0.7764670086588022	0.8863985320073990	0.0124338936779377
0.3596246521616707	0.1173111326665576	0.3015969922853713
0.3824536746391505	0.3151344356127663	0.2933360171683492
0.2378155236780584	0.3869524807267913	0.0078340069414957
0.1070056808539174	0.4365994536992784	0.0511775929356592
0.1323239415179414	0.1897541529152572	0.0560798317597351
0.2181302470050274	0.1366602937134519	0.2588349002639806
0.6353564736574187	0.8990513077116939	0.0592949001762128
0.6123364744396466	0.7061015104319937	0.0506314187615657
0.7546915332189860	0.6343561372849391	0.2510693913747964
0.1361490410704220	0.6158653703409845	0.4591193976168033
0.1072500450577161	0.8179751222458574	0.4703312647527678
0.2527273868100091	0.8860426732809406	0.2561258203632951
0.6056211952117760	0.0840404131553965	0.4715904928472563
0.6316368289880586	0.3315674299603482	0.4645638226479071
0.7165739069239913	0.3846675618263973	0.2607114400162386
0.8591614558225251	0.4021475222382057	0.2194204148596277
0.8820766141857183	0.2066723747089705	0.2273575107000762
0.7351751345622425	0.1362721389361645	0.5129423403294260
0.3831363342053611	0.9366326846293522	0.2118645047267984
0.3565503530173175	0.6895146595691913	0.2075711093958185
0.2776093427874776	0.6336118753142905	0.5055957932443786
0.2516953580312281	0.2641730585134812	0.2505557012931584
0.7408453262735692	0.7593407127841757	0.0071357854819243
0.7569419750492055	0.2651571646258413	0.9947692769938024
0.2320147626407975	0.7560386319591446	0.2378377036504568
0.7315664096063021	0.7632101991740763	0.2739770110502306
0.2565545787213149	0.2566324876797376	0.0273068460868624
0.2431594066681471	0.7626602003584390	0.0173873242781628
0.7515433353316026	0.2577872862141974	0.2694516079235991
0.9943146472721967	0.4056214154936260	0.1563587665957748
0.9982327681116884	0.2043402991058288	0.1212601663210858
0.4983198843285330	0.9036444115416000	0.1130017226964028

0.4920642481978286	0.7034487100682590	0.1504018757256378
0.9988887171564135	0.6162106337519567	0.4048491649615628
0.9913912502064508	0.8151186164232233	0.3653821331379899
0.4945018242648629	0.1153898946774860	0.3645794954994671
0.4981962949760783	0.3163788993492427	0.4000145951225615
0.0842577575082786	0.3104765215984511	0.0947635875003018
0.5858313565270862	0.8042471755035182	0.1673020017673866
0.9026836957073677	0.7100994410916239	0.3546907584849993
0.4032283783751820	0.2145695476155873	0.4088067592902667
0.9039961863190845	0.3059532294200782	0.1109380620523056
0.4044861987401164	0.8099953518531497	0.1689643499747227
0.0892155059444597	0.7155000343281808	0.3576976939916849
0.5839404471373858	0.2102278586451129	0.4270789740182941
0.1723797992929565	0.2198068387556463	0.3884869839822290
0.1549528708583997	0.4442769115313071	0.3820141745496710
0.6757909933480356	0.6729688443579379	0.3891636264443687
0.6726804533291669	0.9141519525447276	0.3904144978804605
0.8222943829044356	0.8041304294180397	0.1433409420553801
0.8413483301119711	0.5724619193176348	0.1366125616323058
0.3240006215972718	0.3417752054441607	0.1366831187617599
0.3181572292555305	0.1072793429653769	0.1339062207645950
0.8226391448032271	0.1794656879459887	0.3832681389124930
0.8161749660302126	0.4133829437618461	0.3865873196562837
0.3175003914114713	0.7190099312077854	0.3752589444870421
0.3375333423607700	0.9430516479650841	0.3799655137702406
0.1672203276632973	0.8407840609817322	0.1259550655110886
0.1728607848875426	0.6075464168226723	0.1278257062639803
0.6702266382408234	0.3007662853403554	0.1319638866808471
0.6520003736905483	0.0803938917771527	0.1383045506101125
0.2054369432151246	0.5066536902134410	0.2352353457542371
0.7053910971212588	0.0141580658705803	0.2809396614980883
0.7833317500071827	0.5145670282524445	0.9913377311997520
0.2848476909162839	0.0063549570260797	0.0264680389944351
0.7723716284695443	0.5041406591976682	0.2651483237739107
0.2719311759966542	0.0163870589548941	0.2541274685723370
0.2178527055764621	0.5169197603837302	0.0074035047658161
0.7171444716522922	0.0046511726115239	0.0147517171369535
0.1305076739013969	0.3947302135087792	0.7190266240376816
0.1124643300293892	0.2261550625194344	0.7278048481355689
0.2452007198792171	0.1290330431765056	0.9804247374216010
0.6056352823554727	0.9336518912064602	0.7321102636533263
0.6233241306163025	0.6794321785613323	0.7289096916859154
0.7313281008659398	0.6301242233528694	0.0473781917869571
0.8655883877347835	0.6242133442824589	0.9763423689825097
0.8801342666775855	0.7961246968887584	0.9815419812309549
0.7437304696962256	0.8908003404181413	0.7368638442271869
0.3857043639623793	0.0950207366084011	0.9758874042434011
0.3665525719370010	0.3333658811397487	0.9682826677869105
0.2661416914413330	0.3937465333072669	0.7867650957333000
0.8842257417177112	0.4268039606243059	0.5444073737348502
0.8657457984114491	0.1876414407782727	0.5514903770439837
0.7654769341313333	0.1301299028258382	0.7316337226317250
0.3667075782022857	0.8958995999376137	0.5416296042377472
0.3744626369145616	0.7300001537024631	0.5354754136900680
0.2445814104540621	0.6290313638305447	0.7848340963544679
0.1042069652024586	0.5939195735286663	0.7863221687456117
0.1189060598469000	0.8357735076496995	0.7920374460140653
0.2309326321258302	0.8876008395127737	0.4757478349633703
0.6310556208228620	0.1252328943585720	0.8004294952289797
0.6122800313262210	0.2960972849285545	0.7916019991326024
0.7433523688399029	0.3927738540485311	0.5406015496174800

0.8860392364475070	0.5808164365557489	0.8071901189830213
0.8564558043985158	0.8286796675438742	0.8086604344961025
0.7745528934426303	0.8856306942292528	0.5139676799074916
0.3590584260432094	0.1168936860881989	0.8005789143284776
0.3831880939377786	0.3157996386015340	0.7921667402584529
0.2357651169360891	0.3839999581216946	0.5063809295607375
0.1067801631957965	0.4372672998422977	0.5502514871932630
0.1324084394713332	0.1901375179162754	0.5559021641829706
0.2171231332859699	0.1372957307808808	0.7596661538893332
0.6333666897930345	0.9047818569691644	0.5596176051856135
0.6155196902595685	0.7106404836833518	0.5414234547449064
0.7580286791751645	0.6347468176770635	0.7650969180066365
0.1358425646479352	0.6212491941820647	0.9588676718691648
0.1127034674388864	0.8114658815921145	0.9682393938170747
0.2510866831324432	0.8862989132127123	0.7598117790313161
0.6055914267122186	0.0835835474007804	0.9694581491465720
0.6323362805652394	0.3306633785420505	0.9638688145231552
0.7165259513059277	0.3850983220431651	0.7582001711919841
0.8596802793210001	0.4024427742836550	0.7198997154773606
0.8830224070401392	0.2077391586767800	0.7276079521339209
0.7355890932018482	0.1351855186625707	0.0118079624772943
0.3798012480697068	0.9389112015736247	0.7112810296726355
0.3524963095101051	0.6925624504738997	0.7051312152005427
0.2762365791760123	0.6355836697912094	0.0058457271731765
0.2518869658592509	0.2643024175516241	0.7519176273090080
0.7484467087956722	0.7570334790257399	0.4913092438325008
0.7571427207388918	0.2657153341260697	0.4918975712169784
0.2288254747519879	0.7567567551955136	0.7428527361016942
0.7314292549629375	0.7627324819391896	0.7826432734395703
0.2583082703744997	0.2542143592793347	0.5267167933400516
0.2316263866754631	0.7575024640951341	0.5122061889895803
0.7525874464938536	0.2588747937418568	0.7703136956396104
0.9948234225223657	0.4058681909058324	0.6565687415148421
0.9984813097734407	0.2050314804188050	0.6205612900628725
0.4978161205700393	0.9111314033299380	0.6160622414350527
0.4861948869189185	0.7009549656671286	0.6396102959981255
0.9989394232596126	0.6151145854737604	0.9072325310405019
0.9911953685774489	0.8159746350065081	0.8712002124512097
0.4948676538686525	0.1155036988668706	0.8614368821754013
0.4988751276463001	0.3162821785501561	0.8985460328128713
0.0846833451760913	0.3110763177409680	0.5939218662847405
0.5835228769341345	0.8109428096952703	0.6722711996860049
0.9060674257041006	0.7086651656754973	0.8493075348197191
0.4037323642684336	0.2148122634683290	0.9062134638294782
0.9041072144325665	0.3065253010237400	0.6105722457627885
0.4063734051442028	0.8129599124583710	0.6704511340360616
0.0850596062007562	0.7150364364154957	0.8501849504963241
0.5847327101066607	0.2096448006745196	0.9243781183881823
0.1714642686741944	0.2205338620075935	0.8890678514955400
0.1540624871798979	0.4421932758289440	0.8821031147666116
0.6725247418686330	0.6759353593478554	0.8959292771033680
0.6727553062609258	0.9157325520752193	0.8911328910722376
0.8145479115148756	0.7991526638563048	0.6421804321429923
0.8385409013924692	0.5738026475313048	0.6395417339603261
0.3224276325052890	0.3415288463238441	0.6363987426624979
0.3153927112358302	0.1051681095001069	0.6341936346677299
0.8229393077259162	0.1803151747635414	0.8835704787597954
0.8151383175489297	0.4115477002886058	0.8866879606054442
0.3195838253285054	0.7197411192025412	0.8758660614626324
0.3373552620674590	0.9438129895777607	0.8810345703764726
0.1689337341529153	0.8415643282759385	0.6264779135201850

```
  0.1745455424601437  0.6066778322074755  0.6288869475206130
  0.6716195210407053  0.2998433078678983  0.6310413147971059
  0.6556446685617354  0.0762782168413482  0.6387302975350414
  0.2075379070182489  0.5069231042851823  0.7378462790713219
  0.7120374215579286  0.0148540965415510  0.7842813569771279
  0.7824144928655400  0.5145893243371917  0.4936418017601130
  0.2832108021564542  0.0044513707647985  0.5252897703371779
  0.7717801400604439  0.5036804047804008  0.7666871819836674
  0.2685859205321761  0.0166810857963977  0.7573988413276297
  0.2213308469083332  0.5145486235106688  0.5112564968089590
  0.7196498297508516  0.0054984225935307  0.5083959375379907
  0.4648025863494392  0.5225990440973889  0.1115103854726339
  0.4030657212799520  0.5127305146537893  0.0566112389309435
  0.5293902701695578  0.5474811794589243  0.0718573966289880
  0.4051812194555879  0.5281374479566945  0.9647259457090233
  0.5318603554304214  0.5629051113608113  0.9802393023228897
  0.4694725600586879  0.5536492290653178  0.9264236034396017
  0.4762353347529139  0.5968474042473969  0.5742264075119112
  0.4608023639136316  0.4804259490447698  0.5629314660971391
  0.4167462965370064  0.4782386692971782  0.6414068934201715
  0.5371251040655546  0.4675954501067191  0.5691638979380450
  0.4493192236361423  0.4678027203351393  0.7250430866690838
  0.5693136640073372  0.4573557605299715  0.6531190643484921
  0.5254571309169250  0.4578835743223977  0.7308461140116804
  0.4624225069188692  0.5071632948304629  0.2110626121658568
  0.4936580115885638  0.6118534443935584  0.4796205002421927
  0.5126248826828668  0.4488209640666411  0.2375126034054048
  0.5103355916495983  0.4392578077143494  0.3104729445336508
  0.4958433261547561  0.4028407883240648  0.2028559094683904
  0.5701360076749782  0.4593357912482660  0.2193630519964256
  0.5444943528740180  0.5879110478892758  0.4567726946784584
  0.4474132872946338  0.6008197942217730  0.4344526938008229
  0.5040614376390421  0.6660942153826289  0.4761843055427750
  0.4052680341760415  0.4960272965564871  0.2306901796745578
  0.4791201915353480  0.5521147984690545  0.2487845201825133
  0.3523389437871037  0.4936828029505373  0.0864903509030346
  0.5779627935288447  0.5557963426073195  0.1137688632033040
  0.3558236317624459  0.5213587235021003  0.9238896326994982
  0.5818684709704436  0.5833082952190599  0.9504834038206209
  0.4346393338478127  0.4840099418856195  0.4970256994203702
  0.3574979108187616  0.4858206680628554  0.6359335324129620
  0.5707316825707137  0.4669023495067823  0.5083114346115433
  0.4159954086930880  0.4672174090719269  0.7860633284561271
  0.6283774649655496  0.4491832589986206  0.6588331477859100
  0.5506966574755233  0.4505789761150548  0.7967861307973394
  0.5184808399814063  0.5871587037476844  0.6243445496815269
  0.4199269626815475  0.6003608559270150  0.5982260727502815
  0.4711482078302328  0.5671690724205871  0.8552811609631692
```

MOR-Al-T4-EBH+EB

```
   Al     Si     O     C     H
   1.00000000000000
    18.1842269897460938   -0.0090872207656503   -0.0001607598969713
    -0.0105508984997869   20.0691013336181641    0.0015994716668501
    -0.0001797722798074    0.0011982563883066   14.8440780639648438
   Al   Si   O    C    H
     1    95   192    16    21
Direct
  0.5900569189532272  0.7172560934764542  0.6333169419022755
```

0.3174764317622628	0.0760565532729079	0.0260727233470666
0.3045182035969064	0.3209512912638150	0.0299626249813937
0.8157836078939070	0.5780492596847356	0.0249884427794631
0.8121098880857213	0.8023153756866481	0.0253212262252060
0.6942194281038577	0.9297283933199280	0.2743338361506710
0.7092814458089998	0.6841122038362289	0.2748263743235370
0.1971278970719488	0.4275158512862519	0.2766299239532538
0.1971758707923551	0.2034538796571679	0.2770845871936587
0.6956164128299891	0.0804281173355711	0.2268534230295668
0.6971233181811579	0.3052863675620061	0.2293039476764379
0.1937817099976047	0.5775485522789054	0.2281311333038080
0.2042020724635815	0.8213237756450623	0.2259980952677977
0.3137210829389430	0.9262243062406748	0.4781731406964040
0.3126276163578694	0.7045412435693590	0.4777568334881738
0.8166908967960977	0.4316050320786472	0.4802344079077500
0.8046946744343014	0.1864553148700080	0.4763186693088010
0.7102704232706590	0.9225098406181154	0.4827080200571167
0.6981206396297589	0.6854686890213209	0.4833500425773785
0.1896730464491755	0.4382579169947798	0.4820660368980890
0.2105794494853227	0.1920062755932314	0.4833267319096576
0.3003289218636355	0.0865887031635723	0.2336497066597181
0.3134068149087069	0.3207762670819285	0.2378990227948854
0.8238245333660256	0.5691535445533054	0.2277590337654449
0.7999030630629594	0.8155263494016284	0.2303169243107607
0.3207703640528001	0.9376501659113280	0.2725078965709063
0.2989221754233943	0.6921495333845962	0.2718259999593445
0.8004552640775980	0.4221324440273491	0.2716787071264983
0.8119156440134855	0.1877343878760758	0.2686071286817425
0.6889067784727955	0.0681387703371994	0.0221437233570436
0.7112010707027879	0.3148520078282467	0.0231488688930559
0.2129004916020988	0.5871105665077908	0.0208209231069837
0.1990863368292148	0.8176089477186462	0.0183491119680841
0.0880468081347347	0.3802338961083033	0.1243980763575293
0.0909848376046898	0.2256187512685177	0.1198852356567881
0.5914564908114229	0.8764444030179888	0.1324402920701544
0.5884502644466562	0.7171231215758910	0.1388871367088734
0.9231881130685611	0.6262120462771305	0.3754540978554110
0.9174858871234129	0.7816297848807215	0.3718164022283353
0.4187835729399121	0.1287969920692973	0.3833242353293154
0.4228260671189104	0.2877111757157905	0.3870771224015715
0.9194693038132550	0.3790526225930116	0.1230232334130175
0.9216502866606616	0.2200949520373939	0.1191401796859062
0.4230904279910857	0.8800230774458112	0.1302739259333363
0.4203102848483120	0.7252782440221570	0.1355499912652555
0.0915884205702352	0.6294520375106274	0.3714627389246606
0.0868566564032265	0.7882175374889540	0.3685031250341173
0.5875732057613576	0.1267389477191178	0.3807758710465267
0.5917651180192089	0.2820393938303077	0.3860078287195550
0.3162292381207236	0.0763749994734539	0.5263888710285177
0.3057730912682354	0.3216392027898914	0.5293385354613783
0.8153110144568416	0.5804288586343012	0.5301292604688936
0.8097749790796098	0.8033414962756391	0.5259553487292822
0.6934646734276739	0.9300598196059355	0.7754392325496783
0.7078040241579669	0.6828185198334257	0.7813725918144394
0.1977907638360340	0.4279065589797994	0.7764590175153554
0.1969990922927583	0.2035977713173147	0.7771981836864754
0.6988036709970915	0.0784772186176705	0.7277886248084761
0.6961230640924936	0.3042987149018124	0.7293977161077833
0.1936082983325419	0.5768924570242434	0.7287881256827421
0.2036692466608638	0.8215800553229081	0.7277883991324644
0.3136723603361720	0.9262024933617526	0.9782362931062539

0.3168931324233477	0.7022596822733512	0.9786954891954609
0.8179854771395418	0.4289515421485779	0.9781644169801256
0.8053295101663457	0.1848853684572462	0.9762537050189155
0.7095758770089308	0.9188073531487936	0.9828227598802551
0.6971342507358920	0.6840377592027441	0.9891106321585803
0.1899445989075384	0.4384132533384020	0.9822005166823632
0.2110000193924094	0.1912887413609593	0.9838260585148074
0.2993535805294799	0.0863261675809019	0.7346870609759230
0.3135339402143066	0.3198767641575558	0.7371792111603427
0.8257999085621274	0.5680364158708260	0.7355721841366603
0.7981452027715586	0.8126045880130240	0.7337406774961168
0.3203675415124396	0.9377823984632007	0.7732873791183236
0.2979434063636761	0.6918274810518211	0.7733778429667291
0.8010477492490242	0.4191963684225551	0.7717079170849315
0.8125140599336276	0.1881373416490989	0.7688712596206017
0.6900859943175532	0.0690886465586608	0.5221493060603225
0.7117068873075970	0.3157813359079767	0.5233891895495768
0.2107040530259097	0.5871621664233999	0.5204851084326667
0.1959067585065517	0.8191084984792798	0.5184853902967559
0.0877497868776022	0.3811124588726195	0.6258634241542738
0.0907833560211382	0.2262370342477602	0.6214488799688394
0.5920592201354996	0.8799660749629673	0.6311296620801009
0.9260627367649649	0.6245526853353269	0.8745562869832136
0.9193412494817162	0.7787649913511994	0.8670674541835880
0.4195431742371372	0.1277972544920023	0.8823378675043322
0.4221639952956409	0.2864038614005094	0.8880374221254984
0.9196652210583671	0.3790605584115299	0.6217249325783970
0.9220242433617207	0.2208645277191182	0.6173017586564894
0.4231403443093504	0.8804528813574660	0.6307484257230758
0.4186272850586802	0.7256254911472332	0.6355132525010040
0.0929021574481364	0.6280358867800772	0.8713505205867498
0.0880570628443452	0.7859323060466220	0.8678148824231687
0.5885113072046628	0.1258344020240031	0.8779682127166381
0.5912607551581067	0.2812459869687015	0.8852655616801792
0.1382052791413335	0.3878934116126508	0.2147510006508116
0.1215891321937237	0.2183308718259752	0.2226585874668576
0.2532344976108047	0.1211319372302923	0.4765798461605866
0.6162219171298132	0.9170610121879108	0.2225214114590764
0.6322578134275086	0.6786162885875666	0.2188572709958925
0.7426918729694193	0.6263464037289304	0.5364054450089417
0.8771618705481556	0.6159478150167123	0.4674624865211517
0.8881445365615116	0.7913003133515095	0.4744935034557154
0.7575980435036069	0.8867113385632147	0.2228070986590349
0.3938090954274917	0.0885592895504601	0.4736370532124801
0.3776236022364447	0.3278092815338878	0.4647452869348279
0.2738565193905556	0.3856319174071695	0.2834331828724629
0.8954256511458090	0.4191126051978407	0.0322299047041600
0.8769020693930952	0.1804387807859872	0.0412883180011518
0.7706696642371020	0.1246138524585686	0.2219163191746824
0.3739572081825725	0.8874497880260883	0.0396114626244111
0.3935161978987170	0.7169482987707655	0.0316498514842525
0.2577610256324997	0.6202487640136560	0.2794973170999800
0.1165090742521916	0.5888163747540129	0.2812513662612409
0.1326479398977777	0.8272504604202138	0.2906132249333122
0.2384490336780991	0.8824589241009528	0.9732196560271449
0.6357368178323788	0.1185738061791861	0.2894936087400986
0.6205627444084607	0.2916837782728450	0.2829708045373777
0.7547499004970034	0.3855026167777170	0.0297377358439940
0.8941738181132056	0.5780756009152253	0.2946573959215188
0.8669562701910759	0.8233761665182970	0.3016023045012626
0.7849758147643653	0.8774607162999815	0.0008911383446941

0.3667968582345592	0.1108150421083437	0.2984313823664207
0.3914949261663989	0.3092330660888200	0.2884604449707418
0.2466943654797325	0.3779213804245106	0.0001838304703990
0.1172390300914242	0.4297300545340397	0.0452282688377381
0.1412134505649447	0.1822722192647698	0.0509510379659828
0.2257237109334309	0.1286551703336105	0.2540822433483944
0.6437068161069315	0.8932331790360515	0.0477152903898221
0.6189671252469222	0.6935041480693261	0.0413334199314594
0.7683174336833499	0.6315052459419211	0.2354256608600233
0.1448483262005184	0.6113246300180193	0.4556973191494677
0.1180386723182153	0.8084874776417357	0.4674737829933274
0.2635462246506438	0.8778500202537836	0.2528101067088315
0.6164699059531171	0.0778091882748709	0.4602824442011867
0.6422695969158077	0.3245794488990933	0.4553873765670792
0.7255542050966406	0.3805880197073629	0.2502571442355792
0.8677219544576364	0.3979995788300109	0.2078733462367489
0.8895870783338770	0.2005763533242051	0.2178607049378234
0.7443932781416868	0.1317452286027640	0.5056426474702588
0.3933227063876975	0.9295154966684576	0.2090133339594712
0.3675123579423218	0.6826575398910151	0.2029670442830307
0.2877326899093213	0.6267494188092475	0.4980554448420588
0.2611806569475512	0.2558289695206787	0.2476712803815313
0.7454356916209023	0.7514415811875108	0.0020117793537297
0.7664819762557771	0.2574229771672412	0.9865877216678384
0.2419467155182100	0.7480418736398959	0.2357504883098107
0.7420694393187751	0.7590870364325087	0.2650914801341407
0.2663172639661315	0.2479402185546951	0.0238709597380570
0.2539029987691727	0.7538703627238826	0.0129855695819971
0.7602310703541826	0.2535793455276689	0.2620457206631476
1.0036939599135370	0.3989478132343262	0.1486301210376520
0.0074247868784067	0.1982218864152241	0.1152945268817409
0.5078309903438244	0.8982159563132307	0.1073300435829851
0.5024702253721339	0.6947256869227211	0.1462573377555981
0.0084946068715291	0.6077613543555989	0.3968092682502505
1.0012017733578142	0.8093027253496755	0.3640803782178968
0.5025254470367305	0.1086528239004384	0.3581170131985785
0.5081756327786560	0.3103583117887738	0.3926940614164120
0.0946532205684687	0.3036719905002783	0.0891490934693520
0.5956484434024469	0.7969288716772809	0.1528811980121698
0.9153919569530516	0.7032139256988122	0.3426920612827659
0.4127924851303905	0.2084339857332657	0.4034136685570033
0.9131971093979928	0.2997537750900058	0.1025258162497084
0.4172168632417298	0.8031722068955899	0.1658740996338875
0.0973125354909037	0.7086532898515955	0.3515766388924694
0.5940817530639327	0.2038242331973240	0.4148051506457334
0.1799063613211540	0.2119367992982041	0.3839881090880253
0.1632679821296668	0.4363319490563848	0.3774174525103698
0.6909794880496718	0.6640761218558207	0.3773926729207501
0.6871805609000121	0.9072369236888308	0.3785234281778817
0.8327468964489593	0.7963071160936326	0.1318276502666407
0.8522529210045789	0.5652564085632432	0.1238983705343627
0.3307493697109782	0.3350419389205042	0.1327236984809184
0.3267872851961939	0.0991586477553062	0.1302578169934975
0.8302069612720793	0.1728691429715949	0.3733960030861447
0.8254202784302759	0.4093517710890758	0.3755916701629111
0.3302217798574871	0.7121090016205031	0.3708322491729535
0.3468549395290512	0.9355429266333120	0.3771565541326387
0.1784199424577484	0.8321248946302316	0.1227415337992935
0.1856269120049277	0.6004249124152531	0.1240170914561834
0.6802116832599905	0.2955994947983839	0.1226203411040704
0.6613088312765324	0.0726437144250550	0.1262938870321334

0.2170344010274269	0.4993518422686342	0.2325210235568009
0.7157585335218373	0.0082419111330857	0.2691845341989793
0.7949724402520344	0.5069912293579911	0.9786768277566513
0.2938559548692836	0.9980765117798782	0.0228496345292560
0.7814447962322787	0.5002441789666390	0.2541316842531400
0.2811467271894961	0.0085515069559256	0.2509579223727710
0.2303294824543005	0.5083396539333066	0.0063660332020727
0.7278843015791490	0.9967550516845877	0.0035956736311215
0.1369657280380111	0.3887218689949206	0.7165360178941251
0.1203423856022109	0.2182069428785835	0.7245915854202544
0.2541016599313430	0.1206031729903917	0.9766159496296656
0.6167580994411301	0.9232507245777278	0.7204494916968047
0.6415507027333534	0.6744837033700876	0.7130229751836628
0.7406307157219090	0.6212217148425335	0.0342548134787192
0.8750571540794315	0.6180322443209225	0.9638641460583153
0.8856352362444917	0.7848304800734786	0.9684072117042971
0.7568709745098098	0.8844229537540754	0.7285773125492416
0.3948107130620976	0.0866623132349497	0.9722402806036476
0.3764469141816483	0.3253924581815731	0.9658706227829906
0.2732168220425093	0.3843650584846285	0.7818269032932854
0.8946823249673644	0.4208990248697599	0.5327289543321510
0.8770087072783003	0.1808509441358933	0.5399740674455168
0.7730852961229149	0.1238763074272395	0.7227618111578835
0.3740680326446649	0.8882669605482321	0.5393687500392521
0.3878218115400087	0.7198250344814540	0.5310112795767058
0.2551979143180421	0.6206797306762103	0.7815709482163832
0.1154208247433514	0.5858473839097746	0.7810506378981260
0.1308013028964882	0.8279575156592186	0.7902015933560479
0.2388099337064075	0.8817993496365347	0.4729990593780349
0.6370324830446413	0.1173298966763018	0.7871808927623264
0.6187456434040677	0.2916634476638149	0.7816656938675043
0.7538969388777281	0.3869361681224500	0.5306354020892111
0.9006788092567991	0.5724454947380613	0.7964579437420718
0.8719563920655222	0.8217013104596371	0.7951665352842594
0.7831100655373879	0.8797110956796876	0.5086185830028369
0.3668111738186550	0.1106501514751380	0.7978102963698515
0.3905358317316006	0.3068705512057397	0.7891503589577673
0.2487337235534165	0.3798477549222198	0.5009100017019957
0.1181934159783043	0.4303794941968672	0.5467076000999863
0.1424364146378388	0.1839732604365323	0.5528925043438427
0.2248445681761315	0.1284175598689604	0.7555279236743837
0.6403476764387099	0.9055073405864088	0.5450882622376515
0.6179789641099201	0.6946058351645216	0.5244373260063120
0.7732605756751715	0.6290541262787723	0.7622774980731467
0.1481114743390230	0.6116819533805199	0.9541063247535134
0.1228388991567380	0.8024582574875454	0.9661986431387228
0.2624505572930985	0.8783094038601649	0.7552134785407172
0.6171669128259365	0.0764242846380119	0.9573262654523852
0.6424210496668505	0.3235049699970912	0.9543632824343197
0.7251002436936014	0.3793989811441892	0.7500137965063551
0.8672077546903412	0.3938171871858471	0.7070710371478831
0.8887738478501340	0.2037609035624973	0.7162358001547573
0.7474051861017499	0.1269682819645026	0.0020389095811946
0.3918188405556912	0.9303890871492951	0.7086041969825110
0.3618743732627798	0.6834819040374716	0.6990446044837705
0.2889585939102599	0.6271807759350551	0.0023517784241632
0.2603633458390544	0.2551931925070313	0.7435804630201455
0.7481281105139248	0.7532599449563688	0.4858004995563021
0.7684152806899872	0.2603314707148020	0.4844110481333525
0.2398342980298122	0.7483206110592215	0.7405019335756933
0.7457337939486925	0.7570734576249735	0.7800281979902114

0.2671139048679382	0.2492041015710877	0.5195876787454743
0.2449004921031191	0.7515030021826273	0.5081433918266322
0.7579623198131368	0.2520920172953262	0.7641533554956770
1.0032908159710814	0.4004890883714778	0.6480049058779898
0.0075976669438397	0.1979379498003743	0.6150520427937567
0.5069289031520245	0.9007484372876489	0.6085734804170423
0.4979682012604648	0.6933008578035386	0.6411072966253717
0.0105284840856723	0.6071378690973366	0.9020984346994724
1.0026150620351815	0.8078714748605471	0.8682622832079804
0.5031983365069681	0.1081162229792636	0.8559031589943044
0.5075830217395491	0.3085301895155478	0.8939055788147198
0.0942306992235825	0.3044175437924781	0.5909684721956164
0.6017007079477609	0.8024482111594438	0.6488935390442490
0.9204534386027370	0.7005441799641833	0.8367011981093080
0.4129347211046003	0.2069323700627828	0.9045186290530935
0.9159310079366315	0.3002556811704868	0.5972581335266156
0.4145278114232904	0.8039648098475456	0.6659657204265547
0.0972992639043696	0.7067624890249923	0.8468257387988358
0.5954133176800532	0.2026980500551379	0.9127730498145036
0.1822712053492192	0.2132501282584251	0.8844208028824290
0.1647346342773851	0.4383127014935910	0.8771204775142420
0.6792909546553640	0.6702720822652007	0.8846856186425207
0.6817570163714426	0.9083392520756953	0.8799698669268420
0.8228234464412021	0.7902381469598863	0.6327304363174229
0.8499877908263379	0.5697545522488490	0.6300011593948945
0.3326005727453324	0.3342983787454029	0.6320519043192908
0.3246518558240504	0.0986910313826805	0.6308059113004493
0.8328563879165741	0.1741250965076838	0.8734179881729996
0.8268870062105633	0.4039629198505306	0.8748313798137721
0.3314318423780556	0.7113363998902411	0.8715263337144697
0.3474449913541748	0.9357917364358528	0.8778262787097377
0.1792164035333927	0.8324264736556271	0.6237657764129421
0.1865199629008867	0.6003872236222965	0.6241030687428646
0.6812576374658352	0.2942941786541974	0.6221748224995491
0.6654913083221778	0.0677997440371863	0.6272811226194840
0.2178949864014996	0.4988505720514926	0.7296749450809007
0.7203328541092211	0.0077907782521663	0.7737062111217160
0.7924762484464866	0.5091318981091651	0.4854228467083277
0.2929229147064905	0.9984235830555190	0.5211393770043588
0.7843680625727278	0.4975983236204475	0.7561463221249969
0.2798942234834331	0.0085863176587504	0.7530822756648565
0.2306484896764277	0.5090882126960877	0.5025830444470226
0.7324500207808904	0.0009670108675749	0.4943836625833444
0.4353384619132579	0.5196254766787530	0.9738256427230481
0.4007714179117712	0.4920776213044418	0.8981083740217268
0.4855865334553745	0.5718630598451496	0.9610625345318643
0.4159803013492817	0.5161665586356921	0.8117250550170563
0.5012874019337017	0.5959213798275438	0.8748829626507632
0.4664360446840572	0.5680214936142537	0.7998137319021946
0.4208599061046131	0.5041927629435676	0.5686760537431933
0.4225757280204868	0.5386136683336682	0.4758936147516284
0.3994690195473325	0.5002961193725359	0.3973548138275597
0.4782778413626488	0.5898904614173397	0.4612397472770763
0.4232553053856081	0.5171558985824810	0.3122070262964276
0.5025536597236768	0.6050748623306528	0.3754270669001686
0.4747213721247870	0.5691528734651344	0.3015746563003266
0.4211598922045932	0.4923210423934409	0.0669320739217749
0.4858920543473347	0.4567880502961317	0.5811836560633687
0.4822086735240369	0.4443294862302116	0.0971461380640398
0.4711184051496693	0.4231275118234480	0.1642143579109717
0.4869513940285147	0.4027002085059065	0.0494067447180666

```
  0.5356795183383536  0.4700217033655802  0.0991907252511072
  0.4831967867789619  0.4333954491952359  0.6480300913243943
  0.4853973549793130  0.4171440536182164  0.5300533406874192
  0.5383773548135402  0.4838582084254205  0.5763408873916464
  0.3676760570893768  0.4666141431351156  0.0680393024257600
  0.4169597589789149  0.5343487430684254  0.1146425194351494
  0.3615898685913990  0.4513933744566889  0.9069766166804321
  0.5126601991793904  0.5942917817648563  0.0193504867081303
  0.3876349307215354  0.4943899624011350  0.7539577202164903
  0.5408684830904474  0.6362349285503319  0.8659056955153256
  0.3713538960313718  0.5729277926856684  0.4790870515332543
  0.3600351749639226  0.4599330810797289  0.4075069727663934
  0.4981702980490137  0.6183012119255897  0.5192191800400235
  0.4031294682149614  0.4903960077843715  0.2532900763625318
  0.5435354385156190  0.6440664043213941  0.3654186905135651
  0.4933252832186289  0.5823515605818153  0.2339372987445786
  0.4213859297800840  0.5431627236076171  0.6206993048235616
  0.3683364289015647  0.4773387234367462  0.5748281064878495
  0.4786860194415760  0.5879525317452764  0.7331468905744769
```

MOR-Al-T4-TS4-Transalkylation

```
   Al     Si     O     C     H
   1.00000000000000
    18.1842269897460938   -0.0090872207656503   -0.0001607598969713
    -0.0105508984997869   20.0691013336181641    0.0015994716668501
    -0.0001797722798074    0.0011982563883066   14.8440780639648438
   Al   Si   O    C    H
     1    95   192    16    21
Selective dynamics
Direct
  0.5916759371757507  0.7188676595687897  0.6393225193023682
  0.3165232539176941  0.0763325765728954  0.0311785731464625
  0.3043941259384156  0.3213416635990158  0.0344364270567894
  0.8156739473342899  0.5796346664428758  0.0306418277323247
  0.8137264847755432  0.8045156598091162  0.0295175220817329
  0.6942579746246363  0.9305654168129014  0.2794213891029371
  0.7088899612426761  0.6842985749244732  0.2797146141529083
  0.1963990926742554  0.4270557463169130  0.2822557389736179
  0.1964389383792877  0.2025928348302842  0.2813420295715332
  0.6961035132408142  0.0801708698272709  0.2318569123744972
  0.6961495876312258  0.3053177297115326  0.2333890199661262
  0.1942043751478204  0.5767747163772631  0.2340790629386910
  0.2047590315341959  0.8215734362602295  0.2319003492593772
  0.3134682178497314  0.9256558418273981  0.4837790429592136
  0.3143078386783594  0.7028219103813218  0.4843214750289935
  0.8159846663475037  0.4316454529762305  0.4844759106636060
  0.8043233156204247  0.1864416897296921  0.4802949726581586
  0.7098278403282166  0.9221226572990492  0.4872539341449753
  0.6980715990066528  0.6859697699546854  0.4876125454902669
  0.1886177361011505  0.4383566081523920  0.4877082407474533
  0.2105897963047028  0.1916630864143383  0.4877064824104323
  0.2997975945472718  0.0864722430706030  0.2390821874141703
  0.3122846186161043  0.3196625113487244  0.2422223538160334
  0.8235717415809656  0.5691069960594201  0.2325707376003273
  0.8002236485481296  0.8157396912574829  0.2354208976030361
  0.3216567933559430  0.9377319812774721  0.2783809602260590
  0.2991894483566284  0.6922761201858565  0.2778874933719639
  0.7998639345169070  0.4216617941856421  0.2758934795856480
  0.8115583658218387  0.1882356405258190  0.2726476490497593
```

0.6887031197547936	0.0687687173485760	0.0270007438957692
0.7105109095573429	0.3154806494712853	0.0270433388650418
0.2120382487773896	0.5867742896080040	0.0264655090868474
0.1977433115243914	0.8183168768882809	0.0241707023233176
0.0872844830155373	0.3808463215827966	0.1295057684183121
0.0906038805842400	0.2258775532245652	0.1248948872089390
0.5920390486717224	0.8787831664085449	0.1375268846750270
0.5893222093582153	0.7208060026168870	0.1436158120632172
0.9229987859725954	0.6270449161529589	0.3792797625064854
0.9182267189025932	0.7824614048004197	0.3759267032146479
0.4186199903488171	0.1289546638727198	0.3879696726799031
0.4216154813766479	0.2875219285488145	0.3921852707862871
0.9184736013412482	0.3792083859443688	0.1267074644565582
0.9212623238563579	0.2205385714769372	0.1227688193321233
0.4229122102260590	0.8814871907234253	0.1355441659688952
0.4194378256797791	0.7273501157760646	0.1406367272138598
0.0913700684905053	0.6294329762458865	0.3760741055011753
0.0876058563590050	0.7877213954925584	0.3730731606483463
0.5872561335563663	0.1267335265874870	0.3848268091678633
0.5905053019523647	0.2820374667644516	0.3897505104541792
0.3164402246475220	0.0760799869894983	0.5307593345642113
0.3046971857547776	0.3216494321823132	0.5342107415199284
0.8149049878120442	0.5808825492858927	0.5337200760841402
0.8104394078254703	0.8035547733306948	0.5298255085945134
0.6937733888626122	0.9308220744133070	0.7800415754318245
0.7083447575569153	0.6832804083824222	0.7863525748252894
0.1967942118644721	0.4274684488773383	0.7814967632293736
0.1964855790138244	0.2025634646415720	0.7814023494720467
0.6979286670684818	0.0788490250706677	0.7320082187652596
0.6960566639900206	0.3044640719890606	0.7334773540496861
0.1930864006280899	0.5766969919204735	0.7337241172790535
0.2040953189134598	0.8213118910789527	0.7319589853286751
0.3129458725452433	0.9269037246704165	0.9835504293441807
0.3150286376476299	0.7028833031654421	0.9847138524055522
0.8161409497261086	0.4307908117771186	0.9829798340797450
0.8047131896019009	0.1855744719505319	0.9808140993118319
0.7102921009063721	0.9202406406402635	0.9874426722526600
0.6986682415008545	0.6872812509536806	0.9936597347259568
0.1895317733287812	0.4383870065212269	0.9870169758796700
0.2106768339872360	0.1916745007038122	0.9880243539810211
0.2994971275329591	0.0857733190059668	0.7385901212692291
0.3125706911087036	0.3194037973880792	0.7417755722999601
0.8260034918785099	0.5685051083564805	0.7388290762901334
0.7980346679687531	0.8133078813552900	0.7374100685119657
0.3209289908409119	0.9370594024658266	0.7782000303268433
0.2979278266429901	0.6917687058448817	0.7784867882728584
0.8003751039505005	0.4198954403400442	0.7755082249641452
0.8120543360710147	0.1885167658329022	0.7732178568840052
0.6896395087242129	0.0691116824746136	0.5262913107872037
0.7107673287391665	0.3156797587871552	0.5270017981529240
0.2099176347255707	0.5876514911651641	0.5258448123931908
0.1972599178552628	0.8185396790504494	0.5237228870391850
0.0869977846741679	0.3806228637695344	0.6305678486824043
0.0904470235109329	0.2255797535181056	0.6254211068153408
0.5914016962051418	0.8810162544250563	0.6368238925933846
0.9256570935249326	0.6250739693641709	0.8787819147110011
0.9193773865699773	0.7796477675437974	0.8715441823005704
0.4185520112514497	0.1278956085443508	0.8875486254692108
0.4215256869792955	0.2865100502967846	0.8927848935127305
0.9189107418060309	0.3786467015743279	0.6261041164398223
0.9214841723442078	0.2205562293529521	0.6216410994529752

0.4240178763866436	0.8789786100387610	0.6353936195373561
0.4210113286972046	0.7225360870361359	0.6410201191902161
0.0927714183926585	0.6284440159797700	0.8771665096282991
0.0882394462823868	0.7865427732467715	0.8730822205543548
0.5876069068908691	0.1256646513938912	0.8830205202102669
0.5907200574874905	0.2810725867748284	0.8891472816467313
0.1359825581312180	0.3888583481311820	0.2206729799509050
0.1202897652983671	0.2174778133630771	0.2278734594583518
0.2532830536365510	0.1207365542650232	0.4811387956142449
0.6163049936294556	0.9210896492004488	0.2264837175607694
0.6329640746116661	0.6804956793785158	0.2221106588840492
0.7411604523658772	0.6255955100059556	0.5411561131477384
0.8756351470947242	0.6169560551643419	0.4705770611763014
0.8888646960258484	0.7918384671211274	0.4787116944789903
0.7568390965461731	0.8864094614982675	0.2282033711671841
0.3938845396041871	0.0875876098871235	0.4775117039680485
0.3762978911399854	0.3277693688869500	0.4693434536457080
0.2714591920375837	0.3831853270530732	0.2887049317359938
0.8934193849563599	0.4204114079475433	0.0370439216494562
0.8765599727630644	0.1804904043674476	0.0451935715973378
0.7707189321517944	0.1249190047383316	0.2260673791170134
0.3740664124488844	0.8895012736320498	0.0447620302438736
0.3914666175842285	0.7185320258140633	0.0369825996458532
0.2579819858074188	0.6202781200408937	0.2842725813388828
0.1167317703366284	0.5876342654228242	0.2871167063713078
0.1332705169916153	0.8282867670059242	0.2965997457504272
0.2382961660623561	0.8823579549789436	0.9786364436149605
0.6358363032341003	0.1183470413088808	0.2939082682132721
0.6194840669631958	0.2917642295360577	0.2867659032344818
0.7528930306434657	0.3867461085319542	0.0333494804799558
0.8939998149871833	0.5783592462539719	0.2991056740283970
0.8680639863014221	0.8245368003845261	0.3056153357028965
0.7861416339874268	0.8797962069511507	0.0068081603385508
0.3661729097366333	0.1120896711945542	0.3030800223350525
0.3899961411952974	0.3070785403251672	0.2931350767612471
0.2457489371299744	0.3776307106018101	0.0048094317317009
0.1170062795281415	0.4303392767906219	0.0505561307072642
0.1412274688482285	0.1830828338861477	0.0558163113892082
0.2243892103433609	0.1274774372577673	0.2591187357902527
0.6438891291618347	0.8955797553062500	0.0521344952285292
0.6215866208076477	0.7009680867195153	0.0453396365046504
0.7670849561691286	0.6305150389671389	0.2409476637840281
0.1437194794416428	0.6123387217521691	0.4613822102546706
0.1199344769120216	0.8052093982696572	0.4724613726139081
0.2648721933364868	0.8777605295181335	0.2576365470886242
0.6160269975662233	0.0778184160590176	0.4645239412784580
0.6413788199424746	0.3246341645717650	0.4590825736522678
0.7245380878448486	0.3806161582469965	0.2551840245723736
0.8667623400688171	0.3968304991722131	0.2121909558773043
0.8889565467834473	0.2017137408256531	0.2216136455535898
0.7441680431365969	0.1314568370580679	0.5090944766998291
0.3941973149776472	0.9314475655555778	0.2144568264484417
0.3673836588859572	0.6843618154525803	0.2082293480634702
0.2863209545612335	0.6269701719284103	0.5051479935646080
0.2603477835655222	0.2541731894016289	0.2496116161346437
0.7484229207038879	0.7540699243545579	0.0005697023589164
0.7667431831359864	0.2586276829242706	0.9907820224761988
0.2414060533046733	0.7480230927467394	0.2424037754535684
0.7441927194595340	0.7584767937660281	0.2716996967792530
0.2665323317050934	0.2481906861066830	0.0268033165484668
0.2508365511894226	0.7534395456314146	0.0178908389061690

0.7592617869377136	0.2536968886852280	0.2662674486637127
0.0024967382196337	0.3994956612587007	0.1519705057144167
0.0069495574571192	0.1985675096511852	0.1192653179168702
0.5079053044319182	0.8984277248382629	0.1119720041751863
0.5030453205108643	0.7013776302337693	0.1491183936595923
0.0079140746966004	0.6082926988601716	0.4014751911163344
0.0021074025426060	0.8097060918807991	0.3695051968097706
0.5022648572921782	0.1087657436728485	0.3619488179683709
0.5071221590042114	0.3102661371231102	0.3969860970973993
0.0941955819725993	0.3041048049926769	0.0949325263500218
0.5990834832191471	0.8001831173896864	0.1613804101943970
0.9153975248336794	0.7040252685546922	0.3464461266994489
0.4129381775856018	0.2081835120916378	0.4103571772575393
0.9126580953598022	0.3000960648059863	0.1048637554049496
0.4141103327274324	0.8052008152008112	0.1712888330221186
0.0965766608715057	0.7083413004875230	0.3534818291664136
0.5938543677330017	0.2038633823394787	0.4187186062335983
0.1802264302968979	0.2118305861949936	0.3883521258831039
0.1625766605138779	0.4363347291946429	0.3829626142978688
0.6903262138366699	0.6639404296875047	0.3823062479496006
0.6859759092330933	0.9073098301887514	0.3832820653915426
0.8321866393089297	0.7966502308845568	0.1363445967435839
0.8524789214134241	0.5656528472900414	0.1288722008466726
0.3302158415317535	0.3348962068557751	0.1373621374368668
0.3258514702320087	0.0989406481385237	0.1354852467775347
0.8302017450332642	0.1735314428806317	0.3774051666259786
0.8250406980514530	0.4095461070537599	0.3799439072608962
0.3301059603691101	0.7119684815406846	0.3771409094333664
0.3475943505764008	0.9348619580268921	0.3831809461116805
0.1782237738370896	0.8326657414436394	0.1288231164217001
0.1852206438779831	0.5992957353591961	0.1297483891248709
0.6797003746032715	0.2960547804832471	0.1265167146921158
0.6616913676261929	0.0715151131153112	0.1314779520034796
0.2174822688102722	0.4986443519592317	0.2381213456392297
0.7176281809806825	0.0087518580257893	0.2756330370903029
0.7921335697174096	0.5088552236557038	0.9846833944320712
0.2920705676078795	0.9985916018486086	0.0276513639837504
0.7814299464225769	0.4998800754547150	0.2582433819770815
0.2814180850982680	0.0084961308166385	0.2578156292438520
0.2306459397077560	0.5083628296852158	0.0100306160748005
0.7288900017738342	0.9983119964599684	0.0065624187700451
0.1358350515365604	0.3885785341262841	0.7215008735656767
0.1198782622814184	0.2169481515884415	0.7285168766975411
0.2535263895988464	0.1208720952272424	0.9812517762184182
0.6166583895683289	0.9245574474334766	0.7254967689514188
0.6416578292846711	0.6737452745437659	0.7182861566543620
0.7427707910537722	0.6256531476974518	0.0415385477244856
0.8751639127731323	0.6178155541420023	0.9681668877601661
0.8881617188453677	0.7876568436622666	0.9738181233406100
0.7563844323158264	0.8848726749420240	0.7319115400314378
0.3939987123012544	0.0867334753274923	0.9774910807609586
0.3765309453010569	0.3261335492134110	0.9705198407173191
0.2721588909626007	0.3838828206062343	0.7864828705787693
0.8936331868171696	0.4209232926368732	0.5375121235847478
0.8762971758842468	0.1805947124958054	0.5444226264953621
0.7715600132942200	0.1250773072242743	0.7266011238098152
0.3725148439407349	0.8864833116531428	0.5460720062255867
0.3906562328338624	0.7160421609878588	0.5373630523681645
0.2560998499393463	0.6200132966041596	0.7855781912803678
0.1155978366732598	0.5863076448440575	0.7871742844581632
0.1320409327745438	0.8275287151336744	0.7955782413482696

0.2378747314214707	0.8823195099830702	0.4776082634925862
0.6358377933502174	0.1167188957333570	0.7919688224792506
0.6187841296195986	0.2914804220199601	0.7856741547584541
0.7525205016136172	0.3872509002685570	0.5341696739196801
0.9002428054809564	0.5729113817214966	0.8004851341247589
0.8710374236106876	0.8221782445907595	0.8000927567482002
0.7830813527107239	0.8795705437660278	0.5120269060134888
0.3662007451057436	0.1103893369436264	0.8027907013893135
0.3896496891975403	0.3068086802959465	0.7939600944519069
0.2464736998081206	0.3792209327220942	0.5078523755073547
0.1164494678378106	0.4302354454994239	0.5515732169151306
0.1418502032756811	0.1834310293197640	0.5565484166145332
0.2246334254741679	0.1274272352457058	0.7595677375793457
0.6408395171165466	0.9038609862327650	0.5505989789962776
0.6191456317901611	0.6968254446983368	0.5310234427452115
0.7729369401931763	0.6292228698730492	0.7650010585784920
0.1467185020446777	0.6113480329513555	0.9606533050537137
0.1209918409585953	0.8045211434364382	0.9720683097839412
0.2631425559520721	0.8774871826171949	0.7601932883262675
0.6165384054183960	0.0767353028059009	0.9627819657325770
0.6419528722763062	0.3236341476440454	0.9578241705894520
0.7243268489837646	0.3800261616706866	0.7540290355682381
0.8663525581359867	0.3937571942806256	0.7114894390106227
0.8885651826858525	0.2034335434436817	0.7208061218261747
0.7459408044815063	0.1286812871694573	0.0075714038684964
0.3930109739303592	0.9272499680519178	0.7152110934257537
0.3650100231170670	0.6824498772621188	0.7079594731330913
0.2877903282642366	0.6273136734962510	0.0071603893302381
0.2596312463283539	0.2545625269413018	0.7483574748039271
0.7496331334114101	0.7525784969329882	0.4899956881999987
0.7677034735679626	0.2602419555187249	0.4887177646160143
0.2407983690500259	0.7478652596473729	0.7427535057067901
0.7449841499328613	0.7576599717140267	0.7826812863349915
0.2666193246841431	0.2488181293010724	0.5246733427047753
0.2497971802949916	0.7532224059104966	0.5168275237083435
0.7586506009101868	0.2533118426799774	0.7683939933776885
0.0023347444366664	0.3996565937995911	0.6529781222343453
0.0069893915206194	0.1977435946464556	0.6190964579582252
0.5068459510803256	0.9022374153137268	0.6122176647186287
0.5005285339254750	0.6848490167365843	0.6490828379770699
0.0099627319723368	0.6078262925148034	0.9062274098396331
0.0027804626151919	0.8081332445144691	0.8700513839721710
0.5023013353347815	0.1080521717667584	0.8613362908363376
0.5071622729301455	0.3084499239921593	0.8969562649726893
0.0936603844165802	0.3039183616638195	0.5959030389785774
0.5987620353698730	0.8034589886665391	0.6573441028594996
0.9190160632133482	0.7012093067169208	0.8418522477149989
0.4122290015220654	0.2070506513118762	0.9095799326896697
0.9144510030746456	0.2999457120895416	0.6015340089797981
0.4207907021045685	0.8014075756073035	0.6688116788864169
0.0981157571077350	0.7072235941886933	0.8536882996559171
0.5949758291244546	0.2026918232440958	0.9170326590538055
0.1811981052160270	0.2124161124229446	0.8884732127189666
0.1637012362480164	0.4380911290645627	0.8821002244949369
0.6811593770980837	0.6696300506591828	0.8897306323051490
0.6830599904060345	0.9086787104606703	0.8844616413116502
0.8233045339584397	0.7907898426055977	0.6367710232734706
0.8502526879310633	0.5704070925712632	0.6333274245262154
0.3321844637393951	0.3336392343044313	0.6369265317916878
0.3252245485782623	0.0988199785351759	0.6350555419921909
0.8315674066543578	0.1740790754556671	0.8778253197670014

```
0.8255484700202942  0.4067006409168262  0.8792231678962733
0.3297737538814545  0.7114954590797471  0.8772884607315108
0.3466663360595713  0.9364642500877383  0.8830956816673329
0.1787193119525910  0.8328948020935096  0.6285696029663094
0.1845709830522541  0.6003859043121385  0.6296503543853785
0.6807710528373743  0.2947593033313775  0.6263021230697640
0.6648185253143311  0.0681964755058292  0.6314385533332832
0.2173257470130921  0.4986579418182404  0.7353485226631172
0.7208499908447267  0.0084208501502872  0.7777759432792694
0.7913388013839722  0.5092462301254304  0.4901695549488083
0.2933106720447540  0.9980148077011116  0.5266975164413479
0.7832596302032472  0.4982770979404476  0.7580885291099556
0.2809896171092987  0.0076787532307208  0.7556097507476814
0.2287912219762802  0.5091925263404886  0.5091142058372521
0.7316070795059204  0.0005815818440169  0.4995348453521753
0.5017760396003725  0.5326592922210693  0.1614278256893160
0.4804889261722565  0.5470354557037400  0.0729066133499149
0.5748155713081361  0.5135791897773790  0.1775603145360954
0.5310615897178650  0.5429165363311799  0.0022695064544678
0.6253122687339786  0.5088985562324571  0.1071247011423116
0.6035701036453274  0.5236950516700776  0.0190044641494752
0.5592809319496157  0.5402352213859581  0.7534903883934057
0.5352228508678576  0.5466126553400252  0.6564790445406523
0.4591634273529073  0.5325611233711244  0.6371405720710762
0.5896176099777219  0.5371220707893413  0.5865933299064662
0.4368659555912019  0.5215691328048737  0.5498927831649788
0.5668233036994934  0.5240502953529405  0.4998858273029351
0.4910647571086884  0.5179186463356068  0.4829385876655596
0.4469005763530731  0.5350875854492215  0.2373540550470362
0.5566806793212894  0.4672327339649232  0.7838090658187892
0.4268946051597595  0.4648088812828097  0.2696053981781020
0.3851898610591888  0.4656375646591215  0.3232595026493076
0.4046806097030640  0.4358045160770440  0.2130709737539302
0.4755727052688619  0.4380053579807319  0.2945461869239811
0.5734518766403198  0.4624608755111713  0.8545929789543180
0.5008971691131595  0.4464533030986823  0.7773272991180428
0.5940323472023011  0.4368129372596767  0.7427015304565457
0.3970190882682802  0.5611180067062425  0.2150159925222404
0.4697843194007874  0.5649290084838914  0.2931829392910016
0.4237494468688965  0.5619812607765222  0.0594510696828367
0.5924911499023436  0.5022856593132066  0.2462559789419184
0.5136762261390689  0.5551452040672333  0.9339973330497798
0.6817231178283718  0.4938774108886749  0.1211652755737311
0.5140934715048960  0.6113767326239873  0.6474369927994138
0.4205919504165649  0.5374563336372407  0.6931977868080147
0.6474406123161316  0.5436985492706345  0.6043639779090907
0.3789099752902985  0.5160323381423997  0.5329664945602440
0.6067290902137756  0.5186022520065339  0.4456006586551670
0.4727662503719332  0.5114910006523180  0.4139538705348989
0.6148971319198632  0.5605548024177582  0.7606840729713482
0.5231199264526367  0.5711849927902253  0.7956348657608062
0.6434334516525269  0.5210183262825059  0.9642421007156422
```

MOR-Al-T4-2EB

```
 Al     Si     O     C     H
 1.00000000000000
  18.1842269897460938   -0.0090872207656503   -0.0001607598969713
  -0.0105508984997869   20.0691013336181641    0.0015994716668501
  -0.0001797722798074    0.0011982563883066   14.8440780639648438
```

```
   Al   Si   O    C    H
     1    95   192    16    21
Direct
  0.5946845468912264  0.7214282308330360  0.6499277729750018
  0.3145997158430906  0.0760068375551328  0.0431305175361507
  0.3036360207059093  0.3208147204089997  0.0455001484624643
  0.8134524176165671  0.5791784060937120  0.0398722445338888
  0.8110367881373226  0.8033782965703724  0.0407977103448309
  0.6931044164586238  0.9292275858661584  0.2899516446060369
  0.7083598831720513  0.6839049174651227  0.2894710589931777
  0.1956343688408514  0.4250566519077930  0.2957523449299130
  0.1941590045275737  0.2010250059544187  0.2923250010312982
  0.6950494694438091  0.0794325586987800  0.2427615547843660
  0.6946232121454349  0.3052170697307005  0.2445103785978004
  0.1942725228330403  0.5743680002158315  0.2481579849286236
  0.2052004534835939  0.8211732650217920  0.2444551377355398
  0.3107723779030084  0.9264593791915359  0.4958181670702833
  0.3132395511069381  0.7022446792584590  0.4976780334159162
  0.8156513369138521  0.4314291493938144  0.4947565847043106
  0.8025614701832637  0.1863595955274578  0.4904337226978093
  0.7086399787344391  0.9215397803654344  0.4975777854953846
  0.6966135201649328  0.6860642144917937  0.4972177653854343
  0.1857984948996003  0.4383159367335214  0.5009660375340078
  0.2088085950102488  0.1923229238214592  0.4992047209556754
  0.2979458303972128  0.0862644713475536  0.2509952023509496
  0.3103785313695227  0.3167014592022221  0.2531969787027104
  0.8241174017567545  0.5687538423309018  0.2421875219449847
  0.8007562958545450  0.8153796348861390  0.2463366048245041
  0.3221080566496613  0.9373091797396352  0.2905225438507550
  0.2983367949401835  0.6914599314752387  0.2909224768809139
  0.8005187679358405  0.4201842336875450  0.2865124532172692
  0.8104367360003533  0.1878322667216445  0.2830016840845042
  0.6877125184789509  0.0686712179549445  0.0375854433063178
   0.7090162459273922  0.3155951336145782  0.0380248034613131
  0.2103495895963914  0.5854919739985567  0.0397728631015244
  0.1952106898835608  0.8185154933685653  0.0373021189080430
  0.0864415093774027  0.3804212947695534  0.1413202782579693
  0.0892830782580651  0.2250893599836630  0.1363311772355563
  0.5908843356079473  0.8777203037617181  0.1458331726322983
  0.5874058774485549  0.7183443097864034  0.1516948981839573
  0.9223535185431376  0.6271593991294092  0.3895261977174427
  0.9188207594498286  0.7824910613672873  0.3865512541411092
  0.4171823897143978  0.1283892973384179  0.3995334292718540
  0.4198141605294819  0.2861705680244848  0.4038909226669092
  0.9177690735793914  0.3780981946437275  0.1362614464476791
  0.9202203195704378  0.2199029214414092  0.1320462236071815
  0.4227602176586419  0.8806711689801204  0.1482036043793420
  0.4187590613808596  0.7262095945650225  0.1545389300492266
  0.0902781117176488  0.6293094039242126  0.3878433724149148
  0.0879410322205631  0.7863518767433583  0.3847200550953215
  0.5854932805235927  0.1265767464477070  0.3946561398385517
  0.5887921435159787  0.2821276468444093  0.4005205279155111
  0.3152334590377880  0.0763222784526848  0.5422998389844061
  0.3030854197654582  0.3222699756061899  0.5462892316048971
  0.8131924248584536  0.5809326031191967  0.5430606828672637
  0.8098450479296502  0.8035109270461991  0.5398040949523540
  0.6938321808433642  0.9319616689741121  0.7893370977051274
  0.7096319021435175  0.6843425226676819  0.7971198464962472
  0.1945902703854574  0.4268358487022651  0.7925592636628808
  0.1945696012036224  0.2020861146336928  0.7919220269797631
  0.6963617970327276  0.0797797857460578  0.7418258110889564
```

0.6941481043792376	0.3061172866179156	0.7442404219531127
0.1902419725403873	0.5768938249319757	0.7469654119724486
0.2025077265443182	0.8215093135660425	0.7449813442332291
0.3114560098721200	0.9261869917099809	0.9970892631725244
0.3118652736458054	0.7026380883695598	0.9987590195506875
0.8149913168410328	0.4299732630468170	0.9929621338054528
0.8030412377183415	0.1858702336642110	0.9912539833907018
0.7094540037847626	0.9206983897354943	0.9970171416239836
0.6961136694850973	0.6851156541607250	0.0047591083303475
0.1885140686362954	0.4374666917784414	0.9979126178695272
0.2094595535392317	0.1911843241441111	0.9986043419720383
0.2987641802267779	0.0856698036132381	0.7503416535053142
0.3108180394038431	0.3191306781241718	0.7529277520870850
0.8259223882865836	0.5688530257002177	0.7483748752224211
0.7981932446468082	0.8143294561894672	0.7474697424548111
0.3198390596225154	0.9369783765736407	0.7915871591684519
0.2952609770894208	0.6916280098804870	0.7928775992670198
0.7981511272136188	0.4200278255260234	0.7861060877538802
0.8098358681311406	0.1902782275745387	0.7835788330251781
0.6874261406185324	0.0690910386908677	0.5361300562338589
0.7094937270409286	0.3159208676681595	0.5375296780055380
0.2065475541668534	0.5890628632994012	0.5397924345994507
0.1967905748061531	0.8181024010480140	0.5373698243512346
0.0843591616600057	0.3793557625718286	0.6425617640070803
0.0881008666462928	0.2242711246714263	0.6362790832002656
0.5892654541203373	0.8831065783077612	0.6473932931108497
0.9237128714096215	0.6260078697962290	0.8888663298546039
0.9177670081633440	0.7799337456428047	0.8816051320618798
0.4172311347909965	0.1274458374706747	0.9000953551155351
0.4202888074317237	0.2859030853153666	0.9047691886662822
0.9167939592892654	0.3777629243004074	0.6371608858252603
0.9194951334922027	0.2200877330283663	0.6321900644017304
0.4230264615358350	0.8822659675501090	0.6469165092042488
0.4153020670582606	0.7259499830649421	0.6534877670637802
0.0908530499947705	0.6290251754092242	0.8919544507639321
0.0866143074075379	0.7871877533911972	0.8866778805512162
0.5859987019496165	0.1249707706315363	0.8941192223620533
0.5893912604181363	0.2805278173275538	0.8996944527913758
0.1343988581363962	0.3892837350563744	0.2328136626047018
0.1174940869029726	0.2154488351553075	0.2397470138555701
0.2529906437800350	0.1221003664310513	0.4932752614659007
0.6157554122069566	0.9176415934344834	0.2364748246342557
0.6338636030530191	0.6806466284757551	0.2294941621451866
0.7391989532750832	0.6255128889261340	0.5500530182848685
0.8733817229985998	0.6179703757719269	0.4796166945497723
0.8881663418147568	0.7916633357561927	0.4888558443003830
0.7568859136153838	0.8858416270129730	0.2398310387669481
0.3926114831426299	0.0855302596318148	0.4880914978464150
0.3732900082339158	0.3267504383044828	0.4793873561427309
0.2692936912283114	0.3792895646406725	0.3018461153054433
0.8921474307058935	0.4197964428251649	0.0473976021790316
0.8753868521433614	0.1797001183753958	0.0546880204377622
0.7701442449455248	0.1235363124402399	0.2378108575143008
0.3715572619043548	0.8880000918962749	0.0591326367239585
0.3876937781276082	0.7192545496353991	0.0517752729228997
0.2569274043896040	0.6197755644055442	0.2973646235492765
0.1170344433558843	0.5846010766403495	0.3020919944468900
0.1348528495868548	0.8283983848455216	0.3108942229955737
0.2367522328912272	0.8817916424017751	0.9912472345044337
0.6344113592131612	0.1176976861745910	0.3040305024350650
0.6180769670979148	0.2916927012160894	0.2976923943564610

0.7511363867062439	0.3870200322511730	0.0439275147301475
0.8948016795705229	0.5788350382716088	0.3081768115277478
0.8691697246340065	0.8246000863956675	0.3156794294909864
0.7839929840434012	0.8786100290324008	0.0170307320923094
0.3639528448292886	0.1130460209037387	0.3146605745611684
0.3874514108510378	0.3023357240602647	0.3040586145866517
0.2454153521750833	0.3772434864246786	0.0152207037958960
0.1166842241277587	0.4296714543288017	0.0624048648522606
0.1406875129990540	0.1829491672310341	0.0673833540519196
0.2214774096614097	0.1253126833799344	0.2721081919426657
0.6423568837623553	0.8966469743310368	0.0609988983932809
0.6166755519347313	0.6953917719492282	0.0528647722148739
0.7664916842112823	0.6290472331196023	0.2537561830582508
0.1421456720440101	0.6171643454827493	0.4750841941835082
0.1212317392928931	0.8001899830417402	0.4848682706758712
0.2661400127263974	0.8768772018412836	0.2688402473704835
0.6137249945201650	0.0772917316209080	0.4744902263710752
0.6397964723563456	0.3241183584133953	0.4701391135512221
0.7248130157957644	0.3797835578989120	0.2665944949792572
0.8664769990887050	0.3936105436112011	0.2226591534766283
0.8873334750204928	0.2018590283576530	0.2309207037052101
0.7421732517068694	0.1309033522445614	0.5171723188891491
0.3953932125025376	0.9305287016649514	0.2280246515059931
0.3677833834000717	0.6835455632391019	0.2234253396019206
0.2839019545659445	0.6275615205425256	0.5205228904402648
0.2578296078474853	0.2514020640674938	0.2566936748271993
0.7444281775799613	0.7525229642158884	0.0170964666938895
0.7659654463193583	0.2591184615769045	0.0023951921412735
0.2416181030306992	0.7474049763084615	0.2533884676567727
0.7452098673669707	0.7576586324007556	0.2823222390274157
0.2659872143687885	0.2476926485310258	0.0359444927083588
0.2466117206643258	0.7525461145433543	0.0299759019203219
0.7570905934155303	0.2524244553101053	0.2760752522912570
0.0014860427808298	0.3991570673744591	0.1619920567871610
0.0058206652968807	0.1976026788275781	0.1288837989868818
0.5068502404288419	0.8987513233924863	0.1216195000123121
0.5014952963636777	0.6970624050380955	0.1589380369864024
0.0069440989169555	0.6080986318899341	0.4135532639752037
0.0028647389296319	0.8099437181141637	0.3825453448881170
0.5004968240292019	0.1083059879723161	0.3716266417253642
0.5050487581722353	0.3092495688820718	0.4078967791460267
0.0937699309220520	0.3035158092367209	0.1076191349895103
0.5963976607709884	0.7982631323700371	0.1648384172470166
0.9164254322550934	0.7042864348601551	0.3564829658750915
0.4118884139054196	0.2069771721725891	0.4249896853410894
0.9128760944346014	0.2992783242849976	0.1125624795283115
0.4167040280979536	0.8042611543915104	0.1848491091895084
0.0947825064974850	0.7075416638150369	0.3600785784992319
0.5923613121874558	0.2035315415961245	0.4285505455576120
0.1795395545241824	0.2122152830983683	0.3992526941832849
0.1611372313434971	0.4342469041404843	0.3958709442688639
0.6868421176956557	0.6655729019655469	0.3919143155508871
0.6842641669719396	0.9063438325840673	0.3939661569040869
0.8323957581530117	0.7968918553466245	0.1468942711478932
0.8517542648218304	0.5672084217724828	0.1378202249430395
0.3283623751632953	0.3341906026262024	0.1489187544544128
0.3231378237160960	0.0994465575106282	0.1472000362723858
0.8299055255729144	0.1741214638966836	0.3878401053233721
0.8259488267958303	0.4089196195454457	0.3904850880759576
0.3275530688483164	0.7120761566477918	0.3907583008938883
0.3463312123873644	0.9345277551261255	0.3959522455068041

0.1769623095190944	0.8328267963727294	0.1422070039395322
0.1842177588238955	0.5959322087401097	0.1436322889773699
0.6779382833428896	0.2967148312187667	0.1374655091580534
0.6611001141207540	0.0704201419592912	0.1421712815539014
0.2193607781336318	0.4967572869839517	0.2534545900748156
0.7153879071324133	0.0076037818525801	0.2863746448650602
0.7921716466548471	0.5081472378089918	0.9940365018419716
0.2907636829995987	0.9979755012745481	0.0408690339512362
0.7840801435958920	0.4984721464331923	0.2667896984879859
0.2818038148962056	0.0077624183168939	0.2691536849801783
0.2295028983726955	0.5077788725437814	0.0195493047700762
0.7288267485831403	0.9986088499219813	0.0161145066723079
0.1339950970855739	0.3874688792968623	0.7328230557886248
0.1175806535301138	0.2150303522244822	0.7392688391357181
0.2518160475498469	0.1200171775024467	0.9920176907402369
0.6161849008006265	0.9259086866593641	0.7355296446869527
0.6419343484884035	0.6767414945298440	0.7297084487109293
0.7384683304530105	0.6224106625816456	0.0514933433579367
0.8705756436729474	0.6195553106841221	0.9761860845186694
0.8841143846883140	0.7860142279948208	0.9828091112410373
0.7556282799808411	0.8854154259409468	0.7406932004872319
0.3922235443678933	0.0859003199366988	0.9896005697361441
0.3767247129383367	0.3260437345250700	0.9833648926243265
0.2705752492660731	0.3841645506248571	0.7966314684991697
0.8929781637534325	0.4208649135353450	0.5487816285379573
0.8736738344042937	0.1796667328872123	0.5559276265268863
0.7685428651204117	0.1275834153452043	0.7359375352790174
0.3698567353161327	0.8881946119355680	0.5594315588668807
0.3914933324375499	0.7142434584454749	0.5494674929607328
0.2540844664149760	0.6199353246964652	0.7980806893057727
0.1136288766861155	0.5868149669769760	0.8020561045192590
0.1312477667666689	0.8281517510085334	0.8096720699493872
0.2354989988599879	0.8824763371027413	0.4901520811380413
0.6335034888214369	0.1155873590070596	0.8026369002138560
0.6173284423483324	0.2909423797155834	0.7963416876232895
0.7520460585188419	0.3870480226475981	0.5438398166880208
0.9005114596176953	0.5737771713329857	0.8097042249698200
0.8712836108858779	0.8238127118516695	0.8096077332310635
0.7824148741114985	0.8795503876118456	0.5219142794004900
0.3648874957998952	0.1106056794124518	0.8150243854973398
0.3866461415484747	0.3050554473707336	0.8067393977937972
0.2454511835824117	0.3811323829033160	0.5231383974890365
0.1129513036143727	0.4289030653421647	0.5632318150831844
0.1393946075242984	0.1825744439081924	0.5666792734908879
0.2237188848893327	0.1272148346736440	0.7699042218582736
0.6400034912195892	0.9018392096145476	0.5610324545702692
0.6177212606007330	0.6987461857345353	0.5416640146301624
0.7735781027198454	0.6304650202314654	0.7744710030562694
0.1443437399731028	0.6113651437649357	0.9757249648135022
0.1178343507151869	0.8062272846347266	0.9860447346214328
0.2629793301048882	0.8769530516416219	0.7717549281448858
0.6153049731421366	0.0760317991196358	0.9737179102231887
0.6404159388866902	0.3229476873092866	0.9688071811095129
0.7208255686729712	0.3820064060256216	0.7654161304260554
0.8629624286099936	0.3921086835326439	0.7215031884483224
0.8868994591221989	0.2040127443034602	0.7318361522246277
0.7438573167173564	0.1294389532951982	0.0186795249424761
0.3927794528342298	0.9277691729354156	0.7294848387552538
0.3635243536019860	0.6853350028623690	0.7224024416307283
0.2858929056145709	0.6267883966250822	0.0212189515385166
0.2564986871764904	0.2550513316629361	0.7585640713401439

0.7485138912709471	0.7526177573140544	0.5007752204901187
0.7660387213551171	0.2600052422064646	0.4992544540142529
0.2388939468698258	0.7478484115778737	0.7558564179545872
0.7455888128703478	0.7590027548593987	0.7945692969872442
0.2628962923442671	0.2502432404273787	0.5375058734280875
0.2533007303845241	0.7553046077798746	0.5346193689729970
0.7579616133505361	0.2559948542291557	0.7796047889975964
-0.0000670087567922	0.3982451805090801	0.6663871141517833
0.0047115807770769	0.1963882788644903	0.6295299818524797
0.5052917492572322	0.9062721746631701	0.6225435658410997
0.4986382144385976	0.6908037118468034	0.6680824276188624
0.0074666087677690	0.6095063296670170	0.9200932977154526
0.0011527163777234	0.8084800924504263	0.8824493560156932
0.5006530311101719	0.1069839812490383	0.8735434120270033
0.5058586256110703	0.3078562246041143	0.9076429114429372
0.0911372056364911	0.3027454226788069	0.6077066591469774
0.5916512246367061	0.8052465899271476	0.6691871810020840
0.9180324951927832	0.7019003943978921	0.8504444011908268
0.4115190790172294	0.2064607640728884	0.9229093258241595
0.9130188186476067	0.2992996672274066	0.6109062835278641
0.4219406180806020	0.8037467003457022	0.6784021887060181
0.0976358866240128	0.7078440533981631	0.8684866993836644
0.5936644376960707	0.2020541514637417	0.9275734386903853
0.1794072185663270	0.2113989600616635	0.8991377554477867
0.1616835214923324	0.4366447200854487	0.8934223044274561
0.6811684286848422	0.6700522869711020	0.8995531087901968
0.6830668310944975	0.9094794647990899	0.8935620547238816
0.8225347532232768	0.7909219384276093	0.6469299211114583
0.8490995209008468	0.5711948307790772	0.6426027559924842
0.3322390443367754	0.3327801684089701	0.6485915771196202
0.3253237595689942	0.0979506971679501	0.6468768676038286
0.8287986620590249	0.1742910667253132	0.8879459078668185
0.8240233107225704	0.4056576673742218	0.8893784762309009
0.3275851647596869	0.7111490137123248	0.8913398195217265
0.3452937840519789	0.9356125946183216	0.8965563422136631
0.1765114821773595	0.8331032704005551	0.6417001307194478
0.1809735139549773	0.6016797794561223	0.6435426737888901
0.6799647572439461	0.2953444217898246	0.6371427177486799
0.6629505953322091	0.0687332277235024	0.6414321910420870
0.2136818791291186	0.4987322003466842	0.7473516120452472
0.7212632496982905	0.0093463381332540	0.7865203760092274
0.7917976717188179	0.5091781477718333	0.4990576722479939
0.2898887395509715	0.9986910394090619	0.5380032534098433
0.7839097300690979	0.4988481774340468	0.7691526526240117
0.2802625796176561	0.0075183853735497	0.7683201439108808
0.2218926164376424	0.5107044052886006	0.5215417593354240
0.7291529690276679	0.0000602053393653	0.5105606559029287
0.4679317283462346	0.5615625776273500	0.8770169196721145
0.4062308095012319	0.5296163355827957	0.8402745999611690
0.5282670814086360	0.5741086446148951	0.8204399626625787
0.4043285832230174	0.5107848013348005	0.7494967550611075
0.5271540242152279	0.5547369485294962	0.7297716166280057
0.4648052288810122	0.5229902537632201	0.6939387930156745
0.4211954320019282	0.5455738256044096	0.4052305005778848
0.4704316000381159	0.5156812097483523	0.3342710729025616
0.4417666568056277	0.4704978969147685	0.2710777168948578
0.5461716175817442	0.5283728758277376	0.3339142466404656
0.4878395213327885	0.4376125177747410	0.2104843858327593
0.5925213782813012	0.4958406521701525	0.2730328490797116
0.5635321764937792	0.4499867244669389	0.2117936185310180
0.4718076148563042	0.5807011953519179	0.9750600521690924

```
  0.3940464464886728  0.4913668384238164  0.4701655044598302
  0.5316507871020597  0.5421574446309786  0.0259075572012357
  0.5334510475368843  0.5569820125310165  0.0970573512905271
  0.5210988956570100  0.4882504645366573  0.0233284639753582
  0.5861297363254020  0.5513896430641125  0.9961726308800376
  0.3626225298690280  0.5126448922574779  0.5262964486794298
  0.3584790056444300  0.4557894601108009  0.4345787819112854
  0.4406672936163379  0.4626543602122624  0.4970018258668141
  0.4180142122089773  0.5720012767993614  0.0068971838217025
  0.4834651748521046  0.6344031447732685  0.9805553872655431
  0.3589124779149298  0.5197060203009598  0.8836240982822179
  0.5766549853219846  0.5991554178414019  0.8475769655776426
  0.3555607827061079  0.4862609674073191  0.7224828906191224
  0.5759943785480864  0.5627078224897759  0.6882368781770919
  0.4980641297498631  0.6447346808772495  0.6953611186987608
  0.3826712815335942  0.4606450867206153  0.2704909346180175
  0.5690479715476789  0.5640486774285522  0.3819991347347745
  0.4646440029296647  0.4022591880697528  0.1623652206939310
  0.6513597544574394  0.5060480169818002  0.2736918417913906
  0.5997585288678701  0.4246881099397375  0.1642955459586112
  0.4514702959686240  0.5844402689294429  0.4423173269477207
  0.3732550241845343  0.5696315961750024  0.3737394007250682
  0.4638448663602360  0.5077327046480866  0.6235609567937129
```

S4.4.- IWV-Al-T3-Dyaril

IWV-Al-T3-DEBH+B

```
   Al     Si     O     C     H
   1.00000000000000
    11.6056394577026367    0.0835597589612007    8.7649669647216797
    -3.6825602054595947   11.3529796600341797   10.3282289505004883
    -0.2346490621566772    0.2412826269865036   19.0520343780517578
   Al   Si   O   C   H
     1    37    76    16    19
Direct
  0.9072277810697152  0.6974069833171002  0.7038331222238963
  0.1452162352933241  0.2550861115719994  0.0527518100921280
  0.1182101218858646  0.1034371678812613  1.0159844843559271
  0.0993026393795420  0.3337128034348880  0.2844629822650404
  0.2448537988105310  0.1596694495965081  0.5625435198709781
  0.1003250367020465  0.1239796736977622  0.7821238562647209
  0.3352434169023145  0.1246552835359291  0.2840567849077885
  0.0605742854352250  0.5782773798030616  0.1408693181721777
  0.9964643002364615  0.7769978442357254  0.1338052460693977
  0.2823951999823447  0.3103006049976947  0.1001662193677621
  0.5626076343446441  0.0650009254461845  0.2500268143727963
  0.7689587823485107  0.0102458711912624  0.1110365139258960
  0.2883316203313326  0.2958470548366994  0.3284084812477045
  0.8536451203817856  0.7657687549265778  0.9501868633183002
  0.8736183996165228  0.9030251044635480  1.0047955430183242
  0.7490602116871641  0.8711685051864152  0.4443519682246806
  0.8938026093828723  0.8823422039609597  0.2387799251691662
  0.6777348226529899  0.9077570670207051  0.7107970668691366
  0.9536761930608609  0.4569180199011706  0.8478793553729959
  0.9923722390312328  0.2313406935231203  0.8906501329583277
  0.7131823149565834  0.7159633527400583  0.9021362577815568
  0.4475640299387003  0.9665651898462380  0.7501562720962668
  0.2248500972085530  0.9976386157079151  0.9077406657045614
```

0.7054207295643288	0.7347807937766373	0.6766668683593454
0.9214342789382500	0.5315249592818673	0.0829633754879177
0.5078761356234358	0.9325769723974389	0.4868402690908660
0.1038569525524213	0.5037919138427512	0.8934866444894459
0.4903840799507004	0.1284814105093979	0.5029429643711576
0.9455810307309503	0.0737719543762663	0.4840363410005135
0.9101328548358828	0.1007005044826556	0.3265285269278607
0.1139950625624886	0.9230676084991929	0.5349473958356835
0.1262918416044219	0.8909160448037489	0.7129809321419687
0.4837370146460091	0.5282439355412677	0.9416929528862749
0.3269808698456555	0.6935279313521793	0.9117150888175591
0.5398575384190741	0.4593986493138604	0.1059390474218355
0.7195122248387690	0.3019946699532583	0.1241125992578137
0.7560869154574315	0.2733174303685957	0.2853592605098000
0.2836226821383077	0.7222422937345642	0.7564312029891570
0.1793702514114706	0.1786129287873726	1.0156193485368354
0.0182913819614592	0.1824197415521552	0.1849899053730443
0.2510374784442478	0.2773716971175695	0.0507410290475495
0.1350307938999019	0.3797158602147810	0.9583103400245103
0.8071978820402491	0.0099312398533776	0.6042905976310295
0.1753626385449640	0.1938011789372613	0.6426328257830416
0.1889556835393118	0.0183790770006663	0.6518821598939558
0.2242959772287494	0.2390030357233529	0.4620547023510297
0.3874517302814323	0.1911648856869116	0.4968494328005009
0.0476765877634445	0.8016740060905434	0.5937114962233456
0.9944089771659766	0.6409071546854002	0.1933532642278019
0.2003616486179808	0.6570952363088883	0.0311409642222464
0.0456385550116080	0.4456426445563106	0.2507150547248714
1.0031353676083352	0.5762970055615714	0.0907442625162790
0.6032623296763076	0.6056583012462329	0.8053097190539289
0.6324159475584040	0.0049010679576953	0.1951489361271480
0.6605517025976688	0.1718142042498348	0.1874320670489650
0.4709801318366811	0.1167522965424430	0.2183644152632410
0.4875146253771445	0.9689471898294675	0.3951386227490883
0.6003960149929394	0.5935453996402321	0.0335357349256604
0.8155059596595218	0.8244116283556026	1.0062147559273809
0.9857569794100364	0.8479345890921983	0.8211921511716807
0.7560948728347411	0.7667239822448585	0.9339196587558397
0.8534618817302694	0.6292598107755873	0.0408114630090053
0.2422314519037729	0.9913478218135741	0.4010714839154772
0.8120637294329389	0.8256850091659742	0.3743382454959132
0.8477991785662288	0.9806406297096444	0.3771924889837434
0.6993027226932634	0.7603986582786638	0.5810596208308869
0.6411619506890865	0.9187632865576854	0.4384411535728880
0.9843322834146553	0.2152472369422723	0.4097336743560938
0.0009769311874423	0.3686520788734494	0.8234159152906032
0.8314127696034700	0.3739850602766944	0.9865622934990061
0.9284021066643547	0.5588270920712822	0.7570860573655693
0.0547817487091575	0.5094196853015999	0.8339472652482887
0.4078199663452851	0.4063295762179855	0.2321104600138483
0.3600450039791143	0.9933790053493130	0.8354414694338498
0.3892249507139913	0.8348201271810312	0.8163758302291625
0.5755676910252346	0.9764033669774733	0.7230903746700609
0.4614418730858272	0.0623012851687914	0.6280063071354680
0.4001021769471048	0.4269495010779600	0.9835509201574959
1.0047230935715463	0.5153239423639966	0.9894115140588466
0.4963877221326661	0.0358805319155155	0.4890334265918025
0.8223005033885725	0.4043539634403180	0.2161536817860021
0.0516480767741017	0.1804577352178744	0.9572081182981502
0.4085810178963672	0.8070592844918154	0.6205083675314705
0.1808978766037523	0.0597564982266821	0.8263737026685655

0.2274819136819496	0.6130044329433115	0.7879743029748911
0.9337476475232798	0.8259175072981193	0.0709237406290230
0.6161372074695932	0.2352453986665174	0.3928734077810362
0.8224851115471178	0.9472497037527861	0.1838126268578215
0.0191100162808713	0.9791849129516028	0.1536415436959395
0.9607420930269709	0.0650763171567600	0.3969805328487992
0.8155734769069070	0.1750883033029834	0.3437445880435355
0.9777163570899674	0.0242149352540993	0.8662292183021680
0.1454983774537510	0.9034385530680515	0.6137593999313448
0.1888403907113984	0.7970966509247904	0.7701169660112481
0.1346141209952148	0.8621235175623075	0.0331759771478987
0.4066391208801983	0.6163391051645974	0.9531815506838610
0.3077210483075656	0.6665410869554020	0.8515133305614214
0.8531403454198064	0.1487393510850801	0.9888720563039616
0.6202477184521915	0.3727202618674892	0.1367150659204722
0.7685255047449699	0.2796651717561794	0.1913110359110244
0.2226733038031590	0.0738085659176213	0.9381125861984819
0.0663271211014079	0.2253489722316262	0.7881924661868351
0.9234414982869350	0.7744251658889535	0.2415544470487140
0.7698878569267060	0.9401174665223320	0.0750083677677538
0.0343349110016977	0.0170926546478133	0.5181993882404093
0.5189573916170933	0.4595090513158792	0.0306585915884894
0.1686662961341064	0.3378434781072495	0.1756804761142261
0.3262175221871141	0.1949370877736836	0.3223017905667961
0.8181574331443132	0.6925659520514922	0.8222655308824998
0.6646289381852584	0.8400335546446316	0.6783947680456779
0.1940706898273369	0.3449271340119105	0.2996993865430131
0.3094846310067478	0.1980167984855332	0.1879903892415312
0.8347277323195248	0.7338435138385980	0.6435779949786589
0.6565009013989974	0.8133243920828609	0.8372809131117908
0.2473125801172268	0.5872105613925369	0.2946376818426061
0.2192599189545218	0.5106480856024782	0.4085063463762212
0.1547968457010983	0.5876987875304680	0.2884330210493990
0.1045662327309470	0.4354907201237656	0.5124629215080863
0.0398454499521299	0.5135514861583548	0.3911423206053125
0.0118574081433777	0.4333878142934420	0.5065062567279072
0.8970717617327572	0.3512167102101031	0.6080292453007319
0.7954392078586251	0.4741159447656810	0.7162181025610304
0.7224654439034988	0.3795404276975345	0.8405891745962377
0.7613630906320614	0.4948999384045445	0.6519846365616394
0.6155094808251329	0.3053678415456323	0.9012530123839498
0.6536866755817862	0.4212617551128777	0.7127378431322511
0.5810342866712389	0.3264097115539170	0.8371065650893128
0.3717592000376350	0.6668640625924807	0.1804166177203115
0.8556026018264700	0.2506347698635972	0.7307933953217047
0.4653998695568851	0.6719651875085689	0.1917271998812378
0.5521179035036746	0.7339995312835185	0.1025979376710602
0.4395519728500611	0.7019465735736392	0.2374679421808763
0.4783213235659484	0.5840691109786524	0.2419626844665760
-0.1587007729896885	0.1675154345332262	0.7524560506607203
0.9169227718723244	0.2510351139122034	0.7461881062234449
0.7659277369835635	0.2457346112958206	0.7945616698125120
0.3659907879462021	0.7569146557984354	0.1217089727414798
0.4009731853009835	0.6369746138878757	0.1336602355963731
0.2896348033403374	0.5106089327015197	0.4140330344402753
0.1761757102188906	0.6473403196041729	0.2006122685738090
1.0854471382871977	0.3772227931638804	0.5989829955167421
-0.0302940531507289	0.5137457922156340	0.3851391905511874
0.8770509397908827	0.5324016308066140	0.6706599707050103
0.7484352455899227	0.3644319826158592	0.8904667529889966
0.8159900357996591	0.5712794499673183	0.5552780117252836

```
  0.5582905813440329  0.2321898792563318  0.9986526230853978
  0.6261121558685783  0.4393815069001799  0.6627416027035613
  0.4966224718620152  0.2699667306854306  0.8847278463878452
  0.8343242477414397  0.3562547151407048  0.5920761476972558
```

IWV-Al-T3-TS6

```
   Al     Si     O     C     H
   1.00000000000000
    11.6056394577026367    0.0835597589612007    8.7649669647216797
    -3.6825602054595947   11.3529796600341797   10.3282289505004883
    -0.2346490621566772    0.2412826269865036   19.0520343780517578
   Al   Si   O    C    H
     1    37    76    16    19
Direct
  0.8965613787582367  0.6703741381273429  0.7281756434652461
  0.1427379015166325  0.2262357209369802  0.0749835728745739
  0.1289278636478721  0.0892941573971336  0.0214544199719797
  0.1029015767025614  0.3150787476950594  0.2970287886213797
  0.2511490838099849  0.1325852410780287  0.5759283762272396
  0.1064254599826087  0.1071291756991878  0.7914168546840650
  0.3282916772984431  0.0945805788019162  0.3035611564499050
  0.0500794786590917  0.5549831604202088  0.1630270840936155
  0.9998463807539916  0.7600717672505549  0.1470837310936646
  0.2804351069987871  0.2846667855876999  0.1192517437043649
  0.5557984924402564  0.0423648557409954  0.2646928385345467
  0.7766044513855168  0.9972378462886550  0.1207602121393283
  0.2930134974010856  0.2706603401212245  0.3414364565614201
  0.8505483586269200  0.7508035860332664  0.9653330936310557
  0.8783146667378702  0.8863570844960020  0.0173675113497036
  0.7431643078387972  0.8528395720141483  0.4602521550038821
  0.9015665420006150  0.8719873983888687  0.2475695253121051
  0.6800129300759791  0.8919249247568050  0.7245114204076843
  0.9511179801011228  0.4296585409758721  0.8710313855935877
  0.0024495735012788  0.2166072506724750  0.8960591587189464
  0.7073550849279411  0.7010002057556445  0.9192110053119960
  0.4471936054088022  0.9373997254890964  0.7705591549485536
  0.2298913154284813  0.9798718336644434  0.9192217674562863
  0.6995312467508482  0.7128314910912594  0.6953091606139485
  0.8996924685835073  0.5073840851406541  0.1127376306308634
  0.4980489648690534  0.8959905118198949  0.5052871302806428
  0.0869299418111043  0.4711724377675580  0.9307693531448256
  0.4872725144195570  0.0853058997161323  0.5272566150826203
  0.9451960985857734  0.0535615326439719  0.5013833881230529
  0.9112443659278563  0.0792846806657863  0.3432572615404827
  0.1068308055599268  0.8969616098889123  0.5557618127842636
  0.1241697542352887  0.8633816996900942  0.7322386976111099
  0.4772843309629371  0.5027540663717313  0.9645007200746785
  0.3211216554403987  0.6653324462397094  0.9343882440271076
  0.5458601607492931  0.4450423844838594  0.1171227784244548
  0.7157907851013310  0.2774800332010190  0.1425843280703858
  0.7548894551101286  0.2477741526631961  0.3024765445448105
  0.2805245991254293  0.6926886133820158  0.7762055945559490
  0.1907670297179236  0.1692810355777845  0.0166459651992249
  0.0304243161150474  0.1255559938856571  0.2156385805067952
  0.2535413444026693  0.2653997009587908  0.0585628309618067
  0.1005033714714327  0.3391258487251773  0.0086526279365927
  0.8091188524303393  0.9920333286510669  0.6242523314245869
  0.1836632368089696  0.1682217988957846  0.6545004548642840
  0.1946494522830126  0.9913469551253417  0.6643525685498580
```

0.2312032845706381	0.2115273914126895	0.4751604943626780
0.3945390192598493	0.1608618576607787	0.5104071470524452
0.0342920436298979	0.7809352325806880	0.6109410933650830
0.9950948099862225	0.6226896214430898	0.2105726404383655
0.1847373486658617	0.6380825355447196	0.0385122761313454
0.0521031262601359	0.4285059095134294	0.2649732410208611
0.9702289164922736	0.5373401406855606	0.1385006304367323
0.5835237176733625	0.5930802138277781	0.8220148711360107
0.6398071626725689	0.9937102669231163	0.2006313722376026
0.6372957998251122	0.1520952157713617	0.2135398985242379
0.4629228705235432	0.0862117573277355	0.2339237839134645
0.4805927036073360	0.9385667804822682	0.4099810875580771
0.5968972543412332	0.5781570031711681	0.0530137734170605
0.8152866916721272	0.8105701855017668	0.0195895275029191
0.9860614297236988	0.8266151884222439	0.8403579429433097
0.7571499244671237	0.7568314915106201	0.9429411226820249
0.8428377205968677	0.6125379788204485	0.0605654013926994
0.2373925566552985	0.9607528010782648	0.4247939059263757
0.8259086182595892	0.8171274429363171	0.3820923861959752
0.8173059076104990	0.9719178381925265	0.4028023700231539
0.6989374530493856	0.7423589758893953	0.5951685357003877
0.6298131101095333	0.8777243106127707	0.4592577969737807
0.9870365316066194	0.1967439575706890	0.4197797062417041
0.0112842824214118	0.3534503272962453	0.8325814874524361
0.8310039940557659	0.3334117152161180	0.0106883531049455
0.9205350823066019	0.5321405455371697	0.7855129946363341
0.0447117300231052	0.4831415423186710	0.8636695439003335
0.4126011538925095	0.3803991799399468	0.2455637168413423
0.3643073386644954	0.9749612787574824	0.8462595194455771
0.3956370652129194	0.7962014658018707	0.8558747284682331
0.5816149743190073	0.9651627255849018	0.7260136699833679
0.4444670531595155	0.0100935670145642	0.6598110463529310
0.4084322146616103	0.3889321868618983	0.0094073587697623
0.9900330600682310	0.4918653380639382	0.0190746976073108
0.4875776790541263	0.9956964875146824	0.5116887616530867
0.7900429820260465	0.3841250624416444	0.2400373278480344
0.0636185367471475	0.1635970233444543	0.9612166769158078
0.3958064522204227	0.7701365877419198	0.6363491958488525
0.1848862438650894	0.0441567050674049	0.8388618567227547
0.2171753480751046	0.5696287932453881	0.8305845654005899
0.9381216920579269	0.8063149875249277	0.0835760899248111
0.6201133412095429	0.1776388999614232	0.4285725048867454
0.8266845299373294	0.9373805945441935	0.1966897421266893
0.0291356939959258	0.9669990625511479	0.1603603624546269
0.9496589103349842	0.0337797710175595	0.4253857391301258
0.8501404294527549	0.1848502349144586	0.3262574632260979
0.9828962943446778	0.0064415893889847	0.8787797094833224
0.1345598060027435	0.8689378622849817	0.6401432383100653
0.1850062833317878	0.7667288885917394	0.7901585487123216
0.1380901516688452	0.8452850845830209	0.0455751889055265
0.3792754264244325	0.5689829473288385	0.9963398557318330
0.3251915010626171	0.6588431875684100	0.8508891761593076
0.8625236728270651	0.1344654164579020	0.9963743426206244
0.6346872528310028	0.3667139956342797	0.1391528184921073
0.7624713824744264	0.2470046538457996	0.2144039231939494
0.2336335766337567	0.0562125504117012	0.9476622572961900
0.0738539191523372	0.2116931097705805	0.7931561639826330
0.9264968477609953	0.7628652731573262	0.2516844435346097
0.7772075536348396	0.9227574432149737	0.0892948047060550
0.0365356050908902	0.9990638365114279	0.5323752082531711
0.5326328109228798	0.4514483431816227	0.0343710282152767

```
  0.1716370008479010  0.3219758741051499  0.1862690021569166
  0.3291676882086790  0.1714239449441001  0.3324884374083983
  0.8084088080689512  0.6761255145010986  0.8387599075410938
  0.6759489467085823  0.8239680144108308  0.6886163007033117
  0.1974835229961652  0.3208820523934800  0.3145500095320370
  0.2905683170433086  0.1618361361277786  0.2166915247949046
  0.8215518700023036  0.6887098019844486  0.6748916572143134
  0.6482110929701299  0.7965179526056134  0.8559778881516789
  0.1965842693846345  0.6783016614021568  0.2641966195034337
  0.1522890311564713  0.6993364621609630  0.3385735867905078
  0.1328467191724551  0.5759449152237566  0.3119777286503004
  0.0486160249825003  0.6206390410695832  0.4567038895279220
  0.0267816564512512  0.4988068126291630  0.4287783467430180
  0.9843433792618717  0.5185831064020640  0.5037819962539046
  0.8777834992134667  0.4312734242029717  0.6289422766040551
  0.7477948130488090  0.5066182253357830  0.6407673454044633
  0.6471450516999674  0.4250159004847943  0.7654982725158764
  0.7412937146547708  0.5231819511989171  0.5603826478145955
  0.5508002213190500  0.3559178945092674  0.8085625553431424
  0.6445994100530729  0.4540448748900311  0.6040940960495637
  0.5499004769063249  0.3698683074177042  0.7280898875853890
  0.3109939995818993  0.7615879874777710  0.1377198501212866
  0.8797346490477975  0.3975661491927153  0.7225206570256408
  0.4215717018248742  0.7315774666883206  0.1325420127394141
  0.5040660708076360  0.7935603720029419  0.0393610306952189
  0.4255516805609879  0.7378903390776397  0.1848423782446394
  0.4189697314025507  0.6406418932638510  0.1689721573616402
  0.9574421288190762  0.3640459694927936  0.7122732765751174
  0.8937503979256542  0.4730776469009025  0.7108251185818453
  0.7985379200156688  0.3267439742045989  0.8136002912344431
  0.3171471958206351  0.8540285825487148  0.0980573130383150
  0.3097298641246478  0.7562171943558509  0.0836064504215109
  0.2004692884796964  0.7790068763274735  0.3023153361602300
  0.1664053114441890  0.5576672046582879  0.2560835536131810
  0.0157328442272063  0.6386476396922000  0.5128118191368889
  0.9767919347137527  0.4212968964374342  0.4634496958345291
  0.8016269651011051  0.5817113311039080  0.6062523878278432
  0.6475069328232571  0.4178154468993635  0.8262616878572216
  0.8160275522854105  0.5878118697780860  0.4647476882896495
  0.4744302410590790  0.2935039698901776  0.9036222837359096
  0.6414867360540808  0.4648456351628013  0.5428189400944687
  0.4739012421913180  0.3147926419615141  0.7626741257164262
  0.8381423914291802  0.3562522718293257  0.6529541777315531
```

IWV-Al-T3-I1^{+}

```
   Al     Si     O     C     H
   1.00000000000000
    11.6056394577026367    0.0835597589612007    8.7649669647216797
    -3.6825602054595947   11.3529796600341797   10.3282289505004883
    -0.2346490621566772    0.2412826269865036   19.0520343780517578
   Al   Si   O    C    H
     1    37    76    16    19
Direct
  0.8893825845589514  0.6832428012765558  0.7215907862422091
  0.1342742555801460  0.2398753056016276  0.0619360143732348
  0.1182575776603873  0.0992393036460577  0.0136952053985522
  0.0929542718902608  0.3269996882113879  0.2871171840244112
  0.2409264557439865  0.1423488026487356  0.5697732931749839
  0.0977044359950188  0.1184960921490876  0.7818299036276201
```

0.3199695204017097	0.1091476144375045	0.2891142661816340
0.0434553463869902	0.5690069370991059	0.1507916165647837
0.9888852148863653	0.7711362763503402	0.1370983150712031
0.2747133473550806	0.3018310413931214	0.1028936600624108
0.5470679457168255	0.0537259937429313	0.2555709274030169
0.7658232887163867	0.0072160188264489	0.1128559402792836
0.2785993506401896	0.2776044424951263	0.3369487388611793
0.8427901755407111	0.7648415855455242	0.9518474540585230
0.8696901125919129	0.8993823174707327	0.0055003424354604
0.7345084153535647	0.8624311803206102	0.4514060472435170
0.8897046983426724	0.8817117083811848	0.2392828486358718
0.6704357078908082	0.9029111344364000	0.7152915345828927
0.9385948138110177	0.4423456512393342	0.8576392318737709
0.9927380353354716	0.2262094564933032	0.8891425348432650
0.6974941093083263	0.7035750800318026	0.9153748216532698
0.4395401010777991	0.9523059147492652	0.7575651014072169
0.2185043011771585	0.9888200506767320	0.9109961511080547
0.6932463194603696	0.7320019147861758	0.6826689398373172
0.8960145785361274	0.5221568868725029	0.0977831399337801
0.4872301022264846	0.9064946870602488	0.4987781047364442
0.0759667049325950	0.4819719462005304	0.9181010278674466
0.4810993372211095	0.1020341834220179	0.5126851269800169
0.9333465051895589	0.0672097110169565	0.4900427559873110
0.9015877976800418	0.0921804964095271	0.3309057612940492
0.1003393951308748	0.9159697999022990	0.5429345987476450
0.1161911376504670	0.8792394362033312	0.7204140326709294
0.4686094809563956	0.5195830628500387	0.9518753674936413
0.3103563118391948	0.6811448760312111	0.9194666815244414
0.5285775357907284	0.4533502218685841	0.1140797874948491
0.7053939682994476	0.2928173068923485	0.1282194588194884
0.7504864843492419	0.2658680105363312	0.2816907977443521
0.2657077072080794	0.7008152884117360	0.7675887221188913
0.1816429499385069	0.1767632947862213	0.0098664468079731
0.0228166851159411	0.1409913494343168	0.2032803950312762
0.2437142026829794	0.2851537965646500	0.0431219810393412
0.0904272619684352	0.3497656010886559	0.9928758295137959
0.7982524869259573	0.0049463836461246	0.6143873432255571
0.1743890001367793	0.1838445246162113	0.6434220706470415
0.1855921783872570	0.9986392648002064	0.6670191616130757
0.2167597204736648	0.2119072588259563	0.4728847473618152
0.3848234332282034	0.1739495964246240	0.5002041771401406
0.0264820306139828	0.7907923107501593	0.6133740170847235
0.9841895318824989	0.6338652507881493	0.2011057292014871
0.1774899441796289	0.6558693959415894	0.0260258079015744
0.0460799589830932	0.4424603574757894	0.2513406721684340
0.9666697678999167	0.5512411010029803	0.1236438788375490
0.5816633793009224	0.6089559897058832	0.8110295276641961
0.6288334903566247	0.0049263065519319	0.1907638623635814
0.6314831840166106	0.1628581204879921	0.2029036990062831
0.4538902314266146	0.0967908612335060	0.2252289224879838
0.4733897980564171	0.9511978815569401	0.4004689113653384
0.5820367732899003	0.5859300035528148	0.0497290838847264
0.8076882172540736	0.8256155426948978	0.0052714428899657
0.9750721522818981	0.8438690160759418	0.8225161433433560
0.7450453729786772	0.7649417058853762	0.9361145788892724
0.8413759837194034	0.6289767180831146	0.0439887312510077
0.2250298696254343	0.9762977531245451	0.4094218528619192
0.8142632484213337	0.8261240128438997	0.3738730387423433
0.8101685473949931	0.9842941878265182	0.3887178413249427
0.6975536653446394	0.7531918276841234	0.5839456695760005
0.6171851461811988	0.8847749252155663	0.4571091690707831

0.9747497322799820	0.2105061000779372	0.4070920211823841
0.9989489826953998	0.3636919115854462	0.8231742333600699
0.8107127961836710	0.3544124118301815	0.9915932582023049
0.9174199918569724	0.5474104826599578	0.7613200947937978
0.0256471498328166	0.4957378854483578	0.8553718182513207
0.3964748869896068	0.3880168287453438	0.2434531575455765
0.3521619075340858	0.9828557809622872	0.8388346989116441
0.3858889051707903	0.8169645636765677	0.8281344506221548
0.5672644761266558	0.9694240524013188	0.7288988148190896
0.4521722739865250	0.0403028651958442	0.6364069847534456
0.4010092752912708	0.4091878361464591	0.9915317360967555
0.9850575334474455	0.5039004012267541	0.0062485724754665
0.4744404794944579	0.0039391131589760	0.5084447765626896
0.7850367516979285	0.3998973771256180	0.2241818327196436
0.0514444511140709	0.1771850823285719	0.9554196742694070
0.3830074744203621	0.7814920453934094	0.6281518079750440
0.1739913269243307	0.0499254772402225	0.8303232026185290
0.2066040945596395	0.5811879145382837	0.8141240410015231
0.9314429537742001	0.8196155317227195	0.0691234177711680
0.6131191723707322	0.1979818427508324	0.4023848676248436
0.8130438626677658	0.9479388186429006	0.1905389010755772
0.0175185427567456	0.9765121163325743	0.1519339217531571
0.9362215962041428	0.0453195597351500	0.4161201393479989
0.8396877582442662	0.1975260880431414	0.3127042435909859
0.9712967262920141	0.0224265504963054	0.8670938525825106
0.1397438653381970	0.9046215706635208	0.6129112597056342
0.1683866626950749	0.7733147850579373	0.7824760652833493
0.1269659503147043	0.8550460374548590	0.0382356676900220
0.3737493160516647	0.5904314350069522	0.9777011520479368
0.3043049124991334	0.6614390100480315	0.8482581721477384
0.8531024155762555	0.1439912745393352	0.9886816143612758
0.6164621261072192	0.3731666341314657	0.1360022446098224
0.7662009113187020	0.2719257314463327	0.1861008021708968
0.2205010065824397	0.0681340583748117	0.9369534515218738
0.0674434854733003	0.2212331272181141	0.7862927814383028
0.9131796444971600	0.7747602204774513	0.2408360033294332
0.7680520940974462	0.9339149628749611	0.0795951522883572
0.0256998423342887	0.0137430616939861	0.5198360543664079
0.5141544771886750	0.4617021992238931	0.0309812406077931
0.1660774055933457	0.3339922003362414	0.1759128692075604
0.3183851744332312	0.1836451142745070	0.3208366614826341
0.7984840561014862	0.6670038341006846	0.8464632826995145
0.6632840802253616	0.8446303210183175	0.6720860245343490
0.1819521308851220	0.3274677529241951	0.3122087320793443
0.2909247360724673	0.1796483736898274	0.1953754791538229
0.8164129487928024	0.7164552060874807	0.6627561238642300
0.6467213267527596	0.7991157543132118	0.8424979538966733
0.7140475254422396	0.2496066162105017	0.8646979172614532
0.8380142955506358	0.2683504570112482	0.7917664179816152
0.6672561460710339	0.3454397572651102	0.8410275066096993
0.9167205302546394	0.3825809747204935	0.6969435478143627
0.7449804253676798	0.4602855832284821	0.7428133027102877
0.8738268421907899	0.4845926956860792	0.6697666099749668
0.9452197107060276	0.6042488797358645	0.5335232960917584
0.0784347496414242	0.6452020339456422	0.4648584916893108
0.1591974677645805	0.6013356166695144	0.4149539584596739
0.1252251668807351	0.7352319272077651	0.4437667095163534
0.2826586180804579	0.6478113837442665	0.3444665098695290
0.2483050346336822	0.7810625484683087	0.3739922994127713
0.3293390752037326	0.7390759135643601	0.3219566926350795
0.9156309899649447	0.5882368994665558	0.4771635865621907

```
  0.4625803728246491  0.7956053346764048  0.2392619998353399
  0.5053921451650749  0.9207139631636667  0.1178278894814602
  0.8713211578227266  0.1922239347394733  0.8116802224506063
  0.5708902802429338  0.3298361954564993  0.8978764888741599
  0.0133009069397207  0.3981440938533353  0.6417177551417068
  0.7101031556927613  0.5357727863384932  0.7201811428054323
  0.1262079204510244  0.5320579143761852  0.4284985145100937
  0.0645790490016770  0.7711142346790900  0.4812740946878050
  0.3440627762226846  0.6135331061737619  0.3051209530672341
  0.2815514381348476  0.8513453614425335  0.3591431441675248
  0.9076379390184193  0.6717948370451184  0.5348724926703952
  0.9410570713559416  0.5129885141226073  0.4852177087884380
  0.8191976303731453  0.5717224919552686  0.5211338940646943
  0.9645193303686257  0.6688894254297492  0.3816825491666129
  0.6529206605052635  0.1585967798437002  0.9410507158216590
  0.8948695494325122  0.5100784154628299  0.7068942821174919
  0.4621312144076040  0.9804682529150560  0.1303377163301139
  0.4829137711079336  0.9169802808539710  0.0738937311661337
  0.6030724671782891  0.9583685770116311  0.0585050140481125
  0.4858538876055409  0.8030140327112554  0.2808343727661529
  0.5108207398764444  0.7387387733899261  0.2245211155078968
```

IWV-Al-T3-TS7

```
   Al     Si     O     C     H
   1.00000000000000
    11.6056394577026367    0.0835597589612007    8.7649669647216797
    -3.6825602054595947   11.3529796600341797   10.3282289505004883
    -0.2346490621566772    0.2418282698650369   19.0520343780517578
   Al   Si   O    C    H
     1    37    76    16    19
Selective dynamics
Direct
  0.8590375601096796  0.6990248298456126  0.6976624568247087
  0.1035999122431675  0.2585652615913255  0.0425767541123069
  0.0819114131545132  0.1139894679898449  0.9982976736785885
  0.0622293801654492  0.3425925320565698  0.2693074215124975
  0.2073378250192230  0.1634882873859296  0.5482068746527705
  0.0614906934768438  0.1306966384220075  0.7680935558825605
  0.2875030410103855  0.1246609592830005  0.2764216486865235
  0.0144208651774980  0.5831297667464671  0.1327852897790756
  0.9548276717665771  0.7844841862105352  0.1220150802046969
  0.2408782653949718  0.3139387670314431  0.0897968125274597
  0.5155179671821155  0.0716655445698142  0.2387121922668727
  0.7308586983240729  0.0220641675098423  0.0961877848352976
  0.2510656685517360  0.3015238141180517  0.3134856600917694
  0.8109317985882858  0.7779127670455551  0.9369837982879953
  0.8322293989165618  0.9115337728353009  0.9931126424443155
  0.7033919907135981  0.8786507233702939  0.4333952582658077
  0.8564521581796409  0.8949352465815659  0.2229336900517656
  0.6380213172605808  0.9187783338678295  0.6963860037654406
  0.9100612491504211  0.4582700224439810  0.8412265665136700
  0.9560701560399594  0.2410600621853232  0.8731318321785626
  0.6680913144548927  0.7284824739121335  0.8897399048061363
  0.4054042241749679  0.9681413207998546  0.7411456953515008
  0.1849677984534527  1.0042487478280901  0.8942789664878725
  0.6626596357598555  0.7406032012984296  0.6652906048370754
  0.8708286409868081  0.5388553738984188  0.0755486405571354
  0.4607125219298068  0.9328315542000036  0.4764173345965844
  0.0536601519962784  0.5056766271358013  0.8905364959123141
```

0.4473643412463263	0.1254281200470880	0.4952730362112374
0.9057730518650395	0.0788407588490564	0.4734831467503584
0.8703962956099851	0.1068717268511343	0.3157848310063367
0.0675108354251791	0.9215176253232569	0.5300587304204938
0.0837130861449200	0.8930869361591309	0.7043165162183560
0.4422128657402677	0.5304053285778376	0.9338441752330462
0.2836151912715678	0.6935986189050453	0.9056702702025954
0.5048772152695451	0.4714856958645187	0.0903229069703564
0.6773583607483444	0.3066377999700425	0.1153207691449448
0.7146726570517048	0.2782886132495725	0.2758345392195931
0.2390070165351135	0.7240807468365297	0.7490512357364367
0.1446950077402635	0.1916200117598461	0.9947693309823185
-0.0158356286235038	0.1689808497880714	0.1807199634773119
0.2116399858232838	0.2911041484803992	0.0327804232320038
0.0748189276101969	0.3770111585171351	0.9632780806898863
0.7667775338157358	0.0217593292292071	0.5921158536446787
0.1352165389387287	0.1955301379685100	0.6301363443436105
0.1548207626507201	0.0227334768281213	0.6331529341062783
0.1871807749323848	0.2450433126515620	0.4471689357732701
0.3502139946375683	0.1956081088357781	0.4823424993937120
0.0005721554024060	0.8024626236055946	0.5854235541021389
0.9514699295559884	0.6476759162118897	0.1835135438444540
0.1529667562280426	0.6633230792885646	0.0179548319956586
0.0044036522861904	0.4515288992796714	0.2402643783330285
0.9497955473198818	0.5761379971615371	0.0902989258880708
0.5547173400001902	0.6151874218909443	0.7936557763303966
0.5948323882850854	0.0183613719561291	0.1782970311486919
0.6025770616798808	0.1812353589062990	0.1829284683303467
0.4227546427881586	0.1170996759394157	0.2080193149330622
0.4404813492112830	0.9710952007424714	0.3840911708821817
0.5564452961196480	0.6062292757503163	0.0228252398455726
0.7712790598594976	0.8356146732954505	0.9940115987631334
0.9451510121598420	0.8593087397047005	0.8102279660898397
0.7171921118725995	0.7821419673573344	0.9155891380125999
0.8086713594861924	0.6410086307307197	0.0285555486317022
0.1964725500235038	0.9907405596652767	0.3966958046685586
0.7782125543072517	0.8397621132462207	0.3576729379733545
0.7876573855681869	0.9930690298367385	0.3737219695686447
0.6564813181826837	0.7673653792134998	0.5691903959875040
0.5921447530870956	0.9141653568705813	0.4299593980953795
0.9503548166129822	0.2209417053221630	0.3943269575745074
0.9644949702454818	0.3785221547862922	0.8064116607538553
0.7941854966488047	0.3647335539979861	0.9832735914468531
0.8740480409619131	0.5571026160686223	0.7595342577591524
0.0101135601362021	0.5154912896143287	0.8251940712446865
0.3719478232335857	0.4091966947565370	0.2188969841542973
0.3196192403215889	0.9980973008591996	0.8236177556958911
0.3548454570564434	0.8306501054674320	0.8154267463546061
0.5385991073769882	0.9910732785996071	0.7011078146501377
0.4070165936274698	0.0518223186387740	0.6265026341571118
0.3668389617176420	0.4207471757694710	0.9787111731956837
0.9542131090849332	0.5215959541140823	0.9827184197283556
0.4536348695832763	0.0369342878554709	0.4768478002718913
0.7652037788841000	0.4144012680988706	0.2067910761741145
0.0135945681768722	0.1891520293856496	0.9413092816179468
0.3589851352587897	0.8088756436134343	0.6102489824212499
0.1421094059046976	0.0658116219249752	0.8121091815696622
0.1803432724241708	0.6097981392642940	0.7884369937848258
0.8922963899734028	0.8322639064178438	0.0589061892201508
0.5767893887428298	0.2242375044379036	0.3943851514275329
0.7843238315745636	0.9616735464342829	0.1689409571109869

-0.0163757356324765	0.9899588517670126	0.1368820942562988
0.9165331205089876	0.0645120683383965	0.3916258885105158
0.7949552185625288	0.1996557494974384	0.3158310849939200
0.9365114027497163	0.0323987532047191	0.8543744399273800
0.0963394311438464	0.8960054163726661	0.6128285424279600
0.1427640728661502	0.7972721961244393	0.7653659126532419
0.0929396701216438	0.8699091634491558	0.0206846148907222
0.3531691700124844	0.6065529236950237	0.9567684565374208
0.2726184480976556	0.6755454474159392	0.8357875339962695
0.8160937343885256	0.1596041983252451	0.9710441438104993
0.5935140397865860	0.3936001591352406	0.1138425178306164
0.7218109785519827	0.2759425953011721	0.1888663953599338
0.1849794079530362	0.0828054111363470	0.9215833138418510
0.0303544890500249	0.2338382921435629	0.7716478328107731
0.8820966410982962	0.7856873318062596	0.2274040976727110
0.7300583921194281	0.9486791622768787	0.0638478853290856
-0.0104280997062537	0.0171734547519170	0.5132619432207356
0.4894553486331149	0.4723079020808920	0.0117785937560823
0.1315386921925512	0.3502006058116394	0.1582586161987108
0.2843186876305193	0.1993703094842812	0.3088762861876156
0.7694518197761685	0.7044896321876694	0.8086881574687300
0.6309275390007397	0.8486530837942908	0.6637321999537580
0.1578513469358110	0.3542797718209432	0.2836058783138267
0.2528590594827203	0.1930804432796041	0.1872733233975734
0.7900747227882878	0.7293193726548505	0.6356737397926049
0.6106752743880490	0.8264317982972645	0.8257144019981928
0.7322222894496676	0.2567621345206031	0.5905624902762540
0.7446603428826346	0.3758343872289108	0.5349477595012908
0.7335641687461730	0.1759178869297979	0.6885328906470798
0.7600734244268382	0.4155137649685695	0.5755750324738451
0.7494746534666206	0.2143529311682276	0.7294500740212364
0.7671000361442566	0.3357838988304139	0.6713511943817138
0.7971669891428855	0.3815654619255229	0.7084570777736094
0.9263610839843750	0.4628750979900360	0.5940690636634827
0.0064309684746063	0.4114811843417117	0.5506687883566771
0.9601666556706254	0.5872001807982359	0.5171967414347126
0.1148075951651978	0.4844508558732772	0.4331117110359625
0.0674042095418113	0.6577510396464379	0.4002825384089438
0.1453063314390471	0.6085099492820026	0.3550453398146606
0.7113925459130662	0.4428064050843644	0.7447580562182765
0.2578696290023235	0.6857352841165339	0.2269444692963394
0.2467253682752622	0.6858512120117055	0.1514340498847290
0.1757896653202295	0.7233750974464048	0.1462411346725715
0.2255306292679103	0.5947287953883645	0.1903382518578662
0.3316850686757283	0.7393848580014299	0.0598265712658245
0.2791265619567357	0.7773662804396176	0.1869960224849347
0.3330846783519624	0.6523789567776621	0.2255280703479898
0.7435073868786328	0.4388922764992377	0.4589730930849005
0.7227645962146086	0.0828431885440169	0.7320464920062486
0.7730191295566188	0.5091706573736968	0.5304549544711923
0.7519747635608923	0.1523041110624775	0.8047047259862192
0.8912894129753113	0.4023574888706207	0.5701413750648499
0.9890243075431372	0.3149967369475059	0.6115290474903455
0.9017155080432095	0.6284129642286316	0.5489742251291182
0.1756103023448154	0.4422849193342853	0.4041581220774333
0.0916186679116818	0.7545668604151848	0.3414005703958374
0.7982945432905209	0.3048633124630631	0.7830886716929468
0.7044599724070911	0.5168726737741575	0.6738737337852048
0.6213657136017016	0.3787121112226379	0.8259120514934551
0.7428842086992652	0.4790563841086661	0.7625715483350530
0.7222652008337306	0.2259255872462848	0.5578243808962773

IWV-Al-T3-I4+

```
   Al     Si     O     C     H
   1.00000000000000
    11.6056394577026367    0.0835597589612007    8.7649669647216797
    -3.6825602054595947   11.3529796600341797   10.3282289505004883
    -0.2346490621566772    0.2412826269865036   19.0520343780517578
   Al   Si   O    C    H
     1    37    76    16    19
Direct
  0.8793068860071913  0.6667329568964566  0.7390119073108175
  0.1296995220943130  0.2273681848213606  0.0780760346311164
  0.1162065270984409  0.0904997875503045  0.0243743332989385
  0.0918542647152022  0.3169435170986883  0.2984418389239730
  0.2397160002418860  0.1327160048513688  0.5789493395855918
  0.0942228754452754  0.1103208819703985  0.7930027964840983
  0.3117443418545360  0.0922271947459071  0.3081896217055887
  0.0342385790103031  0.5543884846817794  0.1680231391946415
  0.9884787294241133  0.7626075283432142  0.1481763757171226
  0.2672128255864196  0.2857573241679314  0.1218067287633791
  0.5416137271532875  0.0457601820959520  0.2637017115918722
  0.7644324171593404  0.0017002193500747  0.1202292679694676
  0.2822657636089917  0.2683332675202112  0.3441647774689055
  0.8373155412304167  0.7531716618590155  0.9681310706371749
  0.8667665196308616  0.8889381756166440  0.0189190276163258
  0.7294720201128455  0.8553208139405060  0.4625939056244417
  0.8881589306951759  0.8757165930808168  0.2478194179046118
  0.6679238358643894  0.8944269794501215  0.7266361615736405
  0.9405790134484866  0.4293121875341055  0.8781886863970271
  0.9903467591692247  0.2186676128343470  0.8982463374574877
  0.6918243285299120  0.7035890934339604  0.9242816902105554
  0.4321350183909989  0.9357420998398042  0.7768096372115020
  0.2180665363114442  0.9830315725206925  0.9207605199747527
  0.6855642103472476  0.7123813740298429  0.6998368871185761
  0.8832336721598344  0.5066546618496918  0.1188381710473605
  0.4839277470417532  0.8987905452688286  0.5068501455336454
  0.0754472600567267  0.4727942279495417  0.9355576431690428
  0.4735639498200568  0.0831432731923588  0.5338405089575341
  0.9306794628992220  0.0559859513964487  0.5038306701732933
  0.8973350145628399  0.0822243397721654  0.3447151362112265
  0.0898534439352292  0.8958510200742549  0.5610754317960202
  0.1096461615033839  0.8644717481780883  0.7353136664631612
  0.4613505225619069  0.5053009870161761  0.9672583142580115
  0.3050934954654601  0.6668822107342397  0.9383853960425499
  0.5346265422006236  0.4493075454230766  0.1165029350738957
  0.7010141605064369  0.2789039267352724  0.1459078648218712
  0.7365851823191868  0.2457726038119939  0.3097490353332918
  0.2675112914316631  0.6964559891851906  0.7780421340419499
  0.1765565460813768  0.1709797879908966  0.0197360037186023
  0.0188974941475435  0.1249412635883466  0.2196189698842405
  0.2408045899427943  0.2682855246724380  0.0599677242328649
  0.0845372818585843  0.3387865376191250  0.0144561463842595
  0.7954471562626148 -0.0035437825908136  0.6267457992140810
  0.1709623591668980  0.1692810914988764  0.6568259423292901
  0.1836352225543459  0.9913937082120687  0.6665241492719489
  0.2216716855867565  0.2110933982185741  0.4769317834038087
  0.3824407022610620  0.1609001611502372  0.5158829861885061
  0.0174509706068564  0.7786787066765326  0.6170854932879490
  0.9848431784571349  0.6252237248057811  0.2123157548950306
```

0.1648924783979853	0.6378011329061171	0.0375164491658656
0.0431066843183826	0.4311796736297592	0.2661580163998989
0.9451520773824342	0.5293229950580860	0.1552769746231574
0.5631132422615319	0.5996869637403938	0.8237829396449857
0.6272369015045605	-0.0029432163910434	0.2002577315752000
0.6196113125976461	0.1627294169078171	0.2055432090811682
0.4441565156961343	0.0800882438194270	0.2389581482248779
0.4713765691698349	0.9438324938026760	0.4079500684692732
0.5800998328258963	0.5814216958570981	0.0573925385187160
0.8029508237851423	0.8127718026220211	0.0221904033198386
0.9715355632989310	0.8310183509662472	0.8411565716947065
0.7414240053094628	0.7583406332010724	0.9487259061242808
0.8319811968793128	0.6155492971945373	0.0617021867406615
0.2197438463633906	0.9580719761111730	0.4295697341466902
0.8113799352026956	0.8232601200724644	0.3813030645656259
0.8019417512676779	0.9772761047086282	0.4025674030340352
0.6952376622043850	0.7461353001503483	0.5933916861174643
0.6121137557910943	0.8738296715625760	0.4686669635104628
0.9755585111878763	0.1988335755585713	0.4208101768514585
0.0014907896834339	0.3558296304116454	0.8360171210810797
0.8266032288572960	0.3298062406900946	0.0199928090669269
0.9030698833648402	0.5281434063078441	0.7979977894861769
0.0370533709477585	0.4890131368804441	0.8637477678131102
0.4032123366636478	0.3765024679408779	0.2478069611565927
0.3530102678638746	0.9804445576602743	0.8458927469142253
0.3778333967961612	0.7929688771841999	0.8704712711682221
0.5684654671391063	0.9657717680716819	0.7271499996918673
0.4256964082773427	-0.0002501424228026	0.6696745028392302
0.3961034860417421	0.3881338164844259	0.0139956333260454
0.9779305354679791	0.4928428068255009	0.0241903070813316
0.4763650872176899	1.0000368173951553	0.5106359758171963
0.7704994543223919	0.3846947508893396	0.2413354958288576
0.0497946940656296	0.1625861223806841	0.9649727502263826
0.3767545157811519	0.7753355650777644	0.6363437905816979
0.1723248333572514	0.0483500991824169	0.8408062226959033
0.2068100670944410	0.5683047392657228	0.8382726264860491
0.9263994385911373	0.8081966841375186	0.0850657863830743
0.6062907817095678	0.1730558633477839	0.4411602041389618
0.8150873275458776	0.9430616663725742	0.1954044925285348
0.0160320466571998	0.9685282524024849	0.1638126613141063
0.9322535126259619	0.0346084255743097	0.4299488996274171
0.8394726887321323	0.1905137066305035	0.3235667374574633
0.9708960681972529	0.0093640940904019	0.8806411488507631
0.1197329880256026	0.8699320801164400	0.6431716419172699
0.1666145521646272	0.7646323635666353	0.7963163369788657
0.1268375401075015	0.8478669392085182	0.0461673254787707
0.3581847321775704	0.5656270645920959	1.0038059870762062
0.3204557264381276	0.6727314059827219	0.8430831575350880
0.8498157811658880	0.1392822922722959	0.9963143373704761
0.6301983270408235	0.3774538662411009	0.1303559813265718
0.7308488911743509	0.2347991139720697	0.2334922146057875
0.2208022109288997	0.0571120645896161	0.9514725934659032
0.0615095068845247	0.2158059465709387	0.7938523984363040
0.9145524792259052	0.7671010140356171	0.2516572431542305
0.7655226852688021	0.9259954332739606	0.0899898831049997
0.0209030099739310	-0.0015947076698556	0.5359466816924376
0.5217548016432447	0.4595090121369720	0.0317647248298039
0.1587454269584577	0.3239976867035820	0.1877984344314366
0.3160439450043978	0.1668271409288187	0.3378955856125517
0.7939857632925155	0.6775631595315299	0.8451311073827166
0.6697807820005555	0.8259665116975133	0.6890184173685100

```
  0.1874065436691115  0.3186786070354232  0.3166396365841103
  0.2729867003870710  0.1608798840592915  0.2221718472944300
  0.8015978612992124  0.6767519690566206  0.6906573003580931
  0.6354442894312923  0.7998424932074497  0.8582368019316415
  0.1281346248802642  0.7416738844949582  0.4136247275688034
  0.1121155296609013  0.6302937443493886  0.5157761133358005
  0.0384737083633837  0.7549818933410402  0.4005669743608239
  0.0097800275012769  0.5369046379493352  0.6038459908712902
  0.9361127121102227  0.6619889159436848  0.4870504028673676
  0.9107264581876374  0.5498179826531495  0.5987979460406676
  0.8275380473660748  0.4349658531819365  0.6629411188204667
  0.7227530377890933  0.4611224227216436  0.6597490648697949
  0.6151786492482995  0.4505872039631636  0.7496283884368967
  0.7340490049363078  0.5003698611991543  0.5630303860077275
  0.5216125586283142  0.4798798775831812  0.7425176437369662
  0.6409972902163447  0.5303362533030082  0.5552129557700498
  0.5344962346357901  0.5205929510803182  0.6449183172207185
  0.2384612643399576  0.8418745277519685  0.3240048256278137
  0.7890820806964796  0.3301696805143026  0.7896813303487996
  0.2598490355769601  0.9548365478861617  0.2081261899333416
  0.3433794647531492  1.0215485689251322  0.1498798589462843
  0.2669466442778633  0.9369554400578723  0.1585483943070406
  0.1869743899121096  0.9945282712299861  0.2261860034876542
  0.8651332867712905  0.2998315428175866  0.7876994166056198
  0.7491682302991135  0.3540952228710901  0.8423863871153602
  0.7223703706129869  0.2546071274044658  0.8365691859773292
  0.3157737100022214  0.8068131122908406  0.3056595118436257
  0.2322798293118037  0.8625121688399618  0.3717499730828437
  0.1825697372402616  0.6199948186051173  0.5241686299170838
  0.0512148267214508  0.8394353874341064  0.3210258085516722
 -0.0010906404428652  0.4532417165485227  0.6822757395129029
  0.8669162298061902  0.6726626811704666  0.4773710674842391
  0.8530251432456560  0.5734024500819017  0.6553088767334069
  0.6035153065786687  0.4195007497415116  0.8262291408135831
  0.8172708141833196  0.5087985233503434  0.4922845866765051
  0.4387785351258944  0.4710087002102141  0.8135222526795520
  0.6520889413611902  0.5615101926946070  0.4787571773428454
  0.4617054403866900  0.5445037445603882  0.6390189582820943
  0.8832114514764803  0.4128909322156262  0.6086421907992792
```

IWV-Al-T3-TS8

```
   Al     Si     O     C     H
   1.00000000000000
    11.6056394577026367    0.0835597589612007    8.7649669647216797
    -3.6825602054595947   11.3529796600341797   10.3282289505004883
    -0.2346490621566772    0.2412826269865036   19.0520343780517578
   Al   Si   O    C    H
     1    37    76    16    19
Direct
  0.8886156006744218  0.6785146884614296  0.7237740343628102
  0.1327029413497004  0.2365763367836852  0.0669702570207497
  0.1198385459290979  0.0964704605380496  0.0168214219387572
  0.0919845250564028  0.3231531431471557  0.2926103649791068
  0.2402359445092334  0.1397037917128597  0.5727646980067542
  0.0976567597585706  0.1158989154946125  0.7858141682714882
  0.3188492022866200  0.1050141256007803  0.2953407495063651
  0.0424380505250485  0.5643828899977852  0.1565682328007289
  0.9895969311597296  0.7687318974051972  0.1403954752467735
  0.2720839808341864  0.2963520658373558  0.1102225431694475
```

0.5461245476493236	0.0503716375388607	0.2605893037296086
0.7667110242941159	0.0048080777201544	0.1169349302706435
0.2791966781620817	0.2762896691552458	0.3399051411228966
0.8414001897656216	0.7595601834480634	0.9586778416456148
0.8695974029650964	0.8961505842203811	0.0099760736453696
0.7340096677443798	0.8589216638614920	0.4557474828341980
0.8905493036914431	0.8793531413047889	0.2429772327950774
0.6690703212009693	0.8985442439455742	0.7204271707183940
0.9359716523751171	0.4380605676598014	0.8637253826977792
0.9942276542677487	0.2245045371159909	0.8915829492277054
0.6967644179540725	0.7039131749281912	0.9172814380717370
0.4377945495134717	0.9477581302555326	0.7618335686443959
0.2194487689503028	0.9865078395125759	0.9147365253429357
0.6925568001528086	0.7238222579376468	0.6883814123792467
0.8935060800129221	0.5174967296911317	0.1049449806538406
0.4874158624344053	0.9019817346480662	0.5022147027353565
0.0736264464971075	0.4789503958566354	0.9237481883212537
0.4791114735752366	0.0969467034864739	0.5173892685216558
0.9340665856342921	0.0616699641410193	0.4956570273667206
0.9015980866183967	0.0875051088447729	0.3366076974497954
0.0989615039042166	0.9080420216004241	0.5492218029173239
0.1155090864188723	0.8731320024868749	0.7262744979017079
0.4696917917051728	0.5132434350287091	0.9571170255087296
0.3110679576277374	0.6750475056577477	0.9252107798287272
0.5309720661493731	0.4503214683648127	0.1169376078008859
0.7052462573711565	0.2874101143384219	0.1350190641003634
0.7490909932472678	0.2599745986006425	0.2896810640471215
0.2671305988082914	0.6970564655856438	0.7720589950620882
0.1813924398200760	0.1750103657373359	0.0132630010613186
0.0205091248252973	0.1366352512384190	0.2079891533554674
0.2413425781786483	0.2800284516902979	0.0502009499462469
0.0891883540791285	0.3470862864244311	0.9984382714262166
0.7979082221512287	0.9995421003999079	0.6187118506833535
0.1749921124435785	0.1793273079481263	0.6479644593270004
0.1837914669931248	0.9968647598951418	0.6674097911119961
0.2161128854843431	0.2128139119787803	0.4754136682447351
0.3843219199154927	0.1701403650093428	0.5027415880933450
0.0272772605217574	0.7860264767257240	0.6124914977531419
0.9860131536065679	0.6314247090736946	0.2047862486391049
0.1778955153129553	0.6473056564964158	0.0331452324582855
0.0415599531593164	0.4365227194593831	0.2593685804252334
0.9649868846917091	0.5491114588042105	0.1287494786158299
0.5827638038295841	0.5990727848056597	0.8162713732236303
0.6303157005191345	0.0050350081522610	0.1927454576993788
0.6271004103127072	0.1575364114436530	0.2131235786923169
0.4537242119448165	0.0950961155169724	0.2291947100243287
0.4716662376214210	0.9444655649594281	0.4056854987815914
0.5837506401937906	0.5836612343608826	0.0514863332651755
0.8069260081321157	0.8220530549748521	0.0104263788250629
0.9754668417992608	0.8357163344193055	0.8315726691703009
0.7460808654973499	0.7619212661678308	0.9395909815297065
0.8361152017560293	0.6225790798622510	0.0534238161869909
0.2261341872391291	0.9713775299205500	0.4158817468218459
0.8160857866854577	0.8225898191995661	0.3781942089613972
0.8092982028934066	0.9782052009328231	0.3968908237485410
0.6919239687164150	0.7480872449002435	0.5903505476156907
0.6190886551991033	0.8834764060079329	0.4573712401394145
0.9760456853088896	0.2045979195193851	0.4140071554931731
0.0003072013815786	0.3611304382962722	0.8273856490529253
0.8110467612290081	0.3439792248969749	0.0005388774880150
0.9097203845702857	0.5401443355590771	0.7726092508665818

0.0226230921647259	0.4918855290499720	0.8620892362313946
0.3985079628914115	0.3853752941615766	0.2460022709447198
0.3526211859599968	0.9798639352812987	0.8420632714265340
0.3845537678668365	0.8109893547516641	0.8356527992494615
0.5683286289647949	0.9683158656145594	0.7271339908313866
0.4448261230455615	0.0321563928224357	0.6440817528047453
0.3996136407493396	0.4021233773598107	-0.0005609699942449
0.9829723465871019	0.5007768086126675	0.0122474753363360
0.4744612243842787	1.0005234779054004	0.5106007029528801
0.7842474616890407	0.3939677986284261	0.2320898560768032
0.0543797562796476	0.1731930545385585	0.9569408014087337
0.3852842937517667	0.7760801759185231	0.6327601594256554
0.1736599311483166	0.0488658963505093	0.8350271852734904
0.2043713539266822	0.5784716486320817	0.8195406408611663
0.9315082173815001	0.8156828311746628	0.0734084772734285
0.6116235866468437	0.1916108027606552	0.4107034114513202
0.8124773372593405	0.9453650479671611	0.1959111808246864
0.0184172813816216	0.9740575043661944	0.1552910154754807
0.9394227417296306	0.0421503613896142	0.4191728484011606
0.8382130551200336	0.1914313264026426	0.3208194328365048
0.9715401148255833	0.0189005639295296	0.8717355302394156
0.1341719793254076	0.8903876098324769	0.6246402213960671
0.1730948489680957	0.7725626621455141	0.7853284137092389
0.1275792777800220	0.8525945367240025	0.0416808888417887
0.3768169719520507	0.5860762629730321	0.9812150880024413
0.3061947803996408	0.6562198092649900	0.8529181061055701
0.8546018892457412	0.1410607079576740	0.9922351350690573
0.6195790481135914	0.3714835279617288	0.1388422720033751
0.7647548953498982	0.2662228162733503	0.1940730531457789
0.2230279758137896	0.0641322389490585	0.9417415444225786
0.0665038909704348	0.2198813918629869	0.7884784144561093
0.9135436247283429	0.7727252018281509	0.2440217512472834
0.7685450505367764	0.9305688406888184	0.0846290691620982
0.0246367542674219	0.0062903316135628	0.5276046458205684
0.5165931882336882	0.4564775051022255	0.0348844978021907
0.1642812711038873	0.3308777054084073	0.1810415386913709
0.3166952565094077	0.1799941421698383	0.3268276872579142
0.7972077997801892	0.6736715506837991	0.8415945208361263
0.6627641118292081	0.8336169053838087	0.6825306374326543
0.1836822988670726	0.3275043015080384	0.3135758533698744
0.2866307059048817	0.1737674734840953	0.2040865644022082
0.8180576980405542	0.7083338969634030	0.6629729159065882
0.6414105621128500	0.7999600631067819	0.8498163277578908
0.7172116953832007	0.2784644837466451	0.8689038013862623
0.8356656007958735	0.2838942504499563	0.7935905532032098
0.6792753158990753	0.3786871459934866	0.8265793852208460
0.9173897160314906	0.3893742119208044	0.6772346420271007
0.7593642039069161	0.4861600800504881	0.7055239847505865
0.8813969059466843	0.4946372739416766	0.6277916234979422
0.9567223624199748	0.6000945501652810	0.4927794117998170
0.0845936915957851	0.6512998378730488	0.4328285380760163
0.1831432798806285	0.6350054340149430	0.3616269503035755
0.1054802782886603	0.7216739008949714	0.4454153594630450
0.2997090601652350	0.6896323504140511	0.3029398063290970
0.2216764015265635	0.7754786013119750	0.3873141592350523
0.3208655833727766	0.7612941201108683	0.3140365912511695
0.9469446969120970	0.5570892710407178	0.4442628808310078
0.4467693351495506	0.8269980251845332	0.2439556733629825
0.4895926484452524	0.9540714514228095	0.1241963747833141
0.8624465586339396	0.2031903742393271	0.8286119572201639
0.5883084264020495	0.3759313229655521	0.8833563782210754

```
  0.0094473048767485  0.3944088503324655  0.6195353086437239
  0.7307643660093515  0.5648970671189569  0.6662789831354249
  0.1700791422766177  0.5814207500300229  0.3493789424279830
  0.0300337531688905  0.7363062776465394  0.4997372802854604
  0.3756125612044865  0.6770898250455857  0.2466721310873093
  0.2352872063494388  0.8300478102178258  0.3984049374223088
  0.9131744011667305  0.6707017290136628  0.4776858365447396
  0.9797495877027740  0.4794696951841304  0.4661968566267485
  0.8536132848480615  0.5321559004752895  0.4820363258595703
  0.0007601697396887  0.6285585662276333  0.3466477058929486
  0.6551568084535804  0.1940521887930637  0.9613749457907272
  0.8601349149108206  0.5247110455795004  0.6835029419874818
  0.4333224908671386  0.0072519894347986  0.1394798140228099
  0.4851241280275622  0.9520391319264548  0.0696755674541194
  0.5828606490361495 -0.0015772263901313  0.0732670323718430
  0.4509063029935685  0.8319225291485237  0.2970404575122789
  0.5083625365587541  0.7779842092285236  0.2260812498916246
```

IWV-Al-T3-I2^{+}

```
   Al     Si     O     C     H
   1.00000000000000
    11.6056394577026367    0.0835597589612007    8.7649669647216797
    -3.6825602054595947   11.3529796600341797   10.3282289505004883
    -0.2346490621566772    0.2412826269865036   19.0520343780517578
   Al   Si   O    C    H
     1    37    76    16    19
Direct
  0.9038314107734373  0.6635053025359968  0.7297318933501027
  0.1486130872075683  0.2209369624633501  0.0758089820809842
  0.1347859665641920  0.0834489542135857  0.0225067192937830
  0.1060402747989011  0.3064957268910758  0.3013983923736072
  0.2568358990595144  0.1251353409265130  0.5780316984042752
  0.1131928396911420  0.1008323912938700  0.7925552091810255
  0.3342195510849751  0.0884914049161050  0.3046104534855951
  0.0561441092633782  0.5481396710468368  0.1658069791602820
  0.0058553795511131  0.7534698718255258  0.1484637607079232
  0.2859742051308302  0.2783035785094660  0.1209285096669725
  0.5613879768592296  0.0345105398579882  0.2684602095830420
  0.7822616132357005  0.9911526180187524  0.1222157045356640
  0.2966500883952742  0.2637224791847063  0.3443995651887242
  0.8561341803734354  0.7439178287467559  0.9670503082455141
  0.8847164753272133  0.8795799543076738  0.0188234632532054
  0.7500534210627035  0.8449767944868164  0.4619987966964670
  0.9084534452987190  0.8669996888058945  0.2474431617846311
  0.6840342290710079  0.8817952764054082  0.7279835177350218
  0.9544312595692165  0.4220499937806449  0.8727883557941953
  0.0077471501068962  0.2097646428797421  0.8973777450937713
  0.7135096045120922  0.6917762904900512  0.9221723507728246
  0.4527008001566230  0.9326545710308156  0.7705519373197655
  0.2358551792737883  0.9735790609284450  0.9208999533918956
  0.7066536613941917  0.7055081859501515  0.6962909417499981
  0.9068521894099730  0.5025000444697623  0.1137525505461462
  0.5039921626297728  0.8872458854025848  0.5089310972547751
  0.0909456169134413  0.4647250747873306  0.9325735462146163
  0.4942918245142842  0.0802325444424064  0.5267506312806892
  0.9499266176937949  0.0445543656585208  0.5048440806817402
  0.9184436222063185  0.0715702362595101  0.3446932319528515
  0.1137139631382070  0.8903056993285801  0.5576761403444004
  0.1308998253277830  0.8563225580599693  0.7342538817473117
```

0.4849447423610976	0.4978236599825522	0.9650644207684033
0.3267714427615875	0.6591295300024588	0.9349159139917415
0.5503491939139966	0.4367614650973272	0.1210276010707875
0.7216336213303098	0.2703197477812659	0.1439535953848406
0.7640607679225700	0.2424058619108863	0.3004201621555446
0.2855622026951643	0.6833344459283771	0.7790104408738802
0.1958225715101783	0.1644694307885530	0.0172413225356520
0.0380820756085569	0.1187947485706009	0.2169645226973529
0.2596642346052517	0.2626510513144149	0.0577194668706447
0.1041506478237310	0.3327112828790464	0.0114797211998694
0.8134305266861600	0.9816642303247837	0.6274430959810898
0.1909484500506823	0.1619024636556711	0.6555338896249412
0.2000346388226145	0.9832060503735032	0.6692136506082165
0.2347051031916381	0.2023426210754521	0.4788970468376798
0.4009605045743373	0.1542909763994847	0.5093416536731568
0.0421173959554915	0.7723652865198032	0.6151981441914498
0.0018645571395876	0.6158172416680719	0.2135035421042861
0.1919291679246510	0.6292313164540848	0.0426330428413541
0.0541613500392859	0.4192083944514347	0.2686960290540201
0.9769408001968944	0.5350365325483426	0.1382863882361564
0.5925777278644602	0.5832790674527042	0.8230117702181163
0.6452758113919982	0.9866488058955788	0.2033484090013580
0.6430363980342437	0.1416249996748088	0.2205365722875299
0.4693189136299414	0.0800247205801417	0.2368559010263101
0.4858063775750717	0.9295054695358268	0.4141208593366444
0.6031875153643708	0.5692940916775363	0.0568778373166345
0.8217477355618396	0.8052828899130062	0.0196886513168233
0.9922563895574426	0.8176018921045015	0.8426160326343441
0.7632868684291308	0.7501593415452767	0.9440286801219155
0.8470110246572846	0.6054050958907701	0.0637865212578053
0.2429877029873213	0.9549179737623799	0.4255797729704694
0.8317733753696062	0.8094383003580140	0.3833655652018100
0.8254535931241004	0.9635234231728865	0.4039819948843902
0.7060085071714424	0.7332274941149655	0.5967109253123851
0.6363055144005650	0.8699147771628032	0.4615992955994399
0.9908005061950037	0.1875927402618294	0.4243979394868110
0.0175091151690469	0.3463896977506132	0.8343657379192728
0.8319084585647796	0.3255653666925867	0.0111479126143152
0.9261958489368778	0.5252363118371657	0.7855874792381980
0.0444131032703536	0.4743709205806064	0.8696134608663494
0.4163398761679439	0.3733246023339908	0.2485452549713209
0.3696386008550593	0.9670584079333144	0.8490684565753703
0.3980076914717507	0.7941507149365504	0.8496347666897347
0.5854047421174760	0.9546169986483956	0.7312978521448897
0.4549151114233589	0.0131912505851641	0.6559328426714094
0.4123044370137621	0.3853461998611498	0.0107975104118636
0.9991635548429885	0.4895696171520854	0.0181708577147444
0.4916677189308377	0.9844228028378147	0.5186662280287258
0.7997345377823434	0.3773262531995245	0.2408291735278465
0.0674041870159737	0.1551408509874725	0.9642528553218752
0.4037659766090843	0.7595187557497449	0.6399987240210510
0.1917913611781915	0.0379635377732836	0.8397806623161710
0.2230313416576029	0.5627230060904168	0.8289945587237306
0.9440144878329280	0.7977554175968966	0.0853387045605938
0.6275854906422563	0.1731110736242396	0.4237445712948491
0.8327769083306668	0.9329663012057902	0.1977234722641334
0.0357796475625281	0.9606033532402999	0.1619684408800571
0.9561132027201596	0.0262004110308467	0.4272334205280522
0.8560380092087851	0.1762455904875200	0.3281297884677442
0.9894074082934511	-0.0001368510711670	0.8806116939584654
0.1442929898629836	0.8655128385746983	0.6390484898361151

0.1924040622599454	0.7594695451632932	0.7911735809449273
0.1431762885622482	0.8395380766808546	0.0472103536601727
0.3914486416339232	0.5692514718123814	0.9919222285263962
0.3261923911057659	0.6439802458195898	0.8583784587059129
0.8676076428733734	0.1281085498969141	0.9971678559086194
0.6373182215843800	0.3562906145216215	0.1447075296204309
0.7762418520864279	0.2463249824379417	0.2077798875824513
0.2394029088841706	0.0501035493360892	0.9491392529357141
0.0802851698560760	0.2052316007758853	0.7942693242224259
0.9303626736135083	0.7571205659645465	0.2527481544005022
0.7835603745547983	0.9148457761936540	0.0922037124441772
0.0412556051854504	0.9905987800761089	0.5358771238636626
0.5379907787385184	0.4446253343185366	0.0369999730821222
0.1762113106812173	0.3125074437898023	0.1909924444246357
0.3330188220117957	0.1658503887988269	0.3336151143603940
0.8148587131089281	0.6657843656940075	0.8429848127937434
0.6773948346916046	0.8151703954287252	0.6917389957676445
0.2003546127788782	0.3142802401805052	0.3185065194277811
0.2993589654110189	0.1559426706893096	0.2153964155581300
0.8301730070365162	0.6878002300311183	0.6735461960008816
0.6537051994210599	0.7854114468063951	0.8587392761681888
0.1965766652052887	0.6678707369553640	0.2613620746089547
0.1322287832263865	0.7020791918282538	0.3255952383834431
0.1534037473557459	0.5509036272565921	0.3199396121458623
0.0282482879884191	0.6222182030964140	0.4444820908961639
0.0491063593375929	0.4707498178681488	0.4386373076099769
0.9856462906778130	0.5053348892729070	0.5022065577035991
0.8710880236902374	0.4190572133578154	0.6311317193275185
0.7629457343151453	0.4576988648722392	0.6419510422980101
0.6629599055809871	0.4354629568344117	0.7425971598848905
0.7634713420171813	0.5237809873173901	0.5396284219844585
0.5631502229045575	0.4691392245006724	0.7483153441772923
0.6550167912978759	0.5525240703709843	0.5524084313513017
0.5578197140682359	0.5270149044077155	0.6542585365538468
0.3115078361755395	0.7523153112395826	0.1347285256579447
0.8833059318227595	0.3902158307483792	0.7192357206977722
0.4224828918135285	0.7336703721639681	0.1300957382831730
0.5051602434519410	0.7952821807172763	0.0369306023074056
0.4210713211320546	0.7486943428035282	0.1793774591382790
0.4254228262062468	0.6422102954775817	0.1707178358815667
0.9646857648539601	0.3637081966002046	0.7038090678481569
0.8914223092425546	0.4680330741875184	0.7063051584109674
0.8067577732630462	0.3162161889625868	0.8130226199865873
0.3113451537620191	0.8449263847689956	0.0917370337514788
0.3162687036501656	0.7391963943179868	0.0827976192549934
0.1644404278114562	0.7928081445189195	0.2816526686770300
0.2016299168135875	0.5220090147580529	0.2720482297397270
-0.0195479236727535	0.6506933219537070	0.4928966476050112
0.0169490882841370	0.3801318361286479	0.4822417105710138
0.8323247037932209	0.6127258523882391	0.4685845239755990
0.6611784515158711	0.3921774768334407	0.8179524674335812
0.8092226208373599	0.4935037507630681	0.4919418517530671
0.4864741878771650	0.4497294191890775	0.8291765879085659
0.6565189541015587	0.5995996930548753	0.4762565472399818
0.4779460321353627	0.5517696031600473	0.6635524506586992
0.8551438420953297	0.3350358726776068	0.6543951593969851

IWV-Al-T3-TS9

Al Si O C H

```
   1.00000000000000
     11.6056394577026367    0.0835597589612007    8.7649669647216797
     -3.6825602054595947   11.3529796600341797   10.3282289505004883
     -0.2346490621566772    0.2412826269865036   19.0520343780517578
   Al   Si   O    C    H
     1    37    76    16    19
Direct
  0.8715325978222918  0.6777742993078553  0.7432421483495788
  0.1160411227734881  0.2403737757719890  0.0816345793818798
  0.1047281094125131  0.1010730565494163  0.0289351332511249
  0.0775100649980708  0.3215838316878187  0.3079570320722141
  0.2250096859442351  0.1411895678812373  0.5874603715868444
  0.0860901710633020  0.1258466053789796  0.7937001234407353
  0.3025726391425273  0.1008653631785982  0.3106947355068115
  0.0258362434420033  0.5593815471464820  0.1721742391881327
  0.9832330435558423  0.7736738773739571  0.1466017386923851
  0.2557088216719534  0.2941468732670434  0.1270433675694385
  0.5349338988323771  0.0567986618397683  0.2641267492119346
  0.7551414881768703  0.0091341559754694  0.1255017653778407
  0.2687289034883277  0.2745874631313066  0.3513671208217308
  0.8243946785366125  0.7590961490543897  0.9745334252118876
  0.8605300924629443  0.8995438268379817  0.0197611000432655
  0.7211865550014418  0.8587016850400112  0.4679145055086873
  0.8805382813472041  0.8824418834121927  0.2520351742587504
  0.6533155267889181  0.8974016812817122  0.7344256631047033
  0.9310538227598815  0.4426730337488909  0.8814837186090833
  0.9777631609298927  0.2294728768666667  0.9033424101381341
  0.6780506319146206  0.7086935078467403  0.9325096700942194
  0.4195714678769266  0.9447949224828417  0.7837596996153626
  0.2109163008621726  0.9994609240608209  0.9196607942175244
  0.6790796668737553  0.7199112698309048  0.7030019470496305
  0.8710226922665298  0.5149858911246303  0.1243790257011570
  0.4776455258972924  0.9082913010181048  0.5090261487730381
  0.0656848355255307  0.4877689493887619  0.9363468548029711
  0.4613361074881578  0.0896033901396325  0.5414387655516824
  0.9197805016870596  0.0599714416824653  0.5096481777211472
  0.8847498224019840  0.0880192202674984  0.3509727040758062
  0.0847658876901213  0.9061195896745511  0.5649166704493843
  0.0996696263638928  0.8751558644548393  0.7405764511990152
  0.4576879741949635  0.5113929077448187  0.9692156856975933
  0.3003571292423328  0.6770008084371474  0.9390815830419700
  0.5210661683732550  0.4540021574235767  0.1256034367607440
  0.6913296898205193  0.2875514464929108  0.1514687806627938
  0.7218743379460918  0.2506157562229905  0.3188643322492036
  0.2616722980661977  0.7103038517995324  0.7785109410705012
  0.1633069630771525  0.1845364984824644  0.0225198626186921
  0.0043724601292795  0.1379052163478859  0.2221871521326810
  0.2264175458388104  0.2794404940664829  0.0652537314037425
  0.0716169730383288  0.3525776301345036  0.0178450594634088
  0.7831594564035949  0.9976819712268913  0.6329474724091322
  0.1611760462537653  0.1883885015034031  0.6561545182953750
  0.1584884129699500  0.9998363698400700  0.6868279327653040
  0.2081769342485447  0.2151259492799417  0.4850888463532573
  0.3673578381578314  0.1638452644380533  0.5255492325551328
  0.0119002703988856  0.7824515560342348  0.6323718301613726
  0.9855806367418053  0.6379299954012525  0.2077350310822483
  0.1578664606745867  0.6312290714944521  0.0441669741911860
  0.0262107120507950  0.4347081024676074  0.2775080009764812
  0.9333427965304694  0.5357551753339793  0.1618071721148407
  0.5669819303853005  0.5965787181838645  0.8278888391308770
  0.6170746847807185  1.0023530474477731  0.2058144897109505
```

0.6190722058321194	0.1759953159423213	0.1975379069328319
0.4363374062575611	0.0911940933702435	0.2410457098907512
0.4666394075604274	0.9592224777557332	0.4076413068179178
0.5654683957168097	0.5870513900317688	0.0656556994469959
0.7970833209907263	0.8288126590343594	0.0180122074022847
0.9580028000846738	0.8286098714671354	0.8477490864912565
0.7257522776134917	0.7644458808359753	0.9577575467787596
0.8142033031356066	0.6219804806773155	0.0746947718805054
0.2109775044812895	0.9672783941418138	0.4323264312380596
0.8032462556110471	0.8253325935379435	0.3875198673528639
0.7934346101618069	0.9804662021438727	0.4083238278530195
0.6845413805600723	0.7497695498230995	0.5999048471164194
0.6041633392231990	0.8792883644939730	0.4727775884379004
0.9609552786817747	0.2029157450541562	0.4300064091939283
0.9874491541017145	0.3649341252449167	0.8440699243627778
0.8214400875640944	0.3469989556549821	0.0256175339667597
0.8882435747736820	0.5364944619252561	0.8050385563485806
0.0318608900533107	0.5059877659192938	0.8606763757920886
0.3882233999900123	0.3841462137719089	0.2559055348055926
0.3465440477922798	0.9975720719477337	0.8455806431148577
0.3561893557616532	0.8025726500690992	0.8845633087115894
0.5559573490734121	0.9717098696830188	0.7352636445712273
0.4133042403128052	0.0054257461380734	0.6774194963609212
0.3841877642411182	0.3966472978720267	0.0191586001606193
0.9661349075502245	0.5091424206493688	0.0236579239065843
0.4704683062281129	1.0071906857468707	0.5159238516161271
0.7612133557723305	0.3906725545082060	0.2453081370482108
0.0377571555264272	0.1697142421239883	0.9698135287882149
0.3679484623599176	0.7860619966960241	0.6369808287488717
0.1686303548849882	0.0689232803537778	0.8365356049304998
0.1967786891094563	0.5807665680791065	0.8442625399432553
0.9174163653693826	0.8149165215361280	0.0876471940440339
0.5912246771677634	0.1838355730621526	0.4491629670107288
0.8064448132347711	0.9496445064994607	0.2001852210490895
0.0066099771612892	0.9777450108457809	0.1681032580383088
0.9234718933608995	0.0415914539208557	0.4332430700592692
0.8211870570248199	0.1915195746252042	0.3371863510102749
0.9659325895820640	0.0203887571868649	0.8824952167951838
0.1222357017701718	0.8946557095253467	0.6364456327769555
0.1622482106206601	0.7799787291518803	0.7966733979740827
0.1198717121873687	0.8635474024332304	0.0415437404881991
0.3636640000037651	0.5833015981866794	0.9947213284538614
0.3211140896328016	0.6923238839351264	0.8367360804834583
0.8375225963571645	0.1481341850628035	0.0020546976774342
0.6148619262872360	0.3812689463409828	0.1428832685412149
0.7120578111078623	0.2345315645355668	0.2475865710653156
0.2111184311365839	0.0687901290044096	0.9554819892557420
0.0477178093746874	0.2286977117812999	0.7974855566303682
0.9103105734327369	0.7772551024109307	0.2505210722577434
0.7595108356514816	0.9366744173322092	0.0908050886812528
0.0101481328877214	0.0046296880138491	0.5415636847471194
0.5098228516632084	0.4612246646329782	0.0416732887099515
0.1476453963335778	0.3305836424181582	0.1960029281176030
0.3044539754237194	0.1769276293341007	0.3403122472552414
0.7807120565815000	0.6823049129796526	0.8545631143440133
0.6451890938176432	0.8275117167768108	0.7019019920952095
0.1708017158385208	0.3247450739229852	0.3279992056123278
0.2636953387327357	0.1689010596374254	0.2247806916953143
0.8026520792045562	0.7053831574404804	0.6819593652640861
0.6227225599803627	0.8056205059763861	0.8651320411264295
0.7811427212431112	0.1372342102061128	0.7164939132179394

```
  0.8093368683297059  0.2587637875840760  0.6237193989073743
  0.7650112479795723  0.0956822152274625  0.8161388995489983
  0.8274431522228389  0.3403745912611754  0.6275179950227423
  0.7808043402168054  0.1747031954398276  0.8223688590149907
  0.8119024169855766  0.2967231899658912  0.7296896946999916
  0.8485290645531922  0.3868656709894249  0.7228846746014486
  0.9544750962822675  0.4892965867024696  0.5928354759481811
  0.0435976257019290  0.4589284501326387  0.5281384159664383
  0.9590822969883211  0.6085038651995162  0.5285723606377315
  0.1334617592585872  0.5483782352282733  0.4022911304054649
  0.0479125080543425  0.6951658141302141  0.4037413671157750
  0.1346647938765841  0.6672496201912208  0.3378899478445485
  0.7415876184669622  0.4253376921926339  0.7760422397123612
  0.2258708786142286  0.7651913587249549  0.2031618372339814
  0.2962708743965928  0.7234534857609759  0.1376367951615124
  0.2355997003137240  0.6673472583472517  0.1569384740151021
  0.3519516704805298  0.6718495531854457  0.1628407120407347
  0.3563824682456959  0.8003557118798903  0.0403932142579649
  0.1787882536775873  0.8240997638087446  0.1686873532869099
  0.2885393436842424  0.8209458670103915  0.1826791497034145
  0.8181091245197866  0.2923876175891049  0.5474609917863286
  0.7427215557246450  0.0000865968150534  0.8890057758001042
  0.8203283917839906  0.4296647971816646  0.5705559446932538
  0.7730473714982672  0.1405738267343795  0.8982998981193554
  0.9478043810249468  0.3979780258418166  0.5556703058716741
  0.0585003906750987  0.3721638562694026  0.5774839860322791
  0.8926869377409659  0.6353134666978015  0.5743027030895096
  0.2015478137995766  0.5226662473454295  0.3562123878817524
  0.0480182778196762  0.7879492844557966  0.3551850386785673
  0.8790776638472840  0.3422482270370794  0.7774372899530874
  0.7084120977083561  0.4725068889825588  0.7237310532387740
  0.6679500915512571  0.3466224093134924  0.8685925803073584
  0.7683384583767292  0.4842646046701037  0.7771050351089732
  0.7701637942508879  0.0745882093480253  0.7126283627792211
```

IWV-Al-T3-I3^{+}

```
   Al     Si     O     C     H
   1.00000000000000
    11.6056394577026367    0.0835597589612007    8.7649669647216797
    -3.6825602054595947   11.3529796600341797   10.3282289505004883
    -0.2346490621566772    0.2412826269865036   19.0520343780517578
   Al   Si   O    C    H
     1    37    76    16    19
Direct
  0.9270575263407285  0.6860403167510697  0.6993957043769261
  0.1685455837588574  0.2434805863795157  0.0418336537669187
  0.1505725612893864  0.1008556222688118 -0.0042812883898970
  0.1267290570294432  0.3278374925174746  0.2693126209137680
  0.2723904547269442  0.1487222957906875  0.5496377348201735
  0.1279367955469178  0.1188698744932176  0.7668162963771457
  0.3556440157580472  0.1132665610469111  0.2735638604314956
  0.0811538048357119  0.5709931579732721  0.1337159589829268
  1.0193974568005617  0.7709809125248800  0.1232455102604357
  0.3072902293948051  0.3023552098828222  0.0870657110154305
  0.5814973109220207  0.0559969469566396  0.2418511926769674
  0.7966918605018641  0.0087785498414772  0.0962785072325076
  0.3129038327292920  0.2849541055722351  0.3171064043334642
  0.8759043214124467  0.7665275029055630  0.9348080322370755
  0.8981060016517084  0.8972686070353736 -0.0062127785215726
```

0.7674842231449920	0.8639466657311706	0.4351897523943937
0.9233601426646972	0.8836311028161409	0.2215108841438798
0.7020817153885341	0.9023157657660347	0.6965177237540932
0.9707021408067136	0.4435060006187709	0.8420757014245818
1.0222331960205993	0.2277520446705634	0.8724935341393858
0.7342934715708249	0.7100542403149586	0.8923313084403616
0.4711556698878734	0.9555783078300418	0.7385100113180556
0.2513093108300679	0.9912676523300590	0.8930168374025645
0.7286395000121221	0.7280459112849298	0.6656436275051720
0.9383028713451030	0.5281863081276429	0.0754019230249110
0.5229333007121580	0.9151335026640078	0.4807575166438348
0.1153849628101326	0.4892965810075088	0.8940755910359390
0.5152601672853286	0.1137605996204013	0.4916576652657201
0.9699393734335454	0.0655314975863820	0.4703303792867080
0.9343986285889558	0.0960826391787822	0.3119060255810540
0.1358000772577062	0.9121288433912702	0.5251587041127203
0.1468163470305162	0.8823869280444333	0.7025517386336857
0.5068693888375657	0.5188683911331156	0.9310534229102664
0.3466500193655103	0.6825783529866376	0.9026289124101887
0.5642944676370379	0.4546767091198811	0.0932985617221036
0.7406805437670471	0.2931557221148797	0.1142004867453320
0.7835885032169245	0.2720879478297342	0.2676430124253723
0.2964107018476601	0.7076624370372609	0.7522964395783907
0.2141212668041914	0.1791065203172458	-0.0089757684978438
0.0467410017820322	0.1516345150745765	0.1786700111965488
0.2736675991060329	0.2741683876905228	0.0359698112593135
0.1408144433371724	0.3622156134892002	-0.0382213180140540
0.8311308195030911	0.0058473136922120	0.5890983652564716
0.2014949869803701	0.1837298362144680	0.6290069372481183
0.2195816833368428	1.0064189469931073	0.6396889539409484
0.2480428967322168	0.2252913179303083	0.4519831798726309
0.4159915176366376	0.1814476940499475	0.4809053161343579
0.0694159057594993	0.7888431813920256	0.5866538490079863
1.0189384854570283	0.6336891968369903	0.1869006486796106
0.2197551039807409	0.6509128850083167	0.0190965802785840
0.0703086990538246	0.4379666140890218	0.2407689916382376
1.0140438670337231	0.5662163005271020	0.0922844737798397
0.6249442815487143	0.5996924037147168	0.7932612211274549
0.6608487538598811	0.0045922218023287	0.1793447721215155
0.6685110629221426	0.1602583893030459	0.1929474245801875
0.4914533430981981	0.1058175091185490	0.2079087308542833
0.5038162222071556	0.9533549019123700	0.3877157505009485
0.6200235185831797	0.5891751322641195	0.0232672549254919
0.8385042042738312	0.8236129510816461	-0.0080109583725935
1.0064168355978957	0.8515559421602472	0.8033796324849081
0.7762459701906044	0.7639142748302935	0.9222792756077203
0.8808857779207651	0.6320603890141042	0.0222606593189739
0.2630086346867175	0.9795086853894283	0.3910401354136326
0.8470142061299289	0.8290614553996596	0.3558335335208270
0.8446993327836775	0.9830063306250969	0.3751301981587543
0.7294794592072410	0.7518386832959213	0.5672941982486228
0.6516136788432046	0.8898841617481359	0.4387254789965936
1.0124537324154459	0.2077405537819623	0.3928084507652634
0.0317801428103559	0.3654103909555925	0.8058985331655507
0.8486489434996864	0.3490773892656338	-0.0193585136545468
0.9438062948929551	0.5462232030614134	0.7530260959877841
0.0618594541561501	0.4966784671404130	0.8370066477974594
0.4327534012263487	0.3935499499129520	0.2224854502601262
0.3833940815246267	0.9808703735216213	0.8246284113768262
0.4154227293463841	0.8224642660075457	0.8054761889624886
0.5999652258072644	0.9704582031093834	0.7083662678419089

0.4813939420696111	0.0482998016888152	0.6183839080676140
0.4299103408077014	0.4135036375133673	-0.0280343045203289
1.0236179990285186	0.5047309498294598	-0.0119973671591826
0.5197641004756033	1.0201549380904749	0.4792950357165493
0.8298266482644300	0.4054134206280905	0.2068685653969774
0.0825485457484652	0.1753531799134552	0.9383253481026448
0.4182474744728759	0.7939369969116349	0.6148131170451138
0.2104571480914050	0.0571193938021530	0.8088978540753868
0.2420697691155616	0.5953623985380773	0.7864868056952823
0.9546720045476811	0.8141325429724187	0.0630550943114181
0.6443146732387196	0.2150616082572000	0.3846088641870139
0.8501615371007136	0.9488484621490055	0.1691819686829136
0.0516976814875256	0.9773686215981345	0.1345771535252570
0.9829924878683917	0.0544088463007061	0.3856071808830610
0.8643794891966660	0.1934823605676588	0.3069760283503311
1.0043376387229137	0.0173644374804215	0.8557869546359805
0.1679569510392019	0.8960619881988421	0.6019424306444796
0.1982987121834704	0.7792574158753296	0.7687477643362350
0.1562384750849866	0.8587182283181011	0.0199190575804951
0.4226273930569420	0.6009263944050571	0.9469756914926174
0.3250257504991873	0.6562766715036089	0.8431872013719339
0.8825610658970955	0.1459146229647926	0.9716934085850524
0.6509687569749658	0.3739807096072765	0.1175952661084405
0.7976598088941258	0.2783783109271611	0.1729197714176478
0.2544285025073518	0.0695195155016666	0.9196697320980461
0.0952340049555513	0.2226603701340866	0.7694909629737161
0.9473419920142897	0.7724969346916567	0.2285468994752640
0.7964948368145727	0.9347427344275613	0.0644350576313187
0.0556602597561210	0.0057977345592683	0.5077342346518312
0.5444104713540914	0.4548424894065961	0.0174040762775666
0.1972767336844994	0.3365830424905694	0.1577619660551759
0.3475796813432127	0.1839573146676168	0.3110213346621035
0.8404071534922347	0.6833242103460825	0.8160376667624800
0.6881293280225047	0.8349411954271455	0.6640239863505517
0.2185293887266576	0.3360570067513544	0.2885388238365706
0.3259862577998543	0.1852839799540729	0.1803615230469006
0.8560193194874489	0.7262837327558972	0.6368376925372921
0.6822137596936917	0.8079118204072533	0.8219370876045252
0.0051483912464217	0.5544207500532901	0.5094064424464748
0.0222164147270025	0.4408328671849598	0.5729592756239559
-0.0559441473694507	0.5698812124749518	0.4692129632045768
-0.0164232161950372	0.3468986339990998	0.5938777161972861
0.9007139556984761	0.4754040883878471	0.4885687361600782
-0.0755077625526028	0.3604883297086849	0.5521284881473003
0.8858787016443449	0.2656694439167810	0.5664073606316636
0.7680684049604756	0.2642464714603095	0.5859882772617848
0.6616090651913897	0.2324842507296661	0.6909569447870771
0.7643999718587617	0.2950119740005284	0.4999152687287904
0.5533324771915862	0.2316525601262439	0.7093639723992289
0.6561238272071166	0.2945561534303489	0.5179728815407494
0.5506615925709825	0.2630893393813502	0.6225475911904681
0.0510751586635523	0.6523472431756384	0.4876161773128099
0.8863461080828088	0.1412018790484970	0.6588893448110009
0.1510826145114398	0.7583182307283959	0.3541966192817735
0.1803813028392923	0.8279437387394901	0.3427933598187971
0.2291160924722071	0.7313703795633065	0.3173368498207095
0.1194737677105369	0.7974016297473917	0.3011744615813440
0.9767038613956103	0.1408192757089887	0.6335183238020671
0.8253132206316768	0.1097103521518669	0.7484732628778195
0.8560294107958987	0.0780528051286827	0.6640638729115972
0.0822755612760011	0.6162402253752245	0.5403225088833414

```
 -0.0238908040053043  0.6833750128056238  0.5218959071092546
  0.0672978445267756  0.4250842768765620  0.6089434201681256
 -0.0757042292747957  0.6535801905808357  0.4229722018801310
  0.0015728156188841  0.2631939986257480  0.6429898231023854
  0.9289570470848802  0.5108474138829656  0.4026406478275921
  0.8019791686896137  0.4564174449228343  0.5374879673388999
  0.6632786640200730  0.2072923677522479  0.7592096124796777
  0.8466979675286971  0.3184878978002448  0.4188681864358612
  0.4706802080211345  0.2053488447182385  0.7918136051656088
  0.6546461213967704  0.3183312851437622  0.4502686416642265
  0.4660576249903449  0.2623167780066485  0.6369834036706659
 -0.0448303329176916  0.2951882146339386  0.4775442897209865
```

IWV-Al-T3-TS10

```
   Al     Si     O     C     H
   1.00000000000000
    11.6056394577026367    0.0835597589612007    8.7649669647216797
    -3.6825602054595947   11.3529796600341797   10.3282289505004883
    -0.2346490621566772    0.2412826269865036   19.0520343780517578
   Al   Si   O    C    H
     1    37    76    16    19
Direct
  0.8811119698646661  0.6697266761246270  0.7382459207642194
  0.1319680537872877  0.2272438390077677  0.0812832391265768
  0.1195866952605087  0.0930565176114007  0.0254149796164105
  0.0925878846927436  0.3189867201644771  0.3028335599865470
  0.2414155028712906  0.1339353341694140  0.5806538873655334
  0.0936253588089922  0.1039350328878346  0.8014172770362145
  0.3135383804093183  0.0935384276357783  0.3146383266813569
  0.0391262443340613  0.5595457374124297  0.1700941765509037
 -0.0138168346261546  0.7613685471424317  0.1577982541038006
  0.2657975996821682  0.2835553518862399  0.1303105636920090
  0.5426526377837472  0.0461221928390025  0.2715613143852927
  0.7649766885897015  1.0004565934311045  0.1277632033974244
  0.2840033518765678  0.2734872203553363  0.3466040660158767
  0.8421258286903406  0.7558086281380365  0.9706781017492048
  0.8641481658699863  0.8861460460268695  0.0285427876158650
  0.7300621880685634  0.8591789919531684  0.4665387020230525
  0.8905826023581650  0.8770665805472594  0.2533565173108123
  0.6695823321205229  0.8954478969675917  0.7307291271860830
  0.9400604414598589  0.4309104834227444  0.8757221091768508
 -0.0070333268134101  0.2183918999820365  0.9011131675362607
  0.6986702277771417  0.7038709700974672  0.9258755983452324
  0.4359093541711174  0.9378844235522479  0.7776544213290689
  0.2172078755548227  0.9786913594792916  0.9282859020964771
  0.6811088071929139  0.7137942668880773  0.7063056504578588
  0.8924178559162962  0.5111281315530741  0.1170734028013440
  0.4853232305169131  0.8998573029374937  0.5123324069006976
  0.0769845174804197  0.4727144981874161  0.9357247762353931
  0.4779781766085379  0.0898818428751936  0.5326113624716253
  0.9341332589291628  0.0592775281869238  0.5059835459525868
  0.8997925537586419  0.0856111621821405  0.3475335812575259
  0.0894234710712598  0.8964454729912185  0.5651713027057931
  0.1106064840879174  0.8647036756090736  0.7387418624928318
  0.4604492516315053  0.5054385820574274  0.9737672443193693
  0.3059269264118978  0.6670941973662555  0.9421126147298772
  0.5366876615395828  0.4515920856475503  0.1202409861586173
  0.7048521840365850  0.2822588624412447  0.1460944494051660
  0.7449978215191504  0.2536635570463357  0.3050417080363444
```

0.2660379759240823	0.6938155363880614	0.7839971782037584
0.1810506796207444	0.1697645829895442	0.0235507690101364
0.0166237430877417	0.1264288448166840	0.2214196063234561
0.2397320386562286	0.2647754642096624	0.0690197741067446
0.0929791987307594	0.3414551032066874	0.0115144415010009
0.7975561002438645	0.9977621643852583	0.6278790928960736
0.1687018862806117	0.1615582079403128	0.6660643596493132
0.1915450230054956	0.9946846284047295	0.6579095604417484
0.2223050902173860	0.2185078633569880	0.4789584417487452
0.3843091567525376	0.1645799183300375	0.5164504940323764
0.0163861121378928	0.7854373224170828	0.6134993440429253
-0.0177232147644152	0.6243867604570612	0.2208449426603829
0.1716177793724982	0.6478922623655442	0.0427915538308796
0.0453634665757808	0.4342317336627201	0.2687125883375388
0.9586169889401644	0.5385691249215854	0.1475816458413133
0.5600487820797635	0.5995022529436501	0.8299721244302616
0.6289054403181596	0.9991176177041605	0.2064231780510022
0.6216371068098475	0.1578497222185087	0.2198461583444176
0.4476860592348152	0.0851814059275369	0.2434468067495040
0.4681181732445540	0.9427937470034787	0.4168038170926306
0.5861706615879942	0.5831344570286021	0.0600021249287394
0.8012570217527646	0.8088416252581846	0.0319783041694552
0.9770573712257642	0.8388951836362585	0.8452516811089552
0.7499765958731651	0.7609830472993495	0.9475255576865638
0.8406788479408448	0.6189162412903116	0.0594429373401784
0.2233440962031368	0.9591155792859150	0.4368484153715522
0.8185581466392056	0.8286328111826314	0.3846833959413605
0.8010742619277399	0.9778387838336769	0.4124199455170552
0.6882779868027913	0.7474432741342321	0.6005843326911533
0.6168953672779772	0.8806669867141641	0.4658238797970435
0.9758965199497013	0.2013913884375894	0.4269960178595487
0.0014834628374676	0.3559240092774830	0.8357952898368626
0.8192556376557154	0.3338083276200183	0.0152596811682578
0.9117103817708061	0.5345790147516520	0.7878574398165459
0.0303751385345311	0.4835425783104249	0.8719536764217626
0.4044244622830822	0.3822108206087919	0.2501617026157021
0.3498027481371717	0.9694761424227261	0.8583036021908628
0.3880530415432100	0.7984113275986311	0.8550840558735826
0.5687830288734904	0.9654874566729519	0.7359082181654888
0.4358810336614160	0.0164896577106196	0.6644124482481001
0.3939755206906979	0.3867336601041201	0.0232737335799645
0.9840767977238503	0.4928115063930303	0.0256260029282415
0.4777990935146811	0.9995721994066945	0.5179095804200544
0.7791249360166711	0.3902828170569428	0.2421764315964603
0.0516825334395981	0.1677261492531744	0.9681231969385503
0.3827811033815746	0.7747018378370004	0.6439408058400795
0.1713156325871583	0.0378426241563233	0.8491782668543252
0.2071282051429304	0.5732851955713022	0.8316562657268826
0.9263621942541986	0.8082934651819130	0.0924634537094086
0.6110119596058872	0.1826543966274804	0.4319323391587609
0.8151853321306101	0.9428556760536783	0.2033182202257949
0.0207378016636186	0.9695807655190920	0.1641626036226974
0.9408572721737967	0.0420722692679167	0.4272392245006109
0.8421773237726961	0.1936052173273423	0.3267140526013229
0.9668136573690954	0.0071416037723067	0.8898358813509951
0.1083185849743727	0.8586430166936113	0.6584623436371221
0.1691896944135125	0.7664693276825061	0.7987863012068793
0.1241987231931222	0.8460512949756124	0.0582439837091223
0.3585870215339007	0.5673866901955994	0.0093738634098443
0.3052738433797146	0.6547093944314623	0.8640338699748745
0.8531603124560431	0.1360818372773729	1.0005795789002767

```
0.6281973213091123  0.3753736639081168  0.1380356140662503
0.7521157563468120  0.2519220292642358  0.2175817365249337
0.2231102568765839  0.0615010619122701  0.9496813533864125
0.0666845546621955  0.2112547443636753  0.7995342893182382
0.9115071000722001  0.7653152291207910  0.2617019838120764
0.7622823150152370  0.9214065281312690  0.1016984466824913
0.0241012678200477  0.0022506993966769  0.5385185804693913
0.5235429063148325  0.4624659444594770  0.0353258372164921
0.1569744463361427  0.3223795449299466  0.1950014020551620
0.3174601842964318  0.1707712274605575  0.3422155231127522
0.7988956782058325  0.6749001450314724  0.8488401777528682
0.6650185215635893  0.8279074578200979  0.6946160593608319
0.1896602363762158  0.3233274467625135  0.3183456007738050
0.2726548265965108  0.1594196941576226  0.2302964895648940
0.7985139718888643  0.6811476306973613  0.6941007491970117
0.6423435882420248  0.8001400204995359  0.8598592385517040
0.1331459060254809  0.7363234978712441  0.3823464206038950
0.1378693373768168  0.6180322856523534  0.4355009042279315
1.0217092684367699  0.7550690903188422  0.4255438848173801
1.0357155885196818  0.5218273698766092  0.5295832624359037
0.9157763763032415  0.6593964579594747  0.5188607264327895
0.9192418786603701  0.5384148327031564  0.5756093993543373
0.8059927733126030  0.4340296981261818  0.6703538740867120
0.7010133244999515  0.4723517630373675  0.6691864700928312
0.5890341575188570  0.4392042763567117  0.7712149192471394
0.7159708001232281  0.5417269568189722  0.5629004608555239
0.4941454200062860  0.4744765807158968  0.7673590771017668
0.6219300763128507  0.5778491857194782  0.5585036913916583
0.5101762089191674  0.5442690839052015  0.6611184912696867
0.2457235738227144  0.8400961838596271  0.2779324292689159
0.7775452123532168  0.3532078683021783  0.7929042584461636
0.2826521330676494  0.8911967878731799  0.1589487662646601
0.3577590224008891 -0.0259384162499878  0.0826735790121650
0.3105742013804041  0.8275363365699090  0.1484131636397329
0.2074818899348330  0.9110502535607624  0.1558438490286423
0.8558564838082401  0.3265186001525912  0.7877796007047756
0.7537492961776311  0.4009024603723079  0.8220429257406631
0.7026484304087912  0.2724753854990293  0.8610189005608967
0.3185493783037634  0.8117595454592679  0.2842941107765102
0.2298379504171892  0.9096164309054707  0.2805475528250170
0.2255112649222675  0.6036627514235783  0.4002845870272359
1.0150715329880042  0.8445958047289248  0.3867685484303767
1.0415211175773309  0.4322254420026060  0.5678818918275743
0.8282712782023622  0.6730670123320174  0.5510556137756852
0.9101563974292488  0.5770419519489717  0.6200274964602583
0.5740406972596269  0.3856378396123582  0.8551592606241979
0.8023871585756639  0.5697127866106114  0.4816568125983655
0.4077626244578793  0.4472148962077372  0.8481112470733727
0.6356963338102737  0.6326827564546089  0.4746869520578029
0.4361580390293653  0.5727610657728435  0.6582867978684105
0.8326179458362776  0.3838131169455658  0.6435648962412228
```

IWV-Al-T3-TS11

```
Al      Si      O      C      H
1.00000000000000
 11.6056394577026367    0.0835597589612007    8.7649669647216797
 -3.6825602054595947   11.3529796600341797   10.3282289505004883
 -0.2346490621566772    0.241282269865036   19.0520343780517578
Al   Si   O    C    H
  1    37    76    16    19
```

```
Selective dynamics
Direct
  0.8574952901539613  0.7029939977331603  0.7236540533633278
  0.1040212828511553  0.2648182808365624  0.0621530977892591
  0.0906812280098592  0.1261949965575782  1.0093827759446696
  0.0655425075710044  0.3482811429335890  0.2868050002563882
  0.2128038524700012  0.1666243296385533  0.5676667252123793
  0.0702077318089015  0.1512003775554970  0.7748249078059090
  0.2888792055915423  0.1261243108297344  0.2913652024278190
  0.0121797223183099  0.5853836275173728  0.1521493116429202
  0.9702848110163868  0.8001445622823120  0.1258939096448113
  0.2433109672918505  0.3211891350637388  0.1061015479786138
  0.5214889047163517  0.0829849866775184  0.2432881186120160
  0.7411471273770396  0.0344018385287095  0.1060561850458451
  0.2561813806754647  0.2987850529105838  0.3321492777482498
  0.8117371391422670  0.7851121539272317  0.9542247126156542
  0.8467766156673120  0.9262888563984700  0.9989014191922976
  0.7077498029660593  0.8847124499968875  0.4475863623872720
  0.8656796967487590  0.9075402399346593  0.2329129539445053
  0.6403675583185426  0.9253173143704010  0.7141896637466649
  0.9188002135128597  0.4683519040314592  0.8618579228295682
  0.9640394832471196  0.2553867811467331  0.8834645152271425
  0.6648498725180673  0.7344974646581575  0.9124910218234240
  0.4059624169075240  0.9711959649649552  0.7633580041652257
  0.1958142201297342  1.0241204206526877  0.9010635151330046
  0.6659901554423707  0.7480626596015767  0.6823093567399753
  0.8586592425910193  0.5399851255664275  0.1039799262609661
  0.4648691043326167  0.9349923603224487  0.4877277009763761
  0.0544339910579672  0.5125001460751079  0.9163045517786472
  0.4491782076249659  0.1155882655260101  0.5205062889554750
  0.9055170343204197  0.0864977009005985  0.4900605828930075
  0.8713746121361944  0.1135763693498118  0.3311545627217016
  0.0704200966776713  0.9318433204621119  0.5451225190867015
  0.0865572779415796  0.9010287071353635  0.7201566456085662
  0.4433946624609224  0.5389216337218751  0.9491362913630150
  0.2860224567887014  0.7038047502346051  0.9191217311944794
  0.5082331824543735  0.4799906818035738  0.1051222355913669
  0.6781262615073368  0.3131758930968361  0.1311754155757590
  0.7085433605399138  0.2761891055568134  0.2984850084868602
  0.2487000786069744  0.7361945662028483  0.7582107364977170
  0.1498220321215608  0.2077380421828876  1.0045056844731075
 -0.0074244049797214  0.1635632495025835  0.2031218451224125
  0.2147547923725980  0.3046174899885797  0.0449974552102728
  0.0603315065673723  0.3772966761430728  0.9973402135876328
  0.7690860842133167  0.0275518029848987  0.6129365754789332
  0.1484096500433647  0.2126552779125231  0.6372377844012245
  0.1480143846930634  0.0251329691898834  0.6655364906081371
  0.1953155102657885  0.2408719104420269  0.4654606187648200
  0.3553179383474062  0.1902993992742135  0.5051050425291033
 -0.0022380066175261  0.8081700618631602  0.6126049409497839
  0.9692744464529957  0.6633369073779866  0.1885242043140286
  0.1431551033660887  0.6595143520094190  0.0228319573624893
  0.0158092369774010  0.4622549713895688  0.2559058917525035
  0.9187307292718295  0.5582680064490868  0.1444949501652080
  0.5521124319697938  0.6262553937087183  0.8076204238220808
  0.6029752421367315  0.0281222478968592  0.1854961765048171
  0.6056899322541235  0.2026831359909452  0.1760307352083827
  0.4222815440744803  0.1164881263472723  0.2211006650310192
  0.4533685708350930  0.9855567514634730  0.3867977912198380
  0.5513112719081575  0.6131353871012357  0.0458507262504758
  0.7830638908339431  0.8520666129184560  1.0003876042961659
```

0.9450618723704978	0.8568023771323520	0.8267083823603403
0.7125726613765828	0.7888519009675097	0.9386204700067504
0.8038786509850346	0.6480968563908523	0.0524352531793406
0.1963895252010785	0.9929053634878342	0.4123884347648138
0.7890024024598372	0.8487549000739505	0.3691913769941889
0.7809899445146281	1.0072905747268668	0.3860071470408187
0.6701884936392798	0.7771770713118943	0.5800406735357619
0.5911523699270423	0.9053816964739528	0.4516655416564826
0.9485762074605462	0.2298672847061386	0.4084015659073673
0.9743225736943839	0.3908826727947859	0.8243039388723635
0.8093757866244843	0.3727110600073442	1.0062819550641375
0.8760092077142960	0.5624750858280166	0.7856820247164502
0.0203907747686054	0.5313657331370182	0.8405521862372802
0.3756726272911430	0.4087667438142248	0.2362183699875326
0.3321196229230279	1.0270231016548190	0.8215555187622712
0.3423438296581762	0.8287596597228917	0.8653626679739710
0.5416882138558120	0.9983521350687954	0.7165625602879182
0.4018005659702402	0.0293113616380924	0.6564777028253832
0.3712997697093618	0.4244206772611678	0.9976798419824024
0.9548499648613280	0.5331413371761349	1.0043058033731285
0.4587828696207261	0.0348916016429968	0.4931524206252860
0.7475958042217943	0.4165262891344919	0.2243080651826440
0.0251884075690914	0.1967822334683670	0.9489237289955277
0.3550095707593149	0.8129967949544286	0.6163636118519147
0.1479256290330015	0.0899959006264507	0.8229160162870706
0.1854637175772622	0.6058091739796547	0.8241748497777746
0.9089745841173194	0.8449928210314406	0.0621379939646145
0.5787451973776480	0.2098410534953819	0.4293065661851860
0.7901095198854603	0.9745864744536604	0.1829373279066149
-0.0081694498295439	1.0028562599508677	0.1481711066757740
0.9068332284874078	0.0659894668204591	0.4160497156369705
0.8084190513930454	0.2178153116108460	0.3160772192328094
0.9485876147592521	0.0489445750218301	0.8606144677100769
0.1083041623234844	0.9208890074611945	0.6161456061494683
0.1476366923480447	0.8041596336094536	0.7771757147329674
0.1077675699472311	0.8870159617860879	0.0240243598050067
0.3476925846660014	0.6089655217408591	0.9765868273055454
0.3079772531027863	0.7196618802850471	0.8160657166416907
0.8243504819116854	0.1729793165232505	0.9831123666559233
0.6027000505746716	0.4080025888204136	0.1213198563194977
0.6965148868164475	0.2586977434095447	0.2291695015599687
0.1973778358588821	0.0944419676181687	0.9354577753365777
0.0339882840299329	0.2550646134312970	0.7772562620834357
0.8940127981156428	0.8020415661460802	0.2314017743892935
0.7455917234599391	0.9600725595243853	0.0732816462647038
-0.0043176278368866	0.0303734911924988	0.5220495302096719
0.4956014977243597	0.4878417629535117	0.0216903753974765
0.1350404511822812	0.3582229299880191	0.1743711306197288
0.2917897766627031	0.2002895272969860	0.3224972825422263
0.7666210501929377	0.7072511836231510	0.8352091629840254
0.6342608572784373	0.8571989116841604	0.6793239574332585
0.1585623041151764	0.3489534265317080	0.3081766118796936
0.2504314737839072	0.1965535538735459	0.2050217146716213
0.7887685101565807	0.7315647657346050	0.6621969293106293
0.6098875763017056	0.8319884000792191	0.8449511360416520
0.7355526119169732	0.3684643599477935	0.5257266306344015
0.7458559803536534	0.4736753059076416	0.4989701897785259
0.7661432083683019	0.2749710174242435	0.5967116875186474
0.7911135861534266	0.4888034014173385	0.5393878558715697
0.8102263461763305	0.2877987464281228	0.6380859617809208
0.8251019716262817	0.3945230841636659	0.6096501350402832

```
0.9034999245065132  0.4272957133555771  0.6214625701812988
0.0145649909973145  0.5219152569770815  0.4868630766868592
0.0935320545785766  0.4840324465948892  0.4280262916596356
0.0490422832641613  0.6424986585654443  0.4267835413157390
0.2020803354696998  0.5638503303079673  0.3138957944300620
0.1574552787569327  0.7213050278844403  0.3124555051349603
0.2372232388609397  0.6828275130620426  0.2553759164607433
0.8439061803392285  0.4702826639551132  0.6860821814017166
0.3573369267551671  0.7671913476138444  0.1347172880372252
0.3469505194673540  0.8556653585449697  0.0335792572010288
0.2914468567303511  0.9103671929397890  0.0465159974689806
0.3058086451423401  0.8081930127606014  0.0307112509762858
0.4359887524278441  0.9153125933202777  0.9464836029024172
0.3992077314815798  0.8170572633145315  0.1353326809216582
0.4159700415920100  0.7160089127356797  0.1180463502144778
0.7190536133405440  0.5455325428508273  0.4473056626871003
0.7572584673475781  0.1925901420298915  0.6173827123539898
0.7932847783673630  0.5690584214027953  0.5236446348777157
0.8366944151040900  0.2164318561740124  0.6907002677351047
0.9141209125518799  0.5007858276367188  0.4870747625827789
0.0695556008796571  0.3909857070202972  0.4734817747290044
0.9917270678772367  0.6727984268159667  0.4718289511926363
0.2632469009871000  0.5332288491548199  0.2703771921102918
0.1834641235214235  0.8136535291526861  0.2678779825850222
0.9310013414039395  0.3498510835737463  0.6679418946192912
0.8122719394973656  0.5462015085591800  0.6444568916914114
0.7659365620015642  0.3975448844440738  0.7794034535237109
0.9057669910714796  0.4978973971346283  0.6872136924855580
0.7029829765947078  0.3573655257144110  0.4926576589001517
```

IWV-Al-T3-TS12

```
   Al     Si     O     C     H
   1.00000000000000
    11.6056394577026367    0.0835597589612007    8.7649669647216797
    -3.6825602054595947   11.3529796600341797   10.3282289505004883
    -0.2346490621566772    0.2412826269865036   19.0520343780517578
   Al   Si   O    C    H
     1    37    76    16    19
Direct
  0.9067427235888269  0.6527039610991451  0.7464662435521110
  0.1561118717112879  0.2152874351484221  0.0807532425961076
  0.1381485455445828  0.0746705476268355  0.0321777665604899
  0.1174825423537560  0.2987766790584513  0.3042609272680740
  0.2624609512845747  0.1159523083167657  0.5875595155913809
  0.1212816205891401  0.0970023331659558  0.7969879639462768
  0.3388462469854597  0.0760984460102548  0.3108590280856012
  0.0613404143215349  0.5354139477723123  0.1716089837948271
  0.0184452424306537  0.7475018093322087  0.1484997996278771
  0.2953754184725786  0.2718983380732911  0.1236344928410198
  0.5718283575590501  0.0332310722223287  0.2629273276040505
  0.7905253618717198  0.9822544647666394  0.1275394105837464
  0.3063489797731742  0.2486564073375219  0.3518850062386686
  0.8614486758465427  0.7353342164629155  0.9738597412983956
  0.8965787711470544  0.8749016103488075  0.0199624163296275
  0.7586500447555187  0.8349615731010783  0.4673107731843962
  0.9141713031446200  0.8556097629363303  0.2549140458505059
  0.6905164298459968  0.8774731287256780  0.7339396758800123
  0.9679590904685386  0.4171140596331449  0.8815223244434481
  0.0138904528722878  0.2034577085058025  0.9051725429509030
```

0.7145956899040836	0.6847496546735917	0.9335074921946477
0.4563422400853266	0.9217352674843109	0.7843157158696620
0.2438153048394904	0.9692593084871219	0.9252261422295662
0.7138148127319328	0.7010134424112423	0.7032628613183731
0.9102032333911635	0.4898922579269818	0.1213457319405042
0.5148632347961396	0.8854287379237434	0.5089389602459010
0.1056315787531333	0.4620944901329059	0.9337414030631699
0.4986982250407269	0.0666707344972241	0.5415664846746728
0.9550715865724115	0.0368952749662773	0.5097450010902611
0.9222762971637872	0.0642557211025282	0.3500594424170890
0.1198590663451807	0.8816751850336846	0.5643430232578922
0.1353114851488170	0.8507337331884435	0.7396998318253138
0.4912030710988351	0.4901795769996420	0.9696463906543860
0.3339684066884143	0.6545145813177408	0.9388522813435783
0.5579874022758933	0.4303348228329543	0.1249222771253058
0.7282716262610943	0.2634893642697434	0.1498890784841900
0.7579984314482702	0.2260116898482418	0.3174888905756121
0.2969576150865175	0.6856250122955780	0.7778987185669137
0.2004090104235825	0.1546605337224668	0.0267232171725772
0.0460587093242829	0.1161636661652185	0.2223470724341256
0.2689648251089098	0.2571105740974094	0.0602343245867150
0.1129983244917543	0.3273622040696703	0.0143380418210355
0.8197883805817717	0.9776898877279768	0.6329874894903089
0.1983251669108793	0.1602481128305805	0.6588407303413232
0.1991062502443079	0.9740946814043034	0.6845412016670563
0.2448612082740814	0.1899299600293052	0.4855834715937470
0.4052857728576829	0.1406861097697165	0.5240590440328572
0.0481038284858401	0.7578308005632993	0.6307632852420666
0.0143498499347658	0.6100407568356910	0.2119171297213945
0.1916662565662397	0.6151161785422702	0.0408461207663130
0.0687845019278477	0.4132674674277481	0.2722476607041345
0.9702234696481250	0.5069578724010926	0.1625006917358881
0.5990959305794712	0.5791161215302058	0.8273189172935779
0.6523984821166621	0.9767048397559838	0.2066574912016041
0.6567785371151817	0.1550373493799501	0.1914339842996823
0.4716190934892801	0.0643051104561215	0.2422170940476396
0.5056289592007416	0.9386470987962744	0.4058837922901150
0.6017100571866172	0.5629012485141321	0.0657959997296745
0.8332489641533399	0.7999461968580129	0.0220318723769615
0.9939342557024542	0.8086207255003128	0.8449937737637458
0.7602390049970880	0.7392082676925354	0.9606740671630466
0.8562476498626601	0.5985330015649738	0.0694230357326031
0.2456223125559973	0.9428888425400613	0.4312383031290117
0.8368670972371546	0.7963556269042674	0.3913687518820075
0.8344760304061406	0.9588583013018006	0.4012564740743314
0.7231206725505183	0.7290564556335568	0.5991954841006187
0.6400098142168742	0.8534957419286026	0.4747993013427942
0.0008978605618549	0.1804815111391201	0.4248124866877783
0.0213577566524483	0.3395260547854109	0.8440105501356849
0.8609648249226145	0.3217724979137289	0.0265631226298577
0.9224834599176983	0.5112593936575777	0.8080517365819757
0.0718367252232518	0.4802989218433335	0.8580321366389476
0.4266571678521631	0.3571158379381267	0.2566905198311007
0.3804588442746784	0.9705015944247221	0.8496446486165345
0.3964854093473126	0.7793072810664331	0.8808354744706739
0.5919359843265362	0.9500786728423458	0.7368116433720059
0.4507336338658915	0.9850286613196607	0.6769481754023583
0.4242103636979636	0.3752514426432410	0.0145694922370784
0.0067357614285073	0.4831497167690347	0.0213993769365945
0.5076994147961396	0.9818907813353704	0.5184103007485908
0.7986483797930271	0.3669485586796217	0.2405342630842873

0.0702063591220147	0.1479201834901048	0.9748124593250981
0.4044341739860032	0.7629294238660661	0.6359450271464516
0.1984320600621264	0.0309266561319911	0.8452683544571427
0.2371700889265741	0.5556475305745890	0.8405905336225985
0.9602687312137076	0.7949594044531382	0.0816624222978758
0.6289556565921038	0.1609236787393447	0.4483633378143992
0.8387241322511487	0.9236488361505828	0.2048348665425199
0.0401402387386127	0.9506730627902232	0.1708803170594883
0.9541330093863208	0.0140997255158697	0.4379693446897891
0.8582060445600742	0.1678244129198521	0.3344508121658487
0.9972868751835227	0.9982393348160780	0.8820638088419457
0.1576808317016832	0.8696354248535814	0.6358309339086335
0.1947523915080012	0.7519267010041109	0.7979263353277588
0.1562262942862765	0.8323529880926750	0.0495813256625411
0.3922995817200888	0.5565312643666600	0.9991726540604808
0.3546264811339862	0.6674288813421860	0.8376164168503970
0.8745389086765670	0.1203247835413352	0.0031852740158406
0.6542000448963745	0.3593535557975392	0.1379451103232682
0.7429582618973928	0.2062715868545410	0.2508685479688147
0.2427566812233422	0.0443052543603584	0.9560000031324087
0.0894102677862760	0.2000273594751445	0.8009533832766852
0.9412826898973813	0.7504652958865792	0.2531966471424170
0.7943188763808355	0.9065320413171567	0.0963803720768909
0.0454636565793821	0.9799532458330175	0.5414033855904549
0.5444563286623094	0.4395547004333396	0.0409997720055176
0.1874747277837966	0.3092003012633602	0.1912087132542207
0.3417099914594885	0.1496718549728027	0.3423113745298486
0.8196915291508808	0.6584023143558608	0.8565142243190490
0.6829281566377522	0.8108373182416021	0.6979013396266267
0.2105723251254817	0.2991090770659055	0.3255186828706926
0.3031489340722947	0.1474665815242283	0.2220950806871901
0.8339768375108441	0.6805085343473056	0.6883582404888842
0.6603794880535609	0.7808344358011486	0.8645553782498432
0.6285674691920772	0.5350629068048489	0.5832350286634985
0.5766962566569843	0.5004555232508771	0.6917253192973518
0.7302771682686771	0.5048399875601849	0.5327603050061450
0.6255136865981759	0.4352852748046837	0.7509664911087204
0.7809087518208233	0.4410733378831819	0.5900582550400977
0.7326472656159470	0.4084440365602655	0.6990943639806373
0.7836745327076390	0.3257072116460222	0.7670798445134263
0.9122040213939676	0.4023874615034146	0.6796919658662559
0.0141844930199006	0.3759067204137679	0.6262046324867574
0.9230417779372253	0.5195564880413343	0.6337837044359369
0.1256290751932625	0.4644221785477485	0.5291456601027948
0.0354653772227067	0.6079097471228233	0.5353789371302587
0.1383560372202169	0.5818008636463640	0.4810039291601206
0.7151428044476118	0.2850056272372635	0.8891907019351954
0.2612300574115023	0.6731719398994960	0.3757391617795842
0.2652600929878093	0.7983403231076963	0.3285875400553475
0.2323094267348084	0.7984368770291558	0.3974931070191705
0.2103920263805746	0.8360553932992408	0.2982342922330771
0.3577810964415376	0.8581429513097588	0.2519919444856108
0.3195820847967069	0.6390209927053916	0.4022678014412136
0.2998569352999004	0.6768662983478032	0.3031376930462124
0.4970507598210574	0.5236339930952452	0.7306833195483545
0.7707782477361338	0.5321668715396871	0.4481853370234683
0.5832079419237575	0.4068494682077649	0.8363368027588861
0.8626275784408061	0.4205499463830960	0.5497922742827044
0.8244782897795893	0.5059300761456601	0.6268881217978004
0.0077650445120259	0.2874580390661205	0.6576722345749934
0.8519966039556310	0.5380438366587155	0.6869965297736320

```
  0.2051234678277485  0.4419826029592510  0.4882784009425751
  0.0411818181592626  0.6953092888909672  0.5060181884617572
  0.7831858100441575  0.2472602611140444  0.7790344919522225
  0.7148787906194872  0.3618900420314511  0.8796510775889483
  0.6219268049226899  0.2312835648344993  0.9512040711250147
  0.7594113632071849  0.2309143057088958  0.9291449206050559
  0.5895845597087408  0.5864723729975393  0.5373914341593024
```

IWV-Al-T3-TS13

```
   Al     Si     O     C     H
   1.00000000000000
    11.6056394577026367    0.0835597589612007    8.7649669647216797
    -3.6825602054595947   11.3529796600341797   10.3282289505004883
    -0.2346490621566772    0.241282626986503б   19.0520343780517578
   Al   Si   O    C    H
     1    37    76    16    19
Direct
  0.8901739329628061  0.6829676229025097  0.7206388822256753
  0.1339238940819827  0.2410542173058147  0.0604477745563495
  0.1184146529136609  0.0984703970897053  0.0138583333178574
  0.0929371825384823  0.3268519175888966  0.2871506449153042
  0.2409390700470683  0.1423846375771579  0.5694752242039913
  0.0977458774867016  0.1181560304128494  0.7815424716570109
  0.3197668349619270  0.1095350405118973  0.2883218295794203
  0.0434561391191579  0.5685454691610395  0.1504988016026415
  0.9882568075880376  0.7706528243397137  0.1368580699656925
  0.2745873414432636  0.3022854471232189  0.1021369443337467
  0.5467948136697028  0.0529798758061146  0.2558461962362455
  0.7653863910424332  0.0064260224950724  0.1132243658581044
  0.2784888609657220  0.2779993145738383  0.3364951821279296
  0.8429033153162711  0.7647425272232053  0.9513051774160495
  0.8693711090088122  0.8993071650446490  0.0050271102289446
  0.7348896281755211  0.8620400357186703  0.4510655228443806
  0.8895850137622690  0.8807557730199873  0.2393589279430253
  0.6702281057654884  0.9022896621131419  0.7147810023117509
  0.9380793764822086  0.4414681680727106  0.8572559300535848
  0.9926719784888166  0.2250762189671626  0.8896860434942548
  0.6989602885256462  0.7021483091074924  0.9145600716905183
  0.4390606114277567  0.9516745451263233  0.7575959036392589
  0.2181517174379164  0.9884102712137584  0.9108540800666187
  0.6933837652727683  0.7323157143232067  0.6820040573810031
  0.8962138315432839  0.5221300326024325  0.0970034876995203
  0.4874193491056506  0.9062553943894912  0.4989091612331749
  0.0752300323434593  0.4827578411070518  0.9164818489331299
  0.4812534945196619  0.1021985271509337  0.5123907384454851
  0.9330633263083082  0.0663867899219155  0.4900255240011055
  0.9016529889291214  0.0922586209221834  0.3303340401570340
  0.1003666947595518  0.9147240246015906  0.5432566447885968
  0.1164555206810040  0.8793378782125453  0.7199057304047076
  0.4688533532555866  0.5197115510345880  0.9513511687152748
  0.3101002864888477  0.6807613849623990  0.9190671100583511
  0.5285018865416772  0.4525567549825274  0.1140728673195712
  0.7053310382673140  0.2921996127277388  0.1280034438450490
  0.7505751899970696  0.2657869363606391  0.2809818533472201
  0.2653958472458933  0.7004918976334862  0.7674979124330730
  0.1815064576905764  0.1764370177545999  0.0098741224539758
  0.0221818564000992  0.1429146723780787  0.2016958353872882
  0.2426502680133293  0.2867644558348856  0.0422132241956116
  0.0904343224875391  0.3506278460396197  0.9900847085607305
```

0.7972426180907615	0.0057502289624475	0.6129860450791210
0.1743374204566640	0.1840837862264540	0.6429103351725732
0.1853971014648627	0.9987140067710673	0.6668032167047964
0.2169210800681287	0.2117192510048136	0.4725097758547631
0.3846515813900713	0.1737605202492439	0.5003505264952024
0.0282613905370902	0.7882183445134177	0.6142183796137465
0.9834853117887271	0.6333985749863591	0.2007050520080347
0.1778226628753903	0.6551000143971507	0.0265282540995158
0.0448903842336649	0.4415904327996542	0.2518229470695004
0.9681257001602633	0.5510501739738047	0.1219559494571060
0.5826302065178640	0.6088763934829330	0.8107842162356478
0.6282594353770053	0.0038726055893022	0.1913188383683670
0.6321166876513414	0.1620642578865511	0.2025225001019402
0.4535203797680102	0.0964063645806636	0.2256061354623282
0.4733072209294411	0.9509327313261892	0.4007087145945541
0.5830034937932916	0.5851709034911764	0.0489530073528844
0.8073494830797329	0.8259177130488862	0.0044192979158351
0.9752261606551699	0.8435412967297083	0.8220555553680811
0.7458995020580435	0.7642211493279882	0.9350455179512969
0.8413410033503567	0.6290082218524987	0.0439296157886496
0.2242587342192233	0.9769068101474839	0.4087712475714723
0.8140099711317036	0.8253530656321424	0.3738801570254462
0.8111559031378194	0.9837456641057041	0.3880853991702681
0.6979905976775813	0.7525988916636079	0.5836207403596252
0.6175265230664327	0.8848983310046784	0.4568834943008847
0.9757937564603042	0.2094213730854656	0.4073566748828753
0.9993624629936332	0.3627834128962112	0.8235137553869376
0.8098555261636263	0.3551594120345802	0.9906283588154985
0.9163370387698399	0.5454296615363953	0.7596575466741684
0.0243383141879685	0.4979720427702021	0.8533150627249966
0.3965129159310637	0.3880477051427549	0.2432692517849291
0.3514865688491409	0.9817358802319984	0.8391960958041647
0.3847581815326123	0.8174231811504206	0.8261382807490739
0.5656304273683195	0.9666600644320168	0.7315344202532200
0.4539966813727116	0.0416176131393308	0.6350497464985119
0.4007256239063036	0.4099440941591010	0.9906279420821570
0.9842180030380763	0.5037839467301277	0.0053741180471201
0.4741863801579680	0.0035252499112985	0.5088826903912187
0.7854322626728147	0.3997821282700078	0.2234520385488425
0.0518820615697064	0.1766559652752014	0.9553010948873903
0.3834719410595364	0.7810877018318964	0.6283849705620537
0.1741904631723748	0.0500811209963739	0.8296258718429194
0.2057621951068898	0.5820743670865353	0.8126220253018723
0.9303331538630808	0.8188713294621865	0.0693381774018561
0.6129631220319842	0.1986915244157426	0.4008512984199557
0.8125334677905977	0.9465859475735526	0.1909882249971467
0.0172970245633867	0.9757715833783986	0.1519928686823113
0.9372375170226520	0.0456025093406144	0.4148393202103293
0.8385932325955050	0.1966414945334760	0.3134892833618250
0.9714790678381844	0.0218986448872036	0.8666740837558320
0.1404938118999352	0.9047259548725671	0.6121516151756231
0.1687334988636111	0.7736448209094066	0.7820563709274985
0.1261699330073887	0.8547127083603140	0.0379165726731960
0.3748903794240893	0.5915198829288865	0.9762681853898129
0.3025786596603866	0.6589849301468989	0.8498009912135074
0.8528546986642311	0.1432972368074942	0.9898101152781331
0.6156331013079333	0.3716482958789448	0.1368180039408893
0.7671097030989105	0.2720222197610265	0.1848722491487701
0.2204634560075486	0.0672360622577503	0.9372247402717915
0.0669138871721671	0.2203841976259807	0.7867578606591747
0.9129711906605967	0.7738779558373863	0.2408467307737461

```
  0.7680464036558848  0.9341929907397858  0.0788309945266940
  0.0229730114269974  0.0100153203947717  0.5227056608786915
  0.5134862474062128  0.4607192166498033  0.0313986171449269
  0.1663530531440058  0.3339485385121254  0.1759075283109762
  0.3177193430044947  0.1843946015964995  0.3198274140585669
  0.7997960150876555  0.6640788130702797  0.8470258681371118
  0.6624667756939646  0.8450960528651569  0.6708965545989974
  0.1818569833368998  0.3284948214210651  0.3118599798850366
  0.2916436123178044  0.1800342794477018  0.1938541451228769
  0.8169897079416820  0.7185201900780933  0.6616608890667187
  0.6493800963315597  0.7976148890157498  0.8404319891112180
  0.7139169605497373  0.2506578772016605  0.8685837669253311
  0.8375717352295269  0.2694777633963137  0.7944754844989926
  0.6679933484456531  0.3468285814533010  0.8445420680743171
  0.9165918697622475  0.3838888991948058  0.6981264969629103
  0.7460948000806999  0.4617826337707054  0.7441473980222297
  0.8741351962756196  0.4857055380292336  0.6702086286652909
  0.9455123769810386  0.6050079386286366  0.5352249368244271
  0.0790392221688940  0.6458957349503388  0.4658585588010817
  0.1593319084828941  0.6013819620350477  0.4165847797082023
  0.1264520712307193  0.7368657823311971  0.4432539477207654
  0.2829370003401472  0.6478041464481461  0.3457055696863340
  0.2496115173011899  0.7825479831500828  0.3732418582110388
  0.3301672837195491  0.7396977994646757  0.3220922185724593
  0.9159420323163964  0.5898161814340188  0.4782369314050032
  0.4634903196382473  0.7963448945890933  0.2388984679150564
  0.5055235858910128  0.9221708304044749  0.1183930747172155
  0.8707739269732893  0.1934008324145988  0.8139331136372210
  0.5718344410110016  0.3318555875272262  0.9017142012885500
  0.0129580969279639  0.3991020486956655  0.6417855547011729
  0.7111891610548341  0.5372777369814263  0.7202947624122220
  0.1259541546837409  0.5317914576835249  0.4307027906041972
  0.0661541528601582  0.7737125454475590  0.4796785140978498
  0.3440025767207838  0.6130561754706082  0.3067739714192770
  0.2832530011033144  0.8535261650909810  0.3573586474205018
  0.9085546159827377  0.6731175902846865  0.5358045782995144
  0.9411269470888187  0.5145048698379040  0.4863100229170413
  0.8195727293587221  0.5735699868085002  0.5219921971988386
  0.9651806612901216  0.6705918956943839  0.3827233678463744
  0.6526240196834464  0.1597309765688118  0.9456071573942757
  0.8939168922778878  0.5104733399986839  0.7123945944538659
  0.4622384947296455  0.9810855519606883  0.1322297112693203
  0.4824101023152449  0.9196328785013509  0.0742126938044079
  0.6031885125920302  0.9601986552725178  0.0586049971329572
  0.4874280535390109  0.8025092098741695  0.2807595198112829
  0.5115994950311792  0.7401584577568253  0.2229660239231225
```

IWV-Al-T3-H+I$_{dyaril}$

```
   Al     Si     O     C     H
   1.00000000000000
    11.6056394577026367    0.0835597589612007    8.7649669647216797
    -3.6825602054595947   11.3529796600341797   10.3282289505004883
    -0.2346490621566772    0.241282626986503 6   19.0520343780517578
   Al   Si   O    C    H
     1    37    76    16    19
Direct
  0.8977509294185734  0.6582042787081069  0.7426672480190112
  0.1465628828180308  0.2153227108244972  0.0804395278138115
  0.1391584903847537  0.0851397849906538  0.0180973315501304
```

0.1043610825952905	0.3080538444454594	0.3009470280012351
0.2564095333149946	0.1223173468078163	0.5766895152534867
0.1135201290810569	0.0989592266166018	0.7926063496007376
0.3277735037231913	0.0804586553853189	0.3117618057470236
0.0505657615955791	0.5494633575028256	0.1673504149447915
0.0069021474918449	0.7549417423256231	0.1479455686840619
0.2784315761921272	0.2697585972259846	0.1302545407626295
0.5583900725274358	0.0337323717836305	0.2679540959250294
0.7852501543515363	0.9935694038027880	0.1211243019091952
0.2962324883444858	0.2603176295294254	0.3444910608646657
0.8545100598177017	0.7445422518300747	0.9703830844085855
0.8851044569971450	0.8791630650977598	0.0205649463315764
0.7505149151439950	0.8464312163858946	0.4612664675630387
0.9094154584656432	0.8698202418000924	0.2462496215548127
0.6860155098168867	0.8822605929286828	0.7304798939143350
0.9524262598078241	0.4164687675303966	0.8774530666242688
0.0130702323027507	0.2114247326020885	0.8924693019097999
0.7055539503524807	0.6936952250827341	0.9289799486478906
0.4510581406594067	0.9277931402555329	0.7710226761900911
0.2376514831199810	0.9727945468266577	0.9199007181519204
0.6900048491696400	0.6978829095530793	0.7066545061900260
0.8997205567439206	0.4991451342815833	0.1199107666243623
0.5016869432452469	0.8813926531077471	0.5100040710680087
0.0857632067190413	0.4580680391029585	0.9416068780393303
0.4918610240946384	0.0740148666014300	0.5277686012215175
0.9470779062314068	0.0511345385792412	0.5043757232940075
0.9153609269012666	0.0736504355384993	0.3449398298461123
0.1029465367793160	0.8908905253299242	0.5605466304092313
0.1250062256131358	0.8523812237377479	0.7367878221244619
0.4702998061178889	0.4946781521538418	0.9743610116680442
0.3172459043852833	0.6570655276123466	0.9378591150739181
0.5493965042716517	0.4415846394905408	0.1188085219573569
0.7168833089509694	0.2708963443668089	0.1428591635233258
0.7601585736183741	0.2415337631730882	0.2995748605717714
0.2820723183263630	0.6801165098558597	0.7793218679560092
0.1964757315389247	0.1636612051482002	0.0174652335377935
0.0358676559565895	0.1109422039001575	0.2222900071644350
0.2569125312150313	0.2570011251366432	0.0630683335177007
0.0984194979669191	0.3261692187310580	0.0206628940226653
0.8133153591223732	0.9831318065226999	0.6318189162712476
0.1898600061690832	0.1544016947860699	0.6578531760257859
0.2019668076964315	0.9820696183306301	0.6596398324984020
0.2348006400834598	0.2036978854179134	0.4770476228596130
0.4003486320122548	0.1505217851274351	0.5093383334382396
0.0208926994388668	0.7824200072881382	0.6120415998901796
0.0012842473060911	0.6168148235832441	0.2147533841270869
0.1805190702382920	0.6363546131322041	0.0354098267316337
0.0619412483866163	0.4257776135874022	0.2619182745739059
0.9610132431885958	0.5254243593148689	0.1545062008398050
0.5694486206087439	0.5872140992936283	0.8288007444109610
0.6494309635408919	0.9956392868538045	0.1951191401902963
0.6289947342541051	0.1452397440706273	0.2236697331981941
0.4613872084787671	0.0706487997643234	0.2425891765529001
0.4885155232677572	0.9246575125174744	0.4121165355752148
0.5965741190584547	0.5713280295181415	0.0636305855458192
0.8180779484237947	0.8039179160996508	0.0247935870696717
0.9911353176365629	0.8200974661458285	0.8469649392251862
0.7636774905867936	0.7527170318553471	0.9443152344618027
0.8454182065318409	0.6054306486377515	0.0640968308775179
0.2365626861557463	0.9464705801095883	0.4355036379022834
0.8392656304291700	0.8177293608157318	0.3789229059572033

0.8159195167732692	0.9657242596574042	0.4103555203081471
0.7113940156519297	0.7351617018541575	0.5953990494923049
0.6327708770756442	0.8622927743864542	0.4681753174707188
0.9845163186105894	0.1934689350129720	0.4260334252024804
0.0191662219431231	0.3477294542982246	0.8318956878241106
0.8267127036894174	0.3186976012197630	0.0124824286466986
0.9271966845636594	0.5257856493817277	0.7886296950341483
0.0351067893968532	0.4658030356655644	0.8820587511128910
0.4164232514016146	0.3698128633386285	0.2476647744978864
0.3710672163863391	0.9656199747096685	0.8464172703086444
0.3987148368630687	0.7868835685558354	0.8549693939581263
0.5870969911514520	0.9516643378118553	0.7250971517743912
0.4500565784742258	0.0028676033402001	0.6590338159528323
0.4057933335212620	0.3732524189605956	0.0275569801997897
0.9966391306008940	0.4855412497564217	0.0254624376200226
0.4881817809855146	0.9799493335935208	0.5182699012540319
0.7883112475385951	0.3762106199033792	0.2427425825912790
0.0745077825198213	0.1599569709896553	0.9571345289467192
0.3981424558048987	0.7555293098494481	0.6400382295674097
0.1911305634990809	0.0350371769336605	0.8408513031558669
0.2176688218879716	0.5567923279569958	0.8347426301679053
0.9499354827948898	0.7987244072394196	0.0805496042930540
0.6262553627943227	0.1643388910013285	0.4267825586494676
0.8306928026965883	0.9382244556918345	0.2002197586988564
0.0391176230627324	0.9629225039742727	0.1565106757500858
0.9486811521900247	0.0306211331931219	0.4301002328877430
0.8593503488527350	0.1837887995600433	0.3188062495481246
0.9870397371574199	0.0007868435909540	0.8817623027093539
0.1203856955065122	0.8493368087862800	0.6557044295341037
0.1880941929796191	0.7559839368756036	0.7908858451417782
0.1452383219146067	0.8393856652088000	0.0476001829017939
0.3691629026073711	0.5578388456549415	1.0070115881262740
0.3256057367909885	0.6468608628600218	0.8547076468970716
0.8737880929952754	0.1283772863108090	0.9930633755583427
0.6424755606939053	0.3663589887818768	0.1332274148086118
0.7713785839049287	0.2456406809322106	0.2070753029815454
0.2441073047225173	0.0520239587847800	0.9445309503926473
0.0857548813243194	0.2071632573711025	0.7891861865578552
0.9288060112839276	0.7621416476173151	0.2491914799323489
0.7855152827479813	0.9121482356123830	0.0966422039588533
0.0435695917569020	1.0008265241218461	0.5291765560698531
0.5403211399650165	0.4614210493722078	0.0273212230230112
0.1672696589661571	0.3055802478138669	0.1970289590879241
0.3316611846085748	0.1596800229426709	0.3374324552597064
0.8017441967256574	0.6651198132721681	0.8493592516667341
0.6973181234540740	0.8075409747029594	0.6948565043482156
0.2017976566583806	0.3110589926512041	0.3165666650206140
0.2864650337238399	0.1450053373814014	0.2280919422177068
0.8070281377060829	0.6480709248147974	0.7049197142127608
0.6477626667100754	0.7893926882272144	0.8641955826299499
0.2099249255054453	0.6784008770248162	0.2455275483906871
0.1380071050436284	0.7138696811563039	0.3075323215921820
0.1733313733188065	0.5592252044716993	0.3057281889486795
0.0336953406348503	0.6327150240241509	0.4258145850266085
0.0689267155381158	0.4779755847245813	0.4242250475825189
0.9978042594573525	0.5132114543315075	0.4863090932559726
0.8861646172438257	0.4241647288679137	0.6162256855528394
0.7723490226315713	0.4590143483357549	0.6357159498902289
0.6714283801358678	0.4197741960459156	0.7468803519610990
0.7630986844956860	0.5252433582286314	0.5434399046744726
0.5648166813660710	0.4446132172334860	0.7653118822609467

```
  0.6574550230581623  0.5515512575504928  0.5611579262352719
  0.5574078394982508  0.5110896822669946  0.6724671674962821
  0.3256902564559160  0.7644406774232412  0.1197829257830550
  0.9078816719068770  0.4000071407087373  0.6971280023707885
  0.4345047767593981  0.7526902149032798  0.1180418841867860
  0.5179211734817911  0.8165758949228591  0.0257145788049714
  0.4255028507388776  0.7694118747791268  0.1691032255785524
  0.4422503613057620  0.6623456098362038  0.1587949696204043
  0.9892728403526903  0.3727373839659108  0.6801157852558910
  0.9231515845965080  0.4810835790051943  0.6776818291114466
  0.8331354152017054  0.3291214022772151  0.7929290952791526
  0.3215169957920863  0.8563569181087970  0.0756656814181221
  0.3371708522778851  0.7484464424475884  0.0672624945622309
  0.1641252812687571  0.8067107178523749  0.2618349311018697
  0.2270920361971091  0.5296225256891012  0.2590684275482565
 -0.0218462250338753  0.6631232671869711  0.4713165635147381
  0.0417586270693143  0.3855534769873098  0.4693225760473212
  0.8170373590868889  0.5837591821951198  0.7059129410420950
  0.6737603389178493  0.3658525635300473  0.8218774055563605
  0.8401950331865999  0.5573786632824480  0.4561763801377738
  0.4872485764114993  0.4111882635866784  0.8529127662000678
  0.6533893042556200  0.6037572344674669  0.4874174695563894
  0.4744487935579352  0.5310074214492103  0.6868678011335480
  0.8736413504752168  0.3394201121602847  0.6401832296510741
```

IWV-Al-T3-TS14

```
   Al     Si     O     C     H
   1.00000000000000
    11.6056394577026367    0.0835597589612007    8.7649669647216797
    -3.6825602054595947   11.3529796600341797   10.3282289505004883
    -0.2346490621566772    0.241282269865036   19.0520343780517578
   Al   Si   O    C    H
     1    37    76    16    19
Direct
  0.8824489233072077  0.6783503776690870  0.7320337100948988
  0.1241217615210995  0.2334114013180055  0.0737377814573789
  0.1153087931806533  0.0945415509966980  0.0215489601893247
  0.0823936974341836  0.3189761575675841  0.3019319266061715
  0.2346067762742614  0.1336153676196801  0.5814440470020281
  0.0898573839347367  0.1145468058799553  0.7915464674433728
  0.3136544688392470  0.1040201231481160  0.2979700956569424
  0.0333059746143576  0.5616877298632961  0.1636652775234079
  0.9837071564424261  0.7660800732098981  0.1474315604977003
  0.2642410390357271  0.2945771485474249  0.1161478780587309
  0.5403071901295625  0.0466549023961667  0.2666081733501020
  0.7594713828642093  0.0003443597423868  0.1238422118775768
  0.2698951621318487  0.2711662768633006  0.3486391188446794
  0.8358728828552024  0.7588472284030978  0.9623023083008712
  0.8632977798871950  0.8931645105326529  0.0164956411952663
  0.7265977213252616  0.8574845623900337  0.4619409704717574
  0.8853792058513968  0.8770781238769327  0.2488108066757868
  0.6612965165230437  0.8930670797310485  0.7284019600453631
  0.9280949669757930  0.4360501200121174  0.8688588727685191
  0.9871746186437940  0.2203455433609851  0.8984669299041504
  0.6927989863590234  0.6949474549626524  0.9268185053075183
  0.4323604719521079  0.9457072946196655  0.7677087471629531
  0.2129663739848923  0.9854351825423738  0.9201483975009453
  0.6817123190968994  0.7242593960286123  0.6960244262117647
  0.8844852223504903  0.5146358244702269  0.1122803579901056
```

0.4780065064654120	0.8953181570772473	0.5114799970637480
0.0629905656722439	0.4735319973605835	0.9331512701257777
0.4749469528190781	0.0915841284167375	0.5232921015831442
0.9236223761951329	0.0604376342677257	0.5021172134600107
0.8931314549687018	0.0876639178718864	0.3404143047216748
0.0934647843157872	0.9099961120989943	0.5517132952292586
0.1091519344928637	0.8718856307709083	0.7302634467459840
0.4576937273255134	0.5145909119501941	0.9637250195623943
0.3000196292624522	0.6752497126136811	0.9293005356950519
0.5193157280805522	0.4464565913811458	0.1259513134696192
0.6968739146561749	0.2868582551078355	0.1371756271143452
0.7444128130241452	0.2610592982372833	0.2873836684883584
0.2575931184613204	0.6912671939775654	0.7781318068254985
0.1758855195606803	0.1735242769769776	0.0182631746483110
0.0146083004982902	0.1300070474862373	0.2155691845689435
0.2315373017232689	0.2815114502434584	0.0552003911066317
0.0758475863093160	0.3402143303785391	0.0076016156613127
0.7887171903694516	0.9959507544674533	0.6269879029160432
0.1694522490535227	0.1763296242692741	0.6539564758903875
0.1783005765284283	0.9901365230960523	0.6786405286321315
0.2106367157712016	0.2033152483298634	0.4844868508213568
0.3784276336003032	0.1637986361627978	0.5124483635065527
0.0213971116734221	0.7846682726375392	0.6204640214192836
0.9794367276032772	0.6287698633467806	0.2117710410791320
0.1653578102871120	0.6479298857498553	0.0354806835223073
0.0381805737570108	0.4361531529044465	0.2629772461841035
0.9493082633067460	0.5409590362270749	0.1441481390741765
0.5697153364764980	0.6025844253182870	0.8230886977798088
0.6225108591827028	0.9978531652670856	0.2021301674279312
0.6256134359021335	0.1536934433480711	0.2160236679129152
0.4480832871756085	0.0923397999962336	0.2350747931422439
0.4647990163145805	0.9433617207075632	0.4117994981118989
0.5775477922063874	0.5774055622302314	0.0615244458195273
0.8025866429030514	0.8191667019560394	0.0156159984221914
0.9682931346803761	0.8358302536038119	0.8331637983922855
0.7380797108379632	0.7595193712823342	0.9463306641178967
0.8326647347213715	0.6224498753201737	0.0552179904830203
0.2187030731812002	0.9708425106447935	0.4182182624545224
0.8122891877156491	0.8251866726517506	0.3815491746546138
0.7971871492073301	0.9812058471698814	0.4008871217940564
0.6919337901905974	0.7489282304845433	0.5932476283531423
0.6088648027891967	0.8754695769853432	0.4682052107270022
0.9634165320684294	0.2031492703876788	0.4222137533063171
0.9933306437336696	0.3574119041248788	0.8356429135981130
0.7964383634865785	0.3504323042504383	0.9988304254080522
0.9131859053840299	0.5433592767049180	0.7666417228587241
0.0084375840957081	0.4888643150193122	0.8726640249417048
0.3868869418850615	0.3828054413222761	0.2546424595406351
0.3456343386045121	0.9802881884191770	0.8450522656242809
0.3724162041807056	0.8122156358799932	0.8383852820215145
0.5560397600228898	0.9564317404237922	0.7475209873693847
0.4540134180327703	0.0360181538592744	0.6417838155823749
0.3882848961504736	0.4036783596048547	0.0055925919550294
0.9777466776370123	0.4966764510560180	0.0209578388177263
0.4613559162043251	0.9884994502299267	0.5270770372382787
0.7716686615454229	0.3931335908626507	0.2362394885476053
0.0523970716212602	0.1714583159986391	0.9586324335163543
0.3746997567508700	0.7686312947163998	0.6389359174725364
0.1658443092238126	0.0515407571230404	0.8413654763142833
0.1956731232201165	0.5706137466547995	0.8275826140914950
0.9228431602498266	0.8126729141837369	0.0828181163362561

0.6074847204398525	0.1861228024593365	0.4076671829020297
0.8069188532188443	0.9395580282775002	0.2017775175994833
0.0127709840051671	0.9735365833051930	0.1599673442909875
0.9269416629597267	0.0416999296633985	0.4258805437391336
0.8369146066268158	0.1974878324818143	0.3152839138697718
0.9664717116647674	0.0145588320219603	0.8781425380877400
0.1316968860111719	0.8986492828915993	0.6224499506431795
0.1620224765307966	0.7657442957904003	0.7907689834950474
0.1218460383234948	0.8509644776023789	0.0460360884039789
0.3635345583115898	0.5865793939263628	0.9885152422897554
0.2972846821644512	0.6540502531273605	0.8574447964984661
0.8478959767699780	0.1371235549102953	0.0009417990049810
0.6038418809406001	0.3623114112289735	0.1502524312616666
0.7653147017509812	0.2731034182903888	0.1863950711569542
0.2188585551212633	0.0602223963242903	0.9490855338205094
0.0564045766118037	0.2196765938905120	0.7929340185356833
0.9109002318983626	0.7693768377674413	0.2514054255383644
0.7613715383976296	0.9281352726989773	0.0898281860456032
0.0173394901814793	0.0064549229277867	0.5298019604860361
0.5043818513889945	0.4579572574260187	0.0417102624557402
0.1547534486160006	0.3226018378504557	0.1930860206811068
0.3099376340136692	0.1795521199966362	0.3293455159775345
0.7962224792392267	0.6580980321199119	0.8590903797896994
0.6510348664565757	0.8378022215550212	0.6841216789619156
0.1707011212815509	0.3196898315377973	0.3275710449908740
0.2840105686842719	0.1722671391313524	0.2047674705722497
0.8031633451611744	0.7103739329710167	0.6798882319053559
0.6418636415128077	0.7874910590327798	0.8531692201783838
0.1468258805734739	0.7225930943967673	0.4208226391959842
0.1485750760403772	0.6039041103092829	0.4936896657847781
0.0366604048494406	0.7436470166295716	0.4461084505628868
0.0443543031097777	0.5093747132942519	0.5883227715722510
0.9318376344159371	0.6502819607757734	0.5396524326740862
0.9292170656170452	0.5289899357358866	0.6205463737941329
0.8224524754460786	0.4235272723135270	0.6993509242038154
0.7302940173482300	0.4631802658605148	0.6775352892806501
0.6131641416872287	0.4443202563409409	0.7651916554946296
0.7616495236235310	0.5176390602507115	0.5675195896708214
0.5294866252281587	0.4794802664593021	0.7437669630077636
0.6784386284233174	0.5525056590000823	0.5458512947494388
0.5618695421833889	0.5339054467150210	0.6339823481190192
0.2596809970200622	0.8257708789460569	0.3146561341506693
0.7667055146347777	0.3350551712860718	0.8317611665421460
0.2869607390110456	0.8867157246431057	0.1963359082651773
0.3680126342011034	0.9639971027528661	0.1197837905300866
0.3012329187748587	0.8244631314734281	0.1846322875444839
0.2124529697946976	0.9180292621476316	0.1949122488977603
0.8342786155645088	0.3004660939556293	0.8439551359124602
0.7288180022428563	0.3770008023775993	0.8675393152268465
0.6951591394527524	0.2590457599907064	0.8851305223170249
0.3352685258108772	0.7956487180120636	0.3143585119051604
0.2483649108388322	0.8906633195519035	0.3229896620936883
0.2340446881546688	0.5871404766936759	0.4732472116667898
0.0348161123424727	0.8350178770289177	0.3890784484223364
0.0468389814542859	0.4180373313064323	0.6441067469368977
0.8482724468929737	0.6673902010503560	0.5550618091469759
0.9192273306393742	0.5345337727394590	0.6892377352369441
0.5854456266648308	0.4015591331163070	0.8518019710391057
0.8525169798472952	0.5331899849283408	0.4976757105447547
0.4390768002736746	0.4639082382681106	0.8137318531925117
0.7052572128735403	0.5947412549829975	0.4596032761895152

```
  0.4966847910148157  0.5616581932088113  0.6171312576316355
  0.8641127591405663  0.3770943345013766  0.6683499594631963
```

S4.5.- IWV-Al-T6-Dyaril

IWV-Al-T6-DEBH+B

```
   Al     Si     O     C     H
   1.00000000000000
    11.6056394577026367    0.0835597589612007    8.7649669647216797
    -3.6825602054595947   11.3529796600341797   10.3282289505004883
    -0.2346490621566772    0.2412826269865036   19.0520343780517578
   Al   Si   O    C    H
     1    37    76    16    19
Direct
  0.5280505775796331  0.9375478627326079  0.4678631051794564
  0.1632755227733953  0.2673988588999604  0.0304112200275860
  0.1250567736755820  0.1236851624665924 -0.0100862351946868
  0.1117265308984474  0.3436166408356089  0.2630498326380083
  0.2589169999102648  0.1699207073354034  0.5389722849225784
  0.1072174999016735  0.1377458713640377  0.7593084337485473
  0.3381153520032613  0.1309293753259458  0.2700453388069006
  0.0619970451080124  0.5829549234248894  0.1208755672619766
  0.0121093560503115  0.7904056170140822  0.1070531516721414
  0.3034795908774787  0.3203537243120927  0.0751452812629774
  0.5764633809661104  0.0827966954744332  0.2185088772652802
  0.7748610927064058  1.0164935074878978  0.0929846276376474
  0.3023196599875775  0.3060667229351545  0.3040469538307478
  0.8714218198915559  0.7854560664713348  0.9214377256082538
  0.8830272266367714  0.9133954267698926 -0.0148767998782608
  0.9161271054578205  0.7027007067288685  0.6986960922556051
  0.7660602565208048  0.8777742038341804  0.4236440245800783
  0.9027275563452719  0.8941081344851838  0.2163384708912097
  0.6874133652499106  0.9211628679318338  0.6924217081512085
  0.9611806719358329  0.4630707232662732  0.8373216330167697
  0.9992640786876487  0.2447432794100206  0.8677803413005808
  0.7301924303089617  0.7292576916772284  0.8803065292226693
  0.4517421394402681  0.9777454072233841  0.7352921928504773
  0.2339698129352462  1.0155272578048700  0.8822426924383800
  0.7281378582537781  0.7434277954378082  0.6542710107704138
  0.9266206276153212  0.5397813796007918  0.0619168953856937
  0.1194028011291549  0.5060885734659232  0.8811114426520801
  0.5067489900805728  0.1294666371084317  0.4937282937983545
  0.9075394583943636  0.1171228703383906  0.4385337369767420
  0.8863310225691831  0.1495461441510123  0.2654939967041509
  0.0746681776231069  0.9677303121298027  0.4920676557689936
  0.1003567165934432  0.9396111985897775  0.6546162863445111
  0.4701668946034772  0.5782259298837042  0.8754314104712131
  0.3006675287874569  0.7446751864054327  0.8468320283365042
  0.5244074709682129  0.5117949852321118  0.0406000638851838
  0.6854146902128894  0.3480901077140979  0.0677722689012340
  0.7222974976643078  0.3149409105587739  0.2291811454027622
  0.2584716535029876  0.7733121075626488  0.6889120490875881
  0.1908563833381636  0.1950000436204470 -0.0100366960791605
  0.0263179075307423  0.2091305009674094  0.1493251385708763
  0.2551282192370060  0.2580225146186782  0.0563756825614583
  0.1875942390168086  0.4079404895272229  0.9232759802682438
  0.7836621936677441  0.0549194954736198  0.5716444934621605
  0.1760003717180895  0.1982089556814838  0.6239379238596336
```

0.1935717531777052	0.0434088053659277	0.6005268356796986
0.2758465486921322	0.2766650638494340	0.4149403250128647
0.3843376562730786	0.1673379862890179	0.5169094214469677
0.0275619941175192	0.8253255171704481	0.5761494266135381
1.0105478164661854	0.6546255943194228	0.1645804699521671
0.1858101747012164	0.6701626230579086	-0.0137965051185049
0.0874201577659663	0.4717745293889274	0.2116324458695039
0.9627589128520804	0.5313665874501643	0.1327466485291643
0.6067189906350310	0.6327410034760930	0.7542122518765870
0.6407996149597974	1.0221245632066474	0.1630517766953002
0.6478688996027150	0.2260189458999294	0.1078642128336641
0.4416371581058506	0.0671205552234350	0.2575829728567157
0.5817794480015417	1.0285886535179376	0.3213810952379683
0.6031391263907866	0.6241987509327347	-0.0108531889225620
0.8258076773758979	0.8331591179207251	-0.0124602666456602
0.9766647720725421	0.8944607760288661	0.7800116284798981
0.7573550818536445	0.7454986554124063	0.9424246420645046
0.9226154513168068	0.6743909468003498	0.9753051897197340
0.2114263136585990	0.0274170245067110	0.3705840000166124
0.8299632399373935	0.8330014101383947	0.3520759904752424
0.8403040206955235	1.0188393572543968	0.3237765256181554
0.7916867440956999	0.8007353130715272	0.5214353326419116
0.6262927594306051	0.8556409481033432	0.4888585738597442
0.9809517981495509	0.2428834105099273	0.3731923942618803
0.9897540669298949	0.3789325175895877	0.8088911654767104
0.8273465092876832	0.4110328652023655	0.9572580264451670
0.9756439560815739	0.5947397927327565	0.7257996543640031
0.0537947672701380	0.4686693057369255	0.8562035567676919
0.4104581051881430	0.4314732028771688	0.1814493616929941
0.3723274866683777	1.0175587039280600	0.8100708746728690
0.3643868201197907	0.8682078633685695	0.7907228777536012
0.5542174944919097	0.9349782867816864	0.7529515579011314
0.5108760083721755	0.0919078845855832	0.5956716078869768
0.4071522540297302	0.4463528747818161	0.9429209587316335
0.0257097040919505	0.5087466075850421	-0.0154902124935368
0.5167411015841162	0.0255106222921641	0.4964353247618625
0.7964606173133222	0.4449051384920995	0.1568318921329133
0.0623089010038285	0.2084572119992455	0.9284394744167470
0.3874573888420675	0.8410502751133192	0.5639576892273576
0.1899242513990239	0.0732088761516773	0.8014881589927341
0.2189502317533714	0.6343163579819471	0.7571599906941809
0.9492956282824038	0.8383962537182141	0.0446010270718641
0.6180035454943088	0.2532310143004302	0.3662706481185352
0.8214519459635625	0.9520318391258017	0.1722192204192778
0.0254562236003557	0.9986166508298273	0.1251707909093736
0.8734144585088578	0.1419675761453813	0.3605889367769688
0.8126521735309796	0.2350407358150675	0.2202785249331632
0.9816387812080234	0.0387066032808866	0.8484659786920717
0.0682101873156574	0.9922920831249111	0.5655383873919942
0.1549308938817283	0.8305887205737345	0.6757173827592202
0.1490279565994742	0.8779170301288034	0.0066120674607750
0.3930575013024261	0.6664533235016983	0.8401127308261656
0.2509545478682650	0.7726041099891218	0.7789945214332852
0.8663779800131560	0.1518022627007665	0.9680706793274285
0.6098308877665316	0.4319940751416863	0.0229961824706939
0.6620451979142296	0.3320919239026776	0.1716917976041349
0.2281181349734217	0.0951710931213505	0.9103572475288561
0.0798417785085314	0.2416622327854874	0.7629773049184898
0.9377448176399347	0.7892642441506046	0.2140427014045020
0.7752935096441264	0.9411211277388120	0.0623304806238797
0.9898284022537214	1.0279259078541498	0.4522055100996405

```
  0.4727185231718973  0.5643467949920867  0.9670469204502364
  0.1915275206215329  0.3433543433139138  0.1566925333784097
  0.3381079796070553  0.1977152641231309  0.3089666891867238
  0.8413276527627490  0.6956550715510773  0.8101221507283416
  0.6940151359362409  0.8447509146520568  0.6610343776478449
  0.1817011920737954  0.3214765702224444  0.3134797231794513
  0.3599415674358267  0.2325109054608551  0.1425571572975932
  0.8225642754050018  0.6914782753563377  0.6825383185566511
  0.7177144269614980  0.8528010517233499  0.7849210865765954
 -0.0441331861416872  0.4587563859996488  0.5040405052043273
  0.8474936602107491  0.4123144887099670  0.6164293549854440
 -0.0485169539616420  0.4673975894077588  0.4273426985512091
  0.7386876505886854  0.3763586044180960  0.6510067926040538
  0.8436157242502357  0.4325978267077081  0.4603622502219400
  0.7337141784211868  0.3861928972365327  0.5733356660167623
  0.6259290795032613  0.3547564193030966  0.6017190641856733
  0.5726977453332207  0.6017184312378021  0.5515493471715877
  0.4480742196027392  0.5634518393095601  0.6341753139605391
  0.6276545087328499  0.6078956725743579  0.4574612354275551
  0.3778774578972565  0.5316899372509767  0.6226129112349144
  0.5575865314651464  0.5771206799347772  0.4452242191239396
  0.4325805283113065  0.5388594933596919  0.5279924253424829
  0.0757083736599680  0.4994286757419026  0.4628358810317614
  0.5048282595038175  0.3009119766395273  0.7127858729726975
  0.0751179603457429  0.5077120719733511  0.5383709544059410
  0.1672792899823508  0.5423188776490243  0.4973394245541997
  0.0252863667786911  0.5676875803856922  0.5460052877031518
  0.0351381835553964  0.4200120068627447  0.6290024725056689
  0.4594615365894429  0.2205160887408256  0.7466005268268854
  0.4986988525188641  0.2798807643140375  0.7832473941108230
  0.4514743650527250  0.3625048657086459  0.6916792061144477
  0.1259406489431298  0.5860261462758011  0.3688929777995635
  0.1257168153402644  0.4379427393903621  0.4573579762088276
  0.8503125733224302  0.4058078722282684  0.6757262131673079
  0.0344795692033857  0.5033037991088301  0.3405989927451599
  0.6560188071023370  0.3418631015684264  0.7373408417407854
  0.8407295028352834  0.4412078536611256  0.3999872088296632
  0.6273586316146049  0.6284554842418155  0.5590357756780173
  0.4044649278908319  0.5591762701923076  0.7070847079585421
  0.7252013020545586  0.6392687735785479  0.3921684885732319
  0.2803800443377968  0.5025438063075039  0.6869356750969842
  0.6006967743440564  0.5846493050036788  0.3702416188755707
  0.3776705603967104  0.5152372463924100  0.5186762761630610
  0.6340398538294685  0.3680477929828084  0.5338418135835619
```

IWV-Al-T6-TS6

```
   Al     Si     O     C     H
   1.00000000000000
    11.6056394577026367    0.0835597589612007    8.7649669647216797
    -3.6825602054595947   11.3529796600341797   10.3282289505004883
    -0.2346490621566772    0.2412826269865036   19.0520343780517578
   Al   Si   O    C    H
     1    37    76    16    19
Direct
  0.5245582006684870  0.9080865513904328  0.4839853980845064
  0.1582992680625921  0.2370483794877174  0.0494549646438216
  0.1205449470896696  0.0944309691172833  0.0068160284392746
  0.1069750093962654  0.3131311766945970  0.2813906895821816
```

0.2550675810004606	0.1395075311159601	0.5574201226949711
0.1030664098937564	0.1094258013577587	0.7760080762900046
0.3339335649405797	0.0993549764356629	0.2880400476020597
0.0577337571435450	0.5518677528805398	0.1399876163896963
0.0084539684716063	0.7609062156319908	0.1238181368598603
0.2991507895625602	0.2899386325798332	0.0933727252766872
0.5727561725829631	0.0516819695751565	0.2348617309481256
0.7705515990229372	0.9857998100337753	0.1096527047881679
0.2973243013798879	0.2729171289305938	0.3232392116310550
0.8682190389419731	0.7544984766599612	0.9388506439139164
0.8800927383801707	0.8841367331891519	0.0010308870193967
0.9115169213894134	0.6722497014335929	0.7172708143041575
0.7628292759356299	0.8478149820598375	0.4409022478822138
0.8961034371327443	0.8634095187382727	0.2342505407768840
0.6821748712791162	0.8897336054625811	0.7113650678886856
0.9561978389368448	0.4325067927600514	0.8558525664009267
0.9961456641049438	0.2161631696897501	0.8835021215445025
0.7244153814564456	0.6966435039455376	0.9006032003704760
0.4468455989934554	0.9467079942323551	0.7526762636317059
0.2301456793083929	0.9865904328905017	0.8993619508726240
0.7247272183517381	0.7135053067162076	0.6713173320900054
0.9249034411237744	0.5093843887368763	0.0786113533438098
0.1170423122105291	0.4755359149404867	0.8972722405261919
0.5033910429287189	0.1010375722855272	0.5094625668281623
0.9022982440901686	0.0859656659186214	0.4581505516994113
0.8818530298368117	0.1186252077662715	0.2848328799375039
0.0710170763427698	0.9378315478796040	0.5100573636650836
0.0976861161626985	0.9093533188554375	0.6720462597809913
0.4663872058772753	0.5478953133670004	0.8933855164790003
0.2956839128459663	0.7129426551627347	0.8655724075550637
0.5196176658035867	0.4797801920342479	0.0594605805689810
0.6808339298060417	0.3167611559760957	0.0865214253334159
0.7185218918243166	0.2849020897348069	0.2468613266025130
0.2549434020588492	0.7416778432265190	0.7065196029500953
0.1820623704884312	0.1619316635672732	0.0126790360022753
0.0217903682069363	0.1824190523602240	0.1682990324999025
0.2502812841134272	0.2272858253732863	0.0753654808761297
0.1843233026065505	0.3771974317086801	0.9404044649123762
0.7794791823619605	0.0232185524565338	0.5921245416158527
0.1733289026224725	0.1703530447629973	0.6400838117823682
0.1898692775374613	0.0120105181049155	0.6206141867691102
0.2729581307102364	0.2451914479453308	0.4326349196627035
0.3806046273920458	0.1396116619332446	0.5350641365891775
0.0229782639683904	0.7950834643979476	0.5957935902187188
0.0069145113444264	0.6254621969924727	0.1811931004988239
0.1817144137546265	0.6390517205730006	0.0048360132510155
0.0843279932097545	0.4415529500173605	0.2300334795506132
0.9601606711404855	0.4997308575298902	0.1504506554996254
0.6030910724216838	0.6028820336833007	0.7725443671563426
0.6365857495460574	0.9933085840411309	0.1769536125760780
0.6440910314794337	0.1946772318401031	0.1264496442648309
0.4376156643782352	0.0358339207480824	0.2746853190835523
0.5782848786536903	0.9940526698375081	0.3384125694608784
0.5977570313088592	0.5895450741980071	0.0122584693595016
0.8227970223998113	0.8032684244264630	0.0039655270570194
0.9724382062979515	0.8632575039761363	0.7971157996433149
0.7526821379274438	0.7136049661732784	0.9617967787992967
0.9194514668030607	0.6438126374164455	0.9930340841996568
0.2074862252763126	0.9954159556039812	0.3887049838053116
0.8248464627331131	0.8031220976913644	0.3694268384129487
0.8396017011837316	0.9885678988495172	0.3400972798824498

0.7885092214324558	0.7703323252592895	0.5386844171801641
0.6232895648848782	0.8285203535580123	0.5058794223050356
0.9759555166857794	0.2126883256360657	0.3909808429213371
0.9832679023639841	0.3491025557178773	0.8268147716872534
0.8222914780431552	0.3814290586295357	0.9752829777980381
0.9723484591836922	0.5650554213347689	0.7435336294586250
0.0486728004178900	0.4363186109706660	0.8754241769990685
0.4048089233948247	0.3997244736888490	0.2006422870373541
0.3676654059770215	0.9876745040057671	0.8258982588094739
0.3593603675490300	0.8383252569402657	0.8067842271809269
0.5487304813184934	0.9030991441778298	0.7712343561072643
0.5089620527075098	0.0599383590927971	0.6117987883667269
0.4031386881561261	0.4157561397718652	0.9620628436545076
0.0243074040159428	0.4800762944036663	0.9996584988069301
0.5123043869543551	0.0007186738730454	0.5078056286713348
0.7944366219389991	0.4149194606442208	0.1727602822317778
0.0631022980681450	0.1830468266560956	0.9399461708899182
0.3839471071216920	0.8109447965059644	0.5812572501045778
0.1853900991227633	0.0460515266007917	0.8186608577429837
0.2157721256450529	0.6040059524971682	0.7716505544116856
0.9464502580087384	0.8099745551310065	0.0602722383399765
0.6136323562806822	0.2260261136048030	0.3830857457761425
0.8113181697350385	0.9171448282829218	0.1938848166893834
0.0171470020170617	0.9707717143578520	0.1407652764946477
0.8657670750339790	0.1087507813665454	0.3825370015483642
0.8060961340935719	0.2022217408524298	0.2412689786664117
0.9782753312491295	0.0099309232734441	0.8644451671488582
0.0675965711039836	0.9645025306187165	0.5809384488963047
0.1520008821547876	0.8004984099092454	0.6927024960623172
0.1453560670998897	0.8486294372835772	0.0230810514414372
0.3893206612776581	0.6360972196153440	0.8584716356725082
0.2457557440779610	0.7384048638434564	0.7986618794646887
0.8648972273553994	0.1197856304640439	0.9861271195425516
0.6039877918953493	0.3993087496480356	0.0427574843921974
0.6574473084475159	0.3016523801787230	0.1900471642231878
0.2238568655796840	0.0641209788346089	0.9294674127490339
0.0761845550502542	0.2152139228605705	0.7770873967992458
0.9334200495046048	0.7595899110938049	0.2309055052876658
0.7710258137001418	0.9113416690802438	0.0780966937035501
0.9859575260793179	0.9979429789238523	0.4706313736069845
0.4694024300721225	0.5350750124207551	0.9845951304510194
0.1873971970721797	0.3130100882202882	0.1750283107556854
0.3343488562499743	0.1650839172455107	0.3279090273215459
0.8353741455170862	0.6634445633659212	0.8299859273548478
0.6916805624380018	0.8158431396364776	0.6768137001530690
0.1766257081985714	0.2883246934645101	0.3332626290117986
0.3552622524274724	0.2018750410791554	0.1612274748663367
0.8193032217207090	0.6620162270434591	0.6996311495880079
0.7092570128004141	0.8184686363854397	0.8067555500862227
0.6805843500511060	0.4004353247054340	0.4737770503852374
0.6541819712657073	0.5017883528955380	0.4552740365148154
0.5986870709607359	0.3232553948825156	0.5076154402840629
0.5482425109821065	0.5243583583462339	0.4718968288104151
0.4936664678315879	0.3462319664426659	0.5226908920382911
0.4657693289690030	0.4459229152296018	0.5074047790277803
0.3508408386492761	0.4649403113122261	0.5294210305083453
0.3825729041962376	0.5864841415344060	0.3846300768815709
0.2668485363767985	0.5934754793029882	0.4111856130387679
0.4573037174386485	0.5577650570074866	0.3132977716952752
0.2235885318409245	0.5578886434334223	0.3838447053320593
0.4145222021099784	0.5253111226830569	0.2838671134645239

```
  0.2967700971701248  0.5230022845639439  0.3213367208384597
  0.7916051679017002  0.3716148098408425  0.4618046619384586
  0.2813257548784236  0.4984235831860317  0.6018511436752263
  0.7637446066693272  0.2745540188281552  0.5850595452561578
  0.8456258494811522  0.2532199197062326  0.5745929066511763
  0.7266753233223398  0.3044443539435520  0.6324078335864485
  0.6979475759554958  0.1922850178976603  0.6412258714416268
  0.2630764252719118  0.4260123352552299  0.6890475632298489
  0.3323563982590727  0.5808255329655481  0.5601258904078605
  0.1950249011847731  0.5064960075701421  0.6189937313738980
  0.8586472574749564  0.4526401576291050  0.4061255439965024
  0.8306831613029999  0.3396423557204216  0.4161481485055134
  0.7180179991403719  0.5634059330989061  0.4271078461009523
  0.6179174745326108  0.2446796940066491  0.5215586053847829
  0.5296536146683337  0.6036491820862295  0.4569906139143072
  0.4316801197768285  0.2859889833507196  0.5477197113446195
  0.4303742750724879  0.6545805367884086  0.3633287592184757
  0.2120671410994817  0.6234479009248517  0.4570670159280610
  0.5467565035819746  0.5564035872736781  0.2885534232280378
  0.1340215094765114  0.5583956383367337  0.4089076415055146
  0.4700020067589605  0.5010144473329916  0.2326281659358145
  0.2623717666910679  0.4941896155306722  0.3004090918035871
  0.2948362005892378  0.3910030237675670  0.5639660322857125
```

IWV-Al-T6-I1^{+}

```
   Al     Si     O     C     H
   1.00000000000000
    11.6056394577026367    0.0835597589612007    8.7649669647216797
    -3.6825602054595947   11.3529796600341797   10.3282289505004883
    -0.2346490621566772    0.2412826269865036   19.0520343780517578
   Al   Si   O    C    H
     1    37    76    16    19
Direct
  0.5121528894905483  0.8962609516645897  0.4939078370066200
  0.1461070976505911  0.2280665632197968  0.0612746788002355
  0.1193156539647732  0.0960504565247021  0.0072009772224655
  0.1037017483950198  0.3139231961107333  0.2833894818614243
  0.2510407502412337  0.1340842917456789  0.5614633197819849
  0.1016907567327638  0.1092598560402201  0.7769877605140607
  0.3253684567121191  0.0957114125935163  0.2938733679482310
  0.0471836508107910  0.5495130925871964  0.1491496772515942
  0.0015913601172661  0.7619057941013898  0.1268708676201772
  0.2923061978238858  0.2882635479627801  0.0975709149778206
  0.5609259516983278  0.0488627472696867  0.2420371256285263
  0.7697119682141405  0.9954055785554267  0.1076942979778518
  0.2947127268742514  0.2709066871059715  0.3260116544977400
  0.8555372764469441  0.7540714376795919  0.9483094898164273
  0.8766351408168885  0.8874133271063271  0.0018493832006549
  0.9008735246969205  0.6704480821858969  0.7234780903918239
  0.7477149254614867  0.8443218344501785  0.4483998169873712
  0.8940200488523972  0.8704972524613827  0.2334638376099716
  0.6782263661170403  0.8930966272070660  0.7149599058754087
  0.9511560851950470  0.4317140933886183  0.8586141177304522
  0.9966808564011159  0.2199268647357598  0.8814084441276675
  0.7175946156174657  0.6986481921684269  0.9038858800732138
  0.4419184867940879  0.9422014136388240  0.7615383991755891
  0.2253509006246840  0.9853006563167955  0.9033392065334955
  0.7137233624064463  0.7157107156275063  0.6771174722052509
  0.9062080418646394  0.5076191309045128  0.0949259242817998
```

0.0974130768145334	0.4715680256242871	0.9150976977213051
0.4970907576706500	0.0867185625298115	0.5193539739541460
0.8970031140684579	0.0861127290302677	0.4607181035991278
0.8710454580378242	0.1148754059359515	0.2915744906122537
0.0613740407230639	0.9339707578308417	0.5172858948246134
0.0864101610444474	0.9041488951619089	0.6815021983171543
0.4564613931636124	0.5432827233582371	0.9004631376583608
0.2872426765281757	0.7098222439121092	0.8745087732116460
0.5149890881812910	0.4808492565199636	0.0618507762718322
0.6751537209271110	0.3163506758855220	0.0902739285207080
0.7138213853112944	0.2841094232316524	0.2513439618004927
0.2441424889288935	0.7387402738710959	0.7179539742413490
0.1840400604300376	0.1679998007534081	0.0073578446598005
0.0114497847931886	0.1515786067305756	0.1861847001093032
0.2402523446726283	0.2239284200444573	0.0828347762318783
0.1554186678910029	0.3667147577327404	0.9663311775545074
0.7738070258130857	0.0267129800635091	0.5930351046495976
0.1734210104728184	0.1672003057668210	0.6422504953431951
0.1798518527064050	0.0072745830162652	0.6276631218591834
0.2689576450143221	0.2399322491625180	0.4371112500808874
0.3762459197899645	0.1264554202935367	0.5399368699108189
0.0146087411750728	0.7913672477226440	0.6014516902096898
-0.0008076679593663	0.6251043972127670	0.1882762912965792
0.1672605152846040	0.6356910694248693	0.0115214037519319
0.0775936251269075	0.4407529267813824	0.2362008750718078
0.9450562174121887	0.4948899435043637	0.1657095592872612
0.5926011819101604	0.6050257541503703	0.7762126996204874
0.6340140821791549	0.9990639791177077	0.1760084898740251
0.6226013956126201	0.1926351349590869	0.1410014816023752
0.4266055604616409	0.0285582526887256	0.2813131520668624
0.5637999893619290	0.9855901404739892	0.3485590870213983
0.5882036121457180	0.5975578115229281	0.0093329433476261
0.8108538786275783	0.8094610520132520	0.0075841642233000
0.9621545783917008	0.8588744974404241	0.8064171652845352
0.7429603548261152	0.7104792422839961	0.9696704261246053
0.9031347864131505	0.6431817528398700	1.0098128763472567
0.1976786911400550	0.9931303337248349	0.3955143955300969
0.8234161944524084	0.8089770324748662	0.3689560913280201
0.8017994817455514	0.9881682499213104	0.3575881863070758
0.7747272568440688	0.7744169018940810	0.5442741538701132
0.6072847583686217	0.8074286291582814	0.5148680738485919
0.9741576813161350	0.2118526066708920	0.3917358557617822
0.9925325953306755	0.3556520201530560	0.8202929446555216
0.8169639310347451	0.3658551451000940	0.9821243491286353
0.9586165360565928	0.5615610196645187	0.7511570884454164
0.0401579793374558	0.4454067245540420	0.8774436637006412
0.4042073065727609	0.3955076722811608	0.2040796191747493
0.3627260859411714	0.9865454034996780	0.8311318022764412
0.3550814974668656	0.8263465052300485	0.8249236953257963
0.5450794960095557	0.9050473346013042	0.7785594666692325
0.5005353481058972	0.0491037358291672	0.6213105972503461
0.3993013761717366	0.4115288122646412	0.9637878721499222
0.0021047126508757	0.4790663941886733	1.0140035722847236
0.5057881510787476	-0.0181601624402879	0.5230689137437817
0.7755194300192957	0.4134444251243101	0.1896169362670280
0.0581383771998954	0.1811012741918486	0.9439820622117789
0.3704344657617826	0.7996352762435104	0.5913769157790193
0.1806874755848219	0.0429185894807043	0.8232079501132985
0.2046637218005564	0.5963247126652860	0.7945972336150562
0.9418986873050816	0.8077567575847697	0.0614831047102866
0.6095428246905086	0.2088525928927111	0.3919246102475428

0.8109653947839603	0.9315148294986649	0.1913566787628662
0.0177595392955447	0.9725465096110968	0.1431508242929160
0.8612042745385790	0.1078826022017235	0.3850022718315111
0.8156453321126583	0.2166993532290396	0.2305697977369865
0.9756607064493333	0.0112574361465882	0.8649428075334025
0.0564540516206027	0.9573075593271148	0.5909206330360909
0.1397609976984950	0.7933596660645936	0.7052875328177312
0.1385621827312524	0.8482365239507205	0.0281952473796623
0.3737612809593892	0.6260810528529787	0.8706150717111971
0.2462253568924234	0.7509988655565651	0.7959098565260797
0.8612164499902862	0.1301603431597527	0.9828440333981594
0.6081679524704063	0.4084779679271986	0.0380782816845081
0.6572760523240616	0.3043166316492351	0.1899539159077161
0.2212385588338471	0.0650004583565216	0.9306214641247564
0.0747152376808977	0.2143246325720048	0.7787756360331227
0.9237109988622080	0.7664516307452479	0.2301141596263139
0.7728025088585652	0.9171796064075148	0.0789648008773570
0.9760322757983729	0.9936719681510371	0.4783170081202722
0.4591676157987060	0.5273901783469170	0.9928636860133032
0.1841628287601834	0.3181828755183476	0.1737551199367939
0.3298383455255202	0.1617249106393602	0.3317439086631045
0.8266621450914906	0.6619123623951502	0.8357751261968462
0.6809323560363272	0.8156966952433592	0.6854537068049124
0.1750505865619159	0.2874768498530221	0.3339423245952030
0.3463070095754586	0.1987237701408905	0.1671606354694654
0.8090455372209244	0.6629567654673852	0.7039674079821407
0.7122723255941409	0.8254243918721103	0.8046678507791271
0.8745475746791769	0.5043765964154834	0.5182122848185942
0.7899622829073559	0.5258477388200234	0.5907270850114623
0.8411130248889179	0.4767199089584349	0.4783813483449297
0.6759609537894978	0.5214440607413552	0.6215628410401701
0.7272439087766545	0.4722211549097750	0.5089767534278979
0.6428009633986481	0.4938011882307930	0.5815731649975046
0.5198181874885837	0.4854850698566577	0.6164735580153726
0.5124170387333381	0.6243799205591098	0.5242602013407694
0.3881844458248765	0.6193068521464683	0.5721115532198661
0.5940648096514096	0.6692760696628638	0.4042705672534062
0.3478157823538353	0.6444674861615248	0.5120064200933856
0.5529661551547982	0.6945891738688943	0.3449504057583933
0.4298603761096703	0.6808377899640208	0.3994318614082281
0.9979253929281633	0.5092628643778230	0.4846507852182680
0.4197755516990004	0.4245624158702163	0.7463896843069038
0.0091226659212845	0.3842789981098148	0.5621829622184139
0.1000572971362698	0.3894445127395965	0.5355487217640982
0.9455693245230049	0.3378981927066224	0.6573479792627350
0.9895304220744517	0.3299157228622247	0.5530392400972999
0.4319442390698557	0.3394675504718132	0.8042286640193508
0.4205419663485396	0.4779923368715278	0.7638924221727776
0.3314770214444321	0.4055265097610573	0.7721093894332908
0.0188247370767801	0.5628095819162626	0.4940089803539251
0.0642868381605009	0.5547619618683589	0.3896491910591148
0.8150205377688617	0.5485469412415946	0.6214538595872653
0.9060766524519075	0.4609598720545830	0.4208917513699139
0.6128668197797196	0.5402994433316202	0.6766150117590113
0.7028300801373601	0.4514033071522176	0.4765667136808602
0.5463249504118303	0.6693574941676416	0.5346178112716284
0.3265745603122459	0.5935686392998034	0.6581724234208020
0.6883579779628068	0.6763625119208421	0.3652553527235508
0.2536987048696727	0.6377579221946941	0.5506297323627165
0.6142287048458239	0.7259823170532000	0.2561926522067043
0.3980632462394074	0.6996696549012061	0.3521608667408768

```
  0.5036013229163772  0.4401091353249443  0.5977752794893572
```

IWV-Al-T6-TS7

```
   Al     Si     O     C     H
   1.00000000000000
    11.6056394577026367    0.0835597589612007    8.7649669647216797
    -3.6825602054595947   11.3529796600341797   10.3282289505004883
    -0.2346490621566772    0.2412826269865036   19.0520343780517578
   Al   Si   O    C    H
     1    37    76    16    19
Direct
  0.5258392787353834  0.9355983053054493  0.4947672843198596
  0.1587837999946041  0.2600983271524985  0.0579473118367182
  0.1204160405665645  0.1167068132162790  0.0167489793566340
  0.1092759899904180  0.3369414157658279  0.2906144645562438
  0.2573918843660458  0.1600390826906244  0.5670807063304177
  0.1016719478785388  0.1281803480834875  0.7882746063346929
  0.3329503468686654  0.1235283509395699  0.2980211619448587
  0.0608879330170723  0.5763565835383639  0.1497871248800512
  0.0044124337551844  0.7807174300646472  0.1385900935666144
  0.2981451604187568  0.3141536277172214  0.1026511173604026
  0.5701901051621848  0.0755403099505139  0.2465057791503115
  0.7690831425011639  0.0082976341314238  0.1219149114097433
  0.3018342335041775  0.2976216101932181  0.3312440638157402
  0.8689656566516640  0.7796502233560697  0.9482654354724038
  0.8768777983499213  0.9056854417234351  0.0142947372322056
  0.9112576043741170  0.6951726767701990  0.7275305337853004
  0.7616855707148594  0.8718551786114219  0.4517877051536286
  0.8925222469630093  0.8851842129876587  0.2469439514013976
  0.6825219492734336  0.9153349828155857  0.7202916040864149
  0.9591004446612723  0.4545757833242051  0.8637346648508926
 -0.0009079774805444  0.2387248352280117  0.8917947879811748
  0.7268536818698266  0.7233688770167760  0.9075024858644296
  0.4486589884687248  0.9721567814215047  0.7608895046242281
  0.2274268381748392  0.0043939166326824  0.9136687389456064
  0.7230128721915990  0.7382056810150832  0.6821327318602833
  0.9314039209960345  0.5368075072928050  0.0815572941891298
  0.1222381510952044  0.5010864963073391  0.9007587692325763
  0.5089161437448846  0.1303823028222945  0.5163982032338000
  0.9044043040261706  0.1084123756156956  0.4685334088828361
  0.8826906106575182  0.1430458806952713  0.2940657189359370
  0.0705837153158037  0.9577608963969185  0.5225203937965467
  0.0971158949414863  0.9302763989059171  0.6849079126612607
  0.4665014789345932  0.5695037246937020  0.9049186753335015
  0.2960986705572937  0.7357006625694146  0.8794998524833320
  0.5247499642400805  0.5039374417302909  0.0679383951696713
  0.6856293370065053  0.3411887101418982  0.0963527423153335
  0.7212524977509964  0.3125362309588477  0.2561825721672929
  0.2517094196945516  0.7630987968284291  0.7245063907326660
  0.1834768961168934  0.1830414097817213  0.0226562327024830
  0.0213746508569251  0.2059590681589080  0.1750046771257358
  0.2495090081026808  0.2508406190922683  0.0848615323361158
  0.1858860961220153  0.3999060820526785  0.9474666244890364
  0.7788981120577063  0.0489630198615859  0.5999480791957547
  0.1721190669335662  0.1860050400734520  0.6537027645484630
  0.1949818442807743  0.0327272938292749  0.6278664566541996
  0.2738268561833053  0.2671419308947245  0.4433241225884151
  0.3828840816747379  0.1609971621741215  0.5443092834177470
  0.0263846313562038  0.8150473823469542  0.6057814625459806
```

-0.0002987485493446	0.6449584826915384	0.1962354538477072
0.1891964580436929	0.6680510155013727	0.0187457972579886
0.0816561313009090	0.4628079133614691	0.2433264581929616
0.9744574918108709	0.5289212592759412	0.1472493927353364
0.6026075405175125	0.6264760531491750	0.7826515015686509
0.6372211226844938	0.0211277376988762	0.1845783145428512
0.6407676864325833	0.2180460517238097	0.1417021555372250
0.4368155876601919	0.0601141278489336	0.2828435185812883
0.5710494541268167	0.0118810368209020	0.3540705001338453
0.5993237580368938	0.6185439855323419	0.0171389102145234
0.8195765191713998	0.8232124065950673	0.0186604929528170
0.9732997989288519	0.8915527041320696	0.8072820471364728
0.7546295404888100	0.7397962732753935	0.9693146733829314
0.9230658029218994	0.6704506502188897	0.9986477953962883
0.2065264596206913	0.0192540717799348	0.3993933968514360
0.8242369704976037	0.8261732106220206	0.3810986009132176
0.8372767682149266	0.0129272783524571	0.3508052043512707
0.7872119728186122	0.7953114332732891	0.5493370427995145
0.6220792027179852	0.8510053851416235	0.5160234797374942
0.9810050316220370	0.2322958102152427	0.4031006092415951
0.9868401301667068	0.3721757657812735	0.8332120930796313
0.8290689730034504	0.3955328406232297	0.9898559498590411
0.9662180805334305	0.5843232022102320	0.7567865173476678
0.0601592961655965	0.4667287036569439	0.8712187332516599
0.4134730997588603	0.4208956573259729	0.2101799497397882
0.3651719062145218	0.0071143696040911	0.8393525958964647
0.3652874698601943	0.8660657142105485	0.8081882911638468
0.5488330872940899	0.9278751652936058	0.7819450236251656
0.5109130485909283	0.0884580315468690	0.6209032487237383
0.4033376239455674	0.4388928314321294	0.9704819885017733
0.0253325101867395	0.5063158638794396	1.0018312243553413
0.5283956428596808	0.0336370297222980	0.5082544639599992
0.8001269104599983	0.4419778000845725	0.1801051807514277
0.0721709446120505	0.2111461222169047	0.9415172170399063
0.3809925732456391	0.8419475220389167	0.5994748831573713
0.1818055236269738	0.0649070564139794	0.8336999017626072
0.2243057700784484	0.6288951947716771	0.7774904340884181
0.9411963441733925	0.8322371055213590	0.0757884864630094
0.6159133431657765	0.2578547029340277	0.3907671993781254
0.8022662114080246	0.9322220881764612	0.2127941376378040
0.0109601524947240	-0.0026714530340756	0.1495073792576013
0.8731673672468563	0.1339336524681447	0.3885062031124786
0.8061266717775217	0.2272017413153536	0.2530900850633259
0.9759595653867850	0.0296764495113195	0.8771153638432957
0.0665528249188946	0.9822116030480738	0.5950765013166803
0.1460850929216010	0.8167035402827973	0.7130974110012539
0.1422617318815609	0.8669090283403397	0.0371036132216588
0.3888104722071102	0.6581343728543927	0.8720743097539583
0.2346056471018357	0.7498485719551206	0.8252178724169693
0.8684613507984791	0.1397949650778250	1.0003205281046768
0.6159458887306803	0.4297065604930799	0.0453964670870510
0.6614513752422707	0.3289899558755263	0.1984899771883150
0.2226589512664236	0.0814213568005554	0.9441772504491693
0.0743272962727419	0.2351929144749329	0.7875747773431028
0.9319970881490682	0.7815664785325417	0.2439979168024891
0.7682025254862352	0.9356602337117370	0.0889293125661078
0.9821047802439800	0.0147803233464486	0.4873216753311139
0.4699630258118220	0.5519698051771951	0.9979661100592339
0.1868794105145489	0.3390715180092498	0.1825034499547525
0.3359968241285521	0.1880909825783811	0.3377086170798141
0.8378253961837345	0.6887895298668615	0.8384738523844009

```
  0.6881326550846948  0.8396985608113615  0.6885059785473495
  0.1840998269387222  0.3160833840499345  0.3363963020250387
  0.3530357060425164  0.2258978426963535  0.1717954808879323
  0.8179396823096532  0.6881344899507464  0.7097665821285678
  0.7140108003375305  0.846331022656063   0.8121645838713597
  0.4090897804447858  0.5257462289951447  0.3593062728790441
  0.5026814325869551  0.6117470088257556  0.3030394899159178
  0.2994181641442827  0.4753601130703269  0.4712587829033509
  0.4876978714189862  0.6470256965391787  0.3586391472652622
  0.2829008275501816  0.5100078060916411  0.5275754461684687
  0.3779815910787602  0.5941480514330032  0.4743891281510288
  0.3613541789425426  0.6302136599128019  0.5386995666219485
  0.4313274622192305  0.5556955624717518  0.5857858833274524
  0.3760924449399227  0.4303222243293338  0.6882727684497107
  0.5503903928595566  0.6054227592909391  0.5291616195310490
  0.4400122090424120  0.3583398276033243  0.7261517951852214
  0.6131899484093692  0.5319002747092921  0.5677652131423376
  0.5600612111427614  0.4074061406076100  0.6654823542353043
  0.4040164162960921  0.7634072841885766  0.4602629453488355
  0.6292026759511355  0.3269761998478081  0.7045629297640940
  0.6426168861779501  0.2386399481790857  0.6905580495245880
  0.6915021558553349  0.2860433064691673  0.5964149942524428
  0.5545284860480038  0.1817872032906798  0.7438210790499753
  0.6922254999567492  0.1803888673394432  0.7210852523926179
  0.7179436770954016  0.3812988136876493  0.6529606324592536
  0.5815773562109423  0.2762857820277996  0.7996138675186698
  0.5872355409365854  0.6522885469045328  0.2151990682745715
  0.2269128317257084  0.4086091286942550  0.5144054066611650
  0.5609369634567009  0.7131170425222413  0.3155249991336442
  0.1972567475457578  0.4716727691799336  0.6140727102261043
  0.4360618163069890  0.5242127802966013  0.5254739990351645
  0.2831664490954846  0.3912443138377161  0.7352308156960856
  0.5925286245228663  0.7017348160155922  0.4553484157389047
  0.3961154426174051  0.262702179204986   0.8039942574792238
  0.7053386563389346  0.571621134584681   0.5220461539232736
  0.2659838988725453  0.5929159836569530  0.6160999236645893
  0.4978263539387842  0.8047010982745628  0.3795980818075522
  0.3497200338825324  0.8080342471246137  0.4303810150656084
  0.3959653376428507  0.7807894936084049  0.5104159810724778
  0.4211556863580406  0.4973472264985995  0.3162231405279767
```

IWV-Al-T6-I4^{+}

```
   Al     Si     O     C     H
   1.00000000000000
    11.6056394577026367    0.0835597589612007    8.7649669647216797
    -3.6825602054595947   11.3529796600341797   10.3282289505004883
    -0.2346490621566772    0.241282269865036    19.0520343780517578
   Al   Si   O    C    H
     1    37    76    16    19
Direct
  0.5290779508518053  0.8986790651420217  0.4745884095389155
  0.1609766135589206  0.2720816910480724  0.0238231162020517
  0.1555941018307901  0.1407370135988272  0.9608397681319217
  0.1061421164949426  0.3398855823682760  0.2729405648013196
  0.2604072680992847  0.1608476005456643  0.5362444414507028
  0.1268738137397885  0.1516910329823379  0.7396983528563421
  0.3397057417467740  0.126493962240354   0.2675677214204182
  0.0664826945068313  0.5796574475151587  0.1271121310062117
  0.0308866933965039  0.8033094189763635  0.0864459017265340
```

0.2912328433975769	0.3213533707730457	0.0819954761216622
0.5743165257925931	0.0711057054341455	0.2219681184268038
0.7975657729584783	0.0341241384966788	0.0733406351026857
0.3075731186071485	0.3014873520163241	0.3006464388576886
0.8714300037231260	0.7757088063761360	0.9276180431372518
0.9044586918817700	0.9282068746907914	0.9628280262357604
0.9151042361541006	0.6979055556484509	0.6973154726633209
0.7807137432477753	0.8690650548023601	0.4170990997959916
0.9218519476993883	0.9125553454163158	0.1930924023893730
0.6873463734187178	0.9101672547907013	0.6883115108520811
0.9529209581029464	0.4541154695813143	0.8397944360832075
0.0324098679547232	0.2623965291749914	0.8381395949628763
0.7323921108337477	0.7268412565077286	0.8782268907952420
0.4555581869022938	0.9744077014113351	0.7206478526301480
0.2543112160390670	0.0251190555024647	0.8655697162916428
0.7307004479604616	0.7296471069091233	0.6535553399279340
0.9115151833521367	0.5257061669276830	0.0883049705886120
0.0899397107980992	0.5006171011809828	0.8972485284613774
0.5061555668995668	0.1020524526248557	0.4868772306776262
0.9144693896869389	0.1161048349492907	0.4350887168398710
0.8900961926279416	0.1433485180616784	0.2659023501370054
0.0773026917424575	0.9586723323639018	0.4925659581498339
0.1064925988963301	0.9300873169688926	0.6544151311305912
0.4750745677889211	0.5787595835376103	0.8736884024779931
0.2979662122072958	0.7352085420560238	0.8512802551010547
0.5308142726782046	0.5072973463278412	0.0391659646131003
0.6894588546601100	0.3415564266454635	0.0677128409254418
0.7288969993363646	0.3102383170417271	0.2249869308615317
0.2601397848016377	0.7573456660825014	0.6947841474121252
0.2099492259422085	0.2099756832473170	0.9703599851474252
0.0254430966773099	0.1929615386000050	0.1465187720189188
0.2492193784086266	0.2727512061299024	0.0494173842878253
0.1695426129195120	0.4103778558831954	0.9265571712224233
0.7875904240914878	0.0416616251233225	0.5694776422582963
0.2083560291648550	0.2061993419141962	0.6052602740737492
0.1554992152567376	0.0529587284537828	0.6081047607090814
0.2960991555924313	0.2741248740119706	0.4042010535344059
0.3716715869318834	0.1135076712577071	0.5310420682519024
0.0238095740492011	0.8205839559978314	0.5672585464185779
0.0235580336334405	0.6645760213983377	0.1549843107363530
0.1926359008168782	0.6558161175593048	0.9934986087379397
0.0856540768217687	0.4687115666419265	0.2242200602986625
0.9643341559411249	0.5277862380918117	0.1424310878109407
0.6066038018894809	0.6220698022117341	0.7524830586562322
0.6684766852140257	0.0564677860605331	0.1260049075713207
0.6311180552930170	0.1997905080523951	0.1646187609973949
0.4545880196401415	0.0801893944761795	0.2293914759856163
0.5476081092881908	0.9660462720129271	0.3470592863192132
0.6144134019875537	0.6094049500670461	-0.0037759054929188
0.8429181560561937	0.8528075169234201	0.9634117497699446
0.9985123199715848	0.8426830325790523	0.7967842299016756
0.7651216625790954	0.7594899996973469	0.9271009125700495
0.8776835439878913	0.6477474149478163	0.0233849443754216
0.2189183952203331	0.0120226787800828	0.3798637726512366
0.8677225772853250	0.8483015787325383	0.3259170155514364
0.8326012360751049	0.0067868995965636	0.3435510585768596
0.7964464824176242	0.7811816441288681	0.5222826440390013
0.6451578637110653	0.8351599185459121	0.4709491271173718
0.9753467915327660	0.2389676174388584	0.3832941168378261
0.0191244557327454	0.3939416706517412	0.7850918327536830
0.8111451880703707	0.3827226659089367	0.9430805520113849

0.9764657158918161	0.5917514304630767	0.7294313811528310
0.0045371240222346	0.4466542925218318	0.8988221747727477
0.4074219515392039	0.4315070005680524	0.1749516861703339
0.3895136194085808	0.0285559404949623	0.7822069805970459
0.3536890778247070	0.8670637515401227	0.7869855290459696
0.5541850724577130	0.9242620247086770	0.7426407222716339
0.5208336142929474	0.0771496908938375	0.5782384754966362
0.4019487041528209	0.4444080013784512	0.9547794507979537
0.0096983382633335	0.5071872118478766	0.9984024989134465
0.5286419101832972	0.9991665462498506	0.4867055570861305
0.7896829293259936	0.4139742878376518	0.1976058984032697
0.1149779437963780	0.2350724860909645	0.8783677660036078
0.3965538888451479	0.7796351275366662	0.6002260780725930
0.1978005611717644	0.0886733049802541	0.7954164371245249
0.1771932826085027	0.6319739950128306	0.7661262031800391
0.9715568835512429	0.8487088163131221	0.0207833043135290
0.5987071687678991	0.2343703574917159	0.3548520592861586
0.8224331696432876	0.9590201640042066	0.1710438800285595
0.0409542471532712	0.0244536524080831	0.0926048695598259
0.8928149856865129	0.1520063430460932	0.3450676182449640
0.8142476262604226	0.2250140709344879	0.2220101446071779
0.0028838688880018	0.0525191372369787	0.8254713743084565
0.0565503629222840	0.9639084195774392	0.5834020587668607
0.2084371358374804	0.8647977362854822	0.6310499993345542
0.1690776307279855	0.8868503744787029	0.9883840727179056
0.4026019735477856	0.6705839778190920	0.8346098876702970
0.2391117745106060	0.7448715466371945	0.7968277682879545
0.9015643526943826	0.1624068277730742	0.9525376277922146
0.6027331279385072	0.4145754626954534	0.0341508563395536
0.7169670024483978	0.3744050437587051	0.1200337827410709
0.2583057984678122	0.0971592109349377	0.8981272994578886
0.0964055746083214	0.2610887121407240	0.7340688612923354
0.9540050263750521	0.8100641819088146	0.1880219915188955
0.8022233011081253	0.9588851306553021	0.0407117291456872
0.0062120651993375	0.0360015058008267	0.4391219084185962
0.4958526580594877	0.5764478263425066	0.9499118744451791
0.1808365268936733	0.3449830752159611	0.1626799190927972
0.3453265033731091	0.1943027491720541	0.3033493434565241
0.8455194692414372	0.6990591072948963	0.8025980814563232
0.7019413200192974	0.8361498880623197	0.6523351563543394
0.1807268984882676	0.3078115837707744	0.3219503804421589
0.3402987526642369	0.2228769600867056	0.1524816876775868
0.8185780573842414	0.6735229232469191	0.6902332821699413
0.7048324393131451	0.8387222091322093	0.7900695503182962
0.6297907107851368	0.4489148800766656	0.7115642493430907
0.5164513472926539	0.4633512608058989	0.7388148334556804
0.7319328037883541	0.5118534927394434	0.5909547062553020
0.5049478482581247	0.5391634391076046	0.6501521090095974
0.7210516566992896	0.5839332957870836	0.5015958603202102
0.6069638868494760	0.6041838680254291	0.5246348241994326
0.5818631396204357	0.5948394595836979	0.4584666887764078
0.4895135248717015	0.6529879408512710	0.4454793649420631
0.5274689864132723	0.7611704898989554	0.3318548588399572
0.3665082656488203	0.6018462449819427	0.5422250907607096
0.4456131962429979	0.8182363971757558	0.3149075079709347
0.2845893162828701	0.6596235521447879	0.5259254669686078
0.3238776626711855	0.7682975333155446	0.4124725553465539
0.6373227854282251	0.3673477540533390	0.8118747893750724
0.5518552955466320	0.4643404068797815	0.5208219470633649
0.7619723374325571	0.3734958904321144	0.7787833088919042
0.7541701934287435	0.3122400109693250	0.8605695380911922

```
  0.8162665509557279  0.3481215670608930  0.7275714129596611
  0.8105278700522476  0.4638258731690458  0.7254079258310104
  0.6218559651685570  0.4254765353589534  0.5272427014245415
  0.4659844739399748  0.4100729198760184  0.6118413548059012
  0.5444190437003809  0.4589120934663439  0.4697318860059412
  0.5887038604098532  0.2758314045314456  0.8664231134483622
  0.5811114858844866  0.3831008417821402  0.8716768565291503
  0.4393424308678883  0.4155010802796236  0.8316480260991350
  0.8189550045155792  0.5018612503935619  0.5700829632106829
  0.4194681240036799  0.5557524053978306  0.6705855159531334
  0.7991918953279592  0.6304185394073355  0.4090450156741594
  0.6209409223200932  0.7009363793366382  0.4777865301852215
  0.6225190380596871  0.8002750125144160  0.2554776353574273
  0.3335779169771180  0.5158715344036655  0.6311561462042008
  0.4774528298812628  0.9024762554739993  0.2257412459997034
  0.1896111450127113  0.6203123487494117  0.6016294126369839
  0.2596149415378384  0.8138449197080181  0.4007051369962001
  0.6681738567322441  0.6461377137866574  0.3677612852566545
```

IWV-Al-T6-TS8

```
   Al     Si     O     C     H
   1.00000000000000
    11.6056394577026367    0.0835597589612007    8.7649669647216797
    -3.6825602054595947   11.3529796600341797   10.3282289505004883
    -0.2346490621566772    0.241282626986503 6  19.0520343780517578
   Al   Si   O    C    H
     1    37    76    16    19
Direct
  0.5285499714311001  0.9233383388290254  0.4735285652577340
  0.1599000654481945  0.2533702544680565  0.0362726969846896
  0.1247519257030286  0.1104852669312566  0.9938617914232426
  0.1032336519706538  0.3252753618795580  0.2776067227416882
  0.2575677347604179  0.1542719430189730  0.5466926750179488
  0.1046379217486593  0.1235762273890135  0.7655080378640143
  0.3328574775848194  0.1127846958472212  0.2801912293865295
  0.0593001972820226  0.5654053237207187  0.1313396723956424
  0.0114269406464614  0.7758404966505007  0.1122069139351793
  0.2927710827601132  0.3025931417130237  0.0896043366846423
  0.5727690503166651  0.0665149803066066  0.2239545004641005
  0.7731732721297437  0.0019806198014710  0.0983336658120192
  0.2983002077710045  0.2877916210034965  0.3135517222397087
  0.8697965032005198  0.7685092620710244  0.9295033443029852
  0.8820680499212313  0.8992429128463837  0.9902193054557498
  0.9105051267121105  0.6855101973135447  0.7088774695139459
  0.7681077739306256  0.8599533262875158  0.4310002839086493
  0.9004239928977157  0.8804647053240451  0.2213785982240232
  0.6811348996657455  0.9001583007650377  0.7012719151695096
  0.9571405546015197  0.4468465980029583  0.8451712144398635
 -0.0013092296926588  0.2313656332783218  0.8724148204954449
  0.7262423918824086  0.7115921963014517  0.8897591142289690
  0.4480248292815454  0.9601844547352988  0.7414403145859072
  0.2325305425064189  0.0003074230804646  0.8881022339614635
  0.7232070696568296  0.7215013410496246  0.6646954118882344
  0.9292060705006223  0.5231641029507762  0.0674412429915773
  0.1201954914391324  0.4911466100078158  0.8842782204015256
  0.5055233001340029  0.1167482273130270  0.4985756616248728
  0.9028284920736982  0.1019793737363346  0.4477499991466622
  0.8844505963173540  0.1323259295232590  0.2738778077715969
  0.0698633918442134  0.9514621858755599  0.5021531507037964
```

0.1012124346918083	0.9232857415104587	0.6615637313741991
0.4658905691832010	0.5619423290508719	0.8854069752583765
0.2956746168468055	0.7264070492250246	0.8567927624323999
0.5208451202147002	0.4956322236011027	0.0494345911035451
0.6811965255166624	0.3323160050213451	0.0756297697757674
0.7204263604529110	0.2963389748371467	0.2371635474901365
0.2574008062872801	0.7540893766216815	0.6972233227510733
0.1877061476711755	0.1786781571758740	-0.0016204267366335
0.0210735000221378	0.2013321029851561	0.1505378026526320
0.2470538542641653	0.2427692882858756	0.0673087873612592
0.1895705489140352	0.3942854694440688	0.9250879122549083
0.7769648042237514	0.0339508592696997	0.5812025262189793
0.1734660665501533	0.1828806297582707	0.6307552819544917
0.1943496793008833	0.0271114036794481	0.6073066869129443
0.2765127005504567	0.2596668395066528	0.4217784649636217
0.3814893714727851	0.1521705331488876	0.5272134308707872
0.0209321055062226	0.8093474918370458	0.5854033376048383
0.0062529300131434	0.6395521534622836	0.1718194155628937
0.1844980113009133	0.6526370314860651	0.9964557088169139
0.0840844716288126	0.4552402667245415	0.2229585746674555
0.9646396797376667	0.5137528140978441	0.1388165934108165
0.6014516213130923	0.6112045915627434	0.7655863456133976
0.6382895216999422	0.0084766815839011	0.1657880767574104
0.6432007598277771	0.2082606144180448	0.1187629650023559
0.4377544752296791	0.0500687015709634	0.2633650580205710
0.5723857413683117	0.0003738828961646	0.3329375059580502
0.6008728056038009	0.6035183035920112	0.0026277546440513
0.8244280032440355	0.8180164347541432	0.9938803005133214
0.9797173562696605	0.8744887665560324	0.7890959577667389
0.7568255777309058	0.7343335372775039	0.9459478736432854
0.9160059991937050	0.6545654165894108	0.9874157966024204
0.2083606911534337	0.0045494019359993	0.3841460458176342
0.8290799130428684	0.8184703691095022	0.3570879033263032
0.8474815273045642	0.9984048470556180	0.3342832530672994
0.7884725256134339	0.7758951499937464	0.5326284717868530
0.6296528451847331	0.8459071328143091	0.4907708735777774
0.9714691296477990	0.2259508110357557	0.3886380843240336
0.9879063857333281	0.3653789231299935	0.8136178187375971
0.8223179491489035	0.3943755316837331	0.9636107981863866
0.9738032668038459	0.5800504912329374	0.7333464328026300
0.0473088059231015	0.4500314685557447	0.8673439592999446
0.4048426350622324	0.4138741005604259	0.1894897097546191
0.3699623376806634	0.0017356057845880	0.8142128548939769
0.3578434110537115	0.8551559515960057	0.7932832010510820
0.5467996451122124	0.9108203600437743	0.7636263252832279
0.5135367372022565	0.0733584592364348	0.6001059858151648
0.3967004147605830	0.4289499460630092	0.9603426016093313
0.0336615248372053	0.5018014425553710	0.9820816923424637
0.5144058873642469	0.0175541270843622	0.4948628640614277
0.8034623073354864	0.4233110417918598	0.1609549940989504
0.0662132730920765	0.1989752227837257	0.9281755741026693
0.3902195260013473	0.8181936526751371	0.5756733067459588
0.1858751668487172	0.0590549490469621	0.8092174817229483
0.2207068092045256	0.6189516887257261	0.7544714680327995
0.9484248822009089	0.8256070004520806	0.0494737204631809
0.6151628062109776	0.2416369249897130	0.3715053731392158
0.8135900074427345	0.9338589159210172	0.1829809819093819
0.0203526797097302	0.9881410508499678	0.1287358040023625
0.8757244417932617	0.1313821819550073	0.3635332840623156
0.8003569935586676	0.2061849336282807	0.2377462058287346
0.9791944252656392	0.0250250923260707	0.8538912995674910

0.0647720824503226	0.9748556167131585	0.5757398536883641
0.1598523427559511	0.8174015491948486	0.6763603592371101
0.1487360853911130	0.8620622797104484	0.0115659716675179
0.3919033522530095	0.6524915189511200	0.8466751132467186
0.2424039428826475	0.7451798484632038	0.7943015841491048
0.8659870552045646	0.1367759592995731	0.9759765952795750
0.6043109273856047	0.4142789125770072	0.0322179141516996
0.6605841301374965	0.3170685828292080	0.1779204965736605
0.2261712886460335	0.0782373923536182	0.9180248148424311
0.0776229954320547	0.2289280797884347	0.7673811712101222
0.9379855708003634	0.7774355933450539	0.2172526775536874
0.7740449819452558	0.9279178691503913	0.0665318989602780
0.9892211903126065	0.0160573628025890	0.4582214402448754
0.4736193037194383	0.5544427009700278	0.9713940663225985
0.1792958843033391	0.3219707668799600	0.1732336603663604
0.3354235968882396	0.1790633186278151	0.3183740354562750
0.8376379286658637	0.6770169134008298	0.8207224370345230
0.6903627965037294	0.8258407282067290	0.6670852384620976
0.1759663905221419	0.2991188541534341	0.3267538944552283
0.3497127347604246	0.2127646387918790	0.1558353792936368
0.8165742736128185	0.6711531184162701	0.6948820685937944
0.7113585550914916	0.8311856900576839	0.7939820267482823
0.2317707258137967	0.6302343219483890	0.5370738846817626
0.2686706098255357	0.5271463571491071	0.5857418291911827
0.2971824564844910	0.7383869786747836	0.4765919347223587
0.3716986908351833	0.5322054760602061	0.5728011785846079
0.4046078679300232	0.7463795236241009	0.4596607850146724
0.4447494531453283	0.6427543016222445	0.5077950696893138
0.5729739350423509	0.6582598114347631	0.4633680970890751
0.5875142999836830	0.5610949350806573	0.5589614971126831
0.6964736514423783	0.5345835209098061	0.5274116154204960
0.4947206744157696	0.4982142847411810	0.6802813330292772
0.7116460776382543	0.4484318878208924	0.6143620238058063
0.5097811377790019	0.4105971811959373	0.7669995765410427
0.6184337797862414	0.3844446360676659	0.7359577426833416
0.6415922461611959	0.6662167239814347	0.3579306742853677
0.6356931393360633	0.2933657429789104	0.8297025834274310
0.7046421649871423	0.3512235198629545	0.8326553936556667
0.2154315966521237	0.4435170569481125	0.6323164800468515
0.2689953666940761	0.8180934833541803	0.4400059185999480
0.4022443039636059	0.4539987637038886	0.6082013141908005
0.4600066869390663	0.8302796155763286	0.4104994717779625
0.7714570598385396	0.5820685389074646	0.4340616152945631
0.4087414339036586	0.5165263715581628	0.7092890971466335
0.7975804841813039	0.4299808851713613	0.5870594922320223
0.4357101921159085	0.3618226466408218	0.8607186963132839
0.6064390906862088	0.7448872114671422	0.4276451028359616
0.6126938027392427	0.5800024741031325	0.3911183820898153
0.6258758194045596	0.7349900341713654	0.2928243282636760
0.7380444138703008	0.6892642086557748	0.3110300975790680
0.1498593398190353	0.6235382233537466	0.5488480353205203
0.3826545837903122	0.6707111516565878	0.5693422292462520
0.6562092931538346	0.4042635090652547	0.8530952058802076
0.7936649258027677	0.4114129017328005	0.7451198932343865
0.7167544404399722	0.2829397267449514	0.9010091128620477
0.5475214559303775	0.2327662820939667	0.9183915213946566
0.6846651730561474	0.2384914384769338	0.8119532141503935

IWV-Al-T6-I2^{+}

```
   Al     Si     O     C     H
   1.00000000000000
    11.6056394577026367    0.0835597589612007    8.7649669647216797
    -3.6825602054595947   11.3529796600341797   10.3282289505004883
    -0.2346490621566772    0.2412826269865036   19.0520343780517578
   Al   Si   O    C    H
     1    37    76    16    19
Direct
  0.5243922334654026  0.9142256350875372  0.4817871815097829
  0.1577552564149874  0.2424790483434531  0.0462669797249664
  0.1198601677554019  0.0985066153986602  0.0048257987100325
  0.1062775075835894  0.3170624625986064  0.2804616038051465
  0.2535768468513083  0.1434452796886813  0.5560681618234242
  0.1011972225673734  0.1122769582598177  0.7748487876356358
  0.3326801519954199  0.1040747482701135  0.2859147462987614
  0.0576872292723090  0.5564152326211785  0.1381479718956538
  0.0061487528683021  0.7645555000532435  0.1230275932964178
  0.2975821024550984  0.2940477329167345  0.0919006551529634
  0.5706864721485707  0.0552217900922210  0.2339517176082088
  0.7691864851178627  0.9906321443846589  0.1077140337296294
  0.2974819360297511  0.2777849078749050  0.3208741406903006
  0.8664226714595935  0.7591088642667739  0.9368004500435179
  0.8779551527899813  0.8870164790573898  0.0003938894882384
  0.9112324794489125  0.6766982186195849  0.7153134837140376
  0.7626048057687744  0.8529250653762296  0.4390693456898488
  0.8952011160559324  0.8682720193792209  0.2323437785264402
  0.6807038211306776  0.8941018972983978  0.7091754030910468
  0.9571972454119768  0.4364629335148652  0.8528678542733854
  0.9945452356881638  0.2200248683396647  0.8819733057370709
  0.7237282424168568  0.7020429699355379  0.8974708795109988
  0.4454961497704339  0.9506472117913329  0.7504207059884567
  0.2286933471702539  0.9904236418835781  0.8976991840021382
  0.7240386721381006  0.7172210054793836  0.6697359803271432
  0.9262910651576886  0.5153517645801399  0.0740620401813862
  0.1183136632785110  0.4809014511770095  0.8930021228623315
  0.5031248011330253  0.1081860365802944  0.5062630087806979
  0.9015547726590254  0.0901981734335168  0.4566486527919419
  0.8815392768064899  0.1239570795813974  0.2823137278034431
  0.0699994172089403  0.9419569597632419  0.5082676341728054
  0.0958823443476887  0.9135969799302718  0.6706831458889726
  0.4658009361384723  0.5515582569110814  0.8913422181988139
  0.2946900981319556  0.7160656672204107  0.8647077425942770
  0.5200976934159811  0.4840919717867238  0.0565697446189946
  0.6813513549820697  0.3211994184037051  0.0837979866608678
  0.7179307120956557  0.2905368554445895  0.2444798980166416
  0.2530473333197901  0.7457500344655078  0.7061104014428279
  0.1814296747006728  0.1671359817913029  0.0097277743016092
  0.0202230284108359  0.1899047412093775  0.1631334869620917
  0.2477149051360812  0.2308981944507738  0.0748633186938414
  0.1859368545845407  0.3831536223757802  0.9364585079647447
  0.7769098506913149  0.0280113159566954  0.5896061788409148
  0.1709981682061688  0.1743713961417901  0.6386410149318098
  0.1881803815894853  0.0158129099383836  0.6197635108927549
  0.2727083112530704  0.2494649970589697  0.4307320108940422
  0.3788280474562359  0.1443500659413875  0.5341789133673726
  0.0231109503280805  0.7990289306241137  0.5941540247615360
  0.0056882956896272  0.6293774789619763  0.1800315776505297
  0.1837874725156589  0.6434291175011311  0.0044363364724487
  0.0818209480775169  0.4447005441005745  0.2297446998832535
  0.9626577350944048  0.5068100905762730  0.1446001260746269
  0.6027593752769909  0.6061065771119156  0.7709291274606126
```

0.6352150461648248	0.9978982778129944	0.1750400848522349
0.6406472885700794	0.1985245382318186	0.1265808689918066
0.4355718247550209	0.0395234901134428	0.2737225671395163
0.5766977172512229	0.9967052467230173	0.3374100438466380
0.5962884558456224	0.5957424625832760	0.0088985090775189
0.8201172874050383	0.8066322103464283	0.0031712839576685
0.9696941346524426	0.8684893436205459	0.7949729536304944
0.7501984300571440	0.7175136015835988	0.9607109015128721
0.9187929248093604	0.6490916295109437	0.9895543728450131
0.2056830659723239	0.0012482207763914	0.3857543336900657
0.8233620930790303	0.8086225748723899	0.3673329634176123
0.8407501834287472	0.9931730147214222	0.3383524811866570
0.7881647790709154	0.7743150130568071	0.5369788507134230
0.6231813158423241	0.8350948586392095	0.5040606205788676
0.9762518107729916	0.2150219754150741	0.3915368640608188
0.9859834842462180	0.3547585090386924	0.8215363894283894
0.8233513191676031	0.3817287299242894	0.9739044120457547
0.9709751447080700	0.5688960446985426	0.7426389455350715
0.0498320176526205	0.4402402930714647	0.8722233512597556
0.4067649077213258	0.4030384586137609	0.1980258129679768
0.3657699078867022	0.9903316121043146	0.8248070268462903
0.3589429049043795	0.8436301791166239	0.8020808325881505
0.5467492322124387	0.9063338727807275	0.7699032795507619
0.5082272996411210	0.0650050991975367	0.6095677378945668
0.4012750387278256	0.4198935329079938	0.9601368003925670
0.0251299986021720	0.4862612478418424	0.9947967808317855
0.5148841714538601	0.0101057209178189	0.5013701352232104
0.7957843800625733	0.4200922633947314	0.1695070870509232
0.0615400705830376	0.1858463028467698	0.9384925653890097
0.3825538528595773	0.8180344475840724	0.5814185206392803
0.1850430059386347	0.0510080073862968	0.8158489738225667
0.2164759007765948	0.6095554565211817	0.7669206986205246
0.9432192629090193	0.8119007220156343	0.0605723646208099
0.6114252507371141	0.2350667866340234	0.3795377899695503
0.8116673696641058	0.9230379301705698	0.1904308038970330
0.0168769686114999	0.9748474133713816	0.1391339686256211
0.8683306692316752	0.1161513674727501	0.3777173507757234
0.8024857552314858	0.2049309976823934	0.2423984851779657
0.9774044680664166	0.0118028880907241	0.8633418884291558
0.0672732376460082	0.9690812009285804	0.5785938821729002
0.1496069913483005	0.8040955106671738	0.6928923635460827
0.1428954054528301	0.8529850308121499	0.0213481564863449
0.3896314790014356	0.6407821215094074	0.8558090278960124
0.2402842291096819	0.7372810912953989	0.8026222227030240
0.8624096598645079	0.1254568723266229	0.9842437897088464
0.6074452551197187	0.4066180914619988	0.0370984161770294
0.6584255809536974	0.3082775792473203	0.1860540007433858
0.2229839843896049	0.0680858961599649	0.9274526988268548
0.0729898037410452	0.2177077021786556	0.7764295112309511
0.9317459223348288	0.7640067782216964	0.2295002758292874
0.7702297374243908	0.9164807435336058	0.0756354981846051
0.9826057434298175	0.9997541992870923	0.4715871349484255
0.4684151137690135	0.5379231440206872	0.9831811821625764
0.1861599504053692	0.3167676975263143	0.1741765920518011
0.3328350674241507	0.1692695338050740	0.3263452167073037
0.8348218607366019	0.6683755445599395	0.8278730812576158
0.6898989396087353	0.8191033916997545	0.6758623167910436
0.1773195894645756	0.2949794236583237	0.3301812459944075
0.3550383051029120	0.2069812523643536	0.1585610180091833
0.8187366911323157	0.6665914054988111	0.6979296843318181
0.7091122578755193	0.8243171030285330	0.8035571615528672

```
  0.6760637423439256  0.3942462237387890  0.4839029916455124
  0.6531955534196546  0.5006304759591390  0.4533866540006969
  0.5852064397809646  0.3051062899833138  0.5402077604867388
  0.5429047199875913  0.5181093047831261  0.4778174204098249
  0.4752890566294848  0.3218235517694388  0.5651922107858366
  0.4525488867435635  0.4286704114391002  0.5343899830246894
  0.3354892288175944  0.4464672847669914  0.5543760820923357
  0.3358022042304171  0.4765522503966788  0.4609616588099499
  0.2312041803372339  0.4769524470244475  0.4737369888202654
  0.4482551905686696  0.5067485336394926  0.3530291267934033
  0.2351428215065954  0.5125125056202816  0.3821034790603173
  0.4474285523907435  0.5457932070312203  0.2588740953807437
  0.3425961563068343  0.5486009756889845  0.2740695368507038
  0.7933781120330721  0.3728939589273444  0.4597569716050257
  0.2966268500842076  0.5399455247320482  0.5635890330560339
  0.7795819270264716  0.2723957433131407  0.5752047950057265
  0.8664172175176591  0.2574796005015769  0.5540279292692099
  0.7442718739665443  0.2944002287536947  0.6290070605524010
  0.7175652632318260  0.1881735988869705  0.6303725315417886
  0.2958006091118554  0.5171096312769695  0.6324913886822200
  0.3587923366564254  0.6300948281400064  0.4782110990067218
  0.2064565035360062  0.5406278129438450  0.5888306648101220
  0.8571034957973922  0.4559472502459305  0.4045790126325376
  0.8318024845734987  0.3490117216777157  0.4070132784501078
  0.7224667276460027  0.5709085023103900  0.4099909674403184
  0.6010062557743174  0.2217404612688247  0.5642077044423334
  0.5276871327472316  0.6018899354313697  0.4532727690036200
  0.4062693115562164  0.2509459649956471  0.6089327337168808
  0.5229688020604241  0.5661982440321336  0.3177600338118295
  0.1461691587558192  0.4502932594724747  0.5551374170873647
  0.4799164398168847  0.4259434835507035  0.3840413529773604
  0.1516364165975075  0.5126726848626578  0.3942933460330296
  0.5312021467045328  0.5697472954970769  0.1781759621543942
  0.3406690228754444  0.5755091544803769  0.2055517051847696
  0.2633131856562302  0.3610265721892189  0.6402591163736131
```

IWV-Al-T6-TS9

```
   Al     Si     O     C     H
   1.00000000000000
    11.6056394577026367    0.0835597589612007    8.7649669647216797
    -3.6825602054595947   11.3529796600341797   10.3282289505004883
    -0.2346490621566772    0.2412826269865036   19.0520343780517578
   Al   Si   O    C    H
     1    37    76    16    19
Direct
  0.5296377175027507  0.9226355671036655  0.4851332317406301
  0.1626303965171822  0.2499400950901758  0.0456724272454498
  0.1238395416208761  0.1049485503091953  0.0061807478353729
  0.1093911300387011  0.3244501136103854  0.2821410194166084
  0.2598308127775872  0.1513225069055831  0.5552150767913147
  0.1059838866528181  0.1174301297069694  0.7769035494783672
  0.3379719478128190  0.1150278962289071  0.2852401787160714
  0.0656762144548365  0.5652660377584755  0.1387931562841422
  0.0097877640313750  0.7711479271421808  0.1255232603361133
  0.2984269281082372  0.3039860916490145  0.0928367763705322
  0.5752853597736637  0.0623876151694532  0.2357499017783883
  0.7737516690993678  0.9978617107961045  0.1088816308540519
  0.3033666862787884  0.2868077036038839  0.3207792773684907
  0.8726745225511733  0.7679127763233603  0.9365460049962047
```

0.8818011672415247	0.8938909284922048	0.0024633554331923
0.9179828793989413	0.6843076582397893	0.7136304478809703
0.7679368497770680	0.8600027591950523	0.4401053829008192
0.9000137377404243	0.8756322106779072	0.2334449162704267
0.6848235931581902	0.9000327987407934	0.7095791935674235
0.9624873435574860	0.4440914347045268	0.8524034836625755
0.9989892887672021	0.2260939021364913	0.8831754894502954
0.7313557943054475	0.7103185015344838	0.8955972798270398
0.4503845484747741	0.9598036758810050	0.7495488291136062
0.2325641801724367	0.9954774991742599	0.8998484500389798
0.7288496755386330	0.7225023958323972	0.6709179874587523
0.9349518086669593	0.5250315604848285	0.0718454024661666
0.1241306137918044	0.4883950447286818	0.8921222549522678
0.5107390890947948	0.1196193652273344	0.5050495702168393
0.9070026326861409	0.0974824677967465	0.4572227498156864
0.8867930705446533	0.1318558304175689	0.2822671179784340
0.0757265993634754	0.9486448346631993	0.5088485873522374
0.1017952441577318	0.9201817278469093	0.6722814644471169
0.4709267302968121	0.5593944374743604	0.8920880832448895
0.2996559635582823	0.7237332549058906	0.8677575690183206
0.5264018789390639	0.4899533109166726	0.0580099752786345
0.6881624475217932	0.3286575101871354	0.0858320170568747
0.7244742302728369	0.3003752416994484	0.2454734011293493
0.2551558123708330	0.7499998156990175	0.7141187911839263
0.1879967031163573	0.1745713352436375	0.0087693401389756
0.0242910172462809	0.1979682364689396	0.1607972315810763
0.2507953523125827	0.2387595356575730	0.0759672350093050
0.1923509946323707	0.3907392066904585	0.9345803504038639
0.7811796930173828	0.0338080655430508	0.5897027433081411
0.1752505317155984	0.1782807773403850	0.6412042617162909
0.1973233393112621	0.0240063311639887	0.6159626194195449
0.2755237493918248	0.2579133884466935	0.4319834540413474
0.3854919326889961	0.1521147705624556	0.5321682554564053
0.0312668851113537	0.8063237975573755	0.5896921445781341
0.0076839838488280	0.6356753871829008	0.1831616438266001
0.1954439913033141	0.6524937399709778	0.0084853960405775
0.0830511115807922	0.4507693256137229	0.2350780567471944
0.9763912658701461	0.5194053333428407	0.1376392053250292
0.6075857570567590	0.6114104501210260	0.7718627237553807
0.6396293233264628	0.0043615081978465	0.1774783102448768
0.6530907491560707	0.2029003900542065	0.1307266361231954
0.4445045493946171	0.0555638903933236	0.2650625642057475
0.5698885847472293	-0.0041403944575122	0.3471959636077038
0.6072351210935441	0.6013839354176781	0.0061386360312735
0.8242688357640808	0.8123728918826111	0.0059936521372543
0.9782015608613885	0.8789941574075009	0.7957914132513542
0.7595328199469052	0.7290416328093402	0.9557716089893264
0.9251769547460709	0.6576193720273706	0.9885800024583711
0.2126801680829569	0.0084571834351000	0.3870840410044776
0.8281099111046811	0.8158058143647747	0.3684500142235851
0.8457274016463573	1.0002191056953171	0.3398551793309639
0.7939725235358446	0.7799222830015413	0.5377287267525738
0.6286245668550043	0.8423680902099439	0.5050249757255953
0.9797079125802010	0.2215730044288585	0.3947557147189930
0.9893555657268504	0.3604455779526312	0.8236779395795789
0.8295623036790013	0.3931248668554271	0.9727548639708286
0.9781570420751983	0.5767722091364289	0.7408145228183209
0.0580765951333349	0.4495551782758693	0.8683769392699413
0.4123525138873148	0.4115242146600327	0.1985805093780716
0.3696544071539203	0.9949820235392304	0.8281496511441235
0.3637129706395811	0.8561288688696826	0.7966794673394271

0.5507330473662603	0.9119266590459016	0.7697181403081168
0.5130470244988100	0.0772215453221530	0.6095418695739562
0.4021938677046047	0.4294992910644641	0.9604785544327853
0.0304359599236863	0.4939407372161797	0.9933082834786362
0.5289122298856557	0.0232001531377948	0.4968522926168982
0.8046996768086597	0.4281679109452906	0.1710736167188450
0.0620608639072383	0.1908958346211493	0.9435898873206486
0.3867633069952980	0.8245774819562043	0.5927649303840441
0.1880798245836342	0.0523710175885073	0.8193545201097694
0.2237390026210258	0.6169531856057027	0.7661996241538449
0.9479174780314341	0.8195411922630372	0.0619138809104672
0.6186298757527896	0.2467862171297319	0.3796532720414731
0.8180342693513268	0.9332767978068204	0.1899034106391835
0.0229547124203345	0.9804154499876927	0.1412580546181349
0.8778570555520964	0.1252334823415611	0.3751073904598288
0.8064160312956495	0.2122414060501017	0.2445956602482862
0.9797926428605309	0.0194208393964338	0.8657771721440329
0.0701392859341688	0.9707339418055339	0.5834537547150200
0.1537849321466462	0.8088641798332754	0.6973222344483510
0.1466775280565647	0.8585053924599689	0.0251901379819717
0.3979095523148243	0.6523197846238592	0.8546309560895816
0.2346984669091557	0.7333211287368900	0.8179309552836840
0.8666465127147629	0.1322775080909934	0.9834755798046998
0.6095607137683285	0.4076730913998210	0.0428170053815448
0.6663209092788484	0.3195760337548824	0.1855190380323427
0.2264347413144618	0.0760421773681386	0.9270328552819085
0.0807616453858479	0.2230106591533001	0.7782734496506574
0.9344763057410983	0.7705133933491719	0.2320143904378744
0.7729747772148213	0.9209721897037985	0.0799491608988134
0.9891835338293444	0.0077963004738014	0.4715397874789191
0.4752117455580813	0.5429202871560087	0.9841553026157803
0.1857100428234537	0.3271940599342023	0.1737474966891457
0.3395319879903799	0.1772532993310626	0.3274603063650642
0.8449322155003925	0.6801428520625185	0.8229252695865599
0.6947508010068202	0.8246046643220497	0.6764122065871269
0.1840583491289398	0.3010710673348022	0.3287177999420338
0.3547312786056602	0.2180016818290474	0.1609904944274107
0.8241132110491998	0.6721972496254603	0.6985142104998446
0.7136414242704205	0.8310106549609791	0.8033902182817360
0.3033486591619728	0.6360843297145152	0.5379343867450679
0.2381170890802307	0.6072185883431968	0.5132390457222159
0.1840939201568586	0.6825874953694082	0.4629109967625584
0.2483119849901068	0.5050797571666826	0.5271511734307500
0.1405732668791635	0.6587414077087160	0.4269273105718470
0.2021107869903269	0.4812470438178145	0.4920518186895243
0.1513836891435961	0.5599733049618063	0.4390634236171205
0.3318868260438498	0.7672474444265265	0.4743712888955326
0.4168639035222411	0.5997597830713077	0.5013881210172197
0.4489345682105776	0.5283357783676239	0.5755505151249116
0.4840229852986091	0.6255540341452506	0.3925800966738399
0.5444760344676779	0.4824802324620217	0.5444778044603542
0.5819238425956246	0.5805858254188465	0.3618370140804801
0.6108272749985223	0.5048887258266774	0.4392170243998439
0.7099303606348856	0.4510297660693188	0.4086207152627931
0.7699758046123882	0.4162650158077911	0.4649575787164499
0.1767637082889634	0.7615570215465511	0.4497595043956220
0.2604095113036953	0.4295319970286918	0.5929238760920051
0.0987172572335596	0.7195092657292198	0.3873301796105686
0.2052275547380037	0.3999827491016969	0.5091193206333293
0.1176476600574557	0.5438001232350108	0.4096892669324426
0.2464243339485894	0.5804231638367273	0.6354124260674467

```
  0.3979326717457321  0.5058867722405520  0.6597415947282698
  0.4797194109863777  0.7020033648445175  0.3206689215978353
  0.5665756391662492  0.4257444436413201  0.6054746787878582
  0.6358099963399808  0.6064365383407735  0.2756872573044652
  0.3766608446409515  0.5543680982920233  0.4453415657287715
  0.3855352770402735  0.8267851012621455  0.3769584455938001
  0.3819120075409477  0.7831627470337428  0.4925086644370616
  0.2489373827267181  0.7904644764966329  0.5071550078041188
  0.7776689162922739  0.5145549372236840  0.3103935417979750
  0.6725958390920008  0.3719303844710607  0.4355548493648478
  0.8443448749346004  0.3837411728801737  0.4328237806687611
  0.7071440947655335  0.3444801394972373  0.5632628565342250
  0.8064065381073956  0.4933235490324255  0.4407989989332479
```

IWV-Al-T6-I3^{+}

```
   Al     Si     O     C     H
   1.00000000000000
    11.6056394577026367    0.0835597589612007    8.7649669647216797
    -3.6825602054595947   11.3529796600341797   10.3282289505004883
    -0.2346490621566772    0.241282269865036   19.0520343780517578
   Al   Si   O    C    H
     1    37    76    16    19
Direct
  0.5073816262143388  0.8753558316164971  0.5118967986036725
  0.1404184318528506  0.2507855699077028  0.0580607614161535
  0.1294390302969994  0.1116621827226330  0.0036173708748468
  0.0877021793185185  0.3159980437767975  0.3056160341431852
  0.2364545568506684  0.1383471981605015  0.5739868651464443
  0.1066207823750419  0.1300763940254402  0.7746492434124352
  0.3213542597114755  0.1043677048771837  0.3007335046944853
  0.0468008100745649  0.5548812034825481  0.1622727633412715
  0.0111784266264299  0.7798159344539707  0.1197924579696976
  0.2742861369210949  0.2984240844240972  0.1133477506726319
  0.5548184449430767  0.0452306156849957  0.2578737439748747
  0.7758032344916331  1.0087640270540550  0.1082226387510532
  0.2863343730975593  0.2770738564022196  0.3362695649238874
  0.8506174374587989  0.7503662459527169  0.9630378955260575
  0.8852140904295234  0.9026730337902940 -0.0019423659429173
  0.8973515175808275  0.6748766111077878  0.7322121833701151
  0.7589154543891148  0.8429163261106060  0.4537860996966946
  0.9010407714901794  0.8860328030161153  0.2304357370758050
  0.6693847447122492  0.8867091389147622  0.7230101381471394
  0.9343421068868236  0.4330181109708651  0.8746704164984918
  1.0038628721714957  0.2353672334702485  0.8791701250850535
  0.7120991928817525  0.7004557598272919  0.9146548124793383
  0.4358317974128859  0.9495950905469366  0.7590375604980929
  0.2344196549834534  1.0038022531489512  0.8991626346453933
  0.7132159965193086  0.7073800726483137  0.6869126620842895
  0.8896585777401409  0.5005003787303980  0.1262870168328494
  0.0700770346921388  0.4783597898577990  0.9337591461846508
  0.4814759872225405  0.0788321032378907  0.5260281673724843
  0.8944478178753030  0.0914707215700924  0.4693987846820178
  0.8681596936814886  0.1178345639649066  0.3010857822519032
  0.0590134831040155  0.9376009915288896  0.5262120449017689
  0.0857332633000806  0.9083855653211376  0.6892839330207250
  0.4565602568624640  0.5536531700592828  0.9064619688186586
  0.2799905976522356  0.7115690623725286  0.8861908003264443
  0.5082795890101514  0.4811170156900504  0.0757653197257487
  0.6688050505112101  0.3168656778287382  0.1038465550199384
```

0.7047411978096946	0.2843051938986906	0.2644145124332170
0.2399465107136158	0.7373808559792067	0.7287962336261232
0.1860008653693098	0.1879436699380758	0.0054652200363982
0.0028415690109365	0.1770252997282370	0.1778296090077541
0.2257024528398338	0.2442474007151895	0.0889246904478105
0.1523761345165058	0.3904154412641380	0.9594867769900712
0.7689279761768745	0.0189776996994476	0.6045965816808883
0.1837069931360676	0.1883870862264871	0.6391309424727656
0.1310146504795248	0.0277193742968080	0.6503915577122308
0.2728625658753738	0.2486085148587205	0.4412882578917121
0.3474700742110136	0.0929587454432775	0.5712809189011573
0.0053850543152677	0.7985647026187945	0.6041720909694828
0.0080144753350331	0.6417441807083489	0.1879305264359120
0.1712791118788358	0.6282518591067598	0.0285197819694361
0.0663462620696500	0.4442924402986944	0.2591702487894290
0.9401290129974799	0.5028347479625763	0.1821362606580934
0.5894532949205349	0.6003564136578560	0.7852522994717800
0.6428428644544316	1.0213212375270622	0.1689535186926999
0.6174343425664218	0.1748129424404347	0.1948383363574092
0.4344404548647864	0.0546145510953774	0.2662989494095557
0.5275337776197563	0.9413873387362265	0.3836136819465521
0.5925753936077531	0.5847017753719445	0.0307487308335539
0.8250543595472791	0.8311990695367952	-0.0055726248314037
0.9759538354894458	0.8159793781354538	0.8315204313611270
0.7417595223775458	0.7309396590326150	0.9668083953497327
0.8575518262333032	0.6234369288883601	1.0595159723350114
0.2000262988932833	-0.0093654024757330	0.4129741677033780
0.8373190987701092	0.8156894949138543	0.3671443617298211
0.8192669068442158	0.9830051162821827	0.3699142902339145
0.7805491079232286	0.7606337001529030	0.5546894622844906
0.6211598650545143	0.8124821916464237	0.5132844631684425
0.9555714672733869	0.2171065719093974	0.4135535021441782
0.9985052811928640	0.3689127173807427	0.8238395535487153
0.7919103077014450	0.3655638841987554	0.9764794952719720
0.9616303361136126	0.5706009350559920	0.7615197915839160
-0.0148117870618078	0.4247144586936604	0.9346591602304494
0.3848887212717961	0.4082401132976439	0.2116153594393727
0.3709959690643850	1.0063827258593609	0.8185773104197415
0.3332119014076629	0.8402357793007665	0.8294321483130659
0.5365940265103346	0.9024056741327250	0.7781874442582362
0.4985456173360824	0.0518348743075961	0.6171013094855096
0.3830871537243739	0.4213996435200322	0.9839436804717541
-0.0108535148900087	0.4808321811481318	0.0379711841048353
0.5001938543530735	-0.0234383910362520	0.5253071841835614
0.7676379047806207	0.3889990478579776	0.2348523636956260
0.0734871308465898	0.1950778443332807	0.9344306095445771
0.3741335393641622	0.7569843567343109	0.6329907601382709
0.1844076683131347	0.0681633101760470	0.8229519684115149
0.1525346225666722	0.6121024808332569	0.8047328143791498
0.9550470541978954	0.8216848520633251	0.0521620666217780
0.5747000905194832	0.2113178563855718	0.3945612182119096
0.8123937591848001	0.9470232749968548	0.1955881019414499
0.0253671688655452	0.9886697079661191	0.1383605500110062
0.8685412119627087	0.1215008906087032	0.3842744863734547
0.7865391813058278	0.1961238613292408	0.2643619544278466
0.9819258204693404	0.0304630495487548	0.8631552719297741
0.0398989750100771	0.9477385744487106	0.6136109306494080
0.1894654989615571	0.8455937079976857	0.6637987446521965
0.1477898477881971	0.8665141155862355	0.0224333807874095
0.3845043950796231	0.6475435510340201	0.8681200314790178
0.2245434088812520	0.7298805194542771	0.8258108395295145

0.8707960606538561	0.1416835803752035	0.9820658227177830
0.5782078562117605	0.3858690459670702	0.0750262952349082
0.6914970929044308	0.3466945914896406	0.1607724225942360
0.2358576234078579	0.0784756783190407	0.9301398181891803
0.0797205646229705	0.2388537643901066	0.7705074125188367
0.9293443815062541	0.7858623718119901	0.2217252834046022
0.7818057882640750	0.9268236221995466	0.0817992140089474
0.9869736050935560	1.0127305195619698	0.4733714677733369
0.4728054985314506	0.5471537035788913	0.9875288282787313
0.1651306524402160	0.3236410320366954	0.1939342567385882
0.3272419025553429	0.1714918606403374	0.3371757507642489
0.8259428403503896	0.6720438831684188	0.8405431559003783
0.6828985330104729	0.8139035404125275	0.6864070883602045
0.1589321337547112	0.2810853459525528	0.3585227136067169
0.3237900300954346	0.2017392381874119	0.1847111355614352
0.8012073481740997	0.6529460995692836	0.7237520404888097
0.6877182379529468	0.8145080050504249	0.8243712328497882
0.6613933240439152	0.4290877371456812	0.5132791330117356
0.6576686951347883	0.5425791056711485	0.4663446970468013
0.5531241568972263	0.3444217631411217	0.5858273902743574
0.5474299052258360	0.5761510625092263	0.4888869548894194
0.4462772608577982	0.3715544424502437	0.6076492879110582
0.4400088791641025	0.4858672179578326	0.5593998408720507
0.3289168437851662	0.5194787739248325	0.5758019180421047
0.3610313545382226	0.6165064252848359	0.4514153390868870
0.3911680456599322	0.7380181558218524	0.3873103338233050
0.3670519370501948	0.5819430019744416	0.3977323105122244
0.4262290413467618	0.8237945405813443	0.2710700144631546
0.4024001672748888	0.6676593707361671	0.2814800104782758
0.4323935467103909	0.7887911444379447	0.2178943816373557
0.7724564793106579	0.3922580346528143	0.4937946239803673
0.2760018566601362	0.5545390752715346	0.6479618691962341
0.7688644455492459	0.3269256720853543	0.6016569748177413
0.8493898682222978	0.2984518016725713	0.5867866872804810
0.7649869419904755	0.3856049280653049	0.6153025875692779
0.6898888781396981	0.2472143624889359	0.6845375689497748
0.2488776001359876	0.4781582730688939	0.7383867298689420
0.3397413795901337	0.6287497603695180	0.6061180084868881
0.1970977285744701	0.5819338859198067	0.6551855980521517
0.8514546832994749	0.4718240400757369	0.4125099008714654
0.7812034924895011	0.3338762680065707	0.4785395887667767
0.7377784958259047	0.6110578463178040	0.4109470930037220
0.5538320809612602	0.2531108099577473	0.6256782766195095
0.5651352278110695	0.6604200434468332	0.4040902956011952
0.3675211818427255	0.3024374679821853	0.6621578046026809
0.5239886915780259	0.6072999634060392	0.5341977482773010
0.3859790518946202	0.7668629246839560	0.4277635726688709
0.3432396924647282	0.4871937765337263	0.4477128959260176
0.4485908226083973	0.9179644982172811	0.2226870392031234
0.4070262335190034	0.6396121951600666	0.2406382714427461
0.4605268809450173	0.8558944804529326	0.1269657546252301
0.2625608671801909	0.4405967906531188	0.6266895270835726

IWV-Al-T6-TS10

```
Al     Si     O     C     H
1.00000000000000
 11.6056394577026367    0.0835597589612007    8.7649669647216797
 -3.6825602054595947   11.3529796600341797   10.3282289505004883
 -0.2346490621566772    0.241282269865036    19.0520343780517578
```

```
   Al   Si   O    C    H
     1    37    76    16    19
Direct
  0.5330437652413879  0.9178346133460490  0.4811873729264411
  0.1635663088204166  0.2447568671986225  0.0472217318269216
  0.1291316872755132  0.1062129516283818  0.0002392907853444
  0.1125977503923598  0.3218157709846792  0.2798116003217694
  0.2633500276859106  0.1465298414568535  0.5538015875207807
  0.1095614902689576  0.1177595827077486  0.7730364818303905
  0.3399023501213347  0.1093407747356835  0.2851755238548558
  0.0657494947661306  0.5611621580200722  0.1373483219059478
  0.0148084525749367  0.7687571263594134  0.1220122154217401
  0.3033479766624879  0.2995838663332926  0.0912996195397391
  0.5797933527768451  0.0626743469120466  0.2313025022041835
  0.7765223675390485  0.9908539800815572  0.1110385218802560
  0.3041224874907581  0.2828574221962064  0.3201412186377052
  0.8753643546997719  0.7637631637553323  0.9357245375806384
  0.8849911324491782  0.8919077544142331 -0.0000876373534959
  0.9167503497093605  0.6803520094651614  0.7144654518768285
  0.7696507097235991  0.8573327984702398  0.4375796201293531
  0.9009514611065252  0.8722769563693291  0.2316197756796690
  0.6884914975044022  0.8985500895828430  0.7092676465021637
  0.9625465533820468  0.4414257077575711  0.8527269962453055
  1.0072017771038253  0.2254906589429732  0.8791695464893474
  0.7318991083582356  0.7062249526774640  0.8968913180744000
  0.4551923212753451  0.9571796534748463  0.7483891996043869
  0.2366820133560400  0.9935115000842851  0.8975389788740171
  0.7289766611876942  0.7222304367823045  0.6698765273637447
  0.9346670198088528  0.5202467774444054  0.0724748108856595
  0.1255437416429100  0.4849959535298076  0.8919445474065474
  0.5141791329419967  0.1113929522362073  0.5049392374480435
  0.9095025998311679  0.0947099817104472  0.4548137690490401
  0.8868310188773938  0.1288321080456508  0.2817822498543705
  0.0775050010913342  0.9451365093696722  0.5071502314628983
  0.1031625783978368  0.9166006446999193  0.6703213116684806
  0.4721508479126806  0.5566410054492267  0.8906099151986857
  0.3020304855483060  0.7224429746049377  0.8641970567103119
  0.5265940351329997  0.4896234905652849  0.0561691794520928
  0.6884823080124989  0.3280590744134388  0.0835826840010582
  0.7254318312027632  0.2971108612770246  0.2433753096052020
  0.2597699765212840  0.7497582158609801  0.7076152907306640
  0.1886326402481200  0.1672351209595868  0.0125757588428553
  0.0264771301183737  0.1904347242405699  0.1648065134395927
  0.2544715565023764  0.2364748617987315  0.0736492492791233
  0.1907051284192993  0.3845809023279283  0.9365410178159241
  0.7865754387420465  0.0310417957260483  0.5887288587182886
  0.1810916600327485  0.1746951765406544  0.6387141057033711
  0.1977356647579765  0.0199969929410616  0.6153254180988572
  0.2809805018864308  0.2534861228143841  0.4296439874554971
  0.3884784214224323  0.1427681612572519  0.5323858426052919
  0.0286797574883169  0.8027848911900483  0.5910087789058027
  0.0083368209560628  0.6317150664502399  0.1823581479027780
  0.1915940191789137  0.6507320638885552  0.0040688820072589
  0.0884186546958928  0.4496120274030682  0.2301045769093101
  0.9719253749191665  0.5114675561547214  0.1427197480418076
  0.6079745519059994  0.6118685726900611  0.7677474164915907
  0.6455653268269210  0.0066064865429850  0.1708481410695628
  0.6526038206646705  0.2048433332317972  0.1246460953945920
  0.4461852121539039  0.0488689617034214  0.2676743643483852
  0.5818670199442847  0.0003200443815565  0.3378675441417967
  0.6053616704889130  0.6003318882443319  0.0069165029962948
```

0.8283181152667943	0.8094249551159941	0.0040204208379032
0.9791771307447966	0.8741642008476485	0.7943428968157290
0.7609472270646347	0.7225024569329079	0.9579874015315384
0.9293798225907760	0.6547588715587979	0.9871950014974837
0.2151429502001683	0.0024669951691767	0.3869213440765194
0.8371016846041150	0.8132835656917745	0.3651366112462942
0.8411615477839401	0.9986715660500873	0.3386012962376070
0.7939492338652775	0.7805648177750359	0.5362507763712812
0.6297459208355114	0.8324002197220745	0.5019032909004539
0.9821579724747728	0.2210157791417201	0.3888710081095859
0.9845469463947349	0.3543333738151243	0.8284920597512804
0.8306013994316455	0.3915560031354305	0.9737188244495123
0.9756347371587289	0.5721380207830570	0.7411958290811884
0.0589612202986395	0.4494896434018715	0.8665173627196325
0.4113242330956295	0.4087804125194312	0.1964333672127723
0.3743917602114493	0.9965159572651534	0.8229377615169584
0.3691359824583473	0.8496260410400677	0.7998292391521148
0.5554214661918455	0.9124214913041871	0.7691211666492024
0.5176460210045830	0.0713443950106804	0.6079275896543279
0.4086535853715177	0.4246273487973015	0.9589926275537880
0.0316798750890110	0.4890964196877248	0.9945876162234578
0.5327904073747352	0.0128522190659733	0.4991393609455471
0.8031571472256038	0.4265421597653122	0.1687336242490244
0.0905868918331258	0.2057066592559338	0.9168156268419252
0.3898109938987044	0.8226537604598564	0.5832189557240893
0.1920868037711267	0.0601898886488138	0.8162210039626210
0.2268966505565221	0.6128552226151291	0.7682667822452361
0.9424722042281097	0.8148308156384042	0.0686053716146077
0.6211899391835390	0.2391191362121244	0.3793209747387566
0.8018971975907825	0.9034534330302003	0.2086000015622937
0.0113918221091360	0.9923663967520632	0.1308548745348390
0.8736280119119780	0.1182649253653542	0.3784653785955959
0.8123192759938132	0.2138846652264555	0.2376761238985837
0.9878012674956710	0.0133882803213306	0.8630588154222000
0.0714924702372309	0.9684099522093980	0.5812016390990282
0.1558116506861009	0.8057908667601512	0.6941503748656869
0.1527151419200510	0.8544519816238805	0.0156133563176949
0.3946302234035177	0.6447124023621319	0.8562599416353477
0.2456543160224281	0.7432804254821429	0.8036298793634485
0.8806431188249241	0.1189686964582189	0.9956901928987423
0.6121802645647220	0.4097419390609691	0.0386753842297627
0.6638810542448383	0.3137277855514228	0.1870538052907783
0.2283659625033918	0.0618527106417061	0.9369956563152059
0.0750564733785143	0.2224481672254517	0.7751920382829035
0.9502577062968305	0.7722063871031051	0.2250304560168980
0.7772372846707750	0.9257717870094258	0.0706636434646224
0.9941882999556158	0.0074069771524195	0.4658619373417636
0.4782118304616295	0.5445632648758589	0.9795811641559846
0.1926723785801862	0.3246403473694519	0.1712701646268617
0.3405495803049628	0.1741446577457669	0.3254060279432792
0.8432109867926255	0.6737207975829764	0.8253907539515829
0.6947267483617726	0.8227608941306986	0.6772764415005698
0.1827880530809606	0.2957047229136324	0.3313472281247329
0.3587630189623805	0.2110619390246717	0.1596514252302948
0.8226574821771853	0.6691082128441403	0.6989171934324373
0.7178095282321206	0.8288663107672480	0.8026827386227405
0.7025584192872979	0.5370393334517978	0.5403408943617461
0.5947261505807429	0.5632470782666222	0.5682385227454648
0.7653457484193882	0.5263926103837125	0.4555381673086987
0.5463118871130742	0.5741698133854567	0.5175949662124936
0.7213105701904350	0.5376464598145252	0.4033931336392139

```
  0.6095451811257658  0.5612478415081218  0.4320827489761199
  0.5539817122985498  0.5573823595748301  0.3864244690793586
  0.4474175198356220  0.6051593529488002  0.4102575172811540
  0.3288562663052971  0.5301734102273151  0.5051813800551000
  0.4690651324844955  0.7285532579483542  0.3398899727004452
  0.2338225244446741  0.5779018719790393  0.5299315594345260
  0.3744678726842467  0.7761701134351019  0.3654020273629759
  0.2563619106666440  0.7005005027498961  0.4612886334576692
  0.7494859636524219  0.5205026579798990  0.5997423352595211
  0.5264263530600546  0.4293732848534621  0.4411012800677479
  0.8850429512266921  0.5445831129823254  0.5306620347856925
  0.9099705338609484  0.5381249401622541  0.5780313199144795
  0.9158089549115049  0.4797068483122709  0.5224491301280175
  0.9329232625709390  0.6346148773166027  0.4403815155240494
  0.6085197389870398  0.4036778912230244  0.4123071711183022
  0.4651759965686555  0.3644358703515486  0.5396025466012162
  0.4841742330896348  0.4235267266683527  0.4115799392661783
  0.7028515210581299  0.4277232084755158  0.6882017012675152
  0.7194505263809469  0.5767469494135894  0.6195441254507398
  0.5466438802877112  0.5772986166916555  0.6288503974862020
  0.8520847469558349  0.5106711535873070  0.4291802972269418
  0.4606818942711520  0.5924028458939657  0.5410282832360721
  0.7717589954681594  0.5286587522257413  0.3388589473117497
  0.6240387004513753  0.6642223929888049  0.3968936717075672
  0.3095228433246089  0.4341008500802056  0.5604220128314867
  0.5610465188600913  0.7883439962201337  0.2638788513326484
  0.1419996003074026  0.5185583296267381  0.6038241785299504
  0.3932469531087900  0.8719638104024690  0.3111217631007279
  0.1823022637202759  0.7370336746577036  0.4827472763470769
  0.6235084394374144  0.6165587661872177  0.2884792055562864
```

IWV-Al-T6-TS11

```
  Al      Si      O      C      H
   1.00000000000000
    11.6056394577026367    0.0835597589612007    8.7649669647216797
    -3.6825602054595947   11.3529796600341797   10.3282289505004883
    -0.2346490621566772    0.2412826269865036   19.0520343780517578
   Al   Si   O    C    H
     1    37    76    16    19
Direct
  0.5317886285880998  0.9413692847708648  0.4849807276047550
  0.1620427422300940  0.2635844753983848  0.0515999281320074
  0.1245058977627708  0.1205110959332043  0.0094880096228716
  0.1130689622444928  0.3405999147315985  0.2840177545762159
  0.2605985639075904  0.1633561299229872  0.5610346538885541
  0.1054479283428355  0.1323325963987150  0.7817738172387969
  0.3373172913066372  0.1265961323118210  0.2915294992472303
  0.0643876120680841  0.5793336734001685  0.1424552060842153
  0.0090733801375536  0.7837325315832121  0.1315315500088772
  0.3017766601380761  0.3169092633713621  0.0966845852273508
  0.5769588313226780  0.0821961938557882  0.2363994914370683
  0.7728751487349416  0.0084964504647355  0.1175826400212016
  0.3047170468215743  0.3008599566560590  0.3252387646849080
  0.8737404717305702  0.7816134411668305  0.9418075969626301
  0.8812318523722433  0.9089425461887074  0.0072665097777324
  0.9145941636954101  0.6979834576979004  0.7210976937374596
  0.7678789963171297  0.8756719733222046  0.4436430886832801
  0.8956508820311104  0.8865149734139280  0.2409197404281657
  0.6868104065161237  0.9183481531633124  0.7137141063905422
```

0.9618203522861503	0.4579208228683654	0.8587020830240617
0.0038890062593233	0.2417712286922607	0.8861366769656026
0.7303601216444302	0.7265836331748977	0.9011775743212672
0.4525063730446405	0.9752046238954533	0.7545848478759914
0.2317080656569009	0.0078728663978324	0.9071837966822704
0.7267212152096255	0.7404804219844211	0.6760622397242340
0.9355785515879260	0.5383121799187960	0.0753726170769180
0.1276631331776221	0.5046319996956471	0.8932202692670388
0.5119468392620982	0.1334187385733024	0.5104361521903242
0.9088363505256118	0.1126235503307672	0.4609343623536252
0.8864528969724664	0.1464540551043583	0.2874381938302096
0.0750924088164832	0.9614085765217126	0.5150957076669864
0.1021575012959227	0.9334954073447896	0.6772048505501963
0.4698812491108726	0.5725478790200074	0.8986724964401206
0.3006341653426033	0.7396355035029604	0.8710012298767056
0.5273195205941692	0.5079384192506220	0.0616645118282526
0.6888033567157501	0.3461406016471555	0.0892533479334973
0.7235354187180211	0.3138827391792158	0.2504978704186769
0.2593139538918544	0.7680466782471117	0.7131987489436008
0.1847722791159945	0.1830949237750872	0.0202596366940273
0.0244517403576267	0.2138826980460083	0.1665183830277360
0.2524243363798508	0.2539292412783041	0.0790443590322694
0.1919345590349275	0.4032993199394623	0.9385512487150827
0.7837219887278905	0.0516062590696471	0.5930240290946344
0.1760103100089801	0.1896417687692556	0.6473283030286293
0.1988717074294145	0.0362114450413018	0.6211000067444048
0.2769727727106701	0.2703465239153118	0.4372329265633211
0.3863134152601496	0.1648407511988176	0.5378298319890129
0.0288705465472791	0.8186660739584235	0.5989889525738494
0.0038661602828175	0.6481292977424229	0.1886838140779652
0.1909827494984243	0.6714958377696196	0.0098408321309541
0.0876333169331658	0.4676237463008512	0.2346226483106428
0.9760116466968651	0.5291829591010943	0.1433801220493758
0.6061233036893977	0.6291235087137090	0.7765133996414881
0.6430406267673470	0.0264757636701567	0.1759502590278170
0.6478494867157468	0.2250051179685006	0.1295936594755137
0.4426925083898086	0.0651969626270696	0.2748401004736030
0.5791432416963833	0.0218047792408163	0.3425533520181923
0.6024289009975342	0.6224940613085456	0.0105340979137084
0.8255739147260411	0.8261312314104614	0.0110812340815318
0.9787254755367092	0.8925834773879172	0.8007199874128822
0.7596113547593745	0.7425185784006423	0.9623062773337259
0.9271742624303040	0.6718801335667817	0.9927564381723402
0.2117978353774426	0.0210025940441958	0.3932112069299810
0.8309562347234914	0.8289356695189778	0.3739387545961487
0.8458945326874237	0.0158465185582948	0.3427070283816270
0.7904912575112559	0.7977743557429705	0.5433422004078380
0.6290229895274969	0.8575945995398341	0.5049713989635507
0.9839187060002008	0.2371598011251782	0.3957981734093692
0.9844920249425559	0.3722707675557912	0.8327045805404758
0.8326571604453810	0.4038247907445088	0.9836654380710042
0.9710087269441497	0.5877369175242777	0.7495317976840392
0.0644703956815225	0.4705519706907279	0.8642250582703765
0.4157864279926998	0.4242603259986363	0.2037630108167983
0.3694831586407492	0.0105972141301630	0.8326929660965237
0.3690965014685095	0.8687419007620840	0.8025460343503119
0.5533848899344018	0.9314065192392005	0.7746291334857097
0.5143040006798582	0.0917304313620938	0.6146370827502188
0.4070341589342030	0.4416548579427094	0.9645061963156969
0.0319004921711687	0.5092812369122025	0.9948758199234815
0.5302303427900065	0.0359271747844059	0.5034689788434029

0.8048748360959124	0.4430985656102781	0.1723685870520867
0.0835170404220440	0.2199149126307167	0.9282711611464056
0.3885241270026150	0.8452477905275165	0.5877151811169653
0.1861945846262468	0.0709302063027130	0.8267866754933136
0.2297283592472489	0.6324318949155633	0.7694010737058121
0.9439311628221848	0.8357013587199511	0.0705821567915900
0.6191341040081770	0.2610012649450993	0.3845963103375922
0.7999258957602259	0.9257442631813860	0.2135055606714782
0.0108299121651689	0.0033510489851469	0.1397404544693348
0.8764849011614529	0.1378959489483882	0.3816957005545376
0.8060097771678323	0.2263098453358995	0.2496009536011951
0.9812857501429346	0.0323082795652305	0.8700686234100312
0.0705313746579109	0.9856823020731668	0.5879913497633802
0.1535582940802791	0.8217935487473887	0.7019785839346970
0.1470390080077928	0.8698942795119041	0.0290946263166834
0.3924577188202026	0.6610809421341448	0.8650772540662031
0.2435581586030845	0.7572296580003186	0.8120387351648346
0.8765215177902874	0.1374562862632449	0.9995707523491846
0.6186636101624517	0.4339330398558342	0.0388072785641662
0.6617178606481037	0.3299513501319394	0.1945364619153062
0.2267905824169866	0.0822636360886567	0.9402986248814469
0.0753400754804475	0.2385739032134829	0.7820087410009204
0.9386807941859262	0.7837945393337467	0.2373741567829619
0.7727553399682773	0.9405420228433049	0.0801473326009188
0.9888046481993459	0.0209175387054502	0.4772651573947275
0.4729494696935959	0.5562020463620301	0.9912870794246603
0.1908316719227124	0.3418099985324595	0.1766046772154619
0.3390444167760545	0.1914187613910996	0.3315045587829916
0.8406797567230500	0.6915188924838365	0.8320023676227870
0.6926533696380577	0.8419043141712708	0.6825733216458618
0.1863235338471361	0.3184937543665998	0.3315980417229224
0.3566185772827392	0.2283556691200340	0.1657413974811114
0.8218416858352120	0.6895903577054843	0.7033537030874866
0.7180875590487110	0.8499934649975230	0.8056720187432400
0.3097010273417691	0.4731665602368783	0.4567792998565667
0.4250539336375514	0.5094923384285823	0.4122193938021388
0.2166884156774152	0.5006520370458094	0.5193478520034573
0.4466942417130001	0.5711186822896850	0.4332099194419990
0.2365684564552315	0.5618713312039306	0.5408205586915433
0.3505728652699903	0.5971001647880209	0.4987468294056573
0.3796745093537220	0.6401566547180496	0.5367666235338787
0.4477691409844355	0.5569148203473232	0.5812767955967579
0.3846146501305873	0.4330443839732136	0.6886573704362904
0.5677233545823194	0.5995891712832560	0.5284850030426460
0.4421952274247362	0.3561631656418240	0.7328309788527900
0.6240522626319749	0.5211247701898066	0.5734582482012966
0.5630324205529348	0.3983450472332780	0.6754169087771449
0.4514994133961175	0.7724714081871782	0.4331873173620698
0.6243045033652441	0.3122876214179099	0.7227457944819067
0.6338487336729151	0.2211819364202178	0.7127221352057690
0.6873375121889209	0.2659291498466660	0.6186914059858877
0.5447335830640250	0.1695139620179633	0.7622129215038798
0.6769323203289787	0.1583442230219176	0.7500911399088249
0.7138873296528589	0.3619054155242359	0.6744656421973457
0.5723870841090122	0.2639721590247788	0.8178575533919553
0.4992629404556794	0.4934034782511000	0.3588102058622069
0.1261505928252816	0.4707363905308375	0.5553306418260675
0.5397434999720803	0.6221755932183414	0.3752483590447767
0.1620659588244145	0.5781651197787903	0.5938289722172985
0.4569661667613757	0.5148568798040460	0.5291246660098099
0.2910785120113764	0.3992785820323611	0.7329981231514100

```
  0.6159373522128162  0.6946311049441362  0.4514491793240967
  0.3928793354997924  0.2619181579267661  0.8133658181363786
  0.7170653578145380  0.5555787097292308  0.5297454556183356
  0.2964287916911275  0.6258589152996742  0.6124752933144340
  0.5347906344371191  0.7901268648623818  0.3553305170765189
  0.3989695361846556  0.8259804043721976  0.4043131975968332
  0.4733919522746374  0.8026797831688475  0.4607367556923350
  0.2916028563881063  0.4246599551883028  0.4422432774027220
```

IWV-Al-T6-TS12

```
   Al     Si     O     C     H
   1.00000000000000
    11.6056394577026367    0.0835597589612007    8.7649669647216797
    -3.6825602054595947   11.3529796600341797   10.3282289505004883
    -0.2346490621566772    0.2412826269865036   19.0520343780517578
   Al   Si   O    C    H
     1    37    76    16    19
Direct
  0.5206666227867334  0.9138333391767229  0.4902229880830126
  0.1572064402089115  0.2400025945141845  0.0502996947504863
  0.1199483360212582  0.0937860154631788  0.0129686891396755
  0.1040991128572433  0.3137560114251820  0.2877510133406533
  0.2518760495763430  0.1362693407341966  0.5671518991670274
  0.1078104813800296  0.1041179960761716  0.7881041849142930
  0.3262160444619550  0.1025394816470011  0.2944625419337915
  0.0544764126951553  0.5526645452762907  0.1490194125069140
  0.0023573830972514  0.7585202087149591  0.1342524937166959
  0.2923877802864065  0.2929197060286772  0.0979319337294401
  0.5650414653929331  0.0582563487513689  0.2412621304044276
  0.7669334741120860  0.9919733721029540  0.1153271841059512
  0.2949358626964355  0.2747840508588569  0.3309645628811992
  0.8655808452437123  0.7588195457442706  0.9439706053893026
  0.8796195491681537  0.8858725319124158  0.0081313895521798
  0.9059383153471712  0.6723250858246149  0.7248571760482885
  0.7569379585640199  0.8497101014208737  0.4471540660137905
  0.8945000610176547  0.8668528689834133  0.2389720559333467
  0.6805552085483820  0.8913527399463368  0.7168992007213233
  0.9553610707101985  0.4327625836813081  0.8615730644956581
  0.9934756862905494  0.2154692554254203  0.8938594173299955
  0.7210505818448755  0.7001882993269100  0.9063589315802263
  0.4464555298244700  0.9460029318930001  0.7605280748148450
  0.2294247614563952  0.9809348848393935  0.9097486531151484
  0.7204232236369096  0.7146945478734036  0.6774257342329232
  0.9261607921941786  0.5151784729482808  0.0799402853222487
  0.1169956547522677  0.4799808067325070  0.8992628692807395
  0.5049076776635932  0.1052832630115243  0.5148448777039744
  0.8994825170432003  0.0871688982693855  0.4634039993667791
  0.8808974144217746  0.1218585076140580  0.2876931786530704
  0.0653959177204148  0.9369216652087752  0.5182280532019967
  0.0940882515915676  0.9086239018951227  0.6795791988345518
  0.4613120154448722  0.5469595251232563  0.9014876274058204
  0.2903270825501751  0.7119331604198245  0.8775302883412754
  0.5175152348616335  0.4812244607347790  0.0658947632234215
  0.6806137014631686  0.3212720120959600  0.0920439587922349
  0.7188793874955480  0.2908120516328812  0.2521066012623962
  0.2479199059532936  0.7422103968007581  0.7193514295744734
  0.1857675722676729  0.1646133797046514  0.0131083889365564
  0.0192599095015203  0.1805624101905385  0.1706760893990258
  0.2462267144169128  0.2294007663992403  0.0794367942847184
```

0.1824425145803500	0.3804233831408503	0.9441778227630306
0.7743487087385830	0.0260786209058722	0.5956623319656396
0.1701587884292027	0.1586077156101243	0.6562103622431709
0.1903809789536251	0.0098456292160786	0.6254143898631110
0.2655783245475744	0.2413218120193343	0.4446512681416426
0.3803752425730471	0.1373915990310794	0.5399630837277660
0.0187165270856135	0.7940264227832426	0.6027790044218841
0.9988217390795996	0.6226003150644894	0.1938956647081977
0.1810246114570694	0.6443394986626695	0.0157286367802009
0.0777976972300463	0.4398288160890551	0.2398527198044807
0.9642688205386952	0.5049297377605300	0.1499579320862924
0.5980549702864523	0.6045424971133997	0.7788752244741111
0.6338063689151533	1.0014098842895229	0.1818632021429601
0.6358646993178394	0.2011453082443084	0.1328550759471778
0.4300024912605389	0.0386798857158737	0.2826025145662285
0.5676123481613110	1.0005206888721911	0.3467479264903433
0.5932716435857233	0.5969009051154079	0.0130591701541215
0.8220706083526860	0.8085983212481088	0.0077355449478829
0.9687860697044095	0.8676364302370512	0.8019934316632650
0.7496952437517709	0.7162403785638879	0.9676084195791351
0.9179877527901907	0.6487428030413968	0.9955666744567604
0.2001479941548120	0.9981417225234774	0.3946447131278096
0.8215969012483555	0.8066891146250121	0.3746200610289981
0.8318485782142546	0.9906651960721347	0.3481394089290131
0.7821291205310009	0.7700618088489863	0.5458539098401678
0.6173438150281417	0.8287542464386772	0.5114430206131103
0.9740135430195617	0.2111430793741731	0.3990372026519560
0.9855267492878733	0.3507230430849945	0.8308931866454102
0.8231074303330718	0.3759146258984520	0.9851899688590956
0.9625285644201433	0.5630189921203403	0.7546552735086818
0.0509552546848036	0.4423340439194921	0.8748459152754369
0.4067251200819619	0.3983612454333564	0.2077840558606953
0.3664738436802790	0.9808971098997196	0.8387314298916165
0.3637701761861083	0.8384503147322909	0.8098703170092793
0.5468277905960612	0.9015403338887891	0.7817326891985865
0.5070994021983214	0.0613057041287652	0.6205498512952256
0.3990544028598901	0.4169480452959862	0.9659886998630624
0.0215446614443930	0.4832095776535971	0.0024369930563888
0.5232624714175583	0.0070479636664428	0.5090567617573698
0.7944001961451156	0.4204279086328550	0.1775407987503606
0.0569165626230890	0.1816046676863316	0.9530485703884068
0.3767917318336004	0.8203925457593175	0.5933554502182149
0.1938995630036846	0.0386900758007955	0.8229351711136770
0.2183688486719304	0.6073748391036669	0.7755974560264177
0.9432598039734661	0.8068520721230923	0.0686032161447914
0.6145227805682851	0.2310023314925911	0.3889635613381401
0.8133652103127896	0.9271501962106078	0.1947585666276912
0.0184927314106532	0.9695105369049664	0.1477751410727726
0.8691717000987292	0.1137331577202699	0.3825775557300446
0.8064372210538033	0.2084828172756338	0.2447906897691144
0.9811063621336981	0.0089995757074683	0.8745537411843913
0.0630543879977084	0.9618934346720268	0.5895985186547413
0.1421167382186505	0.7950182991880931	0.7074920404808164
0.1392737428808911	0.8460503403684839	0.0334796449370141
0.3819078685501981	0.6331428296257773	0.8702160921739504
0.2323846660739023	0.7319209447315991	0.8182095594887956
0.8589899126820250	0.1272955932539706	0.9920955459975165
0.6096252423824738	0.4082625170238466	0.0422766694489962
0.6588739804028118	0.3075452390939268	0.1941654550681854
0.2208965477506357	0.0643947430943888	0.9341684593820613
0.0785478395791561	0.2056572425097983	0.7941374735366309

```
  0.9272222301427955  0.7615611393601333  0.2382172226623066
  0.7726725145194688  0.9168070309796396  0.0826833694189394
  0.9784886616028009  0.9949488255619416  0.4808973569162551
  0.4627548919519608  0.5281694605069276  0.9961915685236911
  0.1804666613477319  0.3159774091787835  0.1795029722839120
  0.3283219929089080  0.1668003962792529  0.3345692920021259
  0.8304022071112884  0.6655868248240564  0.8360990615248500
  0.6881015585313425  0.8167189028273082  0.6833933602902211
  0.1782548365850568  0.2925069855268715  0.3342529177969114
  0.3460402239613590  0.2027144900859267  0.1684601334182638
  0.8153060765491612  0.6628838656307166  0.7050480853090498
  0.7117138265278444  0.8245517098036179  0.8085306251198214
  0.2623714305269459  0.6515006557180694  0.2777284131130356
  0.1674580479688919  0.6528885446302262  0.2744785864253051
  0.2625528979761922  0.5460723067570665  0.3693187314311774
  0.0739818838805976  0.5500777917613857  0.3609214862534322
  0.1618891886390871  0.4418200042927894  0.4629129970904143
  0.0664482293719406  0.4431609066219245  0.4590001261123746
  0.9406391064113501  0.3489214537319446  0.5576095811286458
  0.8504759948027896  0.4190398824097245  0.5788308344691429
  0.8427601468361966  0.4480676174200242  0.6395838229210491
  0.7538380403441031  0.4268373743473146  0.5722134533910902
  0.7549406677594448  0.4934090808987359  0.6790126190186405
  0.6659883008417974  0.4728828858474387  0.6123461664617932
  0.6662399190970203  0.5051711889078435  0.6660934927413349
  0.3646013095997777  0.7644726201388193  0.1785092342132195
  0.9208085175740465  0.2340996828078442  0.6721181529789971
  0.4797891932327157  0.7439200835526086  0.1642382242454937
  0.5536470166700119  0.8275023475976218  0.0866166816976048
  0.4682365896242936  0.7074262335704766  0.2431995516668576
  0.5064284460626932  0.6806937821227287  0.1531226183907062
  0.9819987229077155  0.1867483617013873  0.6510631090906407
  0.9408874238725897  0.2538913420910567  0.7079686110897623
  0.8286483685542601  0.1740494091021667  0.7428112272828707
  0.3359833268991537  0.8224142770469545  0.1966991726851895
  0.3809272096737424  0.8119302857998304  0.0943594982184947
  0.1693145941133372  0.7335810864529823  0.2007739724001474
  0.3471909734120497  0.5585853685773600  0.3558525023693480
  0.0105328675102989  0.5523118273798262  0.3457479130508910
  0.1549187307466589  0.3593232425106863  0.5393645061840120
  0.9245830311902247  0.5167251562471273  0.4865271875389090
  0.9159397069854397  0.4426228980816951  0.6434333124004591
  0.7548650820845533  0.3973179548157331  0.5332798867679949
  0.7567399204334625  0.5205365569593804  0.7181663596854796
  0.5941458984173431  0.4829762566967316  0.6053829723274713
  0.5974361315475719  0.5406930927989697  0.6987317846922372
  0.9242182988513568  0.3259032243955559  0.5212652506875904
```

IWV-Al-T6-TS13

```
   Al     Si     O     C     H
   1.00000000000000
    11.6056394577026367    0.0835597589612007    8.7649669647216797
    -3.6825602054595947   11.3529796600341797   10.3282289505004883
    -0.2346490621566772    0.2412826269865036   19.0520343780517578
   Al   Si   O    C    H
     1    37    76    16    19
Selective dynamics
Direct
  0.5414755046719675  0.8978265277645152  0.4872212020136224
```

0.1732611919749869	0.2755800503551733	0.0356212071182045
0.1744384512335233	0.1433458665141600	0.9707373170468047
0.1200304939917421	0.3387045281009702	0.2923308794223263
0.2712164260721169	0.1618750002614489	0.5527498315786622
0.1397107154154738	0.1555728540862129	0.7520295564752875
0.3534941838493970	0.1286992109728123	0.2825042395172408
0.0800047872751889	0.5776858963975946	0.1439914180493845
0.0495171297751816	0.8069021510683563	0.0968199035684900
0.3007148370983418	0.3173082662758527	0.1006798418195444
0.5898757271607100	0.0671302401117381	0.2362278989752983
0.8174078723971789	0.0375127324139457	0.0830389601620470
0.3245440363627718	0.3060108655314343	0.3137471338836736
0.8836401265347206	0.7744183313165898	0.9438391542069409
0.9203986982351756	0.9285381783167302	0.9760834881518483
0.9304502451833003	0.6971352955960501	0.7121612820594969
0.8019378638631030	0.8738725594713527	0.4293158808275448
0.9403414247829674	0.9153242024537167	0.2043898839620667
0.7012288814028735	0.9072454840517288	0.7007165594559557
0.9654395244182894	0.4503806723609882	0.8595600570220416
0.0494674287962348	0.2656029940783357	0.8493312500900134
0.7504496339029946	0.7272961010392407	0.8892959546787924
0.4696419737463292	0.9687666173256558	0.7356178643850599
0.2726668113121576	1.0309152626319722	0.8723948756326603
0.7427135369964948	0.7242896227378288	0.6702631539281895
0.9239386797573900	0.5250407328278325	0.1049692229053227
0.1050745929479527	0.5028429007850209	0.9116718028376556
0.5134960735608564	0.1015100333885960	0.4998872475745444
0.9347456549061349	0.1153339290549190	0.4481835161169955
0.9091263037177467	0.1444977924910920	0.2809759709260753
0.0929302604189709	0.9548634780190106	0.5105824719355316
0.1201241257319796	0.9292059857509318	0.6722109660614521
0.4913347496028530	0.5690964978815548	0.8905490418226689
0.3143328097490387	0.7260169497173498	0.8720401779451877
0.5503593483128169	0.4996591617655121	0.0558095380661261
0.7046539115035795	0.3280156994521315	0.0941352568750737
0.7387532143850631	0.3034869966041341	0.2507344162322318
0.2751172834037464	0.7571226649208365	0.7110914168639600
0.2247742012898341	0.2128959325317080	-0.0174941699934500
0.0322103025088168	0.2050825001950327	0.1488523137259005
0.2475255398754225	0.2641191899697283	0.0784841415391443
0.1885327793868498	0.4163341625833640	0.9367740803076854
0.8028415012082306	0.0379097272585492	0.5788236181569340
0.2174402721214319	0.2115390820680275	0.6176405811757673
0.1664791138025684	0.0558719978829199	0.6216010174099686
0.3163771013518614	0.2747581613012016	0.4172811610204094
0.3773273572070064	0.1105176148438803	0.5578748487575589
0.0428941357270293	0.8172963554489996	0.5812035393144608
0.0468677604334666	0.6696981013448189	0.1625687339640458
0.2085405022606594	0.6409487899399967	0.0146835108082031
0.0906139114625978	0.4637530625375047	0.2469374699141632
0.9758300159663286	0.5336429427104665	0.1562402070684042
0.6221409262152668	0.6134003720495268	0.7686100945875700
0.6871487152151003	0.0593165907855143	0.1366849634035338
0.6481930391857248	0.1823205950237380	0.1965482991993763
0.4712066170613913	0.0848166837116764	0.2435524327173657
0.5510475039482116	0.9356666803359985	0.3673224151134491
0.6372999276968855	0.6054581072066092	0.0058106272098903
0.8564394052166854	0.8520500293997058	-0.0209375567087182
1.0141564538667767	0.8406167097877068	0.8152377403002118
0.7799089001868276	0.7634631092707096	0.9378670286905966
0.8834870622186222	0.6429768842993542	1.0421325693576857

0.2356988814967161	1.0109525013356284	0.3991806093107175
0.8838972832515796	0.8553915698054975	0.3361855011308260
0.8672142513319373	1.0034091413032911	0.3634437585818291
0.8085628212744085	0.7733324649370461	0.5399592644524296
0.6680618525366265	0.8614770412926233	0.4738941893504452
0.9932514201739465	0.2311628010610284	0.4074133892186392
0.0357169958259529	0.3958052230543821	0.7995980280700141
0.8251932249093482	0.3701183241269057	0.9684297623757850
0.9853234761994633	0.5866351315704419	0.7500580729584090
0.0179493816671459	0.4468208853708612	0.9161336151237450
0.4265348873367522	0.4346225185498131	0.1889002420062200
0.4066937525615776	1.0351800495571621	0.7846517046601587
0.3639104070684770	0.8560727021548551	0.8145059347004887
0.5696613301185057	0.9248457555883711	0.7566789687010964
0.5363723270844316	0.0548856931764782	0.5939917793462359
0.4116120327554749	0.4384692993328396	0.9706109638416935
0.0246746496392003	0.5087696058454432	0.0135876465924564
0.5304071469704098	0.0175004487699157	0.4761631327430559
0.8058408768739522	0.4085156481492032	0.2173213012341880
0.1326095878661106	0.2357136084238048	0.8891241801151617
0.4106801277026624	0.7791266024836853	0.6140779345651119
0.2132484675617210	0.0952391783474938	0.8048816915345756
0.1884215970539688	0.6356798912379030	0.7800270076957218
0.9877469116963364	0.8495152086727193	0.0334667405590495
0.6042894120024380	0.2378810250368854	0.3756970075256438
0.8414690691595775	0.9642620180638457	0.1808285350297574
0.0607733346284618	1.0264300257404657	0.1027862636105344
0.9293939117058366	0.1662943936334428	0.3438030167417107
0.8110534938572111	0.2058736015416300	0.2635634968121993
1.0173414282710158	0.0535540900092605	0.8390354812314250
0.0679768809219518	0.9562253257277674	0.6050497375567140
0.2260385289743269	0.8680540287251549	0.6472836430302016
0.1874452398629032	0.8929331904695750	-0.0042142085489663
0.4225957719219021	0.6665310664087181	0.8511253400393555
0.2586977542317385	0.7402136552833688	0.8131880698730775
0.9184035301967997	0.1660465856602324	0.9630929773889669
0.6144276784693248	0.3973427221769739	0.0647592477945147
0.7338371445298220	0.3649956684738551	0.1423095058903223
0.2786451708328177	0.1018585933660867	0.9056016502260438
0.1099739040483339	0.2656218755270850	0.7451316503728824
0.9730244934638697	0.8146762866268900	0.1973818190267798
0.8188172282894648	0.9611606883705901	0.0526984777956070
1.0228037991241599	0.0312272058221466	0.4561710768639652
0.5159634747441150	0.5611318572798444	0.9675239938135806
0.1967168666764426	0.3434792180979350	0.1821988014207378
0.3600973857454121	0.1996986932879287	0.3145100490511669
0.8675375471140272	0.7029541252067566	0.8132911228752639
0.7082469640105478	0.8305418197183658	0.6694038054366402
0.1975087306708135	0.3156080268712901	0.3363838615266364
0.3509117238651238	0.2175480245634375	0.1699840188895043
0.8321111102625351	0.6758622879730293	0.7057345999751720
0.7212195438463579	0.8359365928009946	0.8010226921292622
0.5384607584481634	0.4574186875973737	0.6798413522660787
0.4947869178832576	0.4298888086474833	0.6463264607492123
0.6036636342784367	0.5760810529594259	0.5998309019456278
0.5149946375022891	0.5177326978095709	0.5367668510876407
0.6249932705149450	0.6630986684238488	0.4900839825614222
0.5811255379337794	0.6360429117006348	0.4567073308451310
0.6046751384349315	0.7360054365197739	0.3374129047786238
0.5037729740142821	0.7971697449684145	0.3478359580039978
0.5040231523236632	0.8780075769827510	0.2472191659553676

```
  0.3905485958077010  0.7356129442336651  0.4638625445360736
  0.3989192698977542  0.8970031950028570  0.2620374098670816
  0.2856793473245050  0.7582801186631710  0.4776036490718614
  0.2894108311567816  0.8359595717101220  0.3773522021359074
  0.5180826398297991  0.3629394071321230  0.7973026750511509
  0.6213394975947939  0.6927463567435137  0.2793540277138826
  0.6351563695912841  0.3446569123004977  0.7807823644604237
  0.6171199882469186  0.2769654769066430 -0.1319015287891273
  0.6823590433512579  0.3154228368048601  0.7325916829415833
  0.6955791886414998  0.4281432035331845  0.7287452242132859
  0.6892801144024312  0.6456753802816322  0.2756315775096350
  0.5372231837395997  0.6309513955403852  0.3315912779767910
  0.6508785978444893  0.7674989191446684  0.1879287063741649
  0.4578530730296341  0.2784999850623466  0.8508662496074182
  0.4720324659858890  0.3889842562833188  0.8485423336035492
  0.4442827014400432  0.3377858653946280  0.7072493467879886
  0.6379437042454951  0.6005634889289156  0.6237721573261773
  0.4791656232983292  0.4930799860444788  0.5139900542774333
  0.6765908117939827  0.7545305651179605  0.4284856141354230
  0.5164735913276659  0.8522512316703799  0.3793079853057864
  0.5879668558605708  0.9212575167294050  0.1584587912237905
  0.3878069340844026  0.6710555233990937  0.5422480233867971
  0.4008039119828563 -0.0421587834622259  0.1841417706152134
  0.2009006666518545  0.7116155031178076  0.5667828113644986
  0.2064873714998861 -0.1508190930117501  0.3888101306352590
  0.6889216088071808  0.8025598829300451  0.2780245346452397
```

IWV-Al-T6-H+I$_{dyaril}$

```
   Al     Si     O     C     H
   1.00000000000000
    11.6056394577026367    0.0835597589612007    8.7649669647216797
    -3.6825602054595947   11.3529796600341797   10.3282289505004883
    -0.2346490621566772    0.2412826269865036   19.0520343780517578
   Al   Si   O    C    H
     1    37    76    16    19
Direct
  0.5188173428632804  0.8890156842596173  0.5009763464994802
  0.1550471441862832  0.2320289891012318  0.0548430442072466
  0.1366782321919569  0.1039199420493886  0.9924984991910565
  0.1086445653817937  0.3129874112234635  0.2865737587081387
  0.2581438800205824  0.1332056387855345  0.5594347526209327
  0.1053384748093851  0.1109964903954346  0.7737421058572262
  0.3304920258411996  0.0949529751305273  0.2945333599831045
  0.0650951414850404  0.5568543702945574  0.1441870440123197
  0.0119363567002835  0.7660129058427506  0.1213059469073320
  0.2970668393076491  0.2860133508696343  0.0995939490852942
  0.5707739316913381  0.0508185964673375  0.2367004823387476
  0.7830030008217242  0.9993419709367360  0.1023972317092269
  0.2996273217840942  0.2715775574842631  0.3257294025927126
  0.8628288439443493  0.7559884547351670  0.9453788546353312
  0.8841192394102976  0.8862362033365841  0.0006662340121693
  0.9075767556621958  0.6695499965957683  0.7228928280695390
  0.7639220863342830  0.8470878430205413  0.4443556493635898
  0.8975946525198774  0.8766490019841144  0.2284059052001375
  0.6828779744347090  0.8884581443284260  0.7140839519890902
  0.9527618125571804  0.4273068509225394  0.8591954097129538
  0.0189048913364347  0.2278416989036279  0.8673095611311419
  0.7239509144056875  0.6964775728100285  0.9040339532686568
  0.4532828820021484  0.9454734938140735  0.7452219093776768
```

0.2357258031889679	0.9880178993013169	0.8962836316646138
0.7219496715212742	0.7100040235133444	0.6770401383862767
0.9270314185999894	0.5161265432742292	0.0843162118287505
0.1034239500590776	0.4698476084538830	0.9106362061089672
0.5104142267454207	0.0983139579307304	0.5020719037219106
0.9050640869963961	0.0882960172266851	0.4583371269734235
0.8793376629032731	0.1208007119899883	0.2877403330775401
0.0678026955454079	0.9338354025274463	0.5146356776744199
0.0903986381486522	0.9050529370056190	0.6795365436000061
0.4656437108904249	0.5428365431633461	0.8969222460905153
0.2939959354135190	0.7067349775207208	0.8726762678201246
0.5208993683875514	0.4779545363442076	0.0608722505481568
0.6834821227397301	0.3189236512634264	0.0895426391737030
0.7284123659005164	0.2990738639543274	0.2405740484192428
0.2420051682239974	0.7312773481607699	0.7227345114414635
0.1904001781327999	0.1628128178041162	0.0102032417435708
0.0180513734570233	0.1642421579803146	0.1766581352963580
0.2440575764416874	0.2263058433508210	0.0821104245504242
0.1704643168570011	0.3708354090056091	0.9527628707809762
0.7820604592544406	0.0206320484826742	0.5934746510443163
0.1790251918999475	0.1646843115462265	0.6407465177852248
0.1869551842986491	0.0095535889356839	0.6207750429990966
0.2842982232278024	0.2422396120463792	0.4316004651582381
0.3789259097122796	0.1179116853085706	0.5466398689918950
0.0171885627125246	0.7926396375892828	0.5975014145624895
0.0110388857476578	0.6286003793323108	0.1874942747358794
0.1911539779302234	0.6461949352735664	0.0097301157513834
0.0894941082831497	0.4427635698259223	0.2339948821841279
0.9716041721356276	0.5118394163861921	0.1468915265679480
0.6002321533569651	0.6025871120061148	0.7718949557067962
0.6549898893584866	0.0273506419421615	0.1491171716334421
0.6212361673067710	0.1766593438434297	0.1835345238455014
0.4364033038064272	0.0308387757620910	0.2812425385795316
0.5749731814186527	0.9454963966947963	0.3531413925470017
0.6001207911254153	0.5874025668459072	0.0125593093950588
0.8169511410614909	0.8081387522777728	0.0077354228139238
0.9676402704377309	0.8636955182399876	0.8031224840104079
0.7497412945164099	0.7124977121217063	0.9671135259393293
0.9162897298528302	0.6475736104855531	0.9997121013012849
0.2071794012813954	0.9872865513923401	0.3981228294637823
0.8417448510300773	0.8174513601984493	0.3599126138342847
0.8160702405451388	0.9904282220698019	0.3556700324047614
0.7887923163317421	0.7713771647191279	0.5417696750581551
0.6235211687641559	0.8139516165961354	0.5082977814210661
0.9771008418496405	0.2138976976912904	0.3939527928953265
1.0043007725115536	0.3594699838942219	0.8118362508221533
0.8122693614878439	0.3538705086590520	0.9737709733371982
0.9672549771491671	0.5621025639476885	0.7523208104990664
0.0215805578881631	0.4265443433110839	0.9019747424991066
0.4049146479775576	0.3976631021045542	0.2002577172426686
0.3705242898282492	0.9903056916638122	0.8137198119951168
0.3701671196893909	0.8371422719192052	0.7951087797702486
0.5497558306089959	0.9030730009869770	0.7715572461984812
0.5246800823538250	0.0514155009736133	0.6013158985090040
0.4053846137889425	0.4085175678758722	0.9678145859698024
0.0200629043896231	0.4822426905516354	0.0076380951719328
0.5301835483121641	0.0092511544081859	0.4830671598410664
0.7967297688874254	0.4169955668288152	0.1897907282161314
0.1027475781190587	0.2047131360193053	0.9050540439632977
0.3744475937002432	0.8032749564212474	0.5986911681733689
0.1815308232870730	0.0503380865397790	0.8238004328477579

0.2038943451549290	0.5968276221068092	0.7808870417597581
0.9528105464543768	0.8077355031668162	0.0562752474478920
0.6096075745199221	0.2290547904599568	0.3763685298619725
0.7911298384131124	0.9215572109276897	0.2128383180073636
0.0150679068204938	0.9921256102676010	0.1214783478310185
0.8706140820794043	0.1159581079845633	0.3794347325495120
0.8144120419776616	0.2147881734075213	0.2347456997543413
0.9807715824096110	0.0106049288797294	0.8630391054610722
0.0582923375701137	0.9553782909627807	0.5911084069346326
0.1433138622830001	0.7940634622427843	0.7042222767097667
0.1498503173198807	0.8507902904692459	0.0208722783947261
0.3823241416004031	0.6248420367647267	0.8675467310250050
0.2306441056610208	0.7211520465891533	0.8195514356683180
0.8893955236442208	0.1242443567569595	0.9863102843047127
0.6030915066502532	0.3934892077788203	0.0477098394297240
0.6938716268887339	0.3433738950183253	0.1552127528481619
0.2369254834164652	0.0590391047171468	0.9304100759023414
0.0770020354771861	0.2204956759908554	0.7682604340538935
0.9318624627433134	0.7768208295420971	0.2201919201564488
0.7844494883933399	0.9193684573109696	0.0769283083584645
0.9907699452537133	0.0019580928589265	0.4664589366542928
0.4746792196284354	0.5348509773910657	0.9824393380757906
0.1905380036639151	0.3132144447969477	0.1794492970117235
0.3351134649651809	0.1628041371792730	0.3301378976691304
0.8364275241860528	0.6651345016177076	0.8322039711498587
0.6896628926986472	0.8120629397340177	0.6825668682928254
0.1753603717555096	0.2818840986992465	0.3425558398738030
0.3515030333686750	0.1928525095073624	0.1681905744121774
0.8119751164816998	0.6542231358772803	0.7106133392138869
0.7085892451526943	0.8182425721707733	0.8101918683795524
0.6684106244484598	0.3928315287792294	0.4768528874916861
0.6568380679609767	0.5056443603631793	0.4336353329687632
0.5700019912409710	0.3063927825010617	0.5394262201136448
0.5502559744323149	0.5312994620033772	0.4527334801533589
0.4639714815728102	0.3319355229847968	0.5584795804029998
0.4517771051166623	0.4447163284180645	0.5159924025961657
0.3357117284024557	0.4692652258304846	0.5361513191485012
0.3304773583469721	0.5043010543434268	0.4423674254399126
0.2184375835012408	0.4941952803496142	0.4684395646723299
0.4333372920710379	0.5484526690239594	0.3300491764840430
0.2093614757948889	0.5278968714695959	0.3844270059607460
0.4246763989738710	0.5823731626906383	0.2455810529714955
0.3127820192625724	0.5723496109881967	0.2723481024903678
0.7816783085983429	0.3630144345862813	0.4593090545161120
0.3059656008279029	0.5613834548273346	0.5469686189924319
0.7635030755879949	0.2671479122467939	0.5775593948730448
0.8474941692805545	0.2465880625737703	0.5612223360707325
0.7330147479065343	0.2974068093883637	0.6238408202380272
0.6956229566128496	0.1840744039562499	0.6380350833808976
0.3067461730578864	0.5356478738339595	0.6162933008302030
0.3709416876432737	0.6504022021037293	0.4614972164273247
0.2168919529540776	0.5682737843046443	0.5705562864900205
0.8508328408032908	0.4444882216316679	0.4000624562196188
0.8156715722734984	0.3316753272638429	0.4133813823467365
0.7323526746860409	0.5742676612114659	0.3847850414682101
0.5770945821394246	0.2181359043088856	0.5730794634800367
0.5439517745264786	0.6200738086897464	0.4179404289697533
0.3888336656083169	0.2634449672234224	0.6066592507683271
0.6425279028906907	0.9136718711531960	0.3290745842347298
0.1375301015650088	0.4586980316377866	0.5561969615313933
0.5210260503916988	0.5555021549411522	0.3088006957619350

```
  0.1214060313978200  0.5190872635449643  0.4065220672729840
  0.5058567714984634  0.6158510153553074  0.1590180785949210
  0.3059415279052789  0.5984309311858009  0.2066176566762455
  0.2636057615917285  0.3843847678264882  0.6224081140425856
```

IWV-Al-T6-TS14

```
   Al     Si     O     C     H
   1.00000000000000
    11.6056394577026367    0.0835597589612007    8.7649669647216797
    -3.6825602054595947   11.3529796600341797   10.3282289505004883
    -0.2346490621566772    0.2412826269865036   19.0520343780517578
   Al   Si   O    C    H
     1    37    76    16    19
Direct
  0.5265512988942233  0.8908907473786531  0.4778723534211671
  0.1559727877119475  0.2587939069217309  0.0355415260929016
  0.1567842578785379  0.1375948712009600  0.9611837868464549
  0.1043663794613825  0.3342358999225973  0.2762133494883383
  0.2614549927297859  0.1514810331868288  0.5406495356055252
  0.1279698137374473  0.1462530244853400  0.7423061466445199
  0.3383518320696080  0.1205393689842938  0.2725807505991033
  0.0646157618178167  0.5735949852564877  0.1320464339713130
  0.0298082601059487  0.7983648794535343  0.0892108494983225
  0.2897641291934106  0.3152325673362206  0.0864064137784108
  0.5739836645216944  0.0670718747176957  0.2239943314018668
  0.7981205401498421  0.0306313899394864  0.0753468451303724
  0.3049158857795790  0.2938516101160498  0.3063710662775818
  0.8684292079054043  0.7696889725880697  0.9336154519433251
  0.9046885359293639  0.9252292751965587  0.9640341503417563
  0.9133963899806395  0.6921531419435297  0.7010141013014251
  0.7779745061589977  0.8627498768907950  0.4197320898963631
  0.9216752382092517  0.9098923941365972  0.1937616817349637
  0.6853906759621941  0.9047048085712412  0.6937842175664932
  0.9520444517464494  0.4480570055664592  0.8428012609914592
  0.0350730100436144  0.2586565763248059  0.8383217359080946
  0.7295728912225246  0.7203503868154110  0.8836451718405471
  0.4537213101350389  0.9679376231616855  0.7247458617689488
  0.2534718619290620  0.0185902008149011  0.8702685245083970
  0.7290358136833368  0.7251701036185371  0.6574744111236244
  0.9076673816497809  0.5212299398949702  0.0937254945850416
  0.0849499870207582  0.4912872391539750  0.9056123345977798
  0.5086230407355082  0.0926993772844756  0.4894240281284269
  0.9110948176548797  0.1086874982599612  0.4415625643086613
  0.8862338512371354  0.1335148405756008  0.2730165319044569
  0.0756729761621529  0.9526239309754048  0.4981996892746354
  0.1044602002741954  0.9201222768100197  0.6619151317515857
  0.4727710189141056  0.5721307864244981  0.8792660409704020
  0.2957271895450884  0.7284377857998234  0.8567852905731306
  0.5284446586817675  0.5005018854584123  0.0449934796031864
  0.6894163617042927  0.3363240920640908  0.0708442729501243
  0.7317480963044128  0.3057210448829972  0.2261177155760499
  0.2579822526961384  0.7470950811846657  0.7021224112697831
  0.2080370053578171  0.2041407272499808  0.9747644830448987
  0.0235161391956598  0.1695968360751015  0.1626335693830860
  0.2472346028731154  0.2630929728171880  0.0567101299423274
  0.1551350498408580  0.3950859247413546  0.9468549964775106
  0.7850391749988960  0.0366020388523302  0.5760680072620452
  0.2142735767761504  0.1968311204627034  0.6090837933564046
  0.1554711964702239  0.0414764189566585  0.6167572075519092
```

0.2903964789281948	0.2651898776556927	0.4118129977159757
0.3755478768233441	0.1054689557973943	0.5285462363530631
0.0229299169899106	0.8146312754751969	0.5722179884734271
0.0201993016143401	0.6589860117387396	0.1592768029464481
0.1911821834666734	0.6502645279167999	0.9986693496302759
0.0840621307068856	0.4628391039871914	0.2290727332751502
0.9642463453557704	0.5205400286409089	0.1464051846010360
0.6048092783413873	0.6181336932939371	0.7571531392616004
0.6704062744500727	0.0558899858319494	0.1257760329536348
0.6294978836042021	0.1944755042759153	0.1696329706458599
0.4537489854172780	0.0755122148221837	0.2326261847226760
0.5470700193091905	0.9609290971850795	0.3493608738477296
0.6112524378087275	0.6032950663562563	0.0019266038980107
0.8415102641824912	0.8506587754820890	0.9649525052451148
0.9961868967505356	0.8323661517921972	0.8042710153775252
0.7633550280662850	0.7522893644281468	0.9323516331021251
0.8715899343007955	0.6427711047751007	0.0335283098302815
0.2168922719809169	0.0069125105963794	0.3847960606691135
0.8715168063003838	0.8483348609376782	0.3250079322799152
0.8128076292558336	0.0010025512585813	0.3521172287524010
0.7905894439722533	0.7780961223177657	0.5261942815680313
0.6422222099796597	0.8182120132154549	0.4733226173579976
0.9730511366047211	0.2342667243035609	0.3848174431522359
0.0187090912561626	0.3890145536480031	0.7870372872365705
0.8101274014793620	0.3761540753596594	0.9464828231193285
0.9744542818144512	0.5859651191970122	0.7337760632892405
0.0035701448924908	0.4398283156355560	0.9022374796828878
0.4054794579974099	0.4237291652831578	0.1812617166370146
0.3884909659791343	0.0233561653671937	0.7849365885964369
0.3513776187555775	0.8610631853841668	0.7910392032832511
0.5516806537079048	0.9172254137130926	0.7475905390322081
0.5192873259847196	0.0693945389563769	0.5821587427059010
0.4002427569197267	0.4383447065560254	0.9588441526661646
0.0029831108555398	0.5072899679268793	0.9994570184195484
0.5338343007094242	0.9881473801330178	0.4909575887689149
0.7862164092079610	0.4088909003277074	0.2032309205727610
0.1210374021239687	0.2340921489182430	0.8745908356855445
0.3952093502912341	0.7725410274258327	0.6060194459818062
0.1944836403867641	0.0823613503551706	0.8025561129052022
0.1803745353696342	0.6191078283834153	0.7731408056740063
0.9712242897175873	0.8449786401268590	0.0225992028934936
0.6031724753838940	0.2235696491027941	0.3572268692775108
0.8186771616562053	0.9524346678257510	0.1765896319912332
0.0389600504231838	0.0236282146784151	0.0913386349069584
0.8855351728221255	0.1369504389623774	0.3566224830683622
0.8246043409751963	0.2278364518270365	0.2161093708753455
0.0037801748268121	0.0485610291814847	0.8262558280879677
0.0551497452112544	0.9569673297961531	0.5895228505529900
0.2040037688698415	0.8521644309462674	0.6395355973111975
0.1688395227579443	0.8798169050183684	0.9921106924779876
0.4005901373109307	0.6642641091444422	0.8399296587332042
0.2364483466188378	0.7364881582258058	0.8032705746971491
0.9055011812028342	0.1565231614846958	0.9556633347006589
0.6022736648132395	0.4094856453342082	0.0380992378082055
0.7188776741438132	0.3698078929857573	0.1218057093135280
0.2584066161296339	0.0898744519835605	0.9031589916743311
0.0970669710904382	0.2568357863974539	0.7347368939824286
0.9541305843705928	0.8071712997196230	0.1893469508425205
0.8032480215412920	0.9566235569160590	0.0414533825230202
0.0030746188199420	0.0290648508557831	0.4465067146626722
0.4924415137088677	0.5687333875177821	0.9566302783190292

```
  0.1790268279277704  0.3407877717413635  0.1650862354673894
  0.3443902127194035  0.1867287274062022  0.3095670810859078
  0.8412920039948989  0.6926269473098252  0.8070104650358445
  0.7032979359966679  0.8320104028111719  0.6556590298765782
  0.1789364563450757  0.3000347551420818  0.3258122732023625
  0.3382571396719765  0.2182562857804186  0.1581698394396084
  0.8197304708912646  0.6694977633345890  0.6901552638067752
  0.7014106486096059  0.8327682657742019  0.7964998836398087
  0.6380944951053159  0.4571807718623172  0.7050020599756772
  0.5229528163075665  0.4665483778223473  0.7375349556690917
  0.7356371236491469  0.5256856563914792  0.5827896983262542
  0.5045938361167349  0.5404738675896069  0.6530741817052511
  0.7175295574718921  0.5959519109964380  0.4978426424781650
  0.6023938921212955  0.6120172034600828  0.5270051754300678
  0.5849908332259998  0.6391632591573067  0.4415170097492392
  0.4885510621452679  0.6928515962262297  0.4327095896900778
  0.5199273011698177  0.7804652581344657  0.3176113812975803
  0.3682164751511161  0.6521654973767949  0.5305984511479025
  0.4355880297235191  0.8279870198817927  0.2999041717767170
  0.2837029587712629  0.7009564177917229  0.5133207957283548
  0.3167699368195449  0.7888393173756472  0.3981812281715188
  0.6520690268380054  0.3752968957053789  0.8014957155766849
  0.5685249731069356  0.5220497782745104  0.4724169691959359
  0.7789297499686905  0.3715563060706157  0.7610782391817321
  0.7775758317213772  0.3101226992869769  0.8391905546469005
  0.8182199353558418  0.3407900077344198  0.7143575290295028
  0.8384338674192715  0.4599713705140334  0.7004496117527454
  0.6421407740272900  0.4846444489394321  0.4706863337081786
  0.4833859766940567  0.4557662361773016  0.5623635808693919
  0.5658824597358585  0.5388167356235957  0.4077678001341927
  0.5923686915813013  0.2851516365433608  0.8656864690973709
  0.6130577602471371  0.4010594750838887  0.8527515132479377
  0.4473143643435014  0.4142339087010444  0.8317920935120304
  0.8254041518403671  0.5204368329027320  0.5553772694310236
  0.4157529390199066  0.5497740041494950  0.6812010123115493
  0.7928179462474370  0.6432925000272905  0.4044016021210390
  0.6196195304978882  0.7100078892386604  0.5013991549455720
  0.6122691935549504  0.8108665164043044  0.2403540664021345
  0.3370369702890788  0.5814686427235891  0.6222615762227565
  0.4634138867962152  0.8966348995715032  0.2090986303894198
  0.1912487670888393  0.6696666955871085  0.5905644846183452
  0.2503286778654417  0.8267075319535808  0.3855441473004015
  0.6712045684100231  0.7027875502756992  0.3512583277000607
```

S4.6.- MOR-Al-T4-Dyaril

MOR-Al-T4-DEBH+B

```
   Al     Si     O     C     H
   1.00000000000000
    18.1842269897460938   -0.0090872207656503   -0.0001607598969713
    -0.0105508984997869   20.0691013336181641    0.0015994716668501
    -0.0001797722798074    0.0011982563883066   14.8440780639648438
   Al   Si   O    C    H
     1    95   192    16    19
Direct
  0.5905661500811905  0.7186444533961915  0.6401787360978148
  0.3154672775491401  0.0742089290082887  0.0301942962047228
  0.3038699076161527  0.3195947060720813  0.0333337290159399
```

0.8153922804424074	0.5764098975780141	0.0297850986984030
0.8115491639725249	0.8014857208897774	0.0304053409840285
0.6930184312455543	0.9271395549924453	0.2800549273729448
0.7077737523666221	0.6827782545503168	0.2797485599371868
0.1956951667774249	0.4250490688938687	0.2819964846113538
0.1962190738147134	0.1997651223564684	0.2803015210823945
0.6943694064448493	0.0774488338370640	0.2320734332807567
0.6955732245140994	0.3029493121022762	0.2326640683474937
0.1936350207758762	0.5744423527983069	0.2333761585988535
0.2051521836603146	0.8187928717580001	0.2295580837444788
0.3132955264726707	0.9236448741854923	0.4829193831935717
0.3103787983584606	0.7015816176338656	0.4837453054160482
0.8145532211970431	0.4295596271964327	0.4838514924815632
0.8027574898817166	0.1836066376749538	0.4799829551275948
0.7094211820353824	0.9187070691064745	0.4880178871421828
0.7010177694490273	0.6864052483489282	0.4874011394722079
0.1887266660069923	0.4357715208784514	0.4875788413052617
0.2097760410306283	0.1891413876819064	0.4871073670936392
0.2986017538537025	0.0831136461999654	0.2378529522372195
0.3114361846602985	0.3167572607379083	0.2408919313624918
0.8221297940796435	0.5674551134085914	0.2328255562092306
0.7996594867258215	0.8138475047709252	0.2357535822621734
0.3216049389695004	0.9348291183597508	0.2771991594914036
0.2973508147461237	0.6900780236925724	0.2766673942434181
0.7980411230317360	0.4202371485205881	0.2755579076546514
0.8106954247445918	0.1853653569346795	0.2722061123719232
0.6879465362509322	0.0669010443407988	0.0272706273589212
0.7095233468732555	0.3137592664223918	0.0264877126689319
0.2116963973192109	0.5854765406111196	0.0257070378526488
0.1970356221339788	0.8166467766963896	0.0226993959407267
0.0858944163968622	0.3790855288321079	0.1305300743437379
0.0899550014334075	0.2240124862089750	0.1246369904472671
0.5913519977884482	0.8749806878455949	0.1355930759895782
0.5874894499826505	0.7151857063458712	0.1420192724541382
0.9220740362234935	0.6245491605289203	0.3801750752472294
0.9189029247534434	0.7805856624718767	0.3759320637275502
0.4169859826365616	0.1252103775886617	0.3874398990921171
0.4204314150563410	0.2838407153377983	0.3916891837409629
0.9170960327379415	0.3769994137212310	0.1267844546938990
0.9205107389602776	0.2178609061208650	0.1225480277712965
0.4232628135035044	0.8786476696589018	0.1351659404357100
0.4191593617521673	0.7237908491052016	0.1409379862821525
0.0916683135251948	0.6265880067508978	0.3768619356613307
0.0883371455131692	0.7855401056428387	0.3728029815810504
0.5858005156653882	0.1234044657054486	0.3847993653283189
0.5898459214252199	0.2782722138176880	0.3892328035810301
0.3146041455701462	0.0730796628408637	0.5305550876486628
0.3037741620586834	0.3184309195032881	0.5336607452023587
0.8136243971865116	0.5783712754043631	0.5344024575572427
0.8143034689773438	0.8026056119274440	0.5293419465976271
0.6944196402787615	0.9301716068903795	0.7794440096056947
0.7070780895718950	0.6812673810146503	0.7852853636346763
0.1953103724009980	0.4250750858365631	0.7819373429727188
0.1949614980536966	0.2001375408685458	0.7801132930818190
0.6973516379666171	0.0775032195138373	0.7316104210088101
0.6946720136679330	0.3033389561452137	0.7322994434992304
0.1935493758372405	0.5746939104694413	0.7342477530870738
0.2035749831165240	0.8200177633744968	0.7322607701360760
0.3128314486621483	0.9244394242837283	0.9830341896234024
0.3143149392090894	0.7011480691753593	0.9837503454778542
0.8158479262506451	0.4275155085476438	0.9815709757794026

0.8034747989962742	0.1838693311772480	0.9803995706140548
0.7100801549380036	0.9187155680242687	0.9869044275056824
0.6971073962217735	0.6830508538225945	0.9936411592939930
0.1882093820356678	0.4366565103832231	0.9874599423022100
0.2097479871343388	0.1897218341370310	0.9864976169225432
0.2987435009908711	0.0840897380230246	0.7385678704450999
0.3111126055586612	0.3167827645094696	0.7412182716775492
0.8248806113361578	0.5664286956654173	0.7396210414554735
0.7974011525141279	0.8114913526910627	0.7369350626091388
0.3207806288303611	0.9355998135810910	0.7784490723652088
0.2978395346261781	0.6904303742822400	0.7774376910018886
0.7988243789832198	0.4178760831179687	0.7750089951088074
0.8104350950469162	0.1875263782378852	0.7725123189059393
0.6880501782355619	0.0661851773041301	0.5264440620324727
0.7096610016518180	0.3131344899304262	0.5261014886857461
0.2104461163531857	0.5839952174564479	0.5261390343480884
0.1951535763635306	0.8168132062465298	0.5240720431835305
0.0857484392681016	0.3787134917055595	0.6297741604071043
0.0889291301485079	0.2234727224972487	0.6238905957624962
0.5916672947152285	0.8789991679724388	0.6393825841534350
0.9252753022439346	0.6228763895914418	0.8790331257791172
0.9179869738966749	0.7771652045172688	0.8717988680083640
0.4178404659260871	0.1265078543553457	0.8870851944791821
0.4209265721050491	0.2849340291853817	0.8918787162965686
0.9174930630984393	0.3767230180641378	0.6253569440473387
0.9199166623949123	0.2185291595523780	0.6216014052459147
0.4233899732513387	0.8789827956435832	0.6354640710466370
0.4187851407201240	0.7241898334435539	0.6389044123651100
0.0923891564027585	0.6271659387647662	0.8766078014078130
0.0869679938849943	0.7850860957436112	0.8723744162194983
0.5867434866441184	0.1237675677008819	0.8830892510721416
0.5898092243459971	0.2793559650978670	0.8883063179487908
0.1340397554580686	0.3871136463403032	0.2220089838669697
0.1197603159590101	0.2147323847156788	0.2274699188738843
0.2515161018537173	0.1177959077755916	0.4808750094259276
0.6156119211815778	0.9142751971499765	0.2270219495049513
0.6319391000277409	0.6779092319623469	0.2219734417309858
0.7409741955516621	0.6244297774909232	0.5407958671099398
0.8760260658070992	0.6131498945508677	0.4720060051148086
0.8940232807784561	0.7935193667238361	0.4799614789546819
0.7573470905224005	0.8851045529142375	0.2288659713973059
0.3920603887503183	0.0842429449076975	0.4771515395549439
0.3758579619308677	0.3238243915773110	0.4696754665987802
0.2702009275369544	0.3802610341729877	0.2868037279600588
0.8931183973295608	0.4164363375122943	0.0353767570671532
0.8760849138316490	0.1789457149365407	0.0436272272403239
0.7691626887301691	0.1219043626747911	0.2267637719613354
0.3726037638955450	0.8863046233750934	0.0456874549557599
0.3906376068510464	0.7155394879898710	0.0372684332046762
0.2576181872561101	0.6175192736184160	0.2838146217303245
0.1163896916723003	0.5857628646668990	0.2865310909420842
0.1351451303506738	0.8250783965837848	0.2963906670073369
0.2379739560388480	0.8802377064728261	0.9765915021947827
0.6339321599319213	0.1159189179349429	0.2934487628627059
0.6193342655080980	0.2879831982353168	0.2864672288318111
0.7517014042770944	0.3850869113489359	0.0331116957886857
0.8927492235777882	0.5765532914984302	0.2993040311479087
0.8669143482974311	0.8218518876815597	0.3068072462379146
0.7849582857225624	0.8768166288460738	0.0061271143441052
0.3651386956445645	0.1076254343233402	0.3022627223051225
0.3886890617367109	0.3045882019191922	0.2929868673335929

0.2461746852727242	0.3771664854199296	0.0054218867247720
0.1164793678235218	0.4286406633744209	0.0519232562598294
0.1409155276138206	0.1818759416583180	0.0551968082218227
0.2234630060454598	0.1242740553736998	0.2590480907774549
0.6438029678976539	0.8941213796589517	0.0518152036487944
0.6181118949016373	0.6907994332370133	0.0443259041413314
0.7667510763849074	0.6296817195633265	0.2409128691329795
0.1431968959567976	0.6063212332367869	0.4616339973514987
0.1172586134421060	0.8065620868917439	0.4726093401126418
0.2666067014569438	0.8734981263345355	0.2564127211897032
0.6145827011837457	0.0742729973912009	0.4641800160396081
0.6399410306720519	0.3216106516953549	0.4584349061323434
0.7232231848777894	0.3783088008775369	0.2548693712835892
0.8648148579653812	0.3959552534168640	0.2110349048974857
0.8883010875092967	0.1973111760469399	0.2208264304762712
0.7427603449738966	0.1282161679633742	0.5077379787557671
0.3949528719267865	0.9282013689836425	0.2146896660401835
0.3674088813694422	0.6817309831553183	0.2097690076328587
0.2857816710029472	0.6245140402071669	0.5034300760783270
0.2599834820435435	0.2508992499469397	0.2465107419455835
0.7445312293025326	0.7509245955138983	0.0063358974244328
0.7660255576415349	0.2571598833700939	0.9902234388590390
0.2404368541823725	0.7443887843194746	0.2357414199083964
0.7418552000818015	0.7573932115491200	0.2697763364819675
0.2659402126494159	0.2467869843694230	0.0234941589639408
0.2500704968145533	0.7515349962304233	0.0168156724136000
0.7593799549452086	0.2514133154085398	0.2641683203353162
0.0010786204079756	0.3977173649094405	0.1521052541807747
0.0064358364850398	0.1965187576287128	0.1184341418168083
0.5076878537844020	0.8967159994592441	0.1102944083550785
0.5014673626681790	0.6934781400495797	0.1474620413147638
0.0076148769018834	0.6066852151753379	0.4004570823866312
1.0028905157590391	0.8063436628015896	0.3637513556180620
0.5008029681826214	0.1054640337797800	0.3620261323045106
0.5061074516638679	0.3057354622705643	0.3954193019024068
0.0935303440390633	0.3024576667966154	0.0956759287048303
0.5958035144439403	0.7952412157798616	0.1533559432283903
0.9136558829965080	0.7018497182385803	0.3491520624829111
0.4105654170015853	0.2045485794612245	0.4087339530704978
0.9116352376983493	0.2975947390014561	0.1071996287834579
0.4171231779243855	0.8018599945246931	0.1705224087165530
0.0997105016108213	0.7058464600398040	0.3574419367788155
0.5928688026814356	0.2003697463584617	0.4192040304633149
0.1812989612344679	0.2103220257197939	0.3873124687746953
0.1629306925129096	0.4356052386159199	0.3827481419484950
0.6896171557907599	0.6639453994867455	0.3827089663467213
0.6851151102969273	0.9041914719681087	0.3840465713653267
0.8321750041385498	0.7950897184473360	0.1367716689089854
0.8509017228222516	0.5632383713242035	0.1289491956304024
0.3305615678001947	0.3320810645499766	0.1361943892740171
0.3244320216310103	0.0969820216039034	0.1344671111642729
0.8292927455740431	0.1712193737069309	0.3771363593100641
0.8237018672459163	0.4076338918299270	0.3792280939282687
0.3247467098607234	0.7125247599431098	0.3763212250028346
0.3465587760492952	0.9339406714501309	0.3821598198119010
0.1778071952046381	0.8319616630827897	0.1272394549395050
0.1851303168858162	0.5973895724317084	0.1290994904030103
0.6784427282365191	0.2941795212152195	0.1258088573956905
0.6604494384666157	0.0685500010354230	0.1315067159730072
0.2170525440016191	0.4962259885614134	0.2365300929196382
0.7142535972520367	0.0058201560829010	0.2761393894208439

0.7939658227046867	0.5058317840809006	0.9824045968223740
0.2917572922560130	0.9963080835099267	0.0265776322835979
0.7795936209597873	0.4984398481611484	0.2585288310214183
0.2803251962036492	0.0047944972409120	0.2542716585312639
0.2295238445567034	0.5069321631094335	0.0082725790295757
0.7288644359566281	0.9967987003463413	0.0060837972079402
0.1346219736275005	0.3870075319990973	0.7208669231036171
0.1186836525918890	0.2145463688838459	0.7268379222481838
0.2524337314427718	0.1188222716569482	0.9802528346081196
0.6171892775566638	0.9258753595555679	0.7248855289690401
0.6392281123017092	0.6713008541703486	0.7189158632798874
0.7407802335802718	0.6203522031690579	0.0385736284784132
0.8751589754001281	0.6159736869346727	0.9689387269458030
0.8850627731684350	0.7834994904084954	0.9734987115721560
0.7562094059633282	0.8833369740729438	0.7313423279059027
0.3928810408906213	0.0850981848246539	0.9765946391249308
0.3759677870128010	0.3255703870387276	0.9691174454184172
0.2706113446229160	0.3810025529999196	0.7870975426166104
0.8924785831858973	0.4185082514947124	0.5363449455546871
0.8742289928130369	0.1781855421634944	0.5450984440024016
0.7698937255797468	0.1248607714168212	0.7242588858088362
0.3744921261745827	0.8870115040306967	0.5441494177381117
0.3860511700724663	0.7180066523936615	0.5358676362972744
0.2565126097330763	0.6183742450891900	0.7854402815048547
0.1159535876005514	0.5845273747727637	0.7873303818174753
0.1311399683266481	0.8264749858641645	0.7954897255181670
0.2392309784104359	0.8782610871586699	0.4770358956738360
0.6353094997700608	0.1146852456461723	0.7924422675038439
0.6176044793283778	0.2896628493904433	0.7848629945091016
0.7514678355828518	0.3844758449831885	0.5330402302877407
0.8995144167576389	0.5708567766826976	0.8008992252413235
0.8697707534091639	0.8200962618004185	0.8008105829539065
0.7840757202943206	0.8777201994774786	0.5113907053857057
0.3652688525636982	0.1103229623702394	0.8020144930170668
0.3883940439912230	0.3045171037073154	0.7930792888559135
0.2450193062742575	0.3749644708017621	0.5053134796476039
0.1161230151813843	0.4278629382474629	0.5508509823741559
0.1398648866511508	0.1815169972207869	0.5542720157947286
0.2233382658403983	0.1251988713448373	0.7583918942516350
0.6412845394312997	0.8985205626521593	0.5515949529524740
0.6240747597062873	0.7033124814507004	0.5322338868041613
0.7724865077142380	0.6278010163161527	0.7654564618985523
0.1467963908539866	0.6111569260402214	0.9602078967986011
0.1201515499525000	0.8025720474997785	0.9713457474960467
0.2632138467968410	0.8758353546984561	0.7603311663148866
0.6157209481885673	0.0751093489262397	0.9630945962130391
0.6408154813941035	0.3218367644352803	0.9573606283662736
0.7223121906968406	0.3790296098238314	0.7523247899345671
0.8648963476542953	0.3923213907821025	0.7104736285842821
0.8876570912180205	0.2019139810740542	0.7212439218665886
0.7446550461350302	0.1275055884559944	0.0092271672114524
0.3922696745986345	0.9285288741495643	0.7138765847972889
0.3640688723385698	0.6820498424316284	0.7059788803982917
0.2881409805227080	0.6251430518452438	0.0065352598928512
0.2584920027588862	0.2519314406525967	0.7473047644337583
0.7576541060817243	0.7495973169600847	0.4856599609997114
0.7660269885165397	0.2573509788948379	0.4876615510840382
0.2395144564610035	0.7462788036548280	0.7434583194751067
0.7437810817953172	0.7557291505480389	0.7808347911429193
0.2663037175674134	0.2452045538202893	0.5261359303064022
0.2432916567649427	0.7487786962224905	0.5171965218377510

0.7578435768108439	0.2528236515404387	0.7680196650279743
0.0011222184637288	0.3977149585200550	0.6519832515280197
0.0054532797900826	0.1957039831803121	0.6180318719744019
0.5071913216551291	0.8997564872391327	0.6135993998198024
0.4988533911678686	0.6929103363657393	0.6438789207589667
0.0098769688999678	0.6058279561870886	0.9059877392177501
1.0013007999217729	0.8063443706429332	0.8709717667259350
0.5014399581911096	0.1062250748526407	0.8610604521158104
0.5063232672478544	0.3074768577181202	0.8960826118363319
0.0925585273701117	0.3019610624627043	0.5953684889028232
0.5993925139885131	0.8025898736837090	0.6659017877121637
0.9188884023402670	0.6989188034258184	0.8415986075635861
0.4120864259439678	0.2056900214021790	0.9100395720649346
0.9131033788541021	0.2978843084041906	0.6012815838228646
0.4147383566712696	0.8024124522948617	0.6700407417592030
0.0969194637530585	0.7058303040602214	0.8522755617912239
0.5934764365449310	0.2010388029491077	0.9165537498157376
0.1795032969697105	0.2099931674936820	0.8871372459636201
0.1620203058584513	0.4347992895710873	0.8826602751815769
0.6796237157422974	0.6686999459124007	0.8889827034849141
0.6831127774474389	0.9078031821954844	0.8837581868170606
0.8244478335885574	0.7896149298786348	0.6366868760291846
0.8487912831563796	0.5676302994589324	0.6340924975490578
0.3302063968535933	0.3325065623429760	0.6365810918646951
0.3241540753546977	0.0959533171864501	0.6346953246191065
0.8288761702158037	0.1716474144542956	0.8770732818557584
0.8240089809652297	0.4022929939369671	0.8782320825559892
0.3299800119104895	0.7097326020520828	0.8766236798036006
0.3476558197352036	0.9337812869005846	0.8830796198241803
0.1781590039752599	0.8322815285985048	0.6289711460797349
0.1854768158030765	0.5974541503182194	0.6298964219452778
0.6796127121034079	0.2922602569514541	0.6253866865126763
0.6631933140599783	0.0664356773538985	0.6315833225610519
0.2177137808713283	0.4964514740696483	0.7376678001224682
0.7224573867666623	0.0076059741284032	0.7773557687573555
0.7899244822837523	0.5069652752522567	0.4904177016885488
0.2906718884982566	0.9952850028614552	0.5263666795229953
0.7825459320738254	0.4964888636728552	0.7605202782782438
0.2802881459114651	0.0063676236603395	0.7582413618114165
0.2300616561697882	0.5054960289293640	0.5113985708164064
0.7298408659619580	0.9974030695069461	0.5009998913133017
0.4497516323080268	0.5339078745314096	0.9441580698633644
0.4009809824149894	0.4951751455167356	0.8909018298113718
0.5028946437458806	0.5744485941543901	0.9013247948803146
0.4043874993582410	0.4970536973447486	0.7983476095362614
0.5078011920531275	0.5763075938140267	0.8085037098991108
0.4583049441353027	0.5376395111576929	0.7544288144045584
0.4639427477909300	0.5421785117134782	0.6609194956367330
0.5230949657547027	0.5902667902721100	0.4269883037002438
0.4574067404949177	0.5861302067578782	0.3783196935801762
0.5655988542292625	0.5330412657442812	0.4403241205485438
0.4337181969527122	0.5251430694228321	0.3431106342720794
0.5420722105320289	0.4718450302785396	0.4053721025316304
0.4761516616781872	0.4679260563756211	0.3567145121235222
0.4463848454419215	0.5294876450954283	0.0445271004757772
0.4180092032843505	0.5103187273178766	0.5929466176619679
0.4902709828770829	0.4683913158240514	0.0783620903578733
0.4878947315398264	0.4664693757484653	0.1523255166211875
0.4674270580925680	0.4218720864844248	0.0510101938000569
0.5482345476508608	0.4719521307924903	0.0578343217854277
0.4537293596805098	0.4830187827082734	0.5448636594794790

```
  0.3741322516363615  0.4781250254187157  0.6185952757679651
  0.3946675023337645  0.5498163848030336  0.5492139833996910
  0.3886573543632239  0.5245886877951883  0.0658044831176358
  0.4691895292094748  0.5750542130730634  0.0747512003407686
  0.3601971026732678  0.4640628284983740  0.9245828971202953
  0.5398364405114000  0.6048113259199491  0.9423169116644845
  0.3657360865972890  0.4678635034828749  0.7586238488742427
  0.5485990188568660  0.6078437904347225  0.7751728687482996
  0.5414715312016144  0.6379317990049272  0.4540579303214050
  0.4253407595571873  0.6311330044858923  0.3671533654331990
  0.6174205615681889  0.5362539837614286  0.4772807851075550
  0.3823619849825014  0.5223164722089658  0.3051750453415826
  0.5751339279582081  0.4272760265712853  0.4160323965855101
  0.4583408653498785  0.4202929879009626  0.3289231164010051
  0.5073135552793901  0.5748152990847697  0.6350186328894513
```

MOR-Al-T4-TS6

```
Al   Si   O    C    H
   1.00000000000000
    18.1842269897461009   -0.0090872207656503   -0.0001607598969713
    -0.0105508984997869   20.0691013336181996    0.0015994716668501
    -0.0001797722798074    0.0011982563883066   14.8440780639647993
   Al   Si   O    C    H
     1    95   192    16    19
Direct
  0.5858404130759138  0.7127857192301594  0.6363505638469032
  0.3142830746945837  0.0700777491990833  0.0363326554310027
  0.3036924219064210  0.3150222354365523  0.0388920273122939
  0.8102115588836352  0.5708768861102737  0.0280345936852141
  0.8073494474611673  0.7942085373680509  0.0310960673930176
  0.6894435344495087  0.9220374461481790  0.2796104944029522
  0.7039341721453507  0.6765617438928224  0.2790032960377778
  0.1927271453779380  0.4206151518072014  0.2869575796228068
  0.1933094977072078  0.1966736274249906  0.2860036388877383
  0.6920214959822415  0.0726316636654439  0.2316198258263789
  0.6935742721475987  0.2973228983923263  0.2355515954206273
  0.1897544439508974  0.5711661670360264  0.2380502887895106
  0.1984934761559948  0.8168849847192453  0.2366035441002805
  0.3087939148532585  0.9211290700759923  0.4871136413236849
  0.3065569962653072  0.6999898818777526  0.4867110651299735
  0.8146365591839776  0.4238950634417790  0.4844442751161274
  0.8033016988627967  0.1786766285903694  0.4807308695215179
  0.7050278176721466  0.9161037166568869  0.4879591693922476
  0.6931579009821378  0.6775890913053386  0.4879650942390290
  0.1869373441763296  0.4330384083282823  0.4922287238487025
  0.2091462658462668  0.1865956101141370  0.4919010728314443
  0.2962812312558746  0.0804638486438052  0.2440034544819042
  0.3094438511765105  0.3135819354067618  0.2464403867194044
  0.8181864893880831  0.5622072550127427  0.2305540251337561
  0.7956416031750996  0.8086111643358895  0.2355943246934649
  0.3171783064849690  0.9312367304229036  0.2821378126101378
  0.2930934178123469  0.6870315377591276  0.2810990958624251
  0.7981218590738668  0.4136914382152375  0.2756936828783029
  0.8086819451720926  0.1800098762240092  0.2740526555771285
  0.6877402903871074  0.0604650541274735  0.0265580584169147
  0.7094709785473556  0.3063436506383874  0.0289539467173592
  0.2079936431280189  0.5804721773126867  0.0303507659736277
  0.1928941384696087  0.8134503290274887  0.0288624425293834
  0.0850833306093570  0.3734490261637416  0.1339230432824959
```

0.0884977194434843	0.2185732304469235	0.1290288032697855
0.5861342529646171	0.8709417828582746	0.1372964787895924
0.5820783010593011	0.7107886882357243	0.1436692250516868
0.9162940036573380	0.6211644193117553	0.3795768265326113
0.9114037625474548	0.7762096506461276	0.3780586933549340
0.4163397071374429	0.1225595305369930	0.3920560534164196
0.4198011365557294	0.2813943274012521	0.3968706756140373
0.9167872308978886	0.3705861963252415	0.1267000737547311
0.9196611275658341	0.2123443117322738	0.1230296687764493
0.4181777596614147	0.8755942866069562	0.1376915570447650
0.4139008903050962	0.7210459337273758	0.1415329969016334
0.0853846023155750	0.6239860697271008	0.3801312518358253
0.0808506099646445	0.7828745771171584	0.3777351658434690
0.5846865621026571	0.1197440355289167	0.3848743073096421
0.5883581216660226	0.2751488073627054	0.3908929273767722
0.3153124266654564	0.0712922052898309	0.5357359369905966
0.3036788561073225	0.3166514329378187	0.5388566162932734
0.8095487988531525	0.5731176880090929	0.5342746709501939
0.8038766906991955	0.7958717580154139	0.5337669170765784
0.6890379539389780	0.9234187769509703	0.7807311643634213
0.7057130003117437	0.6786677872313984	0.7873101361996736
0.1932373108388001	0.4208176131908322	0.7863960269625125
0.1932297921974560	0.1966725724312218	0.7859302696304311
0.6948310675432294	0.0710051038595255	0.7316172689497753
0.6939554363031089	0.2968722174013841	0.7337254923511956
0.1877689611940612	0.5701561145595867	0.7382501713966586
0.1993002067638273	0.8151508322982581	0.7338798443412525
0.3078938316438596	0.9215089099633279	0.9869249889620756
0.3088478769835746	0.6981706970448576	0.9869747025311362
0.8153757041822186	0.4208017609501239	0.9819139959593520
0.8038430721388158	0.1770003914077154	0.9811583488251333
0.7052614078903694	0.9123824545479240	0.9881421943112340
0.6913573015682687	0.6757674849646264	0.9949263092380578
0.1884663796123603	0.4318689000180957	0.9917137434061987
0.2089721004441937	0.1858509446616142	0.9921970931531360
0.2952886145591265	0.0790623763287426	0.7429141786050484
0.3099896320950766	0.3132092735237129	0.7466786369873958
0.8219456134784314	0.5615854730900705	0.7401978424595804
0.7960501296114162	0.8070846514274542	0.7407792325598949
0.3152850428732316	0.9313142691867621	0.7811258487862804
0.2919575996651944	0.6861588775231464	0.7801207858971317
0.7979959479596103	0.4119916583988553	0.7758425504973705
0.8097797914428825	0.1808746720532753	0.7736528530273010
0.6881195592475859	0.0618349570308403	0.5256595990466523
0.7096475317011360	0.3079106983012591	0.5276725838973954
0.2031599867926670	0.5822591311390918	0.5300422480420742
0.1901820896739919	0.8167515069353630	0.5259445220869933
0.0849520302050867	0.3736593985423278	0.6347104479707948
0.0886804932305169	0.2188755559841894	0.6294024162875659
0.5866544322503010	0.8758283082084983	0.6348133335888829
0.9205600484022685	0.6195897820406596	0.8790376198410933
0.9155048853287346	0.7730107829998167	0.8721474817240612
0.4158259416166684	0.1190286985952091	0.8919500958394643
0.4192389435415452	0.2780908656870464	0.8969672425089754
0.9167488370732929	0.3712450124494585	0.6264382146033698
0.9198743275629618	0.2129181337422970	0.6221604366117817
0.4176048207167363	0.8739911416742462	0.6395154843136853
0.4144294679966489	0.7194464418901093	0.6447449532463869
0.0878933645788050	0.6214877359818199	0.8820504894068186
0.0848943982927582	0.7800234374386171	0.8771315349814123
0.5847435203474308	0.1158631231278546	0.8827462997533191

0.5891709488495925	0.2705933189129650	0.8904389295303098
0.1322285207316702	0.3815456089876358	0.2264151191807178
0.1170313199639672	0.2108036318230981	0.2325625756702852
0.2532632010380220	0.1165392993620741	0.4843923959151795
0.6110143529293872	0.9097587985182546	0.2290070154171004
0.6262672174922969	0.6744761171963751	0.2247560554039671
0.7348620524415352	0.6163563425747992	0.5409892020186792
0.8694466022990186	0.6103988899805523	0.4709879707378646
0.8818519549034661	0.7852473392703316	0.4805704164535810
0.7517689302648276	0.8789434235211143	0.2264169732350554
0.3931429905408531	0.0821275969730507	0.4831831666705743
0.3765734325654348	0.3218622534819428	0.4755523241631603
0.2685490630569620	0.3775763669067476	0.2917720516332845
0.8917984938962236	0.4113815854635203	0.0368945725364899
0.8760988502095051	0.1714065910586256	0.0452447932375777
0.7677902503073455	0.1164442754750886	0.2274720360662937
0.3687444153313759	0.8827126326152824	0.0472187116839595
0.3866480690142490	0.7124136566095577	0.0376303352351789
0.2535424653587772	0.6144078584653289	0.2884756456581142
0.1121703582500357	0.5835729613177287	0.2904163804004783
0.1266884987568865	0.8228091239874010	0.3001785957855460
0.2328900589618376	0.8774721105573540	0.9823671993331833
0.6319483459415237	0.1117083194319300	0.2926695852041081
0.6159736336491252	0.2850144223553099	0.2874375423768975
0.7506631147547906	0.3783580734517690	0.0333417476311805
0.8877044236814270	0.5732018963264116	0.2982413635210546
0.8619173493115212	0.8187042282507314	0.3075052343153930
0.7787311964571720	0.8685696549734352	0.0054155358687111
0.3624851293095153	0.1057436931896180	0.3088064304300338
0.3865422801761267	0.3009887269524144	0.2987397202343023
0.2467952411156846	0.3723474298209314	0.0084404436394337
0.1167214796527719	0.4228556716908546	0.0562184626557627
0.1401195812902825	0.1764524317947087	0.0606803254461698
0.2212887833626752	0.1215597161089238	0.2641783983598901
0.6384188636055881	0.8903154812852345	0.0534030290618805
0.6131823624478241	0.6854710794371925	0.0466391080652631
0.7593605133866329	0.6217368935176062	0.2375288095525180
0.1353687995256710	0.6042187771785795	0.4664934758328832
0.1118707899627437	0.8044415997229067	0.4764432246001650
0.2595162168469955	0.8717719201252385	0.2635679638438064
0.6145993699011164	0.0709671533719926	0.4637591924091593
0.6404458036984476	0.3169949855666495	0.4595785405388016
0.7219893021009742	0.3727020443280007	0.2569350736344878
0.8636080631692697	0.3869114788851379	0.2112871363226443
0.8851879051075232	0.1946010222509768	0.2212358781473850
0.7424443710833833	0.1246314980647498	0.5101048610531269
0.3889196769515880	0.9241980232621647	0.2173444471784120
0.3599207130121768	0.6801540451649408	0.2091646812563799
0.2771519645379282	0.6248868382405880	0.5084377364005789
0.2571258824054299	0.2483409480000351	0.2533303140965761
0.7413298804372630	0.7418025309232311	0.0113908995392667
0.7664744876725963	0.2500687345704829	0.9932857753128039
0.2344812070637105	0.7424906861760371	0.2469074290496386
0.7398349910045986	0.7505271858893450	0.2696190586155467
0.2645698333879077	0.2425970493670678	0.0307114627179449
0.2466731045794006	0.7488551845219128	0.0247572400418618
0.7554687522168934	0.2449929784987889	0.2699228177077159
-0.0001430160926149	0.3923484801272920	0.1536961448086821
0.0053172940653768	0.1900628554883695	0.1222101807900805
0.5027652307874684	0.8940316068218067	0.1133909214707965
0.4958303775492824	0.6902448587780345	0.1504864749791415

0.0013313213416978	0.6028302956547019	0.4011227407298968
0.9952771592664040	0.8033926523134431	0.3722217557651298
0.4992715731482509	0.1017232634305581	0.3636774891156356
0.5053407030634663	0.3042743236410466	0.4001279479399588
0.0918842246924931	0.2967223762407589	0.0992344009780081
0.5903434748939300	0.7911574460370744	0.1537711910527530
0.9081961960069270	0.6982393162907089	0.3470669234168998
0.4116447072229731	0.2019931964587030	0.4142945670583638
0.9123703429167804	0.2916081973727023	0.1039349264702595
0.4122401575005994	0.7988204619372952	0.1723905046957425
0.0921854616953381	0.7034807619188850	0.3613868363498796
0.5912002055321275	0.1968453541223160	0.4188405632202298
0.1775692888379561	0.2065231413951184	0.3929440690153623
0.1598030983137861	0.4289219475030500	0.3879879782264064
0.6885668745403669	0.6569547117868743	0.3823285709194716
0.6838801905474222	0.8990081429835455	0.3835486062792466
0.8300257943557781	0.7899614059295964	0.1370904617496634
0.8473153046020352	0.5591278406000769	0.1267583977927949
0.3291584964460624	0.3281838688639162	0.1419742553091449
0.3228458277970899	0.0933636554195318	0.1405331784149563
0.8289564180345210	0.1651775241233452	0.3780601298734341
0.8236957018287440	0.4021334282241590	0.3797010987981860
0.3259702856983857	0.7065950561771630	0.3797421766968464
0.3438262888460963	0.9292095017863562	0.3867512893014240
0.1731118640843075	0.8284937776298021	0.1333964851040275
0.1820157364008469	0.5929421778314957	0.1336190234945868
0.6786285853175794	0.2876903671760442	0.1283883075110431
0.6592497681533273	0.0646711524913737	0.1304691729931661
0.2120562770828572	0.4926822104513789	0.2433275028091703
0.7112673732585230	0.0005753479907536	0.2748963737363145
0.7918527834705426	0.4992298611432710	0.9814865214482396
0.2883870371549133	0.9928771905834176	0.0330886884042171
0.7791742794244757	0.4914426396106447	0.2561346308759406
0.2775639935590146	0.0024203205119274	0.2615478958714862
0.2283407189678737	0.5023226479589028	0.0138990719862276
0.7278163540599539	0.9896515000768249	0.0078769223028833
0.1323704681284158	0.3811136500364903	0.7269596985022011
0.1169711982093506	0.2111287340381729	0.7329548293522293
0.2523736496848905	0.1153644545774107	0.9848817967890823
0.6124130839984135	0.9169987695996654	0.7255076057002428
0.6416303949865518	0.6740855638948128	0.7155692391239851
0.7330785095105725	0.6109653814282323	0.0371465897231991
0.8670938142176440	0.6130422817561536	0.9662237494580982
0.8803232292901072	0.7773440692252146	0.9728011011422748
0.7521625557874007	0.8773530384030790	0.7342091456724286
0.3921900000188995	0.0795117465332842	0.9832850337362882
0.3768471498774282	0.3180655277384045	0.9762601586986513
0.2692249349106028	0.3775668198404069	0.7905602689292295
0.8924755963633368	0.4132186132533914	0.5371812916440574
0.8755130612942995	0.1728427049799278	0.5443965947641169
0.7681353764165441	0.1177455263093511	0.7279394388406147
0.3664182331674983	0.8810310170309308	0.5498945802195021
0.3816243413200878	0.7138279124858501	0.5414532602954408
0.2503406447465616	0.6144701853012550	0.7891700686251032
0.1103599028250112	0.5792019843325813	0.7920674282325416
0.1277266974224228	0.8205386012547816	0.7991273356133269
0.2312402917525803	0.8806466148369961	0.4807434553765787
0.6310886370970176	0.1081416722335498	0.7902355861458642
0.6171038033161883	0.2830681683295523	0.7871408029142510
0.7517338724333059	0.3791228520690132	0.5346158623705027
0.8968016042777853	0.5672810383766120	0.8003674482566903

0.8703988965701390	0.8182052417233631	0.8003892128629518
0.7754138661352907	0.8717429577771493	0.5181191055381333
0.3619260586231975	0.1011943987358908	0.8089091401318420
0.3869169096723767	0.3001865766519714	0.7992707995511691
0.2472273900722161	0.3752964082137060	0.5118977385941164
0.1158890622846382	0.4235416895951249	0.5570472123897096
0.1414438995969255	0.1768581243303071	0.5616305051082737
0.2213562206193795	0.1216341506137249	0.7641019246837760
0.6328421332398858	0.9043587271508422	0.5484958916611010
0.6127010478668588	0.6859749637248042	0.5285465132220953
0.7673690554301195	0.6212486585104365	0.7674179560458664
0.1411179921001417	0.6032533962886547	0.9656826178249558
0.1168667158567882	0.7986309311015187	0.9763210297468439
0.2590619366065808	0.8703498309381450	0.7647935644745206
0.6157463604465383	0.0669942150108045	0.9611963539668906
0.6403383278929591	0.3126710500201287	0.9601405045998992
0.7215751231881525	0.3725646893589352	0.7543312543840290
0.8633519305813138	0.3863367694766862	0.7109071509224570
0.8860713018758012	0.1953026898159593	0.7207692123380596
0.7446896068291675	0.1202393966330590	0.0069802838051579
0.3878025573668071	0.9239916763463264	0.7183209087416771
0.3597454612580231	0.6770673845699510	0.7111655675475143
0.2827163630857585	0.6223305447508628	0.0090551465375792
0.2572215757039028	0.2483319570171588	0.7521580653415982
0.7433506890344487	0.7446617950280570	0.4942948849074996
0.7668890197493594	0.2527075859337820	0.4893050906130668
0.2346453581179015	0.7411910796626034	0.7412711566579552
0.7468048382915949	0.7509699608432537	0.7914362300097902
0.2646478324540643	0.2445212032092082	0.5281225665520680
0.2413417129420004	0.7511359711578723	0.5138260839351356
0.7567721182860827	0.2462126893693567	0.7688915146794382
-0.0001729961374666	0.3924172392590954	0.6548722815480312
0.0056131399212549	0.1904564265033589	0.6210930571727573
0.5008043489657737	0.8952873154181117	0.6151603757601330
0.4944935594430729	0.6886070723556563	0.6486884126228414
0.0041882128222422	0.6024935495193174	0.9104019942049212
0.9988600686791870	0.8010465436847984	0.8756743174748792
0.4990027277706849	0.0984743046692107	0.8641747933205890
0.5059129025183877	0.2993477191759371	0.8991937030399515
0.0920751405781011	0.2970961629175669	0.5991952723559716
0.5988878473738186	0.7981295274847868	0.6475913151203230
0.9157063292266466	0.6952085494442334	0.8397581560105837
0.4103486009786299	0.1986931912658020	0.9122538865845652
0.9129528077583838	0.2924009257883703	0.6023528829699629
0.4102669964334512	0.7977509264150884	0.6757354875131696
0.0950173027906287	0.7003283522482927	0.8591020983937705
0.5924892425513471	0.1927253637898154	0.9174441710842289
0.1788028336454462	0.2069306783716064	0.8930417732680638
0.1611143909411327	0.4306802827427269	0.8873992093235545
0.6745905785048026	0.6641596885102581	0.8893727949979268
0.6766342853761851	0.9023925200095277	0.8853564720979763
0.8190525714642601	0.7826670541997781	0.6399567710135289
0.8445164351288672	0.5633324776866210	0.6342748591824081
0.3309354259518928	0.3276904582873843	0.6419805367304384
0.3227937437377857	0.0937705301037305	0.6402259860387106
0.8301181125731174	0.1666196174457643	0.8779163753996387
0.8235931587926142	0.3958308021653285	0.8788304490094999
0.3211131530505176	0.7084453371938213	0.8794447883320098
0.3408539170355754	0.9322188543914234	0.8863386847914732
0.1738015786035904	0.8297051630158924	0.6316494783309473
0.1782269515594830	0.5933378282139147	0.6340616720697372

```
  0.6788146024204330  0.2867806701754460  0.6266730051018672
  0.6631469532182387  0.0600466558499743  0.6305858946295076
  0.2125065306352882  0.4921921408605780  0.7400380608741859
  0.7172680961589114  0.0007702144289786  0.7781808365052284
  0.7891290926144140  0.5010747865064634  0.4900276692543460
  0.2907445977502242  0.9938582016985688  0.5304002837881148
  0.7815405943140635  0.4905110938762117  0.7617117760348528
  0.2742684607278911  0.0012904424679555  0.7571528061201596
  0.2253411299181183  0.5045860382188215  0.5120201772560479
  0.7305065561746263  0.9940544400145462  0.4969612302823554
  0.5797335976388877  0.4696007110795617  0.7783406382009328
  0.6025423730658019  0.5228611883170244  0.7222971875235812
  0.5325106508733449  0.4212973311932395  0.7413954895213312
  0.5737633687461275  0.5318851181671141  0.6379172194447388
  0.5109517322124816  0.4243860370316099  0.6529458181569667
  0.5266782740585864  0.4822860826056342  0.6019797562309285
  0.4778091755549764  0.4903324484830233  0.5273266447996849
  0.5515587612988425  0.5528929485363332  0.4053033531039505
  0.4924949652742294  0.5850368533152648  0.3614743177872098
  0.5836168700889284  0.4952792103823786  0.3666302911554468
  0.4658508580816513  0.5623842199767272  0.2787775121492291
  0.5588759073715792  0.4727939870156230  0.2815140621286865
  0.4998996201799720  0.5053089557965085  0.2397749079493538
  0.6081769332452921  0.4657581804797891  0.8732241634600522
  0.4188117558170050  0.5408971000412504  0.5289511577684131
  0.5482115093048144  0.4767780473892712  0.9456046473882667
  0.5719657977145837  0.4712916718455017  0.0137702454131424
  0.5244416476598803  0.5271098981331794  0.9401710958661003
  0.5036315753519668  0.4397404481296694  0.9390109027737307
  0.3803562372007444  0.5143397804024473  0.5781775147601637
  0.4331835010328480  0.5887605623285085  0.5605016400893401
  0.3880462225580544  0.5457983914586751  0.4655295164123565
  0.6527143114885019  0.5027988212574198  0.8834553117743125
  0.6327284548278675  0.4164600904223300  0.8846698145353378
  0.6426207288063696  0.5587607110265724  0.7488204966334677
  0.5131195353536899  0.3811838587602301  0.7833371001177308
  0.5898359029876351  0.5744120757312281  0.5951048031454255
  0.4754302504098644  0.3863033828598352  0.6248638116065010
  0.5811960219161001  0.5794873274068451  0.4573274383287361
  0.4675713177880718  0.6290987947080560  0.3929033178444596
  0.6292414089527855  0.4717876493018098  0.3994787306711503
  0.4290967996002479  0.5910481424327769  0.2380712696318427
  0.5908472691614723  0.4392726950681916  0.2420418484143773
  0.4845664365344412  0.4922231568460351  0.1714566334172170
  0.4658613336709078  0.4465046300921058  0.4852055250446323
```

MOR-Al-T4-I1^{+}

```
Al   Si     O     C     H
   1.00000000000000
    18.1842269897460938   -0.0090872207656503   -0.0001607598969713
    -0.0105508984997869   20.0691013336181641    0.0015994716668501
    -0.0001797722798074    0.0011982563883066   14.8440780639648438
   Al   Si   O    C    H
     1    95   192    16    19
Direct
  0.5853684507888192  0.7121746604364393  0.6367221518597627
  0.3143154442618270  0.0699696691378696  0.0363090328649611
  0.3035395384963315  0.3146531987889973  0.0388692019443824
  0.8102247592140764  0.5708241370622936  0.0278761516329123
```

0.8072674144341735	0.7939912950203327	0.0309111114018404
0.6891562191478277	0.9219904107029742	0.2792891737834551
0.7038042834314910	0.6761780294858382	0.2787550278624910
0.1927491542174321	0.4203974375853378	0.2870610664936560
0.1933432630956523	0.1965343321013200	0.2862852162988964
0.6923351528654850	0.0726779677579786	0.2313422796984620
0.6936214932591801	0.2970716871331635	0.2356673866356334
0.1896694110417544	0.5708409214218818	0.2385714286140393
0.1998612478356179	0.8155395058317356	0.2363624892297166
0.3085974421053030	0.9212002298730168	0.4875455460601736
0.3063589323361259	0.6999032007390229	0.4869382133377645
0.8151339557411952	0.4235675122387147	0.4846368359664602
0.8032541510670353	0.1787252263908433	0.4808034536741534
0.7048213093371732	0.9160142248787995	0.4876895328979279
0.6917421162624933	0.6766115973363425	0.4879727332732738
0.1869493874813538	0.4327349223478073	0.4923292391051566
0.2090244204115342	0.1864610552249297	0.4920637446603816
0.2967212100712135	0.0804306056103042	0.2438873496340206
0.3093359928418576	0.3133999915165599	0.2467149088514916
0.8188584032043267	0.5614379253173641	0.2304350949421765
0.7956855397255816	0.8081050137416665	0.2351217221895291
0.3172712323463787	0.9312528773760655	0.2826665030743086
0.2946649109214532	0.6860254332941426	0.2812563422984120
0.7990353713587595	0.4125250827091467	0.2758457110697471
0.8087812122481914	0.1799522788085600	0.2738656100604008
0.6873970759694432	0.0603425853903094	0.0264322187462683
0.7097336085036761	0.3060571577901855	0.0287842420861715
0.2076890293601734	0.5802619583238262	0.0304703372617004
0.1931502683889120	0.8132961797384014	0.0287127979510441
0.0852855501439310	0.3731335856082740	0.1339867481021927
0.0885612257038894	0.2182026074190296	0.1291730135854174
0.5861648054451728	0.8707734451878518	0.1372367439962780
0.5829673738041522	0.7111384704917044	0.1421007817666601
0.9162087185844024	0.6208421788337243	0.3795941032285802
0.9114780451644037	0.7760192309753353	0.3777326537277831
0.4164866270102059	0.1225100517033299	0.3924277075252249
0.4196984593561275	0.2812135457942049	0.3967541438755612
0.9170725864089236	0.3700345708103288	0.1262408737063361
0.9197645187000226	0.2118317922987516	0.1230277136682196
0.4177210010375612	0.8740531827367165	0.1378636088253627
0.4142204588305525	0.7196795633211439	0.1420564069147138
0.0851416739004610	0.6233075287616449	0.3809252131458138
0.0811675985668286	0.7825345174551515	0.3778324517724707
0.5847168065089088	0.1195376630017596	0.3848502419807565
0.5882453540602920	0.2749617413052001	0.3911629423820326
0.3151888927536035	0.0713321851344461	0.5357663683571871
0.3035412540998929	0.3166376675971591	0.5389559564656520
0.8092735023973063	0.5727788768556699	0.5341043401214863
0.8032334326485893	0.7950768225244540	0.5330055909182090
0.6891615997813070	0.9229634365198086	0.7806774325722133
0.7059793664265199	0.6782081968569970	0.7871894947162453
0.1936232712761523	0.4204912902019793	0.7864153119124532
0.1936037897408850	0.1967921932138445	0.7859669734587102
0.6947445337778051	0.0707661859034988	0.7316759867928366
0.6937842898277423	0.2969491753313505	0.7338354798776462
0.1877146185697854	0.5698740901409347	0.7388390862701746
0.1992561218317188	0.8151550050683193	0.7345464744345971
0.3079592312763408	0.9211204578738053	0.9871808432309940
0.3089409732994834	0.6976915439990606	0.9867364676409048
0.8147893615923733	0.4209516030217219	0.9823197118904483
0.8041877213767054	0.1767211694327356	0.9812608933439663

0.7051905879723718	0.9123438577018813	0.9882377512581845
0.6915637102666163	0.6756604226814618	0.9939606500978616
0.1883377332952672	0.4315117399288968	0.9917336093022598
0.2089864561740049	0.1853235988236933	0.9922985320822492
0.2954220940312502	0.0790882236113709	0.7430292136073308
0.3101258746462512	0.3131586269202625	0.7467367159740745
0.8221691101694052	0.5613278282847076	0.7399566815917672
0.7959745946179667	0.8065424288879054	0.7403734016563642
0.3153453089986284	0.9312548674644221	0.7815047736715761
0.2918863102507112	0.6858956209424528	0.7804874258693277
0.7980649782222319	0.4116802812375788	0.7760428361364133
0.8095859731282461	0.1807709566817913	0.7735990869376772
0.6883183555518647	0.0616716360111562	0.5255513693729618
0.7096082526030648	0.3077691261294797	0.5276416131127093
0.2028411859156943	0.5820426378021635	0.5306298779128015
0.1900198017908554	0.8168892513924677	0.5258186200564761
0.0851190387129859	0.3734301644659545	0.6348688470810228
0.0886842597262147	0.2187638953892642	0.6294083081863459
0.5866252454851706	0.8751913405271825	0.6345841929200227
0.9206391018093809	0.6193683804129986	0.8786867495169961
0.9155535524677140	0.7729576872464233	0.8718515391721701
0.4158793699455419	0.1190095375929888	0.8918879181682340
0.4196309242509862	0.2778983116881262	0.8969806697736352
0.9171368523050516	0.3708927463649223	0.6268521861167359
0.9199607072397283	0.2127666451385122	0.6224051463578401
0.4173480055462305	0.8736717731319252	0.6398915214650268
0.4140056628400794	0.7191473253435283	0.6452680326574298
0.0875293790115869	0.6213138743098382	0.8823135123437501
0.0844823171509815	0.7795547170953625	0.8775064846283888
0.5848560571459426	0.1158569381629100	0.8826701841551960
0.5892418333705017	0.2709223585121806	0.8901909302845834
0.1323809369331718	0.3812899055705602	0.2263005922227411
0.1170248358365423	0.2104597021218830	0.2327454696674510
0.2533302985469893	0.1165219826708858	0.4845823384867838
0.6105166969368654	0.9099724333565720	0.2287081549137729
0.6269200918056401	0.6731442493929451	0.2225096636123204
0.7341721243515097	0.6154604222531395	0.5400161407073518
0.8687998700921170	0.6106042480212281	0.4707261785052617
0.8811498840480139	0.7846003776957496	0.4801161505335368
0.7510390816052891	0.8780320320307181	0.2263784830774150
0.3932288015262329	0.0820747499943951	0.4833436305246125
0.3761081150263328	0.3215935392921843	0.4753005132303171
0.2686826815593760	0.3775624714284204	0.2916984535138112
0.8918892441661850	0.4113068635732940	0.0369473219225482
0.8762375389748955	0.1707633975962492	0.0453007384529005
0.7678173854604705	0.1164983256918431	0.2275964608232937
0.3682398488791523	0.8816067309229279	0.0475843777674864
0.3864618914266341	0.7112114771207817	0.0384981041730829
0.2537690777672257	0.6140306162511391	0.2887662956791002
0.1123598092083180	0.5833798204167570	0.2911156850636893
0.1277019005039135	0.8208854870635485	0.2999982066755142
0.2326463929873837	0.8775285871310562	0.9823058463097887
0.6319783107640231	0.1113878362835087	0.2927391649142257
0.6159814138256235	0.2851163850727293	0.2875260064343930
0.7508360082002541	0.3780389916065818	0.0336566457946610
0.8878489269506760	0.5727361554286612	0.2982902721558681
0.8616389054254586	0.8184712780692699	0.3074297198542926
0.7785464673583807	0.8684857489196774	0.0067133708350698
0.3627016523070940	0.1055244088827750	0.3088486765341301
0.3865484668518907	0.3008926856952543	0.2986478122947845
0.2464816996610177	0.3720307078839445	0.0085917916536470

0.1164871307207833	0.4225089915570829	0.0560488639121976
0.1402467589412511	0.1758047226298827	0.0609095895860141
0.2214855001786011	0.1214599346482272	0.2643496837661422
0.6378353011727037	0.8903797853313730	0.0530986462233227
0.6131510819013780	0.6857160193262414	0.0451658068389401
0.7590702476050801	0.6203513339625154	0.2387697947251436
0.1342840874617180	0.6036418384869625	0.4677314895346449
0.1115285724350067	0.8047467827800455	0.4764523858886778
0.2593605109039623	0.8717466771858277	0.2640250004523443
0.6148339049910212	0.0707000664029628	0.4636587422854685
0.6404004860672621	0.3171408363000195	0.4593622518656217
0.7223760367010303	0.3724372021628458	0.2576183595942858
0.8641107875743100	0.3856605676529720	0.2114157398036372
0.8850924794223806	0.1947723597028862	0.2212333520839245
0.7426854828000188	0.1243463940195399	0.5101094977642974
0.3890628016274956	0.9230232877852080	0.2177968990910546
0.3627868230626616	0.6768945163642845	0.2109776298942379
0.2759769745188519	0.6251684329035686	0.5081757911182933
0.2568866888225412	0.2482741485630746	0.2537859116981678
0.7414739128399009	0.7416004329946982	0.0103104872981373
0.7669731980894907	0.2498816285632250	0.9937596793303632
0.2375375052329988	0.7421341858398193	0.2455064299737297
0.7400991901660064	0.7497446892872074	0.2697343546338483
0.2645330118107845	0.2421075518690971	0.0308244931619096
0.2469735588026142	0.7488370818723612	0.0239414994641447
0.7554370484335085	0.2449337872792348	0.2701893699938130
0.0000459767907490	0.3918534828321069	0.1538504518225288
0.0054146146268731	0.1897275136959321	0.1224722993217463
0.5022269238437272	0.8919678691098619	0.1133736761984970
0.4968576793533542	0.6898566844686106	0.1499499043138207
0.0009483604346190	0.6019262725219767	0.4015513424707959
0.9953535760451830	0.8032415328148542	0.3716025952275709
0.4994146526731928	0.1016236874466320	0.3639502791249490
0.5052996126326734	0.3041348960818180	0.3996376193698978
0.0920402619317512	0.2964218742614707	0.0992796783317919
0.5919301614170565	0.7909223966890189	0.1543427265712490
0.9085752464670256	0.6978680767450716	0.3469690132305293
0.4117059069305273	0.2018580696072410	0.4143805524552525
0.9125996864097600	0.2911707978008613	0.1028223998553617
0.4114290722094077	0.7972922684187360	0.1732188166879401
0.0916390371620778	0.7027889552909158	0.3628464054599753
0.5913888116983611	0.1966682556743017	0.4190114096403758
0.1772610285193333	0.2062408200132332	0.3931890999207361
0.1596520695956432	0.4287700316302568	0.3880947777054425
0.6859028545719627	0.6564288962420327	0.3815687357892187
0.6832172442844170	0.8991860800490268	0.3834609298109913
0.8296720899503935	0.7891385280138919	0.1370835663838211
0.8476578170072150	0.5594571147984850	0.1266548619136305
0.3288107405963237	0.3278437288580796	0.1420650841841472
0.3230702464698677	0.0933818165643244	0.1404402579524053
0.8291291315821651	0.1650466238646087	0.3780136871416742
0.8246635827386137	0.4017783069722519	0.3797918319251497
0.3270649157621267	0.7053132745480452	0.3799427259992215
0.3438043824507879	0.9292268577160513	0.3871937581724575
0.1738111777622224	0.8278776499643026	0.1333557666716896
0.1818590283407843	0.5930740589468724	0.1340844221390657
0.6787962494705772	0.2876980423264826	0.1285744239998252
0.6590717771416001	0.0648870034895124	0.1302996109812398
0.2119625753795999	0.4925007995208682	0.2432393741310455
0.7115404458007176	0.0003594928007731	0.2740795154291646
0.7918748451690316	0.4989934798097120	0.9822739534067353

0.2889118750259112	0.9924434281774451	0.0336534401927462
0.7800118837338855	0.4903514739634459	0.2554680017989238
0.2781250004729833	0.0023209765290044	0.2611533268572998
0.2278054028314324	0.5021873168195282	0.0143621040423059
0.7277035465748569	0.9895222294810018	0.0083080038940603
0.1328077539011798	0.3808549789521087	0.7268053226118260
0.1168419953467633	0.2108875961747395	0.7331934932087886
0.2524151979718772	0.1148546839034795	0.9848509708481172
0.6124155199556851	0.9158560753898930	0.7257930877235671
0.6422985434047845	0.6740481014497749	0.7150267092137231
0.7329700892497689	0.6109280088393649	0.0375976518193702
0.8666783902611461	0.6129111257055810	0.9655395001474961
0.8801380703920360	0.7776989391328815	0.9723503529240719
0.7526173605334662	0.8771378294070563	0.7342774167489963
0.3923535474811595	0.0791507527748241	0.9833494806985543
0.3766318948830141	0.3177917727467819	0.9761930132078372
0.2693981287115411	0.3774025892772092	0.7910923215495961
0.8927482639164139	0.4131034317476019	0.5376621808506415
0.8755442020912049	0.1729480702620173	0.5443537332097839
0.7682018951399155	0.1172803425433207	0.7282796338757314
0.3660387993453270	0.8807423493298099	0.5501092102533927
0.3812250411632487	0.7137022164605337	0.5418829348141051
0.2497564132253431	0.6144660961052453	0.7901475828945926
0.1101144266649828	0.5787638716027864	0.7926549144167805
0.1276556820529656	0.8204808500985091	0.7993312276526802
0.2308519146435029	0.8810439083902413	0.4808801829385350
0.6309781431296059	0.1079787877241624	0.7898676256673018
0.6170711572662662	0.2826921866886446	0.7870745187988043
0.7516595396099358	0.3791322399565781	0.5340180674724703
0.8975557018041069	0.5670534984657684	0.7995882163815814
0.8709242054953227	0.8176844837511379	0.7990680101317665
0.7747375709608024	0.8708690684988433	0.5172587380867307
0.3621568558394740	0.1009179769677545	0.8088755252269128
0.3868954964057138	0.2996634682125564	0.7992685800521955
0.2470599789382906	0.3753318204488711	0.5120334879691483
0.1155876585432182	0.4233748206766542	0.5570671432213925
0.1412943424086475	0.1765965152382299	0.5619471344886278
0.2213801680082484	0.1216314601726509	0.7642762396609247
0.6327825170546031	0.9047576845822366	0.5488821659851815
0.6110722019154666	0.6843636387594365	0.5284947080285871
0.7685526333272223	0.6215171271036622	0.7673438415431886
0.1404678650710418	0.6031556632366599	0.9663939889162816
0.1168915091732761	0.7983424404686172	0.9764112993252471
0.2590515191471719	0.8703512719679427	0.7645874959208091
0.6159175590507899	0.0667437435347747	0.9608663496908224
0.6406055169137587	0.3120091441584488	0.9602957841581571
0.7218535222393704	0.3723256510005099	0.7542626567253417
0.8634875192484065	0.3860060336573632	0.7109256290307294
0.8859591199804921	0.1950203544164167	0.7206906053704852
0.7448491240932059	0.1200328994344461	0.0068874579992791
0.3877974028922598	0.9239040902702794	0.7185128598288272
0.3592578055482996	0.6765635291611894	0.7112446333181290
0.2821705597548672	0.6220641612805224	0.0090399183629904
0.2569553570132520	0.2483722088446042	0.7523891078966340
0.7422432866752368	0.7437374985852822	0.4940211647439565
0.7666247776681866	0.2525334788328146	0.4888777058617574
0.2345823753004181	0.7410844302027222	0.7418653329783670
0.7469763277898612	0.7508211515831452	0.7919177200064949
0.2641523402319885	0.2445379918560981	0.5281893801367546
0.2413345295625501	0.7515362338442508	0.5124388286717049
0.7566305613106620	0.2458084621098913	0.7689251954619962

0.0000766591941769	0.3921374188047015	0.6553717148792573
0.0055897075556464	0.1902716495123213	0.6212624031018906
0.5006436210141406	0.8946280411870190	0.6153551288626254
0.4941681911166039	0.6887614706246319	0.6497161403827282
0.0039760604614065	0.6023042163179242	0.9107493834788508
-0.0010151924678294	0.8010177719139032	0.8759051658615324
0.4990537120058396	0.0984566527931906	0.8641948617100961
0.5058158270792200	0.2986782164093319	0.8998112488742183
0.0920622600565211	0.2969445086563319	0.5992699514611298
0.5992260489017532	0.7974882488810955	0.6460274138099412
0.9158075644961475	0.6950548325966811	0.8397941673590169
0.4099926229784864	0.1984575590221777	0.9125350915136469
0.9130475174160352	0.2921582703760167	0.6023102670385584
0.4096204434285994	0.7975820614431437	0.6761930156411590
0.0948066318436259	0.7001415705189152	0.8591718806838110
0.5929069601561233	0.1925852928249675	0.9173699015773957
0.1787207498438866	0.2064458041746893	0.8932054038408646
0.1610303450675706	0.4303051727252487	0.8874308320399306
0.6747785989234268	0.6633664113566953	0.8887712804430553
0.6772089346235625	0.9020210234242423	0.8854134266990363
0.8180057088067627	0.7819100787296104	0.6394484305679177
0.8445516439349512	0.5631046260036581	0.6337407566564180
0.3306421130290055	0.3274546499935451	0.6420788194530344
0.3227372394666940	0.0937298188663172	0.6403482223815848
0.8303245350028059	0.1665015582259587	0.8779399312316762
0.8237363511759199	0.3957963842312993	0.8791175165412183
0.3217623651194724	0.7080668741950988	0.8795771613677573
0.3409461107800507	0.9318716324623225	0.8866673615180727
0.1738003357068324	0.8292483486483054	0.6316766229225017
0.1781522045138599	0.5931190318704592	0.6347882668306264
0.6791670142837649	0.2865929844394161	0.6265798046585965
0.6632483081453797	0.0598205007879421	0.6304715142374751
0.2125541906174167	0.4919879906907466	0.7405400063731090
0.7168371379935602	0.0004106911275922	0.7779947455552241
0.7892467139780369	0.5007576687513183	0.4890916363038070
0.2908337838290378	0.9938240386270535	0.5308165748766674
0.7822966116020689	0.4903542248082923	0.7616782471480376
0.2742898472475530	0.0012365817241886	0.7577164813172849
0.2248138887708338	0.5046258694897359	0.5117984126120126
0.7308318097986254	0.9937720487610564	0.4968648175715246
0.5807794421057692	0.4697849978875115	0.7828265584095242
0.6057159521250406	0.5206251958261253	0.7265125191386345
0.5288909881576387	0.4247510697714489	0.7477993829995294
0.5780092086258311	0.5290085624812102	0.6397439220867156
0.5021649910923369	0.4317584367097771	0.6604209654062223
0.5245295763455763	0.4855755629851747	0.6061426784133420
0.4815472056305029	0.4979573439408321	0.5209177756647914
0.5255642852827191	0.5435998354007776	0.4497385965898918
0.4788466646522392	0.5732841483137506	0.3804311035144474
0.5905809325608455	0.5082411863719938	0.4174080420631675
0.4941819792261158	0.5665449842512184	0.2901976177076258
0.6052301089825523	0.5017306409245215	0.3268840561508966
0.5570378304577397	0.5306331903599720	0.2637527739595362
0.6090080512951890	0.4648889771172781	0.8779909004103347
0.4075954780528981	0.5300735444389090	0.5443438367670392
0.5489354070174065	0.4764800010201211	0.9489927826973623
0.5719648678083212	0.4712802838520013	1.0172658921748332
0.5253660761098651	0.5266943485712313	0.9423098878220338
0.5041829768940861	0.4399610986407982	0.9418761674228359
0.3808325519790436	0.5000246671412589	0.5967846977233684
0.4157948714942781	0.5804884643681645	0.5713631767446644

```
  0.3705169769578071  0.5322570678801815  0.4861890432167966
  0.6534097063104231  0.5015478118189861  0.8872106765872285
  0.6334411712206994  0.4155416973595180  0.8889672215156147
  0.6470086459144998  0.5550916191237831  0.7512918601988986
  0.5086029941681187  0.3839869835300411  0.7893040750849584
  0.5990234452485719  0.5706472883719267  0.6000833947864233
  0.4610872449264943  0.3966809924722559  0.6350220776915192
  0.5439187919821398  0.5866898678918857  0.4928729945606275
  0.4316299377146891  0.6025511673760356  0.4022907867615101
  0.6263458933573456  0.4855882440311257  0.4679480830471089
  0.4590242259322505  0.5895974947750058  0.2397808581751732
  0.6531283949393977  0.4744468749815965  0.3032739913939347
  0.5689375905477867  0.5252051478631929  0.1919770718468018
  0.4711664204940593  0.4501595780657565  0.4865702765789129
```

MOR-Al-T4-TS7

```
   Al      Si      O      C      H
   1.00000000000000
    18.1842269897460938   -0.0090872207656503   -0.0001607598969713
    -0.0105508984997869   20.0691013336181641    0.0015994716668501
    -0.0001797722798074    0.0011982563883066   14.8440780639648438
   Al   Si   O    C    H
     1    95   192    16    19
Direct
  0.5844276776940172  0.7131260184045549  0.6363633873640547
  0.3098382502782723  0.0709037586717082  0.0260831808288491
  0.2965249659427913  0.3155221531645898  0.0294707910097503
  0.8111666960828118  0.5733973180295275  0.0267459972765136
  0.8080728929486245  0.7993582605749439  0.0278226351794221
  0.6878680233064348  0.9255165719148599  0.2774949093960589
  0.7041712792319416  0.6812274122509315  0.2772423445206563
  0.1903518523965774  0.4201533907442390  0.2760677030074332
  0.1882411571364885  0.1968753674161470  0.2750799776106100
  0.6878368622271427  0.0754945924581700  0.2292494618727478
  0.6901069191400306  0.3013346574905781  0.2305223443835489
  0.1868886051538853  0.5712684710716247  0.2299283911309846
  0.1993260127253103  0.8171541795592211  0.2268152842480427
  0.3071337270479756  0.9208611853660310  0.4784210897464726
  0.3060636069388182  0.6968520553427595  0.4802320164750601
  0.8087635576758259  0.4282366034247272  0.4816877293851263
  0.7956927776501344  0.1816109428209683  0.4782342018316144
  0.7041312723022303  0.9175142590493046  0.4856678243279123
  0.6949690828049191  0.6835359756751703  0.4863018991353002
  0.1782343398358420  0.4317578591976458  0.4810898540988901
  0.2015918322405901  0.1856827700673092  0.4812185491496624
  0.2932310337290177  0.0823945284599505  0.2332032564750353
  0.3059749408573040  0.3131204082939857  0.2369074426413861
  0.8165509522635441  0.5653746059805655  0.2295908133104241
  0.7949068528348954  0.8128634934186824  0.2327355174064988
  0.3165702249427446  0.9330771333176757  0.2728034278465436
  0.2924134411525722  0.6870007702523897  0.2734732867007676
  0.7928301591335422  0.4184414038356854  0.2732705413916817
  0.8044854651600936  0.1833716212949159  0.2699516277004146
  0.6809175665721529  0.0638780933294165  0.0241586105806688
  0.7031648541435863  0.3116923092030963  0.0237167735031715
  0.2054747888628905  0.5815968300141723  0.0221065214114103
  0.1928715202463465  0.8122352033165681  0.0199525059719887
  0.0803699229933039  0.3745850091290585  0.1229422905882159
  0.0826558177578734  0.2201034423262342  0.1183298405542239
```

0.5861048761720411	0.8725462955729818	0.1346313060246582
0.5827571719541186	0.7132704225322826	0.1420004553711041
0.9160855367672212	0.6237977641696369	0.3778906227324921
0.9126890817631903	0.7797136070788142	0.3739483116840576
0.4104535611075784	0.1240777172812782	0.3842579898763848
0.4143444156587918	0.2819789855721189	0.3879269973728936
0.9121207924696660	0.3734882456986758	0.1254351497589873
0.9136320679406778	0.2143545597103472	0.1206110451982778
0.4182589105235283	0.8755798981337956	0.1307915798082655
0.4143894066480888	0.7203115945637785	0.1367576158419310
0.0847772313145024	0.6266479756894882	0.3711867848347236
0.0816929489782727	0.7843028016057628	0.3674953825148898
0.5785456258990520	0.1208951824983334	0.3819189867421218
0.5831276230819691	0.2761103534478220	0.3862589653822320
0.3075602912843610	0.0703610933395531	0.5256889220547559
0.2959620241893127	0.3159683873987283	0.5278114613730841
0.8094618826942698	0.5771853934137592	0.5333950362225920
0.8071622804851725	0.8011184268528028	0.5283094669305143
0.6873393138456257	0.9258884090443424	0.7776971577190153
0.7015199038277729	0.6779896862681681	0.7833336111485787
0.1905109638069246	0.4212562769934288	0.7751410516178223
0.1885103081389872	0.1968100149871420	0.7763947831274969
0.6912886915461219	0.0738902504058191	0.7294069274770940
0.6883419798942739	0.3003228496282130	0.7310325638625680
0.1865531935007172	0.5710540065455534	0.7289438649997768
0.1976055078368436	0.8172328818283419	0.7278073270259504
0.3075842681534499	0.9212344246862632	0.9795858799815554
0.3096041899507661	0.6963563199626356	0.9814469443093201
0.8122165575881715	0.4239789612877495	0.9786856902328270
0.7961752341214796	0.1812289120657177	0.9776323832625826
0.7043380144663625	0.9152840435029583	0.9848243585909709
0.6933113272494262	0.6805278041198906	0.9917590980055003
0.1812607020174434	0.4324784675781170	0.9815116851893624
0.2029809719940050	0.1855787118846725	0.9826181311741221
0.2921081562030230	0.0811777026353225	0.7340453098400198
0.3058540173501366	0.3124291571138987	0.7355307919597313
0.8196446881308116	0.5630215981303385	0.7385136237606894
0.7926591604913108	0.8084541532888212	0.7357261697674171
0.3147083707097820	0.9328419779818002	0.7743575415687108
0.2906380100025928	0.6871853989677591	0.7743527003403785
0.7937667365536624	0.4134446524177208	0.7730884450670451
0.8046386398319965	0.1838764623345226	0.7698187514273697
0.6808371322215545	0.0644047842421792	0.5238435305335558
0.7037002177927077	0.3117697744056611	0.5243332224771948
0.2018117218766330	0.5825598649246967	0.5202444575319101
0.1904907093030551	0.8128163586995706	0.5190764487639888
0.0784774784468231	0.3745829242942534	0.6252303131439364
0.0822106905828294	0.2195874594970512	0.6203575048679273
0.5860345920375818	0.8749333041238806	0.6350001736322521
0.9202741641962892	0.6206142233541815	0.8769944594412874
0.9137523780682777	0.7747901511792475	0.8696005249601947
0.4115386634278951	0.1232549400048368	0.8821887564712476
0.4138502005690324	0.2814250458652662	0.8876601176577811
0.9109915818872191	0.3719247217241237	0.6222183903325692
0.9137751634327155	0.2144235825269593	0.6181575418544235
0.4172604640871012	0.8763758669216982	0.6316144939758740
0.4128556409829148	0.7214074740374107	0.6365226318255046
0.0871349268204750	0.6242188462918317	0.8724107915871421
0.0825389118968298	0.7816152033321765	0.8678624398643950
0.5805015656025039	0.1208840046081433	0.8800036764503714
0.5828834496591440	0.2763661997562458	0.8864283417303436

0.1325631999117956	0.3816240284880715	0.2111770261021431
0.1125325611588822	0.2126565597671247	0.2212866322548680
0.2447197693119936	0.1150310406058984	0.4756282090331490
0.6099703745177502	0.9131492276966007	0.2252180923726769
0.6259200307773343	0.6760983112742911	0.2237837305169836
0.7371804495214230	0.6234322846725563	0.5407296586826895
0.8713613826282057	0.6125354647726049	0.4707562691966249
0.8860943428305453	0.7909294223398554	0.4774866538971559
0.7515396382068283	0.8835027682300434	0.2250250939527770
0.3850201917764037	0.0815585032951492	0.4723592985932744
0.3675162197302317	0.3234851954632462	0.4626907578177871
0.2666080286414741	0.3777969771118881	0.2824618507586749
0.8887262991835145	0.4125200885826193	0.0335247996797415
0.8674852994716818	0.1760111051775280	0.0427535988888661
0.7619876000001478	0.1208344477051484	0.2237316194126494
0.3687057976194790	0.8835634544604787	0.0406214430221756
0.3871290324873539	0.7106478112538914	0.0327101651889611
0.2507121370007898	0.6153485277179904	0.2787535496274659
0.1098896172572649	0.5830303608256976	0.2836248789149512
0.1285368996565074	0.8250946651899670	0.2923634681293139
0.2332144348179243	0.8763580540141315	0.9743656311269627
0.6272428175839830	0.1133491213437062	0.2910432630571494
0.6138368489467823	0.2860535497259741	0.2840927296885938
0.7464032521493360	0.3826436932453552	0.0288742258766650
0.8857387470423900	0.5756085510236738	0.2976521659190268
0.8611762107246365	0.8214975260364379	0.3048145894281748
0.7799840312355668	0.8740751517927012	0.0023032007147700
0.3578022408991243	0.1100522651454517	0.2985315799204557
0.3834741524544161	0.2991912464664155	0.2878332373288548
0.2376353690216231	0.3725576830235141	0.0036982696129261
0.1075110106588134	0.4245972172358990	0.0431167528700011
0.1334415823318418	0.1767999488744837	0.0501429060951947
0.2165497311401850	0.1220121910386878	0.2515356784098526
0.6394871841888481	0.8897457168173195	0.0510633128561201
0.6159581312395872	0.6899053537353148	0.0455270315769739
0.7605028757740527	0.6274404813548724	0.2344406260560631
0.1370773467217181	0.6119999421929778	0.4572005364636806
0.1128618669145156	0.8011602018379627	0.4676895062585912
0.2597510350684805	0.8734285347853229	0.2508008953785387
0.6069423312947105	0.0722558751999818	0.4621199154955456
0.6339822402380761	0.3183198235741222	0.4561551736614494
0.7173403163043642	0.3769270343632407	0.2534127482904182
0.8595182409854104	0.3940832443940826	0.2089087044228391
0.8828228443406174	0.1937740234920118	0.2196529274177830
0.7338569619864915	0.1277600262970593	0.5053531489663239
0.3896828469992348	0.9250892086950616	0.2102821029522729
0.3613704916517284	0.6790041437236450	0.2048642304419181
0.2796607970095948	0.6203667101744597	0.5011981286657785
0.2524531036767416	0.2487950289262417	0.2455283432117677
0.7425727983817483	0.7472147337962738	0.0043552626087437
0.7584916936336591	0.2544263567138080	0.9869848572991080
0.2358524598032677	0.7435995316642585	0.2375541691357872
0.7376230149804884	0.7556925881850691	0.2661442306711824
0.2585596295039786	0.2424961699642279	0.0208760450156665
0.2470927749797802	0.7479449859184893	0.0161763032070098
0.7546387816390264	0.2504208033884084	0.2612287128936470
0.9964019550201182	0.3930660817648996	0.1506238258229658
0.9991123210213044	0.1923565011059277	0.1135099714232880
0.5028859977616437	0.8940071903888407	0.1073871470085353
0.4969885181575619	0.6907797599321933	0.1460243384686998
0.0015209783439078	0.6054308026928429	0.3970091779551833

0.9963705377389065	0.8065794074962569	0.3630425611458146
0.4937682966002673	0.1029038552469319	0.3579453976846876
0.4999312755281350	0.3052940855318474	0.3931068487348860
0.0855677481449497	0.2980528694765186	0.0870074173528000
0.5900964102869046	0.7931769731678637	0.1551794447185004
0.9077477411453774	0.7010217179697436	0.3461636405547199
0.4066620025554427	0.2030107539256988	0.4093667945318655
0.9046379400291948	0.2941793653546204	0.1065465055929637
0.4117534311182053	0.7986600164261949	0.1652595279850440
0.0899837714322282	0.7051287135803133	0.3459706095157226
0.5849468519130435	0.1981589645706494	0.4156362105917236
0.1704493410708496	0.2046594806570624	0.3818892834271613
0.1540246447785416	0.4258166960227415	0.3759672451895272
0.6895751140996143	0.6611215745139482	0.3805432819963443
0.6811626644790300	0.9021015143400242	0.3813713558798708
0.8285182959479961	0.7942798205283904	0.1344089646144846
0.8460313542983711	0.5597320338771320	0.1262471382978347
0.3246558708982239	0.3269671198841623	0.1319114638526110
0.3201213740891892	0.0947608413931316	0.1298376000734809
0.8215821795331356	0.1693601271382960	0.3752130173174555
0.8181755876824202	0.4062725410751191	0.3770526785496504
0.3225860495488644	0.7060768426205989	0.3731817265417792
0.3411185184764875	0.9308091328131173	0.3779141898668384
0.1718001877174411	0.8272880781061019	0.1239402141975398
0.1775083966107061	0.5927301885983198	0.1251700248222133
0.6727385177260894	0.2931875889509075	0.1236733368806797
0.6539823545169821	0.0663926620744077	0.1286631325601224
0.2098671986666736	0.4931363588472381	0.2354397554075862
0.7090220001195116	0.0042201728232916	0.2734631973371928
0.7917061203733573	0.5026437556255735	0.9783914914832571
0.2864204398984204	0.9928251504638874	0.0241016540658814
0.7735555066704632	0.4965938427230187	0.2561462503905748
0.2770761105176264	0.0040314328276975	0.2514588708272666
0.2212793092404020	0.5030768356734766	0.0030330352203687
0.7219855615107249	0.9936741513359644	0.0038735518840008
0.1296390887624440	0.3831169224829167	0.7140783961285724
0.1121524675616712	0.2114492540223169	0.7232645940166729
0.2461758983498945	0.1148715580634758	0.9763049356729341
0.6104861541005817	0.9200921735439285	0.7228079101670224
0.6352076477561666	0.6683942077982620	0.7153283765908323
0.7357272698611205	0.6162103635770574	0.0345615092658174
0.8707743215871013	0.6142628170940276	0.9675350938983552
0.8820117574709682	0.7818197371711828	0.9717570297619627
0.7502831327956203	0.8797813951180380	0.7308214611151852
0.3867120415137853	0.0811516412377097	0.9710967851893113
0.3673269156611971	0.3216816028377277	0.9634019893892811
0.2660894322700164	0.3778021010467313	0.7784962146093730
0.8859944262393077	0.4152864854140513	0.5346604382638068
0.8671681206646443	0.1737861349093068	0.5427619964619738
0.7648899069501314	0.1202074197382855	0.7228306260869619
0.3684364417882223	0.8850273010002294	0.5403541987279216
0.3817004415757842	0.7126674627733052	0.5329312038534787
0.2495476694734647	0.6150359163448131	0.7795602772650262
0.1095571105194350	0.5805509135878621	0.7835905985148861
0.1251315868397440	0.8242999694294062	0.7908341069306229
0.2334476959008141	0.8749861953495535	0.4721723844315409
0.6301032540932562	0.1123780033127720	0.7900994112494768
0.6109256504730430	0.2869204553563934	0.7828991915309835
0.7440056670949918	0.3839371666623810	0.5294290098227157
0.8941005737553805	0.5678223664929541	0.8000144993646542
0.8651393879996346	0.8178997853510144	0.7992368328223907

0.7785664592896923	0.8769179712835690	0.5110126469809355
0.3591045288918049	0.1075515467673099	0.7967721865690871
0.3830990506254217	0.2999825904365394	0.7876234388570813
0.2374438838161959	0.3742927400723314	0.5056414220860720
0.1057279219937822	0.4235523529696668	0.5443360052144400
0.1336105804566552	0.1777461613491819	0.5511668805690284
0.2165522058596874	0.1217745200641551	0.7546473161942971
0.6352799790645396	0.8982068598141574	0.5484617137339755
0.6155791464940487	0.6957279381260620	0.5272772173072381
0.7661358025542716	0.6229305425176416	0.7664510817169023
0.1422692980028734	0.6097799417220048	0.9557170086353616
0.1171273660211494	0.7972512444923872	0.9666125871405348
0.2573660119490339	0.8730853605701165	0.7553296540682556
0.6085134984093991	0.0717507543611817	0.9601028909584427
0.6337736399148556	0.3194956433829459	0.9552510274910572
0.7157550760412019	0.3756639436755222	0.7542122696944068
0.8571062183408643	0.3851286048432404	0.7068823165646521
0.8815049035659402	0.1990765808348601	0.7182421359547511
0.7371552210280248	0.1245652203307259	0.0044569747568834
0.3865668878065390	0.9259812155674949	0.7102484668293343
0.3569156910823876	0.6800706391302798	0.7028697635787722
0.2826323739184801	0.6208568834028183	0.0052299058302865
0.2522154460487495	0.2483042276037094	0.7437878279835052
0.7475408381154366	0.7497232479513853	0.4870748040689383
0.7610796572067142	0.2561957914174163	0.4879230988749519
0.2327847519188343	0.7435172718447028	0.7414412108062359
0.7403721277055043	0.7516614002876694	0.7791843032059815
0.2577541319058410	0.2435187786566967	0.5158249295672895
0.2400362244927643	0.7457115837421542	0.5122356014084540
0.7508979408680920	0.2484935908116927	0.7638345407844181
0.9941502623539825	0.3930590545718947	0.6506239560080948
0.9990213944864398	0.1908710808906228	0.6146861984191401
0.5014006714812794	0.8959119226126250	0.6100355457188262
0.4923309053738450	0.6898261496716293	0.6435076816786364
0.0048680978432512	0.6034567356005290	0.9035804012107453
0.9971012734345237	0.8035385920348385	0.8683484862120794
0.4954414870774991	0.1036411851044721	0.8561876577185190
0.4993496340119772	0.3040871763479043	0.8935045562695185
0.0846880203979303	0.2978142034741115	0.5906892147399655
0.5951845367727220	0.7980311609699295	0.6571398152053050
0.9136389202620816	0.6965330783383156	0.8392143804343234
0.4055507911883125	0.2021410201052558	0.9063220353789698
0.9075843704424885	0.2935150040455139	0.5957563098816606
0.4075257814805831	0.7998433081789866	0.6657924576333640
0.0913144315339487	0.7025595183604806	0.8456118148952472
0.5873367709854915	0.1980578770716360	0.9143429301407049
0.1730463076785160	0.2064129392859114	0.8833642161822312
0.1573609828803746	0.4297810201599403	0.8760324785104763
0.6734670022733548	0.6675858997857983	0.8874593360753936
0.6758659148899456	0.9043160762430731	0.8823082107571761
0.8198044893269106	0.7878094313572067	0.6350840860898473
0.8442014678603343	0.5655722942584948	0.6331133576045587
0.3255401167631242	0.3247383560491888	0.6303002646775708
0.3167726181276305	0.0932249937382276	0.6298780166585759
0.8232705255892316	0.1696969699382461	0.8747632708400486
0.8209568853339647	0.3983774915112568	0.8756224948568033
0.3232680489612469	0.7047829452164112	0.8738219738553953
0.3410775382490193	0.9311606736498751	0.8790909937586850
0.1729817187675539	0.8281487261524998	0.6239412729468076
0.1771587313667784	0.5935797804106024	0.6246104453876303
0.6724146938836515	0.2925123264721874	0.6236946517454908

```
  0.6569156781937707  0.0636986768069440  0.6292935090008444
  0.2109881363624891  0.4930592473769491  0.7311850605209024
  0.7145362267470142  0.0035992602917503  0.7750591421418354
  0.7859876412141195  0.5060122667377831  0.4882061823783178
  0.2837433573840111  0.9925381843063067  0.5211987521018246
  0.7786832211718913  0.4919939329955087  0.7573318089990863
  0.2740658962242340  0.0034306704358841  0.7538092482596629
  0.2154105457799003  0.5041161715743149  0.4987999570156703
  0.7239177984037869  0.9965558802437091  0.4971475414382254
  0.5708728936590841  0.5523650479299267  0.3700261376326840
  0.5196838205786356  0.5913008182306646  0.4167792824054333
  0.5881759826864186  0.4877966286758163  0.3993805831330609
  0.4823260516724114  0.5647894086025533  0.4900431027417588
  0.5518293979036364  0.4609588914558152  0.4730050245363623
  0.4977362201380624  0.4986328382576073  0.5200227031292863
  0.4484357288140620  0.4635035362577148  0.5906736387838518
  0.4821258918382316  0.4890652745313979  0.6798668406689166
  0.5365736486766156  0.4514496224190147  0.7261142043705084
  0.4524499059356795  0.5459590983153660  0.7237468726285847
  0.5595688982612547  0.4698809965641220  0.8117292963474064
  0.4759968491148461  0.5637410124228739  0.8087944067160288
  0.5281751182491214  0.5251228994919578  0.8556511479873559
  0.3672027826318490  0.4779947871901868  0.5786099470992504
  0.5468641201801353  0.5442110510525696  0.9508269598304374
  0.6014160518932870  0.4991784216208560  0.9994029056464395
  0.6547776765204095  0.4985777698946829  0.9648431493874868
  0.5811588426814511  0.4476068452105079  1.0034718146182537
  0.6110415178558765  0.5173331614286334  0.0682018967000143
  0.5669631849818867  0.5959994950830050  0.9495534849208719
  0.4946598662040445  0.5466610514275547  0.9887242344242895
  0.5085495121657585  0.6422420555050414  0.3955012942097711
  0.6286248439120652  0.4581697055887032  0.3628213255630761
  0.4421821776247682  0.5951193673568734  0.5257380122709971
  0.5628602097646048  0.4100351787007158  0.4942845380542513
  0.5269260824672003  0.5172947978780770  0.6050717260095230
  0.5596713180701779  0.4072392611535907  0.6939688425187388
  0.4102892108120266  0.5755615087803428  0.6908342568552839
  0.6011632887900725  0.4400933267909086  0.8458461768440053
  0.4526428109258185  0.6077416382652153  0.8411835709881630
  0.4586651526590168  0.4096615282381849  0.5859059674160730
  0.3556213227326110  0.5315729852292358  0.5785771702338963
  0.3488163524320821  0.4584291863001372  0.5134597540278064
  0.3349172480828009  0.4548510298744041  0.6326187850528210
  0.5975042126702947  0.5727992797824242  0.3104593107782891
```

MOR-Al-T4-I4^{+}

```
   Al     Si     O     C     H
   1.00000000000000
     18.1842269897460938   -0.0090872207656503   -0.0001607598969713
     -0.0105508984997869   20.0691013336181641    0.0015994716668501
     -0.0001797722798074    0.0011982563883066   14.8440780639648438
   Al   Si   O    C    H
     1    95   192    16    19
Direct
  0.5862149297146572  0.7114360872893852  0.6465549312820242
  0.3143812169472147  0.0701922228260940  0.0367327291974440
  0.3020985521192029  0.3150166148098784  0.0401110305565402
  0.8128064380879791  0.5715404297014239  0.0360666442387961
  0.8087386509156467  0.7964300493727980  0.0376714386201092
  0.6901454160738317  0.9224630883603532  0.2870645588420786
  0.7054858661451573  0.6776779804660642  0.2871680470494660
```

0.1938538418360765	0.4208536453382949	0.2875412384849105
0.1944874391670021	0.1968400488318704	0.2871420331982640
0.6928695335658025	0.0731063798263020	0.2388521380535309
0.6933898386545644	0.2982924651447728	0.2396075133481878
0.1899034184130310	0.5707201767997677	0.2384636550999227
0.2011965424222570	0.8150844938023792	0.2367523682287921
0.3115719730054895	0.9200564736331572	0.4882346985161983
0.3096844856844166	0.6971640901871902	0.4888510203737165
0.8133676040353434	0.4252575124120522	0.4911661490294607
0.8019095283003869	0.1794151904254469	0.4870360187773237
0.7068298894832928	0.9152491165756430	0.4951350380907906
0.6951461354201726	0.6792504098979381	0.4958055222521106
0.1862997336530606	0.4315355396259225	0.4932720828219436
0.2085038552788449	0.1847762800042793	0.4934620745261854
0.2966525359902820	0.0799831101173627	0.2440699927604245
0.3104300692598953	0.3138269407033726	0.2477321143165686
0.8191656012913320	0.5624886915998499	0.2387929704812338
0.7971334851530074	0.8091490467005232	0.2428801293524610
0.3177509119973849	0.9313645451601676	0.2828363631442594
0.2949385753091761	0.6858704301700225	0.2825619576436059
0.7968179047652091	0.4152592384533772	0.2827360354447845
0.8090804931871167	0.1810222068962479	0.2790688923670888
0.6871671721506971	0.0624416419178975	0.0336893620587256
0.7078820008843743	0.3094041569830931	0.0333538913678020
0.2091243049491684	0.5806549533799886	0.0308758581487786
0.1958666538072298	0.8124924272270132	0.0293661090167553
0.0845919493778922	0.3744470470641647	0.1358531566501029
0.0881616095695523	0.2197504256988828	0.1308720285084982
0.5886138634576901	0.8706707500862690	0.1433109012225545
0.5848291023916105	0.7107325435626084	0.1497089415463800
0.9191218039028098	0.6203882675481728	0.3857675681232905
0.9150189013914466	0.7757155675444861	0.3830290363128828
0.4157161476684853	0.1221052293534193	0.3933135127220364
0.4191686558687135	0.2809528796501815	0.3978214089465166
0.9161651398657903	0.3728233256727821	0.1341653902860126
0.9190604484852980	0.2138799896031827	0.1291489586910843
0.4201906140262701	0.8739948697352259	0.1403312657049027
0.4162990397055775	0.7192903719256509	0.1459358139427063
0.0879889013734322	0.6220955514463197	0.3821607700835168
0.0847161095228262	0.7811283892351949	0.3796172394348642
0.5841907094560729	0.1198331020505918	0.3909820939809246
0.5879169368377870	0.2748267726003775	0.3959521662045463
0.3143022204411960	0.0697209357397267	0.5368601679130359
0.3018933490769171	0.3146258186683268	0.5401824165629380
0.8120681764715213	0.5744812280686701	0.5412710668366740
0.8079694347061347	0.7970841956551814	0.5374068531960251
0.6904531796279695	0.9246202082021709	0.7870419101245244
0.7053377042394855	0.6769366058864668	0.7930942149001980
0.1933477568149068	0.4207140713604859	0.7871571712173855
0.1943892169640666	0.1967662282970963	0.7874403910213046
0.6953600717611227	0.0727975431994179	0.7380790053408725
0.6931861545910037	0.2983184315032156	0.7394547050064337
0.1890565329053098	0.5705936670510969	0.7393169705711553
0.1997963449764101	0.8159882816513814	0.7382553509874022
0.3115483248095484	0.9204417597371232	0.9880671522063745
0.3122308029361831	0.6964356254021771	0.9896440637229094
0.8147387370136245	0.4229209386355121	0.9883930834602428
0.8021496738951100	0.1798018121199033	0.9866237623723407
0.7075980507240008	0.9143803570639039	0.9946277657235705
0.6943295521554450	0.6778121409860230	0.0015042068570266
0.1864327438173984	0.4316832148166602	0.9926176322856040

0.2082933236144089	0.1852814565525270	0.9937742391012747
0.2969426390255217	0.0797120685940059	0.7444553782105942
0.3102558141755941	0.3137499657341927	0.7477146502510962
0.8235506717362799	0.5615738784987292	0.7461523048163389
0.7956201643896625	0.8070986349027992	0.7448659374143283
0.3172987667932827	0.9313542958149492	0.7832177678753379
0.2930229350084683	0.6858527268170770	0.7836719288516365
0.7984554177692564	0.4118630312498812	0.7817942436675532
0.8097332484125553	0.1821384074739255	0.7783904376487493
0.6864345159216387	0.0623784260006008	0.5324062220955728
0.7084831126566600	0.3091138213882119	0.5336137605612480
0.2070905829805103	0.5801091545614493	0.5311511690820208
0.1941361368335442	0.8133147735674386	0.5293819079727149
0.0844680158525915	0.3734133737641635	0.6356087154555294
0.0883063440440454	0.2186627084745403	0.6306037672828301
0.5890382859627791	0.8732374925489464	0.6446964973608749
0.9227642550173193	0.6191355030785327	0.8862431574221693
0.9157254694271104	0.7730239094542530	0.8792017789984566
0.4167309593784447	0.1213300986432850	0.8928314885851723
0.4191803269165224	0.2804186398377072	0.8978577139016687
0.9164939600674155	0.3706499654549941	0.6316756725869821
0.9196195857335062	0.2129076886345217	0.6277550474468229
0.4197836981148684	0.8730522500193247	0.6406486950839928
0.4150302209568850	0.7181174775615167	0.6464722525391963
0.0894692834650860	0.6229353680930783	0.8820963626734052
0.0846828747421307	0.7808532029046216	0.8788947844323841
0.5856056006204844	0.1188852242764439	0.8898547836141423
0.5883010105589362	0.2741219980233430	0.8959333378287320
0.1336586485432330	0.3815583859182203	0.2267406560838293
0.1183376031947037	0.2120831562007563	0.2337727503075951
0.2511623313049248	0.1139861582780521	0.4863674109489451
0.6122886586132334	0.9096597985262131	0.2352646179630342
0.6290761126196933	0.6742829252746013	0.2306243137917008
0.7381603659156599	0.6186288072196822	0.5493085639515767
0.8728240660803184	0.6103666476763877	0.4779366900174044
0.8863150397963663	0.7854248729707997	0.4860124610323961
0.7538500766729191	0.8798973414466871	0.2355352569088761
0.3918359135613767	0.0817060184816275	0.4839227181249763
0.3742184382380488	0.3204482849031956	0.4762026129237812
0.2693626637286206	0.3775185536401289	0.2932099875677437
0.8914477430302575	0.4127044424508341	0.0433268134107658
0.8744509667551121	0.1751198854872385	0.0502267024047758
0.7684240344112393	0.1167331974482295	0.2347037591548543
0.3722064301326727	0.8818799187618672	0.0488000343331929
0.3893730373130221	0.7106991113600259	0.0416933190580119
0.2536150216632260	0.6139502397758768	0.2891524280347471
0.1123858002441026	0.5820118535645160	0.2912188357821297
0.1293607564745151	0.8205093661526947	0.3010447659043630
0.2365167662265333	0.8763601469166924	0.9833545925497411
0.6329504452633579	0.1124268898269488	0.3000962968469627
0.6167795375146273	0.2843559763671439	0.2930224048367193
0.7501385687737226	0.3807145387356026	0.0395085924747603
0.8892252937778763	0.5718253963238736	0.3058368841135188
0.8647107492552142	0.8181606416092650	0.3133022248544012
0.7819250874912654	0.8716071024147475	0.0130806361094900
0.3630066720303449	0.1042761145378321	0.3091462272980244
0.3880159648373845	0.3016792411228496	0.2992672834771749
0.2435308148926591	0.3715909600517931	0.0114760706424669
0.1143446208972819	0.4239000545773193	0.0569596857950190
0.1390491777123342	0.1770795822123171	0.0619403017857626
0.2217633518384053	0.1215723735436178	0.2647050629965649

0.6412819928935639	0.8913150796790252	0.0603051112156105
0.6157385230205330	0.6853817626118225	0.0526457287875902
0.7630039445597895	0.6241221299724361	0.2458429299098629
0.1395058886356162	0.6017858023642254	0.4667038949908384
0.1164147428712432	0.8017207474300576	0.4781265019145308
0.2608299483788160	0.8710755376790739	0.2649633654799461
0.6127853447723621	0.0707525681165011	0.4704966575762645
0.6391016353690698	0.3178166470388985	0.4650388398398463
0.7219029713170955	0.3733662773102127	0.2622377151084579
0.8638731247902589	0.3910982727911336	0.2188417322677917
0.8862040709164786	0.1938471760801984	0.2272731655637428
0.7409643552077517	0.1246063258897567	0.5135410500261953
0.3899677141032687	0.9235994578412590	0.2187269302629584
0.3630404492037464	0.6771857503056737	0.2128761842323410
0.2816777502692385	0.6210198930520396	0.5078411235074747
0.2587472228817491	0.2481514801834487	0.2556503923697360
0.7418921519128554	0.7455019028601169	0.0147822355658234
0.7639706519514082	0.2528217150440184	0.9963848764145070
0.2378309736278698	0.7414605713318887	0.2450920716688525
0.7407957982664656	0.7519408986375585	0.2785060211887681
0.2645401638706484	0.2417857066772319	0.0318515344856839
0.2492277194093805	0.7476581256944994	0.0245884969502432
0.7564036584634496	0.2462277158291462	0.2713414213307595
0.0000234083836089	0.3935382164192763	0.1592394257571339
0.0046939719751048	0.1917772976955332	0.1257138085022685
0.5050394871038476	0.8922651804983024	0.1181668553566600
0.4987572900236149	0.6899086539189345	0.1554797093104618
0.0041474383155020	0.6014890446227488	0.4064051615155569
0.9989985123605782	0.8022409077235582	0.3753994145763765
0.4993971934860561	0.1020769845130970	0.3671432460308515
0.5047989404763741	0.3035198023058236	0.4050646956573151
0.0906434747239820	0.2978578514541240	0.1003919827912259
0.5941331285566145	0.7909017364390578	0.1596318704444931
0.9112226546258916	0.6973848354208070	0.3532553305792962
0.4101614456458934	0.2015671544665222	0.4143119117412656
0.9104440932152912	0.2935917232466670	0.1133732050426882
0.4131508383207821	0.7973930950992164	0.1756423109477256
0.0948666995427644	0.7014618983471342	0.3633377114410976
0.5909059159247992	0.1969223866214371	0.4256653106565600
0.1786102939003856	0.2059524692836262	0.3942868332987572
0.1604575250319276	0.4299580400839272	0.3884575998944184
0.6891556441635383	0.6573669350584160	0.3901375969387044
0.6829247587543683	0.9000865881108450	0.3912657483935005
0.8296419233486486	0.7903884875420966	0.1439901553711029
0.8482166999712044	0.5575416929840701	0.1351174428203034
0.3288700578759867	0.3279185344415793	0.1427267404417486
0.3232407919461425	0.0933870633850426	0.1408911816498092
0.8283941185336607	0.1681987109296679	0.3841005851779491
0.8219679110554995	0.4029296377923398	0.3866756117492578
0.3251763197276822	0.7066873474495429	0.3814759221778937
0.3443321734194426	0.9307519170576383	0.3872900769790796
0.1756805553423711	0.8278201922835379	0.1336097897917094
0.1814125242723773	0.5927836851796618	0.1339265155571679
0.6768067792160588	0.2899093465314920	0.1326094900979640
0.6594650310929362	0.0651963746563748	0.1378435065073949
0.2131975573084727	0.4925253135942674	0.2427987106077162
0.7116384061108815	0.0011044495982664	0.2823289400955535
0.7921262551293196	0.5010910521122746	0.9874532739998200
0.2914572347092488	0.9921245318265718	0.0331501770962096
0.7770860158285193	0.4931569005504154	0.2651528820027312
0.2775476896867434	0.0019018426661810	0.2603683570509693

0.2268198778913472	0.5021828545353977	0.0135689325214226
0.7281388114498097	0.9922747340530604	0.0133466581842411
0.1335932478579084	0.3808291772761597	0.7263748702422863
0.1182919376378775	0.2113097904749767	0.7336389542196101
0.2509374862397543	0.1143519454342133	0.9867792084684304
0.6133139153836832	0.9180542502684028	0.7329697606359707
0.6381201810940348	0.6677987908664463	0.7261902941640626
0.7376722200452683	0.6144651395088695	0.0455451988015205
0.8721355906807713	0.6120560605614616	0.9758033215643349
0.8820432829229933	0.7785136610812848	0.9805495119394713
0.7531475868382057	0.8782657879030590	0.7398665376222514
0.3919658867237998	0.0815956523317044	0.9836338085914060
0.3741074668651370	0.3197638727595868	0.9755964905032600
0.2695081528078390	0.3779751053070853	0.7919515045076193
0.8908870298720110	0.4134408548923646	0.5438104404706712
0.8734634831445632	0.1725673023976622	0.5516537214292540
0.7692893014028876	0.1186980095946551	0.7318726572957017
0.3724261723114629	0.8817498366780658	0.5481443976960310
0.3852903115423495	0.7114990686749089	0.5420635580928224
0.2508612465451869	0.6142884949107547	0.7922954287097115
0.1109354510404744	0.5806002294189058	0.7917405686027591
0.1266137749762071	0.8226187812651526	0.8003943716283097
0.2362842907670802	0.8762550961497700	0.4830742495638979
0.6342461957451252	0.1113706590530231	0.7989116303397227
0.6164576304867044	0.2841365840828055	0.7924034649872488
0.7493008383891140	0.3810043475292747	0.5404341054304521
0.8972964727966256	0.5666626961500827	0.8083296010411950
0.8684009143654727	0.8166070687465344	0.8078825659118545
0.7803576909626674	0.8730511484113483	0.5195930392136384
0.3642568396897036	0.1035405539694563	0.8084453321262915
0.3882401870996515	0.3020823503730589	0.7989220780844536
0.2434244739848464	0.3714474262435855	0.5126972559569704
0.1138529709359691	0.4231031370798770	0.5567564979812213
0.1395240472922571	0.1762622888602224	0.5618634391088169
0.2225162817775374	0.1217966488471791	0.7656608044232532
0.6378047421498921	0.8978821548090784	0.5585340524929957
0.6161760426198272	0.6895534831148543	0.5386528296430222
0.7681981573752114	0.6206762998187199	0.7723523012077709
0.1445734933266166	0.6063395629682076	0.9646447819259346
0.1194881097943165	0.7981489271390864	0.9769623247242850
0.2590654763324969	0.8718112603183744	0.7676421676735184
0.6150681628488355	0.0699500730653956	0.9691012075715909
0.6390138055265947	0.3176155441094779	0.9642143946435607
0.7206791169770661	0.3739582755632483	0.7612724064536877
0.8631684829235244	0.3844504624470267	0.7169285686610251
0.8869400387854217	0.1966171274162385	0.7273259633598406
0.7435992331665926	0.1231199456453048	0.0148055521879536
0.3881821179023289	0.9234711254956731	0.7179129058776780
0.3579468075167697	0.6768288742949071	0.7110818957607893
0.2853559644986842	0.6208252566996109	0.0128885772619923
0.2583650344273858	0.2484144140822441	0.7554409288760027
0.7471893873813651	0.7457374427053051	0.4986617788344874
0.7654672953929850	0.2533661503827551	0.4971554103278846
0.2348609010113019	0.7421680361363450	0.7505798746326778
0.7440427797400635	0.7503599722003242	0.7903517437166517
0.2646661534731188	0.2414471687187414	0.5318920766193941
0.2442161189511461	0.7466613663863031	0.5214730691332266
0.7568169714894177	0.2473279917235513	0.7716589275619445
0.9997312434847085	0.3921420402952522	0.6586749581687449
0.0050574562630376	0.1900215724037782	0.6250598904521607
0.5040991570829055	0.8930812110263543	0.6205348783415746

0.4948477860186642	0.6875914595025001	0.6568281055212383
0.0071302178879135	0.6021424301777537	0.9134915789279733
0.9989397045624369	0.8019612280653136	0.8802022157711195
0.5005185301332348	0.1010405550072118	0.8674940568215452
0.5049781290966295	0.3029821870003904	0.9020716686522956
0.0907948976941429	0.2968139805201492	0.6002052240203616
0.5997647901826206	0.7961809343683758	0.6650190161045961
0.9158708450040579	0.6948752001285705	0.8479911060431959
0.4109187260280894	0.2009246398929929	0.9129972362040429
0.9127238220771006	0.2921001801137875	0.6062137641910395
0.4096776378399578	0.7969517226484431	0.6758430169504031
0.0938980682633745	0.7016726213529074	0.8581998911252835
0.5917493491249557	0.1960654764116075	0.9246250374234979
0.1784180990485267	0.2062241614972400	0.8944081356979942
0.1598692067818543	0.4293788359182396	0.8879658435699187
0.6780618051231895	0.6638080154884579	0.8965591209487789
0.6796003705310415	0.9035871392628760	0.8918790897018336
0.8215415305792843	0.7849925687389202	0.6444193093435905
0.8478584960102434	0.5639402943595985	0.6407701623258490
0.3301933215270017	0.3274741147475139	0.6426186178365226
0.3227206408802206	0.0930349181952784	0.6409882165467968
0.8279852818364570	0.1680955838655420	0.8833975584445122
0.8237126473590600	0.3972768894138430	0.8851939732786620
0.3260833541070310	0.7053975143144195	0.8822850495287081
0.3449453313475613	0.9307811547672926	0.8876087922089135
0.1762364406329339	0.8281183867657522	0.6342839465651735
0.1817966649560439	0.5934488954170042	0.6348194397055016
0.6762185420946074	0.2896888257323988	0.6324447982360277
0.6616160723043949	0.0629343771832533	0.6376203949750924
0.2125531403303480	0.4923495920278354	0.7422335805461494
0.7178271704869745	0.0022413770969406	0.7835411470522863
0.7891584024854760	0.5026885232739882	0.4974558860226610
0.2911811092142709	0.9918372199740197	0.5329558673780596
0.7826319974141003	0.4903873204188781	0.7652170624650385
0.2769333231859918	0.0020845223006521	0.7619302608134083
0.2270640870998069	0.5016808660236998	0.5157171444353603
0.7286416441203729	0.9938691840429764	0.5067517288511066
0.5858765961601490	0.4812814815227793	0.7460921536254563
0.6149955471463744	0.5156685418399329	0.6704361285656875
0.5371419241262886	0.4265002138875634	0.7333479873792177
0.5947236273665177	0.4977429674571846	0.5850995415035788
0.5158486257393013	0.4083560653298848	0.6485621092208643
0.5416659894659430	0.4440205360345815	0.5681438170065443
0.4731812418262378	0.4694191387933632	0.5101607187703506
0.4931189913527430	0.4872104998944536	0.4135239900650324
0.4388721447257767	0.5175037700232185	0.3597773667233178
0.5612660121219161	0.4731517422372920	0.3736203521141278
0.4526371850341935	0.5334019406710498	0.2698473295313900
0.5755261011674525	0.4896166917847045	0.2837104255073447
0.5212764386978703	0.5200188175210502	0.2315806221530066
0.6022625640435778	0.5050410757255920	0.8385958488255373
0.4343790509660029	0.5261281024727340	0.5607186233048118
0.5422058816580854	0.5561593220788918	0.8662278073284723
0.5545153404600215	0.5747107773778163	0.9341738832783667
0.5420552973482997	0.5986129794014343	0.8195626409156785
0.4875212922767437	0.5327706575915295	0.8664177372004099
0.4232306615332957	0.5118516601916453	0.6309329484411247
0.4678499360526999	0.5717647511047009	0.5610359444961472
0.3814691247325862	0.5376798818303393	0.5291267250292058
0.6562992285024034	0.5294747278603638	0.8406038579626456
0.6018705970669048	0.4629407196419597	0.8861316648917482

```
  0.6515405592473548  0.5579401812437681  0.6813958727323436
  0.5167305326887481  0.3996650371250576  0.7921757118611221
  0.6161031453319193  0.5252077777275900  0.5272865196741113
  0.4771001361951691  0.3673499212093476  0.6385794344065178
  0.5671958412138732  0.4061969103046389  0.5230036936373568
  0.3849808503138036  0.5287299937807655  0.3882299021495260
  0.6051401018691558  0.4491353859703714  0.4117882175474857
  0.4096901087532805  0.5572052955768997  0.2300615764594615
  0.6294864534794734  0.4787539106758637  0.2553058823211773
  0.5324010671379172  0.5336731015748046  0.1617655334065047
  0.4363413089120737  0.4258416772888257  0.5076649102110105
```

MOR-Al-T4-TS8

```
   Al     Si     O     C     H
   1.00000000000000
    18.1842269897460938   -0.0090872207656503   -0.0001607598969713
    -0.0105508984997869   20.0691013336181641    0.0015994716668501
    -0.0001797722798074    0.0011982563883066   14.8440780639648438
   Al   Si   O    C    H
     1    95   192    16    19
Selective dynamics
Direct
  0.5871950387954712  0.7479045987129389  0.6588524580001928
  0.3148823678493501  0.1034002825617826  0.0484460964798935
  0.3042129874229431  0.3477149009704680  0.0508722215890891
  0.8111656308174131  0.6030626296997166  0.0497668683528908
  0.8088895678520203  0.8271297812462016  0.0497338436543947
  0.6895365118980408  0.9555145502090462  0.2988728880882316
  0.7034505605697633  0.7087053060531737  0.3004462122917234
  0.1943347901105881  0.4539819061756206  0.2987007200717926
  0.1947635263204575  0.2304773479700149  0.2991016805171970
  0.6943363547325134  0.1061207950115221  0.2521547079086349
  0.6966838836669925  0.3290120065212317  0.2544366717338607
  0.1895154565572739  0.6041203141212583  0.2495782673358951
  0.1998314112424853  0.8479461073875570  0.2469545453786882
  0.3120489418506622  0.9543995857238913  0.4974952042102872
  0.3084730803966574  0.7312510609626990  0.4993352890014652
  0.8132179379463220  0.4554765820503319  0.5056412220001311
  0.8023581504821781  0.2112460285425187  0.5012186169624333
  0.7046493291854858  0.9489573836326956  0.5076840519905095
  0.6944367289543268  0.7103819251060756  0.5080748200416619
  0.1876453906297714  0.4647167921066364  0.5042094588279729
  0.2098986506462097  0.2188244163990057  0.5048462748527632
  0.2976493835449219  0.1131356284022361  0.2557428777217910
  0.3107204437255861  0.3473098874092151  0.2586320638656661
  0.8189603686332705  0.5944641828537107  0.2529891729354861
  0.7944587469100954  0.8398537039756918  0.2556374073028567
  0.3164118528366090  0.9645511507988331  0.2919614017009739
  0.2946049273014069  0.7187541723251518  0.2933875620365201
  0.7980309724807737  0.4468344151973813  0.2968931496143357
  0.8116141557693484  0.2122572809457851  0.2940431237220813
  0.6858987808227540  0.0933235064148925  0.0477296859025961
  0.7086603641510161  0.3386342525482269  0.0478075221180922
  0.2084607481956482  0.6135284304619016  0.0418836139142522
  0.1947858035564440  0.8455900549888862  0.0389578454196454
  0.0860125347971917  0.4058585464954516  0.1473585963249207
  0.0888600870966921  0.2512139678001494  0.1427666693925858
  0.5875235199928294  0.9027907848358442  0.1573196649551394
  0.5841063261032247  0.7439643144607722  0.1634762436151506
```

0.9194150567054749	0.6525334119796931	0.3997150361537986
0.9134277701377869	0.8086348772049233	0.3961389362812095
0.4171447753906250	0.1538831889629373	0.4054291248321593
0.4200120270252293	0.3131182789802640	0.4089924693107609
0.9167880415916601	0.4045574963092830	0.1480612307786943
0.9194461703300476	0.2453714609146190	0.1441222727298737
0.4191519916057587	0.9065282344818357	0.1496974825859094
0.4153889119625093	0.7517414093017820	0.1561504900455492
0.0881858468055725	0.6560359597206294	0.3935145437717496
0.0830229222774508	0.8153011202812551	0.3899968266487141
0.5853303670883179	0.1517271399498016	0.4061319828033496
0.5890249013900757	0.3065907359123324	0.4107644259929717
0.3141757547855377	0.1031351834535636	0.5476925373077400
0.3036029636859894	0.3482719957828582	0.5514452457427979
0.8114250898361206	0.6050972342491270	0.5540909767150887
0.8058817386627231	0.8288668394089033	0.5502550601959229
0.6905115246772850	0.9561806321144253	0.8017923235893346
0.6996625661849976	0.7056040167808710	0.8042982816696308
0.1948815584182746	0.4540892839431897	0.7984899878502023
0.1940185725688935	0.2317229509353638	0.7998348474502563
0.6975882053375244	0.1035586446523673	0.7538949251175023
0.6988695263862611	0.3255791962146833	0.7542231678962715
0.1887291520833974	0.6037093997001872	0.7497556805610665
0.1988801211118717	0.8489193916321087	0.7478621602058418
0.3103869855403912	0.9541519880295086	0.9990872740745755
0.3116714954376220	0.7296611070633054	0.9992201328277734
0.8138597607612703	0.4541180431842948	0.0033656358718872
0.8027274608612061	0.2095785588026070	0.0003879070281982
0.7071583271026624	0.9445897340774679	0.0093606906011701
0.6932228207588198	0.7099456191063107	0.0121225016191603
0.1881705373525620	0.4642116129398467	0.0038204193115235
0.2090251892805099	0.2184159457683574	0.0055838823318482
0.2951791286468551	0.1127372533082982	0.7561196684837349
0.3110886514186861	0.3478808104992003	0.7592627406120308
0.8199660181999208	0.5920572876930327	0.7590107917785750
0.7915283441543599	0.8362502455711607	0.7576435804367165
0.3134343326091767	0.9662757515907295	0.7942641973495491
0.2926248610019684	0.7192841172218413	0.7936626076698422
0.7996083498001099	0.4429912865161967	0.7964254021644698
0.8154199719429061	0.2107257843017579	0.7932927608490087
0.6871228218078617	0.0947680547833466	0.5479885935783483
0.7091459631919862	0.3395360410213560	0.5482245087623725
0.2082187235355400	0.6135904192924635	0.5420421361923347
0.1932987570762657	0.8470915555954123	0.5384634733200078
0.0864720791578295	0.4060256183147502	0.6469087004661709
0.0899539142847064	0.2516822516918242	0.6416870951652535
0.5898979902267458	0.9076750278473044	0.6587459444999791
0.9209852814674384	0.6494831442833082	0.8989062905311592
0.9142967462539705	0.8038732409477413	0.8921175003051937
0.4167402386665361	0.1525623351335555	0.9034237861633428
0.4207640290260360	0.3117199540138303	0.9079543352127286
0.9178168177604833	0.4024355709552837	0.6469068527221821
0.9215829372406006	0.2436231374740654	0.6420075297355652
0.4168061017990114	0.9099289774895001	0.6536687016487218
0.4113182425499007	0.7581878304481596	0.6585046648979195
0.0881751030683518	0.6544997692108334	0.8932171463966475
0.0832144245505339	0.8129210472107077	0.8883455395698676
0.5856223702430745	0.1487511545419738	0.9041695594787598
0.5904690027236938	0.3025237917900085	0.9113095402717707
0.1346368044614814	0.4135204255580974	0.2385420501232149
0.1178694218397141	0.2433098852634490	0.2461319714784664

0.2528524696826936	0.1483401209116005	0.4957232475280831
0.6112598776817322	0.9446830749511961	0.2469102293252972
0.6272143721580506	0.7046838998794746	0.2431907057762175
0.7376610040664671	0.6496574282646362	0.5603972673416145
0.8733410239219666	0.6403089165687741	0.4916156530380315
0.8848234415054321	0.8201310038566733	0.4988816678524078
0.7514232397079468	0.9105901718139914	0.2476280033588461
0.3927807211875916	0.1142663285136241	0.4966847002506310
0.3758690357208252	0.3527323901653379	0.4876360595226394
0.2707561850547791	0.4118720293045074	0.3035757243633274
0.8909219503402710	0.4446606934070662	0.0579736642539502
0.8725085258483888	0.2056242078542754	0.0681719630956657
0.7698291540145874	0.1497483104467436	0.2465380281209986
0.3721220493316651	0.9146847724914694	0.0573886409401904
0.3887005150318125	0.7428628802299724	0.0519317053258427
0.2530362308025360	0.6471117138862754	0.3009199202060703
0.1118763983249672	0.6163486838340879	0.3019287884235382
0.1273270249366756	0.8535068035125876	0.3101183772087101
0.2351552248001101	0.9103004336357382	0.9949028491974072
0.6358934044837952	0.1443072259426155	0.3166836202144671
0.6205941438675018	0.3153737485408879	0.3088236153125834
0.7506227493286132	0.4100855290889812	0.0538087189197548
0.8897940516471867	0.6050500869751093	0.3185104429721836
0.8624237179756165	0.8493680953979779	0.3252766132354740
0.7822436690330505	0.9026468992233563	0.0268239062279467
0.3651261329650879	0.1346048414707213	0.3209300041198734
0.3876819312572569	0.3338835239410414	0.3107911050319728
0.2471686303615570	0.4052985906601073	0.0207163505256179
0.1164610534906401	0.4548976421356322	0.0683527514338494
0.1404243111610413	0.2086026370525421	0.0745099037885667
0.2238622754812291	0.1556107252836250	0.2782756984233909
0.6411215066909790	0.9187855124473633	0.0735470503568669
0.6166707277298106	0.7218505144119441	0.0661238655447971
0.7618650794029236	0.6555274128914053	0.2605122327804610
0.1420408338308335	0.6365022659301935	0.4762644469738113
0.1151177510619168	0.8380632996559430	0.4874974787235293
0.2588039338588715	0.9046027064323712	0.2748791575431894
0.6129825115203857	0.1031737700104731	0.4868800044059802
0.6384180784225484	0.3489285111427396	0.4822544455528359
0.7237340807914734	0.4050273895263745	0.2734829783439636
0.8664715290069581	0.4239396750927074	0.2341652065515548
0.8896208405494690	0.2250915765762389	0.2438066899776497
0.7407373785972700	0.1579018235206641	0.5308521986007780
0.3882260620594077	0.9562479853630306	0.2274006158113504
0.3624601364135743	0.7096882462501651	0.2235512435436251
0.2839930057525635	0.6545404791832153	0.5221335887909025
0.2573271989822398	0.2827413082122863	0.2655368149280550
0.7439496517181400	0.7762358784675781	0.0200255122035744
0.7640975713729858	0.2826246619224637	0.0073031187057496
0.2374984920024872	0.7748164534568966	0.2573711276054427
0.7380492687225341	0.7831980586052061	0.2929744124412541
0.2648546695709239	0.2751740813255372	0.0439051799476156
0.2488920390605934	0.7812787890434443	0.0335066579282286
0.7607852220535281	0.2787288129329741	0.2881027460098315
0.0013631664915010	0.4247792661190117	0.1701861470937753
0.0051528499461711	0.2238960117101708	0.1364401727914845
0.5044650435447696	0.9242340922355938	0.1294547468423866
0.4979633390903474	0.7226292490959351	0.1666315644979501
0.0049061118625105	0.6345033645630066	0.4195446372032217
0.9970799088478091	0.8358061909675794	0.3865915536880546
0.5009840726852417	0.1335262209177033	0.3803214132785797

0.5057415962219221	0.3353579938411795	0.4139493703842227
0.0923422873020181	0.3292694985866630	0.1122014299035092
0.5920634865760803	0.8236379623413328	0.1796155124902735
0.9102652668952944	0.7298096418380738	0.3691911101341248
0.4109236598014832	0.2335289567709005	0.4246957004070286
0.9097293615341391	0.3251024186611258	0.1284402459859871
0.4115120172500643	0.8299541473388958	0.1850882470607760
0.0934609621763230	0.7355524301529059	0.3754640817642216
0.5908064246177653	0.2287145256996243	0.4409037232399058
0.1792455017566682	0.2406511008739523	0.4062146544456530
0.1610790938138962	0.4632230103016018	0.3996243476867740
0.6850807666778566	0.6878661513328735	0.4028887152671818
0.6815317869186402	0.9339737296104718	0.4034324884414721
0.8259051442146391	0.8191888928413631	0.1568885743618038
0.8466628789901735	0.5900340080261254	0.1489037871360804
0.3302007615566276	0.3613230884075256	0.1537568122148538
0.3235507011413640	0.1273438334465056	0.1523896157741571
0.8288497328758363	0.1966699957847643	0.3989444971084677
0.8214902281761189	0.4330687224865031	0.4011770486831729
0.3262374401092529	0.7387378811836512	0.3922879099845966
0.3439087569713596	0.9642067551613150	0.3960666060447697
0.1744296401739129	0.8593532443046753	0.1435270309448242
0.1817597597837448	0.6266872882843108	0.1450640857219721
0.6804286241531373	0.3177391588687986	0.1478595882654220
0.6589292287826539	0.0997434332966827	0.1519394069910079
0.2119580507278443	0.5258199572563291	0.2533199489116671
0.7140008807182315	0.0333434641361242	0.2928727269172672
0.7896427512168888	0.5319706797599882	0.0034672021865845
0.2912034094333649	0.0253739263862376	0.0460280328989037
0.7783104181289676	0.5249347686767668	0.2808149755001121
0.2765222191810608	0.0351717732846737	0.2696815729141239
0.2272919118404401	0.5351879000663847	0.0257211551070216
0.7261908054351808	0.0222159400582320	0.0314244739711285
0.1358924955129623	0.4132505059242320	0.7376242280006505
0.1177975237369542	0.2453652322292399	0.7457875013351440
0.2522215843200684	0.1477901488542601	0.9972842335701085
0.6131410598754883	0.9524198770523311	0.7473208904266365
0.6316493749618530	0.6963045597076596	0.7373149394989014
0.7362089157104496	0.6467021107673686	0.0574735738337047
0.8716119527816836	0.6421264410019041	0.9894195199012884
0.8842069506645204	0.8109013438225080	0.9952295422554176
0.7509176731109619	0.9083261489868307	0.7533506155014046
0.3926064074039460	0.1135600134730375	0.9952497482299933
0.3769079744815871	0.3506869673729007	0.9874250292778211
0.2720855474472048	0.4130193293094707	0.8039363622665413
0.8908595442771915	0.4434244334697902	0.5579507946968086
0.8738188147544861	0.2050429880619115	0.5659114718437203
0.7720655798912048	0.1485538482666046	0.7476995587348938
0.3753640055656478	0.9192333817482281	0.5572302341461182
0.3828380107879649	0.7496517896652342	0.5536240339279175
0.2509959042072345	0.6475801467895732	0.8019700646400557
0.1107784733176261	0.6132319569587823	0.8023990988731503
0.1249718517065048	0.8538658022880737	0.8088876605034021
0.2382194250822069	0.9086725711822796	0.4933747053146426
0.6364799141883851	0.1421762704849289	0.8147611618042089
0.6244899630546570	0.3084988892078491	0.8099412918090927
0.7490227818489165	0.4120390117168548	0.5561937689781291
0.8935508131980896	0.5976333022117714	0.8214796781540061
0.8633190989494324	0.8452455997467184	0.8224436044693110
0.7756236791610801	0.9041126966476584	0.5341193675994979
0.3642107546329499	0.1329733580350920	0.8196527957916389

0.3885824680328369	0.3345940709114165	0.8102965950966026
0.2452896982431413	0.4050001204013828	0.5224894881248564
0.1156154274940495	0.4562051296234274	0.5684642791748055
0.1420669406652451	0.2088625878095641	0.5745694637298692
0.2227974236011513	0.1571070104837448	0.7779123187065132
0.6336959004402178	0.9354432821273947	0.5701733231544495
0.6158296465873718	0.7222386598587037	0.5519297122955330
0.7637228965759367	0.6506065726280332	0.7841516137123250
0.1430068314075470	0.6377433538437070	0.9759759902954260
0.1186526566743859	0.8315410614013815	0.9857546687126357
0.2571993172168733	0.9051319956779693	0.7787582874298212
0.6135738492012024	0.0985260829329514	0.9830417633056747
0.6380573511123658	0.3466944098472685	0.9817619919777036
0.7242795825004578	0.4018930196762089	0.7747961282730110
0.8668516874313460	0.4190544486045851	0.7331547737121702
0.8931341767311096	0.2223999947309554	0.7419253587722940
0.7431237697601322	0.1526609212160140	0.0245334375649694
0.3842909038066876	0.9621485471725706	0.7277306914329537
0.3583438396453859	0.7107733488083063	0.7212062478065498
0.2839920520782470	0.6542123556137260	0.0216516125947241
0.2572080492973359	0.2839147448539792	0.7677267789840804
0.7475145459175110	0.7761555910110716	0.5088129639625639
0.7668455243110799	0.2858059108257301	0.5073839426040649
0.2346168756484986	0.7752667665481716	0.7596386671066412
0.7380689978599548	0.7793285846710295	0.7989503741264351
0.2657907605171204	0.2752297222614288	0.5439226627349854
0.2403851300477981	0.7787327766418636	0.5283941030502401
0.7657926082611085	0.2776367068290755	0.7840877771377571
0.0016954997554422	0.4241010546684337	0.6703261733055250
0.0072078304365278	0.2225061953067840	0.6335518360137939
0.5031917691230771	0.9228786230087456	0.6393090486526617
0.4935240149497987	0.7339019775390808	0.6683061122894408
0.0059575480408967	0.6324030160904114	0.9234299659729133
0.9972679018974304	0.8336308002471958	0.8884973526001112
0.5007435083389282	0.1322034150362037	0.8784945011138924
0.5071831941604614	0.3311665058136031	0.9102643132209887
0.0928606092929840	0.3296809196472228	0.6105546355247505
0.6081232428550720	0.8312319517135763	0.6765953302383431
0.9142435789108344	0.7255529165268120	0.8620638251304809
0.4098034203052521	0.2321800440549851	0.9219326376914986
0.9122974872589111	0.3232896029949277	0.6258500218391426
0.3982513248920441	0.8352227807045126	0.6902672648430013
0.0923806950449945	0.7334059476852617	0.8701575398445258
0.5917300581932158	0.2248404771089590	0.9425427913665879
0.1781059652566911	0.2403159588575442	0.9070187211036839
0.1611886024475097	0.4627529382705792	0.8992391824722407
0.6733847260475179	0.6943413019180475	0.9084902405738952
0.6793376207351686	0.9347925782203818	0.9063807725906514
0.8191673755645755	0.8156845569610739	0.6570339202880867
0.8457884788513288	0.5946409106254755	0.6542133092880249
0.3302791416645098	0.3616904616356017	0.6542267799377449
0.3210249841213226	0.1251920014619854	0.6524528264999486
0.8327592015266420	0.1976219564676321	0.8987942337989998
0.8243191838264465	0.4296525716781737	0.9000381231307991
0.3259618282318117	0.7389289736747862	0.8919466137886169
0.3422847688198090	0.9653969407081890	0.8980750441551349
0.1763586848974254	0.8613857030868770	0.6436706781387426
0.1813220828771617	0.6265938878059486	0.6452714800834656
0.6804869174957275	0.3168996870517820	0.6475113630294808
0.6635679602622986	0.0934161841869375	0.6535919904708980
0.2129364907741547	0.5257684588432402	0.7528675198555169

```
  0.7202113866806030  0.0330180600285541  0.7994329929351934
  0.7887595891952638  0.5330209136009217  0.5109573602676392
  0.2892364859580995  0.0257136411964894  0.5414879918098457
  0.7795791625976713  0.5206217169761838  0.7782367467880391
  0.2708589732646942  0.0359788089990622  0.7738537192344808
  0.2278164178133050  0.5352894663810762  0.5263338088989262
  0.7296312451362610  0.0268785152584319  0.5192499160766691
  0.4918190538883211  0.6019067764282366  0.5156483650207524
  0.4582650959491766  0.6064234972000331  0.5988191962242134
  0.5591191053390505  0.5675677061081175  0.5059296488761992
  0.4917728900909424  0.5774372220039492  0.6760298609733582
  0.5905512587231828  0.5344758045615516  0.5792223496279461
  0.5550722579475521  0.5371343991724211  0.6666194821747149
  0.5739341974258424  0.4842411577701640  0.7351076602935799
  0.5516102313995451  0.5015853047371014  0.8306660056114313
  0.6041688323021039  0.5270081758499235  0.8907554149627791
  0.4797433614730851  0.4919403195381276  0.8622525930404782
  0.5852807760238737  0.5416520833969144  0.9795502424240243
  0.4607798457145694  0.5073744058609103  0.9510879516601570
  0.5132944583892822  0.5321558117866696  0.0114972591400148
  0.5342971086502079  0.4210532307624839  0.6989309787750388
  0.4954856038093569  0.5484102964401357  0.1086291223764436
  0.4153295457363174  0.5386107563972586  0.1366954147815730
  0.3982675671577454  0.4862103164196158  0.1302275359630607
  0.3782080411911011  0.5689514279365647  0.0950327813625348
  0.4074618220329285  0.5535416603088469  0.2072801738977459
  0.5314894914627075  0.5179400444030902  0.1520509719848635
  0.5117935538291930  0.6004711389541671  0.1213257238268868
  0.4057001471519515  0.6313544511795286  0.6065551042556763
  0.5868664383888247  0.5672270655632020  0.4407940804958347
  0.4672106504440308  0.5810364484787165  0.7428427338600317
  0.6385554075241087  0.5028460025787443  0.5696148872375496
  0.6070256354046565  0.5792812733370231  0.6456993490670612
  0.6605275273323163  0.5349080562591733  0.8677195310592787
  0.4378173649311066  0.4709950685501220  0.8178647160530219
  0.6274077296257109  0.5607007741928284  0.0252629518508914
  0.4044167101383268  0.4991037845611729  0.9735193848609932
  0.6335442066192627  0.4758570194244389  0.7335996031761352
  0.4755802452564240  0.4306821525097013  0.6871343851089585
  0.5586615204811096  0.4044951498508547  0.6350563168525817
  0.5385275483131409  0.3808684051036907  0.7483661174774352
  0.4668468236923220  0.6260392069816679  0.4574359953403543
```

MOR-Al-T4-I2+

```
   Al      Si      O      C      H
   1.00000000000000
    18.1842269897460938   -0.0090872207656503   -0.0001607598969713
    -0.0105508984997869   20.0691013336181641    0.0015994716668501
    -0.0001797722798074    0.0011982563883066   14.8440780639648438
   Al   Si   O    C    H
     1    95   192    16    19
Direct
  0.5861858401307178  0.7135070895830937  0.6404728179367623
  0.3138033908084026  0.0702922698159765  0.0348296455395288
  0.3027416294225854  0.3155478199701218  0.0379818195967491
  0.8110054298466562  0.5721189425370841  0.0306895237278859
  0.8076242288700687  0.7952704907189981  0.0328501555240761
  0.6892802852749347  0.9221883677110804  0.2807469147514395
```

0.7052040808525382	0.6770073074062812	0.2819198112624583
0.1935798537239232	0.4199249147520690	0.2857116016553974
0.1936246202596859	0.1971447037091437	0.2848010269925684
0.6934593036113837	0.0727980139683514	0.2338280977198359
0.6936689886689614	0.2982334997448158	0.2357998921250777
0.1897230423937855	0.5708335768625300	0.2387246592595612
0.1997114293789477	0.8166452918266446	0.2349289449673703
0.3085528698441499	0.9209312837821154	0.4862534434384155
0.3091576173701135	0.6981922190230107	0.4876768177574708
0.8156625185415786	0.4245090637182463	0.4861994898247124
0.8023743270696837	0.1797370800912546	0.4818226712994095
0.7046374417247819	0.9159721859842691	0.4889634282078212
0.6922309692494878	0.6773713878290307	0.4905428023050768
0.1840947034785425	0.4326649550115371	0.4906744387916983
0.2081612571066744	0.1873591566756053	0.4905681561351961
0.2973633797303364	0.0814188037353551	0.2422994910608435
0.3102698435889786	0.3137014653331872	0.2456164214408433
0.8203515664236621	0.5621849893040739	0.2332587495627615
0.7969149490827134	0.8089687514347806	0.2370538669502060
0.3177389766889251	0.9319328126529307	0.2809006695318831
0.2945077406885047	0.6871491521747428	0.2813431587734301
0.8002108611382016	0.4126870028516983	0.2780182051416826
0.8094421634480635	0.1808299280813500	0.2742142538579438
0.6871052272964493	0.0611938685657968	0.0286195815487642
0.7083932188946851	0.3083268289138027	0.0292339874838092
0.2072657315461799	0.5812923991018730	0.0303027327308830
0.1942984788394697	0.8123234794821181	0.0279252188316464
0.0854622805148183	0.3734416566927510	0.1313144386256043
0.0883399637903187	0.2185356304447717	0.1272204868061338
0.5865506338562023	0.8721553312457581	0.1377253726652575
0.5837311931303192	0.7123910219279688	0.1443496940526446
0.9177029265876997	0.6230071526718913	0.3811603909175461
0.9129008447255841	0.7781615487938276	0.3791148724392325
0.4164054230999692	0.1228798142238557	0.3913720460584765
0.4195459013427849	0.2813276938093424	0.3958440413720710
0.9173120746864808	0.3707286623742891	0.1273316022909627
0.9196514829560171	0.2125203404816236	0.1230823133780879
0.4185029035625016	0.8750750840524508	0.1368828889337759
0.4155384424904395	0.7199964794539905	0.1428072174225795
0.0859635075469182	0.6259753005537485	0.3796442495877258
0.0822279619319225	0.7835486824421621	0.3758697299076795
0.5848660781040986	0.1197172228009828	0.3865986824644370
0.5882676485069456	0.2749740169511665	0.3920323907183043
0.3138611618437972	0.0711217049570640	0.5333689430817843
0.3025700950018862	0.3179170358706505	0.5375946304136551
0.8106303298087180	0.5743021930551772	0.5350295040709381
0.8042697227382926	0.7955527546512154	0.5341628426577054
0.6904605986446040	0.9237747149918869	0.7815860583661065
0.7068989378415687	0.6783918768617152	0.7900642735440566
0.1946123055447667	0.4210681256609759	0.7835586601086564
0.1940933427193665	0.1974408526762909	0.7847097641533186
0.6961287879044927	0.0710913746936302	0.7331345733620078
0.6927936899846660	0.2981197993347476	0.7350944242562195
0.1880842842907585	0.5704357619445042	0.7377123449215525
0.1987021445345550	0.8155808671049806	0.7350105249039951
0.3074208577273052	0.9215781363045989	0.9869422833138933
0.3112842468391591	0.6971167847693306	0.9877344279847289
0.8144178622481467	0.4227415380169723	0.9840224125992478
0.8025895486505862	0.1783988923598884	0.9823178454070818
0.7062126707193326	0.9134990191901340	0.9892846527513992
0.6922400907210556	0.6769073920218170	0.9971570012029138

0.1875232931513550	0.4322306545152949	0.9892116070825845
0.2087810018519175	0.1860741363097703	0.9910352736359883
0.2962087203244582	0.0794017204062756	0.7414003624357023
0.3107395949238779	0.3136497118523642	0.7448948265646493
0.8229825558295656	0.5618362461306742	0.7404510181835547
0.7967889667050831	0.8066331940283290	0.7415739432654612
0.3147340143554431	0.9318352347241083	0.7813131598912156
0.2920939371281707	0.6859924514460911	0.7812668764286457
0.7978820031230857	0.4117257410915984	0.7774735811913556
0.8094020420373715	0.1821070405054444	0.7742180427324742
0.6879965494530732	0.0618789336938224	0.5267326616438421
0.7090101497371537	0.3094010995767074	0.5289412965165841
0.2028165361859384	0.5842968985723844	0.5295220431319736
0.1920510396507041	0.8142055323338452	0.5263645241276040
0.0841871979019974	0.3729101839311018	0.6344120249749229
0.0880071263458666	0.2182088388692942	0.6288402230528639
0.5871214253909248	0.8761347380775305	0.6369740588049668
0.9212290151990502	0.6196591185436117	0.8803296877673367
0.9157316124592793	0.7732740896539454	0.8734984920348677
0.4157746836518316	0.1193593335058428	0.8908682192150020
0.4187163438305469	0.2778244495303299	0.8963240568777450
0.9168989867387418	0.3703221488635683	0.6290444809146576
0.9197805156033761	0.2126937669472510	0.6233297630472726
0.4172501338350030	0.8741420496364416	0.6400333834941186
0.4146232132495153	0.7190177547764152	0.6456688841581467
0.0878544887784664	0.6219549555263006	0.8808693859053299
0.0843969861200839	0.7792905235494260	0.8759283687032298
0.5857951633911356	0.1166326665279883	0.8845955334299513
0.5892101420017077	0.2714764959808756	0.8913104855422992
0.1348458605840959	0.3814691348068490	0.2219066417917194
0.1177331248296806	0.2113231673391197	0.2304703665420047
0.2530054971505840	0.1177460551390811	0.4828903075939702
0.6107203376752686	0.9111218122024710	0.2295176900816934
0.6290019678770522	0.6756806994922401	0.2243943262389346
0.7347418257129292	0.6156845989311552	0.5417061874144826
0.8696864594311552	0.6129734249859696	0.4718856260528476
0.8826056345018822	0.7866787436035485	0.4814974454500852
0.7509385660660485	0.8781247148325110	0.2277515092692381
0.3920211751625173	0.0813944060386029	0.4808518479591370
0.3745371597449696	0.3224915680373800	0.4725340106288516
0.2700063622514774	0.3777458821327045	0.2915280099886641
0.8913138698865358	0.4127285333703414	0.0390323651795533
0.8748974692254966	0.1721797916965912	0.0458651555719311
0.7690978460534849	0.1163495011445735	0.2298629154845042
0.3684668674744802	0.8828600418955733	0.0471076222106019
0.3893372205642405	0.7098413024363014	0.0388354666576637
0.2536959930811246	0.6150123199852747	0.2874140664333651
0.1129220560462989	0.5823279095005395	0.2929151121257372
0.1284600522258618	0.8241203788295000	0.2998435614482053
0.2324140519349735	0.8775743658446791	0.9823507296630084
0.6334528768668102	0.1117512124676642	0.2955688638036803
0.6165188773411483	0.2856030447539415	0.2888477675415957
0.7502490056658945	0.3798648533463919	0.0348766564760416
0.8897238609874071	0.5743176066284413	0.3001979395347649
0.8631744811712170	0.8206523958186277	0.3086449002684717
0.7797850366655438	0.8700261492343527	0.0083482094265270
0.3635981808789739	0.1073089431559798	0.3064147590991890
0.3878829375276334	0.3006602041612068	0.2968439815531701
0.2458526494964804	0.3731507282455073	0.0080296211224498
0.1149173639996421	0.4226808336502574	0.0521921230384903
0.1394938507162264	0.1758773487389821	0.0586468461527697

0.2219242405219774	0.1221059395587519	0.2630834724818579
0.6389384633186456	0.8915007570765746	0.0541755841894109
0.6132695849356840	0.6862677642168734	0.0473651355634549
0.7596928593617350	0.6202797641450947	0.2423324252542166
0.1366295799693614	0.6122053231309714	0.4672707146744363
0.1146132801588298	0.8005083116980882	0.4753593397687912
0.2597465709473658	0.8732095229874407	0.2590320453713043
0.6136507925055499	0.0709181207965120	0.4661370837648692
0.6395742547567859	0.3183166055552787	0.4602532981213818
0.7231139043164916	0.3731039303994726	0.2597036340377156
0.8650315430301542	0.3856427950021789	0.2134077517747257
0.8859235337119049	0.1952671615162695	0.2217962884997670
0.7417405396638587	0.1247765680678025	0.5088858869962288
0.3901608233921818	0.9239447364415323	0.2171124431703130
0.3629077674394773	0.6783601021827840	0.2114944646889608
0.2795043813462017	0.6227647976496242	0.5079454552046742
0.2577195579325711	0.2487051474919205	0.2533454440334340
0.7407509512590819	0.7437385118409913	0.0133633919365543
0.7653226855981182	0.2515471504229932	0.9950037235279994
0.2372595943489424	0.7433761047946833	0.2463215131364011
0.7429853003272774	0.7499794929646236	0.2734016197434578
0.2633482155260028	0.2430997250366399	0.0310045688527797
0.2508203777484153	0.7497643330783417	0.0249994095852565
0.7556384134285054	0.2454011139001059	0.2676007619304474
0.0006506516058284	0.3922653465020675	0.1539981899052869
0.0051601405318944	0.1899644623690422	0.1213751743517423
0.5029277237223728	0.8936315666830559	0.1126343652089402
0.4979780035493112	0.6900225487290332	0.1542577666012320
0.0024296012276724	0.6042002754911616	0.4037543491420510
0.9967159979964714	0.8057896116481064	0.3734108145738232
0.4997128288012286	0.1024045846955838	0.3639523711077077
0.5052499375136034	0.3046402295608433	0.4004961447151269
0.0913572728158077	0.2966534843662886	0.0967340054644601
0.5916353488934181	0.7923248787421434	0.1549065687597790
0.9102599849106185	0.6999640676290239	0.3481415465001597
0.4117900038026239	0.2021756613057774	0.4147861791887867
0.9127609099328791	0.2919711587056937	0.1032567350259571
0.4127535489533459	0.7981258211335840	0.1715028740692860
0.0903440069967093	0.7044086301350952	0.3538755922790420
0.5914189831030774	0.1970404881762912	0.4206527188287766
0.1765309114498987	0.2068526405138689	0.3915044489026072
0.1584975260805174	0.4258126549771782	0.3860876977581088
0.6855615703313037	0.6569124940385686	0.3840701289441180
0.6830320585263093	0.8990423813076847	0.3847353428659691
0.8306405836193177	0.7901653551691833	0.1388191304275679
0.8486218507839547	0.5600192552019606	0.1292600921186187
0.3289549274738323	0.3286135374389176	0.1409217178174375
0.3232141102774533	0.0941950052323219	0.1386594948687821
0.8292595753715798	0.1678997472242311	0.3790212040066747
0.8258201666994245	0.4015032258365018	0.3818782763622425
0.3265748955576190	0.7058967690104577	0.3802204029447141
0.3432384436792489	0.9285588312485560	0.3856184368692401
0.1724578265839957	0.8266116058443413	0.1319475523541603
0.1802550927875901	0.5921824167047254	0.1339972225646473
0.6767969996778432	0.2904679196121654	0.1289656628575873
0.6597726969534848	0.0651614382668908	0.1329204563375249
0.2132001722225692	0.4927778285713587	0.2445160546118804
0.7124239318574556	0.0004411958388507	0.2763840124842920
0.7915478056870002	0.5007499333446980	0.9844499993618698
0.2876429431742735	0.9929593911280779	0.0330470501306000
0.7820152572239868	0.4908104122568570	0.2586588830064696

0.2792439126441751	0.0034341085329756	0.2604044745527215
0.2260465535118150	0.5032094008217619	0.0121221321611912
0.7281455423954322	0.9908982943529768	0.0092046226647492
0.1343654664910700	0.3806039086509193	0.7241957686395175
0.1169333924817662	0.2103461252190623	0.7322441533928604
0.2524920593993193	0.1157820491869217	0.9832995314873256
0.6135076265696044	0.9183541085725361	0.7267797606282085
0.6420547948976286	0.6750989303056562	0.7196967014937868
0.7345393540679461	0.6131296241446018	0.0420175590005171
0.8679186247617400	0.6134322477869789	0.9679255942710311
0.8798234291337892	0.7778841064590296	0.9737139229425376
0.7529496335668171	0.8769595685465539	0.7352049337521612
0.3917215541856665	0.0784423458835787	0.9812244952761537
0.3753360284999237	0.3184132294665090	0.9745027648354958
0.2708336332615451	0.3785804195455055	0.7885611436536506
0.8923523610611620	0.4134943640427796	0.5408441182236472
0.8739637013339823	0.1730189811803961	0.5463492205508953
0.7689787246507570	0.1182962870200746	0.7285795877026802
0.3676919870445101	0.8831317417869854	0.5491668014054828
0.3848152796119971	0.7115642402079315	0.5411131030852891
0.2497570023216204	0.6145441419354669	0.7902713985499237
0.1106770145050289	0.5779632376161611	0.7924727656451502
0.1261523253038106	0.8217582191533572	0.7981553206796095
0.2323749665049331	0.8783281333322767	0.4805381258578359
0.6325462585630672	0.1076223509217230	0.7924722142067193
0.6162382475657739	0.2825646665996880	0.7877243796429325
0.7506981924534530	0.3809425606294423	0.5343349945570939
0.8975807327399353	0.5673217763313676	0.8015283273088839
0.8717170112210815	0.8181776258235761	0.8003032197490521
0.7746342354546779	0.8711282557432045	0.5189097126829864
0.3640248029781021	0.1009281180768686	0.8059354888715009
0.3872062666073364	0.2994680193564957	0.7978304126957485
0.2465801827040478	0.3777462550487457	0.5135297314944831
0.1125697652406049	0.4222163667468231	0.5547180773205979
0.1405716059780526	0.1767682544696098	0.5604323039633936
0.2228226713989635	0.1225993710467612	0.7635215800964689
0.6325203602799827	0.9047642114081534	0.5500195717913812
0.6128506730550113	0.6873886398660832	0.5325018011282571
0.7682332425860825	0.6209359538376052	0.7685719838520665
0.1417606344450281	0.6069263088359997	0.9653341911044833
0.1194360598214567	0.7949771826474024	0.9743414524340793
0.2578473260532100	0.8714616557040049	0.7644663584021887
0.6150476483138954	0.0674206084039700	0.9638307191062077
0.6398686965938968	0.3150345479704186	0.9596896328587723
0.7207938981753614	0.3732069244421908	0.7575390120127506
0.8625979299200790	0.3832524715575781	0.7131776869862632
0.8863151795130397	0.1963680589234029	0.7223862747625687
0.7434773777929961	0.1217088527014430	0.0086246019197600
0.3872193250456932	0.9241201588949690	0.7186700366785812
0.3580366296318569	0.6769867294181041	0.7100906931597010
0.2828900718006445	0.6222267007644803	0.0118524464528127
0.2564816194009887	0.2496429088796359	0.7501788306456784
0.7448847644652571	0.7434033511487542	0.4941799669833928
0.7655174882019944	0.2535283062744066	0.4909233147781149
0.2342192880189350	0.7418731310972025	0.7460515628800558
0.7487542140144701	0.7506193562250246	0.7938765386564481
0.2624009298690891	0.2462466184753736	0.5264798634923475
0.2445275083635975	0.7492273640221251	0.5174788318182296
0.7562255947805718	0.2468599696290840	0.7680794341438462
-0.0003066521225236	0.3917790218848577	0.6582358430888362
0.0050723919580888	0.1891194147487151	0.6214151934716657

0.5010191117171009	0.8948355281736798	0.6179079364587990
0.4951318851211004	0.6905393047904450	0.6554891008967705
0.0048549502405981	0.6022992896267169	0.9111428133170258
-0.0008818754906965	0.8014240415546373	0.8785142179411808
0.4998469452552725	0.1007044375924710	0.8648675428746468
0.5051036004081103	0.2973990678232740	0.9008612788173261
0.0903556836141414	0.2964022607802264	0.5989240993945444
0.6004919080046925	0.7988396973879947	0.6511696807900098
0.9161470328191974	0.6953442034945395	0.8414829101658442
0.4075413108368847	0.1985543254489589	0.9123544251722299
0.9139073503133790	0.2919866918852591	0.6020221206919018
0.4075352898332565	0.7978841316058612	0.6745193194051566
0.0930286854276809	0.7002239747426512	0.8538959321617261
0.5952092944153096	0.1933794008720414	0.9193699947178025
0.1793743164441268	0.2077204129599120	0.8918007329977782
0.1617139442004350	0.4306880601931275	0.8844342534611024
0.6768770603116568	0.6636882925424283	0.8920410624832371
0.6784703624046230	0.9030049945371733	0.8864143750942117
0.8188591888481814	0.7821263387894353	0.6406417017546314
0.8463452467632530	0.5649075554002726	0.6345747926251946
0.3317544276011478	0.3274017078017712	0.6401426178642514
0.3222936607777787	0.0930973730171976	0.6380211651278548
0.8287235490946609	0.1681969207065201	0.8790148812075086
0.8237746965097552	0.3977367731451791	0.8808686475848729
0.3236335061252285	0.7064114658174003	0.8803289911884223
0.3402836540282104	0.9324452266603072	0.8864462729881039
0.1740798872657713	0.8280668925324620	0.6314650849890606
0.1787155439761770	0.5949038410408491	0.6340567050199645
0.6777750859995265	0.2891680908103384	0.6276600309196466
0.6640680304678050	0.0605215476305465	0.6320503036877494
0.2129076313074726	0.4924613142773488	0.7370540241548414
0.7196101714008820	0.0008590679260624	0.7787138604118010
0.7915517105263421	0.5021464850841721	0.4893608982385269
0.2883441869811528	0.9939121016737024	0.5273111536718780
0.7837671407806337	0.4901722807851910	0.7594930551556305
0.2741109755848881	0.0019607958624696	0.7572858821486537
0.2181698243196454	0.5061589751377226	0.5077654996657576
0.7302732999107204	0.9939618409731560	0.4977717987845389
0.5853626929569038	0.4778613746874421	0.7763316166709761
0.6013859925934911	0.5232099492921252	0.7075030172591087
0.5336307197208942	0.4277982226228823	0.7585128870064126
0.5651044405111512	0.5207319855671118	0.6246342724232878
0.4993365962988754	0.4232141977036583	0.6747549235116618
0.5138738433225735	0.4703674067126027	0.6073668225129534
0.4662801637631811	0.4685755242889584	0.5228968712688177
0.5007071525207104	0.4978905111829280	0.4407086113266425
0.4644663119505105	0.5423964895226177	0.3843847958096673
0.5758246203415004	0.4769893605579222	0.4158309744626820
0.4957667436996602	0.5618342910588853	0.3022214621371191
0.6041307999663853	0.4964906933084568	0.3271196006445366
0.5644238037986287	0.5375033827990610	0.2718718382918527
0.6199279266776155	0.4848103332859814	0.8681163691077654
0.3894051738706890	0.4953728089687057	0.5439613416855551
0.5697825648603475	0.5225240522351212	0.9340170856373388
0.5964711912327680	0.5272572032399553	1.0002921788565993
0.5579528750935938	0.5728800033126702	0.9088000887995008
0.5172032981573925	0.4961409064454760	0.9429255854071767
0.3664529448111310	0.4677439068193410	0.6012167123591992
0.3917178472079425	0.5483134813721072	0.5625330735301491
0.3518878378353607	0.4895013626663287	0.4866739730899659
0.6724153118085134	0.5115895704137036	0.8618973335066671

```
  0.6321110944144965  0.4350977333226468  0.8956862414565795
  0.6406822451006506  0.5630470327858593  0.7191769629272401
  0.5198777242843872  0.3918152631068068  0.8107258221556137
  0.5757089343409940  0.5603830520739692  0.5757896469171961
  0.4594390493832861  0.3835890472666719  0.6621237896998579
  0.6136256369413312  0.4998850770536482  0.4668239712989692
  0.4105054770748330  0.5615300980778104  0.4029193134210386
  0.5859196008728369  0.4237909265209019  0.4313510808808337
  0.4658212804653120  0.5969793656758740  0.2596813226435962
  0.6585576912510701  0.4792304674630108  0.3074410912976665
  0.5857634375006210  0.5531393648086869  0.2067195102544408
  0.4603596167833867  0.4150938440174875  0.5051027226280125
```

MOR-Al-T4-TS9

```
   Al      Si      O      C      H
   1.00000000000000
    18.1842269897460938   -0.0090872207656503   -0.0001607598969713
    -0.0105508984997869   20.0691013336181641    0.0015994716668501
    -0.0001797722798074    0.0011982563883066   14.8440780639648438
   Al    Si    O    C    H
     1    95   192    16    19
Direct
  0.5887365529620863  0.7150636217509292  0.6455962653597906
  0.3125332463880361  0.0677569681318061  0.0340639541032226
  0.3008488875864623  0.3127419862024994  0.0390360855301039
  0.8130037817967297  0.5740307084862248  0.0335763309449790
  0.8110465475133479  0.7975793163628331  0.0332919130821441
  0.6915421940411312  0.9229807332864620  0.2832344461447350
  0.7068960926811587  0.6778190419042526  0.2825036337761042
  0.1878852526038626  0.4176137568408417  0.2887084123527614
  0.1925393230933546  0.1936766656426043  0.2853446136387069
  0.6929116794899024  0.0736307391844898  0.2369188856376896
  0.6929322479158730  0.2991740982427999  0.2388453531394181
  0.1871983705978252  0.5691727324439165  0.2375145880545939
  0.2044953446605590  0.8134036490131238  0.2341661553319983
  0.3122885416292761  0.9191579120273428  0.4864343303139306
  0.3090710534326325  0.6952749429232372  0.4868296872717695
  0.8128368492545056  0.4257412105834327  0.4887194689236700
  0.7996239491190996  0.1799361393428597  0.4848130206131367
  0.7070069050111609  0.9147297830722712  0.4918875878795820
  0.6992203417752823  0.6847503437967657  0.4909887202498995
  0.1847665706227879  0.4297225363610591  0.4934061177153738
  0.2066849051839712  0.1836966103229626  0.4918362350112768
  0.2961948265230399  0.0778048515132438  0.2413801217758254
  0.3064239377222023  0.3120649853212810  0.2468920538843428
  0.8241688315031588  0.5630486357814087  0.2358079278505263
  0.7990343457363497  0.8098467040720129  0.2379618127701454
  0.3206480467769749  0.9297418354961894  0.2806993914246301
  0.2956079835933022  0.6828093793716713  0.2802181465726509
  0.7987256718319397  0.4130280223970196  0.2797661327081709
  0.8087890943598258  0.1821818853681660  0.2775848167255576
  0.6840005040147160  0.0617062221520675  0.0316893858503079
  0.7063192373465905  0.3085939740419192  0.0319728298960899
  0.2089563140591867  0.5777414536138928  0.0304182887502164
  0.1963839881540023  0.8102538450862343  0.0265534687491447
  0.0824208364995601  0.3711628779534505  0.1339938073343955
  0.0865739274117785  0.2162098550972890  0.1286175934580366
  0.5899562620254193  0.8690544311215602  0.1407988468210518
```

0.5857247191025667	0.7105120722893437	0.1469091713985795
0.9209319625233872	0.6222686175775544	0.3847152457790344
0.9177273849216374	0.7783866799373871	0.3799139336779017
0.4144680652710052	0.1203739476063318	0.3910017301182823
0.4169999853913440	0.2793323708271491	0.3957162670788083
0.9144526297639946	0.3686584432414660	0.1299451793328404
0.9178383112305953	0.2111580602250967	0.1254540757028987
0.4216842779654318	0.8726107970685767	0.1386948834214470
0.4170294123163412	0.7174973616375380	0.1445162944337573
0.0886899771782475	0.6243669703611137	0.3812979720229802
0.0863000147823696	0.7832346041483506	0.3762847761777955
0.5829374752061467	0.1185784379776792	0.3898311024877322
0.5866248908312502	0.2738127097209571	0.3945517030725645
0.3124633052124970	0.0684760755920365	0.5349923477732377
0.3012365258498427	0.3132693141357262	0.5392221700147043
0.8113346100701860	0.5756510601352925	0.5373994652191736
0.8130602617049203	0.7998448091251558	0.5332843058256255
0.6928529802285862	0.9259663596498322	0.7837844439761112
0.7055747433140606	0.6766761515518718	0.7891330290052905
0.1926657648016153	0.4197654461731375	0.7867471829061314
0.1911930525491746	0.1950208577046164	0.7851659336820126
0.6961843206755248	0.0729012148372508	0.7368295051809192
0.6921608472664862	0.2995005062629350	0.7387381874737146
0.1898068406119487	0.5688196375567587	0.7365495463290145
0.2025054685687605	0.8137814592121093	0.7359212632949234
0.3113661922254796	0.9188334781970796	0.9867077331523079
0.3122139288369600	0.6934645008058107	0.9882053254847465
0.8123511277217631	0.4238466319023768	0.9876875414867256
0.8001306905318604	0.1783230674421223	0.9851426960267329
0.7077675331352126	0.9130646754239004	0.9914874589341045
0.6949373520388609	0.6799783401079706	0.9977366817659056
0.1860830252934119	0.4296307676413368	0.9922075992855611
0.2073051650267274	0.1833192763664977	0.9915461987763183
0.2954047207754925	0.0789367284711487	0.7429601914627145
0.3074615563305904	0.3113082045347291	0.7464375728582315
0.8234211099888625	0.5621874029932433	0.7425585551265832
0.7962061828792697	0.8070705401354790	0.7410101190889343
0.3182012625414380	0.9306224844254214	0.7821300669872477
0.2959994679409038	0.6834908872156230	0.7810325412174810
0.7967201363985695	0.4131836304637351	0.7803875737992022
0.8089700337685490	0.1840424458474978	0.7777683043306224
0.6850440190305970	0.0621749425677912	0.5308268946410629
0.7065813691385670	0.3098465393122481	0.5315214045666823
0.2080597134076846	0.5791336217755809	0.5287781450832522
0.1942477705842864	0.8117598168514659	0.5263310821112422
0.0829444940229868	0.3726169283238257	0.6372392415699152
0.0859202571343300	0.2176785333788887	0.6294526387653011
0.5901761640455900	0.8745950934192882	0.6440592556723982
0.9233478306049088	0.6184041665753308	0.8811047890670413
0.9170891872747963	0.7722833119656037	0.8742962957345266
0.4148994395841415	0.1194984340033856	0.8917195038569216
0.4173082919448007	0.2773136161619977	0.8983254942215299
0.9146764016002175	0.3709547531681429	0.6307548170241339
0.9173255474056575	0.2136271507988818	0.6256729504086013
0.4210701990846834	0.8736502667945024	0.6397135169107604
0.4166188648124964	0.7189210145522229	0.6433803353585927
0.0901480955111167	0.6207452600184917	0.8804167295932797
0.0860109355290338	0.7791951215051328	0.8764991898215981
0.5835592864391743	0.1170732871579125	0.8876310906954687
0.5867123478100517	0.2726241499673379	0.8935583832246584
0.1289374503119880	0.3781805594444910	0.2268949413099791

0.1163068217573852	0.2079484863720538	0.2316331903407798
0.2490938936772370	0.1127575217328253	0.4852011625717736
0.6140006396886701	0.9110816945276902	0.2301812905370954
0.6313405636263186	0.6707913650229278	0.2242745116823508
0.7379053149781649	0.6208828641310533	0.5420411403562908
0.8725763175122228	0.6112355558828821	0.4746192665524963
0.8928827046889466	0.7914227777531906	0.4839654255363214
0.7549382711121245	0.8801438219316500	0.2316473423130350
0.3898461587193748	0.0804058457582006	0.4816080784574207
0.3737163255823972	0.3189557827066209	0.4752732140625132
0.2641083279435739	0.3750034119239520	0.2930056082940465
0.8886287952176642	0.4121860544808792	0.0430760184067027
0.8712376826792072	0.1703141308757954	0.0503864562706286
0.7676406339413526	0.1183949066701119	0.2324210311680149
0.3720184081653388	0.8813895345011118	0.0486700611874020
0.3888405606853282	0.7086214352563135	0.0408632132689350
0.2530644334993267	0.6112204029646303	0.2859429156678767
0.1103504318920656	0.5836438370594793	0.2898292936100352
0.1338003015061231	0.8215942408013082	0.2995712301495802
0.2376074811370931	0.8733737144156321	0.9797673242994284
0.6327076582197181	0.1109813191798441	0.2999289768541760
0.6163917654686468	0.2846367956072291	0.2921470823652946
0.7475602021430108	0.3804362748112112	0.0362654542628672
0.8939721031759510	0.5739305115401043	0.3027935867390414
0.8647200130733396	0.8186321219222864	0.3108821318538310
0.7841621691068043	0.8727279462922750	0.0079611403953484
0.3625702238244752	0.1024215334690972	0.3060893953039692
0.3842320228484814	0.3011950680163851	0.2982646774306771
0.2423350925388384	0.3685238276340984	0.0076568399069400
0.1141077715603118	0.4208115745251847	0.0564323085022369
0.1381666415696581	0.1742702231125336	0.0596652729542628
0.2209720263407550	0.1185806862415608	0.2636007967156335
0.6427998730080769	0.8852691207340287	0.0561324261052434
0.6161668914222477	0.6893246255118364	0.0481208341850829
0.7641968066592358	0.6211558041165206	0.2496511549040038
0.1426915392810031	0.6042968495624447	0.4634134665396592
0.1154057756835006	0.8037658730728907	0.4761600357182408
0.2661413245527050	0.8679132924597152	0.2610285404723505
0.6104206801612910	0.0700266300238601	0.4704905380216527
0.6359601752299049	0.3170146594272452	0.4647198151568723
0.7209256288428918	0.3745668337353372	0.2617821655927968
0.8627947359482280	0.3813755074805432	0.2170626841207903
0.8857744019252304	0.1955043935230832	0.2254612675066449
0.7387727006365807	0.1245275577308689	0.5094718866439930
0.3939620975636910	0.9225751092568292	0.2182113362626061
0.3648293600902329	0.6745851366444978	0.2120591127576270
0.2846257750070417	0.6183790700748976	0.5086435349741879
0.2562209672846054	0.2455109455446289	0.2531853692118634
0.7435003036876613	0.7471848379048928	0.0115339869223956
0.7633220637606627	0.2517527261339199	0.9975703068939622
0.2390189882903393	0.7388965153642908	0.2429928165097061
0.7425612312313887	0.7512048930809583	0.2686629149101858
0.2636718415656437	0.2393942371432613	0.0310319163370016
0.2482454462333532	0.7444589388336248	0.0208550705358252
0.7562597325932044	0.2475142285843136	0.2705701229037866
0.9978540491700024	0.3905855096162214	0.1554516557321255
0.0032321270298895	0.1879845363350238	0.1222743998000231
0.5063814712450893	0.8899321227680544	0.1141830160531794
0.4998688295634789	0.6885348017215633	0.1533464316797952
0.0055655370791186	0.6040889200906132	0.4090098962059478
0.0011618917121125	0.8052898000720177	0.3671253497076563

0.4982839633303104	0.1006754978745648	0.3653338001196262
0.5027676042407095	0.3019253624782183	0.3995139294664453
0.0893953588763829	0.2945489984859765	0.0989167946300768
0.5942234604008819	0.7900415281727088	0.1637556595981459
0.9133947892856678	0.6995172709313996	0.3532344176282310
0.4082319822002886	0.1999683370791614	0.4114688351849711
0.9112027391766399	0.2902974238102930	0.1032634045775273
0.4139209258545531	0.7957773823685638	0.1735916706457176
0.0956718345390466	0.7033980989442622	0.3610396317497042
0.5889853883885614	0.1959094462955553	0.4237086910842387
0.1765621784432299	0.2037167326378184	0.3922137585942467
0.1553521317448772	0.4257738862056586	0.3898681801978589
0.6860609602598398	0.6635291507882886	0.3860493781118347
0.6838534545293595	0.9001360025725578	0.3873673709718697
0.8333158504463004	0.7928021866649038	0.1394164198396705
0.8519192179182462	0.5638726358987440	0.1315925131457918
0.3250285117301963	0.3279100174557336	0.1422741526926899
0.3215283864186665	0.0919808015949309	0.1378813321937391
0.8280903182470447	0.1687342985359051	0.3824495233536794
0.8234143726366479	0.4035168605576147	0.3843049831198877
0.3260428342197597	0.7023263633110134	0.3794771291701629
0.3455247966040563	0.9302479415294561	0.3857504030630781
0.1781090718384938	0.8258358114901910	0.1313055546652760
0.1796679353012177	0.5902674534619160	0.1326418921611111
0.6757752950525392	0.2906742677171774	0.1320839868057740
0.6582648478004303	0.0666544129932127	0.1364433872520638
0.2064709844221431	0.4900348738554643	0.2449376797790702
0.7136337390407622	0.0014796988749104	0.2787602788038401
0.7905069256207443	0.5020451369254060	0.9905743227243405
0.2885412674233097	0.9899065345609345	0.0317333961423009
0.7861422397679098	0.4910885935866876	0.2564366218926201
0.2787565588341586	0.9992689512913824	0.2569112148692509
0.2278214453681422	0.4989636819913482	0.0168169814356169
0.7246544437150321	0.9910764003933773	0.0138029341779950
0.1311473982734885	0.3797036321226284	0.7288722380502567
0.1139238333814530	0.2079768305677344	0.7330515956583912
0.2499552468443223	0.1124576639473973	0.9833585193767043
0.6160973905240543	0.9228362110569861	0.7281141953988340
0.6390348481586239	0.6665242354977809	0.7212339194095294
0.7381456209565433	0.6173071456963639	0.0441513931462727
0.8708957681643014	0.6123091608468957	0.9690287147215372
0.8837706952151098	0.7782935398290304	0.9757997489015661
0.7545317617883183	0.8786818578797196	0.7365109198073645
0.3901973816558533	0.0771134665726450	0.9804050116612845
0.3741194013816647	0.3179254114841431	0.9769184921014269
0.2682289362340165	0.3761519489178315	0.7914086963530637
0.8896587304463280	0.4144183992750268	0.5432316221877552
0.8700347199464102	0.1728720914971634	0.5511824815974183
0.7680653271925660	0.1212259677449726	0.7303960368652882
0.3737889023771494	0.8825405132626248	0.5471718627870533
0.3834145978719284	0.7133362716978956	0.5403436905781884
0.2531951370827591	0.6120719799232296	0.7872908804448461
0.1121243762348838	0.5795266223479113	0.7893835801421795
0.1294652852978844	0.8197862526583816	0.7981861915845173
0.2385380240644492	0.8735003109819158	0.4803023817397816
0.6332074182082051	0.1084466997043340	0.7978952333330367
0.6150785095932375	0.2841814603815254	0.7904815185236738
0.7481206057837497	0.3814661583354838	0.5360126258329682
0.8985832067533759	0.5659860929170940	0.8028180834515080
0.8692894973116511	0.8158892823977028	0.8036393483860956
0.7821491070560894	0.8747486116721596	0.5159883195001547

0.3620933926098506	0.1048262663901073	0.8064718548490255
0.3833129862844863	0.2960897873390520	0.8001363173505400
0.2432246765217093	0.3706733888560833	0.5118378339534309
0.1141652323485623	0.4227824712665437	0.5599136410755645
0.1378952222886611	0.1760644124116637	0.5606423917225147
0.2202752401824445	0.1202393710251110	0.7635528791249547
0.6387302513252557	0.8939206490531312	0.5549137369717339
0.6226941624928938	0.7016868699331743	0.5365818389074217
0.7708011326222366	0.6225595494673106	0.7708577271395300
0.1449319914153198	0.6016682312060916	0.9624427690468060
0.1191502527408693	0.7975346843909233	0.9752490393096925
0.2610237530412811	0.8703951590739812	0.7651264910575094
0.6111729383323770	0.0682572601909840	0.9682590500643263
0.6370527654260238	0.3149818903979648	0.9634455110125698
0.7189159270758834	0.3751455525453997	0.7611599404265321
0.8611102781155574	0.3840949092030658	0.7159094787470415
0.8859597617480173	0.1985483295629760	0.7262056668863375
0.7401012836574514	0.1218686766726789	0.0085195089018833
0.3894255206718819	0.9238915686038311	0.7171686244915519
0.3625260894315739	0.6760695170035740	0.7101383718058638
0.2851582835423943	0.6180301807570240	0.0127553131954916
0.2527799465125084	0.2476868050514556	0.7499758876128203
0.7572169015660837	0.7466462483953415	0.4883410920933208
0.7628875963924147	0.2537054832770925	0.4936955926703298
0.2384837547460265	0.7403225668962703	0.7476531866998710
0.7435729105624077	0.7507372015113697	0.7848323543312128
0.2632532291080690	0.2405382517927433	0.5287497311031757
0.2407494495454124	0.7430940265645450	0.5148883568257352
0.7559675446252605	0.2491421777917835	0.7730176941227376
0.9980640161044601	0.3916836400958255	0.6585022457758651
0.0024689724043889	0.1899333802984881	0.6211432639329644
0.5052524777030739	0.8940633837763083	0.6192271511479506
0.4973872833496624	0.6898447320757204	0.6474010216132494
0.0072652337321815	0.6007001661253296	0.9104002048636772
0.0004903172972967	0.8009997257595221	0.8748058300781935
0.4984395008281854	0.0998406635854840	0.8643731194507116
0.5028655715047389	0.2997919808201661	0.9001297661226800
0.0897764454292490	0.2962382713291039	0.6012888564511171
0.5996411014210522	0.7987683603366590	0.6724234379833047
0.9180310852538536	0.6942624588999682	0.8429452739890754
0.4092173553864124	0.1982171601907161	0.9173017022587230
0.9109646969259997	0.2927054256459558	0.6032627953433509
0.4108138428473511	0.7974061914533335	0.6744238606803945
0.0959708212841900	0.6997268194345556	0.8585133367261109
0.5906831611367382	0.1943049692800149	0.9212654148314660
0.1775742870808948	0.2052853214187572	0.8925724657111660
0.1599948245101641	0.4308719312303284	0.8875692305873836
0.6785469709368808	0.6654470880899994	0.8931279050311944
0.6802654942446099	0.9041507399691070	0.8881357133815317
0.8228055188181633	0.7858751221197735	0.6402632804071828
0.8465229334571970	0.5653862234576195	0.6368854995320444
0.3290134108733379	0.3254676478537853	0.6419382287163367
0.3210794561290385	0.0914715264983016	0.6392119776572159
0.8280568836844359	0.1689670503632589	0.8823063135293291
0.8226473543193908	0.4001244419944387	0.8839085661801986
0.3272965181359051	0.7014201485683282	0.8810218423686289
0.3455367558222918	0.9298289722252319	0.8867186732842947
0.1787348274785786	0.8257129612853664	0.6319151786162451
0.1813698206727640	0.5911348504358356	0.6319634074893351
0.6772118430048145	0.2901899982095147	0.6315223427746078
0.6622566909582555	0.0627341423758137	0.6365350903797303

```
  0.2125186549379118  0.4903638520073562  0.7394417878931245
  0.7222713819531815  0.0030502813329344  0.7816212879729655
  0.7895372256446347  0.5035119596781682  0.4939435923482309
  0.2893233900426083  0.9905237490765028  0.5304978302184967
  0.7819372667816069  0.4914605601538846  0.7616140695118594
  0.2769655180701335  0.0011231445974486  0.7616966345946498
  0.2258487759575815  0.5006519726108801  0.5098660631001790
  0.7266598671647335  0.9935982541442742  0.5045288473445971
  0.5691170057451684  0.5414908900978519  0.3980254411920832
  0.5031054607989202  0.5293795350210255  0.3378523953900858
  0.5093712469210164  0.5134926561819743  0.2471977819540668
  0.4318950630438907  0.5253317341415275  0.3798280751968347
  0.4475417913435978  0.4932340613238187  0.1978170430629251
  0.3700959963832661  0.5047409750706884  0.3283997733796261
  0.3784867411616267  0.4872655294536978  0.2384752986515942
  0.6405201423166341  0.5137849091129978  0.3583307231127513
  0.5545596319808869  0.5110870568967405  0.4899079847924197
  0.5696439154257106  0.5450772137021703  0.5688067786997948
  0.5169955141082315  0.4485795713331145  0.4955459481787101
  0.5496278646359570  0.5186239509220176  0.6525323587875536
  0.4978391265647911  0.4222229323781924  0.5810671487145651
  0.5128662160466616  0.4571587161881311  0.6603622224831567
  0.4915129532767898  0.4286790608208027  0.7508603080464501
  0.5367233314336884  0.4546174592680953  0.8304493345839546
  0.5628258545095595  0.5154351193596645  0.2142527578847643
  0.4207232558990291  0.5561563326814336  0.4395478658049516
  0.4537935439737939  0.4807260960128220  0.1266705372120160
  0.3162330202641635  0.5042441045261368  0.3602839871175788
  0.3315138289965345  0.4707109037745719  0.1991336339435195
  0.5756003198444103  0.5957141388511705  0.4074221167704251
  0.5963574619372074  0.5934390646471550  0.5654532886850172
  0.5200331931289831  0.4132752196560474  0.4393467341282151
  0.5626625379068491  0.5478365922793765  0.7124050145711213
  0.4722259213644154  0.3730820287944878  0.5844381340010233
  0.4567329754669617  0.4784454602186909  0.4430902879818572
  0.6358455368215893  0.4599309999595463  0.3459646280448044
  0.6860103242658727  0.5226473634049780  0.4050250812008638
  0.6542300461638744  0.5382276033469655  0.2944868066810923
  0.4957653659595336  0.3740947485989027  0.7471187038294860
  0.4326723408969474  0.4392020856978537  0.7625542523476587
  0.5209927517484588  0.4282667472383486  0.8923324419445335
  0.5280899733147182  0.5081935662111305  0.8416107593259363
  0.5958484145292121  0.4465136513953709  0.8190543136737932
```

MOR-Al-T4-I3^{+}

```
   Al     Si     O     C     H
   1.00000000000000
    18.1842269897460938   -0.0090872207656503   -0.0001607598969713
    -0.0105508984997869   20.0691013336181641    0.0015994716668501
    -0.0001797722798074    0.0011982563883066   14.8440780639648438
   Al   Si   O    C    H
     1    95   192    16    19
Direct
  0.5884885004241928  0.7151203456173563  0.6377175687572786
  0.3160634017199188  0.0720971222171636  0.0347050147685011
  0.3040052098618257  0.3172975141613733  0.0372312309310604
  0.8140288392383818  0.5736511444027882  0.0273650219465126
  0.8104774507539914  0.7977695361664956  0.0319007646291735
  0.6912513776880357  0.9246832432798597  0.2793990045199203
```

0.7076235573163832	0.6809639576812314	0.2801542459730502
0.1952912880963239	0.4214342496892218	0.2846869372240239
0.1953312140026048	0.1983925344660678	0.2837583558630399
0.6940054288435578	0.0744513970276799	0.2308345363281339
0.6959636120469482	0.3006790800479959	0.2330977871892920
0.1925552993950086	0.5723554978191160	0.2376636676594557
0.2042687973049589	0.8178501156954452	0.2335681096928099
0.3108741420553723	0.9230070433503093	0.4860394047469702
0.3105378251160099	0.7003429474893372	0.4855406357232420
0.8171391059310962	0.4269483357775563	0.4833137105923893
0.8047604752335829	0.1804566415351650	0.4800330856389527
0.7076703276626121	0.9166532170657247	0.4872256658216299
0.6965975850971764	0.6800557801722791	0.4887432249925955
0.1865633040120691	0.4343531154547546	0.4892238728992833
0.2100705982160340	0.1885293419014651	0.4900759846921225
0.2980702809641340	0.0822190536857464	0.2420247634320946
0.3119090209519141	0.3148821949555917	0.2447930103673162
0.8200466174291916	0.5657343967829341	0.2300763113792391
0.7994239697452423	0.8124854545032311	0.2363745216517838
0.3210772955319590	0.9336185572408512	0.2809315415637769
0.2974927591777405	0.6882073250409010	0.2794548861141830
0.7995303669577880	0.4179327183270570	0.2748133924810908
0.8103756999891872	0.1825571816810448	0.2721737519896588
0.6903612179526276	0.0637969134330522	0.0254881291279114
0.7113388403629534	0.3109604656228633	0.0260384543313966
0.2103035226164771	0.5829006240972916	0.0293097917229127
0.1962251934713997	0.8151241664312764	0.0262465299742473
0.0869657075317217	0.3756785075076541	0.1308712844131605
0.0902515363596817	0.2211432337848847	0.1266564370056701
0.5893228543047492	0.8754322193695916	0.1346719541460177
0.5865784364307191	0.7153554783605026	0.1420187982149902
0.9186606399676401	0.6255631541771576	0.3778294599761753
0.9166821892581406	0.7807257396593253	0.3760653208486439
0.4174102331605990	0.1245041614773799	0.3909359714247848
0.4214041589278331	0.2826983421485571	0.3949745186368626
0.9186876855829825	0.3738488486857240	0.1265371525914676
0.9212403495622137	0.2149282990138812	0.1222173850429905
0.4213289839617588	0.8773548047477777	0.1370114217496167
0.4178372004038591	0.7226524989499987	0.1416099037749626
0.0872964846881492	0.6272917765616032	0.3775954851017176
0.0858363609799049	0.7852408144239678	0.3740216652106307
0.5860346644623671	0.1213104656907069	0.3825778750697013
0.5906229700828372	0.2764139653540376	0.3888391474370422
0.3166340962075423	0.0728921864488679	0.5341038227959786
0.3045606063480281	0.3193555458872264	0.5365318887051955
0.8131255489180441	0.5770900617672761	0.5337847132769004
0.8099723730705587	0.7982572622463157	0.5317188349816184
0.6922683306640274	0.9268712212913238	0.7786364500972401
0.7085179651632406	0.6807018499691235	0.7866168218590535
0.1950537269873827	0.4227154295298686	0.7832589895059158
0.1957338623750381	0.1978828868938805	0.7842446585166776
0.6962276812921949	0.0744241535539087	0.7294450397933616
0.6941505162986753	0.3014094083909011	0.7331832013890084
0.1902464675867114	0.5722818551110966	0.7364353437271859
0.2021430189108290	0.8172320497753672	0.7334086865057534
0.3107701613402961	0.9230996488338737	0.9857828844502604
0.3126719905889827	0.6993945706674556	0.9860117055611611
0.8187120401873527	0.4241994089720761	0.9797661315144126
0.8051577433529346	0.1811637553003088	0.9795611329110192
0.7088676849899134	0.9168121830108377	0.9861482071912446
0.6952637205715345	0.6789369527460665	0.9943721083690740

0.1888968710959492	0.4341752387025081	0.9889865653049554
0.2104866658461422	0.1873567354912895	0.9906521967733515
0.2985956677638453	0.0808634332713210	0.7414726940497339
0.3114026534033824	0.3148550121696435	0.7441367745144115
0.8263044334310420	0.5647088301663881	0.7386485409020013
0.7992951745003740	0.8102307465058706	0.7387162571812257
0.3192735146008343	0.9323904626441278	0.7805427339180580
0.2954177332061562	0.6876209451682912	0.7791421587450239
0.8010620742351758	0.4136324402843699	0.7735433379568066
0.8095143406169873	0.1847972781559795	0.7718492611090479
0.6889990561231343	0.0635193739704800	0.5239201828389468
0.7118612134775526	0.3106833305118054	0.5264757708906656
0.2034060505842917	0.5858346810240810	0.5277665066103020
0.1943981958283382	0.8170336036076089	0.5251292985761636
0.0858434689068010	0.3744311353368268	0.6329146353247188
0.0896907233729667	0.2193769114930615	0.6281422381749726
0.5887737113114122	0.8773374463990787	0.6360715339564774
0.9240428941867196	0.6229582826958160	0.8793669437756574
0.9179908483957201	0.7767145676977871	0.8728087492683719
0.4179403279443450	0.1221063249756700	0.8906637197735826
0.4203579693775164	0.2810475424768515	0.8953514025252791
0.9187867319479439	0.3713552243327112	0.6238151981445004
0.9213304541540867	0.2142143871696952	0.6206329210655830
0.4211561712447030	0.8747915753399446	0.6372605732133213
0.4187050977892479	0.7185556537483875	0.6421158273363133
0.0908794044663170	0.6247983053960467	0.8806562941219591
0.0869311410212033	0.7825901881635695	0.8755316209289166
0.5872966590969736	0.1200733718127079	0.8818575026966731
0.5901562882932039	0.2758883081769413	0.8889553156549806
0.1361216314374123	0.3829303955597652	0.2215681005351878
0.1194256438102312	0.2139212306861036	0.2300033240026673
0.2552122161761545	0.1189958288673778	0.4836538448563719
0.6131404978879158	0.9132060522110609	0.2275236767739078
0.6307355671299755	0.6808579180560023	0.2244444675908438
0.7368420004868793	0.6180118364899974	0.5423076354074220
0.8709504732329131	0.6158416440194694	0.4690576942123842
0.8882502733437763	0.7890934305834628	0.4793212441222142
0.7542058285481249	0.8820596196273687	0.2265748273027257
0.3945029064698535	0.0830877135695365	0.4811608684748654
0.3770294776773792	0.3236372644028966	0.4726030450350308
0.2710717803160652	0.3785063227643429	0.2907064945749263
0.8952339031773804	0.4132425450464636	0.0348007797904126
0.8780807006040612	0.1761762686869378	0.0420743534229007
0.7688313666624720	0.1189868087723759	0.2273076062802833
0.3705538248402940	0.8844974525904292	0.0476726542493264
0.3897891690727815	0.7142197541649666	0.0378545790543539
0.2564521786019238	0.6162692898302982	0.2866632542786655
0.1153917124727701	0.5840559323970556	0.2911149964990697
0.1331259089031713	0.8253583589383335	0.2986590249766279
0.2356977326577494	0.8793028414439513	0.9795316667769179
0.6322185540710166	0.1132770979769343	0.2897702423972406
0.6187555264987658	0.2872872400836660	0.2855675090516982
0.7532051421644093	0.3825902598454853	0.0301409561072980
0.8894722499968097	0.5772013017090302	0.2973683197008756
0.8663605348811945	0.8239862841098360	0.3071455186121971
0.7811442052370500	0.8720008551875862	0.0067792267265229
0.3635127494981737	0.1079673434825026	0.3073282007374709
0.3892939607822204	0.3019171740401184	0.2962164444377037
0.2464641002533245	0.3744186934738352	0.0075949768171054
0.1164694129395053	0.4255713904626620	0.0524183107912520
0.1414243233714246	0.1779750338391637	0.0586658173271350

0.2223811843782164	0.1229607888340098	0.2615123842645081
0.6408905367950959	0.8976438739474799	0.0515180892322793
0.6171667319965454	0.6879456193132605	0.0460328397561295
0.7622254639902851	0.6263617827360154	0.2354593627940665
0.1359651758994598	0.6122633789038631	0.4666160009232364
0.1171221140903360	0.8025012227416832	0.4740858223796716
0.2652250412623961	0.8730149952057509	0.2601549903008782
0.6160179310539710	0.0719168725815961	0.4609374121632026
0.6429179255754501	0.3180854262708921	0.4575849855420183
0.7245797728292428	0.3757488555284638	0.2554492648951326
0.8655265425468119	0.3931398066678698	0.2098211037565151
0.8872220989408242	0.1949082189706856	0.2196418008500117
0.7437593942998957	0.1257444578612652	0.5067280873090939
0.3937098340416513	0.9267615981697404	0.2172074334535213
0.3658931807570249	0.6803837143910632	0.2099487285250957
0.2789573781171038	0.6256758221973848	0.5056167867074285
0.2598734598017203	0.2493579381685191	0.2520348576954513
0.7454726492116477	0.7446713450287515	0.0106827020574063
0.7677843340927579	0.2545014208431677	0.9898022027706502
0.2398612933043647	0.7437617661796067	0.2425807145785018
0.7449081512039303	0.7544850898089610	0.2735468473374184
0.2657275968223620	0.2444341352593366	0.0290914935402724
0.2498307814730184	0.7506280497178940	0.0207817575244490
0.7583670137469946	0.2483208531868940	0.2659207469365223
0.0022109212599846	0.3943416054610123	0.1536397628881728
0.0069331512008884	0.1930770837833211	0.1205198037768409
0.5052649877986303	0.8962692151496332	0.1109302060769802
0.5007298455616199	0.6938554378741613	0.1498239379636990
0.0032961929850603	0.6059497379318304	0.3995686135633765
1.0005666705279719	0.8078768453891579	0.3690009378398967
0.5004323124374365	0.1043638337615081	0.3621814252531456
0.5073211401889602	0.3042879525599885	0.3989688836245506
0.0934032884645053	0.2990918056898102	0.0953447801673720
0.5958605272310858	0.7955343715130609	0.1488048532624881
0.9125158515837252	0.7026497816817192	0.3450068977337172
0.4119427409431016	0.2036111578234349	0.4142178516548686
0.9126930514846023	0.2946127292954167	0.1064239410833851
0.4154997741926120	0.8006248251557375	0.1720882838497747
0.0934178870330456	0.7058943912001504	0.3532581691861825
0.5943691793866375	0.1981824005439344	0.4168901110370267
0.1788787741826862	0.2076502034490538	0.3907090959428817
0.1602502667801827	0.4275947000327917	0.3850673383026983
0.6919580591722210	0.6594114880653846	0.3826062160401552
0.6846576070094563	0.9009662487451079	0.3831437514586349
0.8325646606500156	0.7930835698032476	0.1379877373663497
0.8496324450405519	0.5609776007522530	0.1265831048996101
0.3303809218797631	0.3299652642982147	0.1400177769998212
0.3250018545162508	0.0952642395081362	0.1388083765658437
0.8304366882098866	0.1689827354725097	0.3767830041566673
0.8258412183679446	0.4058775816935753	0.3783119471401475
0.3279680454711159	0.7087232714835157	0.3784689196267255
0.3462555047803203	0.9319129834970703	0.3859651591457429
0.1773914547801947	0.8302894701991003	0.1308700908461530
0.1836911388532511	0.5939697830764630	0.1329264301728097
0.6806513216804274	0.2922325336423647	0.1257209001447294
0.6621207595665848	0.0646707034093504	0.1294442667152038
0.2149253840808868	0.4940212814923769	0.2429463382741518
0.7134552236816296	1.0031961188384935	0.2762659878930257
0.7970519361143227	0.5025127880525058	0.9788546074889646
0.2908768542227350	0.9946367923474718	0.0311502415533280
0.7797574345451158	0.4957243908624379	0.2567256874392326

0.2802970515030310	0.0040048787562518	0.2593129902505847
0.2287640827707935	0.5047465353644991	0.0107612679212050
0.7321664798088159	0.9943248344115304	0.0030667326533888
0.1344854690014076	0.3827105097332365	0.7239355202418850
0.1189903671939936	0.2113857101644557	0.7313874715892467
0.2540454949743859	0.1170117077553741	0.9835049240527255
0.6150645967409046	0.9210142942022383	0.7244489564088433
0.6432622047587481	0.6742210479404818	0.7165016480255679
0.7365747923181133	0.6133784200880658	0.0354652387148438
0.8714035750843536	0.6165353176551530	0.9672718599798646
0.8838485311900349	0.7817526579636069	0.9739003238823354
0.7548172844174289	0.8802608108895594	0.7316504098541909
0.3940198825695553	0.0820266729110255	0.9816405668070820
0.3764295159181836	0.3212447883058136	0.9732616008699843
0.2709848470582694	0.3795598402368926	0.7877409965727518
0.8947906103955986	0.4146436219892863	0.5357631506435092
0.8767611340451491	0.1729946813648804	0.5439687763520246
0.7689554179730267	0.1218697669312161	0.7241976967587934
0.3687833457482772	0.8831207925260711	0.5487218162506371
0.3868158929562675	0.7113527203706315	0.5389244039768296
0.2533490209000592	0.6159472102833978	0.7872505841005379
0.1133865694973346	0.5814117232543146	0.7916458421025341
0.1303182886391158	0.8235110986250563	0.7976327276373949
0.2340574776690895	0.8814373669124195	0.4790170985980273
0.6331372556522001	0.1113743639741727	0.7890555289774315
0.6162146084352692	0.2891949908130851	0.7851215557410157
0.7532263081225716	0.3823593348059737	0.5320513217391372
0.9001246105431316	0.5705592956473824	0.8006472947764861
0.8719831182093122	0.8214915148674214	0.8013684826498707
0.7796834487065002	0.8733479386266230	0.5139835390122812
0.3652265535379495	0.1040686818134369	0.8067172172179057
0.3886579307670467	0.3025664286732835	0.7967608866071577
0.2493808323111649	0.3797317112225371	0.5120612065219737
0.1153622632277383	0.4235486523926440	0.5537898220625973
0.1419817373735423	0.1780066038102883	0.5593602652029016
0.2241501303166586	0.1228264227782351	0.7631218932168218
0.6372164866813376	0.9013039875059665	0.5493841117846566
0.6173357593875812	0.6929535539713452	0.5296775289602426
0.7694766928141644	0.6221876670402131	0.7676671280708334
0.1443320178517386	0.6082369722358142	0.9647895710068544
0.1196462105502675	0.8002898524008758	0.9745869254667288
0.2619447872822362	0.8723902660164637	0.7634448056624172
0.6185446184380822	0.0717273910522428	0.9606561130008555
0.6418658480140428	0.3182580433368958	0.9574715801090385
0.7224621587193648	0.3767605593107198	0.7549495532951543
0.8635635845903384	0.3837503450105064	0.7075153873822337
0.8857513303779027	0.2005270969470364	0.7193946928392544
0.7463622778467669	0.1251670722200605	0.0092882095220515
0.3916313765681742	0.9231306126850707	0.7180486119057115
0.3626861439366980	0.6785394426331980	0.7095189747670445
0.2857195600299670	0.6239411505145142	0.0097780793106112
0.2588028463983173	0.2498780890084994	0.7502909372032176
0.7508276495753021	0.7450597905884071	0.4942309904285695
0.7691483199743133	0.2545390758336365	0.4912909641766656
0.2378715006262810	0.7432918787137736	0.7423338453504467
0.7496469236378825	0.7530206915308468	0.7864116710413845
0.2636604039466054	0.2479454878813432	0.5261068115636388
0.2478684139433628	0.7527211489731522	0.5169810686406423
0.7555784837432228	0.2494342951138583	0.7692993738280218
1.0009901661523741	0.3932388442300943	0.6548828415413107
0.0066076645241027	0.1904147018580060	0.6217903585460289

0.5038147148910590	0.8981628387567729	0.6129188555835368
0.4983249354576945	0.6859923707262953	0.6494234091848030
0.0075891252474355	0.6054936899295461	0.9098085338892546
1.0015464406969556	0.8046548646069459	0.8746428058255161
0.5015388688983948	0.1024388698180521	0.8639429988358684
0.5063711772401872	0.3030495679032928	0.8990870305985962
0.0923550694022146	0.2976553379900166	0.5985253067727445
0.5974465833799456	0.8000066683301136	0.6557530099765845
0.9180400167398706	0.6986984117860984	0.8407296687006007
0.4115359701912371	0.2016713016136674	0.9111801819942252
0.9166238619631810	0.2931087835550352	0.5965124128379420
0.4163141109530070	0.7976307050234362	0.6701526537419547
0.0966692777641894	0.7033287453142666	0.8556339584631448
0.5952180209404135	0.1974720195075265	0.9148317507035080
0.1807243021012743	0.2082649067070047	0.8912209191331489
0.1626476264514729	0.4323757334856441	0.8842726407954898
0.6781212751260443	0.6679738281037002	0.8888540174481269
0.6813004367458259	0.9054403891693397	0.8833229642581392
0.8250924874897574	0.7870968025709292	0.6386479963071146
0.8500990304244743	0.5692046663253798	0.6331981728791873
0.3329151006838736	0.3291227648566946	0.6396805408072752
0.3251340266723483	0.0954644187894495	0.6385345016261176
0.8296416092276703	0.1689267767889861	0.8759352760499474
0.8277793651358625	0.3991225733787859	0.8763910642548071
0.3260540528850740	0.7081778396786760	0.8785083037978901
0.3448552149983765	0.9333284411339575	0.8856538552676830
0.1766330511135605	0.8308673949711922	0.6304144968163076
0.1801716077278137	0.5959888559736625	0.6326891855489076
0.6799823149538161	0.2917983611663000	0.6258519814206422
0.6634034937344709	0.0636103738860884	0.6288055265294241
0.2142940842572406	0.4940475310041234	0.7370483526345210
0.7201418514135032	0.0043567116053692	0.7754058302036555
0.7939417576727889	0.5044415058092500	0.4908229043559134
0.2916759767960618	0.9954763419871272	0.5293340002205954
0.7874802463634787	0.4923514438106530	0.7557641096976182
0.2791333615724463	0.0027859039633017	0.7564397685533363
0.2204087632344849	0.5079231494185958	0.5062960130108524
0.7311467545893925	0.9949232391347564	0.4982408983891161
0.5926018209332126	0.4864445035750849	0.7889433386956541
0.6100977099361470	0.5266697868366359	0.7170393694796010
0.5344534783777036	0.4402834075971827	0.7762570739078317
0.5667359050145755	0.5267837373094606	0.6340990179893943
0.4949585685300231	0.4338755752803954	0.6958644660540275
0.5087104103997442	0.4764398485621870	0.6241332817563344
0.4602687405065665	0.4755643158341398	0.5426873743740703
0.4966223602643074	0.5013767743858958	0.4575123664015059
0.4729428040161483	0.5586110103609818	0.4121112585366984
0.5561877689762941	0.4653053339490705	0.4224499318561414
0.5073833773972648	0.5786813941635138	0.3322877275533890
0.5904517616255449	0.4850314780241957	0.3427733705337193
0.5657149893097247	0.5417358762790809	0.2972197142962706
0.6318140334350807	0.4901564039175656	0.8779539963639859
0.3893099150670546	0.5129973992941874	0.5688621652151747
0.5830034592328972	0.5209030239027957	0.9513933070024845
0.6126379204294043	0.5221328380373591	1.0158694750256414
0.5675362662844859	0.5721878247877992	0.9337083646411253
0.5323043286447593	0.4917157004632464	0.9609193061098563
0.3618430460384392	0.4883763353214741	0.6257312658822888
0.4013543481995958	0.5647969955925279	0.5877916810483889
0.3519503778334832	0.5133945786728765	0.5110976646133696
0.6821825438086827	0.5197142197817667	0.8701396897304940

```
  0.6482112600975676  0.4395890916351303  0.8985034133093300
  0.6556468705431866  0.5615592375544706  0.7206389688818714
  0.5202959525333983  0.4072137111196011  0.8320719227446105
  0.5380588018158706  0.5767169811747532  0.6318227602349403
  0.4523114857736477  0.3958113859385152  0.6900669896595344
  0.6009377842735877  0.5318390103697161  0.5727917846928491
  0.4275239274117802  0.5880329692653119  0.4387202671587312
  0.5751520720929236  0.4208193101571150  0.4576366103565176
  0.4885245519612529  0.6232726547475941  0.2973995524385818
  0.6359470912723892  0.4559978618832551  0.3156557692046646
  0.5922702264779207  0.5574282557447472  0.2349160480370449
  0.4446673181632120  0.4230675700212982  0.5311311678706271
```

MOR-Al-T4-TS10

```
   Al     Si     O     C     H
   1.00000000000000
    18.1842269897460938   -0.0090872207656503   -0.0001607598969713
    -0.0105508984997869   20.0691013336181641    0.0015994716668501
    -0.0001797722798074    0.0011982563883066   14.8440780639648438
   Al   Si   O    C    H
     1    95   192    16    19
Direct
  0.5876279417698579  0.7100904109906656  0.6449455417873323
  0.3138279702678240  0.0693817777042558  0.0375375104355171
  0.3024046126793712  0.3135770818663983  0.0399898696940622
  0.8127359796603302  0.5708019929708269  0.0355934953776311
  0.8088532154625308  0.7948516759490281  0.0367133749926893
  0.6900961527490166  0.9220784073556392  0.2852518568458303
  0.7053871105251168  0.6771137967209809  0.2860822396086535
  0.1944498936161963  0.4205642689313110  0.2872086860475545
  0.1922632088293141  0.1959492913767506  0.2869217284675308
  0.6924861193124435  0.0721920416857779  0.2368801655616043
  0.6933887047560856  0.2980313114649250  0.2383581834621041
  0.1897259752713761  0.5706979923239234  0.2392678462266636
  0.2009579607910249  0.8166503301831238  0.2379857567241723
  0.3110824146736337  0.9181574645942073  0.4888736301767643
  0.3089340344746689  0.6957718232674032  0.4908866621617831
  0.8163345538880988  0.4235209594081821  0.4893699686644155
  0.8014949452546142  0.1783441357018021  0.4857665236836935
  0.7057737370394561  0.9143630600400041  0.4934849589288025
  0.6963717160218740  0.6773283663256261  0.4943851184065387
  0.1853566274997318  0.4306399666485636  0.4930301525499621
  0.2083323900815190  0.1841496079241461  0.4927551945334687
  0.2976010955672619  0.0815601242304298  0.2451457817759899
  0.3095989829923499  0.3120769426856311  0.2477665168116002
  0.8192088383049111  0.5622557371553091  0.2384404720913409
  0.7967809100319938  0.8083131300017575  0.2421308534532110
  0.3194802610967653  0.9316520533461577  0.2839971986012856
  0.2946334679523895  0.6868897104048293  0.2842070247173397
  0.7968918641353864  0.4148292583757331  0.2811086288854423
  0.8083080448587828  0.1801912918265695  0.2776857841161207
  0.6859114576758240  0.0609648415889052  0.0319216408466687
  0.7076176763929407  0.3084132467790194  0.0314327210139957
  0.2096018009303978  0.5797727455889399  0.0317491471713633
  0.1938421465064747  0.8119240673308240  0.0308411506315076
  0.0846537437770759  0.3737235760992079  0.1354277642402079
  0.0872216989155383  0.2186709567420586  0.1304619001025970
  0.5883221681111016  0.8699959411320318  0.1421042220034433
  0.5845647684462644  0.7104364145784968  0.1494849691834493
```

0.9193032345377430	0.6211126053279534	0.3856177093684636
0.9150995320533052	0.7760198150234912	0.3820176094765338
0.4156774853504289	0.1228417787524588	0.3947951290203944
0.4193025189834469	0.2801447364730421	0.3995157024834291
0.9159281240432079	0.3718151356683348	0.1317391228498212
0.9179471656873356	0.2128152208206025	0.1275647899826074
0.4203343672918759	0.8738391287419714	0.1409830957537406
0.4161512537412065	0.7195116579054111	0.1471618494695812
0.0881336690733590	0.6226978069775568	0.3823175910497787
0.0846637747696532	0.7814376066380689	0.3803352634608809
0.5842575305978701	0.1191829044634844	0.3897541310975048
0.5884330948189981	0.2743898661884414	0.3946980032789200
0.3132036910410143	0.0681463483764408	0.5355359201453278
0.3019934446306110	0.3143154156602531	0.5390078832140912
0.8145946901481235	0.5742380340439317	0.5415498866764807
0.8079376414675272	0.7958944172283179	0.5368182637505652
0.6905653947048664	0.9229422929561388	0.7857234010105527
0.7041349967273788	0.6747339083671036	0.7919872012702337
0.1940632621449122	0.4200875080761679	0.7870359476961291
0.1930087497580182	0.1951212802893584	0.7869648628335794
0.6941576340129308	0.0712941233962800	0.7369380038036168
0.6936437133218518	0.2976317003823096	0.7377011260695426
0.1895716901900007	0.5698478745015898	0.7399644310714208
0.2006054798269603	0.8147030163108309	0.7387605563720291
0.3098740193357497	0.9198627391782239	0.9904625447647122
0.3109737333285930	0.6966349835882722	0.9912560774287307
0.8152513109494550	0.4220583879583816	0.9862526584716290
0.8011678522685601	0.1782025482396523	0.9850916079712266
0.7075278747478337	0.9124357927231986	0.9934686440486734
0.6937522537006356	0.6767267228432138	0.9996544698809625
0.1872489069989140	0.4309973932168821	0.9930934168092588
0.2077641447091556	0.1841292701735962	0.9935477293667757
0.2961579217991624	0.0784254091080105	0.7435660700032666
0.3091336315921010	0.3118082546934455	0.7469789176817168
0.8238774671263046	0.5613114352390940	0.7466474920775001
0.7944483048812438	0.8050655194867009	0.7441408310680254
0.3173277182586952	0.9303935713384225	0.7850396059194070
0.2936707277968947	0.6850181455379799	0.7847439313331217
0.7979641515560199	0.4117962191487465	0.7798989377502556
0.8092241526645982	0.1806479030165180	0.7768599482195324
0.6861414152679838	0.0613466544043329	0.5314382896647163
0.7092380234241077	0.3088786213360965	0.5317374030024895
0.2067068983226819	0.5795998872085821	0.5319128832780968
0.1935678985235148	0.8117976673960325	0.5303605985407733
0.0842465761853157	0.3722228260441315	0.6359655105143757
0.0879266673698876	0.2171656813335796	0.6308348632907036
0.5892059155509413	0.8715912526732047	0.6433541811504689
0.9231151833289540	0.6176836783746650	0.8865952381490700
0.9155879721487867	0.7715610654713879	0.8787959815521614
0.4154241189208738	0.1204393204094171	0.8924579801151357
0.4188127742794435	0.2791118033560845	0.8973170036633630
0.9168864173377743	0.3686438130701476	0.6312994269654493
0.9196262843582673	0.2104109931126879	0.6265765901152563
0.4192697830566259	0.8718894466162905	0.6432374788394384
0.4152585705550444	0.7174876866642403	0.6483135217333230
0.0898109136671659	0.6216943323751696	0.8831076677111334
0.0843691679710393	0.7798139403062790	0.8792827512286165
0.5844239919754040	0.1180614214405544	0.8883198456342654
0.5880485650195142	0.2733264608523344	0.8938335232961648
0.1339240314003441	0.3821283643362906	0.2259651162483566
0.1156374895944898	0.2099028480389462	0.2339815817021720

0.2508341146510512	0.1133005352693555	0.4851807752243972
0.6122562609476780	0.9102371072107517	0.2329655760840161
0.6283454875100680	0.6736763645697798	0.2307983793338262
0.7384206477951338	0.6159072322549357	0.5471820697430093
0.8735880563712540	0.6128274794765548	0.4785108497246480
0.8863460893512716	0.7856573772350720	0.4850027167035747
0.7534099030000396	0.8789141668944802	0.2339012005216059
0.3907498071418752	0.0791154628014584	0.4821703004864207
0.3744871045700169	0.3222788399353217	0.4756440906985122
0.2699651053696814	0.3770877349382057	0.2920538701996276
0.8923754210175319	0.4114877983880310	0.0404530209352110
0.8732492987102434	0.1736305776468939	0.0492053292644323
0.7674486966018225	0.1165204139515407	0.2319618308947868
0.3704992505282482	0.8809278284912815	0.0508193188377087
0.3877819731965352	0.7117638218975232	0.0434022521695696
0.2523298477263035	0.6154225029709739	0.2896278324008258
0.1121977168600465	0.5816411228317111	0.2919848974267132
0.1303118836534990	0.8223160980226020	0.3040698181817664
0.2348697368460669	0.8758045134580612	0.9853906270362809
0.6321498770636449	0.1107661356398316	0.2983497845883422
0.6162311048193976	0.2846649222241009	0.2911413690175172
0.7509571078397579	0.3791970063292540	0.0368466753232898
0.8887195347841699	0.5722505850374410	0.3064103831244036
0.8646375431033896	0.8181324483060209	0.3120436070610961
0.7821353223784926	0.8702634286682323	0.0128817913443909
0.3629037708761528	0.1098387437428032	0.3088881714216923
0.3859328039572109	0.2968914595521205	0.3004842635506630
0.2443716718122915	0.3710753262954859	0.0128057044043546
0.1145222103994680	0.4229958335787306	0.0562198654784278
0.1387524723135697	0.1762097313185816	0.0620217029735642
0.2211265637966726	0.1211970007960052	0.2641804090126971
0.6409572404136870	0.8886812046838901	0.0583351602917857
0.6162429688458134	0.6860432592499947	0.0527127612343187
0.7628789958059846	0.6239394575942790	0.2442541417340822
0.1401606899681013	0.6031800991614650	0.4669820050219901
0.1154557453652905	0.8003372688565714	0.4799685731107892
0.2605222612308413	0.8736817972424813	0.2619554047803702
0.6127751757186271	0.0699353785619921	0.4690764678279223
0.6402835340005436	0.3172225906262228	0.4626655174226763
0.7217381630945207	0.3730923346452251	0.2614660291784282
0.8630328862099429	0.3910387897471098	0.2155034971000274
0.8855817647511905	0.1931743808493273	0.2261264317152368
0.7406985549883982	0.1237277396189719	0.5137865552830452
0.3915884489699176	0.9239436473818651	0.2197075379242522
0.3630266263339957	0.6779753888581217	0.2150724566252558
0.2827709246610129	0.6190559813082398	0.5098755559897712
0.2549146977818232	0.2484165176013764	0.2542650134343157
0.7427733613594061	0.7439148498308760	0.0111821637121387
0.7629795744343869	0.2511054027578263	0.9949131451680061
0.2386761315491898	0.7435646267722841	0.2479123111922974
0.7404488264103368	0.7514836135003724	0.2792100427410816
0.2641906097768404	0.2408142036336230	0.0307243299952583
0.2469306534959037	0.7468402654261106	0.0258196064247929
0.7559037097938198	0.2456720426135980	0.2706716722503149
0.9998119848686984	0.3921542988843074	0.1580147781567576
0.0037194816911669	0.1911234919723710	0.1233309315268138
0.5048338790491965	0.8916841661558613	0.1163568957194399
0.4984206764099641	0.6895865982567768	0.1553341464959731
0.0043433183189647	0.6021553539435407	0.4066145980560368
0.9990774450271599	0.8027705359977448	0.3749688044150851
0.4989315159135608	0.1023567208739015	0.3672567827872399

0.5049814116353418	0.3024878592523567	0.4028342837553069
0.0913704633457537	0.2969429378605703	0.1009543296408914
0.5934373908890065	0.7905383669095223	0.1611367692489909
0.9118684340175044	0.6978162221197023	0.3517691526165921
0.4112581008682495	0.2013425923992944	0.4218682907727189
0.9096229027271214	0.2925403971756990	0.1116596852350557
0.4138471975760287	0.7975030125144653	0.1775224673157452
0.0950721917839620	0.7019439398807539	0.3620990480257616
0.5921748371011827	0.1963221551254223	0.4236060310261321
0.1767301729054581	0.2048097917365614	0.3941712923518869
0.1605364144468336	0.4290449522340656	0.3879602039655442
0.6889486440717623	0.6555307385563613	0.3887665324553647
0.6823470100436523	0.8994385635302722	0.3893516047757810
0.8285156473412562	0.7882142190015099	0.1433996575206343
0.8482063742815229	0.5571829994838968	0.1348628044967399
0.3297624078882568	0.3253479917320387	0.1428174902390924
0.3236026305946711	0.0933420889011338	0.1413029082236745
0.8270235452079511	0.1662004679468963	0.3825406320128634
0.8235568216852773	0.4021132948026987	0.3843509627880725
0.3254400906279012	0.7056167525357430	0.3837741547016990
0.3456494067261278	0.9270469344884252	0.3884770701383410
0.1733197490066103	0.8267670666786729	0.1351178123729534
0.1814358871826890	0.5921391114362282	0.1345258860249286
0.6770453016132375	0.2897287950361261	0.1313346194514803
0.6585806217855693	0.0638109329493741	0.1362200517221505
0.2141350081469507	0.4928124880615592	0.2441067742178006
0.7125369840180851	0.0004781612999485	0.2805673324883582
0.7928461617121935	0.5002372708842752	0.9871682535524959
0.2901843042774095	0.9913760807446055	0.0361783591262377
0.7772845221037369	0.4930269707499180	0.2652435146177373
0.2813249576194157	0.0035562259590813	0.2654019754227803
0.2279343051309330	0.5013404542338004	0.0148890242158346
0.7266727405191004	0.9905896988632501	0.0120424725037198
0.1340572435552071	0.3808228113121728	0.7261016299688784
0.1162432665483558	0.2092983786831196	0.7345079022630325
0.2502380838401554	0.1131210664432859	0.9872934350410136
0.6133211132264647	0.9161087560834683	0.7318085851529653
0.6385353745507378	0.6638422038263374	0.7227463107735932
0.7374348480815949	0.6136370951803054	0.0438322388717463
0.8725999796088659	0.6114553168515167	0.9762415689741930
0.8830829495004822	0.7776969583800678	0.9806932704278675
0.7533150771342589	0.8769389685418043	0.7381176675748978
0.3907697618597857	0.0798147082756504	0.9827398367487521
0.3742687705438241	0.3190940235925691	0.9753240186628704
0.2699096112009062	0.3769171967578923	0.7914490556938854
0.8939116505366903	0.4095737428523607	0.5408720633939142
0.8737912819481355	0.1711961682057239	0.5491836876368159
0.7677661021807409	0.1174750495355204	0.7310097917337488
0.3707113896944954	0.8809615399090563	0.5517880742006454
0.3845104172865455	0.7101958441170680	0.5443117998296271
0.2519772704741500	0.6131315811426622	0.7927840812582675
0.1117320453414913	0.5795500642749150	0.7927025782273365
0.1278443716467940	0.8208997138671644	0.8014178542187164
0.2362990175769067	0.8736846556886355	0.4824163661742086
0.6325385090682896	0.1095536640681987	0.7971858266799418
0.6170746916836273	0.2827714649184079	0.7906996215586269
0.7512456626473230	0.3803292150912546	0.5380489310004833
0.8977352114563508	0.5648760835145966	0.8091935221059566
0.8668000865379234	0.8141310718632322	0.8079759857543423
0.7784898633336717	0.8712689743593709	0.5189054035259991
0.3629996700120667	0.1031379518302322	0.8076732998124027

0.3864419958270994	0.2994198270142294	0.7986935185081632
0.2433994108665778	0.3714349900226395	0.5127859276821556
0.1127990643916521	0.4214660309506035	0.5560383224488930
0.1408438204707396	0.1755611352231990	0.5631747039209963
0.2215324683245560	0.1203451520897976	0.7644879796716020
0.6360888855613046	0.8975295243846871	0.5562885337712588
0.6168257104509866	0.6877805474732054	0.5364025642782149
0.7701374611570335	0.6210958139452804	0.7747200002564718
0.1451027224132919	0.6048548368152443	0.9652775966531001
0.1175802800662558	0.7979794035793737	0.9779575292112770
0.2601549758465098	0.8700644226676447	0.7687373525268608
0.6137960378161342	0.0688846123239108	0.9675538147723334
0.6383841234652239	0.3163672292106906	0.9630662210691612
0.7207254066582226	0.3731501482587307	0.7602363071748842
0.8623260085545490	0.3844570767911683	0.7142872329065412
0.8869235795172522	0.1928300961602336	0.7256807802920194
0.7428055754146553	0.1212199665383779	0.0127653477445795
0.3893668391111236	0.9223339625949590	0.7214271935691240
0.3603426616524505	0.6749280013110994	0.7144067701574710
0.2855562509592369	0.6204636314415023	0.0139817751034969
0.2559575097404571	0.2473704894285821	0.7538034333944139
0.7484884216309631	0.7434275972340639	0.4973660992215789
0.7660579011017821	0.2525817879691598	0.4955502238496270
0.2360164861690866	0.7406984739178093	0.7487115228123867
0.7410659368083615	0.7491851101606876	0.7880461997956435
0.2652404647901075	0.2411408812752523	0.5285050903223705
0.2427843033450138	0.7444241196664597	0.5231809074459619
0.7577735500999520	0.2466395443048252	0.7693006184494701
0.9997466512339984	0.3904050085699389	0.6601876662150299
0.0052025686921614	0.1877678467919096	0.6229040734427967
0.5035831775953983	0.8901823630891103	0.6210106588966197
0.4956622680121510	0.6878388373050489	0.6550785487748571
0.0075658896827161	0.6007055854673302	0.9140533410116437
0.9986993213533293	0.8010645331935669	0.8778558216337855
0.4991409008726595	0.1003867591746388	0.8666417479774911
0.5044518544690788	0.3013164361938012	0.9003721897513330
0.0909059379840716	0.2954770678089407	0.6013018769056839
0.6020706927863937	0.7945923762454203	0.6625390318026020
0.9167999912610036	0.6933925732006841	0.8480547236441488
0.4095116523203114	0.1997583940289039	0.9141126452072450
0.9132160047214486	0.2898692121281851	0.6077599619653106
0.4084852902004358	0.7958040422745816	0.6785250977964464
0.0945039840346956	0.7005344075519342	0.8597383920283994
0.5914356663785589	0.1951150643391703	0.9227330019309770
0.1779584915775797	0.2046071888172024	0.8940660256504077
0.1614711368082257	0.4291292912142420	0.8881420924914154
0.6751286216188853	0.6630585498922084	0.8951288112616331
0.6802639796994818	0.9013409120510872	0.8904501695080993
0.8214002239406836	0.7833948050456258	0.6436908602276176
0.8493864675145408	0.5657345534782068	0.6416434274374053
0.3287388101874938	0.3263047228942736	0.6421481772958162
0.3218590950208684	0.0910190372296299	0.6398601421253709
0.8274027256354027	0.1667768000613100	0.8819819948168282
0.8245560715965318	0.3973841847480584	0.8828180752386078
0.3248053754108491	0.7055903257940352	0.8838190931664420
0.3431897587273218	0.9306727819104124	0.8900184555134301
0.1766713400699332	0.8277937849090823	0.6351250963752995
0.1816386463534760	0.5930395982449802	0.6356600821368011
0.6773650519162493	0.2892185174560535	0.6305468975582862
0.6607774917243013	0.0609745923499940	0.6363871289112356
0.2140441896140663	0.4917582339803673	0.7422857886980483

```
  0.7170336887229238  0.0008024294714235  0.7824965555771585
  0.7964850773011245  0.5017982972356027  0.4973266053984332
  0.2891690736171042  0.9904200521293485  0.5309893268815822
  0.7823629347531094  0.4902434555401599  0.7628645417600349
  0.2767778293374034  0.0006719571748927  0.7616829758102251
  0.2253209889139892  0.5010158795142294  0.5163755471232014
  0.7282769923585379  0.9927959702364685  0.5052714921869799
  0.4605695293196373  0.4996024148526953  0.6975516986386546
  0.4243621708658739  0.4741275269949442  0.6222607231319358
  0.5268581097085578  0.5355514749410011  0.6840587016909460
  0.4522014534588261  0.4841509149015039  0.5345128525593790
  0.5559776380168227  0.5457901738695656  0.5994458943168886
  0.5201485744057468  0.5214988344471747  0.5203560900484870
  0.5569381835790834  0.5292723593646818  0.4284044367360624
  0.5102661020956043  0.5053709070267187  0.3499669027824102
  0.5270567162545660  0.4467631627932055  0.3029517356107972
  0.4493875913935917  0.5430577212891498  0.3220649924023540
  0.4839749313372294  0.4258735207659343  0.2302696768774776
  0.4062799227850202  0.5222947816352832  0.2494818826216249
  0.4233541247087905  0.4635781592522304  0.2034692827642764
  0.4283582974851526  0.4897766168880517  0.7900949451663080
  0.6322234990112684  0.4950611791014829  0.4340238228813597
  0.4816217725703462  0.4990115021699531  0.8688666316119430
  0.4537885746754532  0.4870886185069060  0.9325398474354725
  0.5295300269420500  0.4658885680909019  0.8625514947293473
  0.5016754669712699  0.5505920077450785  0.8736169325803419
  0.6656965523341684  0.5176438596039691  0.4869686374378261
  0.6266426647940337  0.4416272728599258  0.4485265128471130
  0.6614231515220919  0.5012261252293755  0.3698452552493575
  0.4030215328145650  0.4399445239943548  0.7925468476137707
  0.3820635822843388  0.5250833813995670  0.7966900976376570
  0.3737231224931774  0.4456378704577769  0.6303213943715674
  0.5560625093510320  0.5567463402195285  0.7414290213036216
  0.4245346440575049  0.4636832783242419  0.4753826197999282
  0.6072013516360216  0.5733761287453712  0.5919447237826612
  0.4566469957938785  0.5500035168706714  0.5198022207249613
  0.5748799796295095  0.4173609361164684  0.3218910221347461
  0.4365708954390777  0.5899789293155038  0.3561408774625418
  0.4979354559246834  0.3799378079296388  0.1949583938758832
  0.3597909905578622  0.5524161788580236  0.2282824116333400
  0.3895127697159278  0.4474356536244972  0.1469084990873652
  0.5670848310889732  0.5831279482976958  0.4197386107180607
```

MOR-Al-T4-TS11

```
   Al     Si     O     C     H
   1.00000000000000
    18.1842269897460938   -0.0090872207656503   -0.0001607598969713
    -0.0105508984997869   20.0691013336181641    0.0015994716668501
    -0.0001797722798074    0.0011982563883066   14.8440780639648438
   Al   Si   O    C    H
     1    95   192    16    19
Direct
  0.5827484775719375  0.7170561303761327  0.6431690639574140
  0.3113913081043896  0.0708429705976771  0.0330295411307411
  0.2996828828269857  0.3149829406137495  0.0363202571557679
  0.8076811543318204  0.5718605761854328  0.0318268426812125
  0.8047572194394268  0.7962461796085170  0.0333115273687080
  0.6837042240686364  0.9224235258343677  0.2822563777515003
```

0.6992473369178416	0.6776741573363814	0.2824298520008768
0.1903420527491884	0.4217473008223648	0.2855272294302633
0.1892848221020174	0.1971790846172353	0.2839069501404255
0.6894514595561962	0.0732570083612380	0.2354125845513083
0.6924042241528444	0.2968226110880763	0.2352326170744712
0.1854128051587321	0.5725827315870911	0.2369671360135283
0.1974530154285485	0.8176901059664663	0.2324469199749251
0.3065244067649248	0.9206620295100626	0.4846118484470301
0.3000096062898209	0.7005108586661483	0.4861139179444619
0.8109175225840967	0.4251126093887003	0.4864351676208217
0.7987399160168652	0.1793117033877555	0.4815862632198923
0.7008195843709913	0.9144081827527604	0.4901240312584086
0.6947520759156249	0.6821467409829138	0.4900921097796530
0.1829093378355811	0.4325010008244168	0.4911462970440482
0.2043263085032961	0.1863161266814154	0.4897843633655266
0.2943622002514510	0.0820198218770211	0.2404533289748033
0.3056185475661198	0.3141827904797415	0.2446372155247470
0.8147593377585453	0.5630023766515493	0.2352413728250360
0.7911172685659933	0.8093400337021063	0.2382544742670452
0.3155301237718554	0.9327803772044237	0.2791972668940252
0.2898542272587517	0.6884945096673184	0.2798176615092648
0.7961299435159048	0.4150045363763638	0.2783126856519750
0.8076157795909427	0.1791507701412825	0.2746650494034543
0.6831760833698097	0.0625208165734807	0.0302099090549539
0.7052316223205459	0.3085781987126224	0.0287635168976535
0.2045907972952938	0.5811632805842534	0.0286466710730918
0.1886580961629732	0.8148796070895216	0.0260867896287311
0.0830555918108307	0.3741965691110384	0.1315520105002189
0.0852588896635262	0.2191982681010196	0.1266949862420029
0.5833161705566148	0.8708910075314747	0.1376716478883777
0.5796193888606056	0.7115569426351828	0.1448089456540916
0.9138431104427044	0.6228844610665062	0.3826230582683641
0.9107985123912127	0.7789871395115082	0.3781621306407879
0.4127585957693244	0.1231493408168706	0.3904027548471135
0.4159854777006361	0.2814750616167039	0.3945492138035918
0.9139483182920028	0.3726690336690502	0.1284162088508173
0.9159642201491327	0.2134645612724136	0.1240776168736583
0.4156803926338629	0.8749043714698064	0.1367481435487113
0.4111968646118108	0.7207945688587707	0.1434432589923120
0.0833923371705974	0.6256270175784152	0.3796385161108251
0.0796947195511920	0.7849700358904226	0.3752270800296651
0.5811313341977288	0.1210204128256997	0.3875463803385149
0.5849296710099879	0.2761849018837775	0.3920075524895666
0.3094761713104477	0.0702724767227351	0.5321792224602070
0.2993213991794179	0.3157466088101106	0.5367295903256855
0.8063311866026629	0.5739827617373240	0.5360766767123820
0.8074143151919981	0.7980950872223783	0.5316947638401834
0.6877551030183892	0.9266593481324815	0.7821884671670944
0.6985050720725448	0.6768835748695392	0.7873812037372805
0.1911765773897101	0.4229593089713346	0.7833602740430252
0.1894819187641856	0.1980727718287492	0.7839959578400070
0.6936402073901227	0.0733028926109982	0.7337867718783039
0.6911351664958127	0.2978097021360640	0.7347918794227712
0.1856320883720353	0.5725615681255147	0.7359494451184485
0.1960149015937779	0.8171473139170168	0.7336187166223375
0.3055422332327387	0.9214459033887801	0.9860330939096136
0.3052774797505788	0.6992721199676502	0.9869964438917511
0.8112622789032938	0.4227212508966665	0.9841401353225064
0.7990793711771426	0.1789163557361510	0.9815965660143314
0.7032426635843445	0.9140862045199464	0.9902258408567923
0.6895516449600373	0.6779033780326970	0.9961379170340655

0.1853803875727386	0.4326068254581827	0.9891720625810254
0.2051491847315781	0.1854580924988014	0.9897288709058221
0.2927262491266748	0.0806629323134945	0.7406815060872307
0.3064358387659191	0.3145398732896423	0.7445315975281284
0.8169677094501608	0.5621361169234070	0.7413702288456253
0.7897372395230192	0.8069313516154680	0.7395239928840641
0.3121143496036283	0.9332354700977495	0.7811422042802615
0.2901060284935177	0.6882255502413650	0.7800693235501747
0.7939243905095685	0.4129080036214132	0.7776162617794800
0.8074970004219399	0.1825531988911847	0.7742419591696584
0.6833457858516989	0.0622302709837855	0.5281639754712241
0.7052893733771934	0.3087649268564390	0.5284123157346654
0.2022594492247485	0.5819273051773223	0.5278175672190665
0.1855321428261944	0.8157664186753453	0.5251413031538675
0.0810635497013090	0.3749125168090555	0.6336101357778534
0.0843282278333681	0.2199580983746190	0.6276061020551221
0.5839467811802068	0.8769895269915331	0.6430506022915096
0.9171012919337697	0.6192526054653950	0.8811213275252117
0.9105015912054630	0.7732692487491577	0.8740177967250848
0.4135455167519720	0.1210393826586313	0.8885129345119356
0.4166362503773721	0.2798827916800297	0.8948055332950884
0.9131028737677087	0.3726923376655343	0.6282643252842618
0.9159356066425119	0.2147100016077517	0.6237018797579463
0.4152669842320127	0.8770660156891340	0.6393094116268138
0.4099714837723135	0.7232641519248165	0.6430427204431814
0.0846703956082447	0.6227687941497281	0.8797812319084634
0.0797487443460980	0.7813609576818765	0.8751800330992312
0.5825546401705470	0.1187401534468851	0.8853330572289170
0.5857303467240885	0.2738653950747267	0.8910920121524643
0.1310635862242283	0.3828030285764922	0.2231793462147356
0.1127594404206127	0.2105670451619362	0.2305535885560909
0.2472746478534097	0.1157449913207264	0.4822253828230891
0.6060936488657003	0.9110487789204388	0.2291541037385660
0.6227598302017721	0.6736961911903384	0.2258842552716774
0.7321831406340499	0.6183107924257579	0.5422759609875044
0.8677148128866031	0.6107767986008067	0.4743056955312283
0.8878099120908556	0.7921920230042734	0.4828600922169226
0.7467445669387608	0.8794411939461176	0.2300660633171674
0.3872415947942291	0.0813126057251586	0.4791603723098578
0.3710298428883531	0.3221715572059034	0.4717657133649299
0.2670709046612806	0.3796646113622578	0.2892352663229382
0.8888015144701735	0.4122511373401613	0.0376420768781887
0.8711779231375825	0.1745890267473779	0.0455947626059345
0.7664890629726268	0.1147710590784994	0.2312523411635118
0.3650600742821845	0.8816664284543451	0.0471527664071773
0.3817995109596859	0.7136148740224266	0.0402226855056509
0.2492968845174690	0.6162018833784603	0.2869176749270473
0.1078282879728339	0.5852704420809143	0.2887338781057326
0.1277117059491689	0.8239222895666747	0.2995338904417613
0.2298577242104571	0.8784459979234781	0.9800849032280519
0.6306433324788449	0.1137283051714364	0.2971056018746484
0.6156820083776413	0.2851381659212256	0.2893808552513080
0.7477344816786622	0.3796666308679021	0.0359591329997202
0.8848223747580414	0.5747376572071983	0.3015950639284651
0.8572436105866845	0.8197231926209677	0.3102635033440322
0.7774604684381776	0.8712814306973446	0.0086987095162950
0.3604889243890712	0.1079337458241369	0.3047178746091888
0.3828173747815372	0.3000847591636108	0.2958750609934949
0.2430255425994814	0.3726890218480802	0.0062725489933473
0.1132391648941504	0.4234755249373408	0.0529117844717800
0.1369370365321701	0.1761837995800175	0.0591286041520635

0.2189271257091897	0.1227757392021193	0.2611943219705085
0.6364451398989497	0.8895955794267383	0.0543668621071654
0.6115502179716804	0.6876063886461006	0.0480381254418856
0.7560879605613783	0.6226894003395721	0.2437393045108748
0.1345799409728316	0.6042543294013957	0.4639289794662526
0.1065174033450578	0.8082330020688974	0.4751120502781038
0.2584711513661961	0.8732049643363820	0.2575285664046165
0.6093061396958614	0.0720926488114533	0.4673957541049202
0.6351959815311997	0.3189989971632084	0.4616319515508567
0.7222890081145454	0.3717377405951304	0.2567623122343662
0.8633744853576772	0.3925891261394590	0.2139434780701188
0.8844724383814250	0.1922522465902054	0.2224051319142193
0.7392330257957164	0.1232110263387748	0.5082350262372137
0.3881547563739727	0.9254914604704134	0.2155683289547290
0.3596962762369473	0.6787393034096572	0.2128194343310404
0.2771877167383894	0.6229098901186103	0.5048346817245186
0.2515582217551060	0.2503559252836470	0.2518337183009489
0.7389193736790260	0.7446779035066429	0.0094386023895272
0.7609807076691950	0.2519761748787793	0.9911964210752128
0.2336405188698005	0.7436118621034226	0.2393949235651973
0.7350620420498437	0.7514190764336811	0.2725534002102024
0.2610064138092553	0.2424381500711782	0.0276479492630255
0.2413507493225709	0.7495953341010869	0.0215414679248815
0.7546556179632147	0.2441369533151849	0.2661614684029211
0.9981221851828560	0.3926775506247510	0.1528287278323475
0.0017186491454326	0.1922331052988510	0.1185408244505691
0.5000154857171745	0.8922382316661478	0.1105936088913514
0.4934693023173183	0.6905492672294721	0.1503087302590028
0.9993381681354618	0.6054059178971719	0.4028456598342105
0.9942660198137719	0.8050438019249461	0.3631138817668544
0.4962948532994636	0.1027551393419784	0.3639302075769000
0.5014433770854289	0.3048034003751497	0.3966352721902097
0.0903270834721955	0.2974474175786870	0.0972018066597783
0.5885361108792649	0.7914832708946795	0.1573839691341627
0.9050025300349891	0.7001270785464292	0.3518427428789135
0.4081100850364046	0.2023275186864961	0.4141388605460357
0.9066069856162414	0.2932760140735418	0.1094969425422741
0.4094198707180262	0.7987202152403472	0.1742458343508556
0.0915324685549962	0.7051033017713618	0.3619048253712903
0.5870798093945326	0.1982035939934224	0.4217070174450487
0.1730861551319477	0.2064759719764895	0.3908315554693311
0.1567806874748496	0.4284533232272968	0.3864396797411145
0.6802495479642350	0.6588124312093434	0.3856740152341967
0.6761762205581953	0.8993810999950345	0.3862824686450259
0.8250392554189903	0.7905957998064553	0.1400121172011846
0.8432244285790785	0.5593164684203121	0.1313464799621556
0.3250453870673580	0.3282544953599205	0.1396959135663239
0.3203251555000521	0.0950855905838373	0.1368465265168946
0.8273641803413514	0.1670356125693549	0.3796290542044201
0.8210543411684985	0.4024558928892018	0.3821094169478365
0.3183299729409204	0.7101546785743286	0.3792650914763416
0.3407627310906321	0.9297867881142295	0.3840383487190764
0.1688941242674356	0.8302265215722250	0.1304877790090795
0.1775110439621792	0.5932489160554204	0.1319666567343712
0.6742865178852081	0.2890783241018929	0.1284084332868723
0.6556122140203268	0.0666857522184862	0.1344111875849919
0.2093551290780945	0.4945704938046541	0.2437098798338534
0.7061579848322191	0.0008144013023392	0.2787137424784245
0.7881894110655496	0.5006404513130496	0.9850131129520204
0.2868424655427588	0.9931209791541282	0.0319215117969867
0.7746103239605933	0.4927118674815945	0.2618230925897079

0.2762898219891296	0.0039738052862272	0.2584186338213713
0.2251831909802336	0.5030039761015362	0.0128452238301386
0.7232925526593021	0.9916768708175572	0.0116708058951280
0.1303984594137322	0.3829592873999556	0.7241645954364705
0.1127030063200973	0.2115615525459636	0.7311922519990967
0.2487363040186448	0.1151255269960845	0.9817411408114698
0.6102994966102394	0.9243159408299094	0.7278943497864332
0.6308915709626853	0.6684599531986285	0.7206752866598830
0.7321285969617993	0.6142737473592704	0.0407374649381302
0.8667579281079784	0.6122402956954314	0.9709682560968796
0.8786040832748867	0.7794050495756331	0.9763281433826628
0.7480313008761448	0.8785093300511161	0.7336851559591392
0.3887970897394111	0.0804612582612231	0.9788928276011883
0.3732657544991310	0.3189802168275948	0.9746124450456273
0.2674155848961594	0.3803673265431938	0.7868282550538260
0.8886408100013319	0.4153170569856581	0.5397049793192921
0.8693633434925903	0.1745577505072648	0.5479742665574974
0.7666942563144341	0.1200907354888083	0.7259849522882039
0.3670585744318357	0.8842841279379474	0.5471559691293203
0.3725143501352001	0.7199679153944771	0.5421468936253818
0.2478926819625873	0.6166263477243666	0.7876559533176971
0.1077198896378795	0.5818693397173399	0.7888661471727895
0.1235434445748250	0.8218699018648288	0.7971045319308024
0.2318820598617000	0.8760647850965252	0.4785063383723088
0.6319456501899606	0.1107467877147986	0.7950550653880391
0.6144340931934201	0.2831707321808922	0.7877314362797408
0.7483680323449438	0.3795007617869482	0.5357499619858413
0.8913182489992699	0.5673516891255818	0.8028723281289812
0.8620383857372177	0.8164113630777657	0.8034378899030591
0.7742889076027529	0.8721011623075462	0.5139486729868025
0.3607572100749838	0.1041560607475015	0.8038999645811014
0.3831301802128250	0.3012283409863659	0.7973184578555800
0.2416049526666648	0.3740072110247330	0.5119136453398040
0.1105311936283665	0.4243658933988920	0.5545420864902917
0.1364365450944326	0.1778018170448476	0.5595823453170728
0.2190269272039778	0.1238128300252161	0.7611696298479429
0.6322382253854197	0.8966975873379287	0.5541043483306162
0.6193352493396048	0.7027037960083461	0.5356852930355903
0.7627327335315940	0.6221938384578061	0.7662849371085880
0.1382695871515743	0.6038336795024473	0.9630390836092731
0.1120666505369301	0.8011931848770804	0.9738015948413035
0.2550201936082114	0.8730245719165768	0.7637327269726223
0.6112928281551864	0.0698758962122944	0.9652448464327460
0.6360473319643689	0.3170296464112930	0.9603379359168761
0.7174787180002299	0.3737162134660527	0.7554272771838894
0.8597622779996212	0.3875150681324721	0.7129041471732249
0.8847745965172584	0.1975506161915400	0.7236946534809152
0.7404657149114773	0.1223597512139345	0.0101308474787103
0.3835705794098278	0.9275658399904404	0.7162943258580526
0.3585247495854836	0.6797809910462584	0.7119756887736340
0.2791246914317972	0.6233193258142090	0.0091429755430219
0.2518659161550333	0.2510645832279224	0.7520844931103634
0.7546147991981677	0.7426823526172476	0.4857471329798313
0.7603619277413739	0.2525050935277311	0.4882733706327070
0.2326338600338856	0.7435921183097156	0.7420057296192156
0.7370989665073674	0.7506954300274595	0.7836447881778492
0.2608086687979665	0.2433581597364756	0.5258985815521490
0.2299337931517616	0.7459913687275499	0.5142508105724468
0.7550932513951035	0.2480054680475040	0.7695727671241989
0.9963704338744077	0.3934838280151156	0.6563299172991773
0.0012556037621642	0.1916847066491400	0.6193778441102237

0.4992859901991336	0.8976138487564682	0.6182934671119074
0.4906478244411729	0.6938325769629062	0.6397047986658131
0.0016189497262084	0.6021787352157945	0.9081390470477895
0.9939058851923607	0.8019795396348809	0.8723633442608770
0.4973809613904085	0.1010393593060472	0.8626134104910776
0.5023098758441988	0.3021076816205548	0.8974191867511921
0.0878722662531676	0.2983052128918094	0.5983823132842992
0.5929591097381928	0.8008257838029960	0.6699415137653950
0.9105074234518991	0.6951156283028653	0.8433450074398340
0.4078796310071148	0.2003695752590496	0.9104447822478204
0.9087182278778334	0.2940052054857281	0.6032128206492986
0.4061135179780174	0.8012580793508561	0.6761923712282547
0.0906891190044641	0.7018628469240844	0.8584157012001968
0.5891019415355757	0.1957728691556652	0.9201583167402544
0.1739359831488750	0.2066509701319255	0.8912301069149529
0.1588598619269072	0.4319463511047673	0.8846725894693241
0.6723827959781147	0.6644525530786072	0.8914287579821093
0.6763359469480530	0.9049749666236310	0.8867352982535291
0.8166991287860155	0.7850147765811979	0.6390818152611123
0.8411693494611920	0.5633000269407781	0.6358527543717963
0.3276129319802586	0.3261987460342795	0.6394324886923313
0.3183897402182793	0.0923222807666651	0.6367569264800493
0.8254970881617154	0.1666771247588773	0.8788381457039006
0.8194539096230087	0.3976359684348597	0.8807573125688712
0.3193415329017341	0.7091746547001002	0.8799154560243857
0.3392398638236634	0.9319456402609987	0.8857502837946947
0.1707458226318236	0.8310033391670391	0.6305877399682751
0.1772071020991879	0.5951384013032712	0.6314755080795236
0.6756078743767602	0.2874859130862305	0.6278014208165741
0.6595607394584657	0.0627481935014682	0.6336573127742855
0.2097733913736718	0.4945190731490660	0.7380903346195101
0.7182025424518745	0.0033857795233680	0.7796844966601306
0.7851102497993648	0.5022741219244550	0.4908939092222323
0.2844489020246894	0.9928939858013812	0.5266378510576223
0.7764231654903202	0.4912183059204048	0.7623753394151372
0.2709523874533049	0.0036431835692358	0.7603150919839061
0.2222062085936050	0.5037376754998742	0.5108452462606555
0.7232869128415488	0.9928336891076429	0.5015095191809629
0.5169392266889180	0.4257744781148353	0.2146948242861768
0.5676569129259429	0.4553638753414780	0.2723936115204463
0.4444993303264049	0.4494503582171391	0.2118600864878505
0.5448101902900958	0.5070106705436369	0.3309051996061239
0.4207716656085702	0.5010264657482246	0.2689958977749620
0.4700390802862044	0.5297154362429620	0.3285171191853117
0.4504797134673432	0.5731711326904024	0.4083945379211230
0.4810054009404532	0.5335250131132508	0.4897824374854511
0.4438910452994541	0.4744493194079110	0.5193324672366703
0.5354429986685315	0.5606285366565925	0.5477707216936425
0.4628195619686230	0.4443027092878669	0.5997886640890484
0.5538064434696619	0.5299164560472627	0.6284907350746310
0.5169387154355083	0.4721152396706679	0.6569311564865052
0.4836188233840432	0.6430808077811533	0.3979522974378760
0.5325829348763300	0.4393444821326346	0.7461011380183232
0.5794486188449911	0.4794309788379540	0.8121513307393173
0.5542611683047468	0.5279985577112883	0.8270953151095453
0.6348660295927798	0.4885439036338454	0.7854210994361750
0.5854416839801035	0.4518946571700375	0.8757365108368177
0.4793653364133584	0.4261110410583605	0.7770322501711304
0.5589458270472558	0.3908791198966108	0.7324824024743801
0.6251715853292068	0.4404243084773983	0.2711322609452861
0.4052171441981805	0.4260610492086838	0.1662103576477204

```
  0.5876203917205693  0.5404217679952713  0.3573270311667298
  0.3632736647499905  0.5165745530275999  0.2690427592332100
  0.5281238224449233  0.4960766059628274  0.4244454286935483
  0.4013231804898734  0.4526007580041299  0.4766552306323414
  0.5626474872284857  0.6067896017859546  0.5285782216151494
  0.4343373645222176  0.3990252103061808  0.6207735667399776
  0.5950681086766001  0.5533874319407754  0.6713307704108252
  0.3903621778295433  0.5765114466850436  0.4153436821420378
  0.5432928216650639  0.6408255718127354  0.3874103505948483
  0.4590903021079194  0.6675050379682573  0.3391361136187052
  0.4732127828130740  0.6734620425114640  0.4578460704511421
  0.5335728317108837  0.3849073857014261  0.1708511164539329
```

MOR-Al-T4-TS12

```
   Al     Si     O     C     H
   1.00000000000000
    18.1842269897460938   -0.0090872207656503   -0.0001607598969713
    -0.0105508984997869   20.0691013336181641    0.0015994716668501
    -0.0001797722798074    0.0011982563883066   14.8440780639648438
   Al   Si   O    C    H
     1    95   192    16    19
Selective dynamics
Direct
  0.6127756928940131  0.6919993211578621  0.5307948355374181
  0.3431906802909833  0.0507129555621544  0.9268196584051638
  0.3325928297939577  0.2954348920258787  0.9290240777930804
  0.8390395381571727  0.5497772809507769  0.9219416516588657
  0.8342622493026824  0.7729622665310483  0.9253644236174567
  0.7171685972544750  0.9008810586255038  0.1722135400607782
  0.7321927540906881  0.6563091851516267  0.1734998638913301
  0.2215529593658806  0.4014591522619306  0.1768190936973354
  0.2220379887306980  0.1766429513100684  0.1772352673149954
  0.7205107522277093  0.0513849139656513  0.1226130265763123
  0.7227066057749015  0.2757564915002975  0.1247055764944398
  0.2153588712657277  0.5513954898738233  0.1288092042249159
  0.2258941121200877  0.7969196779218881  0.1254780697610071
  0.3375951231398855  0.9004057838100799  0.3782969941218397
  0.3337241281780869  0.6794868091947220  0.3782328069914160
  0.8448698367015115  0.4017933479582913  0.3751930487071082
  0.8334209805727761  0.1561129891547764  0.3713513182870271
  0.7336507960734739  0.8936132640395520  0.3805315385035551
  0.7209060186624368  0.6548562936905582  0.3819815532561533
  0.2168202076214637  0.4117732337108941  0.3821209377242595
  0.2378386455359479  0.1652770249486094  0.3830980798565885
  0.3248484601928219  0.0598970604343505  0.1341883957678993
  0.3381892315941482  0.2933558267296691  0.1368378764809428
  0.8463966016300178  0.5414833486321300  0.1248288535849898
  0.8234674094827364  0.7876453764969323  0.1298399717300315
  0.3444999572556189  0.9111097499336641  0.1730203074545827
  0.3186547355404280  0.6683092877202442  0.1714230637635505
  0.8250470073862207  0.3940994634175414  0.1669896199368466
  0.8376101785690950  0.1581428731498687  0.1638941102592970
  0.7165282702717751  0.0403124735831080  0.9180578644880215
  0.7380478403861181  0.2865089684262441  0.9181077187913455
  0.2342914046302023  0.5613442647989622  0.9209929871745893
  0.2214906686545655  0.7946083402041384  0.9190516271056788
  0.1132919814177062  0.3544826284791796  0.0246746656284360
  0.1172771074168526  0.1996727703262986  0.0201646567549586
  0.6142336115093325  0.8494846598149337  0.0292848346165365
```

0.6104812997625292	0.6892308593252735	0.0364046317827278
0.9442933876622460	0.5998009423849537	0.2730947187595003
0.9400476140960649	0.7547135858810132	0.2709727804922914
0.4454237318828852	0.1003225704064377	0.2827686610854051
0.4493906474255123	0.2595816043432380	0.2871060355350983
0.9446870810972446	0.3519244006968983	0.0179610570841859
0.9480386108090552	0.1927128007269455	0.0141290447793882
0.4459428992254448	0.8534503084544193	0.0294717834859565
0.4421887299145812	0.6987540568481078	0.0355249395973943
0.1136551106236281	0.6023623348927990	0.2726933779827633
0.1101470594961034	0.7615547065235899	0.2691469285853112
0.6135221899290737	0.0976541968473277	0.2754705812231930
0.6179199719777626	0.2527461810837240	0.2804487126730227
0.3434067976681627	0.0501726612619325	0.4267655957803025
0.3325027929848572	0.2951365150251716	0.4290122970630340
0.8375108014218859	0.5511323457204904	0.4273961217906969
0.8332353215820892	0.7730567100646779	0.4266807318284870
0.7172567425866937	0.9030832297374967	0.6729366001828559
0.7325819291308093	0.6578184083324941	0.6811216848716570
0.2225618115861525	0.4009792173381754	0.6775314771967005
0.2220513395520012	0.1770247451901705	0.6771382033218374
0.7226808195261429	0.0506746877846230	0.6223648930332044
0.7226103633213086	0.2762078093320259	0.6230517790123384
0.2167163128430441	0.5506706138462354	0.6293198844581984
0.2258845753348047	0.7966183319883133	0.6270756976575893
0.3371140780097296	0.9011312083216017	0.8780757658081680
0.3376198834843855	0.6785902242297335	0.8795360286911895
0.8440138056957803	0.4007367978402342	0.8725100437753378
0.8323462256803231	0.1568844552027878	0.8714427292646081
0.7333936299366322	0.8917606810726626	0.8804621129052010
0.7192520453637268	0.6543656277840890	0.8886752559643895
0.2166566631159763	0.4123627429492957	0.8828289087385309
0.2375347188815197	0.1659217086800147	0.8832081689874864
0.3249642700490670	0.0599625404101081	0.6344176225340966
0.3382405068812203	0.2937217794285111	0.6370064793607099
0.8486614530905937	0.5412013740689936	0.6327232470460595
0.8232948194503578	0.7861367505059088	0.6338318833365235
0.3437808776389083	0.9113886762558351	0.6734180432948198
0.3197264140800748	0.6672562565113058	0.6725477646173338
0.8259749448651994	0.3919344524787467	0.6658341973741487
0.8377112250423340	0.1597073515555986	0.6635737741631107
0.7177933675742465	0.0397249156747647	0.4168532538124429
0.7398075629055494	0.2851602531505724	0.4174515708539173
0.2336213345781919	0.5605671522860205	0.4205992276825204
0.2187890885589035	0.7955891031897601	0.4189105173369550
0.1145272384589394	0.3529416700412374	0.5251882369463751
0.1176791424481123	0.1981675902955029	0.5205813719187324
0.6145732772427954	0.8549178286043123	0.5281443254464175
0.9480597981731467	0.5986399937605366	0.7722012343439414
0.9424050472496804	0.7522230916485934	0.7663753225522514
0.4453085924457764	0.1009252875974061	0.7827140290778478
0.4488173439860192	0.2597057185602402	0.7863542194256549
0.9462547976683250	0.3504878771806162	0.5174469225032754
0.9488286397156420	0.1918025464428572	0.5136400317435443
0.4461912142036850	0.8534746059073292	0.5303423048675660
0.4418652495017008	0.6985663490134556	0.5346896071589168
0.1150523160795444	0.6018511735526343	0.7712422722872392
0.1112792608294403	0.7598932785056089	0.7684211012331181
0.6143010197254273	0.0979979647870522	0.7736644823505111
0.6179854162931079	0.2534950235355676	0.7798560026426805
0.1605946483084944	0.3627188337311547	0.1168701741492268

0.1458453155489013	0.1915110968052406	0.1237815079752443
0.2813999507040379	0.0949981950873583	0.3751479352020960
0.6388593040411114	0.8877397684101401	0.1214109680044829
0.6555708301604425	0.6533442207749734	0.1171352913067264
0.7613665176151685	0.5924414326816192	0.4339617835655168
0.8965192120284723	0.5896213974334088	0.3639990134208627
0.9115031114543888	0.7635877927468945	0.3741882024074770
0.7802815846936686	0.8582363111187251	0.1197367582843391
0.4213947474035935	0.0610814244447700	0.3743232845999463
0.4065304261985407	0.2988107084835197	0.3675538137314535
0.2975314555416569	0.3583970990676185	0.1799156818247102
0.9218616630148248	0.3906257847205033	0.9254863007866003
0.9056035121850612	0.1536938385327151	0.9337299615766810
0.7963552323768606	0.0944772997130366	0.1186265552069083
0.3962272039809868	0.8608044384222960	0.9392216907265416
0.4155757115304732	0.6899788639826440	0.9312314007490707
0.2780341100335486	0.5961770628110283	0.1790713088552952
0.1378979952096548	0.5618919656164267	0.1817713790930182
0.1543543429698198	0.8010565633981743	0.1902559435761832
0.2608597172084394	0.8587895201356880	0.8722173026690945
0.6601011152669406	0.0910753405393680	0.1827671204553561
0.6452441747416089	0.2621405601500634	0.1767082439178631
0.7811466534686875	0.3573830850409617	0.9249592365938358
0.9165071421565503	0.5511965884424153	0.1920333811449615
0.8901446004529272	0.7977344684189348	0.2013728867620336
0.8064022692065003	0.8476440414710165	0.9002152353582428
0.3913880977171779	0.0830819926064592	0.1995612174805324
0.4149025932832370	0.2818439787720748	0.1905832074601234
0.2753697053602055	0.3535926843622406	0.9015384248000446
0.1445969728578059	0.4039062199132055	0.9467942769549806
0.1692077453822433	0.1570275069377678	0.9524453060726187
0.2501487184976303	0.1016500534880699	0.1550564101498271
0.6659499688832795	0.8702108789862868	0.9455087355797739
0.6403868806375391	0.6627903390602228	0.9394925675933763
0.7901926832720219	0.6033388109616759	0.1320243279863681
0.1647981214102003	0.5810807570620704	0.3572780424362914
0.1412226203366902	0.7838005436837767	0.3673587225256387
0.2864712313241831	0.8515374037589347	0.1553566824091761
0.6451790938622525	0.0489385528933496	0.3534481598042318
0.6715631055826391	0.2939149400345160	0.3477795162570270
0.7509241765292046	0.3509654846101812	0.1471441336640893
0.8914115993166687	0.3716585929643566	0.1008872301509718
0.9141478298767347	0.1717104967002723	0.1112372185853819
0.7736751681711024	0.1012789732125365	0.4015092835549111
0.4165748078554230	0.9025387154515149	0.1088566965590455
0.3882363135556999	0.6579559595486155	0.1036925206538882
0.3070361717941925	0.6030558818931099	0.3966462862941308
0.2857251319104535	0.2284477968925031	0.1449842611476971
0.7678000886698273	0.7212274800021691	0.9045714100882563
0.7936345423925083	0.2295682260693751	0.8809629780351517
0.2622530526603546	0.7228820938481124	0.1307684395242471
0.7668348813261787	0.7309166205172917	0.1664839140483929
0.2933565191513121	0.2230673028762630	0.9202587593554763
0.2769987822880586	0.7311628799615036	0.9164849266745109
0.7849823996173377	0.2235319274755238	0.1585820439012683
0.0281900346726896	0.3728780665221608	0.0449937889454015
0.0340742841048419	0.1717254259922649	0.0126129309043364
0.5304602806509333	0.8716933712330317	0.0056027973623937
0.5242630741201282	0.6683229693438463	0.0458380660484884
0.0294715953616996	0.5825472215925861	0.2960857342569105
0.0241256981710788	0.7812110908117393	0.2640183595712721

0.5282802822168713	0.0785998735402930	0.2554715450572199
0.5350254277212908	0.2826676110404533	0.2900283409192111
0.1210673348524419	0.2778199469207433	0.9900844597401582
0.6191131592243388	0.7695019843554562	0.0447016832601567
0.9360526989458161	0.6766951066170612	0.2400220224611595
0.4411535716872731	0.1801255914968517	0.3023112912003171
0.9390416736100629	0.2724369368987380	0.9991870671222747
0.4398670059576878	0.7767878287134575	0.0650440022868371
0.1222195007836749	0.6817991359970444	0.2543886073519446
0.6190198246510485	0.1747207239624930	0.3098740736831512
0.2063470316536135	0.1859185903489217	0.2843712801394628
0.1891647294387313	0.4100378923403851	0.2780202436888891
0.7145507414620418	0.6348902080850360	0.2757365158773077
0.7113134666961693	0.8777509693179492	0.2762994383743734
0.8564561417471355	0.7676807317339017	0.0315451048494165
0.8753055635854454	0.5371914442637498	0.0209990543350630
0.3588617383018780	0.3069532633513970	0.0323543878073820
0.3515492563006950	0.0738222568868814	0.0311141672939837
0.8581410465382699	0.1431623159582608	0.2680490736706929
0.8524943869315933	0.3811687293769795	0.2699592393141262
0.3476352215358787	0.6906950199955468	0.2704614949484778
0.3711878594025421	0.9101347605703726	0.2774687342097572
0.2002401324823881	0.8110980993588530	0.0227475720795880
0.2065995805760047	0.5729195762696355	0.0241355646846913
0.7073714124844117	0.2666850225534345	0.0175200391993795
0.6873293035279954	0.0431036141280920	0.0216311521790542
0.2410954929486393	0.4737453750425150	0.1334139237441538
0.7383681237076254	0.9794934729513319	0.1672756976397655
0.8211617810989744	0.4787889622417131	0.8736138952500178
0.3189566203339979	0.9729223905453667	0.9237281624621063
0.8045685631595696	0.4721513366978919	0.1513591715030870
0.3051008521881060	0.9818726238071410	0.1500269776650816
0.2556139024136703	0.4836007102214526	0.9037916519382174
0.7558968101183970	0.9691916715546302	0.8988014229367628
0.1624070656505740	0.3615102028121600	0.6169493901824598
0.1451355741668341	0.1903677295291095	0.6245454477075659
0.2806857069195097	0.0953097000788170	0.8759291012695715
0.6403480217963350	0.8966194593700092	0.6183841975762331
0.6670721722548382	0.6534703453138480	0.6114779111833963
0.7621010310728814	0.5902823612707803	0.9313965090225736
0.8965236848851165	0.5923197445027116	0.8611810973324403
0.9072339081506053	0.7560795626149274	0.8670690836399255
0.7803245224097706	0.8569282117897180	0.6260488754143354
0.4209784609209518	0.0609501641875178	0.8737633381501232
0.4047311611393969	0.2995437020874129	0.8649429997114307
0.2989548149397895	0.3586191030009728	0.6821007848152688
0.9234124541670332	0.3911407432859199	0.4264132143241336
0.9060489753060051	0.1516385317638306	0.4345221657351176
0.7963576450305470	0.0967735690855709	0.6170106210200537
0.3964660643183894	0.8605232918094468	0.4393236356837022
0.4097782228715977	0.6935516143940996	0.4308650103429820
0.2780530174359383	0.5954103005321384	0.6817947958146461
0.1384187889834530	0.5595800731424452	0.6814928016670907
0.1531426176779929	0.8017116523779350	0.6900235424456368
0.2611756842039003	0.8582121657124619	0.3722230875701810
0.6599236350946541	0.0893880079749496	0.6805099185404915
0.6456033562273584	0.2635352303726494	0.6761657184871003
0.7833379478288355	0.3555706623527136	0.4253010636666847
0.9229899128334845	0.5462325319571756	0.6942280306256188
0.8971729884949636	0.7972230545180723	0.6944853159429418
0.8035248965854775	0.8482847981442100	0.4093247468973523

0.3919687583514929	0.0833286238993995	0.6989448205927187
0.4152868665078824	0.2798533705473493	0.6883629404206028
0.2763988923261703	0.3530928029852577	0.3979233218062303
0.1454763039748163	0.4020983328740105	0.4467775515773482
0.1702524508307503	0.1558379261756073	0.4532156307222502
0.2507253048122363	0.1022586500172529	0.6553759836950070
0.6619088192764983	0.8818724257377322	0.4420375044504088
0.6412701553915072	0.6662594831038230	0.4233447104538922
0.7947763343517824	0.6015088868090404	0.6584953595813789
0.1686641771234843	0.5846527677139765	0.8550821270256338
0.1460796312382744	0.7774299506749219	0.8664040556646988
0.2852848580329612	0.8520030316498653	0.6576506857209574
0.6457847981134269	0.0491292802581944	0.8518484238087187
0.6694738200318469	0.2954940042650668	0.8489762069043465
0.7504740854769897	0.3519218029957433	0.6422340732249570
0.8924512376191106	0.3684409657029348	0.6005534041090563
0.9146756506165916	0.1732138967431860	0.6115619298011039
0.7751997391210604	0.0995536128445714	0.9015907218742385
0.4148770106555046	0.9029670191626590	0.6086341632689984
0.3859124649799041	0.6579046158370563	0.6015583495185240
0.3089578736079255	0.6035725116886951	0.9024874816607844
0.2843504090027003	0.2295800236756013	0.6430806354143429
0.7741024623855925	0.7202546224094394	0.3878337614938641
0.7964587427733641	0.2295344768587323	0.3799105644906526
0.2617891966781248	0.7227806612071522	0.6365225950374982
0.7731321305038247	0.7306663968798665	0.6844247932442163
0.2937874756318639	0.2227017563043297	0.4197180018775397
0.2685205080934635	0.7285933785451809	0.4120846012499260
0.7856244449776864	0.2253899597714751	0.6584164926378948
0.0294912978573326	0.3713370879346972	0.5458117315921419
0.0346279955496566	0.1699893651328758	0.5123270728095840
0.5292976004737098	0.8757881821394025	0.5074720924216576
0.5214906245831925	0.6668216951591162	0.5399114003667509
0.0322590893621134	0.5812473704007918	0.8004566461720926
0.0256651188363011	0.7809583508089945	0.7701263557640801
0.5285042221091929	0.0800642305847269	0.7557210105065371
0.5345689950772594	0.2814913410579623	0.7884525128913140
0.1219909117186092	0.2763189706719281	0.4904498768449000
0.6247647389629888	0.7772304633349855	0.5429062642440541
0.9429473827084591	0.6744505413219498	0.7335400312092626
0.4398136790117690	0.1803428879902237	0.8035380089899566
0.9411620964615707	0.2713706600025716	0.4955647431760301
0.4390529326114342	0.7770935479763116	0.5655731890208708
0.1209443546017719	0.6806802229507115	0.7479215081175443
0.6218927729330690	0.1750421185415380	0.8076244919871866
0.2069108221054140	0.1867376727736216	0.7842113302126930
0.1896133430560155	0.4097721561285166	0.7784388872407934
0.7027636178271895	0.6422106663436208	0.7831633355931483
0.7058089816000962	0.8813833332254651	0.7774594435889218
0.8472080626683890	0.7612047540136853	0.5336556149856531
0.8731739781312642	0.5429337402672393	0.5272177829541738
0.3576527993764599	0.3088720676283552	0.5321981265601705
0.3511835854032534	0.0733010584831384	0.5310131917585953
0.8570394223282640	0.1444981137020026	0.7679921354614773
0.8514990095713665	0.3759363700562819	0.7688298643311438
0.3506157667075488	0.6878540156670455	0.7719751038524063
0.3711975856190742	0.9108848471729506	0.7778825029707913
0.2012171280644903	0.8105909164358915	0.5238728640683639
0.2090999187619877	0.5730366792019495	0.5247284501557229
0.7074589764981588	0.2651151271418466	0.5159962453682323
0.6909630899337967	0.0390438187151010	0.5213047888284656

```
  0.2422791037043458  0.4728946538545605  0.6331039844020244
  0.7445814262846260  0.9807037968664404  0.6701296331279049
  0.8188564694040369  0.4786246632201950  0.3835320982455217
  0.3189830434819479  0.9725875015667321  0.4226911744036570
  0.8074951076144182  0.4703748763441764  0.6523613813559546
  0.3042247598200662  0.9823471257341682  0.6514267041732621
  0.2557964560753009  0.4827519926675989  0.4044417653540450
  0.7598752342209057  0.9712227195701287  0.3903532887584624
  0.4331541438577043  0.5226720834691565  0.2243890613701957
  0.4380014622853028  0.4588571068462831  0.2624855220860861
  0.4682130588616256  0.5773626833456182  0.2646934412358455
  0.4810117037972410  0.4491057595827074  0.3382982239298831
  0.5083069326959668  0.5689461747688876  0.3430189416046386
  0.5158673524857847  0.5042135119443720  0.3826700150968833
  0.5847643625381186  0.4933673929875234  0.4439202083523655
  0.5590746080479944  0.4917765895321083  0.5409339661654500
  0.4853122830392717  0.4725672602660067  0.5563828945161520
  0.6306931182004976  0.4325067496393383  0.4191286966606601
  0.5992967470930859  0.5179974528718584  0.6119229044034098
  0.5648028652441092  0.5305861957910217  0.6944723830628781
  0.4506689916771874  0.4859434810747290  0.6394022701450555
  0.4897170933700043  0.5168679643256168  0.7086238885566288
  0.4529116309312637  0.5321837625085006  0.7970432432792286
  0.4510605631643808  0.4712760616503861  0.8596543863759821
  0.4098012568542354  0.4169514204015578  0.2311327550313256
  0.4655180505679488  0.6261896477396134  0.2332408458592975
  0.4876485649847011  0.3991730547104475  0.3659988355109273
  0.5372109395207716  0.6107232454743399  0.3741226185831839
  0.4718853235244751  0.5020827651023866  0.4587626755241119
  0.4589832543489429  0.4342113125321519  0.5144725671846938
  0.6189610380243199  0.5382234463648350  0.4359616447354075
  0.5992890175024560  0.3861683261521570  0.4273308974133414
  0.6504454213023322  0.4352248963033941  0.3493283300329962
  0.6786978388054018  0.4300438162789598  0.4637369503453505
  0.4020248887872174  0.5299494161227856  0.1624276773224269
  0.5070014610514758  0.4541343280639455  0.8756157127675487
  0.4217113970603162  0.4296293397827517  0.8276286343879471
  0.4225642755590900  0.4833662577032613  0.9228828321974677
  0.4817192060879052  0.5737375770064932  0.8302592564703642
  0.3961836034705028  0.5485218652035753  0.7841216751502132
  0.6558392892798387  0.5338971841891010  0.6011068485828209
  0.5961824873797775  0.5551202585074209  0.7477644521967043
  0.3936937723077804  0.4705736309494575  0.6499094250769603
```

MOR-Al-T4-TS13

```
   Al     Si     O     C     H
   1.00000000000000
    18.1842269897460938   -0.0090872207656503   -0.0001607598969713
    -0.0105508984997869   20.0691013336181641    0.0015994716668501
    -0.0001797722798074    0.0011982563883066   14.8440780639648438
   Al   Si   O    C    H
     1    95   192    16    19
Selective dynamics
Direct
```

0.5916759967804024	0.7188676595688077	0.6393221616745212
0.3165240287780762	0.0763307660818142	0.0311786681413653
0.3043943941593264	0.3213408589363261	0.0344354659318924
0.8156754970550707	0.5796354413032760	0.0306398048996933
0.8137274980545044	0.8045173287392204	0.0295178424566991
0.6942583918571360	0.9305668473244171	0.2794214785099150
0.7088904380798340	0.6842997074127533	0.2797147929668518
0.1963993161916775	0.4270553588867323	0.2822555601596836
0.1964387744665194	0.2025919407606193	0.2813409268856151
0.6961029171943665	0.0801707729697270	0.2318574339151384
0.6961494684219360	0.3053183257579893	0.2333901524543848
0.1942045390605926	0.5767741203308309	0.2340789288282440
0.2047584801912308	0.8215732574463447	0.2318997979164168
0.3134694099426311	0.9256553053856353	0.4837791025638584
0.3143084645271301	0.7028218507767180	0.4843213558197185
0.8159844279289294	0.4316459000110978	0.4844765663147156
0.8043232560157998	0.1864417344331847	0.4802946448326231
0.7098284363746644	0.9221237897872933	0.4872536659240847
0.6980722546577650	0.6859708428383180	0.4876127541065388
0.1886180788278581	0.4383558332920318	0.4877077937126164
0.2105901986360551	0.1916616857051959	0.4877066016197304
0.2997978031635285	0.0864730551838918	0.2390816360712137
0.3122845590114595	0.3196630179882163	0.2422238439321558
0.8235725760459900	0.5691074728965765	0.2325709462165899
0.8002235293388483	0.8157403469086180	0.2354207634925893
0.3216571211814898	0.9377329349518364	0.2783811390400006
0.2991897761821746	0.6922768950462677	0.2778867781162371
0.7998639941215535	0.4216629564762344	0.2758941352367405
0.8115571737289599	0.1882360726595008	0.2726483941078294
0.6887030601501465	0.0687684267759352	0.0270021259784699
0.7105111479759220	0.3154807090759444	0.0270435642451053
0.2120382189750671	0.5867745280266036	0.0264661200344569
0.1977445632219315	0.8183169960975918	0.0241717305034405
0.0872838571667685	0.3808453083038500	0.1295046657323863
0.0906036719679839	0.2258754372596908	0.1248943731188813
0.5920396447181702	0.8787850737571724	0.1375276297330913
0.5893223881721499	0.7208060622215606	0.1436162590980559
0.9229997396469121	0.6270467042923201	0.3792805075645451
0.9182260036468510	0.7824634909630183	0.3759275972843289
0.4186195135116577	0.1289543807506604	0.3879700303077812
0.4216154515743256	0.2875213325023745	0.3921850323677216
0.9184737801551823	0.3792093694210222	0.1267077177762986
0.9212622046470644	0.2205391824245567	0.1227704584598566
0.4229129552841187	0.8814870715141567	0.1355445832014130
0.4194394946098328	0.7273516058922139	0.1406361460685740
0.0913702175021183	0.6294320225715851	0.3760732114315076
0.0876051262021086	0.7877217531204715	0.3730735480785436
0.5872555971145630	0.1267336159944619	0.3848268687725159
0.5905053615570068	0.2820375561714354	0.3897506892681216
0.3164398968219759	0.0760790854692485	0.5307588577270512
0.3046968281269073	0.3216486573219413	0.5342104434967045
0.8149058222770691	0.5808836221694947	0.5337201952934463
0.8104395866394272	0.8035562038421810	0.5298262834549120
0.6937742829322964	0.9308231472969513	0.7800409197807320
0.7083441019058228	0.6832802891731602	0.7863521575927734
0.1967937499284744	0.4274670481682095	0.7814964652061661
0.1964856088161469	0.2025623619556481	0.7814028263092258
0.6979282498359680	0.0788487866520910	0.7320075035095223
0.6960566043853760	0.3044641315937220	0.7334773540497156
0.1930864006280920	0.5766960978508242	0.7337234020233412
0.2040963172912598	0.8213111162186050	0.7319570779800683

0.3129464983940128	0.9269029498100552	0.9835491776466636
0.3150290250778198	0.7028827667236663	0.9847132563591011
0.8161410689354167	0.4307914674282232	0.9829798936844117
0.8047130107879822	0.1855749487876948	0.9808136224746712
0.7102921009063724	0.9202396869659878	0.9874394536018716
0.6986687183380127	0.6872820258140754	0.9936611056328164
0.1895308643579484	0.4383862912654878	0.9870159029961003
0.2106762230396313	0.1916720867157039	0.9880238175392367
0.2994964420795442	0.0857720896601720	0.7385905385017403
0.3125702738761902	0.3194026947021648	0.7417750358581742
0.8260031938552856	0.5685057640076023	0.7388290762901314
0.7980335354805141	0.8133081793785366	0.7374106049537874
0.3209294080734336	0.9370586872101101	0.7781996130943482
0.2979269921779633	0.6917681097984806	0.7784868478775049
0.8003747463226547	0.4198961555958021	0.7755082249641621
0.8120542168617249	0.1885166615247831	0.7732186913490563
0.6896387934684753	0.0691121071577079	0.5262901782989672
0.7107668519020264	0.3156798481941308	0.5270016193389893
0.2099188417196295	0.5876510739326647	0.5258449912071228
0.1972597837448121	0.8185396194458462	0.5237234234810045
0.0869977548718458	0.3806217312812947	0.6305675506591805
0.0904474183917046	0.2255778163671568	0.6254205703735535
0.5914016962051445	0.8810160756111416	0.6368234753608945
0.9256559610366853	0.6250750422478056	0.8787816166878011
0.9193761348724365	0.7796487212181238	0.8715424537658875
0.4185515940189362	0.1278944164514599	0.8875476717949257
0.4215255677700044	0.2865090966224837	0.8927836418152131
0.9189118742942814	0.3786466717720216	0.6261051893234261
0.9214839935302734	0.2205560952425117	0.6216403841972359
0.4240182340145135	0.8789786100388063	0.6353920102119629
0.4210115969181159	0.7225360870361663	0.6410201191902359
0.0927711054682733	0.6284437179565679	0.8771658539772232
0.0882404372096063	0.7865407466888884	0.8730797171592926
0.5876062512397768	0.1256649494171202	0.8830194473266871
0.5907191634178165	0.2810717821121308	0.8891466259956543
0.1359830796718628	0.3888565897941756	0.2206722199916892
0.1202899962663654	0.2174768745899368	0.2278732359409334
0.2532829940319061	0.1207358166575466	0.4811381399631626
0.6163047552108765	0.9210901260376518	0.2264844924211568
0.6329642534255981	0.6804959177971222	0.2221108376979880
0.7411604523658752	0.6255958080292015	0.5411565303802689
0.8756361007690431	0.6169575452804912	0.4705770611763144
0.8888645172119440	0.7918399572372783	0.4787112474441631
0.7568382620811462	0.8864106535911970	0.2282055169343964
0.3938845098018647	0.0875870361924228	0.4775116443634154
0.3762980103492803	0.3277691602707031	0.4693432748317824
0.2714596092701057	0.3831848800182510	0.2887051105499268
0.8934200406074824	0.4204123616218838	0.0370443388819699
0.8765595555305481	0.1804911643266792	0.0451938621699816
0.7707189321517944	0.1249186992645298	0.2260666340589581
0.3740672171115904	0.8895022273064289	0.0447641499340544
0.3914679288864136	0.7185322642326695	0.0369824692606931
0.2579828202724457	0.6202778816223314	0.2842728495597973
0.1167322695255280	0.5876336693763733	0.2871159911155705
0.1332730650901795	0.8282861113548821	0.2966006100177769
0.2382973283529286	0.8823568224907192	0.9786348342895516
0.6358360052108765	0.1183471828699146	0.2939095199108174
0.6194838285446167	0.2917642295360678	0.2867660522460938
0.7528930306434632	0.3867470026016405	0.0333502851426603
0.8939998745918281	0.5783608555793990	0.2991057336330418
0.8680649399757385	0.8245387077331990	0.3056153357029074

0.7861428856849847	0.8797975182533557	0.0068068429827692
0.3661730885505676	0.1120882630348244	0.3030799329280953
0.3899961411953080	0.3070781826973129	0.2931351959705357
0.2457486391067506	0.3776302635669936	0.0048101060092450
0.1170074567198757	0.4303387701511564	0.0505558736622343
0.1412256360054017	0.1830815821886146	0.0558158941566956
0.2243886590003967	0.1274775117635805	0.2591190636157992
0.6438885331153871	0.8955796957016326	0.0521361976861967
0.6215870380401634	0.7009684443473839	0.0453402139246464
0.7670845985412598	0.6305150985718113	0.2409488856792542
0.1437202692031865	0.6123375296592882	0.4613819420337769
0.1199354156851769	0.8052093386650120	0.4724612832069510
0.2648733854293822	0.8777611255646224	0.2576363980770196
0.6160266399383545	0.0778178945183796	0.4645242989063367
0.6413787007331848	0.3246339559555139	0.4590826928615662
0.7245388031005859	0.3806163668632694	0.2551840245723724
0.8667618632316593	0.3968310356140304	0.2121915668249187
0.8889558911323554	0.2017147392034620	0.2216148972511367
0.7441678643226624	0.1314563453197492	0.5090942978859171
0.3941972553730016	0.9314462542533900	0.2144585251808231
0.3673840761184737	0.6843623518944058	0.2082292586565090
0.2863213419914283	0.6269698739052159	0.5051482915878315
0.2603474855422973	0.2541723549366088	0.2496116459369661
0.7484232187271288	0.7540711164474834	0.0005702484631911
0.7667430639267012	0.2586275935173205	0.9907824397087105
0.2414066493511204	0.7480232119560559	0.2424037158489299
0.7441933751106328	0.7584780454635880	0.2716992795467377
0.2665322422981262	0.2481894940138020	0.0268036052584655
0.2508376538753511	0.7534399628639594	0.0178912561386826
0.7592616677284241	0.2536973357200736	0.2662682831287469
0.0024974711705000	0.3994943201542059	0.1519704759120978
0.0069493446499110	0.1985651552677269	0.1192655116319683
0.5079067349433898	0.8984288573265361	0.1119721308350599
0.5030452609062195	0.7013778686523658	0.1491185575723648
0.0079147554934027	0.6082916259765795	0.4014739394188019
0.0021069301292301	0.8097057342529568	0.3695048093795868
0.5022645592689515	0.1087654307484673	0.3619486391544346
0.5071220397949215	0.3102655410766764	0.3969862163066868
0.0941971540451071	0.3041036128997910	0.0949326828122162
0.5990836024284366	0.8001843690872524	0.1613800525665283
0.9153963923454252	0.7040269374847750	0.3464474081993107
0.4129382669925775	0.2081833034753913	0.4103576838970351
0.9126571416854861	0.3000974953174697	0.1048645526170753
0.4141106307506646	0.8052008152008157	0.1712889969348958
0.0965761616826057	0.7083415389061222	0.3534805178642408
0.5938539505004883	0.2038639336824531	0.4187193214893515
0.1802264451980591	0.2118289172649451	0.3883516788482670
0.1625773757696163	0.4363338649273190	0.3829630911350364
0.6903258562088016	0.6639409065246752	0.3823067545890812
0.6859762668609789	0.9073115587234523	0.3832826912403208
0.8321877717971802	0.7966525554657322	0.1363437026739120
0.8524787425994873	0.5656536817550993	0.1288719177246136
0.3302153944969178	0.3348962664604370	0.1373625844717028
0.3258513808250429	0.0989387482404733	0.1354845464229586
0.8302016854286390	0.1735322326421828	0.3774052262306217
0.8250398635864400	0.4095476269721989	0.3799445927143243
0.3301060497760774	0.7119690775871504	0.3771399557590605
0.3475955128669740	0.9348628520966118	0.3831809461116935
0.1782257407903672	0.8326662182808193	0.1288233101367997
0.1852206587791481	0.5992953777313567	0.1297487467527411
0.6797003746032716	0.2960546314716340	0.1265175491571428

0.6616911292076281	0.0715158209204706	0.1314785033464433
0.2174830138683354	0.4986438155174496	0.2381209731101999
0.7176271677017382	0.0087528079748156	0.2756333053112129
0.7921332716941833	0.5088562965393523	0.9846828579903041
0.2920728921890260	0.9985928535461697	0.0276510119438175
0.7814291119575720	0.4998804032802749	0.2582437992096047
0.2814172208309174	0.0084948474541312	0.2578139007091607
0.2306461781263352	0.5083625912666656	0.0100290747359397
0.7288898825645467	0.9983143806457977	0.0065640248358251
0.1358349174261093	0.3885779380798595	0.7214998006820895
0.1198774352669723	0.2169457376003377	0.7285170555114754
0.2535262703895569	0.1208712905645439	0.9812495112419495
0.6166585087776262	0.9245579242706753	0.7254962325096138
0.6416582465171992	0.6737455129623817	0.7182861566543838
0.7427718639373817	0.6256545186043125	0.0415372923016560
0.8751626610755920	0.6178164482117035	0.9681665301322945
0.8881614804267940	0.7876586318016388	0.9738177657127564
0.7563846707344075	0.8848724961281167	0.7319109439850149
0.3939984440803528	0.0867314338684111	0.9774886369705571
0.3765304088592529	0.3261323273182029	0.9705184102058652
0.2721590101718985	0.3838829100132162	0.7864809632301338
0.8936331868171724	0.4209241271019117	0.5375127196311955
0.8762959241867067	0.1805952787399360	0.5444222092628479
0.7715591192245487	0.1250769793987280	0.7266018986702177
0.3725150525570017	0.8864848613739328	0.5460721254348763
0.3906564414501189	0.7160419821739570	0.5373625755310228
0.2560991942882541	0.6200127601623991	0.7855784296989657
0.1155977845191956	0.5863063335418720	0.7871742248535505
0.1320402622222950	0.8275276422500613	0.7955774664879044
0.2378759831190154	0.8823189139366684	0.4776083230972455
0.6358373165130615	0.1167183965444606	0.7919691801071350
0.6187826395034997	0.2914802730083482	0.7856737375259599
0.7525200843811036	0.3872512280941177	0.5341698527336290
0.9002416133880610	0.5729128122330047	0.8004858493805148
0.8710376620292666	0.8221797347069255	0.8000932335853584
0.7830828428268435	0.8795724511146553	0.5120264887809923
0.3662008643150332	0.1103880032897063	0.8027896285057267
0.3896493315696815	0.3068080544471828	0.7939599752426155
0.2464738190174110	0.3792205154896011	0.5078524351120165
0.1164499968290329	0.4302347898483417	0.5515730977058634
0.1418503373861313	0.1834291666746197	0.5565484762191780
0.2246338129043579	0.1274253576993998	0.7595670819282717
0.6408405303955257	0.9038620591163637	0.5505990982055672
0.6191459298133850	0.6968259811401595	0.5310242176056107
0.7729359865188601	0.6292235255241729	0.7650006413459979
0.1467182785272620	0.6113473176956347	0.9606509804725841
0.1209927052259449	0.8045196533203582	0.9720663428306587
0.2631438076496124	0.8774867057800293	0.7601914405822762
0.6165384054183962	0.0767352059483571	0.9627807140350706
0.6419523358345028	0.3236336410045747	0.9578237533569519
0.7243275046348572	0.3800263106823136	0.7540296316147074
0.8663530945778081	0.3937574326992208	0.7114900350570686
0.8885640501976045	0.2034337520599433	0.7208065390587058
0.7459404468536377	0.1286816298961684	0.0075722392648461
0.3930108547210693	0.9272505044937553	0.7152109146118406
0.3650096058845521	0.6824500560760903	0.7079596519470455
0.2877919673919678	0.6273140907287768	0.0071620596572759
0.2596308588981628	0.2545606195926780	0.7483569979667880
0.7496330738067627	0.7525787353515853	0.4899961352348448
0.7677035927772522	0.2602421939373016	0.4887181222438953
0.2407982945442201	0.7478651404381138	0.7427537441254006

0.7449841499328613	0.7576612234115936	0.7826817035675057
0.2666187286376963	0.2488161176443235	0.5246738195419481
0.2497973442077637	0.7532225251198150	0.5168280005455017
0.7586501240730642	0.2533123195171483	0.7683939933776863
0.0023342708591372	0.3996552228927890	0.6529779434204286
0.0069906474091113	0.1977411955595121	0.6190952062606995
0.5068459510803419	0.9022374153137664	0.6122180819511421
0.5006789336867818	0.6856761127131573	0.6501372633677404
0.0099619440734389	0.6078257560730208	0.9062276482582309
0.0027803548146040	0.8081307411194119	0.8700513243675499
0.5023007392883301	0.1080508902669006	0.8613359928131111
0.5071616172790652	0.3084486424923003	0.8969568610191544
0.0936596766114235	0.3039172291755846	0.5959014296531918
0.5987624526024085	0.8034600615501861	0.6573437452316552
0.9190154075622554	0.7012103199959258	0.8418523073196619
0.4122282564640083	0.2070491462946052	0.9095805883407601
0.9144519567489628	0.2999462783336827	0.6015349030494873
0.4207910299301191	0.8014077544212843	0.6688117980957236
0.0981172695755966	0.7072225213051178	0.8536859750747890
0.5949754118919373	0.2026913166046232	0.9170320034027298
0.1811978220939636	0.2124142646789619	0.8884730339050509
0.1637013256549836	0.4380899369716796	0.8820995688438716
0.6811594963073733	0.6696310043335307	0.8897314071655281
0.6830598115921021	0.9086804986000087	0.8844633698463656
0.8233047723770338	0.7907908558845860	0.6367716193199165
0.8502528667450081	0.5704078674316740	0.6333273649215706
0.3321840763092113	0.3336387276649645	0.6369271278381445
0.3252238333225250	0.0988175496459057	0.6350555419922096
0.8315665125846864	0.1740781962871649	0.8778260946274037
0.8255472183227539	0.4067011475563075	0.8792225122452006
0.3297739923000336	0.7114943265915201	0.8772886991501071
0.3466666638851203	0.9364640712738491	0.8830956816673287
0.1787191480398179	0.8328946232795986	0.6285693049430855
0.1845712512731565	0.6003857254982222	0.6296509504318420
0.6807704567909241	0.2947598695755173	0.6263021826744087
0.6648178100585940	0.0681967660784737	0.6314377188682773
0.2173260450363159	0.4986574649811020	0.7353485822677620
0.7208498716354370	0.0084194717928769	0.7777757048607115
0.7913382649421692	0.5092471837997482	0.4901700019836517
0.2933107018470765	0.9980145096779141	0.5266988277435473
0.7832593917846813	0.4982778728008541	0.7580885291099815
0.2809889316558838	0.0076758069917563	0.7556099295616376
0.2287918031215669	0.5091916918754578	0.5091138482093811
0.7316062450408938	0.0005800136714242	0.4995352029800614
0.4318593442440045	0.5292149782181014	0.9986816048622321
0.4148197472095490	0.4865023195743803	0.9268936514854698
0.4837615787982941	0.5795181989669845	0.9831351041794091
0.4523568153381348	0.4907348155975571	0.8450427651405551
0.5201110243797460	0.5849121212959655	0.9006866216659847
0.5071778893470764	0.5391349196434022	0.8311523199081623
0.5613357871734095	0.5362815974035710	0.7541429121378985
0.5324690787620379	0.5492754718198305	0.6581390126088186
0.4574429684073605	0.5346733310117773	0.6376817943062683
0.5864433524152102	0.5425427874957868	0.5868357425317049
0.4368658959865655	0.5215689539909552	0.5498926043510437
0.5668231844902208	0.5240505337715426	0.4998857975006107
0.4910647571087033	0.5179188251495541	0.4829384386539513
0.4000107347965242	0.5188273191452362	0.0914344489574455
0.5967232238902564	0.4667171094362947	0.7535355587069471
0.4522410333156735	0.4788786172867092	0.1524385809898378
0.4280925095081330	0.4721542298794058	0.2198701649904297

```
  0.4628714621067050  0.4292336106300583  0.1239686831831941
  0.5053477883338954  0.5046121478080977  0.1599154025316240
  0.6196550262347631  0.4555810007782254  0.8202117498427620
  0.5558724186682806  0.4280757165943371  0.7373405085840465
  0.6412383347428515  0.4643010171277411  0.7039969131607954
  0.3472669422626495  0.4924229681492081  0.0862344354391099
  0.3886619508266450  0.5674160718918017  0.1228905692696598
  0.3735065758228334  0.4477318525314602  0.9361471533775575
  0.4974942803382877  0.6140570640564216  0.0371828228235255
  0.4407338798046165  0.4536480009556095  0.7930698990822079
  0.5618368983268738  0.6232970952988011  0.8913593888282975
  0.5129436266819648  0.6142635828179475  0.6529053537468789
  0.4170376758097708  0.5380430867450218  0.6917642173545895
  0.6436980394258425  0.5525001268849619  0.6040545577123428
  0.3789100646972666  0.5160320997238494  0.5329662561416810
  0.6067289113998603  0.5186027884483565  0.4456008970737578
  0.4730966091156009  0.5103800892830065  0.4141552150249548
  0.6044226839197671  0.5732124050381305  0.7671236339505285
```

MOR-Al-T4-H+I$_{dyaril}$

```
   Al     Si     O  C   H
   1.00000000000000
    18.1842269897460938   -0.0090872207656503   -0.0001607598969713
    -0.0105508984997869   20.0691013336181641    0.0015994716668501
    -0.0001797722798074    0.0011982563883066   14.8440780639648438
   Al   Si   O    C    H
     1    95   192    16    19
Direct
  0.5852888772836047  0.7127238350901574  0.6500705090194038
  0.3133630200964758  0.0705909679403327  0.0358792374805912
  0.3022904118898014  0.3160252556077798  0.0388540788494177
  0.8120704775024704  0.5712618208977643  0.0328705996382513
  0.8074995984803494  0.7954647531173427  0.0346916623999750
  0.6914390658367093  0.9231933644607289  0.2836188296406346
  0.7071899168112269  0.6789319162417722  0.2831159176887227
  0.1929444223367576  0.4210034698995304  0.2868132273005667
  0.1931103682575364  0.1966885241316069  0.2856282866525600
  0.6921565014578213  0.0729978698731318  0.2343292205281783
  0.6929973168046319  0.2977801444244784  0.2357509391036861
  0.1898790420732815  0.5705683670752519  0.2383157741228645
  0.2000859946597152  0.8144549614373598  0.2372477086566799
  0.3093554695969411  0.9213157340122704  0.4898966351349676
  0.3087092041840785  0.6977817456410446  0.4879082085987282
  0.8124311944156639  0.4245310863660894  0.4883870009942223
  0.8017398536109354  0.1801209691058555  0.4836086608210016
  0.7075078407304990  0.9183243583653738  0.4917310298057284
  0.6985218475994972  0.6776559755263647  0.4924057301882471
  0.1870101729229744  0.4319390302268558  0.4921510172688667
  0.2077139175743732  0.1853791894584458  0.4921107198843263
  0.2952605008064365  0.0793604964326440  0.2436334843237055
  0.3091251166705997  0.3133387641273785  0.2463429579825589
  0.8189867489351735  0.5616882379135570  0.2358179941483507
  0.7980806966360443  0.8097886810361715  0.2395390381993341
  0.3156799588649496  0.9312256320949048  0.2842016410918303
  0.2946362646695114  0.6854086800844091  0.2821157334233435
  0.7948409123699798  0.4154180456035083  0.2791804061620662
  0.8082546962982222  0.1807206671733126  0.2754767777935455
  0.6862489568968697  0.0621723823648543  0.0294676065746284
  0.707710129266485  0.3090544349875644  0.0295035867646143
```

0.2087715622742320	0.5812311399850809	0.0310250123862475
0.1957433633588521	0.8122773716902435	0.0293890431161494
0.0836445039078062	0.3748549280143323	0.1352424533692928
0.0874501701720202	0.2199522076941191	0.1293637125837540
0.5876374517155608	0.8714527145386296	0.1420100436787469
0.5847698382204634	0.7118117306800220	0.1481181543228890
0.9185014618036333	0.6191543522872405	0.3827223683698531
0.9131484979887927	0.7738031873192287	0.3801858174952097
0.4152406767704258	0.1209662692089976	0.3924163493609640
0.4189094467706975	0.2800789626711997	0.3964493716253982
0.9151848769952898	0.3726333416452222	0.1315386305906111
0.9182907121857404	0.2135007181821594	0.1266083822718808
0.4182594713479957	0.8741586010724250	0.1414289038903598
0.4157414832231026	0.7193870580673107	0.1462203672791555
0.0873489850815193	0.6211131286726549	0.3823679591256470
0.0834485091288387	0.7802276474977204	0.3802985409777088
0.5836071430046890	0.1187087146460186	0.3874765586715263
0.5877636705438414	0.2737121769243925	0.3918546765320305
0.3137242167513180	0.0709571727529394	0.5370052502397819
0.3020370874458244	0.3148727763392890	0.5386100848667745
0.8133225758359911	0.5732920143748436	0.5401107733292331
0.8040385303978782	0.7977067505422900	0.5379611524901549
0.6892472125801089	0.9230860055451020	0.7850552522126623
0.7069083102643493	0.6763668513745100	0.7918537249339865
0.1929111372792033	0.4203752731356717	0.7872266305508011
0.1933258452256818	0.1966662615439073	0.7860940398059120
0.6934800649545317	0.0717173928447811	0.7354039598375613
0.6928342349403075	0.2975267228736320	0.7355687361418197
0.1890269162428532	0.5699963130897718	0.7397862728926755
0.2004798082989533	0.8160260476766159	0.7382791645723183
0.3101884276513026	0.9207656270852901	0.9877753555266382
0.3126161026211397	0.6963560200303048	0.9893692115658639
0.8147900161087236	0.4222420044946670	0.9853650066290102
0.8017155751121307	0.1793241876548995	0.9834952493712897
0.7056536831931746	0.9138451119656870	0.9920773666530137
0.6926693596590310	0.6768132415536552	0.9984161621020063
0.1858194415888035	0.4321108901432693	0.9923966611276107
0.2081247131830205	0.1863985390097101	0.9923421996713582
0.2965437529585556	0.0809152890312841	0.7443210859356207
0.3091048091251841	0.3132298212026932	0.7464958523166707
0.8240866610770310	0.5610462868939049	0.7456517624095423
0.7959691798313997	0.8069531309969135	0.7444670754059319
0.3181441023597211	0.9317757705078675	0.7827165806252582
0.2938114179942240	0.6855982643264817	0.7834778122382069
0.7982442396398737	0.4115556925483247	0.7789719925511041
0.8083513940024756	0.1810854470931809	0.7758733194358900
0.6870777648364262	0.0634279664164078	0.5295843534948614
0.7083172508116005	0.3086555668814487	0.5294919650082849
0.2060039634491412	0.5800537105925920	0.5319186017428046
0.1929245359287559	0.8141385683274132	0.5296946342525383
0.0845789058326688	0.3736109697817657	0.6345736872665393
0.0876808856579354	0.2185345999259674	0.6296412263651388
0.5892117567891181	0.8745546625650975	0.6380412583014164
0.9228083621482787	0.6191677694843443	0.8845125177006785
0.9163423652203816	0.7730310108484026	0.8770859697883522
0.4154059632820557	0.1229918536194626	0.8933906984442428
0.4185328785775860	0.2813255363541323	0.8968322408750875
0.9165122992776560	0.3708457064771476	0.6290258632821690
0.9189915655013735	0.2127372542924472	0.6245920067015724
0.4191928327561558	0.8731893202795039	0.6402610097519640
0.4140935573657706	0.7184176174408914	0.6448823692577491

0.0893960491866257	0.6230648505603296	0.8821761521819226
0.0852561236078290	0.7806078657690773	0.8791514683047393
0.5844842691777327	0.1198631002726769	0.8862583889159136
0.5879265636869687	0.2752052210902230	0.8917729947400771
0.1317086920808978	0.3823835759634123	0.2268648046202591
0.1165895028049424	0.2112075167324797	0.2325874533022833
0.2508479456912216	0.1146665317352148	0.4853476044159127
0.6129585740289334	0.9101476795444140	0.2335384116869199
0.6297085587296006	0.6753292946569472	0.2294481757377565
0.7390305841627224	0.6188250925489022	0.5474182931523912
0.8713950423230655	0.6109710868381725	0.4749910882779497
0.8777506436817106	0.7804027091270116	0.4807202988262703
0.7547667378597412	0.8802657077651053	0.2317417603947959
0.3913774486744903	0.0813135208684295	0.4837640085404157
0.3747016293173702	0.3189938928057470	0.4755215127110629
0.2678580058652847	0.3769731244997195	0.2918921169761179
0.8918052206559359	0.4120596217463806	0.0398219723542266
0.8738118583676947	0.1748240624351064	0.0475591216000554
0.7666630676222491	0.1177268552793645	0.2289890601612852
0.3704543139338781	0.8827191916977793	0.0499178719576935
0.3896083286497341	0.7109121108572809	0.0417804131442354
0.2533155671893289	0.6136571281663122	0.2897933874164285
0.1123355662958515	0.5817123767752935	0.2912582137842287
0.1270137308608709	0.8188463216256880	0.3000547360068648
0.2353257176029030	0.8763758277888145	0.9826257221028297
0.6312600450267387	0.1106861807674999	0.2957276294245974
0.6165433541589374	0.2825369796980443	0.2885702700136077
0.7505594087721728	0.3800946278188577	0.0369457133295953
0.8887915573408173	0.5699255408408922	0.3036624841576674
0.8663466985674743	0.8182538097011419	0.3090364834493156
0.7794059576514865	0.8705699212482816	0.0121032338297868
0.3619358339164240	0.1024737131659665	0.3089206429342266
0.3860413194365492	0.3004813651698379	0.2984900884703906
0.2431056359648639	0.3725404390039952	0.0117791140596929
0.1136963328532952	0.4245160303782427	0.0567731759886900
0.1389818332250961	0.1778989635995144	0.0605969910036598
0.2206938838748795	0.1213161733414672	0.2641474922411807
0.6384854571137506	0.8926149756718570	0.0576294966516909
0.6150667708567321	0.6859045720511538	0.0514383739492981
0.7645264420973163	0.6250171531810932	0.2444273693829196
0.1376975581546238	0.6007144674092182	0.4677080348885317
0.1155470338841163	0.8018725315404085	0.4780055328866355
0.2589114902726453	0.8704402835947999	0.2679149424012707
0.6139200043555623	0.0700281213636606	0.4666770516743283
0.6400286922498434	0.3158828699426988	0.4598856145801381
0.7204685970822199	0.3731902068018895	0.2584163317154829
0.8622277249753877	0.3921340744103455	0.2151495257417982
0.8860898285641254	0.1926581574172777	0.2248427146558733
0.7398863853523192	0.1272208803345303	0.5145670286729873
0.3881967190950525	0.9230407498145111	0.2205802733936333
0.3632245953260451	0.6763687622865090	0.2130565844266119
0.2792990004097712	0.6227646932260070	0.5090966450534601
0.2567403214832359	0.2479820738853314	0.2525014213832218
0.7415075422108719	0.7439460535719410	0.0113981881400942
0.7637930497550663	0.2523754981027558	0.9924963272945755
0.2374189221395722	0.7410087844344740	0.2448475514905330
0.7418558738068446	0.7532163775312458	0.2785555704737099
0.2644278099717614	0.2428910770110408	0.0299634564331491
0.2498693127195458	0.7480091905634132	0.0239292263276267
0.7572016228984858	0.2469022716863196	0.2679868191185434
0.9987901469764225	0.3933850801847006	0.1577496251630049

0.0040636120708282	0.1919841531174663	0.1224664325981130
0.5033568549403281	0.8919857366979705	0.1196409402146897
0.4988947747039466	0.6917862145165439	0.1575319389567354
0.0030879383745980	0.6005571939980572	0.4050492691671024
0.9971329617403678	0.8004651659786494	0.3793232297861698
0.4986449384584542	0.0999472697192514	0.3658816497878108
0.5046681501147744	0.3021585736039433	0.3994711882018417
0.0903264172582481	0.2982811516853106	0.0999879411172188
0.5953796218620369	0.7917375411617176	0.1582976871457450
0.9102211652888532	0.6958973083204192	0.3484642433993713
0.4102709316069911	0.2004371845965154	0.4124363364506914
0.9091318280182998	0.2932851564431961	0.1119634227036141
0.4110314227233769	0.7973912970073384	0.1760991807250221
0.0938092302494923	0.7006424474977918	0.3644652617522239
0.5904654239683160	0.1956479977018249	0.4215411621231703
0.1776946007113541	0.2061621397477535	0.3927849028571932
0.1601424390991772	0.4316724481760818	0.3876563996047481
0.6899753316198173	0.6591433730180717	0.3875195596519397
0.6855078859711596	0.9005675096664371	0.3880688471982649
0.8288130920069956	0.7888355977049482	0.1409813195780825
0.8477431314618002	0.5582124263063024	0.1321900309606034
0.3287569901516902	0.3281528869498142	0.1418267956992582
0.3220919899544621	0.0926378813474746	0.1405131629423203
0.8257858504523351	0.1656513184790016	0.3804296445817644
0.8205673475349933	0.4040162514267630	0.3831968508283669
0.3252441329838731	0.7064813694740929	0.3807616251882170
0.3420645078081015	0.9315647190150174	0.3887966255132662
0.1760580982656744	0.8278309126360864	0.1337534761584120
0.1815513344636514	0.5937670065140641	0.1341968018386002
0.6768480921446848	0.2890164632970120	0.1287099682573989
0.6585255277190250	0.0635836382971646	0.1337089439712829
0.2130090382497670	0.4924205079464289	0.2413475659986448
0.7132338495460184	0.0014981463523934	0.2783455473381387
0.7928686243569176	0.5006138316803770	0.9846552153899850
0.2894810059226282	0.9926595873612408	0.0313441441601375
0.7751394244122863	0.4935600367854970	0.2616087295617084
0.2753400604466409	0.0012680868195929	0.2597614696512786
0.2258659818621562	0.5029014404571209	0.0131676465244000
0.7268585550635338	0.9917667778616028	0.0082651561525475
0.1322403166392714	0.3817273293082082	0.7262927435219875
0.1166988491489360	0.2103723536251779	0.7328974853646911
0.2505618025763465	0.1153031554555748	0.9859193053123153
0.6123294107216336	0.9139466328941215	0.7307680423199071
0.6433082474050728	0.6691152279958503	0.7188478291260705
0.7362549921985957	0.6135086115215910	0.0421140234726119
0.8708162105529429	0.6128806703963535	0.9729972264371360
0.8812105594276912	0.7789906712202822	0.9776621767269674
0.7531021904039714	0.8778132582030795	0.7380827641956573
0.3911544601279081	0.0813976523045970	0.9830841201480923
0.3739720987621470	0.3217312603395923	0.9743096689814492
0.2678076204897018	0.3764361773313883	0.7927424998417627
0.8903043407888471	0.4128225966553834	0.5404399987821421
0.8743923715140635	0.1730529860931708	0.5464024665079136
0.7677268500717919	0.1170652653369684	0.7306899352316165
0.3698123178761021	0.8818235913242199	0.5494873872183516
0.3845889090403359	0.7123476160007752	0.5408078321329406
0.2510327997488127	0.6143997357178267	0.7916106690378141
0.1112164858009714	0.5798687657669798	0.7928842830001335
0.1285481303937704	0.8230051383793900	0.8024360261823016
0.2333875185779604	0.8785528081110596	0.4851421027183042
0.6311990409963939	0.1102734818891539	0.7940231572911697

0.6156484160692847	0.2849574528659746	0.7880108104041215
0.7484142558841620	0.3809057105947951	0.5376201352290323
0.8985793743195657	0.5660994594469954	0.8066860480491754
0.8697013352069064	0.8169397043998935	0.8052358278384206
0.7788085909438585	0.8739125663890682	0.5199855279455748
0.3628628533985461	0.1059080373435620	0.8086927959783646
0.3862588003086089	0.2998347678035090	0.7978309858786061
0.2445642287622821	0.3720539891761225	0.5086943539058520
0.1150092110516787	0.4230552511135194	0.5561003501383067
0.1388150247467275	0.1763236769632172	0.5605507459190793
0.2209450177832879	0.1211918769267129	0.7655410175257947
0.6366936182076441	0.9044749447721072	0.5541523710245353
0.6120106425354777	0.6823107317823685	0.5332536958612021
0.7710810746931905	0.6216422823626589	0.7731897240205383
0.1442322435496378	0.6077147112308733	0.9653542808667273
0.1190217194277932	0.7969109232961314	0.9780921421746182
0.2600718209996972	0.8724754461781105	0.7642275372252677
0.6144246108973624	0.0703825086974688	0.9648877057016870
0.6394142248589840	0.3180320756438369	0.9600990300080400
0.7216656025251048	0.3729214372185600	0.7562224559940600
0.8639459419043756	0.3856014658922962	0.7144484685419323
0.8847748488180122	0.1953276086710888	0.7229900504969031
0.7432714557523860	0.1225051705325162	0.0118857106388721
0.3902138734155812	0.9234278734125644	0.7189810840209607
0.3589238434989845	0.6767676634047072	0.7107388978678094
0.2852403660077228	0.6210154488456582	0.0126788566568685
0.2567420647215408	0.2477906355286004	0.7519511921621570
0.7355838236424561	0.7493761012518755	0.5071981137254946
0.7675441993114763	0.2548320910924110	0.4919017384712590
0.2363607827918365	0.7424976234732009	0.7503273349913313
0.7447079526680400	0.7505317652175966	0.7909142632598646
0.2634507179267185	0.2421653828713589	0.5302993686344494
0.2445690636803076	0.7488272153878868	0.5195764048197433
0.7551311110858199	0.2459121177791831	0.7705155173169175
0.9996353978296436	0.3928735174861416	0.6552924143011857
0.0044438340677242	0.1899918315102544	0.6232644301671727
0.5034629793021014	0.8919383830783443	0.6167764178028656
0.4951531446364510	0.6888621048042937	0.6512207255244997
0.0069062260305851	0.6027278326604298	0.9135835873105538
0.9995032936727601	0.8019868390908222	0.8793178687697507
0.4988931522990672	0.1026999432079613	0.8664686967455857
0.5044627463072180	0.3020247684796866	0.9005506663681593
0.0908753502781089	0.2968428411772998	0.5999533802093341
0.6040285056015590	0.7963903540758960	0.6480382249687864
0.9165316734750725	0.6949584668041129	0.8457034166498366
0.4092923698851600	0.2019620103895001	0.9159743355385792
0.9126049138026899	0.2921558050208405	0.6046279247178635
0.4104802625774969	0.7969147141976782	0.6757090025616576
0.0940544839070326	0.7016417233951447	0.8568283969691280
0.5933169525733208	0.1967937542258958	0.9200321761235378
0.1779786037465872	0.2070534844542159	0.8930483051856347
0.1591574515463203	0.4299468293768530	0.8878415108468820
0.6750733956151468	0.6642182591844176	0.8932917928464688
0.6781081300046523	0.9012322059522088	0.8894283468030417
0.8212670107368856	0.7845384268909354	0.6434402324929780
0.8482110689462238	0.5648299967664713	0.6397684310331088
0.3275388668377810	0.3284893539549375	0.6416128818254411
0.3221729295163043	0.0948638700871898	0.6408674727772792
0.8281254328162866	0.1669996692442663	0.8805437476021396
0.8233212776902588	0.3965608590051270	0.8822421111024316
0.3269509737828253	0.7049630445697156	0.8820427456455331

```
  0.3444620679604770  0.9299379175350160  0.8875278254679435
  0.1744121204649883  0.8268093989974045  0.6349073627215039
  0.1804274523538230  0.5929219274903217  0.6354970937078988
  0.6780756438423307  0.2871487126407881  0.6284093862349082
  0.6614291707720713  0.0604666472591434  0.6343337808551723
  0.2132019583556391  0.4920678769700120  0.7426650695650597
  0.7147197341511895  0.0009433767513971  0.7815809606940688
  0.7891954193940370  0.5025329896815803  0.4962470032354002
  0.2902272317333306  0.9929963281066977  0.5351371846211802
  0.7827104764487828  0.4903315934594470  0.7632519233795367
  0.2786584385356897  0.0026576332357637  0.7611773789746995
  0.2274972199929399  0.5021841508625224  0.5155539298065863
  0.7317456906560627  0.9962382037733006  0.5016260016015168
  0.4624321788938835  0.5700026730335805  0.3916665567522757
  0.4120216384449458  0.5327602294165945  0.4416250610367894
  0.5362711738850908  0.5711627650232263  0.4184748406421038
  0.4347555347638644  0.4977152610275495  0.5181616922604081
  0.5585894342666701  0.5364340904599914  0.4958392331800864
  0.5080977601520521  0.4995633518164139  0.5475263094041357
  0.5349275219463633  0.4586787270251072  0.6267980243122627
  0.5076984647817777  0.4784605218088957  0.7201688695633541
  0.5521786011110764  0.4611551431105661  0.7939498740598002
  0.4382796301889506  0.5054275726601479  0.7378918730388140
  0.5269830505499004  0.4678104141090974  0.8820643326459506
  0.4130688818097235  0.5120501809694272  0.8265658473370381
  0.4561656711751784  0.4922341397339604  0.8999620953840132
  0.5157484582511970  0.3844070955229142  0.6125271297539958
  0.4284491984900670  0.4974921478971013  0.9954718002201562
  0.4697218906829061  0.5505270350777397  0.0501867950414742
  0.4648359992661751  0.5996406093306130  0.0180997866134844
  0.5286924184362660  0.5387281933875019  0.0546150254244985
  0.4479159981627577  0.5540707470771649  0.1191740222509744
  0.3693955486912087  0.5091915936753042  0.9945895677805204
  0.4343397417823749  0.4486734449614944  0.0288401710304893
  0.3546005510698625  0.5304540263315262  0.4209109864231130
  0.5767067230005738  0.5968383185847463  0.3766634946444237
  0.3948005910158401  0.4674346195253899  0.5546246770823265
  0.6166518758088442  0.5356453466076846  0.5151824863202695
  0.6069550298236992  0.4408820608290694  0.7813930120076987
  0.4029259364460513  0.5214159713656082  0.6826740293038764
  0.5623008114679708  0.4527124036779466  0.9380197099001107
  0.3582854228484103  0.5324235144056170  0.8387670710189830
  0.5954117945945157  0.4630713316729512  0.6279655176199401
  0.4559214036420374  0.3772815814708072  0.6142298451535374
  0.5364013169958872  0.3668092789805090  0.5473482534431034
  0.5396106205448370  0.3541437576335211  0.6667863063112482
  0.4452235195733908  0.5966256718262566  0.3311510260207025
  0.5776479673411591  0.6479182777081265  0.5090573627293277
```

MOR-Al-T4-TS14

```
   Al     Si     O     C     H
   1.00000000000000
    18.1842269897460938   -0.0090872207656503   -0.0001607598969713
    -0.0105508984997869   20.0691013336181641    0.0015994716668501
    -0.0001797722798074    0.0011982563883066   14.8440780639648438
   Al   Si   O    C    H
     1    95   192    16    19
Selective dynamics
Direct
```

0.5857727527618410	0.7146795988083066	0.6434236168861588
0.3147209286689848	0.0746522918343579	0.0371382981538785
0.3031393885612534	0.3202059268951581	0.0397054776549352
0.8124087452888492	0.5761272311210905	0.0330720990896237
0.8093664050102408	0.8006231188774560	0.0355368368327623
0.6913427114486694	0.9274783134460886	0.2844896614551548
0.7065250873566020	0.6831563711166677	0.2847285270691023
0.1954879611730621	0.4257185757160208	0.2882486581802372
0.1946010738611222	0.2006301879882953	0.2865326404571593
0.6936748027801514	0.0778129771351844	0.2359810620546398
0.6938474774360837	0.3029664754867644	0.2368229478597706
0.1930868923664093	0.5754956007003821	0.2407665550708837
0.2020708322525033	0.8208995461464600	0.2373254001140648
0.3098134100437225	0.9267864227295609	0.4905595779419099
0.3085673451423644	0.7068430185318417	0.4879266321659197
0.8147287964820862	0.4302551746368766	0.4877024888992503
0.8027591109275838	0.1841967850923598	0.4841268658638098
0.7076348662376406	0.9192172884941745	0.4925778210163271
0.6951250433921814	0.6841862201691024	0.4937306344509222
0.1871764212846799	0.4387821853161001	0.4934374094009515
0.2091405987739563	0.1919199824333283	0.4933212101459609
0.2977841794490938	0.0849345400929466	0.2448396682739322
0.3107613027095794	0.3171084225177885	0.2474244534969394
0.8191522955894478	0.5672305822372867	0.2356378287077021
0.7982144355773926	0.8144267797470607	0.2406843751668981
0.3197637498378844	0.9359025955200553	0.2851021289825443
0.2959986031055452	0.6922585368156630	0.2826333343982700
0.7973407506942750	0.4202905893326156	0.2794280946254734
0.8094523549080075	0.1856085062027067	0.2761711478233440
0.6881731748581140	0.0666380375623725	0.0309535190463073
0.7089702486991949	0.3136069476604642	0.0307166744023572
0.2098765671253225	0.5856078863144345	0.0319986753165731
0.1950484216213227	0.8177332282066977	0.0307602025568492
0.0856138616800309	0.3800760805606990	0.1352642029523849
0.0892154499888430	0.2249375432729793	0.1302193105220840
0.5890280604362668	0.8755253553391079	0.1407084614038525
0.5854759812355042	0.7155433297157600	0.1471508294343956
0.9181195497512850	0.6255719661713072	0.3819934725761520
0.9152082204818728	0.7803754806518985	0.3804495334625380
0.4169281721115216	0.1283675283193601	0.3932449817657475
0.4200935065746317	0.2865363061428382	0.3979578316211815
0.9169030189514167	0.3779465556144818	0.1308168321847961
0.9198247790336745	0.2189905792474890	0.1263005286455159
0.4211967289447846	0.8788331747055561	0.1415899693965914
0.4175765514373779	0.7238960266113870	0.1458407193422340
0.0871133282780647	0.6277751922607734	0.3812124133110176
0.0851059928536425	0.7862042784691410	0.3791110217571373
0.5855847001075748	0.1256121695041656	0.3880179524421797
0.5891273617744653	0.2807030677795573	0.3936356604099379
0.3165007233619690	0.0769774019718231	0.5375665426254272
0.3035927712917329	0.3223253190517539	0.5398265719413974
0.8118838667869569	0.5792810916901071	0.5379514098167599
0.8082860708236694	0.8017314076424277	0.5357964634895465
0.6901670098304956	0.9287824034691502	0.7838226556778148
0.7051371932029724	0.6815261244774430	0.7895934581756803
0.1952599585056305	0.4266014695167543	0.7867077589035356
0.1943207979202271	0.2016209959983897	0.7860669493675426
0.6947066187858917	0.0775293037295390	0.7352900505065926
0.6933752894401554	0.3037914335727812	0.7365582585334972
0.1898217946290970	0.5763995647430693	0.7404195666313366
0.1992681324481965	0.8233469128609090	0.7381274700164994

0.3110856115818024	0.9249243140221194	0.9895322918892123
0.3114440739154816	0.7026288509369327	0.9907389879227120
0.8158215284347534	0.4274331331253195	0.9855517745018340
0.8033505082130612	0.1837678998708857	0.9839867353439542
0.7077343463897707	0.9181277155877008	0.9914212822914432
0.6944423913955688	0.6823011040687873	0.9982065558433940
0.1876971423625941	0.4369889795780494	0.9922955632210166
0.2092885971069336	0.1902991533279479	0.9928876757622200
0.2988398373126985	0.0863385349512138	0.7448240518569954
0.3108773529529660	0.3181510865688501	0.7471103072166451
0.8236925005913032	0.5670611262321896	0.7429580688476570
0.7956736683845522	0.8119081854820803	0.7427689433098174
0.3192017674446107	0.9368443489075102	0.7843716144562090
0.2919994890689885	0.6940658688545727	0.7843188643455851
0.7983558773994497	0.4181225001812293	0.7790935039520565
0.8090180158615488	0.1871338486671534	0.7759203910827988
0.6872221231460571	0.0672072321176556	0.5299448966979985
0.7095645070075989	0.3142009973526313	0.5305061936378529
0.2035264670848898	0.5884996056556990	0.5310754776000977
0.1923826783895492	0.8223132491112113	0.5302041769027710
0.0851702094078064	0.3798157870769926	0.6352110505104277
0.0884442180395155	0.2245506495237437	0.6303723454475411
0.5875914096832294	0.8775720000267386	0.6407435536384590
0.9227179288864136	0.6238046884537167	0.8833582997322383
0.9161128997802734	0.7775990962982614	0.8772928118705944
0.4172497987747296	0.1274312138557546	0.8949540853500662
0.4201964139938355	0.2855318784713904	0.8991058468819000
0.9170658588409810	0.3772219121456368	0.6291104555130216
0.9194943308830259	0.2191062569618303	0.6252371668815621
0.4210295379161847	0.8794811367989230	0.6405560374259994
0.4154655337333679	0.7242497205734617	0.6460042595863695
0.0897633060812964	0.6278408765793292	0.8839875459671028
0.0851578786969185	0.7858145236969430	0.8798644542694442
0.5861557126045227	0.1246659904718435	0.8875390887260921
0.5890963673591613	0.2801628410816283	0.8927856683731380
0.1342604905366909	0.3888985216617714	0.2261726409196947
0.1184101253747940	0.2159076333046031	0.2332132160663676
0.2536838948726654	0.1218866035342252	0.4881486594677077
0.6136939525604184	0.9145562648773710	0.2322140932083209
0.6303750276565749	0.6796475648880226	0.2277946919202869
0.7383795976638794	0.6238698959350782	0.5466781854629867
0.8720627427101316	0.6161757707596145	0.4743889272213088
0.8858074545860293	0.7886119484901853	0.4833245873451318
0.7551861405372621	0.8852795958518984	0.2327026277780593
0.3937218785286102	0.0868733599782020	0.4833821356296698
0.3748787045478849	0.3271844983101051	0.4744928479194645
0.2696491181850433	0.3806460201740445	0.2934091091156010
0.8931178450584412	0.4178501963615842	0.0396012999117377
0.8761807084083559	0.1798473596572936	0.0469039641320728
0.7691987156867981	0.1213059201836649	0.2311513572931362
0.3706560730934143	0.8865324854851127	0.0519690290093440
0.3891402482986450	0.7158468365669430	0.0418113693594947
0.2555048465728789	0.6201362013817145	0.2915432453155521
0.1153936460614214	0.5859078168869455	0.2934419214725494
0.1309257000684743	0.8266333341599063	0.3025466501712803
0.2361466735601461	0.8806858658791140	0.9832516908645824
0.6334863305091860	0.1174035295844139	0.2965118885040395
0.6169347763061723	0.2902125120163161	0.2898795306682694
0.7523072361946107	0.3843760490417791	0.0372644774615765
0.8892890810966524	0.5763298869133516	0.3025194704532678
0.8658820390701294	0.8239192366600681	0.3108625113964186

0.7821285724640100	0.8755473494530276	0.0103274658322336
0.3635551035404207	0.1111601963639331	0.3094069361686842
0.3883023858070375	0.3048012554645715	0.2984128594398502
0.2445785850286484	0.3769822418689905	0.0114945601671937
0.1153239160776139	0.4293733239174052	0.0559350401163119
0.1402154564857484	0.1825143098831297	0.0611840412020705
0.2218410670757294	0.1251674890518278	0.2647383213043213
0.6412804722785950	0.8948380351067224	0.0568296350538747
0.6160754561424254	0.6910688877106149	0.0495289638638512
0.7639364004135132	0.6295493245125015	0.2435548454523159
0.1334425657987640	0.6094540953636594	0.4708352982998036
0.1160627156496049	0.8059520125389731	0.4784261286258880
0.2625857591629029	0.8760266900063024	0.2652902007103056
0.6142865419387817	0.0760849416255961	0.4669796824455393
0.6410461664199829	0.3233576118946190	0.4614978730678686
0.7228561639785962	0.3778721392154856	0.2589442133903503
0.8642463088035583	0.3966775536537354	0.2147988080978395
0.8864719271659853	0.1990821957588343	0.2241900712251712
0.7431746125221255	0.1283815950155310	0.5121311545372009
0.3925615549087526	0.9274156689644504	0.2219900935888343
0.3641530871391386	0.6825685501098973	0.2133855521678927
0.2742400765418976	0.6331392526627140	0.5041797757148923
0.2589396536350250	0.2515070140361798	0.2536102533340456
0.7431505322456362	0.7492800951004581	0.0119870714843276
0.7644750475883484	0.2563257217407406	0.9942683577537980
0.2382571846246741	0.7469248771668019	0.2439980506897064
0.7417573332786560	0.7575311064720707	0.2768503725528814
0.2657570838928223	0.2470504939556193	0.0300125051289806
0.2480734139680864	0.7524752616882761	0.0275813676416881
0.7563174962997437	0.2504904866218657	0.2688452303409666
0.0006469740765169	0.3984225988388241	0.1571391075849535
0.0055836043320597	0.1974648237228482	0.1236116662621523
0.5053640007972878	0.8978157043457676	0.1164044737815889
0.4996170997619629	0.6934354901314118	0.1531929224729562
0.0030508053023368	0.6069586277008369	0.4020060896873603
0.9993644952774043	0.8069452643395022	0.3745891451835732
0.5003458261489871	0.1084417551755956	0.3653420507907992
0.5058625936508199	0.3081662952900245	0.4019816219806788
0.0929590985178951	0.3032876849174605	0.1009941101074266
0.5933633446693420	0.7958399057388590	0.1576277017593432
0.9108276963233916	0.7024812102317811	0.3486598432064176
0.4108524322509776	0.2072075307369376	0.4166688024997870
0.9110740423202519	0.2986539900303007	0.1101815328002002
0.4158470630645752	0.8018872141838469	0.1758407056331705
0.0953754335641865	0.7068290114403202	0.3602949976921085
0.5933156013488770	0.2024281620979311	0.4226264953613285
0.1788348555564932	0.2105775624513698	0.3934074640274176
0.1616054028272629	0.4344807565212393	0.3886525332927708
0.6897889971733093	0.6633328199386598	0.3877409696579092
0.6839784979820440	0.9043740630150299	0.3884076476097214
0.8302619457244961	0.7948812842369516	0.1418503671884551
0.8484075665474122	0.5629680156708176	0.1319721788167998
0.3293552994728088	0.3326218724250977	0.1426608264446322
0.3239375352859497	0.0975962504744530	0.1412608474493029
0.8287622332572938	0.1721784621477219	0.3809936940670128
0.8233121633529843	0.4080668985843660	0.3830631971359253
0.3261656463146302	0.7155222892761539	0.3809305429458618
0.3448446989059458	0.9343734979630068	0.3902377784252282
0.1754604727029842	0.8342774510383963	0.1346943378448487
0.1847968101501465	0.5974687337875366	0.1359279304742862
0.6772512793541169	0.2944293320179088	0.1297829598188445

0.6603903770446780	0.0688410848379177	0.1351342350244524
0.2176065146923096	0.4976007342338920	0.2452648431062782
0.7124246358871796	0.0061430623754862	0.2806054353713993
0.7919719815254213	0.5053163766861387	0.9854812026024253
0.2906598746776615	0.9967422485351976	0.0335812792181969
0.7770352363586425	0.4980905950069739	0.2617632746696562
0.2804370820522308	0.0066588851623241	0.2625977098941893
0.2284039407968521	0.5074889659881642	0.0134817250072957
0.7279954552650559	0.9960217475891716	0.0099584683775904
0.1343267560005222	0.3885999619960963	0.7258773446083405
0.1174312680959723	0.2155931144952920	0.7337534427643022
0.2517541348934174	0.1192167624831216	0.9869965314865311
0.6131539940834045	0.9207652211189628	0.7297249436378783
0.6376550793647898	0.6718147397041757	0.7229458689689644
0.7367092370986940	0.6183973550796934	0.0425253026187428
0.8710002899170275	0.6168567538261844	0.9718924164772356
0.8830808997154622	0.7828798294067819	0.9789280891418465
0.7536914944648826	0.8833135366440295	0.7366385459900326
0.3923088908195497	0.0851976200938254	0.9837271571159574
0.3756029903888702	0.3264165818691481	0.9761950969696443
0.2707861065864564	0.3831326067447842	0.7912284731865230
0.8927596211433408	0.4194438457489157	0.5400828719139107
0.8749433159828187	0.1786445081234087	0.5478919744491760
0.7686951756477696	0.1233733221888614	0.7299167513847359
0.3675826191902161	0.8861429691315055	0.5527827739715584
0.3847696483135264	0.7182855010033078	0.5422808527946480
0.2517391443252563	0.6214450597763373	0.7920666337013252
0.1124986782670022	0.5850687026977719	0.7943999171257325
0.1280539482831992	0.8288096189499504	0.8034009337425583
0.2317509800195695	0.8870238661766396	0.4847035408020205
0.6331669092178345	0.1158539876341892	0.7955843806267086
0.6162420511245728	0.2906649112701417	0.7890599370002754
0.7522627115250009	0.3851367235183836	0.5379610061645688
0.8981474041938866	0.5717786550522281	0.8046389818191969
0.8680130243301393	0.8210715055466056	0.8066983222961769
0.7819035053253360	0.8779351711273748	0.5168990492820920
0.3640781044960069	0.1123453676700664	0.8101255893707487
0.3871127068996430	0.3040985763073088	0.8004028201103637
0.2466520667076190	0.3815238475799753	0.5155847668647771
0.1145813092589379	0.4293204247951651	0.5563427209854434
0.1401209235191345	0.1826078593730971	0.5615814328194053
0.2228500247001650	0.1267395168542862	0.7637149095535286
0.6390678882598877	0.8993886113167167	0.5557751655578620
0.6150600314140536	0.6938973069191160	0.5350125432014470
0.7704359889030532	0.6278974413872201	0.7687280774116720
0.1437895148992512	0.6111764907837308	0.9677882194519432
0.1185423955321315	0.8028475046158194	0.9788660407066804
0.2600972056388857	0.8783348798751949	0.7653847336769219
0.6163377761840820	0.0757394284009958	0.9664655327797242
0.6406284570693970	0.3230095505714417	0.9611909985542648
0.7214433550834820	0.3793068528175514	0.7580190300941475
0.8634808659553528	0.3925030529499271	0.7135999202728572
0.8856663703918843	0.2019900381565105	0.7240113615989693
0.7460522651672364	0.1264433860778854	0.0130587927997116
0.3913200795650575	0.9276179671287544	0.7211852669715889
0.3585134744644165	0.6857328414917426	0.7134129405021883
0.2857100665569307	0.6260595321655453	0.0108274286612871
0.2568578720092806	0.2540103793144369	0.7528913021087841
0.7455067038536252	0.7515672445297665	0.4989415705204118
0.7657820582389832	0.2577428817749113	0.4934507310390476
0.2343226224184047	0.7491809129715402	0.7482441067695625

0.7420957088470459	0.7559804320335942	0.7865960001945884
0.2633029818534851	0.2504548728466125	0.5297891497612004
0.2476594746112852	0.7594891190529295	0.5228239297867031
0.7557985782623471	0.2520779073238477	0.7711182832718108
0.0002755841123872	0.3981455266475861	0.6574942469596863
0.0051157167181373	0.1964994519949048	0.6235988736152657
0.5038418769836426	0.9016237258911490	0.6151238083839424
0.4927368413136089	0.6823676699490659	0.6587045241656289
0.0068999142386019	0.6072897911072208	0.9128583073616224
0.9994330406188967	0.8066623806954074	0.8783283233643011
0.5005654692650049	0.1069571375846899	0.8677600026130684
0.5057026147842427	0.3082104623317762	0.9017012119293407
0.0920877829194069	0.3029505610466003	0.6012882590294084
0.5918794274330139	0.7998476028442935	0.6607762575149849
0.9169190526008992	0.6997019648552377	0.8454188704490669
0.4123056232929230	0.2064068168401839	0.9190974831581418
0.9127213954925537	0.2984819114208342	0.6048215627670639
0.4172080457210630	0.8025196790695559	0.6750910878181660
0.0956746786832825	0.7065874934196903	0.8593720197677963
0.5935914516449032	0.2018904387951041	0.9210787415504756
0.1794525384902955	0.2106519341468900	0.8933022022247615
0.1617435216903692	0.4353317022323920	0.8875138163566597
0.6779713630676271	0.6683338284492923	0.8931915760040605
0.6798510551452637	0.9072577953338981	0.8886051774024971
0.8228418827056885	0.7900558114052243	0.6426567435264730
0.8478540182113823	0.5682448744774045	0.6373427510261543
0.3318630754947664	0.3324564099311949	0.6428806781768799
0.3261924386024475	0.0994486585259507	0.6419163942337330
0.8285889625549524	0.1723688095808117	0.8805522322655046
0.8243696093559265	0.4025099277496338	0.8820421099663169
0.3242227137088775	0.7135053277016282	0.8832545280456932
0.3452405929565466	0.9344513416290641	0.8892790675163481
0.1729276776313783	0.8356350064278293	0.6352075934410307
0.1818211674690247	0.5999324321747262	0.6365138292312825
0.6782056689262393	0.2940648496151038	0.6295306682586881
0.6613201498985291	0.0676968991756462	0.6347162723541471
0.2160629034042359	0.4988677501678824	0.7434314489364697
0.7164578437805496	0.0066465740092102	0.7805004119873271
0.7899968028068545	0.5076652169227912	0.4932354390621296
0.2922804057598122	0.9992968440056695	0.5341161489486916
0.7815870642662049	0.4967612326145316	0.7643278837204330
0.2808124721050262	0.0082795377820736	0.7628675103187569
0.2246298491954883	0.5108378529548804	0.5133038163185120
0.7281783223152163	0.9979074597359207	0.5052260756492795
0.5100136399269107	0.5503223538398924	0.9016639590263684
0.4369778335094452	0.5601162910461738	0.8728717565536810
0.5640971064567566	0.5385394096374562	0.8360660672187813
0.4205341432072878	0.5585042529992597	0.7817717044672383
0.5481042889668919	0.5363944090942327	0.7452568512670461
0.4754947514349191	0.5494029834928387	0.7124668352676816
0.4480559289603619	0.5153275578026631	0.6245493101068462
0.4758759438991705	0.4637126922607430	0.4754982292652236
0.5009243488311769	0.5046183466911316	0.5461933612823486
0.5148770213127205	0.4569441080093741	0.3953300714492957
0.5679501295089722	0.5377125740051582	0.5339753031730656
0.5827863812446645	0.4886202514172026	0.3852323889732489
0.6090192794799805	0.5281896591186949	0.4552134573459788
0.5283716320991519	0.5472295284271552	0.0000611301729805
0.3819164640889587	0.5519862998286299	0.5854194379851264
0.5171305537223816	0.4759345054626808	0.0365008488297474
0.5299402475357056	0.4742541313171744	0.1088579669594779

```
  0.4599699378013611  0.4593715965748145  0.0270315818488606
  0.5531253218650817  0.4403907358646750  0.0014102021232248
  0.3569616847473553  0.5226743010665796  0.5314609517017299
  0.3390684513071577  0.5625917239730572  0.6353022839819095
  0.4005827872775278  0.5989571751263597  0.5557984626433394
  0.4935063123702990  0.5822452306747780  0.0377739183604719
  0.5858513116836548  0.5623404979705990  0.0105014760047199
  0.3939115703106030  0.5684486031532338  0.9229099750518807
  0.6210157871246511  0.5307852029800846  0.8568760752678112
  0.3633515349727857  0.5631254154469405  0.7617708486938932
  0.5919088101036950  0.5241248233931435  0.6983138182490686
  0.4237749278545448  0.4374818205833747  0.4833048284053944
  0.4927200376987457  0.4262764751911521  0.3411753773689274
  0.5895012021064758  0.5723677277565433  0.5837857723236092
  0.6149985194206238  0.4825506210327292  0.3237425088882587
  0.6620330214500242  0.5523509383201919  0.4481617808341984
  0.4297308942509165  0.4654204864582918  0.6466142472988913
  0.4856697451332485  0.6187916115921948  0.6880412174143687
```

S4.6.- IWV Templates and Al Preferential Position

IWV-Al-T1

```
Al  Si O
   1.00000000000000
    11.6056398920140573    0.0835597568938375    8.7649673366017495
    -3.6825601025723138   11.3529798124118475   10.3282290959204754
    -0.2346490665193920    0.2412826202978807   19.0520352385695801
   Al   Si   O
     1    37    76
Direct
  0.0578885615711206  0.5497543827927378  0.1583823885976907
  0.1504601413062734  0.2369795335151668  0.0581418779826023
  0.1302736721299097  0.1060516200560154 -0.0000364784761048
  0.1031337626630027  0.3064995932596515  0.3025577575670101
  0.2564552174240393  0.1357285947586913  0.5622363266318239
  0.1042671082226954  0.1146097034563038  0.7754585921023270
  0.3293782089171540  0.0972762717711966  0.2971944775346906
  0.0066630188871282  0.7683220475423029  0.1286358186377751
  0.2878445998457795  0.2862216402390327  0.1095161408147828
  0.5598848628672555  0.0458673877029069  0.2524313001153796
  0.7783404772291931  0.0066881877833954  0.1032510789007213
  0.3038626808000980  0.2704782705590388  0.3284255378052182
  0.8604326596654269  0.7564751003432597  0.9524307477178321
  0.8804385289877525  0.8931682253107681  1.0035379815658374
  0.9056823295294384  0.6725056881602459  0.7233366120467501
  0.7528489956823288  0.8534939960382784  0.4488334962086917
  0.9030134431804098  0.8861240300389610  0.2272641766277111
  0.6756310959146942  0.8869759224482184  0.7200160239950804
  0.9526598441432454  0.4311073449441416  0.8591335912642257
  1.0050635418030760  0.2276354076759546  0.8743106076076251
  0.7229387093530091  0.6998618848869492  0.9055868928361865
  0.4481696151874659  0.9475998616272708  0.7526948790674618
  0.2313744009054241  0.9924800456347034  0.8993103147580390
  0.7140966964549699  0.7080799363368626  0.6837522937699914
  0.9130939426970391  0.5100721890042955  0.1000805206781558
  0.5131291417406230  0.8937704456468394  0.5005185346569230
  0.0951050736556624  0.4752124478046177  0.9159822703011942
  0.5022806627878934  0.0889546225844473  0.5160298312922449
```

0.9006613285419661	0.0880031192047477	0.4643998627170597
0.8783555496483357	0.1250713635014435	0.2898599014683668
0.0616587204923741	0.9343960479150761	0.5205444083897627
0.0898698274235727	0.9066510040815852	0.6814681211116397
0.4604580460721643	0.5471998463476571	0.9012459396309532
0.2919290419194367	0.7105735470757372	0.8769613425239220
0.5198840012780302	0.4838854901469296	0.0609615985289922
0.6797915454360128	0.3212732130409708	0.0886625046857046
0.7220408859623924	0.2960004463770655	0.2445976652610352
0.2444811861945669	0.7347691161881499	0.7246325024325329
0.1928413844444073	0.1829133028786385	0.9977380065434703
0.0141465568121143	0.1566326368641983	0.1818436545615887
0.2386372194046020	0.2288284172109399	0.0872347838985726
0.1578523954831603	0.3758145054942434	0.9668890508086068
0.7719797858925783	0.0194695397910502	0.5965361680825253
0.1771719646483832	0.1698135988428797	0.6412687601563052
0.1808838051226356	1.0192845946602498	0.6174057262152630
0.2972181864548198	0.2478199995311389	0.4281413978486954
0.3710980741819058	0.1079251909988691	0.5604857539756501
0.0189272150815436	0.7930305995902384	0.5991416359066434
0.9997584053657017	0.6329873599163043	0.1966094093919566
0.1922182110095285	0.6427423706046748	0.0130443416030194
0.0880580786158460	0.4332726578786680	0.2536851993395739
0.9608280437283350	0.4926077358587213	0.1637933633613766
0.5970154396750887	0.5944199613731879	0.7819227435395680
0.6426157831074076	0.0098996366928337	0.1757123805093072
0.6159831142560134	0.1836279999315715	0.1737900930750627
0.4294601530839744	0.0275426035748003	0.2857653728139592
0.5511103727791644	0.9643491427445396	0.3707579026536600
0.5982020911541562	0.5910050959663042	0.0166145054207523
0.8157051673637065	0.8170906614123361	0.0067246776408590
0.9745469934021364	0.8555530714988894	0.8116326046056599
0.7524088964163376	0.7206430561168512	0.9636141824458577
0.8989830159666490	0.6426476474763442	0.0220050382380046
0.2005503621908708	0.9958078970534074	0.4021874511858123
0.8269960893756935	0.8260873010905819	0.3638268193574187
0.8027811138683181	-0.0064878163281443	0.3695136674342829
0.7722331493520190	0.7698773400053400	0.5501914947081229
0.6099590903927180	0.8229495126507081	0.5133366600796578
0.9720191201146680	0.2035755286334326	0.4136303586226558
0.0053259080635158	0.3642539060059496	0.8109718288287496
0.8129594358121860	0.3583705565080541	0.9750496045700294
0.9640893831275477	0.5645044793221365	0.7532046146835103
0.0271033811338186	0.4326800088279492	0.8961193791771938
0.4039573590748326	0.3997794232409546	0.2011016958700508
0.3682997865674544	0.9953465776259869	0.8187609142796068
0.3646095488484369	0.8437887654534534	0.7988543880191666
0.5430454890987098	0.9014501836423321	0.7810380933643363
0.5231582459683938	0.0534026350449854	0.6069642622451423
0.3915564645111104	0.4128724093981126	0.9788086533129114
0.0008123836258729	0.4904506285761883	1.0081704407650989
0.5116859621977535	0.9859266574145864	0.5137516918485550
0.7801444695553874	0.4138208419454114	0.1998515676327127
0.0668041083885199	0.1847418913214159	0.9374344595488338
0.3820786871489973	0.7973284293098286	0.6028747936980542
0.1827779936533667	0.0513836637619419	0.8221719993720932
0.2013636596732875	0.6001706295461248	0.7865195141496890
0.9490138847637705	0.8190003847999986	0.0594298527410106
0.6027351659663241	0.2137817235567536	0.3832096584776984
0.8226450213859203	0.9523328536111905	0.1826958718748836
0.0280167663055939	0.9857455824763982	0.1391770614673606

```
  0.8773764735009076  0.1278493677779932  0.3738252346852474
  0.8170369644947071  0.2203005409148473  0.2323552299713979
  0.9773654681439802  0.0177090283565319  0.8645173312241967
  0.0460598317172398  0.9499457857674359  0.6033180359490679
  0.1579717562580390  0.8071728876323935  0.6904739295768785
  0.1450257205849459  0.8576217611479136  0.0257146776646719
  0.3905806975673268  0.6392580787637510  0.8568715965371844
  0.2346163755195865  0.7324231955779652  0.8151262650043997
  0.8672927854451872  0.1416059982624840  0.9756300711519996
  0.6051635050093873  0.4037879198041482  0.0418559144162367
  0.6847120417827217  0.3371194871983613  0.1624612459988788
  0.2324053534558687  0.0725037845638074  0.9254755370462140
  0.0796123850058837  0.2233941373315967  0.7712285146553899
  0.9308979605037707  0.7827179149736048  0.2254381271098493
  0.7773427463831321  0.9246482405943545  0.0800049732576169
 -0.0211142285295040 -0.0058116187458387  0.4782861645900974
  0.4727347425645551  0.5439586937882840 -0.0171524151583295
  0.1779349519953585  0.3015581841563004  0.1981171496340641
  0.3442508253854155  0.1638623374144508  0.3302403624463713
  0.8331293653276191  0.6671405631195162  0.8334392949593986
  0.6773002235146037  0.8069103502425656  0.6935414019853358
  0.1748216712517631  0.2670488766959963  0.3571237454112893
  0.3492128878822700  0.1961593537073571  0.1712617724667908
  0.8129981051384759  0.6616346749902320  0.7061105951982911
  0.7073578798954675  0.8207994987609873  0.8111842547819925
```

IWV-Al-T1-DPDMP$^+$

```
   Al     Si     O     C     H     P
   1.00000000000000
    11.6056394577026367    0.0835597589612007    8.7649669647216797
    -3.6825602054595947   11.3529796600341797   10.3282289505004883
    -0.2346490621566772    0.241282269865036   19.0520343780517578
   Al   Si   O    C    H    P
     1    37    76    14    16     1
Direct
  0.0727095556729956  0.5679846451833895  0.1267261684012913
  0.1817140299744578  0.2672268770081681  0.0214656070333697
  0.1479031848258210  0.1266582682224260  0.9772327602103126
  0.1323865500690780  0.3334693848950730  0.2661808283774236
  0.2811585780207299  0.1638792080501637  0.5331738382671352
  0.1272696537233248  0.1389184162136493  0.7480956739279316
  0.3566971011198404  0.1232979614253993  0.2644351688103888
  0.0341763347783644  0.7898209133173112  0.0987011485087851
  0.3191962656913887  0.3125982026602774  0.0740413308810148
  0.5920930126973735  0.0775942082011400  0.2112350639149519
  0.7989663514215168  0.0202440377645459  0.0793037888585755
  0.3306318355100903  0.2965222399567684  0.2971881415197249
  0.8906442353269339  0.7801748466550850  0.9183739704704176
  0.9060008625003009  0.9154730165824388  0.9737522035935278
  0.9323561049509534  0.6998985090611963  0.6912627311894017
  0.7799654370399612  0.8814210242230941  0.4139473150455985
  0.9268419327211607  0.9011219970859126  0.2024949638976385
  0.7026633459020676  0.9157607509292839  0.6900940594431614
  0.9817114351404901  0.4608699926815908  0.8289257852728507
  0.0207537052229223  0.2466307400211657  0.8544213255240418
  0.7499005825559322  0.7254669528451430  0.8747159953633394
  0.4706257810914692  0.9694693437087468  0.7329428942149741
  0.2543007146855440  1.0163386102785970  0.8716341575062151
  0.7393333509907302  0.7399204759065510  0.6511216291821915
```

0.9378253767384552	0.5312649598539665	0.0640094634722347
0.5446796274546546	0.9359630583365606	0.4561698806829753
0.1369366879199142	0.5089880019522400	0.8725606695409811
0.5263950931326131	0.1170018511378573	0.4893985221877540
0.9279004988628518	0.1108396374003011	0.4342195504352820
0.9066021587224274	0.1518886203584609	0.2566819657022695
0.0912192604240363	0.9599407561858203	0.4883508455893949
0.1193713120532810	0.9352812876750716	0.6479866179026315
0.4878782054910333	0.5725752319662780	0.8708525519063482
0.3188768372133750	0.7371042365720168	0.8445837601656808
0.5486313238300561	0.5083991176534873	0.0303245401159628
0.7064446962512596	0.3432326623972826	0.0598461349296385
0.7386049463922835	0.3134032749694986	0.2211551489185095
0.2839013929136730	0.7746789850615539	0.6793186558018357
0.2154790415094421	0.2037998514032314	0.9711346609025456
0.0439171775054716	0.1989952328534329	0.1421690777571858
0.2701274776416361	0.2552419486299051	0.0516295014415394
0.1996974227738261	0.4073093355162417	0.9202577734258254
0.7999164246136106	0.0473982542048518	0.5655458957646724
0.2004582877291420	0.1984637218238359	0.6119800265472548
0.2092830681660211	0.0430569579585293	0.5915717973907048
0.3177887985190336	0.2724510021299650	0.4003292374769105
0.3988666249995580	0.1439539087633643	0.5269136475771389
0.0510928801951654	0.8168236719714662	0.5700871177597020
1.0264111381526171	0.6558529786154965	0.1600962187673598
0.1996224527552750	0.6558024014380321	0.9775191712165098
0.1125065620518691	0.4594267810292093	0.2166243464222316
0.9669811275764246	0.5035618102932253	0.1446984570644053
0.6253921209918557	0.6224969801518027	0.7516916832949259
0.6610820457453126	0.0164112107546426	0.1581021420839624
0.6606377155141735	0.2194040638092892	0.1054982894933367
0.4572575673973923	0.0535456113145171	0.2544585249011775
0.5918175129905894	0.0205079866791573	0.3201926985858602
0.6237852835888461	0.6190524086801718	0.9841141243569014
0.8454338739912732	0.8349480116967155	0.9782649224545605
0.9986116168348139	0.8844135072658021	0.7762373337215105
0.7780390845494963	0.7420419223077593	0.9360179045843424
0.9374503976863586	0.6693183841959205	0.9784618335640214
0.2285392601090563	0.0212404580056102	0.3665246173064308
0.8469992184957241	0.8414826548754600	0.3394967140670905
0.8443633075345829	0.0211044730558703	0.3241423768509994
0.7932201136250413	0.8006261141308743	0.5192266987623606
0.6384701217019659	0.8617540231481896	0.4719439134011238
1.0036897132812643	0.2272322602519480	0.3788641888511118
1.0155280984992463	0.3816728148105757	0.7948099350126110
0.8504457165671288	0.3960911911131150	0.9566567270905051
0.9835184699050896	0.5868902345141994	0.7228019611364513
0.0813011605953526	0.4815431608066199	0.8338760806659065
0.4367789996157310	0.4222830725932481	0.1717923290216349
0.3946036731738065	0.0261572564313661	0.7903365237810280
0.3808141885812371	0.8544579501028180	0.7996962921619309
0.5712782452506043	0.9318062434529018	0.7540264585308014
0.5395370085988089	0.0684266417009995	0.5888407237553770
0.4220871619423180	0.4395059186071388	0.9434341296007887
0.0346910870275564	0.5106230773874078	0.9741981802021777
0.5366180398681705	0.0194973923766173	0.4803979707005046
0.8040281168388106	0.4465170436164246	0.1469804967741075
0.0830884690748637	0.2065515295983614	0.9164106095532485
0.4145080063317090	0.8421985146956092	0.5481811361596379
0.2075848896024613	0.0756083107661468	0.7927636294091778
0.2441170018631271	0.6352996387146320	0.7509506821416294

0.9727468811477957	0.8468966372038949	0.0322580855833008
0.6325515705337121	0.2425214844427136	0.3619352971988107
0.8469912290533048	0.9609487707485889	0.1562581282784812
0.0466689353173934	1.0060400629260404	0.1163733569724241
0.9013780734483604	0.1455822307908317	0.3475113078774166
0.8359559502317589	0.2404385406816357	0.2091015997625839
1.0024221343333555	0.0403944326495835	0.8350363521311358
0.0830381611089772	0.9832692072053474	0.5631668744793156
0.1850540932365073	0.8337447079299315	0.6596149734087324
0.1730723119372294	0.8788149690037264	-0.0053900014843138
0.4179185475707461	0.6653814384935355	0.8278164447281566
0.2933416597187725	0.7902071802871896	0.7507360345678918
0.8844526945911804	0.1575890968604493	0.9556929478536982
0.6396502722831434	0.4338768281547457	0.0063469028122055
0.6760569519939722	0.3212329746110993	0.1690239415812630
0.2491059274769802	0.0942503850051786	0.9014842883426151
0.0975291290542585	0.2439062262312017	0.7498989071693176
0.9640598795692916	0.7990903519910847	0.1990850928567877
0.7970384111613568	0.9452507768891704	0.0493750756954121
1.0027984034207285	1.0136022520759056	0.4526266440661584
0.4953614092960855	0.5632274882188865	0.9580830293096745
0.2088377814145531	0.3273201653739793	0.1629041763113633
0.3673493433265757	0.1882821942355405	0.3006354867606953
0.8597735422141303	0.6928746404435503	0.8022341152363737
0.6989576711234109	0.8356418142851021	0.6652648499861189
0.2053448115292003	0.3023713365151959	0.3177372654226689
0.3799580001092163	0.2223614902688595	0.1367623528188326
0.8436449076770051	0.6994693332945542	0.6661226139110861
0.7366040558193747	0.8485163864261909	0.7798283462955180
0.3975711171084910	0.7354838879162893	1.0829594521752630
0.2245668993882691	0.5612180730023697	0.2746755049996250
0.4134378072168671	0.7475893666244486	0.1417190217133301
0.2399899576644403	0.5719545741540190	0.3343216884442146
0.3342117319123195	0.6661665228013613	0.2675642993847882
0.3029673658961730	0.6430354735026795	0.1492890584042991
0.3685789875126691	0.5509001153823447	0.4478000313503484
0.3305209087644083	0.5183303864739586	0.5521453179014950
0.4285670120246236	0.4843445294792103	0.4190516577026792
0.3533669858817164	0.4196855709227152	0.6268038244057254
0.4512938075706802	0.3861481700269174	0.4941786350935099
0.4139592815719064	0.3540248605049569	0.5976769007177103
0.4749097654860739	0.8104324970938306	0.2388399755116422
0.2160648780033036	0.7076726990430495	0.4281572886541820
0.2025288511566139	0.7865483667367434	0.3655739270177787
0.2265564748808725	0.7235346736675493	0.4714438272679710
0.1377747922419459	0.6295851274842231	0.4971704059130462
0.4316125593942263	0.2770774520333423	0.6563369830767024
0.4591951796140329	0.7985840668431834	0.9852906329796630
0.1516920425223901	0.4894878866258259	0.3246424275994649
0.4874609399693751	0.8199335500679131	0.0893693972341362
0.1796076787004138	0.5070471223089236	0.4321874816032943
0.2828445508669771	0.5685407047497346	0.5760710093114632
0.4564775073981248	0.5086603197541593	0.3389909694669561
0.3235118979288252	0.3940463753521427	0.7079938840207327
0.4980496204002824	0.3347292224087738	0.4716922299236305
0.5584639698301086	0.7978792735185091	0.1883718617502578
0.4625308934837988	0.8914320850185993	0.1766628113053661
0.4808532101830073	0.8207996424423724	0.2877311460756997
0.2892663845438130	0.6332709469122744	1.1043111143558186
0.3486454210898320	0.6826229391464216	0.3453628039803569

IWV-Al-T2

```
Al  Si O
   1.00000000000000
    11.6056398920140573    0.0835597568938375    8.7649673366017495
    -3.6825601025723138   11.3529798124118475   10.3282290959204754
    -0.2346490665193920    0.2412826202978807   19.0520352385695801
   Al   Si   O
     1    37    76
Direct
  0.0042252065522586  0.7628495742003188  0.1316941091038256
  0.1478257754879317  0.2315778707434320  0.0617252424833496
  0.1256795272681417  0.1038557197738090  0.0017233272143584
  0.1056763811254039  0.3081649691026382  0.2945487197272664
  0.2560702285195429  0.1337005559747697  0.5641700945772967
  0.1015832742566190  0.1111338070858133  0.7784311795825489
  0.3285148992757462  0.0992961679604722  0.2932300903144168
  0.0578863468224036  0.5492301346275898  0.1537928426320382
  0.2910145210060203  0.2883159774099712  0.1025088891780906
  0.5588502231317592  0.0456504451450469  0.2532520756080665
  0.7768369734668912  0.0061182597527201  0.1028470986470417
  0.3036408707301640  0.2716938206035156  0.3269055106339387
  0.8586610864742545  0.7611484534877715  0.9500469479222446
  0.8759662254460557  0.8932976985699151  1.0039521502834001
  0.9072387103221762  0.6735879459614624  0.7218719344187244
  0.7488997197901500  0.8584154788677448  0.4489074055522112
  0.8988417061078848  0.8818986279281463  0.2306070472131294
  0.6745779888834158  0.8899254401637716  0.7219778601994373
  0.9537886498524225  0.4285062581847485  0.8592653621953317
  1.0039877213206929  0.2265541601996053  0.8740694818166950
  0.7231998552718465  0.7016096156071370  0.9041533113307804
  0.4456932539092181  0.9488948777672250  0.7551087743284415
  0.2298485217670763  0.9893483053958829  0.9014326954373715
  0.7155622709351454  0.7132744635063808  0.6819185667111929
  0.9182209977860167  0.5194478519186839  0.0891046910143239
  0.5100010273466090  0.8960284877773195  0.5008582669403704
  0.0967057453056973  0.4741281310726745  0.9145939252112141
  0.5057987301284265  0.0940485436241438  0.5115400368807946
  0.9003246857329078  0.0849053839411433  0.4684436607797621
  0.8799297851929717  0.1254052033428187  0.2911412434734162
  0.0657731663138424  0.9328177010415807  0.5194281370772219
  0.0928056573536878  0.9036437562381807  0.6835691626137045
  0.4618568849687013  0.5451280327408032  0.9022256386556474
  0.2914498089555407  0.7086031594292699  0.8819488214997554
  0.5239141866850413  0.4790308236369994  0.0625629739809607
  0.6840427591091764  0.3181019073489143  0.0924108461290762
  0.7296618544571334  0.3004147699728166  0.2425857709789926
  0.2422689562252133  0.7297240082061099  0.7324837483767641
  0.1893453332394378  0.1821040930307611  0.9981096826107068
  0.0147649946533779  0.1465333343381839  0.1899595290815541
  0.2417412214811591  0.2265194989608990  0.0841689185546604
  0.1473219196170446  0.3692305438963652  0.9787783780359554
  0.7708425716414071  0.0230414595254407  0.5979696589436844
  0.1749861426775525  0.1660632313017159  0.6438668014175500
  0.1895152699436092  1.0078389943759969  0.6252728443002755
  0.2826604531692946  0.2408269639678410  0.4358513583375919
  0.3798432997757581  0.1237623683928896  0.5470320108859243
  0.0252291834223139  0.7906940009744646  0.5994630538093674
  0.9971571132797766  0.6158211018142014  0.1982723484022330
  0.1905323469011372  0.6323519620536834  0.0224008370734733
  0.0790970242920092  0.4329624956257870  0.2464313890684704
```

0.9781266412182037	0.5002411811573385	0.1460022973015125
0.5990655145000501	0.5977034100583616	0.7821164809118449
0.6425970695228259	0.0145808110980286	0.1728575850731567
0.6137753403021271	0.1805789734572352	0.1831380169379506
0.4300912232391603	0.0319837455391496	0.2818468193739246
0.5438475703381020	0.9562891819470734	0.3756398186204433
0.5987215393263245	0.5938235184115420	0.0108516913764113
0.8094407375700174	0.8234109031872617	0.0020319260649757
0.9697499644987901	0.8599961317249909	0.8073911826666036
0.7508723599483946	0.7135635661817464	0.9681768014427585
0.9042924816772393	0.6518290104628631	0.0171665381452375
0.1999381477913795	0.9989369844502163	0.3931908170757696
0.8219417639839599	0.8320701448041573	0.3641946900642041
0.7936253915860639	-0.0009354238432162	0.3719028783073761
0.7702713081618611	0.7772255154636105	0.5489535023611869
0.6048853465155467	0.8248878183399693	0.5176011525521190
0.9783578146820670	0.2011926121423101	0.4122650442859773
0.0038068166986996	0.3639091160581627	0.8092255725026345
0.8175603233777236	0.3496884265743589	0.9826570241924621
0.9570672523099221	0.5601244777442200	0.7586266227978218
0.0369304557292379	0.4367168700224765	0.8859219250106283
0.4149117317672921	0.3942885764009360	0.2048079723299115
0.3717464417854891	1.0057079813485683	0.8113724193507549
0.3559275555728852	0.8400209393390244	0.8116521948047972
0.5413255643415437	0.9027984215513474	0.7827230397329761
0.5207974838644325	0.0465610895465790	0.6096082870235815
0.3924745015266813	0.4157368563022990	0.9704700973014047
-0.0010078964540160	0.4990436449820083	0.9981601730198797
0.5116295775710821	0.9963352289722628	0.5037049269579956
0.7859761520420454	0.4205185800678327	0.1968299178743291
0.0640142387811686	0.1864028608052433	0.9378794750867315
0.3777725096694951	0.8003997687545795	0.6085021169156025
0.1802395009897451	0.0475004383310540	0.8243437301745373
0.2095840586125950	0.5954755640160396	0.7893716761727577
0.9412465455698503	0.8154326975602638	0.0599084143160432
0.6142497564548597	0.2162946784325652	0.3826324395339769
0.8194999907719850	0.9495586153421384	0.1836414822174566
0.0227242940306324	0.9855778175420946	0.1408000838144058
0.8792563257828865	0.1258100351012271	0.3764328117381862
0.8298290053736376	0.2306122710700783	0.2252142827126088
0.9729791491896613	0.0193813346164567	0.8643438152620209
0.0632122469695730	0.9540800986899823	0.5934176755638263
0.1489501619540438	0.7941676563124676	0.7061623484199979
0.1538433498409096	0.8534412382330225	0.0224460868979748
0.3919340087311234	0.6402580370685285	0.8615606525597178
0.2303867649037207	0.7251613205852612	0.8255796518467268
0.8663581521382099	0.1411681900136385	0.9750191416595612
0.6142090353246205	0.4036060677183370	0.0399172007269117
0.6894685117536997	0.3383529591224373	0.1634703687774678
0.2282017386992299	0.0721665694178015	0.9261626474994868
0.0803042728135579	0.2221730299846654	0.7711830800003643
0.9247428065244793	0.7748267300647153	0.2369835499281812
0.7717026176060825	0.9266163916294301	0.0787877186529969
-0.0271798426872049	-0.0137175377105507	0.4896009502911594
0.4664962385210138	0.5266905235562230	-0.0050362809096541
0.1791354571093990	0.3048397250182455	0.1900719785322962
0.3347682557937122	0.1618008975590421	0.3331458647952986
0.8343385954422063	0.6723991586526588	0.8290599105972798
0.6775253012502012	0.8087592415728490	0.6962180669552622
0.1840124738182689	0.2891632432237923	0.3365156319457546
0.3528493290136480	0.2021712862833528	0.1650024978513017

```
  0.8175248750580325  0.6681815419297037  0.6995735433975683
  0.7072130418139440  0.8251928193632649  0.8123855192487774
```

IWV-Al-T2-DPDMP+

```
   Al     Si     O     C     H     P
   1.00000000000000
    11.6056394577026367    0.0835597589612007    8.7649669647216797
    -3.6825602054595947   11.3529796600341797   10.3282289505004883
    -0.2346490621566772    0.2412826269865036   19.0520343780517578
   Al   Si   O    C    H    P
     1    37    76    14    16     1
Direct
  0.9991309977383901  0.7554983084586308  0.1370996653386160
  0.1495634765947992  0.2340958825832582  0.0566606230395739
  0.1173254543648706  0.0983298847685127  0.0083304878583395
  0.1112105271603923  0.3148558599332675  0.2830476995860834
  0.2487910652638846  0.1348511582232761  0.5667570275210834
  0.0965353573798356  0.1091125490869275  0.7826617738622902
  0.3274022871335658  0.0973201298157748  0.2941173064338154
  0.0444013884082652  0.5477499068627717  0.1509975475246858
  0.2942860275003785  0.2825725268447140  0.1006776172418720
  0.5636999920956260  0.0523432967004870  0.2435787187671343
  0.7696433511171880  0.9949721525860197  0.1104190716312317
  0.3013357410084026  0.2748476761857104  0.3253746568449922
  0.8557099198548690  0.7573534527390440  0.9501519426907901
  0.8712740578591531  0.8872421606403632  0.0081983342826043
  0.9016718457787007  0.6739862671093704  0.7232071388013733
  0.7452927281068155  0.8559334480481041  0.4501524938999315
  0.8951486065419225  0.8725494095559702  0.2366444196404769
  0.6791285661097524  0.8985662722715855  0.7153315645483158
  0.9551847633102886  0.4359578446546419  0.8571791013412040
  0.9914346923271039  0.2200874780716544  0.8854616670713611
  0.7211690901754224  0.7031165277580222  0.9021199242722838
  0.4423215273268201  0.9441411017331244  0.7644398587670052
  0.2237832244398150  0.9856966657343245  0.9055822054374997
  0.7075205517704335  0.7174662550366306  0.6841027796062817
  0.9095873561320773  0.5117698357987309  0.0905806662338445
  0.5119902435277346  0.9083190194888130  0.4903548238634809
  0.1065327744833483  0.4816498397014948  0.9050617299360775
  0.4965185070828490  0.0922611126233384  0.5193068233554949
  0.9034704063275736  0.0930035567898137  0.4612838076539760
  0.8789263519868715  0.1252726732113496  0.2900011012054544
  0.0643843114912271  0.9352498048490447  0.5182744635625561
  0.0910617654642252  0.9042718519466423  0.6829240433727862
  0.4511717035445305  0.5386153774083977  0.9124652573611096
  0.2863122089027649  0.7111609463309259  0.8796902987097973
  0.5199029301182566  0.4800159998359741  0.0651503708527114
  0.6817355668910752  0.3188605711709057  0.0901547278450541
  0.7160214701404917  0.2877989820967813  0.2512862389243098
  0.2542138228653308  0.7438108256593489  0.7141790395179168
  0.1855038642722784  0.1779415636384136 -0.0000188046837698
  0.0149544238682192  0.1566594237917982  0.1829417750342349
  0.2445437757092310  0.2253508884443862  0.0790848678599347
  0.1578470330087658  0.3737774102370707  0.9650907409968137
  0.7753428849122906  0.0316457222766599  0.5916503662908954
  0.1687947556840391  0.1679899224738419  0.6465753096297875
  0.1840010415063974  0.0063634582377235  0.6311241010962924
  0.2701737610078337  0.2381704816670779  0.4408445435530263
  0.3756792904090659  0.1316017611980834  0.5443700962300425
```

0.0180210576112996	0.7929704105672406	0.6008753740060595
0.9865417397417463	0.6072563649018428	0.2042547645863599
0.1568117569652135	0.6449008086841799	0.0077214413032635
0.0935334773288290	0.4446724158987411	0.2201006812327554
0.9480773948233867	0.4845763963767162	0.1668562997486281
0.5877511678150850	0.6040209493382444	0.7881889060816510
0.6324616351473678	0.9935185464132593	0.1885733300700491
0.6303664493770251	0.1943603738116494	0.1403627527415979
0.4283661088746548	0.0287766763372656	0.2878836674287245
0.5632460269732996	0.9931580603788159	0.3532587063133564
0.5923172859824970	0.6022673738697365	0.0049113791037823
0.8080168744304385	0.8173274192365282	0.0043840229413683
0.9660158496292972	0.8563533336548188	0.8078085285164611
0.7450446873400797	0.7119467962931123	0.9701151194163936
0.9010528931198011	0.6464276717654390	0.0160249642314420
0.2001722308424983	0.9945562748329958	0.3954279399679331
0.8157115436428626	0.8241019583425098	0.3698115485335234
0.8009553575834893	0.9949678780582664	0.3703240970423235
0.7589519371859610	0.7704821389433733	0.5556781799000274
0.6029233982941263	0.8323622597148839	0.5108618852460766
0.9807403395769225	0.2124303124909870	0.4018694998058627
0.9883094652731869	0.3563756779372255	0.8237996178204559
0.8250209917791941	0.3687450280274104	0.9867043181566653
0.9534722404962388	0.5615797881623494	0.7538347435784447
0.0538313189372898	0.4576530914694814	0.8628486520628026
0.4177759162226211	0.3937005311784931	0.2090743632891050
0.3664413915231013	1.0012624612750070	0.8201569629507350
0.3570922268670995	0.8240310728816543	0.8329397406180887
0.5474061583900116	0.9132038750302921	0.7790360429260341
0.5051051773716539	0.0417121569899832	0.6217151553704534
0.3974600174753017	0.4096265759842723	0.9692646449521248
1.0048036824794873	0.4944087956255671	-0.0011611675453846
0.5000737799933541	0.9921751132351881	0.5151685694766547
0.7765070249841425	0.4196263784833592	0.1848127582581576
0.0515100860976657	0.1802226071065360	0.9490751927039653
0.3818770773707138	0.8153071476848454	0.5808485400980991
0.1802548795234022	0.0474449620812258	0.8227753411327847
0.2195134312739140	0.6035663339783005	0.7861556348658670
0.9299729744571461	0.8064666684186990	0.0711738776468215
0.6111286601695695	0.2118181363723802	0.3931140471554291
0.8174523142622512	0.9369740991763471	0.1872007059391263
0.0174201829553705	0.9778902697278371	0.1472094346415339
0.8785069215944208	0.1290659486441767	0.3732519836646968
0.8195361227281244	0.2225901602744702	0.2310217842215708
0.9698502610211601	0.0137151137581601	0.8702074570111926
0.0633341736779507	0.9579177299122978	0.5907654899218802
0.1499268168308668	0.7969307252699648	0.7020681950139522
0.1466331106048756	0.8498186055580677	0.0253963795422806
0.3663318802171426	0.6200944058189456	0.8870060139909762
0.2663467873928138	0.7606816331877334	0.7839555870926980
0.8550064551991027	0.1325759274667275	0.9850852922469395
0.6162196845928297	0.4113846823017617	0.0363286302371471
0.6551005618933924	0.2999302568269687	0.1966048562209552
0.2195271246072743	0.0700709027798779	0.9292056329016642
0.0718241558267063	0.2172163099082126	0.7807376906401364
0.9227624446243951	0.7638567407748460	0.2451950670436447
0.7638966602494108	0.9189502718955446	0.0831766552058266
0.9772777444285893	0.9955742738912522	0.4818149857771958
0.4519989292255224	0.5148224795308585	0.0093881591079723
0.1825281304181763	0.2985704833507559	0.1883605346610020
0.3318917961397150	0.1662322896877685	0.3291427109667294

```
  0.8309270275066069  0.6670206064382523  0.8339254457055032
  0.6763428916715711  0.8204777818048409  0.6881013832037937
  0.1878522641547983  0.3017254217397410  0.3246168766718587
  0.3538334451428391  0.1937601682291153  0.1648166458222445
  0.8087254902399515  0.6714410140287900  0.7028182021477856
  0.7162214771607410  0.8303098483601099  0.8027150800545076
  0.2278529210152867  0.5612423625863412  0.5352098366158566
  0.3895970767835181  0.7423863303581852  0.3863102425803232
  0.3084600710993977  0.4948099181347395  0.5379508623283364
  0.4707398391510255  0.6770123993409671  0.3884908398945900
  0.4300009910538095  0.5528168170783440  0.4648449679856000
  0.2680897747710232  0.6843382429966932  0.4604248076771808
  0.5738600371661066  0.4807932289630754  0.5435135636588220
  0.6936690709378924  0.5244096888474595  0.4870953329550788
  0.4801769118016834  0.4496732746678069  0.6525920389429571
  0.7193158553511975  0.5371079702295913  0.5402085834619978
  0.5070539119035296  0.4616485858515357  0.7051793082608677
  0.6266235647957736  0.5056944706325448  0.6487049852640622
  0.4651031632179662  0.3165287813518143  0.5679109057510981
  0.6667860392211659  0.5318216181538774  0.3294705091475537
  0.6416047216528750  0.5193984948446373  0.2920548846912951
  0.7337964539018380  0.4862843068216762  0.3358797910087802
  0.7063331687085822  0.6274022582302937  0.2698544620885089
  0.6467726687735571  0.5168546433475593  0.6893459859017045
  0.1334092421156887  0.5158330277740379  0.5929047441435930
  0.4212316310159956  0.8384951098447918  0.3273292926369805
  0.2754046299291356  0.3986748887728925  0.5974450426649445
  0.5650949465719141  0.7229837280815532  0.3311945107713354
  0.7672746379789815  0.5505474306040505  0.4017969191692456
  0.3867728917890946  0.4179634858673775  0.6953285290345769
  0.8122427843044006  0.5741438003121146  0.4943113576306875
  0.4349725595673197  0.4379802701159004  0.7895019883245903
  0.3903900743520591  0.2752304177228978  0.6602439261999883
  0.4322485430432084  0.3103270102257302  0.5318687379051463
  0.5318047501538739  0.2684927955238753  0.5689274584149411
  0.2044670561313900  0.7354643103750238  0.4601708177574600
  0.5345135746343853  0.4703627086833194  0.4758116831346457
```

IWV-Al-T3

```
Al  Si O
   1.00000000000000
    11.6056398920140573    0.0835597568938375    8.7649673366017495
    -3.6825601025723138   11.3529798124118475   10.3282290959204754
    -0.2346490665193920    0.2412826202978807   19.0520352385695801
   Al   Si   O
     1    37    76
Direct
  0.8991354326084272  0.6871251671910382  0.7035720294163270
  0.1385862987436916  0.2406399583355172  0.0498202621148972
  0.1229567092295920  0.1024503457275807 -0.0007504768296982
  0.0950337428078433  0.3256406499348036  0.2780620157528785
  0.2452023778771614  0.1457468653480126  0.5561172801260670
  0.1010473888277448  0.1200702313079754  0.7699473085604374
  0.3286060362674670  0.1130982666870184  0.2767575429595312
  0.0507112700125213  0.5694301402151234  0.1398284367187673
 -0.0055819175022472  0.7725291768264273  0.1255484167517376
  0.2776368587934638  0.3022560346013645  0.0931225071336449
  0.5533057376189970  0.0540673753185383  0.2462992992631099
  0.7708861740763512  0.0092647602547554  0.0998893719388496
```

0.2840836977286685	0.2823657061443749	0.3233739464276477
0.8469807959593039	0.7662631000952442	0.9401658127743402
0.8732732995292057	0.8992064233421234	0.9956365199019115
0.7386044356029836	0.8630720164095108	0.4403418297092324
0.8968446228175334	0.8844770287550004	0.2254438078616487
0.6722839683023856	0.8992368936044773	0.7044577747476405
0.9404136585243490	0.4430659998000123	0.8472096941635519
0.9953423508163058	0.2289987744434107	0.8757274787081850
0.7048715081875557	0.7082355380762090	0.8992080372935657
0.4428952860516012	0.9556126577074401	0.7433906981821933
0.2238349047205580	0.9922553803657487	0.8969404288593538
0.7010117944070845	0.7278564039518275	0.6703079929609040
0.9051128306301472	0.5264723344234729	0.0835182725755885
0.4927238743722827	0.9095727490609876	0.4870087541751211
0.0818744615070933	0.4849394037382762	0.9046201995308514
0.4871728410241672	0.1082609018021535	0.4973968895511118
0.9391493385930659	0.0644699215949685	0.4779823116815632
0.9059184066267267	0.0937179415636371	0.3180396208594630
0.1083956288778718	0.9142780521798182	0.5295007826417103
0.1196566185729105	0.8812357428899993	0.7081820971481965
0.4784502300020080	0.5199443902968968	0.9357740639440817
0.3179536468705852	0.6827842414826533	0.9062382208137408
0.5345508532653965	0.4531506077755483	0.1004055682663593
0.7111016857334459	0.2916862800561633	0.1186357376986485
0.7570135344242087	0.2704485706438448	0.2696327779854679
0.2681705507229146	0.7034775437194840	0.7572517796251559
0.1867891851644689	0.1811480595056021	0.9941563808679248
0.0214390292133744	0.1419574493342501	0.1886421194177620
0.2447095676508254	0.2769870167322049	0.0397216631073646
0.1015750512166280	0.3555889146328578	0.9778430926436850
0.8016820521551167	0.0010380962297466	0.5994625753543623
0.1785378006402717	0.1846504616501023	0.6317083999809835
0.1907837132797060	1.0023840687897054	0.6509702084608087
0.2207764283214871	0.2185828304569494	0.4590424721474698
0.3895914672160622	0.1778377468170745	0.4851447941455952
0.0408040310954706	0.7894080067760879	0.5949557291901459
0.9923922446696516	0.6356950091096653	0.1886232666728653
0.1887905435965467	0.6489797745454213	0.0217539124544681
0.0430639378126880	0.4382439705597885	0.2452290669164363
0.9791632257468633	0.5615025237838446	0.1033070274670199
0.5971975126049899	0.5991452862858273	0.7985315576838469
0.6341252045928700	0.0042513330432209	0.1826091743606248
0.6385844611799389	0.1578191616840518	0.1996294278382015
0.4637266447499757	0.1038533852827992	0.2117373688198719
0.4750864656105716	0.9506626189267210	0.3921299724533769
0.5923427860990771	0.5863809083567706	0.0316883537875854
0.8119217196455456	0.8248460060076385	-0.0045617414311104
0.9786654209313776	0.8473823952262803	0.8098015344127878
0.7477545907958844	0.7647403877978980	0.9267057516175198
0.8480958203750631	0.6307989357503572	0.0305042555302012
0.2351000898476487	0.9798732285364443	0.3955296874692245
0.8204263092913789	0.8312754680810581	0.3590639453761342
0.8109645009016655	-0.0158993713057136	0.3818209894822020
0.6998879426667287	0.7521640275176947	0.5728132640046656
0.6218881580656753	0.8861084810663982	0.4445975182310765
0.9795865060439104	0.2067091094385337	0.4002134996658042
0.0036723379070076	0.3658257653405202	0.8109649549206588
0.8156448101672789	0.3484632474591986	0.9843651756138382
0.9160091112950810	0.5478208637481403	0.7571233541652804
0.0283900952797312	0.4928553480572582	0.8474764712981858
0.4025333113240587	0.3927173369785266	0.2289918776431334

```
  0.3577114489303408  0.9857515405484003  0.8254817928267837
  0.3839882177547213  0.8227037026617475  0.8123159854058821
  0.5710063127767921  0.9686693537195352  0.7150613424113390
  0.4561445913750214  0.0473189355041576  0.6217086868943626
  0.3998147987168820  0.4140583824820063  0.9786177113524152
 -0.0074607599902255  0.5060505010825008  0.9938934411232541
  0.4849982619922910  1.0104064255540486  0.4916869860296578
  0.7958270195819882  0.4034855252070683  0.2127017858053689
  0.0538365396818162  0.1769999225110804  0.9433233382236649
  0.3879221507422218  0.7865304953734926  0.6188187244449229
  0.1801556382720034  0.0536130431923132  0.8158322680553189
  0.2111386459480341  0.5876856067940465  0.7974878591433244
  0.9336209939673746  0.8181675008413586  0.0610443394423573
  0.6182996012492877  0.2060756363903988  0.3883667606244934
  0.8230392847951952  0.9513854742003481  0.1735015630997897
  0.0251266896190061  0.9788072420894409  0.1379738527203016
  0.9492335278529473  0.0509732479070785  0.3958264879990055
  0.8431781614275240  0.1978914728384575  0.3034570929029355
  0.9766853942051614  0.0203772800215588  0.8572639547505860
  0.1432501515062201  0.9021824947020903  0.6029744925623486
  0.1710998923688384  0.7761266974085177  0.7726652031957350
  0.1322983654255836  0.8583243219673674  0.0240673204416298
  0.3948333659842143  0.6029870120537929  0.9503818685128677
  0.3020880572233707  0.6579037931070834  0.8429709026885621
  0.8558351917795489  0.1463082789815453  0.9748637948066116
  0.6195408467016685  0.3702914633331469  0.1257753937727109
  0.7728136740761623  0.2789246686408592  0.1729474506562586
  0.2264198296225595  0.0718299099590589  0.9223744089124113
  0.0705977407897178  0.2228373824858530  0.7737329372413929
  0.9203543514472091  0.7757870328284047  0.2294153330373563
  0.7711615753663136  0.9335476531863013  0.0695137187627668
  0.0290081376716325  0.0082932706363019  0.5105262432212608
  0.5156188004196914  0.4566022076948058  0.0223509482980259
  0.1666794850305637  0.3330788936229401  0.1667279902936089
  0.3236870395988710  0.1855552051707257  0.3114837999108060
  0.8107475219714070  0.6823241164276874  0.8220927847354842
  0.6582644714906976  0.8335308298457687  0.6706403339766531
  0.1880201067928075  0.3313168960575274  0.2972936125074113
  0.2986139963956912  0.1846117999640132  0.1838374194323658
  0.8286383439140682  0.7284447735714736  0.6405134145916774
  0.6492495251307785  0.8030385815581893  0.8315844613769210
```

IWV-Al-T3-DPDMP+

```
   Al     Si     O     C     H     P
   1.00000000000000
    11.6056394577026367    0.0835597589612007    8.7649669647216797
    -3.6825602054595947   11.3529796600341797   10.3282289505004883
    -0.2346490621566772    0.241282626986503б   19.0520343780517578
   Al   Si   O    C    H    P
     1    37    76    14    16     1
Direct
  0.8692202219406513  0.6800888740625938  0.7464427586637105
  0.1136429117787975  0.2405184984861936  0.0885271048763807
  0.0997923243652799  0.1004240752455536  0.0374359613820840
  0.0742418998250127  0.3237311907262266  0.3129232308823119
  0.2217887765062808  0.1423619591729620  0.5936563057789358
  0.0821954692934129  0.1238379544741541  0.8023473864640515
  0.3001416392818247  0.1040450023589155  0.3156390411116452
  0.0226802123194999  0.5623101998807379  0.1766815503519305
```

0.9785800520949677	0.7735393981445615	0.1547669912743068
0.2540766746143047	0.2971732134973220	0.1311305887650603
0.5316314406067287	0.0577349963653879	0.2708533898581554
0.7498425943005796	0.0083022607923986	0.1327638767241908
0.2636474835056614	0.2761158380492280	0.3585765517735262
0.8219609538031505	0.7601164164279411	0.9794086657045137
0.8558271685026266	0.8995540157462620	1.0268469926092270
0.7168457272613022	0.8608803162456599	0.4738965127164685
0.8748406844413399	0.8822920065385288	0.2597323780331893
0.6511380239156507	0.9003177310356915	0.7399758727547652
0.9287947759273791	0.4450809672713356	0.8847171475059982
0.9735118284045952	0.2283669145399467	0.9115222110660821
0.6755663687719498	0.7084965274180249	0.9379549902200137
0.4173992865100493	0.9476539351669224	0.7888900370536738
0.2060417523374285	-0.0031077379782007	0.9295976645610535
0.6758601761493459	0.7239533040699981	0.7078067349969174
0.8686748756964612	0.5162025630137175	0.1288867880232079
0.4731711985123882	0.9096866068935339	0.5157281495458400
0.0627377974457860	0.4869766619124533	0.9424441205407972
0.4579099651434407	0.0921604485488982	0.5467255141384356
0.9159728676309112	0.0619886452094604	0.5165699304792515
0.8818567812905177	0.0885034744146150	0.3583606408146928
0.0819203831890026	0.9096738084852606	0.5694020586941380
0.0975334017291397	0.8761553922584578	0.7458610530030140
0.4538282778113328	0.5139367332372561	0.9757075044167857
0.2970317131626035	0.6789107529869102	0.9449791783129053
0.5161185949648015	0.4539793499200911	0.1335322254353617
0.6883298861192269	0.2893986323345585	0.1567416360979909
0.7204906837967551	0.2525020934152473	0.3233313605989785
0.2583140912301332	0.7095995757732972	0.7852370655089440
0.1593974128523525	0.1818687915541612	0.0322804590669605
0.0032898079562667	0.1394839457208274	0.2299204112290347
0.2250826625834303	0.2819513264660960	0.0694887511962316
0.0688460882432886	0.3518379181428850	1.0237828772211079
0.7804010477159555	0.0002547523313311	0.6408391512822638
0.1588614667984806	0.1871365428689429	0.6644390259008125
0.1574794776175043	0.0006105631690399	0.6916748008540354
0.2034209998275760	0.2162950489855535	0.4922874274927507
0.3646491959793124	0.1668496622645453	0.5298967007979365
0.0082188914770026	0.7872750576938747	0.6353540415234136
0.9777944017520965	0.6369790587646789	0.2168129749357526
0.1551420627779668	0.6377220872088598	0.0488894082044557
0.0240285319106770	0.4374485566291053	0.2805695531171605
0.9329621953920493	0.5400492179963617	0.1630751388515266
0.5631737927443483	0.6017590035175637	0.8343170554814782
0.6120807050760405	0.0018516627275076	0.2138107080358113
0.6179789589317431	0.1750452327309691	0.2050045606116397
0.4340605628437882	0.0946307051313394	0.2464359244713404
0.4612026014087692	0.9604910741682346	0.4149303399999802
0.5628884096333047	0.5873424413926008	0.0717093497787490
0.7942768339646950	0.8271508633865893	1.0253403141551352
0.9560163423352117	0.8304298309856391	0.8528900406385493
0.7245689543084831	0.7653507534239748	0.9614342490765155
0.8115160690266280	0.6222930408999399	0.0786457096599862
0.2080225239169581	0.9704783270969329	0.4367887774588057
0.7969068681344251	0.8249623504566362	0.3954844760852769
0.7926459290353139	0.9819336475101124	0.4124100194169058
0.6782418724935584	0.7523014861581075	0.6067208882172156
0.6013674900110457	0.8841813968849281	0.4764884626800873
0.9574601385831799	0.2055388702262026	0.4337975926244704
0.9832485354814874	0.3645857753782804	0.8504812614318806

0.8153041758773726	0.3534862791055140	1.0274015341994602
0.8932101564599879	0.5418928787305536	0.8017802750982603
0.0294461710910736	0.5052695440537350	0.8663837588783890
0.3825840940820672	0.3866730874253982	0.2626223331352772
0.3422783061022751	-0.0031378627183509	0.8541028256664257
0.3569175960750811	0.8054071008724719	0.8862517499669745
0.5531822588855119	0.9741455526446822	0.7421190421243642
0.4118521840715341	0.0113218203805668	0.6814906095969244
0.3817023906897186	0.4009129337422158	1.0225506914849976
0.9625718972231072	0.5078898868018243	0.0298740322435113
0.4639517308551996	0.0069318368005134	0.5249624867509963
0.7593168182727322	0.3918890365321561	0.2517548560443297
0.0340384995343862	0.1714952723391939	0.9772762948381716
0.3657983126526874	0.7856093975418615	0.6437447039820362
0.1622824854602866	0.0644835595486153	0.8477454184165580
0.1935756468232708	0.5804703394647615	0.8501877446700261
0.9146098838885736	0.8172673375474735	0.0935858279908161
0.5887181557965028	0.1859579974781345	0.4523038123297344
0.8007990775168327	0.9480217047943595	0.2080255251400853
0.0002928498245248	0.9777314340384933	0.1764424250786131
0.9172011626860411	0.0398044239914734	0.4435801756636357
0.8178832361725746	0.1917913502269525	0.3443279778839129
0.9606548986013859	0.0210688676580701	0.8891334641856240
0.1196623396435904	0.8956315608457384	0.6418416020005331
0.1594334995310757	0.7800462921687142	0.8022965952513511
0.1159447212985914	0.8608231059683464	0.0515492720094438
0.3585829211976268	0.5844486266670716	1.0031686887977940
0.3158940252410635	0.6901202665075569	0.8454979159536929
0.8339375071172134	0.1467351362741370	1.0099916019088120
0.6073605032820801	0.3787194462283479	0.1537614155782495
0.7149710316432039	0.2392719906017118	0.2483104138391193
0.2057821997270585	0.0676604966253212	0.9642731926128001
0.0457713817985746	0.2274859348053924	0.8054232055965910
0.9053168078601408	0.7759828230214421	0.2596875045686278
0.7538713123448891	0.9353342746720342	0.0988224492003724
0.0082365276060888	0.0089626863460829	0.5463902615792418
0.5047392824719122	0.4612784836963458	0.0496133739672656
0.1457269761556772	0.3330008232259879	0.2006372397277828
0.3005915091247991	0.1792522972628029	0.3465194955001071
0.7769013742567288	0.6808162797093221	0.8605661577428758
0.6450620487263510	0.8336014098648429	0.7038238415388803
0.1660010649240257	0.3264661633001135	0.3347192542858557
0.2626385885424560	0.1724731290820375	0.2286495898529082
0.7999531005531499	0.7083493419823534	0.6853493583340724
0.6187214411625763	0.8045939170132896	0.8718038499228252
0.8391599949707191	0.3214669390599852	0.5575522108680093
0.8752203416758709	0.2031918641302739	0.7033952162321392
0.8947947154531368	0.4260005157609416	0.5115263251955307
0.9307336210869794	0.3072680969883976	0.6584395445821706
0.9406656199205209	0.4186473168555561	0.5623670112273319
0.8296995008833364	0.2102586741567287	0.6530101634430758
0.1355745309568380	0.6412812708119037	0.3412138895774754
0.1812866746851571	0.7654121424901175	0.2613972104058617
0.1876744129185105	0.5836329475869486	0.2998494837510732
0.2797966504771855	0.8308027116525125	0.1412865589961703
0.2861860284183411	0.6499523995775327	0.1796000516570734
0.3327605222399869	0.7730451632180912	0.1007292752346952
0.9146135845457037	0.6369917792406755	0.5191756909405303
1.0771391557558485	0.5212237256906948	0.5709505436239555
1.0045448180400292	0.4791747190457565	0.6639979311325923
0.1323365803671950	0.6060039626157200	0.5218768565319392

```
  0.1327321904312782  0.4624788700930207  0.5692051564810774
  0.4107606255809710  0.8238088520143606  0.0073410613191304
  0.8052044206679392  0.3266426290688598  0.5173132436584389
  0.8690163169789608  0.1168348357408255  0.7770540494370819
  0.9048165894618690  0.5123912300586498  0.4348665642169722
  0.9670435599428142  0.3014559442234027  0.6972665700485630
  0.1405591100301325  0.8113529682379568  0.2920901413512333
  0.1511916372408298  0.4877002449750865  0.3609314915882689
  0.3148258290927546  0.9271609234714984  0.0791292321935809
  0.3270286037789500  0.6053624461211073  0.1475400569610669
  0.8698675517238474  0.6530750456145076  0.4826768780856859
  0.8480059977995144  0.5862624453839556  0.6153482629712153
  0.9631639972934675  0.7226822740861165  0.4742386386820059
  0.7877606279595357  0.1291125868776640  0.6869801166088412
  1.0165735713411463  0.5541042962785961  0.4984604015787922
```

IWV-Al-T4

```
Al  Si O
   1.00000000000000
    11.6056398920140573    0.0835597568938375    8.7649673366017495
    -3.6825601025723138   11.3529798124118475   10.3282290959204754
    -0.2346490665193920    0.2412826202978807   19.0520352385695801
   Al   Si   O
     1    37    76
Direct
  0.8920886062801078  0.0942210430987572  0.4640942264680613
  0.1509285947014445  0.2332785229284775  0.0597792966601064
  0.1291061709376191  0.0975145604832598  0.0072478870944329
  0.1031139185444635  0.3193901531503749  0.2893743361618936
  0.2559461059942754  0.1362393333170958  0.5623796228654461
  0.1038814663940232  0.1140590892395310  0.7776527577650836
  0.3272550849357982  0.0998335709391905  0.2973516177022055
  0.0614285843179687  0.5575600952333258  0.1506389281992840
  0.0072868455010264  0.7683301498311892  0.1263803991903852
  0.2915522042651009  0.2947428153050511  0.1020648266892423
  0.5614445617174303  0.0471087539582583  0.2533718404627741
  0.7784784275540634  0.0047852226809296  0.1047833390551079
  0.2952796317113180  0.2729701591462942  0.3300665165728764
  0.8618542780104755  0.7542402278501081  0.9524093769546055
  0.8792915803544082  0.8932824201523772  1.0030871161178876
  0.9063239100134075  0.6749875316657242  0.7211743746963752
  0.7542300497773470  0.8538140347384896  0.4447579226330411
  0.9001492630859365  0.8766571461199899  0.2329658076100130
  0.6732052931483308  0.8890003781206387  0.7241255776308710
  0.9481291255692611  0.4315376213381131  0.8618092636786259
  1.0075587883152222  0.2259709500968627  0.8777101404899159
  0.7198387682649807  0.6959158671929633  0.9097257109338940
  0.4462803319378285  0.9459620185605352  0.7521980252822417
  0.2314526013424902  0.9888809831386541  0.9016080641280807
  0.7184963238470358  0.7169935401593200  0.6777620286210106
  0.9202263797127401  0.5110527934005865  0.0978168121911887
  0.5120988173422534  0.8908122995434462  0.5023404474569726
  0.0954126607280114  0.4722071104355366  0.9173680855049124
  0.5062749089665421  0.0921383053359532  0.5098752073084911
  0.8755676439796879  0.1229016512941815  0.2875894941928398
  0.0633988560004620  0.9384882754237972  0.5178427722884494
  0.0934156157248238  0.9056214967952354  0.6812097922687507
  0.4644009332468523  0.5487705973772877  0.9001708382886386
  0.2889341093681919  0.7107668903530575  0.8772599233015549
```

0.5178058567563971	0.4781769376741132	0.0672358759218038
0.6801537399889779	0.3199809875761794	0.0921154399905321
0.7314789759373150	0.2970168792336610	0.2382373719659449
0.2430622725073097	0.7292956208838681	0.7273885493369566
0.1925592793529962	0.1710375215172914	1.0069451559159714
0.0166650336269499	0.1559880880071036	0.1842282295856504
0.2424724169965166	0.2352172929919351	0.0815745566145724
0.1583771481601967	0.3702510929326192	0.9617121348486218
0.7629605837063959	0.0208777498105683	0.6102265451371898
0.1801233755591435	0.1683168215946651	0.6437076946515700
0.1846947486027911	1.0112139312703978	0.6267240134652426
0.2772766200773350	0.2449644961835716	0.4370681739308333
0.3809632204845698	0.1235188863611432	0.5425126034830033
0.0196326944195322	0.7947023380914182	0.6010538631912229
1.0021126795303490	0.6308025281056774	0.1916558911435886
0.1826544988386026	0.6508941518334269	0.0129685009415028
0.0971658653043272	0.4544739494440983	0.2336189301545303
0.9670291250590837	0.5018175200653212	0.1610438800584939
0.5981187621280994	0.6060287885551421	0.7751044193975176
0.6452577389900609	0.0136749610394324	0.1740969638183868
0.6222650847691751	0.1778551638911672	0.1870634019824217
0.4369050921005650	0.0425273356196337	0.2737054100354847
0.5421554413677505	0.9527922804221106	0.3780792070887967
0.5989275066232337	0.5821019059661172	0.0237669164807789
0.8168957022973169	0.8152042402739684	0.0066392978069676
0.9754941367032434	0.8522978110402191	0.8121059396884254
0.7531294684255939	0.7141390504342413	0.9675558481879388
0.9027836706692455	0.6413216874348202	0.0222905646557156
0.2054635373591120	0.9902703015461822	0.4036317904902106
0.8218758466747398	0.8122895312688312	0.3711091437120015
0.8081248173391373	-0.0031391854837531	0.3484762696865926
0.7762583716043860	0.7806263884207321	0.5434800434802138
0.6098694888705488	0.8217662399807141	0.5143189782934660
0.9715196835066110	0.2311936180557698	0.3887623495997287
0.0083390352856573	0.3623009940597264	0.8163108254278748
0.8074906064516335	0.3576769449768498	0.9734272175822012
0.9624895460969644	0.5644594180079753	0.7530702916017519
0.0108265097955971	0.4354594404767825	0.9073192957268991
0.4005814769865558	0.3999411901332285	0.2059669599075389
0.3677729524696667	0.9948733814525845	0.8176362752485972
0.3563173686377270	0.8449162709870746	0.7992709537693856
0.5336814880201369	0.8909602074684563	0.7881764319234768
0.5195772095118408	0.0515851857354619	0.6070577948532307
0.4026334058110435	0.4158675535485667	0.9695405446671342
0.0169253328447463	0.4902478697603920	1.0097967207854870
0.5102515953680412	0.9872785313362961	0.5108421288577575
0.7922419751697359	0.4087731685319174	0.2011035633360492
0.0699732542498226	0.1804174914639340	0.9407684433395368
0.3821023848836860	0.7911458406709171	0.6101935296834214
0.1778929227279218	0.0474739972013289	0.8291920736958914
0.2012841638180846	0.5962569254448608	0.7856978404697984
0.9470373125494224	0.8167406431439925	0.0608067417403626
0.6135559735467696	0.2126825213387750	0.3775739919003371
0.8208183582490948	0.9417613185169499	0.1870885501412071
0.0245075188561518	0.9767287055937375	0.1451411449793825
0.8524106336710847	0.1164806812578500	0.3835876894486033
0.8245342068956160	0.2236264566501052	0.2169707052559123
0.9771471063253919	0.0176585957984812	0.8649654530313511
0.0559373291649308	0.9551450079550111	0.5978663444399261
0.1584658873089067	0.8035856162433012	0.6924846161629474
0.1465581549627596	0.8512868997482574	0.0261887900315669

```
  0.3823892935474959  0.6338462490411396  0.8707310732075121
  0.2277396061442936  0.7205697337803859  0.8240938129484889
  0.8703785451478975  0.1388954027940544  0.9789300542217637
  0.5958403707504840  0.3913484555156205  0.0546097177694173
  0.6942559446547494  0.3505411028006036  0.1525415342442338
  0.2308543449556859  0.0626414781577064  0.9338387084758415
  0.0801907010688292  0.2226748516886874  0.7738241892454757
  0.9320341442252514  0.7740704937258898  0.2285226247048562
  0.7763160502152204  0.9251741987961422  0.0790690630131679
 -0.0143359101546374  0.0029206924442040  0.4739824660343118
  0.4750051518035153  0.5400523635386025 -0.0147486324766723
  0.1874584439938059  0.3276712798802270  0.1757190695736716
  0.3350801121551401  0.1648857018378871  0.3347655052937757
  0.8300357678305998  0.6670613949957584  0.8333715036915128
  0.6834114317174385  0.8121793895107674  0.6912024890511286
  0.1730025025234620  0.2819800116426383  0.3449901755620413
  0.3423641878073990  0.2006549857302639  0.1740265981506507
  0.8161161495651388  0.6646037872220425  0.7011143663727668
  0.6939465264379476  0.8133650488690900  0.8251744314295334
```

IWV-Al-T4-DPDMP^{+}

```
   Al     Si     O     C     H     P
   1.00000000000000
    11.6056394577026367    0.0835597589612007    8.7649669647216797
    -3.6825602054595947   11.3529796600341797   10.3282289505004883
    -0.2346490621566772    0.2412826269865036   19.0520343780517578
   Al   Si   O    C    H    P
     1    37    76    14    16     1
Direct
  0.8801002449752237  0.0942826156531597  0.4739623562443770
  0.1374344648612024  0.2464244910060878  0.0618256978396537
  0.1170618297663600  0.0988930261430099  0.0177331236538242
  0.0860968039511012  0.3162522470035299  0.3073431749344112
  0.2348056837481310  0.1368067608606462  0.5761530110764754
  0.0975006052915043  0.1206107018531969  0.7838113007598786
  0.3130626307855492  0.0986661490557784  0.3090219453867748
  0.0466862203632911  0.5536647823540839  0.1626336812508637
 -0.0025582552952869  0.7703983682660608  0.1324875085966919
  0.2721185145530410  0.2927380623653702  0.1180454385049380
  0.5483843845140847  0.0450093059123494  0.2634930115065952
  0.7671098593501965  0.0039411283867731  0.1137779146060853
  0.2826595073250420  0.2743616631197228  0.3402867301951971
  0.8488856989759815  0.7456934503545029  0.9666537522865333
  0.8742093879309936  0.8976708956778501  1.0053155450967508
  0.8927320812699010  0.6725634313690619  0.7331102600451871
  0.7517348594873485  0.8493720569006294  0.4536120926090481
  0.8900395702941662  0.8761695092844931  0.2407572658412851
  0.6594680156101795  0.8845494286693936  0.7329134657819976
  0.9329778122969644  0.4311706692613335  0.8752174414768430
 -0.0059611750067145  0.2264576711558571  0.8895323500459911
  0.7076069158057702  0.6954432341008849  0.9191101546674177
  0.4289464904931968  0.9425506893117367  0.7657734633434501
  0.2208187078081103 -0.0087218296627365  0.9118913983371727
  0.7048614217726398  0.7089700083294849  0.6913206490577030
  0.8992616856626915  0.5002753043756123  0.1187921197469393
  0.5050424397603152  0.8881248699364244  0.5104291057714933
  0.0809137691329675  0.4798959343127903  0.9242400097618599
  0.4825483986432358  0.0843332572818743  0.5269294657024189
  0.8612463565843285  0.1228308088027614  0.2988425768049982
```

0.0484771815604993	0.9361004980685371	0.5299117269356310
0.0807827552808084	0.9064676659724651	0.6905512728207243
0.4501338531589809	0.5487018882133263	0.9116936798655294
0.2749829754175082	0.7090055036301482	0.8872911847544179
0.5040466023523248	0.4788506157624900	0.0774854604769992
0.6637248365608686	0.3181604911744217	0.1044398222076218
0.7051125065387807	0.2850629819202475	0.2600992271195995
0.2383174977612882	0.7385949679168755	0.7274178636675946
0.1766318086600066	0.1766095389434814	0.0164130608588848
-0.0014333363775216	0.1830425666427172	0.1772209295238621
0.2224331712350218	0.2396579153096990	0.0931267176761694
0.1569511573473367	0.3868511285150532	0.9559323811835740
0.7477791514324401	0.0181969395817714	0.6201902203710802
0.1762541212893085	0.1802399394410865	0.6475151450529136
0.1352884232304940	0.0245375870563489	0.6482862273758102
0.2712735777908463	0.2476697191519202	0.4439900779207406
0.3492075389179559	0.0960979258403900	0.5706084999115628
0.0030739488693770	0.7940321848999032	0.6090095436564187
0.9928864590694376	0.6330834931808798	0.1969410130396998
0.1672917128253911	0.6402247676378598	0.0254520387945803
0.0786782701070051	0.4515343031442358	0.2500710949326088
0.9476846112359636	0.4945055121654878	0.1797182076480346
0.5831741632693543	0.5996048659394638	0.7887061150601437
0.6327381236466681	0.0103025668354462	0.1850813239132069
0.6106247637735903	0.1756789840062438	0.1964832162484224
0.4241289495414048	0.0433211107609983	0.2827006276892257
0.5270182774727544	0.9498546692012326	0.3896111746285211
0.5881435644785368	0.5798356795613152	0.0349636821264099
0.8168512113773112	0.8230678507482302	1.0027298708395289
0.9740481894291877	0.8173313269245482	0.8333743300128198
0.7409719247358685	0.7208336584502592	0.9724815362977204
0.8636326231922208	0.6223970522798469	0.0588749678986941
0.1919434365344751	0.9872902436927048	0.4194017126693002
0.8135718514007630	0.8083121611740063	0.3796340997714434
0.8194583459000719	0.9882887327507178	0.3599145442619009
0.7650872772174168	0.7656581753721632	0.5588336295412647
0.6099456024004509	0.8285000437123157	0.5133484225559674
0.9522629184158956	0.2282458638463154	0.4108788987982683
0.9940902557264907	0.3617948052092046	0.8300762557741878
0.7914342799562171	0.3605138837916602	0.9833306496717500
0.9529025289842753	0.5649541430922834	0.7635868363950289
0.9922540087617667	0.4316382600300865	0.9250768474799027
0.3841612497698352	0.4030160865031011	0.2147353463826440
0.3561771178406182	-0.0069684823044298	0.8307071258281289
0.3338776670845435	0.8421881269965346	0.8176600986778788
0.5203816065012449	0.8879602286517386	0.7960854375980294
0.4980395822016959	0.0494998110032230	0.6211814249282164
0.3823912439228888	0.4151270893177263	0.9872272374629714
1.0026168556580120	0.4924476990703602	0.0211176983718735
0.4996792959807754	0.9819018573323884	0.5228531711856471
0.7786320937991652	0.3883776641573436	0.2240827059948182
0.0563959353675099	0.1791424592045791	0.9527273361357277
0.3806101149023833	0.7783711162940058	0.6235093039168241
0.1706297665558519	0.0533546342886008	0.8363435145311044
0.1749494390635408	0.6097429372335674	0.7901702671329822
0.9412776126878936	0.8194802232446099	0.0635343584625101
0.5801736900270194	0.2123200029208145	0.3954343357955744
0.8104275835390471	0.9424037808199459	0.1954332276596839
0.0150999805788230	0.9748687130809479	0.1539247552938454
0.8455700854423971	0.1262960001979166	0.3866283330455548
0.7811917854675837	0.1955875023052301	0.2539689273683542

```
0.9723199302231044  0.0237468809007837  0.8686383113430594
0.0322150978418623  0.9448107147462519  0.6183012234202578
0.1821802342005546  0.8395038582168555  0.6669379277129821
0.1351713828273282  0.8543095493145868  0.0360798048421677
0.3736530832619491  0.6382809090000114  0.8755712235089678
0.2174563012477593  0.7228784422294836  0.8298313272610192
0.8574774261943542  0.1392429180013019  0.9884016142606245
0.5772719846722818  0.3875850873030490  0.0693148986404823
0.6768515125739517  0.3475029570772113  0.1663342623210373
0.2226808555205613  0.0669769474245018  0.9419265292100962
0.0699080770010405  0.2270090156529349  0.7829196759771926
0.9181418997575047  0.7727708304313493  0.2370591931513186
0.7698213170838871  0.9263866627099515  0.0843042976269696
0.9759948804977652  0.0065044735548145  0.4804073030791378
0.4644823267084976  0.5449402446384628  0.9922937380911365
0.1667831624438061  0.3207375776523555  0.1967358304095370
0.3216507350840287  0.1671052810434075  0.3433485625294949
0.8191945794636423  0.6667351104429909  0.8436535499908873
0.6729482284801094  0.8108418405763601  0.6962281012385225
0.1561357850731491  0.2803890588791694  0.3623328933338672
0.3232393640630045  0.1959686173008205  0.1882081078553205
0.7983613693063936  0.6551972501229280  0.7202888983602169
0.6794610495489009  0.8096147073550607  0.8339714911076712
0.3707131499336628  0.4219943957115088  0.7102653207777156
0.5464932928837688  0.5925809251465457  0.5551834010628127
0.4437415073278920  0.3523075742292072  0.6975550882294326
0.6204577812016728  0.5242166556207004  0.5410964707964083
0.5687678369801952  0.4036719927914092  0.6125855539033087
0.4218277856741727  0.5416128977056207  0.6395785096310045
0.7453926944455923  0.2951214072390081  0.6471858018490194
0.8373675050680348  0.2421345921044937  0.6269852411002675
0.7106943978825969  0.3228419126604752  0.7143510593915594
0.8937194173597122  0.2164914158594991  0.6754252046479882
0.7681762185883459  0.2970026663146960  0.7619175339888506
0.8588861134493773  0.2435648086499675  0.7428704963122190
0.5791903296562479  0.1733746020555771  0.6804528263415525
0.7670222627356051  0.3884269174727552  0.4415294479634306
0.7161002401127547  0.3908281567388525  0.4151306411948298
0.8301895391671842  0.3374986236048397  0.4309540590724080
0.8162280807491226  0.4794974422322841  0.3853297050795490
0.9026405558053786  0.2228952909628313  0.7809919702561202
0.2736769815155804  0.3822515549625325  0.7766470839398811
0.5863712796113754  0.6854020737899986  0.5012132675501755
0.4031531721904545  0.2585655471013843  0.7542646555881032
0.7176699098124770  0.5646428568012402  0.4755203891748114
0.8645495476795958  0.2206272048729741  0.5745977175021264
0.6394442247645759  0.3640851946074543  0.7293187875131363
0.9647383825942945  0.1749546864467679  0.6607964202801619
0.7418859922810247  0.3183939405867161  0.8140794487311018
0.5167026684928692  0.1230641311683944  0.7764295100958074
0.5293306304795287  0.1856122540286452  0.6495772369130758
0.6420249691563573  0.1243108041337497  0.6640734497886387
0.3645506835691395  0.5951558137389530  0.6510070172678829
0.6649667798492994  0.3162681582079545  0.5954799669544428
```

IWV-Al-T5

```
Al  Si O
   1.00000000000000
    11.6056398920140573    0.0835597568938375    8.7649673366017495
```

```
    -3.6825601025723138   11.3529798124118475   10.3282290959204754
    -0.2346490665193920    0.2412826202978807   19.0520352385695801
   Al   Si   O
     1    37    76
Direct
  0.9079894113295031  0.0946586601872903  0.3198895663608007
  0.1447474275658239  0.2419849228805903  0.0459634319866483
  0.1230002963450399  0.1071244054930593 -0.0047579335931576
  0.0931993122551550  0.3238227223581965  0.2790449031603272
  0.2486998289738534  0.1477969909269348  0.5506707202449869
  0.0988962370232120  0.1200523475806857  0.7689884175763032
  0.3198975736166375  0.1082693996666303  0.2834013715557374
  0.0480706777414137  0.5666859870514619  0.1406485203237029
 -0.0071403375811991  0.7723285159361909  0.1241007285251639
  0.2778824223547144  0.2989147725774053  0.0943645853832646
  0.5524131317509710  0.0523599501179408  0.2477673225821289
  0.7682181647154571  0.0101690593212233  0.0990495275305865
  0.2826438348136826  0.2874104070779010  0.3194489882266166
  0.8507701984442653  0.7728934041154412  0.9342721131284275
  0.8706000283205674  0.9000383759985845  0.9952645318190436
  0.8958427898083208  0.6840027627817638  0.7104755157544922
  0.7416664414216125  0.8624602334483722  0.4398653386564375
  0.8953393696851557  0.8864959720501415  0.2232919690805500
  0.6730285471617877  0.8992830990384555  0.7034587495058071
  0.9419525689953117  0.4430472804636267  0.8492431309975315
  0.9937281001815742  0.2310477854608086  0.8730913456508239
  0.7075510954309756  0.7081919139608591  0.8980655114270962
  0.4398989924110664  0.9530252592056607  0.7447416850193362
  0.2216499627632657  0.9922422873802750  0.8962134442543234
  0.7037501426921738  0.7257675244481027  0.6701560264358912
  0.9075395718160725  0.5299075001780457  0.0772561282603960
  0.4963391616909147  0.9091371046708453  0.4889769498305829
  0.0874633114198355  0.4863162847549167  0.9007410463530594
  0.4928413788236559  0.1124655416183512  0.4928776016655081
  0.9368113114751973  0.0591625468540228  0.4857674962122578
  0.0984546799880316  0.9083573169001539  0.5406229237762257
  0.1190217541849703  0.8790552359523895  0.7105682373348930
  0.4814266754526729  0.5201280858602395  0.9348867749831116
  0.3163288421714759  0.6790347446777746  0.9101173155012661
  0.5388228152644713  0.4532925754085628  0.0988367722222182
  0.7110334022873283  0.2893920783649478  0.1231921582630937
  0.7590917879847623  0.2736060454534079  0.2715468343471680
  0.2636752365152036  0.7063403990397472  0.7612640461653122
  0.1922455160353519  0.1855753142898866  0.9862464464215599
  0.0327948421142719  0.1459076322167434  0.1823960425858849
  0.2554743615603651  0.2840680547042087  0.0290706407891679
  0.1131395851109520  0.3609214443785806  0.9679632065866004
  0.7972394213707527  0.0032650139428842  0.6055829191009134
  0.1768428827980825  0.1797996823671326  0.6323651242003610
  0.1998959944176525  1.0058646592279257  0.6348878456220799
  0.2245606503168097  0.2265314044226241  0.4528606097369439
  0.3919409052964887  0.1783443734991529  0.4830960452174117
  0.0268105929615745  0.7841161186966612  0.6022557397604247
  0.9893704579690669  0.6349168836242953  0.1875250972315349
  0.1888619917484035  0.6415186467019084  0.0285840159247656
  0.0345299896270695  0.4325714681382209  0.2478668156809750
  0.9837149631740484  0.5654511145087764  0.0952271616374055
  0.5962101003272826  0.5998743566361503  0.7950569917807411
  0.6311026520643338  0.0060627796050248  0.1798713181121374
  0.6414424519229324  0.1604480867237194  0.1923997039993651
  0.4535282702882157  0.0944682795091816  0.2250601674734299
```

0.4838550635210709	0.9485820302858061	0.3915088421131970
0.6021622031594658	0.5844650560146395	0.0315127910162202
0.8076872236201919	0.8252587346098648	-0.0038553476828683
0.9809828802870476	0.8617314838454125	0.8036884624700028
0.7556727500615426	0.7718549683796863	0.9157136249784630
0.8574955178594311	0.6390970548286636	0.0171070607188260
0.2218869724054917	0.9759270062462759	0.4054095009391838
0.8188139317483956	0.8305471732247584	0.3574869181055467
0.8021175994567077	-0.0128959074450270	0.3798231779177368
0.7239000227464074	0.7509450996573120	0.5642536943430334
0.6102107354415720	0.8596101861276038	0.4701953645233554
0.9846424784275907	0.2012698492250489	0.4059309479061023
-0.0044890111280697	0.3682973925408863	0.8094182371256087
0.8173268246564004	0.3559733854966439	0.9845589516913574
0.9161005829577314	0.5549714286251054	0.7594490292598388
0.0376674262896625	0.4950417384255670	0.8396441887337414
0.4036963230253615	0.3966487086136554	0.2221869844451152
0.3559419326030934	0.9864929342155596	0.8239219946432477
0.3791399808079672	0.8197919131233939	0.8156658309216951
0.5663914341470085	0.9609116529981906	0.7207516807104447
0.4555847689521775	0.0435742510837485	0.6220887913018230
0.4021028684772440	0.4080873485113584	0.9867646727658286
-0.0080691737615579	0.5044679519267978	0.9913031224633155
0.5028805343555830	1.0204623617738824	0.4770936356170480
0.7926929917219325	0.4132704232715644	0.2047424454008409
0.0563273460696301	0.1852137453226665	0.9358482380235059
0.3779032176322017	0.7999754615689745	0.6214839112382502
0.1772369310714351	0.0538936564921894	0.8155467488733162
0.2119060015895637	0.5955187138573494	0.7923122228412386
0.9311315543997142	0.8185034830848270	0.0601016670223702
0.6184140888327708	0.2155929936432137	0.3887794096202720
0.8190194792887322	0.9510935209115968	0.1735828564357291
0.0224393397978072	0.9841251709048231	0.1332085469561023
0.9500292693727205	0.0371683366867190	0.4102190131557971
0.8456819358664271	0.2093158402723885	0.3037520520446887
0.9738573856326337	0.0209949958295562	0.8567077116411591
0.1342990425916953	0.8757848379632710	0.6209243311748102
0.1620234141284215	0.7731996789627021	0.7859334157356707
0.1314644400008415	0.8571843669518023	0.0217903973875082
0.3981167959775534	0.6032185832656848	0.9491379651685677
0.2989697052494638	0.6537663301176532	0.8477897811354197
0.8545780595333717	0.1472314045213694	0.9739309618199411
0.6173306944252690	0.3660230229159195	0.1313458432316196
0.7679504228975451	0.2720981371336260	0.1818859891482512
0.2234299720732921	0.0721397113286572	0.9212248549594033
0.0682460893778396	0.2244677063833420	0.7711384842115608
0.9209529757156351	0.7785824635337463	0.2255843245478224
0.7685631369211704	0.9351529255903404	0.0680018350596626
0.0145636087933851	-0.0046940269873379	0.5358828329121714
0.5313617746119706	0.4670430015090717	0.0098834030887855
0.1667736549761862	0.3292213797390824	0.1687066257175735
0.3145378949209317	0.1858733165410179	0.3134166674249961
0.8174380577558750	0.6812552003271463	0.8194948252445939
0.6679153929352255	0.8348130593164772	0.6647042669355728
0.1884320359051479	0.3405970246560262	0.2917856890779850
0.2946371531643287	0.1771959420992467	0.1874879487122741
0.8247826992798437	0.7130890936224399	0.6592902728934433
0.6504397909325531	0.7950018838268402	0.8310626156404067

IWV-Al-T5-DPDMP^{+}

```
Al  Si O    C    H    P
   1.00000000000000
    11.6056398920140573    0.0835597568938375    8.7649673366017495
    -3.6825601025723138   11.3529798124118475   10.3282290959204754
    -0.2346490665193920    0.2412826202978807   19.0520352385695801
   Al   Si   O    C    H    P
     1    37    76    14    16     1
Direct
  0.8991580022652778  0.0952563141114898  0.3327293027226802
  0.1324365323794223  0.2490110302230379  0.0603876159260409
  0.1178275668063214  0.1191624189540932  0.0014653739518233
  0.0857801187684568  0.3302676720126876  0.2878606489665239
  0.2391430303373327  0.1519695363622492  0.5635484517809228
  0.0989911139552025  0.1417034421658382  0.7674360425246187
  0.3104975243045083  0.1121997589638581  0.2908616808495797
  0.0355557617555751  0.5689240900993163  0.1498705447924445
  0.9995287048624578  0.7895192565514141  0.1172480830755855
  0.2707631377272456  0.3081112077314566  0.1013864196520647
  0.5472893167119163  0.0610574094601626  0.2463512721572874
  0.7678505747195145  0.0223757365042058  0.0998570099270278
  0.2759893653576453  0.2877825184038700  0.3300624369849421
  0.8377564861334056  0.7729344539112708  0.9494284931739869
  0.8776202444088050  0.9168696023096412  0.9894689973199380
  0.8848298325595444  0.6912746561214156  0.7249585004990350
  0.7373464596280707  0.8657666392122237  0.4469688059713613
  0.8912074592196075  0.8970553131062372  0.2265917476270507
  0.6647819392646713  0.9072529612977340  0.7123421407184430
  0.9397591513452125  0.4550087524732741  0.8599411150537560
  0.9911046937970811  0.2459274334971752  0.8758519045128395
  0.6929784817207646  0.7154264988079240  0.9115826216576868
  0.4271797160382040  0.9550533430659588  0.7620319937574680
  0.2226375546628515  0.0134202975919923  0.8956439023485875
  0.6979767914249029  0.7339061444235110  0.6779986790560013
  0.8783249121361302  0.5260636059117560  0.1039494975249857
  0.4920214530102888  0.9074287005588445  0.4928788833023493
  0.0736569837974926  0.4928357144495226  0.9213484762981029
  0.4782335818395819  0.0978014144579127  0.5144211951207742
  0.9260868913513167  0.0659244863337300  0.4967564581068615
  0.0904463314206901  0.9199650372259913  0.5488514076312683
  0.1131511799655962  0.8845546644341732  0.7199724164679259
  0.4734516128056603  0.5277554178956065  0.9436672119861309
  0.3102567672897957  0.6875890712344797  0.9162509226933581
  0.5302322386175164  0.4612951445534527  0.1078182879788665
  0.6998617113266115  0.2949462548603634  0.1332772246004891
  0.7411687500012276  0.2636839150832252  0.2925528984962943
  0.2702908717806120  0.7186621797788572  0.7594984333601869
  0.1820249500202476  0.2012375486765851  0.9925967464510798
  0.0309915357014341  0.1439982097244568  0.1991281366346954
  0.2477298134076715  0.2996175137375883  0.0327174609611143
  0.0830535989275250  0.3602526337843075  0.9991839731832698
  0.7876117856600806  0.0117486525399118  0.6179733279272366
  0.1830119950799084  0.2018398639594756  0.6295107248681442
  0.1687449910162658  0.0112297925475039  0.6643050136877248
  0.2197441019022695  0.2270409955750231  0.4629921060129775
  0.3814704970320453  0.1694649472855787  0.5007180832506123
  0.0149527801736443  0.7922465907126888  0.6192367326944838
  0.0008849824710735  0.6532396997071256  0.1785356546164261
  0.1673130217429490  0.6313780801446781  0.0233519600247810
  0.0318122164883566  0.4423156160243686  0.2566452375950944
  0.9379259506725838  0.5496108751162886  0.1417363676946387
  0.5863471916925633  0.6110038711767792  0.8026907687348160
```

0.6293282885328776	0.0183300115976207	0.1754559119423179
0.6292429316736585	0.1776808714439521	0.1856899261427050
0.4407120164332562	0.0915246508815616	0.2345645699314570
0.4905955800238999	0.9552254548535537	0.3868831885181114
0.5865103345018546	0.5924876749704869	0.0455904722453662
0.8111628185007124	0.8430249500240322	0.9918960505152614
0.9707626138398985	0.8388645622945954	0.8239473477245380
0.7410913801713468	0.7782306214380459	0.9301913421293186
0.8230374836605340	0.6345484298284496	0.0517575106956252
0.2090756753020429	0.9817972884022218	0.4137675436953769
0.8153187435654183	0.8346609446716734	0.3633542658188247
0.7948917957134509	0.9932230516313466	0.3844738605430231
0.7230736003939173	0.7586915569186538	0.5701950588105512
0.6041763035448884	0.8568092726276737	0.4810460957784852
0.9727367754062606	0.2099255661853653	0.4117609498701689
0.9923765315412139	0.3812175978439751	0.8189203090684201
0.8184831045328831	0.3659410018691395	0.9968740696271152
0.9104892477419684	0.5642347738103488	0.7721838404012680
0.0368778942303345	0.5108235921993017	0.8469946968926770
0.3952717011513657	0.3988792622194763	0.2330860998957075
0.3586668248627516	0.0161660800270768	0.8152335895958110
0.3521532887584055	0.8152981892407566	0.8658239569378762
0.5591156591839090	0.9700992526167354	0.7259678323321956
0.4324936769234229	0.0165705879987337	0.6491759610174246
0.3974425823587762	0.4134452270136940	0.9958443312512268
0.9734810600742002	0.5168731914902894	0.0061319331248445
0.4889207893902879	0.0131923558732328	0.4906272071856429
0.7648378751653456	0.4051909361190920	0.2251465875676860
0.0546029617654059	0.1948022270986872	0.9381556582769062
0.3701159387717881	0.7951876373505513	0.6174042167629227
0.1732526145564658	0.0775497774998798	0.8189414555912600
0.2029880763943303	0.5902679441475914	0.8255432034465482
0.9416511783507626	0.8337345959996647	0.0499737905675806
0.6045193562224850	0.1975779631940969	0.4187879686829387
0.8116909843736472	0.9630123763789643	0.1804878930139326
0.0168392994520183	0.9954749977481389	0.1384302461378003
0.9328110565108723	0.0352931106009424	0.4295997600246352
0.8386495707555180	0.2128908446597023	0.3119029565927288
0.9769433552035434	0.0408538342270664	0.8517776832984991
0.1375117701004453	0.8972610434008850	0.6180394521535481
0.1728666112957806	0.7897735582517366	0.7800264208696879
0.1364446927638770	0.8759767618622588	0.0180307035216417
0.3834732895406369	0.6043748642080345	0.9643097055640204
0.3355260946819300	0.7012378421739774	0.8128706900246365
0.8533066608916486	0.1598896756660192	0.9757871376034679
0.6139434498558750	0.3792591522506450	0.1361373985058204
0.7402510984083512	0.2552926261997215	0.2117112854090772
0.2233266977576962	0.0860619535098538	0.9278815059764822
0.0649637253198756	0.2468932998536268	0.7684016169474761
0.9196735525963333	0.7946942233106739	0.2201401101730049
0.7748598645839553	0.9455565721153021	0.0684859653108444
0.0071895257412413	0.0075718160554240	0.5434214080726365
0.5260992910475040	0.4778603999096062	0.0159817839532036
0.1602099411612257	0.3389519893720208	0.1755639326879829
0.3114218139279767	0.1892154190905963	0.3198198798941191
0.8026893950890200	0.6865688520357951	0.8351805823598537
0.6638730810384854	0.8443850395631752	0.6707281722108350
0.1786891758993190	0.3380610708455840	0.3067208156767697
0.2838686644732409	0.1836628948291621	0.1957041187591406
0.8161000597591729	0.7174895520254817	0.6725760821800211
0.6383944147421299	0.8030043416146166	0.8419714481304350

```
  0.2160299096463036  0.5698210356757994  0.5452851638074737
  0.3519478868202859  0.6850809390213405  0.5083722326702578
  0.3051786629139185  0.5234325350967580  0.5063260824883800
  0.4419034662134668  0.6396986530869067  0.4687354808727930
  0.4187706446875957  0.5590400007333376  0.4671324529526054
  0.2393853589268959  0.6506116952723113  0.5459852545402012
  0.5516280299732987  0.3847095074449710  0.5262399413072407
  0.6433402996738891  0.3345320921614370  0.4963572764321765
  0.4775901335695547  0.3457868902559371  0.6398608643503444
  0.6605449525427604  0.2454404393361039  0.5808142819770054
  0.4957792439174701  0.2565587122684379  0.7236986084118986
  0.5868584743083346  0.2068412797734024  0.6940664862177581
  0.5036094824713940  0.4502593623004427  0.3683873836587137
  0.6750245542959347  0.6212229407468295  0.2863758583629655
  0.6694618359179827  0.6931079927618038  0.2139998894480910
  0.7473058224929140  0.5863410109201899  0.2555581261074483
  0.6942935386914685  0.6554956531886872  0.3124514553845560
  0.6000839505352902  0.1363499789784472  0.7601194422155311
  0.1278494882779214  0.5419200939011287  0.5760855915891914
  0.3698339764979834  0.7477161542336757  0.5094185265743197
  0.2857870856413159  0.4600979977917004  0.5066203058617306
  0.5296406409423897  0.6675361496237815  0.4389648478245598
  0.7006227381541722  0.3641413117716146  0.4082573366110683
  0.4066453600781391  0.3850068242371232  0.6624274476954345
  0.7307591534386837  0.2055415860075929  0.5586347152874173
  0.4374156924959109  0.2253382335919725  0.8124128978679691
  0.4202373755202324  0.3755682511860871  0.4456963047288618
  0.4959830646197530  0.5232102168703904  0.2977362349502426
  0.5777339625400075  0.4188333388087209  0.3338287566033252
  0.1695515864816782  0.6870216348805673  0.5759303096096240
  0.5350447481187830  0.5026387024541167  0.4140504443590471
```

IWV-Al-T6

```
Al  Si O
   1.00000000000000
    11.6056398920140573    0.0835597568938375    8.7649673366017495
    -3.6825601025723138   11.3529798124118475   10.3282290959204754
    -0.2346490665193920    0.2412826202978807   19.0520352385695801
   Al   Si   O
     1    37    76
Direct
  0.5213004748102220  0.9116946210325226  0.4915820439283052
  0.1549092285794694  0.2378423073664409  0.0548068209572715
  0.1175141254582067  0.0944064592455881  0.0137837631696985
  0.1042403836806098  0.3139734121581393  0.2883899840703268
  0.2530965216421812  0.1379739172862603  0.5640893810933882
  0.1000343411113384  0.1075164740456447  0.7836939811669280
  0.3294573187244930  0.1005572187710671  0.2946274186529488
  0.0557075185082950  0.5541360185122260  0.1474676557456148
  0.0023936208807788  0.7602591780212846  0.1333192257395845
  0.2936554147801791  0.2909252671390850  0.1003537130676119
  0.5667031649651991  0.0530684958264723  0.2431775571999765
  0.7681389807406185 -0.0098575265372946  0.1150162589249292
  0.2967807397571918  0.2743843023078513  0.3287128617231773
  0.8636705245195165  0.7580997226627524  0.9451186796072309
  0.8752143255415038  0.8843804201749662  1.0096570409549925
  0.9068425522075180  0.6722566774201362  0.7249103595040400
  0.7565876437518086  0.8484850770835803  0.4491024518642459
  0.893394425749020 5  0.8665689907391229  0.2405348151598812
```

0.6785900153878156	0.8918772214548236	0.7170813115937968
0.9555230911811131	0.4326175068311271	0.8612196178091469
0.9941903793210104	0.2169675747197374	0.8889003599268499
0.7216218648750196	0.7005467869766885	0.9051388537415294
0.4442138038914362	0.9489902404840208	0.7583552558386472
0.2252274785589267	0.9841957989882852	0.9083380080835904
0.7194216921275778	0.7141973469757507	0.6791257754470243
0.9247045182403115	0.5149948090879425	0.0810005831252495
0.1162853299519971	0.4789235027432979	0.9010327612965338
0.5043372159097328	0.1055171908960376	0.5141627398503570
0.8998699779386590	0.0866355317711062	0.4645734475238646
0.8782945001670140	0.1204680317315057	0.2906327363446488
0.0665324867273907	0.9364974755082124	0.5182122646385103
0.0924933401104378	0.9086769134604684	0.6809058014844418
0.4617046694370674	0.5473217732458958	0.9015026083991455
0.2916317560828865	0.7136597120151904	0.8751656918950407
0.5189304052258790	0.4819445021511564	0.0649222591187060
0.6801758328151237	0.3191711837098942	0.0924423106151513
0.7170808468881081	0.2899395041763398	0.2521553639570187
0.2480658673683052	0.7421716676540339	0.7187463487454429
0.1823714338615527	0.1653832967820130	1.0146913675192619
0.0172907519261137	0.1799455225353212	0.1730553575450681
0.2445498226610779	0.2269239083809958	0.0835479512113759
0.1804599792990126	0.3785597261516428	0.9478053206947767
0.7745253198424346	0.0256395920444154	0.5964672545782486
0.1696183657673165	0.1655397357853350	0.6491619745768165
0.1888768264634435	1.0104016416759720	0.6261496276259152
0.2697082096333785	0.2434930482245409	0.4402862088035766
0.3789283533936368	0.1368745426803138	0.5409838092617968
0.0199766206940830	0.7938466512337646	0.6030103692849964
0.9995461034955215	0.6241676544370688	0.1923882171199790
0.1830595681876469	0.6436204698011501	0.0147654726033709
0.0782135949020338	0.4406358584959999	0.2394303933694111
0.9641114991833845	0.5067323433061519	0.1493600632731339
0.5982277144272447	0.6030973474484838	0.7800683015538367
0.6342820281968419	-0.0027699100179525	0.1830921499535801
0.6349120016549276	0.1965600564294786	0.1372715549796286
0.4327391789015981	0.0364499221113200	0.2806520245510777
0.5696562782242819	0.9943429746302593	0.3484336543647182
0.5937968493830866	0.5964626167120699	0.0139737890848171
0.8167637972686723	0.8049904091884693	0.0119861844238909
0.9677622418203145	0.8684014856716609	0.8034192546522193
0.7496356546747286	0.7171553198454252	0.9665313386347990
0.9171238084967388	0.6486662463528735	-0.0030249440760318
0.2023617369975439	0.9973215566503979	0.3952171096733649
0.8208122088171708	0.8058496248506570	0.3759915408593466
0.8301466452011584	-0.0101605545416421	0.3501387670367166
0.7828149497184106	0.7716716970106677	0.5463960169667188
0.6168567819459557	0.8267974932082438	0.5143062605232288
0.9751324422599456	0.2105774940340771	0.4001331649550057
-0.0144862970198182	0.3512310009974717	0.8298641975748934
0.8227922753260540	0.3745086342362040	0.9849503796665225
0.9642839530700297	0.5633251068564918	0.7535541036766313
0.0497599074334400	0.4415998609449094	0.8768464597012564
0.4070695061932762	0.3983124363693969	0.2067166697687784
0.3626215720679434	0.9846662480515315	0.8361685394075294
0.3594086378579873	0.8424948986814504	0.8075160066451170
0.5447403027915487	0.9036034547291754	0.7787857933173051
0.5064569642664815	0.0647824733468407	0.6181866397938952
0.3978163146390898	0.4163991382753315	0.9681941775091337
0.0220807238736163	0.4852849004054873	1.0017930607263956

```
  0.5227934615834618  1.0069068961659533  0.5087250363302454
  0.7943550763756266  0.4195665045858216  0.1772204525743410
  0.0562267289024849  0.1796979786312681  0.9506800191681049
  0.3764707142368237  0.8201604528138029  0.5930499224242114
  0.1810398741476373  0.0419113584194363  0.8276830986169060
  0.2177427239104857  0.6063485482426523  0.7766154082318288
  0.9414105703676495  0.8086905250361132  0.0688467839393712
  0.6121579515986251  0.2324210912317116  0.3878229048344488
  0.8122076504835751  0.9259073134376533  0.1962045795659253
  0.0160237163010866  0.9709615098703699  0.1496949020099000
  0.8686741660213222  0.1131818624961669  0.3841694772176967
  0.8041161202352392  0.2066687039733937  0.2468698930228837
  0.9739307771475862  0.0091649675583773  0.8727402791081611
  0.0631359254744167  0.9620331716823907  0.5897851674172274
  0.1417884341993394  0.7955704187248421  0.7082848877054641
  0.1399378087969305  0.8467348473161723  0.0326941762394806
  0.3848355348101141  0.6364326768391101  0.8673020409482537
  0.2332682123640888  0.7321109238694080  0.8170114497696103
  0.8605415747397462  0.1246623077668808  0.9897385017322782
  0.6098574809858510  0.4076382009236209  0.0424060171234917
  0.6571928803639416  0.3063722485897244  0.1945524559763753
  0.2192674469486457  0.0631615856596201  0.9369074659864391
  0.0739645870466406  0.2126510474789579  0.7852329274602942
  0.9279832430453990  0.7625394837376605  0.2376735302691934
  0.7681724728711656  0.9131151087256681  0.0861098161307003
 -0.0208542121888558 -0.0055325440346416  0.4815244257325187
  0.4648160567339550  0.5310679203968645 -0.0059079045686634
  0.1821712790644651  0.3150189605712669  0.1809966454305496
  0.3316652922009602  0.1653725130328118  0.3343605607151117
  0.8319727803316594  0.6655498010131561  0.8361650017085620
  0.6854545305259621  0.8159153644946219  0.6852839209379724
  0.1779756498478189  0.2916370547766867  0.3355255418013074
  0.3500996342759122  0.2034809174220810  0.1680419124305191
  0.8142422566663954  0.6635558283433908  0.7069412000742195
  0.7100205942774680  0.8239532182456804  0.8088238063432507
```

IWV-Al-T6-DPDMP$^+$

```
Al  Si O    C    H    P
   1.00000000000000
    11.6056398920140573    0.0835597568938375    8.7649673366017495
    -3.6825601025723138   11.3529798124118475   10.3282290959204754
    -0.2346490665193920    0.2412826202978807   19.0520352385695801
   Al   Si   O    C    H    P
     1    37    76    14    16     1
Direct
  0.5169187517984989  0.9177188974865693  0.4935296474962749
  0.1489832804053942  0.2453102805152220  0.0580139980025528
  0.1125293654095369  0.1032600426250833  0.0151831771418457
  0.0984935083106040  0.3216253947318687  0.2905199633043375
  0.2486810593364065  0.1467209294449224  0.5652793182715015
  0.0964440725542289  0.1184313610302785  0.7836930847911966
  0.3240635011617575  0.1082136957121577  0.2968558162721544
  0.0499368295212232  0.5614639638635476  0.1504470009374783
  0.9977753241280245  0.7691686146160380  0.1338549082590401
  0.2890962487319112  0.2997987366593790  0.1018052356687680
  0.5616128728796964  0.0594466392302234  0.2449669565058940
  0.7627564557063302  0.9975935113449794  0.1165960188280751
  0.2902185460166517  0.2816674081331226  0.3312609828943136
  0.8584840545357753  0.7661653417248199  0.9463466944536489
```

0.8725487907295896	0.8950901492814205	0.0080147560638810
0.9010475930463585	0.6807629861149559	0.7275985012005368
0.7514347309310333	0.8565525830807548	0.4507262530514956
0.8857668900223151	0.8742898270694173	0.2430179903623577
0.6731116864254364	0.8992359764677033	0.7201447466013972
0.9486361414133490	0.4405268756272612	0.8646046724416835
0.9910026199547327	0.2266841224081508	0.8892563132882071
0.7152115172425949	0.7070561496524206	0.9089105711783871
0.4393386184695651	0.9565886940136935	0.7607224849576513
0.2203440023396847	0.9925053313123338	0.9104930058108629
0.7144094972781920	0.7237016884024783	0.6801065157744418
0.9192003607965105	0.5232735008155723	0.0832956460653976
0.1099509512562838	0.4864379385884103	0.9039768185275567
0.5002630635565943	0.1125316773746711	0.5150420408078096
0.8949549208870292	0.0929222003474402	0.4668097365302707
0.8721371604534894	0.1278753450821172	0.2930910422340938
0.0624611692210254	0.9444173110201777	0.5198610914919567
0.0873030632423493	0.9176853046216729	0.6822648511670512
0.4578284541136665	0.5558351450973535	0.9020817089118314
0.2864402739246287	0.7204685748745999	0.8784170312557095
0.5130578303942737	0.4890426223476868	0.0672750526066825
0.6740625672483970	0.3265483326952133	0.0965277692446211
0.7122511635517361	0.2990580046102682	0.2542421100005255
0.2421096645058635	0.7502023806361463	0.7223848594904179
0.1768443953459667	0.1720723163608903	0.0183745841554577
0.0116332562204451	0.1869838800271685	0.1766217193287880
0.2396145676022682	0.2357777095721444	0.0851657454605821
0.1738823651929479	0.3856497180615887	0.9504267445223922
0.7706784659270605	0.0325970868334124	0.5993562223377685
0.1698121599649629	0.1779012850278737	0.6478608636954093
0.1812625539486150	0.0188014583339836	0.6307502047595049
0.2652526994554417	0.2521836004328818	0.4416249621188572
0.3754126981054157	0.1451310810514936	0.5410539318231837
0.0161034241726273	0.8010729634184681	0.6063517303596138
0.9927479259418457	0.6323440476610650	0.1944694502296768
0.1776032427801335	0.6496394387433071	0.0182321988801064
0.0707550602023280	0.4474543462261196	0.2436180995667335
0.9570311643524939	0.5145515154770116	0.1529062745666215
0.5940258918315345	0.6122843520692345	0.7799202361193278
0.6299265457768809	0.0086201443780268	0.1797577343383456
0.6304806477309619	0.2017838222440826	0.1440780537022553
0.4280226140349904	0.0450565189509268	0.2816272139052932
0.5624064622877470	0.9913228276175408	0.3535312549758938
0.5881421398885914	0.6019969504000464	0.0183034096209231
0.8129606017930615	0.8146340942992170	0.0118568054934193
0.9609503444590252	0.8759891997807746	0.8045492361818370
0.7426653020591855	0.7231098019201510	0.9711794741025795
0.9121018154020892	0.6571214599172878	-0.0010332112895692
0.1982128406316818	0.0032243824062379	0.3975965853840446
0.8157897173382186	0.8131435157476791	0.3781982397354525
0.8222400948588140	0.9992133935706883	0.3500674598732796
0.7796422351992451	0.7831874267607732	0.5462407411852929
0.6110212045927041	0.8319237885862721	0.5182417774156181
0.9694546049724638	0.2181644452833690	0.4007656056603428
0.9778939687170450	0.3592557366498598	0.8330997130293225
0.8167550154736275	0.3827369380328752	0.9888313695334345
0.9572571083138103	0.5708592535694270	0.7564841667024730
0.0446972913289069	0.4510710422299015	0.8776582659005876
0.3997664134225722	0.4060608333396877	0.2086430671211731
0.3578714641625280	0.9945785424368666	0.8362487838723742
0.3548888028272142	0.8484770198596386	0.8117225209742908

0.5406269016856970	0.9142350094080194	0.7803069211064814
0.5015035619393520	0.0701367167259072	0.6199093538763180
0.3943334436965096	0.4243197655903457	0.9695387172317942
0.0143360636142175	0.4911855802672854	0.0060026815718970
0.5219787357689719	0.0188150415979396	0.5032146654795859
0.7868594146776924	0.4288874286552136	0.1818268648426845
0.0580425369997521	0.1932940194253555	0.9456940071078839
0.3710172291818545	0.8271016318350843	0.5969080937632892
0.1756793087719656	0.0529381761239359	0.8297857752377228
0.2111351007388609	0.6143076615528013	0.7806190083529725
0.9373141354696825	0.8181403512318124	0.0686268717643689
0.6077713373786057	0.2412461720703426	0.3907676283317538
0.7993086020286038	0.9276018711109963	0.2044275141523890
0.0064633539242133	0.9819765003001614	0.1497242026916319
0.8605132925637596	0.1168080024828922	0.3894914086830353
0.7999970331192546	0.2170824441191201	0.2480069659625096
0.9711589231593774	0.0199045636015970	0.8712112140531205
0.0602359274420182	0.9722077531167930	0.5894375722557960
0.1376133173184746	0.8051009963215477	0.7092503141263359
0.1355294327270068	0.8545571785940266	0.0334935914571163
0.3793553699771033	0.6434817108374808	0.8692086100803045
0.2271839347211919	0.7410428616115601	0.8200704767198117
0.8596422805248932	0.1299939008734476	0.9927713741109244
0.6027591322482706	0.4129283459870250	0.0468785413023947
0.6509917981981862	0.3158906894653296	0.1977563354915428
0.2141575674613450	0.0686213056575213	0.9418920840001865
0.0699688936337971	0.2247765098246262	0.7836563947172140
0.9221552054640464	0.7713962732018540	0.2383743464476005
0.7641057294740784	0.9218883484601155	0.0858424702444230
0.9748087869876665	0.0013973298291202	0.4834119628762374
0.4620244476838345	0.5408205098796928	0.9935613266870601
0.1780958439976508	0.3251432092641505	0.1814811914340643
0.3251607412978533	0.1719952005675017	0.3379650227992987
0.8265756859332406	0.6732466869232638	0.8392704196104377
0.6781745173997797	0.8239986140547464	0.6883668848582370
0.1704664850687341	0.2974089196962075	0.3398289120530333
0.3440241903598171	0.2114842495631727	0.1708457992777088
0.8092286447839208	0.6741315520275685	0.7081353679652070
0.7033321929924169	0.8303625958050215	0.8128692089646774
0.2323589226519910	0.5995877337894829	0.5552210362806584
0.3990488948191293	0.7189527774140783	0.4837825998811570
0.3053536121852382	0.5373793073006309	0.5277542155675536
0.4729940971366548	0.6578692823033720	0.4553724295264105
0.4261971476912834	0.5669976883889573	0.4771262218337596
0.2790066224405858	0.6899270957868975	0.5334306893516666
0.5297369233692398	0.3713854986353859	0.5620634040403426
0.6095814528417440	0.3065847823490179	0.5461430922946817
0.4603385989940997	0.3478058082829409	0.6679254979139955
0.6205065190598585	0.2192018421609541	0.6360277662592735
0.4712643157543361	0.2593363893001057	0.7577705176436124
0.5514360277366067	0.1957554399955102	0.7414834654118144
0.4613330145360629	0.4219160784599550	0.4225802517932823
0.6689635676540261	0.5854477191891810	0.3075128885926386
0.6666167755834730	0.6546694764947595	0.2322585656273031
0.7259350941455378	0.5336842930617456	0.2903505040353376
0.7061383097273536	0.6270433553672450	0.3176849589488844
0.5595390434260203	0.1264787093557519	0.8119679559470344
0.1387811293689095	0.5771235737114698	0.5940908355552500
0.4344225737035057	0.7885927345415420	0.4682333700697571
0.2677714739265414	0.4664069996554182	0.5460166584872681
0.5665227670290784	0.6811428373956219	0.4166671905912743

```
  0.6619397315067534  0.3227596381323057  0.4651155044244755
  0.3986913802069427  0.3984987743706493  0.6796440282808396
  0.6820765551526873  0.1686784501827378  0.6239534723215701
  0.4161904036062224  0.2396402256721902  0.8407615284907953
  0.3716490634648516  0.3580676693817096  0.5053060888849503
  0.4570645341754908  0.4927830258834343  0.3488944562203018
  0.5210108014974487  0.3740006562766466  0.4008882964314424
  0.2212103803563735  0.7378315186690221  0.5554485847954321
  0.5197246762573705  0.4862548773291293  0.4438431662940587
```

IWV-Al-T7

```
Al  Si O
   1.00000000000000
    11.6056398920140573    0.0835597568938375    8.7649673366017495
    -3.6825601025723138   11.3529798124118475   10.3282290959204754
    -0.2346490665193920    0.2412826202978807   19.0520352385695801
   Al   Si   O
     1    37    76
Direct
  0.7626927398065999  0.2639328826081546  0.2748320518656345
  0.1397904907121867  0.2439847085044649  0.0503632201612061
  0.1184722139825234  0.1089796346926658 -0.0033662957110755
  0.0944984315192233  0.3278819621415987  0.2743634495328670
  0.2425364695348251  0.1494942110744000  0.5516516035803469
  0.1021176240594944  0.1259430512848922  0.7651926227499501
  0.3208076983189250  0.1117160688931570  0.2804026229639437
  0.0508041235990844  0.5702242816488664  0.1367672198223505
 -0.0082870718553081  0.7740019343334108  0.1225092911080616
  0.2815185228013968  0.3026967985149951  0.0897025400702700
  0.5526028307786849  0.0522181812810824  0.2494191415970987
  0.7674825757960539  0.0125492910656983  0.0966632149514428
  0.2811838827588115  0.2907440210715324  0.3177682608568971
  0.8470712929491967  0.7648406345633976  0.9414984872485204
  0.8710102473224314  0.9013725953878281  0.9937258872541792
  0.8983562854661754  0.6873282039351971  0.7057569659284153
  0.7475849137801422  0.8622212688466255  0.4387370153651273
  0.8939778571508938  0.8875392254151167  0.2220273239453201
  0.6720003038268713  0.9018565378216828  0.6999733676397104
  0.9379870763371192  0.4434031948361470  0.8505254387920952
  0.9914663671091271  0.2337484440984900  0.8715052556870557
  0.7076879023734705  0.7107589255279275  0.8946705402013975
  0.4357101274397046  0.9536869559021088  0.7469921983198289
  0.2229748409782475  0.9985205400114930  0.8932763046651662
  0.7055253261630633  0.7245996519555771  0.6696928890666262
  0.8996291505948353  0.5283940357439036  0.0875834846362855
  0.4941246400204194  0.9037427210610077  0.4936295349832271
  0.0747362092814585  0.4845663257318544  0.9122084534256291
  0.4806011851191810  0.1034851095506525  0.5023534081871570
  0.9383366031125773  0.0614369048509214  0.4826402291287297
  0.9186466377200987  0.0874115005713740  0.3213432299715382
  0.0992887399416131  0.9116430001386219  0.5380401348784430
  0.1232372393551321  0.8768282011045831  0.7093700497234772
  0.4850656304361220  0.5185007366845311  0.9346555384347272
  0.3206392330060414  0.6778447831696806  0.9095625479525911
  0.5387208380482695  0.4521302143492214  0.0997049322482565
  0.7133786553379449  0.2916504651442755  0.1205703564294768
  0.2739738177023825  0.6999454560776533  0.7580357489499990
  0.1816186386718218  0.1916510193248978  0.9883930174700768
  0.0396771952443829  0.1361498690189289  0.1925869897627122
```

0.2550455986003010	0.2878193609829419	0.0263028762232148
0.0884201228808127	0.3540838007892354	0.9950757712588396
0.7972376393315698	0.0077362532605897	0.5961516101617959
0.1746901321197697	0.1863127714464755	0.6293077951851884
0.1827875773922224	1.0078207817070961	0.6430189080519441
0.2200467465527249	0.2280749251942757	0.4523977318936492
0.3850100625540176	0.1762059033549406	0.4854978214578970
0.0288135268647663	0.7875500355271047	0.5991385983255535
0.9959430743179192	0.6384610076939894	0.1840422651397911
0.1938153990408652	0.6362008677664381	0.0304238392976398
0.0312334062158499	0.4329540123759563	0.2456411506662848
0.9906077855863988	0.5810088779417715	0.0832229111426577
0.6035738611370191	0.5965837598450645	0.7963765142924203
0.6308374593680461	0.0073256759835410	0.1797230572205269
0.6387132914417146	0.1606885420066872	0.1955461092185767
0.4521650034302873	0.0929424758297718	0.2284877741945298
0.4863423747538422	0.9445591306709120	0.3930043283047666
0.6005532781360188	0.5873605981178294	0.0261209402081687
0.8113209969853962	0.8343525655336704	-0.0138093459559391
0.9834668928734630	0.8334321946397246	0.8180883232881364
0.7579631012957010	0.7703830123599571	0.9141010330191255
0.8324920278768219	0.6280116664679323	0.0438609835772930
0.2195602976107759	0.9811091394635562	0.4014009087977457
0.8142009430511046	0.8293496645601376	0.3579170086347350
0.8338854178316776	-0.0250632793599458	0.3780814549492382
0.7175859542453374	0.7468577055987292	0.5679773824772069
0.6264084790592931	0.8907201944597652	0.4541645228940449
0.9872784977331648	0.2031251689356556	0.3996735830643254
-0.0043697666509636	0.3708243804094721	0.8083273415487831
0.8071167895526937	0.3587241743890722	0.9796027714574984
0.9216760629027106	0.5598443398912398	0.7537756013438857
0.0291916719417152	0.4866756978726682	0.8511640712113122
0.4028185147501653	0.3985194372949776	0.2219507813041127
0.3554720444367702	0.9854597408339656	0.8286376974099522
0.3788541918527470	0.8185014126537595	0.8192403393466177
0.5668036431298097	0.9649829041447456	0.7147568211004688
0.4388863219731107	0.0418010512988066	0.6319309921206211
0.4054296945739145	0.4119079621779113	0.9799316595250180
-0.0161792676507262	0.5205177973427800	0.9863532419954382
0.4640473321434854	0.9972818149118968	0.5094720435449608
0.8038684105499192	0.4044996301042398	0.2177575952985163
0.0531496581114779	0.1840460847703788	0.9358361513247622
0.3956033570325134	0.7731152126094727	0.6213634808518291
0.1877039582766538	0.0697254657139346	0.8044007693350542
0.2063564566882208	0.5848660433803232	0.8041901194186093
0.9222109654628359	0.8122497727428717	0.0671198961126970
0.6127756476908325	0.1900606105830675	0.3984930806831929
0.8198883752188759	0.9524419694409956	0.1701975643067656
0.0181957534909988	0.9867494556515688	0.1358972738901810
0.9593414942960812	0.0360680874146614	0.4049173175578964
0.8542187211158847	0.1854396441304769	0.3123248945434184
0.9804168962962178	0.0200504329574544	0.8579772501033620
0.1380407948968386	0.8817116044591506	0.6150664441755989
0.1890769446987416	0.7852792053365946	0.7625930892894602
0.1274565865255680	0.8654219586437676	0.0152557931681964
0.4054850073078409	0.6048120173804210	0.9456230718326353
0.3061868506520734	0.6528336742315080	0.8455977907468043
0.8523355468248611	0.1501743106040346	0.9724297703306292
0.6174171338468057	0.3668971054654869	0.1334848228139134
0.7834501703151371	0.2799992126470106	0.1650707367641541
0.2215632351084862	0.0728071048331171	0.9252629155265670

```
  0.0659238074396812  0.2292281337434809  0.7684077434459121
  0.9226133082505836  0.7803949504061424  0.2239075501975705
  0.7688249744396447  0.9391207999203878  0.0637919595496016
  0.0117534873761996 -0.0042853466136250  0.5364448879092673
  0.5282820964352096  0.4579338373022090  0.0159118545980168
  0.1705310681041296  0.3326090194677720  0.1642992035025678
  0.3124615624260840  0.1900396401187041  0.3103535637142302
  0.8159084320995691  0.6841733515530588  0.8148402363826258
  0.6677887832254168  0.8333292109386864  0.6643077999464079
  0.1873934292427055  0.3450214569730106  0.2890115362824881
  0.2997261700019765  0.1811076358742718  0.1811967340514171
  0.8313690783702160  0.7186496658257118  0.6498623146849711
  0.6536881706020018  0.8027979670558575  0.8253972948048481
```

IWV-Al-T7-DPDMP+

```
   Al     Si     O     C     H     P
   1.00000000000000
    11.6056394577026367    0.0835597589612007    8.7649669647216797
    -3.6825602054595947   11.3529796600341797   10.3282289505004883
    -0.2346490621566772    0.241282626986503 6   19.0520343780517578
   Al   Si   O    C    H    P
     1    37    76    14    16     1
Direct
  0.7508380381248170  0.2843101533597912  0.2709598952634753
  0.1351927719117607  0.2720928816819206  0.0359486502795478
  0.1065995819150839  0.1241617168813386 -0.0051113379620888
  0.0893014430307720  0.3513529182456824  0.2647857296069726
  0.2300255873890739  0.1771215270803505  0.5432660218757339
  0.0871951368581626  0.1415409113114811  0.7640161355912387
  0.3181704793347933  0.1374514210382326  0.2738935624168489
  0.0517084794418060  0.5944219447878624  0.1225448960531162
  0.9837648518418960  0.7930028079733593  0.1156949840380622
  0.2735817346141026  0.3228149119612981  0.0840460941399160
  0.5520170142784264  0.0771873012701222  0.2376384117879369
  0.7556587918088304  0.0277651386599633  0.0931212171872631
  0.2769562764108492  0.3192615125398074  0.3068695778848294
  0.8446636681928523  0.7787363549168802  0.9367521677940642
  0.8589631305119457  0.9175500817682966  0.9899989489843352
  0.8977288416411993  0.7112520887813180  0.6886554270564229
  0.7489112961393537  0.8862657061286073  0.4258109343330004
  0.8832120450698455  0.9012750682347529  0.2180941808123487
  0.6681060911848620  0.9228618677990544  0.6905532460923701
  0.9393603008924440  0.4718354458238961  0.8342664926807828
  0.9784459922475962  0.2493308907444959  0.8719358133843678
  0.7102430671639716  0.7316530437826551  0.8804395325378196
  0.4311729301167703  0.9773679006485398  0.7373653649589702
  0.2131042142873626  0.0175286573567304  0.8868845557393592
  0.6986331431961567  0.7445685613743294  0.6610257008785805
  0.9109869869418494  0.5488062201723841  0.0690627638563237
  0.5030150242250672  0.9434736249659230  0.4749631176522974
  0.0896693219011173  0.5215084810060532  0.8795535626818856
  0.4764587449868727  0.1420673770804572  0.4884968179272692
  0.9350653790936222  0.0873066539206364  0.4687592165048537
  0.9061113694547727  0.1102705253628573  0.3136581789296090
  0.0931172734847523  0.9336015619478462  0.5282692066216258
  0.1172836088945523  0.9026637645369412  0.6980146088211284
  0.4785334920954992  0.5408160669362502  0.9236350766454016
  0.3172606852586728  0.7035333607262454  0.8973575467823443
  0.5332210778950932  0.4735234649591146  0.0882654698812128
```

0.7102472370913063	0.3165847150185978	0.1084194712723945
0.2782784464590136	0.7364473041583293	0.7380925238428850
0.1678954578840565	0.2004828164264239	0.9935933676281441
0.0131757112869117	0.1950171088664570	0.1706223567392109
0.2449572320325451	0.2950244216033022	0.0297282208798624
0.1215105448873400	0.3981006080182254	0.9458955143723362
0.7924676952268844	0.0323831564866902	0.5799546147615233
0.1572882097830166	0.2066680341498859	0.6270753623848810
0.1717618044498852	0.0396796930519205	0.6220263063373024
0.2148978526466020	0.2653394088128775	0.4386636252519475
0.3694868639046710	0.2006869207870653	0.4868772959287214
0.0266473886707655	0.8145135733846830	0.5792081493968619
0.9876822150470981	0.6578615170110894	0.1745269116750942
0.1949954945465545	0.6596932222044979	0.0225369601922785
0.0251296631591249	0.4560932814655165	0.2357702972911769
1.0020716811493855	0.6081411701791226	0.0598814269298371
0.5996530714685214	0.6145524704427034	0.7871889516363553
0.6189508142232129	0.0207515153968458	0.1783163531385185
0.6504387362280470	0.1878908886805891	0.1684941827957421
0.4499466352842285	0.1205214976524052	0.2197832712218029
0.4891387224875939	0.9770146356425750	0.3805027188410047
0.6022315328294193	0.6083455791992328	0.0096154986249133
0.7998451367493613	0.8437959496638830	0.9873372964638440
0.9819593239916665	0.8545705148827554	0.8153936887160260
0.7602569171974659	0.7853552669814590	0.9045997568794808
0.8344428897671663	0.6413597261804529	0.0346220740627308
0.2186668726573353	1.0059184442757663	0.3938794303983195
0.8006934255479912	0.8441065123718975	0.3538670984352761
0.8552793786249248	0.9867280374220887	0.3607012049507116
0.7061782904551416	0.7692311445457581	0.5602033925326876
0.6380543852269711	0.9370292340103095	0.4301154744422790
0.9793975543040329	0.2281073371259436	0.3936313301110629
0.9861923251041121	0.3877446250405136	0.8050610476269103
0.8103426348281003	0.3974934505522149	0.9644749839935159
0.9237960894418118	0.5848594091772307	0.7348708902324963
0.0396087207472539	0.5210701185294591	0.8227632523190458
0.3988077734157522	0.4277623247521787	0.2083959950606012
0.3471511340762132	1.0071844518469655	0.8206509453766548
0.3719620116154326	0.8461069691089386	0.8035208292048337
0.5597841634501085	0.9824641130634149	0.7115758800731888
0.4456495011396541	0.0751857651286331	0.6157274069827670
0.3934569593119924	0.4377608036359003	0.9690327621824457
0.9935668381984262	0.5430709151424887	0.9664386150109328
0.4751341021904778	0.0424736697666385	0.4836387962353487
0.8246255561744729	0.4216310770092707	0.2009039280169909
0.0391101165647744	0.2012665689355128	0.9370383318793831
0.4083167336739981	0.8124217573369781	0.6061507046051892
0.1742730375708478	0.0827551769314965	0.8012660085175836
0.2148087079737503	0.6312542595289894	0.7691906783497751
0.9144080700942382	0.8334682542408722	0.0600074474046177
0.5991983071042707	0.2465351544598998	0.3813390643870923
0.8114961862252269	0.9661464707638023	0.1637383181952079
0.0085002842828671	0.9988507151053572	0.1322641446134806
0.9641361473994470	0.0747043034756648	0.3800090043435206
0.8070394036283350	0.1737015711070286	0.3411423939162250
0.9658492564312915	0.0373661619674934	0.8531993710152739
0.1187685227833290	0.8937388003836657	0.6172412897646182
0.1958990881447429	0.8241757363786775	0.7397396882525116
0.1194818689338266	0.8836729829052706	0.0108860100628403
0.4070652356998549	0.6346311735489900	0.9269381302293073
0.2944206372107451	0.6750436256745944	0.8397897285528703

0.8396052088367144	0.1664438118146223	0.9707717494354520
0.6056992888280577	0.3822318228373132	0.1277441816529726
0.7703749581782732	0.2989586650762632	0.1632539240673671
0.2113829491181476	0.0941467529253396	0.9169894342137619
0.0534791477488636	0.2427120074507723	0.7702954511596053
0.9117396910438070	0.7925719534938117	0.2219663103097017
0.7560425920953369	0.9558679620681630	0.0589290489785681
0.0063188572532469	0.0190986432320351	0.5242812087036124
0.5168580745106311	0.4758590971328660	0.0093061129614342
0.1606758138045146	0.3527464726177825	0.1576631052236641
0.3068826557772306	0.2131069273991801	0.3074086488578584
0.8180532530642728	0.7077325474849711	0.7980096115453001
0.6600573350198256	0.8514195132647351	0.6590373090293764
0.1836296890770326	0.3705852472791928	0.2766079450888230
0.2970101495561936	0.2080855152272545	0.1746858555397873
0.8266354435602460	0.7404811817667596	0.6369208923344993
0.6581174811636191	0.8285954831517472	0.8111971743677695
0.3555504801133887	0.3752378815062886	0.5825819231978168
0.3609928109118834	0.4217025754319808	0.6787745703291709
0.4617868898739051	0.3454327818414153	0.5573369593734190
0.4669203388243929	0.3919190939596695	0.6543386363366679
0.5177206363598935	0.3542312853086171	0.5930416501240028
0.3056868727098866	0.4137348100871790	0.6424951881370956
0.6394916270711316	0.1948119421312571	0.6913673843512823
0.7425892172997065	0.1624499511547223	0.6789441926425410
0.5268150689575108	0.1271449019941467	0.8048009334845954
0.7323911201060614	0.0627000467846526	0.7803726208316377
0.5178488028886447	0.0272244979004209	0.9057744073889165
0.6200964972332975	-0.0049369963318036	0.8937758758712089
0.7103186369989539	0.2925015518979249	0.4650838270931805
0.7668489020211275	0.4477947192143082	0.4762350525042578
0.7859556665655199	0.5242023442243724	0.3895056982074107
0.8499952013444122	0.4250957576947080	0.4600477890145023
0.7333580819296266	0.4707916804219627	0.5288571484114550
0.6132651435493010	-0.0831786218045897	0.9723552346930141
0.3118995125936830	0.3684657250862943	0.5550313551105960
0.3215997785986772	0.4512196606728969	0.7260054716191902
0.5007004051838033	0.3163907312290538	0.5096653869586445
0.5095742709606522	0.3978225693941131	0.6831619153535091
0.8301893514253813	0.2135556091245165	0.5905663922342533
0.4466601180778478	0.1511389914249033	0.8143579632016276
0.8118646417848356	0.0368680715304219	0.7711658420504766
0.4302353024794485	-0.0257835404911029	0.9940271555003228
0.6453763084077559	0.2158005661975232	0.5117882572113970
0.7269253668510431	0.3710197333443339	0.3800378101939164
0.7949303095606373	0.2722547419790929	0.4457971619239715
0.2232554656047311	0.4377859337091935	0.6610401904746289
0.6565610789878029	0.3210862512541093	0.5575571507637189

IWV-Al-T3-meta-DEBH+Benzene

```
  Al     Si     O     C     H
    1.00000000000000
     11.6056394577026367    0.0835597589612007    8.7649669647216797
     -3.6825602054595947   11.3529796600341797   10.3282289505004883
     -0.2346490621566772    0.2418282698650360   19.0520343780517578
   Al   Si   O    C    H
     1    37    76    16    19
Direct
  0.9026516763272705  0.6947943140067546  0.7061068732855000
  0.1431796817667681  0.2475244031077536  0.0552510062264184
  0.1241359626825035  0.1076148769527752  0.0070941255667828
  0.0992290593336163  0.3341299042331694  0.2817878699372890
  0.2470543399624690  0.1542211967479982  0.5608396414966311
  0.1031949895145170  0.1244769537827565  0.7769382143979333
  0.3321339140119159  0.1220161402914068  0.2815104747063446
  0.0534765752729833  0.5775848548467077  0.1425114499729788
  0.9964341865080738  0.7789309623999354  0.1310231480229717
  0.2810169550926950  0.3100164647811265  0.0977589741872387
  0.5570638978987560  0.0621366216270157  0.2501242834786732
  0.7719378732232344  0.0141660644802369  0.1064999996681694
  0.2882668681588361  0.2920577165472678  0.3270724263543158
  0.8504953347388008  0.7706784955285860  0.9464133230435504
  0.8750371961765042  0.9052452159082376  0.0010851098818918
  0.7422839902000481  0.8697721358883181  0.4454029907332757
  0.8977782970404066  0.8889799878432176  0.2325068109834156
  0.6773878913014105  0.9081887984151504  0.7080092988647186
  0.9470787300574132  0.4519262718064474  0.8491065873650758
  0.9965627000215882  0.2333128269397094  0.8843358526065834
  0.7098800567591788  0.7151808204811270  0.9024332335778285
  0.4468659118233598  0.9634502483621734  0.7479448205125179
  0.2261837771290817 -0.0023225147407698  0.9034404905077844
  0.7028889361696710  0.7353008659302734  0.6750349285316821
  0.9060613620143793  0.5307583967202034  0.0898103634015868
  0.4967333072655379  0.9173582172454821  0.4913483842579249
  0.0865070359770653  0.4917518026307251  0.9092868549020996
  0.4881648696076558  0.1146031123325435  0.5036749437654571
  0.9429226577574600  0.0742938927613989  0.4837730591961683
  0.9108916234618971  0.0988939858120055  0.3248163681635594
  0.1106404451241325  0.9235606078285665  0.5341796379956663
  0.1246747890229739  0.8872329041535053  0.7131782960883564
  0.4788923659469169  0.5285540083996426  0.9429408793053515
  0.3213250605507179  0.6909697168999560  0.9103784093125701
  0.5385836682680543  0.4609665420534388  0.1052924766747418
  0.7158809806601512  0.3004216518451884  0.1210966335637714
  0.7595765027124026  0.2742263538060927  0.2750745121674089
  0.2762568377176765  0.7109208079763712  0.7595840711001461
  0.1890232202808563  0.1860933641047418  0.0014622249973527
  0.0278667742016024  0.1498966444554739  0.1947535398209253
  0.2516615703960918  0.2846059035418649  0.0423731926565369
  0.1057359538796103  0.3623844461986892  0.9838280376768918
  0.8056145791599163  0.0108295175499747  0.6056381418210854
  0.1795146842146946  0.1919083514030205  0.6379414210246495
  0.1913877190439457  0.0116464459811853  0.6543346760162856
  0.2236481787296694  0.2300188844511384  0.4625870475617380
  0.3909338880193563  0.1853538714789423  0.4917208014022068
  0.0410802142954510  0.8021705895277559  0.5954624489600215
  0.9940735467332101  0.6418894225509733  0.1937822997431373
  0.1897292655839767  0.6607480388723730  0.0219934221890435
  0.0498722678882578  0.4486631206134132  0.2470394205430211
```

0.9799625227903619	0.5658095252376321	0.1098036824651762
0.5953582698469136	0.6104382678399961	0.8049157715479379
0.6353428024472342	0.0099448838116483	0.1882471118036291
0.6457848400281707	0.1671302442110763	0.1988004022634922
0.4673003008161447	0.1127680154659046	0.2156742690576794
0.4794564401247963	0.9602565629211466	0.3956372661145919
0.5970833999876738	0.5940827108431757	0.0365372156728584
0.8141595657794299	0.8304105667020186	0.0010462495854241
0.9843168357136138	0.8488526198999180	0.8190426025831161
0.7548667466157056	0.7731876953950363	0.9275588183251081
0.8462959237132498	0.6337964675000236	0.0400574987816376
0.2381907003704839	0.9892997543911456	0.4005539882570361
0.8202369035038338	0.8340431713307589	0.3669577474617740
0.8221079109436052	0.9861238511173352	0.3869810926905013
0.6985655850007499	0.7578630291729275	0.5798125876583663
0.6293730949824453	0.9018154710502949	0.4440122570522453
0.9824485079667423	0.2169394668098071	0.4058942166880186
0.0050909524123553	0.3711883657197216	0.8158555523523819
0.8192003627377524	0.3619535049665353	0.9845650593517825
0.9276487429395094	0.5582659999236363	0.7548408366656565
0.0361426365705425	0.4980709440940363	0.8504206411512656
0.4062022505075188	0.4028814937000204	0.2331198542925813
0.3603713902513898	0.9919762486332186	0.8315987937065583
0.3895259826108258	0.8301433371156736	0.8166912005359250
0.5747766549052220	0.9761486458114764	0.7202499519611227
0.4600010018567128	0.0559455115515778	0.6263275108484503
0.4014679744065365	0.4231488309974904	0.9844896557088483
0.9942855143850186	0.5139255997548308	0.9972263382040411
0.4809621881819721	0.0140776566430787	0.5025622887635519
0.7987320262686767	0.4067365553337788	0.2188786796009038
0.0546537433220068	0.1834270046115105	0.9515506531659709
0.3963144464582592	0.7903132422263218	0.6214594596467053
0.1818775148171324	0.0575309635527548	0.8228491494200930
0.2152118625202832	0.5946549550792977	0.8030300576850613
0.9362664161407449	0.8260305559579906	0.0658828589502718
0.6200626889886655	0.2110952549933673	0.3919742038662051
0.8245687787137391	0.9553706131592369	0.1797458884844341
0.0256840020738691	0.9837420885842518	0.1454811213115860
0.9530424831556008	0.0594725086187984	0.4025491578783148
0.8431412778824735	0.1997437077053522	0.3118180968098749
0.9781323673797541	0.0269343192271706	0.8623210458470825
0.1443508128745341	0.9048407440215454	0.6111482933261649
0.1827152878525028	0.7872820322994439	0.7722710864547973
0.1347827349870388	0.8634314166333121	0.0309422729381297
0.3925500031433543	0.6084353565835219	0.9618989626808163
0.3109075028021426	0.6658180099523205	0.8447942681074344
0.8571014948938328	0.1517667540420760	0.9817158102773123
0.6221478915136244	0.3765775433503133	0.1318027198773980
0.7782320754506160	0.2853272984529173	0.1757325169766787
0.2269296976030396	0.0774880739203207	0.9292732725250675
0.0720247928732550	0.2262555984542577	0.7826567637724640
0.9226234173182634	0.7801875489013258	0.2363059666373060
0.7727948404984205	0.9404499074450975	0.0739856539849320
0.0338413492150258	0.0201782931796919	0.5154247710650562
0.5189375944156218	0.4648927995977520	0.0273933930113665
0.1679578276402566	0.3382685921336276	0.1730305959759745
0.3281543691136479	0.1952350144198294	0.3153722481912253
0.8130997762039679	0.6866451509854476	0.8267428259534804
0.6680614375257790	0.8452957233177225	0.6699207239570379
0.1932087460851015	0.3417155625202558	0.2993991340465391
0.3036090687642705	0.1933857527155318	0.1875266226677270

```
  0.8298020704654937  0.7287509328090110  0.6468871429700531
  0.6534391297770238  0.8096030206373854  0.8356940401670924
  0.2612207416238396  0.5700847740087024  0.3366185120118205
  0.2083298643111040  0.5058271525187844  0.4559652079041092
  0.1964748005202610  0.5693704799890590  0.3038804414021393
  0.0897460100491966  0.4366148204709240  0.5464473976334674
  0.0759096341808307  0.5019923527455912  0.3949770183626312
  0.0216119297159806  0.4321016507596308  0.5173314974313199
  0.9046055623769109  0.3536142895846732  0.6064036612398025
  0.7964050954796560  0.4777323142849249  0.7069423235589463
  0.7192905006541785  0.3860186012338058  0.8316186872100710
  0.7667632481330265  0.4974139035231734  0.6400567092557090
  0.6123229978585325  0.3138526137394465  0.8900799158704155
  0.6587622175961859  0.4260863124997797  0.6987400640146666
  0.5817562874206731  0.3343010234424575  0.8234974426930973
  0.2542814632684525  0.6373600331988953  0.1757536821169686
  0.8475132596610617  0.2505785089040085  0.7334047289252431
  0.3722905767580876  0.7354843179145047  0.0806783732300491
  0.4041263248844469  0.7834295880661510 -0.0092244757247567
  0.3616185807611013  0.8007243889150909  0.0883490583857430
  0.4430885385462863  0.6990886754177279  0.0868374270129391
  0.8401675094025973  0.1698469049641550  0.7494158030616100
  0.8959860458746060  0.2455302913495355  0.7626420078806935
  0.7537446101730165  0.2458978361404753  0.7901576023696646
  0.1894691603280444  0.6754190928068192  0.1592693944379421
  0.2687702548845415  0.5724080395108647  0.1670702075556540
  0.2613182335161559  0.5103233417125247  0.4770267740453081
  0.3554628807056819  0.6230154210915364  0.2666972873688501
  0.0486589872300421  0.3872687884618451  0.6386717146840614
  1.0225699033577651  0.4992795080819181  0.3731989402966749
  0.8780674372186221  0.5346396772571929  0.6628391877135842
  0.7419218536935327  0.3715183196158241  0.8834506302689511
  0.8245508053292836  0.5720040292198550  0.5429131141477640
  0.5520943716734200  0.2424950310353058  0.9877197048730244
  0.6345215768676561  0.4436519838035079  0.6467111579686333
  0.4971244716364335  0.2796419994422280  0.8695768483008568
  0.8574689200679690  0.3612145491390521  0.5749815475137803
```

IWV-Al-T3-orto-DEBH+Benzene

```
 Al      Si      O      C      H
   1.00000000000000
    14.5438127517700195    0.0000000000000000    0.0000000000000000
     3.3510317802429199   15.4238224029541016    0.0000000000000000
    11.2960309982299805   10.5371932983398438   11.1561899185180664
   Al    Si    O    C    H
     1    37    76    16    19
Direct
  0.8916274789169849  0.6703400212503758  0.7249348217547420
  0.1316079102384803  0.2310222121246430  0.0727870922929594
  0.1063795633923800  0.0801846072209899  0.0347315917052400
  0.0869920195050133  0.3104337870270623  0.3038309487594338
  0.2299173427849081  0.1349603204274575  0.5821098304298439
  0.0876208237756892  0.0996625206501112  0.8014985572004510
  0.3194332005770558  0.0975511990757794  0.3073684272661486
  0.0454618990170974  0.5531457603761253  0.1621591070145197
  0.9851234610475810  0.7534841464917920  0.1526994089455911
  0.2676594165525288  0.2831911078547888  0.1232449701079152
  0.5488920016314371  0.0402351451541489  0.2692467457732757
  0.7561218502533510 -0.0141815528782223  0.1306037737922590
```

0.2763955173662027	0.2727851128006825	0.3469770533057852
0.8405071821723947	0.7379050897368182	0.9731827725309128
0.8610748465516143	0.8774905157038525	0.0251915806800737
0.7373407042438994	0.8466159310794994	0.4641041877379485
0.8816372546098454	0.8594317331959123	0.2575255572722675
0.6656317122901199	0.8827715518290278	0.7300153992424842
0.9398809294107754	0.4320273088270125	0.8681504766789673
0.9798568603328174	0.2066421850549991	0.9105015437806449
0.7015180178867807	0.6922224295389745	0.9213468130464586
0.4346786025413483	0.9406885554302301	0.7708106855697460
0.2136019059848220	0.9750296975388757	0.9256161361053700
0.6901421034906473	0.7075266544927454	0.6978457724930920
0.9032250118187667	0.5037180561691510	0.1077103495750000
0.4966100438677835	0.9054909916525362	0.5060339770869114
0.0883181585708229	0.4795930733006255	0.9151145048639229
0.4742228079996246	0.0989624929315768	0.5256901295937345
0.9341742554426611	0.0497845199996663	0.5036844443944597
0.8984731407899297	0.0742510427446901	0.3476713973755033
0.0976483346537824	0.8950017468440905	0.5582909328098737
0.1144669483646151	0.8630575311322677	0.7342453714018696
0.4706383889595798	0.5014310683196874	0.9639824048761199
0.3145367195953497	0.6671950495906908	0.9314034428141654
0.5293049369917815	0.4367692089888465	0.1255734463582612
0.7061849980195136	0.2770372457488682	0.1442004771956699
0.7416822659368442	0.2437223013526379	0.3074620355425681
0.2764429758008568	0.6973738906501818	0.7725883977708097
0.1675477329008530	0.1547442133073294	0.0350447818537419
0.0050769953825598	0.1550889699836332	0.2059906406477003
0.2370731127675609	0.2541060041628700	0.0708170894954715
0.1183366211189872	0.3545232589485275	0.9805324501899493
0.7949825915922619	-0.0145173067455523	0.6223733365207172
0.1612618883915867	0.1681623712188227	0.6627730229403046
0.1713915005870400	-0.0046680490376088	0.6679773999684271
0.2116191271314271	0.2181715420840843	0.4798376187800966
0.3720970909360869	0.1624490200940538	0.5189008986551572
0.0307737379926989	0.7785164495386587	0.6097987095248170
0.9847090862799946	0.6175996954938197	0.2129800927629037
0.1846267377995366	0.6307100782696611	0.0480610692988124
0.0322865388186919	0.4213993613046050	0.2717826498077459
0.9810865532434256	0.5485934348522110	0.1184711361198778
0.5873419851908711	0.5785928138859494	0.8259722394342706
0.6195109586319076	-0.0189153436073860	0.2135148511372390
0.6453067306270734	0.1482962653738956	0.2065010972303830
0.4548878478193683	0.0899508855783524	0.2406518283586070
0.4767044617083589	0.9425809667397618	0.4139739844366800
0.5897035926779751	0.5703447341344431	0.0542570630941216
0.8026341191185437	0.7983299803878768	0.0273777058724049
0.9771778238960589	0.8126048178043086	0.8502978750803172
0.7497965205729767	0.7461989634646790	0.9473285733622249
0.8319794535074597	0.5986528487526346	0.0683088811057371
0.2279695625898632	0.9635058832112511	0.4259263316592651
0.8004745943801299	0.8018530253663300	0.3934890773361439
0.8381581015506399	0.9508043570517367	0.4016788682391839
0.6838359246490824	0.7335058574712177	0.6019952983619495
0.6329834513218715	0.8996422316835380	0.4540824659302333
0.9729661340134825	0.1910180880715825	0.4298842629461294
-0.0111280649964095	0.3439320314792157	0.8437949446586245
0.8177097345082588	0.3501302141875685	1.0063236586587714
0.9156859535767830	0.5329655044549574	0.7755177712124057
0.0391014487780912	0.4862292923849457	0.8550831290801924
0.3961255065514897	0.3823751406875471	0.2509604962159805

0.3484483941848160	0.9691817428866456	0.8548576138062497
0.3768628235320013	0.8075419102207784	0.8394762045848129
0.5639266775415517	0.9519189811435105	0.7412194982482491
0.4460014441837959	0.0340611044033585	0.6501322075490001
0.3892184016428285	0.3957952593668413	1.0087569720300742
0.9905907498434205	0.4921566990316429	1.0105467383198723
0.4768096799329107	0.0040887790387872	0.5152282717593722
0.8067634289008201	0.3744234986186013	0.2393314340083956
0.0400014684073673	0.1567443033386411	0.9761442397012197
0.4023239709778837	0.7756458814394183	0.6373249070986322
0.1705066482871954	0.0380138435701386	0.8436359751031336
0.2127667547707828	0.5877363779767306	0.8095042231191554
0.9203824654919515	0.7998358514171664	0.0922578734981937
0.6012546022461434	0.2040393495954435	0.4147659584045608
0.8090368281082911	0.9231184731944083	0.2041349566015548
0.0064269978883627	0.9567092433182716	0.1729657273789650
0.9524538426524627	0.0423655341475418	0.4145284233604736
0.8015974669962758	0.1461019244010135	0.3661370940153219
0.9661474138609324	-0.0021618493780526	0.8870206129137846
0.1237728231280464	0.8662917334008751	0.6438440641832207
0.1891096137783709	0.7796368436190274	0.7800883735873104
0.1228576940010694	0.8397862904830569	0.0507904633334971
0.3910639425013656	0.5875823111510529	0.9766323726874252
0.3023518553911467	0.6426186746953088	0.8662013899736670
0.8406656075557686	0.1242619648462090	1.0090762580308839
0.6087157728554423	0.3492447842749751	0.1576848815062877
0.7563890260287935	0.2506607889342961	0.2121536316086066
0.2108173840317265	0.0493221711297513	0.9581582730725220
0.0530330819200653	0.2002192561511799	0.8086350798843828
0.9131849290298542	0.7522209510302366	0.2595984162008801
0.7578550306082351	0.9151765696891793	0.0949500637659450
0.0208906317798744	-0.0083421198173761	0.5403916458113956
0.5113251935979861	0.4385499995672675	0.0477439325463049
0.1554721884892661	0.3149720188755466	0.1948651933293883
0.3119502389279115	0.1702989350302619	0.3428666235980238
0.8036139304549226	0.6665552455449524	0.8420313153947208
0.6525774110364193	0.8133289386707162	0.6988766146235166
0.1831671103069765	0.3232655580723879	0.3168475853387364
0.2881519725136399	0.1672016212549881	0.2155310978415379
0.8194795240978312	0.7033088696779393	0.6648947754929401
0.6448387830928026	0.7892517127611124	0.8561023665172321
0.1430623918767778	0.8347203835100004	0.2837949838163842
0.0601702313404039	0.7797523744102977	0.4039193768407885
0.1768895639927079	0.7680764034031827	0.2525544763834334
0.0112953079204424	0.6572287479204879	0.4926525894678623
0.1294464388002028	0.6444536024430138	0.3393227794121164
0.0436467954788020	0.5871665432599662	0.4634336710919159
0.9917745180772307	0.4629729221937654	0.5551707608994079
0.7304522993822318	0.4838915814205202	0.6102322228218757
0.6485098905152344	0.3848721654286579	0.7329536681610386
0.8095897664577876	0.4676648398377048	0.5318347188263181
0.6457362809100161	0.2694258382748170	0.7773956707285397
0.8062575619173291	0.3522918206971850	0.5761374010663101
0.7245454448823112	0.2531052278954234	0.6991098862585217
0.1662714626445435	0.5729126154529884	0.3055115171150095
0.9149259398014723	0.3886433175459734	0.6841784413686368
0.2692619227596955	0.6419102094226204	0.1734117041850412
0.2905485249392396	0.5803926004308317	0.1603589489908387
0.2458273926038811	0.7111006626119651	0.1156087669100616
0.3506780234006736	0.6842935316037201	0.1438023247875354
0.9607340628213337	0.3244857357849109	0.7172063688864028

```
  0.8930320237619429  0.4357612308680484  0.7131646709501956
  0.8322329392972663  0.3314480071931356  0.7277291274784871
  1.0865776709983743  0.5299523424165147  0.3327818409918821
  1.1906321254400869  0.4988042836774726  0.3600872598086753
  0.0341281707582375  0.8315867269536090  0.4280509965572996
  0.1823578326568667  0.9315490593499124  0.2126766042615950
 -0.0538036437941591  0.6140802433901866  0.5859262007466507
  0.2416509957668370  0.8145091371581030  0.1582099299620640
  0.7332094743859934  0.5737715836906536  0.5751402265177180
  0.5869061232699342  0.3971529617833398  0.7942415501871921
  0.8714912655826644  0.5451298570054486  0.4357393249026488
  0.5815664341171940  0.1920711955512749  0.8733493518294767
  0.8661940323405091  0.3403697481466104  0.5142626520085134
  0.7213365484254251  0.1632879588840023  0.7339057877479648
  1.0169342281822744  0.4136727576624873  0.5281538293445062
```

IWV-Al-T3-meta-TS6

```
   Al     Si     O     C     H
   1.00000000000000
    14.5438127517700195    0.0000000000000000    0.0000000000000000
     3.3510317802429199   15.4238224029541016    0.0000000000000000
    11.2960309982299805   10.5371932983398438   11.1561899185180664
   Al   Si   O    C    H
     1    37    76    16    19
Direct
  0.9005166184768745  0.6619726010646989  0.7364917088793296
  0.1504378422261301  0.2236119838872678  0.0729866341165131
  0.1337398172267671  0.0839036547240531  0.0229410536826964
  0.1110377568899553  0.3077109197530752  0.2963165939464426
  0.2569438544104137  0.1252193073763876  0.5788631273878173
  0.1150158001639616  0.1074679793576157  0.7878803494850418
  0.3329994065763613  0.0848063613450704  0.3035574761238279
  0.0558067209693956  0.5442349796996210  0.1628223896230902
  1.0131589008116579  0.7571547540878434  0.1396380798707091
  0.2887014266062552  0.2795733665067131  0.1172031036283449
  0.5661880757328277  0.0430993838660470  0.2536218212456700
  0.7853914589787709  0.9927334715378061  0.1182239426164108
  0.3006902382914221  0.2581655818680649  0.3433802283994105
  0.8553985659452589  0.7439482221743043  0.9664528189682944
  0.8909296286295606  0.8843417584896375  0.0113942182856689
  0.7520825864171206  0.8446751175488496  0.4583272001281463
  0.9087678601192247  0.8650512954755667  0.2458901594745897
  0.6845338209112004  0.8852497415080276  0.7259396170012689
  0.9628711586119402  0.4265050911821335  0.8740724110638874
  0.0087222026582511  0.2135040837562683  0.8959353212663369
  0.7078700542953945  0.6951736818803969  0.9242928505342088
  0.4493398469152743  0.9299032476175934  0.7761856056765415
  0.2387607732546959  0.9798252904897699  0.9158896391846393
  0.7092097515881303  0.7066305513908215  0.6950324565896367
  0.9024798069143927  0.4976434558660943  0.1152240016816389
  0.5083516939579702  0.8944334858683137  0.5000766111268203
  0.0997216266360209  0.4707395330694089  0.9271200768780880
  0.4920001221438270  0.0745785610292379  0.5338235874387462
  0.9491392497765068  0.0465701670244977  0.5008612463731789
  0.9161600935158362  0.0734926970194899  0.3414667554187824
  0.1130324671206725  0.8912648231373501  0.5564530639017413
  0.1292436566532550  0.8594573646227287  0.7321397819638606
  0.4862420619951994  0.4980276091973369  0.9609126594251045
  0.3288561903428028  0.6634781281605713  0.9295166556063313
```

0.5520651537196399	0.4402687365852923	0.1162514158528895
0.7217100927837363	0.2728793068818066	0.1419984755150223
0.7508135412437167	0.2341995717416860	0.3104259101972941
0.2919666770996280	0.6955499347869178	0.7686500748116015
0.1959803134693551	0.1637254568143062	0.0178594617662031
0.0393185768061724	0.1238569772264687	0.2140242484760511
0.2630116826807716	0.2629526319930857	0.0542972229786122
0.1082891865439629	0.3362172274074057	0.0063794006389087
0.8136303632578622	0.9860777062465911	0.6242771705251519
0.1939532273343278	0.1689472644868588	0.6503819828374215
0.1939030113075164	0.9834199311518903	0.6753495947951702
0.2391487359780363	0.1991177739536802	0.4770395694874894
0.4000626638842765	0.1499512051211809	0.5147439176145370
0.0403255259317765	0.7690463164713379	0.6211396575365451
1.0115318531794966	0.6206204528177974	0.2010234235526987
0.1859524656559984	0.6211110834215519	0.0320006070282740
0.0633353094743333	0.4228478419957837	0.2641630051046871
0.9622364691293666	0.5146974401520478	0.1566556521001566
0.5946320731393149	0.5845285187037628	0.8187387417883925
0.6471808068831199	0.9866123541796209	0.1976349222443557
0.6505383855696244	0.1652068961203057	0.1824194284025789
0.4656721919371793	0.0737446387342041	0.2332459813913677
0.4995294977853337	0.9487185838200594	0.3965878335228534
0.5957110649365842	0.5729852697366646	0.0568838587511139
0.8265327305954169	0.8084895700504252	0.0148498064962545
0.9884662586925802	0.8172756641890365	0.8385069497863928
0.7562101083198783	0.7482414581903720	0.9508628528899854
0.8494027228137172	0.6067271327611815	0.0622993811848922
0.2402586483147748	0.9518188173040718	0.4245361625537097
0.8311327185500391	0.8059291568148576	0.3824752211096261
0.8276312514583355	0.9675223188002169	0.3940174809273129
0.7159169670860879	0.7377278500920236	0.5906148555526449
0.6340337209470734	0.8634688814051443	0.4653164960549658
0.9935320057689834	0.1902616824485431	0.4165202292555963
0.0166858988068860	0.3492833003893548	0.8360572412327001
0.8552125436536933	0.3312956369326324	0.0191193254102142
0.9182805532562257	0.5210687643476475	0.7999685940820803
0.0664742601026266	0.4893486994655075	0.8509488151817157
0.4203976823834796	0.3675115262210042	0.2476219717501994
0.3755225333953155	0.9810714542977633	0.8396034026424950
0.3875705730012678	0.7873155923407658	0.8758737633296292
0.5861319125896252	0.9581258267646107	0.7261409409495736
0.4418141740941963	-0.0083038514128794	0.6702947495538535
0.4179819428876545	0.3818504612760784	0.0083906039667133
0.9993462053778269	0.4895886628801190	0.0163579188728834
0.5015129509196949	0.9903989362341106	0.5100697775750903
0.7904448025820998	0.3749864702387471	0.2344679902726729
0.0681361257267393	0.1574924516762712	0.9631030719533271
0.3976132452270271	0.7723321552405273	0.6266337312529762
0.1920631415917735	0.0436981737173403	0.8370587449632464
0.2305474812153654	0.5643039409084940	0.8348788598640816
0.9542817877368232	0.8041057662179311	0.0735808177045225
0.6226330555468499	0.1673900947297461	0.4424255181905103
0.8338305501037767	0.9335133441935859	0.1955040171239120
0.0350468811785469	0.9602607595442848	0.1614203992280694
0.9491505419547133	0.0240898219955331	0.4284013080092914
0.8526291025129158	0.1774169356128500	0.3252532793052234
0.9926509163217875	0.0066316330980352	0.8732865703883196
0.1487942034802930	0.8767886416737982	0.6302092753232323
0.1893990013626410	0.7612778068780823	0.7896197493415543
0.1507612884379479	0.8430910501282569	0.0402356919362404

0.3886006372452413	0.5662967078950392	0.9882513961097009
0.3520439296433423	0.6803439578485477	0.8254572296516892
0.8691561060098913	0.1309744636043333	0.9950742076064816
0.6481804944075028	0.3692082385029122	0.1297601135175427
0.7350190054329466	0.2146797586215547	0.2440872232038159
0.2402158078536345	0.0532327171256269	0.9477012355323805
0.0813448549544694	0.2108728275854919	0.7913374744306721
0.9354401937911268	0.7592610851362400	0.2450626678526366
0.7897942797116235	0.9176780672452932	0.0863162850065697
0.0404362554817547	0.9912544232237590	0.5315485754265231
0.5393220641163199	0.4493360796612747	0.0319964026533153
0.1807297783204681	0.3180793443940398	0.1832270539149018
0.3375126960335774	0.1597562574522532	0.3330711518338332
0.8103294408641234	0.6700162816802882	0.8448211471534502
0.6790070800899274	0.8147489742722500	0.6925904154493333
0.2042503355145530	0.3072758435203459	0.3176429704265870
0.2947647938435068	0.1547194564310095	0.2168107323752786
0.8306639608429021	0.6850389969709811	0.6771162017917239
0.6525009522967125	0.7924340897671592	0.8572286458648892
0.1730408874342781	0.7293148453821523	0.2697156883022699
0.1142609935158882	0.7438529215513101	0.3505616787956333
0.1305915081591301	0.6270935203659898	0.3039949212734593
0.0114361268974819	0.6532379233719983	0.4668760445555814
0.0272458393760257	0.5377138335388891	0.4199195896834418
0.9676054963209749	0.5493496386179184	0.5029592015922866
0.8625272306776346	0.4513282718837611	0.6269388272164249
0.7246695681597081	0.4957905186843002	0.6362379138609872
0.6225397277831553	0.4119952730499162	0.7608112652022910
0.7336152776114372	0.4951742434192006	0.5573317819132015
0.5419735083285260	0.3253356681354636	0.8054059369141846
0.6527763574229657	0.4082819172720654	0.6026490568484475
0.5578532340312251	0.3227157553494532	0.7266361256326153
0.1649666338318353	0.8531987109886362	0.3120165045386929
0.8471922327076153	0.4254193160476550	0.7256173565229166
0.2802106675314168	0.8503016548273572	0.2990136442032763
0.3209398461974234	0.9337631478887095	0.2631238013646296
0.2607952358232755	0.7766766411509298	0.3864630026138343
0.3462336458842107	0.8377497544294109	0.2381556238286617
0.9300901246424431	0.4095158308601893	0.7156342921716655
0.8373941073902404	0.5006532533677266	0.7193930356126732
0.7715045984824418	0.3457438719475207	0.8146331935448825
0.0980610457081979	0.8599853545370744	0.3775441936867314
0.1843307037181940	0.9330315539437857	0.2255993729643776
0.2529340384930522	0.8001049596904740	0.1780883631202266
0.1773139074990584	0.6177619116333337	0.2398984967255099
-0.0355427299364074	0.6638113092981421	0.5302654080466445
0.9926693253043238	0.4583407597898141	0.4467605283209493
0.7585291160951493	0.5822569291801450	0.5994990791203377
0.6092419143350936	0.4177151699519145	0.8199787320920661
0.8084782664584775	0.5616999702639658	0.4617439077416083
0.4648848370847922	0.2616350322355261	0.9002096365012343
0.6618865015860704	0.4058570115476333	0.5429909079127422
0.4945723728338808	0.2536183515812570	0.7623817307694334
0.8474486459087552	0.3713837913488170	0.6453534751772305

IWV-Al-T3-orto-TS6

```
   Al     Si     O     C     H
   1.00000000000000
    14.5438127517700195    0.0000000000000000    0.0000000000000000
     3.3510317802429199   15.4238224029541016    0.0000000000000000
    11.2960309982299805   10.5371932983398438   11.1561899185180664
   Al   Si   O    C    H
     1    37    76    16    19
Direct
  0.8838146107970004  0.6645702148694607  0.7428386701374800
  0.1365695281756966  0.2278613179570443  0.0775812779527332
  0.1205828981530454  0.0877498110832876  0.0276264380759930
  0.0959971139736727  0.3113709977481108  0.3019717294637153
  0.2435417262298749  0.1291014778315570  0.5834494409746478
  0.1018420541659421  0.1110901410026696  0.7926943267074419
  0.3177624953065071  0.0874374602324701  0.3086618635890044
  0.0410905438494805  0.5489859187131960  0.1674454534860851
  0.9991963339194317  0.7610615762232612  0.1447159385324983
  0.2739806032288474  0.2829278423138573  0.1227266883032171
  0.5514496495854923  0.0467703273675615  0.2592695504021738
  0.7710331127793718  0.9960080455537923  0.1233412765948799
  0.2854470254685652  0.2611621155184079  0.3489065673733496
  0.8427930258575190  0.7487748293358877  0.9695060732407764
  0.8763776701011011  0.8883325455068354  0.0165283946075141
  0.7381787619319349  0.8492962999447151  0.4629873347739438
  0.8955058911026612  0.8694142319805112  0.2504526346862026
  0.6709673740710397  0.8889405318965192  0.7312348698975729
  0.9487846017049745  0.4304594223425269  0.8783085481268109
 -0.0050605794872646  0.2164913923141787  0.9015132171233345
  0.6926619970584913  0.6976231728469299  0.9307131555112041
  0.4368974071902678  0.9341817122698004  0.7800045224886487
  0.2255135129623951  0.9830990145345224  0.9206162406888734
  0.6932851771769684  0.7108181188136351  0.7003555755258225
  0.8892968505236337  0.5022216063399976  0.1186134247503651
  0.4947329779060070  0.8986385005742844  0.5046567071384187
  0.0857697754655785  0.4743534268577160  0.9313668510474489
  0.4787266485039085  0.0793415102222770  0.5377139026922649
  0.9350523180796013  0.0508578870297289  0.5065119841358315
  0.9025625483251126  0.0779028797432811  0.3463539461486528
  0.0989162691508259  0.8955919329749127  0.5610547455026328
  0.1167773898340947  0.8635671950245158  0.7358363860143272
  0.4704891069387531  0.5023535105877002  0.9671742302151529
  0.3141339636732327  0.6669953948742549  0.9354899963117440
  0.5377154321860889  0.4443640946518275  0.1216411929232819
  0.7074420702714991  0.2772079196987160  0.1467015912125333
  0.7378100129180573  0.2391205914868640  0.3141683012987475
  0.2783090695175861  0.6981501101606189  0.7740898309130831
  0.1822980286017579  0.1682305020423696  0.0222215548535814
  0.0263531029820348  0.1281178949782915  0.2191106416063073
  0.2491829347393788  0.2696309578332833  0.0574192161951349
  0.0929158164580493  0.3393920847099495  0.0114405940875805
  0.8004630878197638  0.9885245879446499  0.6314308401781872
  0.1795061841000622  0.1725673512989744  0.6553151148130670
  0.1819088305168738  0.9869749733824293  0.6796179439014388
  0.2248263033354718  0.2021188849433075  0.4823749984838775
  0.3866426977110806  0.1549069333862903  0.5191139661676772
  0.0225429277142903  0.7733402395435311  0.6287910487706778
  0.9947602026627196  0.6239838317968823  0.2070854246368153
  0.1715983888747899  0.6282891872030738  0.0368525725384951
  0.0491773256353853  0.4272417393995940  0.2681148481511639
```

0.9494584330188200	0.5194239444167326	0.1596112789542693
0.5760227598006846	0.5914919998774989	0.8245085069600074
0.6332881295433412	0.9906856263057150	0.2029705051517885
0.6353447176122341	0.1688746299898478	0.1885694762620283
0.4511992178029322	0.0774964021083341	0.2385402712218721
0.4840145907444322	0.9519073064690617	0.4024598359139561
0.5800535902454073	0.5763287172620937	0.0645876022222349
0.8138678899180847	0.8128123315190626	0.0184270982024232
0.9758387311963523	0.8222897430399106	0.8415118036460382
0.7434242930828809	0.7529354789459861	0.9541242228991187
0.8366818332773277	0.6115645204798987	0.0652677173527989
0.2254013209249383	0.9537417954513963	0.4294011432000882
0.8181921176435188	0.8110938987125603	0.3866365406593484
0.8140965162346430	0.9719989570215334	0.3988272807541678
0.6999801074967926	0.7425824272422861	0.5958594133988354
0.6215754154986886	0.8699787608898963	0.4672722371388794
0.9779640825997775	0.1945343529022035	0.4231876835926151
0.0031702353965117	0.3523946941403053	0.8413434054538749
0.8399556041550886	0.3361044974189747	0.0227186528395832
0.9059096580306578	0.5251980045823574	0.8020450341157326
0.0520212877045323	0.4936527088588558	0.8551674687027150
0.4057153952922053	0.3699726179290551	0.2529416329769015
0.3619186389758065	0.9838036831334867	0.8447770926191052
0.3767495617015917	0.7914267205420074	0.8782665927483043
0.5733465322564916	0.9627104840040611	0.7306753447786012
0.4295889051502842	-0.0034870265087877	0.6738517730487408
0.4029920837843077	0.3848084281477833	0.0160639307113652
0.9858394987310846	0.4940190332721247	0.0199126715866847
0.4874014433082754	0.9952070722253410	0.5141185035667105
0.7770206540970310	0.3799566109556106	0.2381625614014424
0.0544806583478831	0.1607511695189376	0.9682028186208677
0.3853464619107940	0.7756830316820664	0.6321365943806223
0.1795468350305074	0.0477920871327158	0.8409388624659184
0.2169157512163922	0.5677160220201021	0.8387658616791576
0.9385419218035862	0.8087209121344334	0.0801007741065125
0.6093669530932080	0.1723598730263221	0.4458425109893415
0.8204276812071822	0.9359701409584921	0.1999244987584630
0.0212129899007802	0.9649429806204088	0.1663842835902436
0.9343969829443950	0.0290091586614240	0.4339885874349652
0.8395475877987044	0.1824414143916698	0.3291014558727737
0.9788903199879520	0.0106026701288144	0.8786367529090098
0.1367370189714691	0.8816028905223399	0.6333309483539493
0.1759861888481578	0.7645113643320774	0.7939922324780445
0.1372842816128075	0.8461990220030303	0.0442445928987146
0.3706245746269801	0.5678942820068460	0.9976434815846437
0.3359320455244986	0.6808811556131747	0.8334966482202358
0.8553916609320709	0.1342738993983340	1.0000664924385507
0.6338667672692528	0.3733967432202911	0.1350033572635974
0.7230009776531779	0.2198209666853286	0.2471665525929822
0.2259152083843311	0.0559552962412481	0.9531911017509981
0.0681828859078978	0.2147724104708033	0.7960476187430502
0.9243181292909439	0.7634494342695413	0.2498389014954925
0.7741921285715666	0.9219743817007646	0.0907741375749433
0.0285438477634060	0.9973703572056253	0.5345207178765122
0.5266955980781135	0.4558983512070371	0.0353042275261248
0.1652158575669777	0.3197917133128357	0.1902953584180282
0.3201446612026155	0.1623678450728324	0.3390478230068757
0.7931635949705603	0.6692946192758665	0.8535488788490428
0.6666767384013587	0.8205878516331359	0.6956832549634548
0.1890931522248263	0.3121244159299158	0.3230876241661383
0.2800325283404121	0.1570837122060127	0.2215322921185033

```
  0.8130475871141445  0.6854981425002344  0.6851931832141994
  0.6373667273486485  0.7945701727135807  0.8633889468298978
  0.2188649989695850  0.6671167440551862  0.2456119224362043
  0.1877164218876186  0.6999654568903002  0.3074773284106437
  0.1458386920503635  0.5640674042168681  0.3028483887543897
  0.0860688575720846  0.6314686296652323  0.4277250658080935
  0.0419333139792148  0.4972955350484612  0.4201549563377512
  1.0116047542685114  0.5267649332514779  0.4858380903530974
  0.9061399564885964  0.4414306979398877  0.6120797023399505
  0.7728750836012249  0.5183088173076242  0.6255828731784187
  0.6771000903704736  0.4409713195753918  0.7513001174301428
  0.7590189399720083  0.5246506721695876  0.5540897257653894
  0.5767806729214551  0.3670888013909699  0.8040396044315478
  0.6585649109183841  0.4504267982845263  0.6075108244106606
  0.5678473701941720  0.3713873329881747  0.7323216673193609
  0.0666238013770135  0.6732583312641044  0.4859527441898780
  0.9052853885004645  0.4009131939097877  0.7094571707759939
  0.1448650518423680  0.6350444316515778  0.5262099345105714
  0.1338440490373299  0.6717276507259085  0.5625576051145735
  0.1201224993151826  0.5375241754440239  0.5955764542974633
  0.2397470647604832  0.6685265855081042  0.4494542880012589
  0.9858340879224212  0.3719005891422263  0.6952462233511382
  0.9110676761587913  0.4693457842748820  0.7090431126540567
  0.8260563328111376  0.3245338331093455  0.7980518380028637
  0.9723273174644333  0.6426137284815965  0.5619093883306007
  0.0922669386196933  0.7713452510342724  0.4196552798552924
  0.2996324734306419  0.7220393140372109  0.1525485745248550
  0.1692228230809731  0.5363823885807724  0.2564494432137841
  0.2443033733853086  0.7810677396724981  0.2621118955302725
  0.9831059244446937  0.4184113716346550  0.4643007986430441
  0.8309282071128437  0.5963819772717021  0.5833315139797773
  0.6843868179024706  0.4409685760336890  0.8049912902816680
  0.8304908434743821  0.5857527842152217  0.4576197044028537
  0.5037476383397052  0.3082917714327380  0.8999521236102552
  0.6494636235993905  0.4534796733350401  0.5530915961116772
  0.4887993965071791  0.3127311657558866  0.7743458814526036
  0.8633601869966536  0.3683003305001443  0.6358855446793875
```

IWV-Al-T3-meta-I1^{+}

```
   Al     Si     O     C     H
   1.00000000000000
    11.6056394577026367    0.0835597589612007    8.7649669647216797
    -3.6825602054595947   11.3529796600341797   10.3282289505004883
    -0.2346490621566772    0.2412826269865036   19.0520343780517578
   Al   Si   O    C    H
     1    37    76    16    19
Direct
  0.8940042447610306  0.6921249191190497  0.7110675929320172
  0.1347560839395804  0.2489653493487969  0.0584275056037571
  0.1155928659981200  0.1052235976767405  0.0135872442164747
  0.0926738501129804  0.3350390266187614  0.2849574068497472
  0.2394292925768774  0.1533338153944035  0.5648562046978476
  0.0934723532316581  0.1221354291587648  0.7833468121539007
  0.3210192243705847  0.1204567738863769  0.2876440941820781
  0.0439100366224764  0.5761187070130154  0.1479679408913555
  0.9864727463318489  0.7765056070587367  0.1372045504900780
  0.2739043929669125  0.3089820668690996  0.1019792798308685
  0.5477900525004966  0.0617536365274584  0.2549254628958546
  0.7614451684404853  0.0113739457375205  0.1142062201591694
```

0.2795433621563864	0.2935545868386859	0.3306829919899116
0.8430670786179113	0.7707449826843356	0.9507700976163151
0.8645902784672437	0.9037783804842930	0.0074763854804043
0.7343360309400850	0.8696218673476739	0.4492523965404173
0.8873346082659174	0.8862301954472960	0.2392907123960525
0.6701278265672398	0.9099492827917828	0.7119651403785513
0.9390438734365565	0.4497388469292906	0.8550226343357612
0.9891947401974984	0.2307596617933605	0.8902059609737880
0.7045720139417944	0.7142263971481446	0.9057914758277368
0.4376598195876639	0.9596618660461811	0.7557340318529713
0.2161333806146967	0.9945698402382676	0.9106527488495827
0.6930676511204372	0.7378855559158279	0.6794349853186827
0.8993305497459639	0.5306745159020114	0.0924544667896015
0.4888982302044592	0.9179532042443816	0.4955602239549398
0.0793991334772106	0.4929506542849315	0.9107397838193164
0.4798708516755237	0.1138769236163487	0.5097895048237452
0.9350303027815878	0.0716424122315583	0.4891895511505741
0.9015629042452896	0.0985852673195969	0.3303536167595822
0.1006738924146649	0.9169547090369455	0.5418634363803885
0.1159995157788039	0.8846402503052089	0.7187002079556822
0.4709014544275313	0.5260788883207799	0.9492681147379871
0.3117345899912214	0.6871337148060195	0.9187666352596290
0.5323458120494066	0.4605412721343766	0.1095855390249329
0.7072414971056100	0.2983388122004353	0.1285057953424283
0.7496358015988583	0.2729008314857573	0.2831905569009365
0.2679826703583932	0.7103206694681448	0.7654547286859666
0.1791291996105897	0.1820527523680615	0.0103493031648866
0.0193607430325155	0.1552283288957690	0.1983353636519710
0.2434612056528493	0.2892891194824622	0.0436217368347712
0.0981138748422216	0.3625759537686949	0.9832530792130123
0.7974875306872184	0.0132750228805285	0.6095513511888048
0.1690786270049865	0.1866905078658719	0.6455167298735907
0.1872337295383551	1.0117093150951455	0.6516235340996239
0.2176848277386149	0.2318531931224684	0.4653166064736906
0.3825601290860926	0.1843378794326073	0.4983153100203274
0.0350707729823271	0.7965806669674756	0.5974569508308423
0.9821096671894584	0.6397370613805120	0.1994069400120595
0.1810755026862608	0.6591165994494187	0.0292106934130357
0.0375631633045990	0.4454672099905778	0.2526798213755914
0.9757039968484303	0.5674171252972320	0.1092111643663718
0.5842512575566299	0.6132975297273637	0.8088571981222580
0.6246396379477307	0.0096690470170473	0.1925272944625756
0.6392284627454130	0.1667426110113324	0.2011270962805609
0.4570212251614567	0.1128095800421774	0.2219174898175378
0.4712257820711777	0.9597880023250623	0.4003426451623922
0.5929180139040985	0.5931704506159621	0.0401542861134701
0.8036903009806105	0.8280968349677501	0.0080860829299133
0.9775688890574370	0.8507526567373234	0.8245159096786115
0.7507078229449169	0.7747617919939898	0.9280142783718219
0.8391585413761097	0.6333905649339426	0.0428200612092237
0.2292279753540641	0.9864431625626193	0.4077974109621542
0.8113078659387234	0.8310702265888982	0.3736874384929858
0.8155246794882141	0.9868938662554644	0.3882936624857894
0.6911646999122617	0.7584246222009765	0.5840028981928106
0.6207037923984867	0.9001917849995562	0.4492595612717709
0.9801905061509810	0.2138948205180980	0.4080977770863679
0.9953910653780390	0.3683047213762522	0.8234388014519261
0.8129456926286707	0.3642278017969844	0.9905980520550757
0.9159812277440833	0.5521721232596617	0.7595947139575592
0.0306061606652015	0.5051776682901943	0.8474887743645497
0.3992638279957510	0.4027410951829570	0.2359667948838323

0.3500434489535807	0.9887005725676559	0.8384709142905915
0.3841148331465571	0.8251616196213696	0.8242914639665063
0.5659341839800515	0.9753360942408932	0.7267323594550953
0.4497900674835970	0.0497301294736640	0.6346897057644105
0.3997618680107388	0.4169093209685472	0.9899832187692590
0.9850686420698086	0.5118380185865768	0.0009749774209212
0.4769150201327003	0.0176159406816835	0.5023507195146488
0.7922542101347445	0.4068218213134628	0.2224589697785425
0.0498076068820112	0.1840986803874577	0.9544270559486138
0.3879049455156995	0.7919277853597895	0.6268790908159532
0.1713345476458674	0.0552152640468660	0.8302344306078300
0.2080684306238017	0.5949475555427165	0.8068262090806642
0.9255205437508893	0.8254710479370559	0.0723042938901881
0.6105776688503359	0.2123332939180224	0.4002320470468003
0.8097283306453563	0.9494250862153919	0.1912684704675176
0.0139885653817415	0.9828826010766939	0.1516490509527002
0.9423442591795568	0.0544333718928313	0.4104552750256373
0.8316529915902224	0.1969291451511820	0.3217535724003237
0.9673835319767005	0.0256365312816303	0.8688269210207300
0.1297477117251682	0.8923592941337473	0.6239511975224686
0.1734342812050113	0.7854215746532719	0.7794122448045552
0.1246456829555284	0.8605758235457822	0.0372887443031998
0.3798870969316620	0.6004367343547423	0.9726019639384340
0.3016554744717303	0.6636917753403164	0.8516910514173306
0.8494619017778262	0.1482133218418311	0.9912480189495290
0.6134847509401012	0.3741036471522290	0.1392409509024932
0.7663224481828653	0.2792852132739767	0.1870414188393250
0.2173892632836493	0.0733377506108691	0.9373916465468632
0.0624086005907923	0.2243625865505729	0.7884556681357822
0.9130451639342095	0.7787592579977537	0.2417356207782461
0.7622974655297938	0.9403835862751642	0.0791378861062556
0.0213415105423657	0.0107471898560830	0.5259503842685257
0.5169616245596977	0.4663145353550407	0.0282666690789499
0.1649919066380900	0.3420380730562759	0.1737955584431206
0.3154328728098464	0.1954543145856657	0.3201105438562276
0.8082086876909453	0.6846289101573348	0.8305136851291262
0.6630757193212298	0.8497474168743735	0.6703078503618292
0.1848651710172576	0.3449980547807910	0.3031112146811019
0.2905898495868643	0.1881334524221534	0.1952196804426547
0.8183639691611219	0.7237396374228702	0.6549777537776358
0.6481961654823698	0.8073388608598450	0.8384147474672777
0.6984119867412613	0.2491000609566113	0.8745350877831299
0.8208982226290248	0.2603005969942895	0.8062656172924381
0.6588242524691106	0.3496547572719583	0.8451229670867378
0.9049746500455232	0.3717935535840429	0.7093637713394039
0.7420630276305848	0.4616875026052452	0.7455282458220346
0.8698144789114987	0.4785616511707662	0.6756688082362883
0.9460177071945605	0.5952813486005321	0.5373937587838655
0.0799716276305127	0.6164157773282695	0.4680264223345927
0.1360263840035593	0.5543514946043818	0.4323988017336207
0.1514611552620939	0.7038927528417182	0.4322733818891247
0.2603937695558318	0.5791738004146610	0.3631078244390059
0.2767487219762164	0.7316729258449257	0.3604608501114511
0.3302512478166479	0.6677576445000346	0.3269376621010974
0.8994974424839617	0.5875584380628036	0.4868599119472913
0.3532804286508349	0.8335749989403684	0.3130404557872301
0.3920760521078881	0.9497730436683591	0.1884125206046608
0.8491622615731109	0.1809282347052390	0.8306440086816210
0.5635500481358994	0.3398064046804741	0.8987052609616907
0.0003121938188912	0.3813826574890997	0.6572039020967047
0.7127491299091198	0.5405874266924370	0.7191822423027388

```
  0.0825933682444613  0.4870717821414433  0.4575689848911832
  0.1091533200734407  0.7526881134474985  0.4596423173112637
  0.3029653623225471  0.5301686226700147  0.3362632301201662
  0.4277337411139173  0.6884370920949604  0.2712069060204079
  0.9254658454623050  0.6692809401785447  0.5329001987272093
  0.9052411370343317  0.5061768628645722  0.5030564829463334
  0.8056657341497289  0.5874355762343225  0.5283943075602997
  0.9531443086859455  0.6642959681852521  0.3898482019987993
  0.6325969242505070  0.1604256565325029  0.9519075915583155
  0.8937475025934107  0.5060510312416611  0.7094747413152427
  0.3135153315360445 -0.0243841054480800  0.1929866016452916
  0.4408407731399798  0.9381149906168212  0.1252949389931663
  0.4503062536051164  0.0228888110818576  0.1531895348592629
  0.3033827488061785  0.8465555684988605  0.3751015233467080
  0.4337713855022849  0.8125309090293729  0.3064977769758325
```

IWV-Al-T3-orto-I1^{+}

```
   Al      Si      O      C      H
   1.00000000000000
    14.5438127517700195    0.0000000000000000    0.0000000000000000
     3.3510317802429199   15.4238224029541016    0.0000000000000000
    11.2960309982299805   10.5371932983398438   11.1561899185180664
   Al   Si   O    C    H
     1    37    76    16    19
Direct
  0.8883623894608328  0.6900187328300078  0.7179386923794455
  0.1325766517928121  0.2498659185223548  0.0600930512991096
  0.1094057347128915  0.1012603267085500  0.0203372436263144
  0.0883950620033946  0.3316846525563789  0.2894408790794842
  0.2324642179987287  0.1511544984350395  0.5719693783336399
  0.0894657292160146  0.1218372353458874  0.7865695632698988
  0.3165724716526991  0.1164977730114960  0.2917634166433137
  0.0446579444586814  0.5746746682739963  0.1481380680106802
  0.9829612250003341  0.7734316709592310  0.1404026286784049
  0.2716679437673020  0.3077686272311220  0.1044372325114910
  0.5453080872647040  0.0602268261555947  0.2572661404054525
  0.7567973654863709  0.0064385485310225  0.1193298861013709
  0.2733460472772405  0.2865454129933296  0.3384428808886661
  0.8397233536485729  0.7647288601359369  0.9557503796311954
  0.8616572095641909  0.9019850838053122  0.0095339815757215
  0.7344330883898662  0.8661736674655435  0.4523714972132987
  0.8809942468252497  0.8785770759047828  0.2461262631964619
  0.6648630468921795  0.9064773370495898  0.7182933338252326
  0.9368860059363980  0.4511050385810695  0.8552259402997717
  0.9843358836819169  0.2284399721712728  0.8959476765717618
  0.6944715121840289  0.7091061752606932  0.9148628586468530
  0.4343903154505031  0.9607340110837301  0.7571738569162281
  0.2121689472445819  0.9933113449101595  0.9141738111363896
  0.6922709773876097  0.7347976934033780  0.6836947575981420
  0.9017551160303792  0.5276046641838642  0.0924604365121136
  0.4901938248383155  0.9203140817188582  0.4968913662191051
  0.0816580920225909  0.4944263528616007  0.9070474312902000
  0.4761095513840448  0.1169588357403485  0.5115539602735595
  0.9301543768526792  0.0703142071868086  0.4936526213763134
  0.8979386633579993  0.0952294575903708  0.3352937853589213
  0.0973216739445369  0.9193075468287861  0.5462829330785751
  0.1142390200901258  0.8853175640968662  0.7218512925724709
  0.4699330825610497  0.5250917253169098  0.9516868689243503
  0.3114987025078849  0.6884476469011195  0.9183359716101039
```

0.5249836439521468	0.4564815110715019	0.1174420354199028
0.7043217277214230	0.2987501769600634	0.1301728514902475
0.7445818085912315	0.2689219958623200	0.2881573499732470
0.2681530329890444	0.7109431614984087	0.7656391916248856
0.1711658979020253	0.1744381859636087	0.0214195944466276
0.0148301081911227	0.1663575404989798	0.1986075008511274
0.2425026588295746	0.2885574868923464	0.0449863193181857
0.1031721320318725	0.3650553579561777	0.9773032242407435
0.7928390982332673	0.0094331904367307	0.6149049082998780
0.1655032517282142	0.1908832145772616	0.6471254281593954
0.1776923736353940	1.0079697226852371	0.6676944457183799
0.2087014538417036	0.2226489508108458	0.4745323119409714
0.3760342485693838	0.1835151531807534	0.5026386090062562
0.0265597055574885	0.7938574307523646	0.6147914718674122
0.9791953753770820	0.6374903553993929	0.2000848181259369
0.1824386271050007	0.6576367801965890	0.0319397701564563
0.0364720814274729	0.4442284748788825	0.2544421324037645
0.9815931088696238	0.5664903837635873	0.1051438218860990
0.5881854311174934	0.6067668701671464	0.8132822252139315
0.6204810171936356	0.0055123843090821	0.1974460915577055
0.6369296238874775	0.1697108629786409	0.1975498180494342
0.4511973727912190	0.1054892984782475	0.2283007440091698
0.4726561607151444	0.9609890990949792	0.4020604566827297
0.5808069824794871	0.5900290113446991	0.0494247688614778
0.8028644810438050	0.8244653121215525	0.0104680081456173
0.9731364998863644	0.8432565885398375	0.8278174982699348
0.7447226575287008	0.7671693509058665	0.9364511856980439
0.8370519336543686	0.6278401809868285	0.0481286571496679
0.2214865419968050	0.9836736629444525	0.4109914246884363
0.8038107991014238	0.8215974411357654	0.3816616036700443
0.8269143717298971	0.9795305483974263	0.3860692035000103
0.6875131740917231	0.7563632844370063	0.5888892114046916
0.6233311706146273	0.9055502432002196	0.4496898102263268
0.9727638626470623	0.2131726645739769	0.4118756665625635
0.9889589446635076	0.3657398355657714	0.8281348835450829
0.8120987144132253	0.3681883919263677	0.9918161301549999
0.9134649123576160	0.5532118639808793	0.7605728143307687
0.0328150840704665	0.5062470561319763	0.8441658705041449
0.3928326935147990	0.3945627157534137	0.2459655644629420
0.3462669860903891	0.9877463367480388	0.8417705128775511
0.3798762841470009	0.8283991515864413	0.8218425306963694
0.5616833552552795	0.9731854674545558	0.7306096101256004
0.4490729486713514	0.0550156950771831	0.6345901286859682
0.3956401466289883	0.4182265758583265	0.9919297231250197
0.9857461230307424	0.5112271620917356	-0.0011063419446993
0.4756954157178476	0.0196081239060926	0.5045605704845104
0.7987377364182962	0.4016254237899300	0.2234145560982771
0.0492713136401551	0.1849126252696496	0.9557796610757158
0.3905409868485398	0.7933307321585125	0.6280995058039425
0.1693001948595421	0.0581591132175230	0.8317154617027723
0.2094375565085319	0.5976624025708288	0.8035860945904780
0.9185329888744221	0.8234063885359023	0.0786970236597932
0.6041197564288497	0.2200983789485171	0.3992446739471723
0.8014671131178578	0.9369127893183510	0.1999814699981645
0.0043238348754381	0.9801096501793164	0.1572939274839002
0.9391909634265839	0.0522500589464812	0.4147366585154469
0.8138086087053358	0.1808347179217954	0.3392962630725189
0.9668344690019107	0.0218970505389152	0.8708796462580141
0.1374615750387124	0.9076123888700197	0.6159717498677382
0.1735082311352905	0.7858938173023787	0.7792852191408631
0.1208739526541906	0.8586377685848809	0.0386529981270736

0.3849007594901089	0.6064766895564656	0.9670297093591591
0.2980847997934210	0.6611964576019561	0.8553009061803122
0.8452114448488363	0.1435763916820095	1.0003783197088938
0.6103247976861981	0.3739726621555653	0.1427705859007725
0.7609340094376638	0.2744158156515252	0.1926519349314747
0.2114416148882605	0.0677452824789538	0.9458461977709011
0.0535047839651281	0.2213130145531121	0.7951289867515805
0.9125196789106094	0.7724192578144864	0.2469949202138585
0.7595562179649399	0.9420018204848198	0.0776272132796170
0.0185453386211578	0.0135999835753078	0.5283112044982706
0.5083633645188113	0.4612934884704839	0.0372288691592864
0.1613671417245395	0.3378995204631215	0.1787032691853687
0.3108798656763736	0.1899758071362795	0.3257129939046322
0.7932395631034547	0.6756320752117642	0.8417332150971418
0.6573900324318078	0.8435338708635285	0.6790878096998447
0.1794568060393568	0.3388370408334639	0.3098583111132753
0.2905243330798251	0.1874791925234524	0.1957101093148992
0.8208212496632310	0.7275309071673222	0.6527729200742591
0.6413813111814620	0.8064711958802736	0.8455582240294404
0.6711442264660935	0.2809640361541419	0.8715817564971404
0.7871156051131536	0.2691322159341697	0.8264190160131450
0.6524296089802214	0.3930366177205408	0.8123418235903221
0.8856713487462411	0.3696544268620098	0.7236413676295027
0.7503695640793067	0.4936892551942853	0.7072640934162123
0.8724257240588906	0.4883904351360454	0.6608212738994176
0.9659061453113054	0.5856602579510213	0.5192996219401889
0.0955412374312171	0.5984700888884598	0.4702278016745097
0.1474651641032313	0.5124446008586929	0.4702701355837390
0.1672769894996235	0.6982819091573704	0.4203457568725159
0.2670872671438757	0.5221539340730663	0.4242338705932996
0.2874446902313450	0.7066262241925949	0.3745662588446225
0.3375365793935854	0.6199369571992674	0.3764099504252514
0.9353503096318004	0.5578968448005509	0.4706094674932829
0.1219179168406277	0.7995813886276077	0.4076154851027776
0.1241137259989453	0.8867098129855938	0.2981396688199272
0.7986845061495849	0.1809391224485998	0.8731163410810578
0.5617634251474546	0.4000191447364463	0.8484087701872393
-0.0239154866712336	0.3624110600745614	0.6895083365734702
0.7384687709907540	0.5809771926018454	0.6564099132446241
0.0938612160003624	0.4364611956154063	0.5058703858451240
0.3423684701206325	0.7837037018647407	0.3364890691194673
0.3047822067415576	0.4539512914788442	0.4255823262857191
0.4312806802871564	0.6289335294653327	0.3400897188522119
0.9474680344927766	0.6692694104658654	0.4943185111632709
0.9438718184799779	0.4706758873892273	0.4999281932108682
0.8431994075278249	0.5578046950463875	0.5011723600247008
0.9964580852497545	0.6261082241754747	0.3722710748770913
0.5937127492991484	0.2010255218873539	0.9544037651275347
0.8946372180077530	0.5200577248086342	0.6914749438878307
1.0683123118457003	0.8415712315869968	0.3040300859307994
0.2158106371307388	0.9261319651793608	0.2149196847048716
0.0901923557163057	0.9593992926072361	0.2909359670199423
1.0303379101004122	0.7663856282076893	0.4882629402232760
0.1776085549192908	0.8475757978765276	0.3993633498177590
